# Limonoids Containing a C_1_–*O*–C_29_ Moiety: Isolation, Structural Modification, and Antiviral Activity

**DOI:** 10.3390/md16110434

**Published:** 2018-11-04

**Authors:** Jing-Ling Ren, Xiao-Peng Zou, Wan-Shan Li, Li Shen, Jun Wu

**Affiliations:** 1 Marine Drugs Research Center, College of Pharmacy, Jinan University, 601 Huangpu Avenue West, Guangzhou 510632, China; renjingling_94@163.com (J.-L.R.); xiaopengnl@163.com (X.-P.Z.); 2 School of Pharmaceutical Sciences, Southern Medical University, 1838 Guangzhou Avenue North, Guangzhou 510515, China; mimowanshan@163.com

**Keywords:** limonoid, a C_1_–*O*–C_29_ moiety, isolation, structural modification, anti-HIV, anti-IAV

## Abstract

Five new limonoids named thaigranatins A–E (**1**–**5**), containing a C_1_–*O*–C_29_ moiety, were isolated from seeds of the Thai *Xylocarpus granatum*, collected at the mangrove swamp of Trang Province, together with the known limonoid, granatumin L (**6**). The structures of these compounds were established by HR-ESIMS and extensive NMR spectroscopic data. The absolute configuration of **1** was unequivocally determined by single-crystal X-ray diffraction analysis, conducted with Cu Kα radiation; whereas that of **2** or **6** was established to be the same as that of **1** by the similarity of their electronic circular dichroism (ECD) spectra. In view of the marked antiviral activity of **6**, its structure was modified via hydrolysis with alkaline KOH, esterification with diazomethane and various organic acids, and oximization with hydroxyamine. Finally, 18 derivatives, *viz.* **7**–**10**, **8a**–**8i**, **9a**–**9b**, and **10a**–**10c**, were obtained. In vitro antiviral activities of these derivatives against human immunodeficiency virus 1 (HIV-1) and influenza A virus (IAV) were evaluated. Most notably, **8i** exhibited marked inhibitory activity against HIV-1 with an IC_50_ value of 15.98 ± 6.87 μM and a CC_50_ value greater than 100.0 μM; whereas **10b** showed significant inhibitory activity against IAV with an IC_50_ value of 14.02 ± 3.54 μM and a CC_50_ value greater than 100.0 μM.

## 1. Introduction

*Xylocarpus granatum*, a true mangrove plant, belongs to the family Meliaceae, of which the main secondary metabolites are limonoids [1,2]. Krishnolide A, a khayanolide-type of limonoid isolated from the Indian mangrove, *Xylocarpus moluccensis*, showed moderate antiviral activity against human immunodeficiency virus 1 (HIV-1) with an IC_50_ value of 17.45 ± 1.65 μM and a CC_50_ value of 78.45 ± 1.69 μM, respectively [3]. Three other khayanolide-type of limonoids obtained from the Thai *Xylocarpus moluccensis*, *viz.* thaixylomolins I, K, and M, exhibited moderate antiviral activity against influenza A virus (IAV). The most potent one is thaixylomolin I with an IC_50_ value of 77.1 ± 8.7 μM [4].

**Figure 1:**
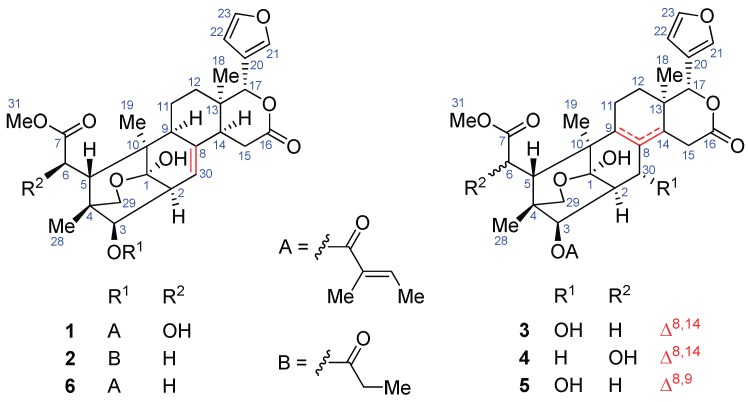
Structures of compounds **1**–**6**.

Limonoids containing a C_1_–*O*–C_29_ moiety are a small group of natural products. To date, only 34 compounds of this group, including xyloccensin L, xylogranatin E, granaxylocarpin C, granatumins L–T and V–Y, sundarbanxylogranins C–E, krishnagranatins A–F, thaixylogranin D, godavarins D–G and K, moluccensin W, and erythrocarpines D–E, have been reported from mangrove plants of the genus *Xylocarpus* and the land plant *Chisocheton erythrocarpus* [5,6,7,8,9,10,11,12,13]. It is worth noting that 28 compounds of this group were obtained from *X. granatum* [5,6,7,8,9,10,11]. However, only one limonoid containing a C_1_–*O*–C_29_ moiety, i.e., thaixylogranin D, was identified from the Thai *X. granatum* [11]. To search for new antiviral natural compounds from mangrove plants, seeds of the Thai *X. granatum*, collected in the mangrove swamp of Trang Province, were investigated to afford five new limonoids containing a C_1_–*O*–C_29_ moiety, named thaigranatins A–E (**1**–**5**), along with the known one, granatumin L (**6**) (Figure 1), which exhibited antiviral activity against HIV-1. To find promising antiviral drug leads, structural modification was applied to granatumin L (**6**). Herein, we report the isolation and structural elucidation of thaigranatins A–E (**1**–**5**), the structural modification of granatumin L (**6**), and antiviral activities of the modified derivatives of **6** against HIV-1 and IAV.

## 2. Results and Discussion

### 2.1. Structure Identification of Natural Limonoids ***1**–**5***

Compound 1 was obtained as colorless crystals. Its molecular formula C_32_H_40_O_10_, indicating 13 degrees of unsaturation, was established by the positive HR-ESIMS ion peak at *m*/*z* 607.2509 (calcd. for [M + Na]^+^ 607.2514). According to the ^1^H and ^13^C NMR spectroscopic data (Table 1 and Table 2), 7 degrees of unsaturation were due to 3 carbonyl groups and 4 carbon-carbon double bonds; thus, the molecule was hexacyclic. The ^13^C NMR spectroscopic data and DEPT experiments indicated the presence of 6 methyl groups (a methoxy group, a methyl group linked to a secondary carbon and 4 methyl groups linked to tertiary carbons), 4 methylene groups, 12 methine groups (including 5 olefinic), and 10 quaternary carbons (including 3 carbonyl groups) in **1**.

**Figure 2:**
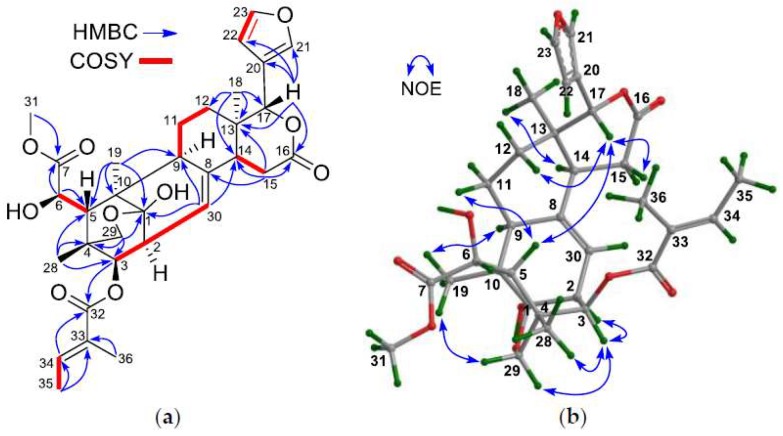
(**a**) Selected ^1^H–^1^H COSY and HMBC correlations for **1**; (**b**) Diagnostic NOE interactions for **1** (measured in CDCl_3_).

The NMR spectroscopic data of **1** (Table 1 and Table 2) and its 2D correlations (^1^H–^1^H COSY, HSQC, and HMBC) indicated the presence of a methoxycarbonyl moiety [*δ*_H_ 3.76 (s, H_3_-OMe); *δ*_C_ 53.2 (C-OMe), 176.1 (C-7)], a tigloyl group [*δ*_H_ 6.83 (q, *J* = 7.2 Hz), 1.67 (d, *J* = 7.2 Hz), 1.79 s; *δ*_C_ 167.2 qC, 127.8 qC, 138.7 CH, 14.6 CH_3_, 11.7 CH_3_], an oxygenated methylene moiety [*δ*_H_ 4.60 (d, *J* = 8.8 Hz, H*_pro-S_*-29), 3.45 (d, *J* = 8.8 Hz, H*_pro-R_*-29); *δ*_C_ 70.1 (C-29)], and a typical *β*-furyl ring [*δ*_H_ 7.50 (br s, H-21), 6.39 (br d, *J* = 1.2 Hz, H-22), 7.44 (t, *J* = 1.6 Hz, H-23); *δ*_C_ 121.5 (C-20, qC), 140.3 (C-21, CH), 109.3 (C-22, CH), 143.1 (C-23, CH)]. A *δ*-lactone ring (C-13, C-14, C-15, C-16, and C-17), characterized by NMR spectroscopic data [*δ*_H_ 2.31 (br s, H-14), 2.82 (m, H_2_-15); *δ*_C_ 36.7 (C-13, qC), 44.8 (C-14, CH), 29.6 (C-15, CH_2_), 169.3 (C-16, qC), and 76.6 (C-17, CH)], was corroborated by HMBC correlations between H_2_-15/C-13, H_2_-15/C-14, H_2_-15/C-16, H-17/C-13, H_3_-18/C-13, and H_3_-18/C-14 (Figure 2a). An olefinic methine group [*δ*_H_ 5.28 (d, *J* = 6.8 Hz); *δ*_C_ 120.2], exhibiting a ^1^H–^1^H COSY correlation to H-2 and HMBC correlations to C-9 and C-14, was attributed to CH-30.

The NMR spectroscopic data of **1** (Table 1 and Table 2) resembled those of granatumin L (**6**) [8], except for the presence of an additional 6-OH group in **1**, being corroborated by the downshifted C-6 signal (*δ*_C_ 72.5 CH in **1**; whereas *δ*_C_ 31.9 CH_2_ in granatumin L). ^1^H–^1^H COSY correlation between H-5/H-6 and HMBC correlations between H-6/C-5 and H-6/C-7 (Figure 2a) further confirmed the above result.

The relative configuration of **1**, except for the chirality of C-6, was established to be the same as that of granatumin L (**6**) based on NOE interactions. Those from H-3 to H*_pro-R_*-29 [*δ*_H_ 3.45 (d, *J* = 8.8 Hz)], but not from H-3 to H-5, established the *α*-oriented H-3 and the corresponding 3*β*-*O*-tigloyl group. NOE interactions between H_3_-18/H-14, H-9/H_3_-19, and H-3/H-2 indicated their mutual *cis* relationship and the *α*-oriented H-14, H-9, and H-2. Similarly, those between H-5/H-11*β*, H-5/H-17, and H-17/H-12*β* indicated the *β*-orientation of H-5 and H-17 (Figure 2b).

**Figure 3:**
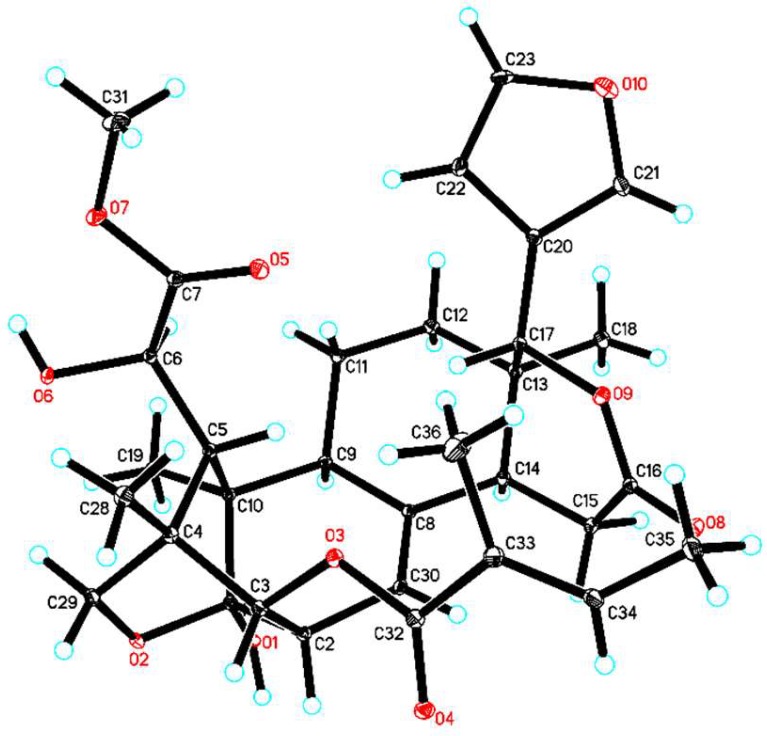
ORTEP illustration of the X-ray structure of **1**. Ellipsoids are given at the 10% probability level.

After considerable effort, suitable crystals of **1** were obtained in methanol/chloroform (4:1) at room temperature. Thus, the absolute configuration of **1** was unequivocally established as 1*R*,2*S*,3*R*,4*S*,5*S*,6*R*,9*S*,10*R*,13*R*,14*S*,17*R* (Figure 3, CCDC-1871152) by single-crystal X-ray diffraction analysis, conducted with CuKα radiation [Flack parameter of 0.02(3)]. Thus, the structure of **1**, thaigranatin A, was assigned as depicted.

Compound **2**, an amorphous powder, had the molecular formula C_30_H_38_O_9_ as established by the positive HR-ESIMS ion peak at *m/z* 543.2593 (calcd. for [M + H]^+^ 543.2589). The ^1^H and ^13^C NMR spectroscopic data of **2** (Table 1 and Table 2) were similar to those of **1**, except for the absence of the 6-OH group and the replacement of the 3-*O*-tigloyl group in **1** by a 3-*O*-propionyl moiety [*δ*_H_ 2.37 (m, 2H), 1.09 (t, *J* = 7.2 Hz, 3H); *δ*_C_ 174.5 qC, 27.1 CH_2_, 8.7 CH_3_]. The HMBC correlation from H-3 [*δ*_H_ = 4.84 (d, *J* = 10.0 Hz)] to the carbonyl carbon (*δ*_C_ = 174.5 qC) of the propionyl group confirmed its location at C-3. The absence of the 6-OH group in **2** was corroborated by the upshifted CH_2_-6 [*δ*_H_ 2.35 (m H_2_-6); *δ*_C_ 31.9 qC] in **2**. ^1^H–^1^H COSY correlations between H_2_-6/H-5 and HMBC correlations from H_2_-6 to C-5 and C-7 confirmed the above result.

**Figure 4:**
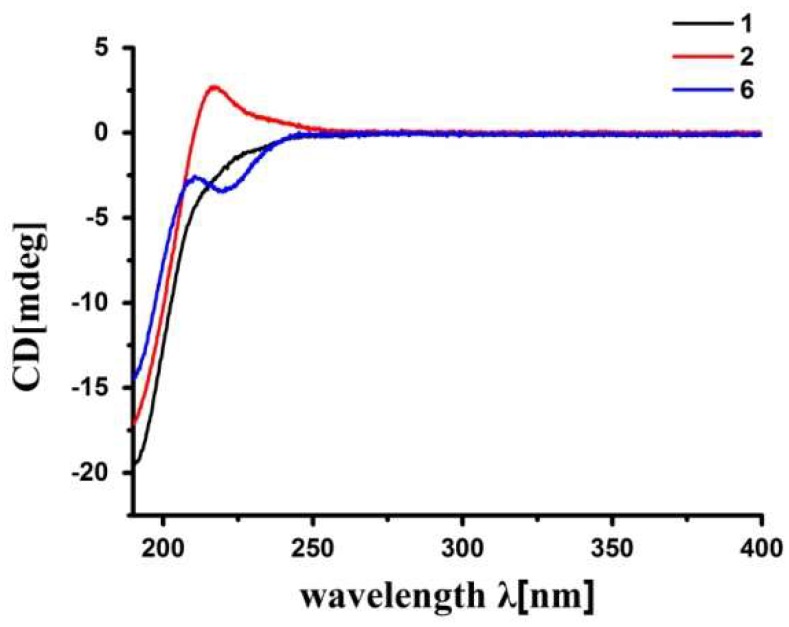
Comparison of the experimental ECD spectra of **1**, **2**, and the known compound, granatumin L (**6**), containing a Δ^8,30^ double bond (recorded at the concentration of 200 μg mL^−1^ in MeCN).

The relative configuration of **2** was determined to be the same as that of **1** by NOE interactions between H-17/H-11*β*, H-11*β*/H-5, and H-17/H-5, and those between H_3_-19/H-9, H_3_-19/H*_pro-S_*-29, H-3/H*_pro-R_*-29, H-3/H-2, and H_3_-18/H-14. The absolute configuration of **2**, except for the deficiency of the chiral C-6, was established to be the same as that of **1**, i.e., (1*R*,2*S*,3*R*,4*S*,5*S*,9*S*,10*R*,13*R*,14*S*,17*R*), by the accurate fit of their experimental electronic circular dichroism (ECD) spectra (Figure 4). Thus, the structure of **2**, thaigranatin B, was assigned as depicted.

**Figure 5:**
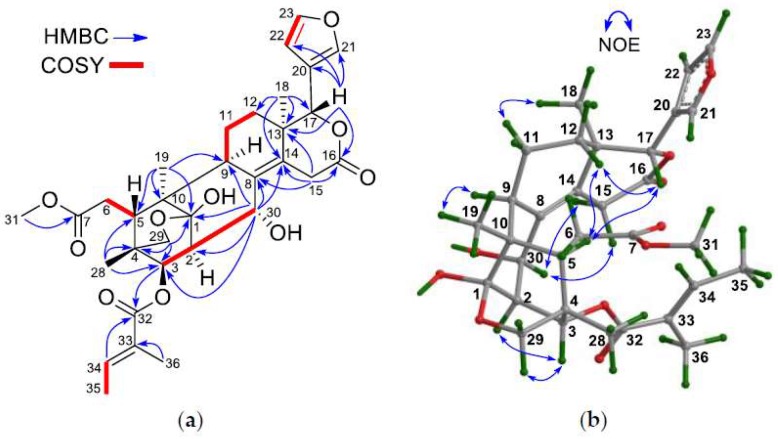
(**a**) Selected ^1^H–^1^H COSY and HMBC correlations for **3**; (**b**) Diagnostic NOE interactions for **3** (measured in CDCl_3_).

The molecular formula of **3** was determined to be C_32_H_40_O_10_ by HR-ESIMS ion peak at *m*/*z* 607.2512 (calcd. for [M + Na]^+^ 607.2514). The NMR spectroscopic dataof **3** (Table 1 and Table 2) were similar to those of godavarin D [12], the difference being the presence of an additional 30-OH group, which was corroborated by the downshifted C-30 (*δ*_C_ 66.3 CH in **3**, whereas *δ*_C_ 26.3 CH_2_ in godavarin D) and ^1^H–^1^H COSY correlation between H-2/H-30. HMBC correlations from H-30 to C-1, C-2, C-3, C-8, C-9, and C-14 confirmed the above result (Figure 5a).

The relative configuration of **3** was established by NOE interactions (Figure 5b). Those between H-3/H_pro-*R*_-29, H-3/H-2, H_3_-19/H-9, H_3_-18/H-11*α* assigned the *α*-oriented H-2, H-3, H_3_-18, and H_3_-19; whereas those between H-17/H-12*β*, H-12*β*/H-5, and H-17/H-5 established the *β*-oriented H-5 and H-17. The small coupling constant of *J*_2,30_, i.e., 2.4 Hz, revealed the *J*_e,e_ coupling for H-2 and H-30, and thus the corresponding equatorial bond for H-30, i.e., the *β*-oriented H-30, which was further corroborated by NOE interactions between H-30/H-15*α* and H-30/H-15*β* of equal intensity, and no observation of NOE interaction between H-30/H-9. Thus, the structure of **3**, thaigranatin C, was assigned as shown.

The molecular formula of **4** was established as C_32_H_40_O_10_ by the positive HR-ESIMS ion peak at *m*/*z* 585.2694 (calcd. for [M + H]^+^ 585.2694). The NMR spectroscopic data of **4** (Table 1 and Table 2) were similar to those of godavarin D [12], except for the presence of an additional 6-OH group, which was corroborated by the downshifted C-6 (*δ*_C_ 73.1 CH in **4**; whereas *δ*_C_ 32.2 CH_2_ in godavarin D) [12]. ^1^H–^1^H COSY correlation between H-6/H-5 and HMBC correlations from H-6 to C-5 and C-7 confirmed the above result.

NOE interactions between H-17/H-12*β* and H-17/H-5 established the *β*-oriented H-5 and H-17. In turn, those between H_3_-18/H-11*α*, H_3_-19/H-9, H-3/H_pro-*R*_-29, assigned the *α*-oriented H_3_-19, H-9, H_3_-18, and H-3. Thus, the structure of **4**, thaigranatin D, was assigned as depicted.

Compound **5** gave the molecular formula C_32_H_40_O_10_ as obtained from the positive HR-ESIMS ion at *m*/*z* 607.2511 [M + Na]^+^ (calcd. for C_32_H_40_NaO_10_, 607.2514). The NMR spectroscopic data of **5** (Table 1 and Table 2) resembled those of **3**, except for the replacement of the Δ^8,14^ double bond in **3** by a Δ^8,9^ double bond [*δ*_C_ 127.1 (C-8, qC), 139.2 (C-9, qC)]. HMBC correlations between H-15/C-8, H-15/C-9, H-30/C-8, H-30/C-9, and H_3_-19/C-9 confirmed the above deduction.

The relative configuration of **5** was established based on NOE interactions. Those between H-17/H-12*β* and H-5/H-11*β* assigned the *β*-oriented H-17 and H-5. NOE interactions between H_3_-18/H-14, H_3_-18/H-11*α*, and H-3/H_pro-*R*_-29 assigned the *α*-orientation for H-14, H_3_-18, H_3_-19, and H-3. The broad singlet of H-30 revealed the *J*_e,e_ coupling for H-2 and H-30, and thus assigned the corresponding equatorial bond for H-30, i.e., the β-oriented H-30. Thus, the structure of **5**, thaigranatin E, was assigned as depicted.

NMR data are shown in Table 1 and Table 2.

### 2.2. Structural Modifications of Granatumin L *(**6**)*

**Scheme 1:**
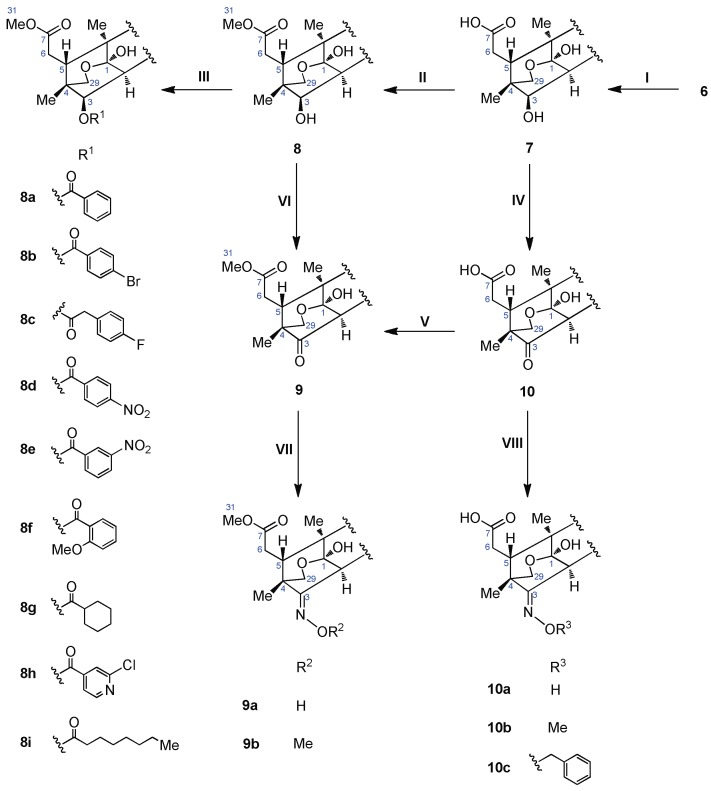
Summary of the structural modification approaches undertaken in this work. Reagent and conditions: (i) 10% KOH, CH_3_OH, r.t., 30.0 h; (ii) TMSCHN_2_, CH_3_OH, r.t., 2.0 h; (iii) organic acids, DMAP, DCC, CH_2_Cl_2_, r.t., 3–12 h; (iv) IBX (2-iodoxybenzoic acid), DMSO, r.t., 7.5 h; (v) TMSCHN_2_, CH_3_OH, r.t., 4.0 h; (vi) IBX, DMSO, r.t., 7.5 h; (vii) (viii) RONH_2_, Pyridine, CH_3_CH_2_OH, r.t., 24–36 h.

In our previous bioassay, granatumin L (**6**) showed an inhibitory rate of 67.10 ± 3.04% against HIV-1 at the concentration of 20 μM. Thus, structural modification of the natural compound **6**, particularly the bioequivalent substitution of the C-3 natural substituent, was investigated to find its potent and selective antiviral analogs. To achieve this purpose, 3 different protocols, *viz.* hydrolysis with alkaline KOH, esterification with diazomethane and various organic acids, and oximization with hydroxyamine, were employed (Scheme 1).

Compound **7** was obtained by the hydrolysis of **6** with an alkaline KOH. The ester functions at the C-3 and C-7 positions of **7** were hydrolyzed to give a hydroxy group at the C-3 position and a carboxyl group at the C-7 position, respectively, in the yield of 96.8%. Owing to the formation of the stable six-membered ring, the *δ*-lactone function of ring-D was not hydrolyzed. Then, **7** was treated with diazomethane to afford **8** as expected. Afterwards, the hydroxy group at the C-3 position of **8** was oxidized with IBX to give **9** with a carbonyl group at the C-3 position. Fortunately, the hemiacetal function of **8** was retained under the reaction condition.

Alternatively, **7** was first oxidized and then methylated to prepare **9**. An intermediate compound **10** was obtained in the high yield of 98.8%. However, the final yield of **9** was 76.2%, which made this strategy inadvisable. Finally, the hydroxy group at the C-3 position of **8** was esterified with various organic acids to afford a series of compounds **8a**–**8i**. The bioisosteric substitution of the ketone function at the C-3 position of **9** and **10** with different oximes led to the generation of derivatives **9a**–**9b** and **10a**–**10c**, respectively.

### 2.3. Antiviral Activity Bioassay

The inhibitory activities of natural limonoid **6** and all the modified derivatives **7**–**10**, **8a**–**8i**, **9a**–**9b**, and **10a**–**10c** against HIV-1 were tested [14]. Efavirenz was used as the positive control. The results were summarized in Table 3. The lack of substituent at the C-3 position of **8**, **9**, and **10** combined with the disappearance of their anti-HIV-1 activities indicated that the substituent at the C-3 position is essential for anti-HIV-1 activity of this limonoid skeleton. The oxidation of the hydroxy group at the C-3 position into a carbon-oxygen or carbon-nitrogen double bond did not improve anti-HIV-1 activity. For example, derivatives **9a**–**9b** and **10a**–**10c** containing a C=O or C=N double bond exhibited no activity.

Anti-HIV-1 bioassay (Table 3) revealed that the C-3 esterified products, particularly derivatives with a fatty acid ester at the C-3 position, markedly enhanced anti-HIV-1 activity. The derivative **8i** exhibited the highest inhibition rate of 99.95 ± 0.01% at the concentration of 20.0 μM; whereas **8g** showed an inhibition rate of 50.84 ± 6.96% at the same concentration. The IC_50_ values for **8i** and **8g** are 15.98 ± 6.87 and 21.98 ± 4.65 μM, respectively, among which the former is better than that of **6** (19.23 ± 0.12 μM). The results indicated that the introduction of alkyl groups at the C-3 position helped to maintain anti-HIV-1 activity. However, compounds with aromatic substitution at the C-3 position showed decreased activity.

Antiviral activities of all the modified derivatives of **6** against IAV were also evaluated [14]. Ribavirin was used as the positive control. Derivatives **8i** and **8g** exhibited no inhibitory activities against IAV at the concentration of 20.0 μM; whereas **8b**, **8c**, **8d**, and **10b** showed marked anti-IAV activities with IC_50_ values in the range of 14.0–22.8 μM (Table 4). The strongest inhibitory activity of **10b** revealed the importance of a C-3–substituted *O*-methyl oxime group. 

The above anti-HIV-1 and anti-IAV activities of modified derivatives shed light on the structural optimization of natural limonoid **6**, particularly suitable substituted groups at the C-3 position. In-depth structural modification and structure-activity relationship studies of the limonoid skeleton of **6** will be proceeded by us in the near future.

## 3. Materials and Methods

### 3.1. General

Optical rotations were measured on a MCP200 modular circular polarimeter (Anton Paar OptoTec GmbH, Seelze, Germany). UV spectra were obtained on a GENESYS 10S UV–Vis spectrophotometer (Thermo Fisher Scientific, Shanghai, China). NMR spectra were recorded on a Bruker AV-400 spectrometer (Bruker Scientific Technology Co. Ltd., Karlsruhe, Germany) with TMS as the internal standard. HR-ESIMS were measured on a Bruker maXis ESI-QTOF mass spectrometer (Bruker Daltonics, Bremen, Germany). Semi-preparative HPLC was performed on C_18_ reversed-phase silica gel columns (YMC 250 × 10 mm i.d., or 250 × 4.6 mm i.d.) using a Waters 2535 pump equipped with a Waters 2489 UV detector (Waters Corporation, Milford, MA, USA). Silica gel (100–200 mesh) (Qingdao Marine Chemical Industrial Co. Ltd., Qingdao, China) and C_18_ reversed-phase silica gel (ODS-A-HG 12 nm, 50 μm, YMC Co. Ltd., Kyoto, Japan) were used for column chromatography. ECD spectra were measured on a Jasco J-810 spectropolarimeter (JASCO Corporation, Tokyo, Japan) in MeCN.

### 3.2. Plant Material

Seeds of *Xylocarpus granatum* were collected in August 2012 from the Thai mangrove swamps of the Trang Province. Identification of the mangrove was done by one of the authors (J.W.). A voucher sample (No. ThaiXG-02) is maintained in Marine Drugs Research Center, College of Pharmacy, Jinan University.

### 3.3. Extraction and Isolation

The air-dried and powdered seeds (9.0 kg) of *Xylocarpus granatum* were extracted 5 times with EtOH (95%, *v*/*v*) at room temperature to afford the resulting extract (1363.6 g). After removal of the solvent under vacuum, the residue was partitioned between water and EtOAc to yield the EtOAc portion (307.7 g). The EtOAc portion (150 g) was chromatographed on a silica gel column (70 × 15 cm i.d.), eluted with a gradient mixture of CHCl_3_/MeOH (100:0 to 5:1, *v*/*v*) to afford 116 fractions. Fractions 40 to 50 (19.4 g) were combined and further purified by C_18_ reversed-phase silica gel column chromatography (72 × 6.5 cm i.d.), eluted with a gradient mixture of acetone/H_2_O (40:60 to 100:0, *v*/*v*), to yield 58 subfractions, among which the subfraction 15 (1.27 g) was purified by preparative HPLC (MeCN/H_2_O, 40:60) to give compound **1** (25.0 mg). The subfraction 17 (1.50 g) was subjected to preparative HPLC (MeOH/H_2_O, 65:35) to yield compounds **2** (14.0 mg) and **4** (1.0 mg); whereas the subfraction 18 (1.36 g) was purified by preparative HPLC (MeOH/H_2_O, 69:31) to afford compounds **3** (1.3 mg) and **5** (34.3 mg). Fractions 19–20 (0.59 g) were combined and then recrystallized to afford crystals of **6** (500.0 mg) in the mixture of methanol/acetone (2:1) at room temperature.

Thaigranatin A (**1**): Colorless crystal, ${\left[\alpha \right]}_{D}^{25}$ = −134 (*c* 0.1, acetone); UV (MeCN) λ_max_ (logε) 203.6 (4.46) nm; ECD (0.34 mM, MeCN) λ_max_ (Δε) 190.9 (−17.3) nm; HR-ESIMS *m*/*z* 607.2509 [M + Na]^+^ (calcd. for C_32_H_40_NaO_10_, 607.2514); ^1^H (400 MHz, CDCl_3_) and ^13^C NMR spectroscopic data (100 MHz, CDCl_3_) see Table 1 and Table 2.

Thaigranatin B (**2**): Amorphous solid, ${\left[\alpha \right]}_{D}^{25}$ = −110 (*c* 0.1, acetone); UV (MeCN) λ_max_ (logε) 200.0 (4.25) nm; ECD (0.37 mM, MeCN) λ_max_ (Δε) 190.0 (−14.1), 217.6 (+2.2) nm; HR-ESIMS *m*/*z* 543.2593 [M + H]^+^ (calcd. for C_30_H_39_O_9_, 543.2589); ^1^H (400 MHz, CDCl_3_) and ^13^C NMR spectroscopic data (100 MHz, CDCl_3_) see Table 1 and Table 2.

Thaigranatin C (**3**): Amorphous solid, ${\left[\alpha \right]}_{D}^{25}$ = −94 (*c* 0.1, acetone); UV (MeCN) λ_max_ (logε) 203.8 (4.14), 287.2 (2.97) nm; ECD (0.34 mM, MeCN) λ_max_ (Δε) 198.6 (−9.27), 219.6 (+3.7) nm; HR-ESIMS *m*/*z* 607.2512 [M + Na]^+^ (calcd. for C_32_H_40_NaO_10_, 607.2514); ^1^H (400 MHz, CDCl_3_) and ^13^C NMR spectroscopic data (100 MHz, CDCl_3_) see Table 1 and Table 2.

Thaigranatin D (**4**): Amorphous solid, ${\left[\alpha \right]}_{D}^{25}$ = −130.0 (*c* 0.1, acetone); UV (MeCN) λ_max_ (logε) 201.6 (4.50) nm; ECD (0.34 mM, MeCN) λ_max_ (Δε) 198.4 (−12.0), 220.2 (+8.2) nm; HR-ESIMS *m*/*z* 585.2694 [M + H]^+^ (calcd. for C_32_H_41_O_10_, 585.2694); ^1^H (400 MHz, CDCl_3_) and ^13^C NMR spectroscopic data (100 MHz, CDCl_3_) see Table 1 and Table 2.

Thaigranatin E (**5**): Amorphous solid, ${\left[\alpha \right]}_{D}^{25}$ = −12.0 (*c* 0.1, acetone); UV (MeCN) λ_max_ (logε) 197.8 (4.21); ECD (0.34 mM, MeCN) λ_max_ (Δε) 195.2 (+8.7), 220.8 (−2.4), 251.8 (+0.6) nm; HR-ESIMS *m*/*z* 607.2511 [M + Na]^+^ (calcd. for C_32_H_40_NaO_10_, 607.2514); ^1^H (400 MHz, CDCl_3_) and ^13^C NMR spectroscopic data (100 MHz, CDCl_3_) see Table 1 and Table 2.

### 3.4. X-ray Crystal Data for Thaigranatin A *(**1**)*

Orthorhombic, C_3__2_H_42_O_1__1_ (C_3__3_H_4__0_O_1__0_·H_2_O), space group *P*2(1)2(1)2(1), a = 12.1554 (1) Å, b = 14.2125 (1) Å, c = 17.2234 (2) Å, *α* = 90°, *β* = 90°, *γ* = 90°, V = 2975.49 (5) Å^3^, Z = 4, D_calcd_ = 1.323 Mg/m^3^, *μ* = 0.839 mm^−1^. Crystal size: 0.15 × 0.13 × 0.12 mm^3^. 36,359 measured reflections, 5945 [*R*_int_ = 0.0396] independent reflections, 400 parameters, 0 restraints, *F*(000) = 1248, *R*_1_ = 0.0398, *wR*_2_ = 0.1133 (all data), *R*_1_ = 0.0396, *wR*_2_ = 0.1131 [*I* > 2σ(*I*)], and goodness-of-fit (*F*^2^) = 1.100. The absolute structural parameter Flack x is −0.02(4), and Hooft y is −0.00(3). 

CCDC-1871152 (**1**) contains the supplementary crystallographic data for this paper (excluding structure factors). These data are provided free of charge by The Cambridge Crystallographic Data Centre.

### 3.5. Procedure for Structural Modification of Granatumin L *(**6**)*

#### 3.5.1. General Procedure for the Preparation of **7**

Granatumin L (**6**) (500.0 mg) was stirred in methanol (25 mL) at room temperature for 20 min. Then 10% aqueous KOH solution was slowly added, and the mixture was stirred at room temperature for 48 h. Thin-layer chromatography (TLC) was used to monitor the products. After the reaction was completed, methanol was removed under reduced pressure. The remaining solution was neutralized with 10% HCl to pH 5–6, followed by C_18_ reversed-phase (RP_18_) HPLC to afford **7**.

**7**: White powder, Yield 96.8%; ^1^H NMR (400 MHz, CD_3_OD): *δ* 7.74 (br s, 1H), 7.47 (t, *J* = 1.6 Hz, 1H), 6.50 (d, *J* = 1.4 Hz, 1H), 5.68 (s, 1H), 5.58 (br d, *J* = 7.0 Hz, 1H), 3.93 (d, *J* = 10.0 Hz, 1H), 3.83 (d, *J* = 10.0 Hz, 1H), 3.34 (m, 1H), 2.97 (dd, *J* = 18.8, 6.0 Hz, 1H), 2.93 (d, *J* = 18.8 Hz, 1H), 2.81 (m, 1H), 2.78 (m, 1H), 2.38 (m, 1H), 2.36 (m, 1H), 2.33 (m, 1H), 2.09 (dd, *J* = 12.0, 4.0 Hz, 1H), 1.82 (qd, *J* = 12.0, 4.0 Hz, 1H), 1.68 (m, 1H), 1.54 (m, 1H), 1.48 (m, 1H), 1.10 (s, 3H), 1.06 (s, 3H), 0.70 (s, 3H); ^13^C NMR (100 MHz, CD_3_OD): *δ* 177.7, 173.8, 144.2, 143.3, 138.2, 122.7, 122.3, 110.9, 98.5, 78.8, 74.5, 69.6, 49.4, 48.3, 46.1, 43.1, 38.6, 38.1, 35.9, 34.9, 33.1, 30.8, 22.2, 20.6, 15.6, 14.8; LR-ESIMS: [M + Na]^+^ calcd. for C_26_H_32_NaO_8_ 495.20, found: 495.20.

#### 3.5.2. General Procedure for the Preparation of **8**

TMSCHN_2_ (5.0 mL) was added to a solution of **7** (50.0 mg) in methanol (2.0 mL) at room temperature [15]. The solution was stirred at room temperature for 10 h and the solvent was then removed under reduced pressure. The resulting residue was dissolved in acetone and purified by RP_18_ HPLC to give **8** (44.8 mg).

**8:** White powder, Yield 89.7%; ^1^H NMR (400 MHz, CDCl_3_): *δ* 7.69 (br s, 1H), 7.40 (t, *J* = 1.6 Hz, 1H), 6.45 (br s, 1H), 5.62 (br d, *J* = 6.8 Hz, 1H), 5.57 (s, 1H), 3.90 (d, *J* = 9.6 Hz, 1H), 3.88 (dd, *J* = 10.0, 6.8 Hz, 1H), 3.68 (s, 3H), 3.39 (dd, *J* = 9.6, 2.0 Hz, 1H), 2.96 (dd, *J* = 18.8, 2.0 Hz, 1H), 2.90 (dd, *J* = 18.8, 5.6 Hz, 1H), 2.82 (br t, *J* = 8.4 Hz, 1H), 2.70 (br d, *J* = 10.4 Hz, 1H), 2.45 (s, 1H), 2.39 (dd, *J* = 16.8, 10.4 Hz, 1H), 2.32 (d, *J* = 16.8 Hz, 1H), 2.31 (m, 1H), 2.15 (br d, *J* = 12.8 Hz, 1H), 1.84 (d, *J* = 6.8 Hz, 1H), 1.77 (m, 1H), 1.65 (m, 1H), 1.61 (m, 1H), 1.45 (m, 1H), 1.10 (s, 3H), 1.05 (s, 3H), 0.66 (s, 3H); ^13^C NMR (100 MHz, CDCl_3_): *δ* 174.1, 170.4, 142.9, 141.8, 139.3, 120.7, 120.1, 109.7, 97.0, 77.1, 73.2, 68.5, 52.1, 48.0, 46.9, 45.2, 41.8, 37.6, 36.9, 34.5, 33.9, 32.0, 30.0, 21.9, 19.5, 14.8, 14.4; LR-ESIMS: [M + H]^+^ calcd. for C_27_H_35_O_8_ 487.23, found: 487.17.

#### 3.5.3. General Procedure for the Preparation of **8a**–**8i**

Compound **8** (5.0 mg, 0.01 mmol) and various organic acid (1.5 eq.) were dissolved in dry dichloromethane (2.0 mL). Then the mixture was combined with DCC (1.5 eq.) and DMAP (1.0 eq.), and then stirred at room temperature for 3–5 h [16]. TLC was used to monitor the products. After the reaction was completed, the reaction solvent was evaporated. The resulting residue was dissolved in acetone or acetonitrile; whereas the insoluble matter was precipitated by centrifugation. The supernatant was purified by RP_18_ HPLC to afford the pure corresponding esterified products (**8a**–**8i**).

**8a**: white powder, yield 74.5%; ^1^H NMR (400 MHz, CDCl_3_): *δ* 8.06 (dd, *J* = 8.0 Hz, 1.2 Hz, 2H), 7.78 (br s, 1H), 7.48 (tt, *J* = 7.6 Hz, 1.2 Hz, 1H), 7.43 (t, *J* = 1.6 Hz, 1H), 7.29 (t, *J* = 8.0 Hz, 2H), 6.44 (br d, *J* = 1.2 Hz, 1H), 5.46 (s, 1H), 5.37 (br d, *J* = 7.2 Hz, 1H), 5.13 (d, *J* = 10.0 Hz, 1H), 4.00 (d, *J* = 10.0 Hz, 1H), 3.72 (s, 3H), 3.59 (dd, *J* = 10.0 Hz, 1.6 Hz, 1H), 3.11 (br d, *J* = 10.4 Hz, 1H), 3.05 (br t, *J* = 8.4 Hz, 1H), 2.72 (br d, *J* = 4.0 Hz, 2H), 2.46 (dd, *J* = 16.8 Hz, 10.8 Hz, 1H), 2.38 (dd, *J* = 16.8 Hz, 1.6 Hz, 1H), 2.24 (br s, 1H), 2.14 (dd, *J* = 12.4, 4.8 Hz, 1H), 1.65 (m, 1H), 1.81 (qd, *J* = 13.2, 4.0 Hz, 1H), 1.65 (m, 1H), 1.59 (m, 1H), 1.43 (td, *J* = 14.0, 4.0 Hz, 1H), 1.11 (s, 3H), 1.02 (s, 3H), 0.73 (s, 3H); ^13^C NMR (100 MHz, CDCl_3_): *δ* 174.0, 168.9, 166.1, 143.0, 141.8, 139.0, 133.4, 129.7, 128.9, 128.6, 120.8, 119.8, 109.8, 97.0, 76.1, 75.4, 67.9, 52.2, 48.2, 45.5, 45.0, 41.6, 37.1, 36.6, 35.0, 34.5, 31.9, 29.6, 21.5, 19.7, 15.0, 14.5; HR-ESIMS: [M + H]^+^ calcd. for C_34_H_3__9_O_9_ 591.2589, found: 591.2592.

**8b**: colorless crystal, yield 70.3%; ^1^H NMR (400 MHz, CDCl_3_): *δ* 7.89 (br d, *J* = 8.8 Hz, 2H), 7.72 (br s, 1H), 7.45 (t, *J* = 1.6 Hz, 1H), 7.40 (br d, *J* = 8.8 Hz, 2H), 6.43 (br d, *J* = 1.2 Hz, 1H), 5.42 (s, 1H), 5.36 (br d, *J* = 7.2 Hz, 1H), 5.11 (d, *J* = 10.0 Hz, 1H), 3.99 (d, *J* = 9.6 Hz, 1H), 3.72 (s, 3H), 3.58 (dd, *J* = 9.6 Hz, 1.6 Hz, 1H), 3.08 (br d, *J* = 10.4 Hz, 1H), 3.03 (br t, *J* = 8.4 Hz, 1H), 2.76 (dd, *J* = 18.8, 5.2 Hz, 1H), 2.71 (dd, *J* = 18.8, 2.0 Hz, 1H), 2.45 (dd, *J* = 16.8, 10.4 Hz, 1H), 2.37 (br d, *J* = 16.8 Hz, 1H), 2.36 (br s, 1H), 2.24 (br s, 1H), 2.15 (dd, *J* = 12.4, 4.8 Hz, 1H), 1.80 (qd, *J* = 13.2, 4.4 Hz, 1H), 1.67 (m, 1H), 1.63 (m, 1H), 1.44 (td, *J* = 14.0, 4.4 Hz, 1H), 1.10 (s, 3H), 1.01 (s, 3H), 0.70 (s, 3H); ^13^C NMR (100 MHz, CDCl_3_): *δ* 174.0, 168.9, 165.4, 143.0, 141.5, 139.2, 132.0, 131.2, 128.6, 127.9, 120.9, 119.6, 109.7, 97.0, 76.4, 75.7, 67.8, 52.2, 48.1, 45.4, 45.0, 41.6, 37.0, 36.6, 34.9, 34.4, 31.8, 29.8, 21.5, 19.7, 15.0, 14.5; HR-ESIMS: [M + H]^+^ calcd. for C_34_H_3__8_BrO_9_ 669.1694, found: 669.1699.

**8c**: colorless crystal, yield 96.4%; ^1^H NMR (400 MHz, CDCl_3_): *δ* 7.76 (br s, 1H), 7.42 (t, *J* = 1.6 Hz, 1H), 6.85 (m, 4H), 6.45 (br d, *J* = 1.2 Hz, 1H), 5.58 (s, 1H), 5.35 (br d, *J* = 6.8 Hz, 1H), 4.99 (d, *J* = 10.0 Hz, 1H), 4.67 (s, 2H), 3.92(d, *J* = 10.0 Hz, 1H), 3.66 (s, 3H), 3.49 (dd, *J* = 10.0, 1.6 Hz, 1H), 2.95 (br t, *J* = 8.4 Hz, 1H), 2.92 (dd, *J* = 18.8, 5.2 Hz, 1H), 2.86 (dd, *J* = 18.8, 1.6 Hz, 1H), 2.82 (br d, *J* = 10.4 Hz, 1H), 2.36 (m, 1H), 2.34 (m, 1H), 2.31 (m, 2H), 2.18 (m, 1H), 1.81 (qd, *J* = 13.2, 4.4 Hz, 1H), 1.66 (m, 1H), 1.61 (m, 1H), 1.46 (td, *J* = 14.4, 4.8 Hz, 1H), 1.10 (s, 3H), 1.07 (s, 3H), 0.51 (s, 3H); ^13^C NMR (100 MHz, CDCl_3_): *δ* 173.7, 170.4, 169.1, 158.8, 156.4, 153.9, 142.9, 141.8, 138.8, 120.7, 119.5, 116.0, 115.9, 115.7, 115.5, 109.8, 96.7, 77.3, 76.2, 67.8, 65.4, 52.1, 47.5, 45.1, 44.8, 41.7, 36.8, 36.2, 35.2, 34.3, 31.8, 30.3, 22.0, 19.3, 14.7, 14.5; LR-ESIMS: [M + Na]^+^ calcd. for C_35_H_39_FNaO_10_ 661.24, found: 661.31.

**8d**: light yellow powder, yield 85.2%; ^1^H NMR (400 MHz, CDCl_3_): *δ* 8.22 (br d, *J* = 9.2 Hz, 2H), 8.08 (br d, *J* = 9.2 Hz, 2H), 7.72 (br s, 1H), 7.45 (t, *J* = 1.6 Hz, 1H), 6.36 (br d, *J* = 1.2 Hz, 1H), 5.36 (s, 1H), 5.31 (br d, *J* = 6.8 Hz, 1H), 5.10 (d, *J* = 10.0 Hz, 1H), 4.01 (d, *J* = 10.0 Hz, 1H), 3.76 (s, 3H), 3.60 (dd, *J* = 10.0, 1.6 Hz, 1H), 3.10 (br d, *J* = 10.8 Hz, 1H), 3.06 (m, 1H), 2.73 (dd, *J* = 18.8, 5.6 Hz, 1H), 2.66 (dd, *J* = 18.8, 2.0 Hz, 1H), 2.47 (dd, *J* = 17.2, 10.8 Hz, 1H), 2.39 (br d, *J* = 17.2 Hz, 1H), 2.23 (br s, 1H), 2.16 (dd, *J* = 13.2, 4.8 Hz, 1H), 1.79 (qd, *J* = 13.2, 4.4 Hz, 1H), 1.69 (m, 1H), 1.65 (m, 1H), 1.43 (td, *J* = 10.0, 5.2 Hz, 1H), 1.12 (s, 3H), 0.98 (s, 3H), 0.74 (s, 3H); ^13^C NMR (100 MHz, CDCl_3_): *δ* 174.2, 168.8, 164.2, 150.6, 143.3, 141.4, 139.8, 134.2, 130.9, 123.8, 120.7, 119.2, 109.6, 96.9, 76.7, 76.5, 67.7, 52.3, 48.1, 45.2, 45.0, 41.6, 36.9, 36.4, 34.9, 34.3, 31.7, 29.9, 21.5, 19.7, 15.0, 14.5; HR-ESIMS: [M + H]^+^ calcd. for C_34_H_38_NO_11_ 636.2439, found: 636.2449.

**8e**: light yellow crystal, yield 74.0%; ^1^H NMR (400 MHz, CDCl_3_): *δ* 8.85 (t, *J* = 1.6 Hz, 1H), 8.38 (dt, *J* = 7.6, 1.2 Hz, 1H), 8.32 (dq, *J* = 8.4, 1.2 Hz, 1H), 7.68 (br s, 1H), 7.48 (t, *J* = 8.0 Hz, 1H), 7.41(t, *J* = 1.6 Hz, 1H), 6.33 (br d, *J* = 1.2 Hz, 1H), 5.32 (s, 1H), 5.29 (br d, *J* = 6.8 Hz, 1H), 5.09 (d, *J* = 10.4 Hz, 1H), 4.02 (d, *J* = 9.6 Hz, 1H), 3.80 (s, 3H), 3.60 (dd, *J* = 9.6 Hz, 2.0 Hz, 1H), 3.09 (br d, *J* = 10.4 Hz, 1H), 3.08 (overlapped, 1H), 2.70 (dd, *J* = 18.8, 5.2 Hz, 1H), 2.65 (dd, *J* = 18.8, 2.8 Hz, 1H), 2.48 (dd, *J* = 16.8, 10.4 Hz, 1H), 2.42 (br s, 1H), 2.40 (br d, *J* = 16.8 Hz, 1H), 2.22 (br s, 1H), 2.15 (dd, *J* = 12.4, 5.2 Hz, 1H), 1.79 (qd, *J* = 13.2 Hz, 4.4 Hz, 1H), 1.66 (m, 1H), 1.56 (m, 1H), 1.42 (td, *J* = 14.0, 4.4 Hz, 1H), 1.12 (s, 3H), 0.96 (s, 3H), 0.79 (s, 3H); ^13^C NMR (100 MHz, CDCl_3_): *δ* 174.0, 168.9, 164.2, 148.3, 143.1, 141.7, 139.7, 135.5, 130.6, 129.9, 127.8, 124.7, 120.5, 119.2, 109.5, 96.9, 76.9, 76.4, 67.8, 52.4, 48.0, 45.2, 44.9, 41.6, 36.9, 36.3, 35.0, 34.4, 31.8, 29.7, 21.6, 19.6, 15.1, 14.5; HR-ESIMS: [M + H]^+^ calcd. for C_34_H_38_NO_11_ 636.2439, found: 636.2441.

**8f**: white powder, yield 63.7%; ^1^H NMR (400 MHz, CDCl_3_): *δ* 7.98 (dd, *J* = 7.6, 1.6 Hz, 1H), 7.73 (br s, 1H), 7.40 (t, *J* = 1.6 Hz, 1H), 7.40 (overlapped, 1H), 6.94 (d, *J* = 8.0 Hz, 1H), 6.76 (t, *J* = 7.6 Hz, 1H), 6.42 (br d, *J* = 1.2 Hz, 1H), 5.39 (s, 1H), 5.38 (br d, *J* = 6.8 Hz, 1H), 5.10 (d, *J* = 10.0 Hz, 1H), 4.0 (d, *J* = 9.6 Hz, 1H), 3.91 (s, 3H), 3.72 (s, 3H), 3.57 (dd, *J* = 9.6, 1.6 Hz, 1H), 3.06 (overlapped, 1H), 3.04 (br d, *J* = 10.8 Hz, 1H), 2.76 (dd, *J* = 18.8, 2.4 Hz, 1H), 2.71 (dd, *J* = 18.8, 5.6 Hz, 1H), 2.45 (dd, *J* = 16.8, 10.4 Hz, 1H), 2.37 (dd, *J* = 16.8, 1.6 Hz, 1H), 2.22 (br d, *J* = 2.4 Hz, 1H), 2.13 (dd, *J* = 12.4, 5.2 Hz, 1H), 1.79 (qd, *J* = 13.2, 4.4 Hz, 2H), 1.63 (m, 1H), 1.55 (m, 1H), 1.41 (td, *J* = 14.0, 4.4 Hz, 1H), 1.10 (s, 3H), 1.00 (s, 3H), 0.75 (s, 3H); ^13^C NMR (100 MHz, CDCl_3_): *δ* 174.0, 169.0, 164.9, 160.5, 142.8, 141.7, 138.6, 134.5, 132.2, 120.9, 120.3, 120.0, 117.4, 112.0, 109.9, 97.0, 76.1, 75.3, 68.1, 55.8, 52.1, 48.1, 45.5, 44.9, 41.7, 37.1, 36.5, 35.2, 34.5, 31.9, 29.6, 21.5, 19.6, 15.0, 14.6; HR-ESIMS: [M + H]^+^ calcd. for C_35_H_41_O_10_ 621.2694, found: 621.2709.

**8g**: pink powder, yield 55.6%; ^1^H NMR (400 MHz, CDCl_3_): *δ* 7.76 (br s, 1H), 7.42 (t, *J* = 1.6 Hz, 1H), 6.47 (br d, *J* = 1.2 Hz, 1H), 5.57 (s, 1H), 5.34 (br d, *J* = 6.8 Hz, 1H), 4.96 (d, *J* = 10.4 Hz, 1H), 3.93 (d, *J* = 9.6 Hz, 1H), 3.68 (s, 3H), 3.50 (dd, *J* = 9.6, 1.6 Hz, 1H), 2.89 (m, 1H), 2.88 (overlapped, 1H), 2.88 (overlapped, 1H), 2.87 (br d, *J* = 10.4 Hz, 1H), 2.39 (dd, *J* = 16.8, 10.4 Hz, 1H), 2.37 (m, 1H), 2.32 (dd, *J* = 16.8, 2.4 Hz, 1H), 2.30 (br s, 1H), 2.14 (dd, *J* = 12.4, 4.8 Hz, 1H), 1.96 (m, 1H), 1.92 (m, 1H), 1.77 (qd, *J* = 13.2, 4.4 Hz, 1H), 1.67 (m, 1H), 1.63 (m, 1H), 1.54 (m, 1H), 1.50 (m, 1H), 1.46 (m, 1H), 1.37 (m, 1H), 1.33 (m, 1H), 1.32 (m, 1H), 1.22 (m, 1H), 1.17 (m, 1H), 1.11 (s, 3H), 1.07 (s, 3H), 0.61 (s, 3H); ^13^C NMR (100 MHz, CDCl_3_): *δ* 175.5, 173.7, 169.9, 143.0, 141.8, 138.3, 120.7, 120.0, 109.7, 96.9, 76.7, 74.2, 67.9, 52.0, 48.0, 45.2, 45.2, 42.7, 41.6, 37.1, 36.6, 34.9, 34.5, 31.9, 30.0, 29.2, 28.4, 25.7, 25.4, 25.1, 21.7, 19.6, 14.8, 14.5; HR-ESIMS: [M + H]^+^ calcd. for C_34_H_45_O_9_ 597.3058, found: 597.3060.

**8h**: white powder, yield 54.2%; ^1^H NMR (400 MHz, CDCl_3_): *δ* 8.36 (d, *J* = 4.8 Hz, 1H), 7.88 (br s, 1H), 7.79 (dd, *J* = 5.2, 1.2 Hz, 1H), 7.73 (br s, 1H), 7.42 (t, *J* = 1.6 Hz, 1H), 6.37 (br d, *J* = 1.2 Hz, 1H), 5.36 (s, 1H), 5.26 (br d, *J* = 7.2 Hz, 1H), 5.05 (d, *J* = 10.0 Hz, 1H), 4.00 (d, *J* = 9.6 Hz, 1H), 3.78 (s, 3H), 3.58 (dd, *J* = 9.6, 1.6 Hz, 1H), 3.06 (br d, *J* = 10.0 Hz, 1H), 3.05 (overlapped, 1H), 2.73 (dd, *J* = 18.8, 5.6 Hz, 1H), 2.66 (br d, *J* = 18.8 Hz, 1H), 2.47 (dd, *J* = 16.8, 10.4 Hz, 1H), 2.40 (dd, *J* = 16.8, 1.6 Hz, 1H), 2.23 (br d, *J* = 2.8 Hz, 1H), 2.16 (dd, *J* = 13.0, 5.2 Hz, 1H), 1.77 (qd, *J* = 13.2, 4.0 Hz, 1H), 1.67 (m, 1H), 1.62 (m, 1H), 1.43 (td, *J* = 14.0, 4.0 Hz, 1H), 1.11 (s, 3H), 0.99 (s, 3H), 0.74 (s, 3H); ^13^C NMR (100 MHz, CDCl_3_): *δ* 174.1, 168.8, 163.5, 152.5, 150.7, 143.0, 141.7, 139.9, 138.9, 124.1, 121.7, 120.6, 119.0, 109.7, 96.9, 77.2, 76.5, 67.7, 52.3, 48.0, 45.0, 45.0, 41.6, 36.9, 36.3, 34.9, 34.3, 31.8, 29.9, 21.6, 19.6, 15.1, 14.4; HR-ESIMS: [M + H]^+^ calcd. for C_33_H_37_ClNO_9_ 626.2151, found: 626.2156.

**8i**: white crystal, yield 84.7%; ^1^H NMR (400 MHz, CDCl_3_): *δ* 7.76 (br s, 1H), 7.41 (t, *J* = 1.6 Hz, 1H), 6.46 (br d, *J* = 1.2 Hz, 1H), 5.56 (s, 1H), 5.32 (br d, *J* = 6.8 Hz, 1H), 4.87 (d, *J* = 10.0 Hz, 1H), 3.94 (d, *J* = 9.6 Hz, 1H), 3.69 (s, 3H), 3.50 (dd, *J* = 9.6, 1.6 Hz, 1H), 2.93 (br t, *J* = 8.4 Hz, 1H), 2.89 (dd, *J* = 18.8, 3.2 Hz, 1H), 2.85 (overlapped, 1H), 2.85 (br d, *J* = 10.4 Hz, 1H), 2.40 (dd, *J* = 16.8, 10.8 Hz, 1H), 2.36 (overlapped, 1H), 2.33 (dd, *J* = 16.8, 3.2 Hz, 1H), 2.31 (overlapped, 1H), 2.29 (br s, 1H), 2.15 (dd, *J* = 12.4, 5.2 Hz, 1H), 1.79 (qd, *J* = 13.2, 4.4 Hz, 1H), 1.63 (m, 1H), 1.56 (m, 1H), 1.45 (td, *J* = 13.2, 4.4 Hz, 1H), 1.25 (m, 2H), 1.24 (m, 2H), 1.22 (m, 2H), 1.20 (m, 2H), 1.19 (m, 2H), 1.10 (s, 3H), 1.07 (s, 3H), 0.83(t, *J* = 7.2 Hz, 3H), 0.63 (s, 3H); ^13^C NMR (100 MHz, CDCl_3_): *δ* 173.8, 173.8, 169.9, 142.9, 141.8, 138.4, 120.8, 120.0, 109.8, 96.8, 76.9, 75.0, 68.0, 52.0, 47.8, 45.1, 45.0, 41.6, 36.9, 36.2, 35.1, 34.5, 33.9, 31.9, 31.6, 30.1, 29.1, 28.9, 24.7, 22.6, 21.8, 19.4, 14.8, 14.5, 14.1; HR-ESIMS: [M + H]^+^ calcd. for C_35_H_49_O_9_ 613.3371, found: 613.3380.

#### 3.5.4. General Procedure for the Preparation of **9**

Compound **8** (50.0 mg) was dissolved in DMSO (2.0 mL) at room temperature. Then IBX (42.0 mg) was added [17]. The mixture was stirred at room temperature for 7.5 h. The solvent was then removed by lyophilization. The resulting residue was dissolved in acetone and purified by RP_18_ HPLC to give **9** (49.2 mg).

**9**: White powder, Yield 98.4%; ^1^H NMR (400 MHz, CDCl_3_): *δ* 7.64 (br s, 1H), 7.39 (t, *J* = 1.6 Hz, 1H), 6.42 (br d, *J* = 1.2 Hz, 1H), 5.77 (dt, *J* = 7.2, 2.0 Hz, 1H), 5.36 (s, 1H), 4.02 (d, *J* = 9.6 Hz, 1H), 3.69 (s, 3H), 3.55 (dd, *J* = 9.6, 1.6 Hz, 1H), 3.01 (br d, *J* = 7.2 Hz, 1H), 2.92 (s, 1H), 2.91 (d, *J* = 2.8 Hz, 1H), 2.74 (s, 1H), 2.58 (br d, *J* = 10.4 Hz, 1H), 2.51 (dd, *J* = 16.0, 10.4 Hz, 1H), 2.43 (br d, *J* = 16.0 Hz, 1H), 2.33 (overlapped, 1H), 2.30 (m, 1H), 1.72 (m, 1H), 1.67 (m, 1H), 1.63 (m, 1H), 1.45 (m, 1H), 1.20 (s, 3H), 1.11 (s, 3H), 0.79 (s, 3H); ^13^C NMR (100 MHz, CDCl_3_): *δ* 207.2, 173.2, 169.6, 143.0, 141.8, 138.4, 120.5, 119.1, 109.6, 97.8, 76.6, 66.1, 57.0, 52.4, 48.6, 46.4, 44.9, 42.2, 36.9, 36.8, 34.3, 31.7, 29.6, 21.8, 19.1, 14.5, 11.4; LR-ESIMS: [M + H]^+^ calcd. for C_27_H_33_O_8_ 485.21, found: 485.14.

#### 3.5.5. General Procedure for the Preparation of **9a** and **9b**

Compound **9** (5.0 mg, 0.01 mmol) and oxyamine (4 eq.) in ethanol (2 mL) were added with pyridine (4 eq.), and then stirred at room temperature for 48 h [18]. TLC was used to monitor the products. After the reaction was completed, the reaction solvent was precipitated by centrifugation. The resulting supernatant was purified by RP_18_ HPLC to give the pure corresponding derivatives, **9a** and **9b**.

**9a**: White powder, Yield 51.0%; ^1^H NMR (400 MHz, CDCl_3_): *δ* 7.65 (br s, 1H), 7.39 (t, *J* = 1.6 Hz, 1H), 6.42 (br d, *J* = 1.2 Hz, 1H), 6.07 (br d, *J* = 6.4 Hz, 1H), 5.40 (s, 1H), 3.97 (d, *J* = 9.2 Hz, 1H), 3.70 (s, 3H), 3.52 (overlapped, 1H), 3.52 (dd, *J* = 9.2, 1.2 Hz, 1H), 2.97 (br d, *J* = 18.8 Hz, 1H), 2.90 (dd, *J* = 18.8, 6.0 Hz, 1H), 2.54 (br d, *J* = 10.8 Hz, 1H), 2.48 (dd, *J* = 16.0, 10.8 Hz, 1H), 2.38 (br d, *J* = 16.0 Hz, 1H), 2.31 (br d, *J* = 2.8 Hz, 1H), 2.18 (dd, *J* = 12.0, 5.6 Hz, 1H), 1.71 (m, 1H), 1.64 (m, 1H), 1.59 (m, 1H), 1.42 (m, 1H), 1.13 (s, 3H), 1.09 (s, 3H), 0.86 (s, 3H); ^13^C NMR (100 MHz, CDCl_3_): *δ* 173.6, 170.4, 161.0, 143.0, 141.8, 136.6, 120.6, 119.9, 109.7, 97.2, 76.9, 68.4, 52.3, 47.9, 45.4, 44.8, 42.1, 39.5, 38.6, 36.8, 34.4, 32.0, 29.8, 21.8, 19.2, 14.7, 13.0; LR-ESIMS: [M + H]^+^ calcd. for C_27_H_34_NO_8_ 500.22, found: 500.16.

**9b**: White powder, Yield 60.0%; ^1^H NMR (400 MHz, CDCl_3_): δ 7.67 (br s, 1H), 7.40 (t, *J* = 1.6 Hz, 1H), 6.43 (br d, *J* = 1.2 Hz, 1H), 5.97 (dt, *J* = 6.8, 2.0 Hz, 1H), 5.42 (s, 1H), 3.97 (d, *J* = 9.2 Hz, 1H), 3.86 (s, 3H), 3.69 (s, 3H), 3.51 (dd, *J* = 9.2 Hz, 1.6 Hz, 1H), 3.43 (br d, *J* = 6.8 Hz, 1H), 2.97 (dd, *J* = 18.8, 1.6 Hz, 1H), 2.89 (dd, *J* = 18.8, 6.4 Hz, 1H), 2.53 (br d, *J* = 10.8 Hz, 1H), 2.48 (dd, *J* = 15.6, 10.8 Hz, 1H), 2.41 (s, 1H), 2.37 (br d, *J* = 15.6 Hz, 1H), 2.31 (m, 1H), 2.19 (dd, *J* = 12.4, 5.2 Hz, 1H), 1.71 (qd, *J* = 13.2, 4.0 Hz, 1H), 1.66 (m, 1H), 1.58 (m, 1H), 1.43 (td, *J* = 14.0, 4.8 Hz, 1H), 1.12 (s, 3H), 1.09 (s, 3H), 0.86 (s, 3H); ^13^C NMR (100 MHz, CDCl_3_): *δ* 173.5, 170.0, 159.5, 142.9, 141.8, 136.4, 120.6, 120.2, 109.6, 97.2, 76.8, 68.6, 61.8, 52.2, 47.9, 46.0, 44.9, 42.1, 39.6, 38.6, 36.8, 34.4, 32.1, 29.8, 21.8, 19.2, 14.6, 13.0; LR-ESIMS: [M + Na]^+^ calcd. for C_28_H_35_NNaO_8_ 536.23, found: 536.30.

#### 3.5.6. General Procedure for the Preparation of **10**

Compound **7** (50.0 mg) was dissolved in DMSO (2.0 mL) at room temperature. Then IBX (42.0 mg) was added [17]. Then the mixture was stirred for 7.5 h. TLC was used to monitor the products. Then the solvent was removed by lyophilization. The resulting residue was dissolved in acetone and purified by RP_18_ HPLC to afford **10** (49.4 mg).

**10**: White powder, Yield 98.8%; ^1^H NMR (400 MHz, CD_3_COCD_3_): *δ* 7.70 (br s, 1H), 7.54 (br s, 1H), 6.51 (br s, 1H), 5.69 (br d, *J* = 5.2 Hz, 1H), 5.35 (s, 1H), 4.14 (d, *J* = 9.6 Hz, 1H), 3.45 (d, *J* = 9.6 Hz, 1H), 3.00 (dd, *J* = 18.4, 6.4 Hz, 1H), 2.95 (d, *J* = 6.4 Hz, 1H), 2.82 (br d, *J* = 18.4 Hz, 1H), 2.65 (m, 1H), 2.60 (m, 1H), 2.59 (overlapped, 1H), 2.42 (br d, *J* = 6.4 Hz, 1H), 2.35 (dd, *J* = 12.0, 4.8 Hz, 1H), 1.78 (m, 1H), 1.71 (m, 1H), 1.55 (m, 1H), 1.52 (m, 1H), 1.26 (s, 3H), 1.14 (s, 3H), 0.79 (s, 3H); ^13^C NMR (100 MHz, CD_3_COCD_3_): *δ* 209.4, 175.6, 169.5, 144.1, 142.8, 139.7, 122.1, 120.1, 110.8, 98.4, 77.1, 66.3, 57.9, 49.5, 47.2, 45.6, 43.0, 37.7, 37.4, 35.1, 31.7, 30.4, 22.2, 19.8, 14.9, 12.0; LR-ESIMS: [M + H]^+^ calcd. for C_26_H_31_O_8_ 471.20, found: 471.14.

#### 3.5.7. General Procedure for the Preparation of **10a**–**10c**

Compound **10** (5.0 mg, 0.01 mmol) and oxyamine (4 eq.) in ethanol (2 mL) were added with pyridine (4 eq.), and then stirred at room temperature for 48 h [18]. TLC was used to monitor the products. After the reaction was completed, the solvent was precipitated by centrifugation. The supernatant was purified by RP_18_ HPLC to give the pure corresponding derivatives (**10a**–**10c**).

**10a**: white powder, yield 65.8%; ^1^H NMR (400 MHz, CD_3_COCD_3_): *δ* 7.73 (br s, 1H), 7.54 (t, *J* = 1.6 Hz, 1H), 6.52 (d, *J* = 1.2 Hz, 1H), 6.02 (dt, *J* = 6.8 Hz, 1H), 5.39 (s, 1H), 4.05 (d, *J* = 9.2 Hz, 1H), 3.52 (br d, *J* = 6.8 Hz, 1H), 3.37 (dd, *J* = 9.2, 1.6 Hz, 1H), 2.95 (dd, *J* = 18.4, 6.4 Hz, 1H), 2.83 (br d, *J* = 18.4 Hz, 1H), 2.58 (br t, *J* = 7.2 Hz, 1H), 2.56 (dd, *J* = 20.8, 8.2 Hz, 1H), 2.51 (dd, *J* = 20.8, 7.2 Hz, 1H), 2.37 (m, 1H), 2.22 (m, 1H), 1.74 (m, 1H), 1.71 (m, 1H), 1.51 (m, 1H), 1.48 (m, 1H), 1.19 (s, 3H), 1.13 (s, 3H), 0.89 (s, 3H); ^13^C NMR (100 MHz, CD_3_COCD_3_): *δ* 175.7, 169.5, 161.5, 144.0, 142.8, 137.6, 122.2, 120.9, 110.8, 97.8, 77.0, 69.0, 49.0, 46.4, 45.6, 42.8, 40.2, 39.4, 37.4, 35.3, 32.0, 30.5, 22.2, 19.9, 15.1, 13.7; LR-ESIMS: [M + H]^+^ calcd. for C_26_H_32_NO_8_ 486.21, found: 486.16.

**10b**: transparent crystal, yield 74.3%; ^1^H NMR (400 MHz, CD_3_COCD_3_): *δ* 7.73 (br s, 1H), 7.54 (t, *J* = 1.6 Hz, 1H), 6.53 (d, *J* = 1.6 Hz, 1H), 5.89 (dt, *J* = 6.4, 2.0 Hz, 1H), 5.38 (br s, 1H), 4.06 (d, *J* = 9.2 Hz, 1H), 3.78 (s, 3H), 3.43 (br d, *J* = 6.4 Hz, 1H), 3.37 (d, *J* = 8.8 Hz, 1H), 2.95 (dd, *J* = 18.4, 6.4 Hz, 1H), 2.78 (d, *J* = 18.4 Hz, 1H), 2.57 (br t, *J* = 7.2 Hz, 1H), 2.55 (d, *J* = 16.4 Hz, 1H), 2.53 (dd, *J* = 16.4, 7.2 Hz, 1H), 2.37 (br d, *J* = 6.4 Hz, 1H), 2.22 (m, 1H), 1.75 (m, 1H), 1.71 (m, 1H), 1.52 (m, 1H), 1.49 (m, 1H), 1.18 (s, 3H), 1.13 (s, 3H), 0.90 (s, 3H); ^13^C NMR (100 MHz, CD_3_COCD_3_): *δ* 175.7, 169.6, 162.4, 144.0, 142.8, 138.0, 122.2, 120.7, 110.8, 97.6, 77.1, 68.8, 61.7, 48.9, 46.7, 45.6, 42.8, 40.2, 39.4, 37.4, 35.2, 31.9, 30.6, 22.2, 19.8, 15.0, 13.6; LR-ESIMS: [M + Na]^+^ calcd. for C_27_H_34_NO_8_ 500.22, found: 500.17.

**10c**: transparent oil, yield 23.6%; ^1^H NMR (400 MHz, CD_3_COCD_3_): *δ* 7.75 (br s, 1H), 7.55 (t, *J* = 1.6 Hz, 1H), 7.41 (m, 1H), 7.39 (overlapped, 1H), 7.37 (m, 1H), 7.28 (m, 1H), 6.55 (d, *J* = 1.6 Hz, 1H), 5.97 (dt, *J* = 4.8, 2.0 Hz, 1H), 5.43 (s, 1H), 5.05 (dd, *J* = 12.0, 4.0 Hz, 2H), 4.07 (d, *J* = 9.2 Hz, 1H), 3.47 (d, *J* = 6.8 Hz, 1H), 3.37 (d, *J* = 8.8 Hz, 1H), 2.98 (dd, *J* = 18.4, 6.4 Hz, 1H), 2.80 (d, *J* = 18.4 Hz, 1H), 2.62 (d, *J* = 8.6 Hz, 1H), 2.58 (d, *J* = 5.8 Hz, 1H), 2.53 (d, *J* = 13.8 Hz, 1H), 2.39 (d, *J* = 6.4 Hz, 1H), 2.23 (t, *J* = 9.6 Hz, 1H), 1.76 (m, 1H), 1.73 (m, 1H), 1.53 (m, 1H), 1.50 (m, 1H), 1.18 (s, 3H), 1.15 (s, 3H), 0.90 (s, 3H); ^13^C NMR (100 MHz, CD_3_COCD_3_): *δ* 175.7, 169.6, 162.8, 144.1, 142.8, 139.0, 138.3, 129.2, 129.0, 128.4, 122.2, 120.5, 110.8, 97.7, 77.1, 76.4, 68.9, 49.0, 46.9, 45.6, 42.8, 40.2, 39.6, 37.5, 35.3, 32.0, 30.6, 22.2, 19.8, 15.0, 13.6; LR-ESIMS: [M + H]^+^ calcd. for C_33_H_38_NO_8_ 576.25, found: 576.18.

### 3.6. Antiviral Bioassay

Antiviral activities of **6**, **7**–**10**, **8a**–**8i**, **9a**–**9b**, and **10a**–**10c** against HIV-1 and IAV were performed according to references [14,19]. 293T cells (2 × 10^5^) were co-transfected with 0.6 μg of pNL-Luv-E^−^-Vpu^−^ and 0.4 μg of pHIT/G. After 48 h, the VSV-G pseudo-typed viral supernatant was harvested by filtration through a 0.45 μm filter, and the concentration of viral capsid protein was determined by p24 antigen capture ELISA (Biomerieux). SupT1 cells were exposed to VSV-G pseudo-typed virus (MOI = 1) at 37 °C for 48 h in the absence or presence of compounds at different concentrations. The inhibition rate was determined by using a firefly luciferase assay system (Promega, Beijing). Efavirenz was used as the positive control for HIV-1; whereas ribavirion was used as the positive control for IAV.

## 4. Conclusions

In conclusion, five new limonoids named thaigranatins A–E (**1**–**5**), containing a C_1_–*O*–C_29_ moiety, were isolated from seeds of the Thai *Xylocarpus granatum*, collected at the mangrove swamp of Trang Province, together with the known one, granatumin L (**6**). The structures of these limonoids, including absolute configurations of **1**, **2**, and **6** were established by HR-ESIMS, extensive NMR investigations, single-crystal X-ray diffraction analysis (Cu Kα radiation), and comparison of experimental ECD spectra. The structural modification of **6** via hydrolysis, esterification, and oximization, afforded 18 derivatives, *viz.* **7**–**10**, **8a**–**8i**, **9a**–**9b**, and **10a**–**10c**. The derivative **8i** exhibited marked inhibitory activity against HIV-1 with an IC_50_ value of 15.98 ± 6.87 μM and a CC_50_ value greater than 100.0 μM; whereas **10b** showed significant inhibitory activity against IAV with an IC_50_ value of 14.02 ± 3.54 μM and a CC_50_ value greater than 100.0 μM. These results demonstrate that the mangrove *X.* *granatum* continues to be an abundant resource to produce new antiviral limonoids. In-depth structural modification and structure-activity relationship studies of the limonoid skeleton of **6** will be proceeded by us for the discovery of antiviral drug leads.

## Figures and Tables

**Table 1:** ^1^H (400 MHz) NMR spectroscopic data of compounds **1**–**5** in CDCl_3_ (*δ* in ppm, *J* in Hz).

Position	1	2	3	4	5
2	3.00 t (8.4)	2.95 t (8.4)	2.77 dd (10.8, 2.4)	2.57 m	2.78 dd (10.4, 1.6)
3	4.71 d (10.0)	4.84 d (10.0)	5.14 d (10.8)	4.82 d (10.4)	5.27 d (10.8)
5	2.93 s	2.84 d (10.0)	2.98 d (10.8)	2.92 br s	2.64 ^a^
6a	4.56 s	2.35 m	2.42 dd (16.8, 2.0)	4.59 s	2.35 m
6b		2.35 m	2.42 dd (16.8, 10.8)		2.64 ^a^
9	2.24 m	2.15 dd (12.2, 5.2)	2.65 br s	2.46 m	
11*α*	1.76 m	1.62 ^a^	1.73 m	1.73 m	1.97 m
11*β*	1.76 m	1.77 m	1.79 m	1.73 m	2.23 m
12*α*	1.52 m	1.45 m	1.11 m	1.20 m	1.51 m
12*β*	1.71 m	1.62 ^a^	1.62 m	1.62 m	1.51 m
14	2.31 br s	2.30 br s			2.52 mdd (11.6, 5.2)
15*α*	2.80 d (21.6)	2.84 d (21.6)	3.41 dd (21.2, 3.2)	3.25 dt (21.6, 2.8)	2.69 dd (16.0, 3.2)
15*β*	2.84 d (21.6)	2.91 d (21.6)	3.59 dd (21.2, 1.6)	3.44 d (21.6)	2.18 dd (16.0, 3.2)
17	5.42 s	5.55 s	5.41 s	5.33 s	4.90 s
18	0.98 s	1.09 s	1.08 s	0.98 s	0.78 s
19	1.40 s	1.07 s	1.13 s	1.38 s	1.11 s
21	7.50 br s	7.74 br s	7.53 br s	7.44 br s	7.44 br s
22	6.38 br s	6.45 br s	6.46 br s	6.38 br s	6.41 br s
23	7.44 br s	7.41 br s	7.42 br s	7.42 br s	7.42 br s
28	0.77 s	0.63 s	0.56 s	0.71 s	0.70 s
H_*pro-S*_-29	4.60 d (8.8)	3.93 d (9.6)	3.92 d (9.6)	4.59 d (8.8)	3.80 d (10.0)
H_*pro-R*_-29	3.45 d (8.8)	3.48 dd (9.6, 1.6)	3.51 dd (9.6, 1.6)	3.42 (8.8, 1.6)	3.69 (10.0)
30*β*	5.28 d (6.8)	5.31 d (6.8)	4.51 d (2.4)	2.24 m	3.94 br s
30*α*				2.24 m	
31	3.76 s	3.69 s	3.70 s	3.84 s	3.72 s
	3-Acyl	3-Acyl	3-Acyl	3-Acyl	3-Acyl
33		2.37 m			
		2.37 m			
34	6.83 q (7.2)	1.09 t (7.2)	6.99 q (7.2)	6.92 q (7.2)	6.89 q (7.2)
35	1.67 d (7.2)		1.80 dd (7.2, 1.2)	1.80 dd (7.2, 0.8)	1.86 d (7.2)
36	1.79 s		1.87 s	1.86 s	1.88 s

^a^ Overlapped signals assigned by ^1^H–^1^H COSY, HSQC, and HMBC spectra without designating multiplicity.

**Table 2:** ^13^C (100 MHz) NMR spectroscopic data of compounds **1**–**5** in CDCl_3_ (*δ* in ppm).

Position	1	2	3	4	5
1	96.9 qC	96.9 qC	97.4 qC	97.1 qC	97.4 qC
2	45.3 CH	45.1 CH	48.2 CH	43.6 CH	46.2 CH
3	76.2 CH	75.2 CH	75.2 CH	78.5 CH	74.8 CH
4	36.6 qC	36.2 qC	37.1 qC	36.6 qC	36.8 qC
5	39.6 CH	35.0 CH	34.5 CH	39.3 CH	40.6 CH
6	72.5 CH	31.9 CH_2_	32.1 CH_2_	73.1 CH	31.9 CH_2_
7	176.1 qC	173.9 qC	174.2 qC	175.8 qC	174.3 qC
8	138.3 qC	138.4 qC	131.3 qC	130.6 qC	127.1 qC
9	48.9 CH	47.8 CH	38.7 CH	43.9 CH	139.2 qC
10	42.1 qC	41.6 qC	45.1 qC	44.7 qC	43.6 qC
11	20.4 CH_2_	19.4 CH_2_	17.7 CH_2_	18.2 CH_2_	21.1 CH_2_
12	34.6 CH_2_	34.5 CH_2_	29.4 CH_2_	30.3 CH_2_	29.4 CH_2_
13	36.7 qC	36.9 qC	38.2 qC	37.8 qC	35.3 qC
14	44.8 CH	45.0 CH	134.3 qC	129.0 qC	36.8 CH
15	29.6 CH_2_	30.1 CH_2_	32.3 CH_2_	33.1 CH_2_	32.2 CH_2_
16	169.3 qC	170.1 qC	169.5 qC	169.8 qC	172.7 qC
17	76.6 CH	77.0 CH	80.7 CH	81.6 CH	81.3 CH
18	21.2 CH_3_	21.9 CH_3_	17.4 CH_3_	18.0 CH_3_	20.2 CH_3_
19	14.9 CH_3_	14.4 CH_3_	14.2 CH_3_	15.1 CH_3_	14.3 CH_3_
20	121.5 qC	120.8 qC	120.7 qC	121.1 qC	121.2 qC
21	140.3 CH	141.8 CH	141.6 CH	140.9 CH	140.4 CH
22	109.3 CH	109.7 CH	109.9 CH	109.8 CH	109.7 CH
23	143.1 CH	143.0 CH	142.9 CH	143.1 CH	143.2 CH
28	15.8 CH_3_	14.8 CH_3_	15.6 CH_3_	16.4 CH_3_	18.2 CH_3_
29	70.1 CH_2_	68.0CH_2_	67.6 CH_2_	70.0 CH_2_	68.6 CH_2_
30	120.2 CH	119.9 CH	66.3 CH	26.4 CH_2_	66.1 CH
31	53.2 CH_3_	52.1 CH_3_	52.1 CH_3_	53.1 CH_3_	52.0 CH_3_
	3-Acyl	3-Acyl	3-Acyl	3-Acyl	3-Acyl
32	167.2 qC	174.5 qC	167.6 qC	167.5 qC	167.6 qC
33	127.8 qC	27.2 CH_2_	128.8 qC	128.9 qC	128.4 qC
34	138.7 CH	8.8 CH_3_	139.6 CH	138.7 CH	139.2 CH
35	14.6 CH_3_		14.6 CH_3_	14.6 CH_3_	14.8 CH_3_
36	11.7 CH_3_		12.3 CH_3_	12.2 CH_3_	12.4 CH_3_

**Table 3:** Inhibitory activities of natural limonoid **6** and its modified derivatives against HIV-1.

cpd No.	Inhibition Rate (20.0 μM) (%)	IC_50_ Value (μM)	CC_50_ Value (μM)	cpd No.	Inhibition Rate (20.0 μM) (%)	IC_50_ Value (μM)	CC_50_ Value (μM)
6	67.10 ± 3.04	19.23 ± 0.12	> 100	8f	NA	NT	NT
7	NA	NT	NT	8g	50.84 ± 6.96	21.98 ± 4.65	86.76 ± 4.76
8	NA	NT	NT	8h	NA	NT	NT
9	NA	NT	NT	8i	99.95 ± 0.01	15.98 ± 6.87	> 100
10	NA	NT	NT	9a	NA	NT	NT
8a	NA	NT	NT	9b	NA	NT	NT
8b	39.87 ± 2.83	NT	NT	10a	NA	NT	NT
8c	45.47 ± 6.84	NT	NT	10b	NA	NT	NT
8d	35.63 ± 8.85	NT	NT	10c	NA	NT	NT
8e	10.58 ± 3.55	NT	NT	EFV	88.78 ± 4.54 (4 nM)	NT	NT

cpd: compound; NA: No activity; NT = Not tested.

**Table 4:** Inhibitory activities of natural limonoid **6** and its modified derivatives against IAV.

cpd No.	Inhibition Rate (20.0 μM) (%)	IC_50_ Value (μM)	CC_50_ Value (μM)	cpd No.	Inhibition Rate (20.0 μM) (%)	IC_50_ Value (μM)	CC_50_ Value (μM)
7	38.25 ± 9.67	NT	NT	8e	43.42 ± 1.79	NT	NT
8	NA	NT	NT	8f	44.94 ± 9.73	NT	NT
9	NA	NT	NT	8h	48.15 ± 1.64	NT	NT
10	NA	NT	NT	9a	44.72 ± 1.43	NT	NT
8a	23.46 ± 2.42	NT	NT	10a	NA	NT	NT
8b	54.65 ± 1.43	17.93 ± 4.76	85.90 ± 4.65	10b	63.40 ± 7.43	14.02 ± 3.54	> 100
8c	68.61 ± 0.99	15.87 ± 6.54	77.98 ± 4.65	10c	NA	NT	NT
8d	51.41 ± 1.54	22.78 ± 1.86	87.65 ± 4.76	ribavirin	87.54 ± 2.86 (60 μM)	NT	NT

cpd: compound; NA: No activity; NT = Not tested.

## Supplementary Materials

The following are available online at http://www.mdpi.com/1660-3397/16/11/434/s1. Copies of HR-ESIMS, 1D and 2D NMR spectra of compounds **1**–**5**; and copies of LR/HR-ESIMS and 1D NMR spectra of compounds **7**, **8**, **9**, **10**, **8a**–**8i**, **9a**, **9b**, and **10a**–**10c**.
