# Supplementary material for: Limonoids Containing a C1–O–C29 Moiety: Isolation, Structural Modification, and Antiviral Activity
Source: Mar Drugs. 2018 Nov 4;16(11):434. doi: 10.3390/md16110434 (PMC6266505; doi:10.3390/md16110434)

Supporting Information-I

Copies of HR-ESIMS, and 1D and 2D NMR spectra of compounds 1–5.

Table of Contents:

1. HR-ESIMS for compound **1** ········ S4-S5
2. ^{1}H NMR (400 MHz) spectrum of compound **1** in CDCl3 ········ S6-S8
3. ^{13}C NMR (100 MHz) spectrum of compound **1** in CDCl3 ········ S9-S12
4. DEPT135 (100 MHz) spectrum of compound **1** in CDCl3 ········ S13-S16
5. ^{1}H-^{1}H COSY (400 MHz) spectrum of compound **1** in CDCl3 ········ S17-S18
6. HSQC (400 MHz) spectrum of compound **1** in CDCl3 ········ S19-S20
7. HMBC (400 MHz) spectrum of compound **1** in CDCl3 ········ S21-S25
8. NOESY (400 MHz) spectrum of compound **1** in CDCl3 ········ S26-S29
9. HR-ESIMS for compound **2** ········ S30-S31
10. ^{1}H NMR (400 MHz) spectrum of compound **2** in CDCl3 ········ S32-S34
11. ^{13}C NMR (100 MHz) spectrum of compound **2** in CDCl3 ········ S35-S38
12. DEPT135 (100 MHz) spectrum of compound **2** in CDCl3 ········ S39-S42
13. ^{1}H-^{1}H COSY (400 MHz) spectrum of compound **2** in CDCl3 ········ S43-S46
14. HSQC (400 MHz) spectrum of compound **2** in CDCl3 ········ S47-S49
15. HMBC (400 MHz) spectrum of compound **2** in CDCl3 ········ S50-S55
16. NOESY (400 MHz) spectrum of compound **2** in CDCl3 ········ S56-S58
17. HR-ESIMS for compound **3** ········ S59-S60
18. ^{1}H NMR (400 MHz) spectrum of compound **3** in CDCl3 ········ S61-S63
19. ^{13}C NMR (100 MHz) spectrum of compound **3** in CDCl3 ········ S64-S67
20. DEPT135 (100 MHz) spectrum of compound **3** in CDCl3 ········ S68-S71

21. ^{1}H-^{1}H COSY (400 MHz) spectrum of compound **3** in CDCl3 ·············S72-S74
22. HSQC (400 MHz) spectrum of compound **3** in CDCl3 ·····················S75-S77
23. HMBC (400 MHz) spectrum of compound **3** in CDCl3 ···················S78-S82
24. NOESY (400 MHz) spectrum of compound **3** in CDCl3 ················S83-S86
25. HR-ESIMS for compound **4**···S87-S88
26. ^{1}H NMR (400 MHz) spectrum of compound **4** in CDCl3················S89-S91
27. ^{13}C NMR (100 MHz) spectrum of compound **4** in CDCl3···············S92-S95
28. DEPT135 (100 MHz) spectrum of compound **4** in CDCl3 ··············S96-S99
29. ^{1}H-^{1}H COSY (400 MHz) spectrum of compound **4** in CDCl3 ··········S100-S101
30. HSQC (400 MHz) spectrum of compound **4** in CDCl3 ·················S102-S104
31. HMBC (400 MHz) spectrum of compound **4** in CDCl3 ················S105-S109
32. NOESY (400 MHz) spectrum of compound **4** in CDCl3 ················S110-S113
33. HR-ESIMS for compound **5**··S114-S115
34. ^{1}H NMR (400 MHz) spectrum of compound **5** in CDCl3················S116-S118
35. ^{13}C NMR (100 MHz) spectrum of compound **5** in CDCl3···············S119-S121
36. DEPT135 (100 MHz) spectrum of compound **5** in CDCl3 ··············S122-S124
37. ^{1}H-^{1}H COSY (400 MHz) spectrum of compound **5** in CDCl3 ··········S125-S128
38. HSQC (400 MHz) spectrum of compound **5** in CDCl3 ·················S129-S131
39. HMBC (400 MHz) spectrum of compound **5** in CDCl3 ················S132-S136
40. NOESY (400 MHz) spectrum of compound **5** in CDCl3 ···············S137-S140

HR-ESIMS for compound **1**

Generic Display Report

Analysis Info

Analysis Name	D:\Data\MS\data\201807\liaoqi_P-4_pos_57_01_5065.d	Acquisition Date	7/3/2018 3:44:16 PM
Method	LC_Direct Infusion_pos_100-1000mz.m	Operator	SCSIO
Sample Name	liaoqi_P-4_pos	Instrument	maXis
Comment			

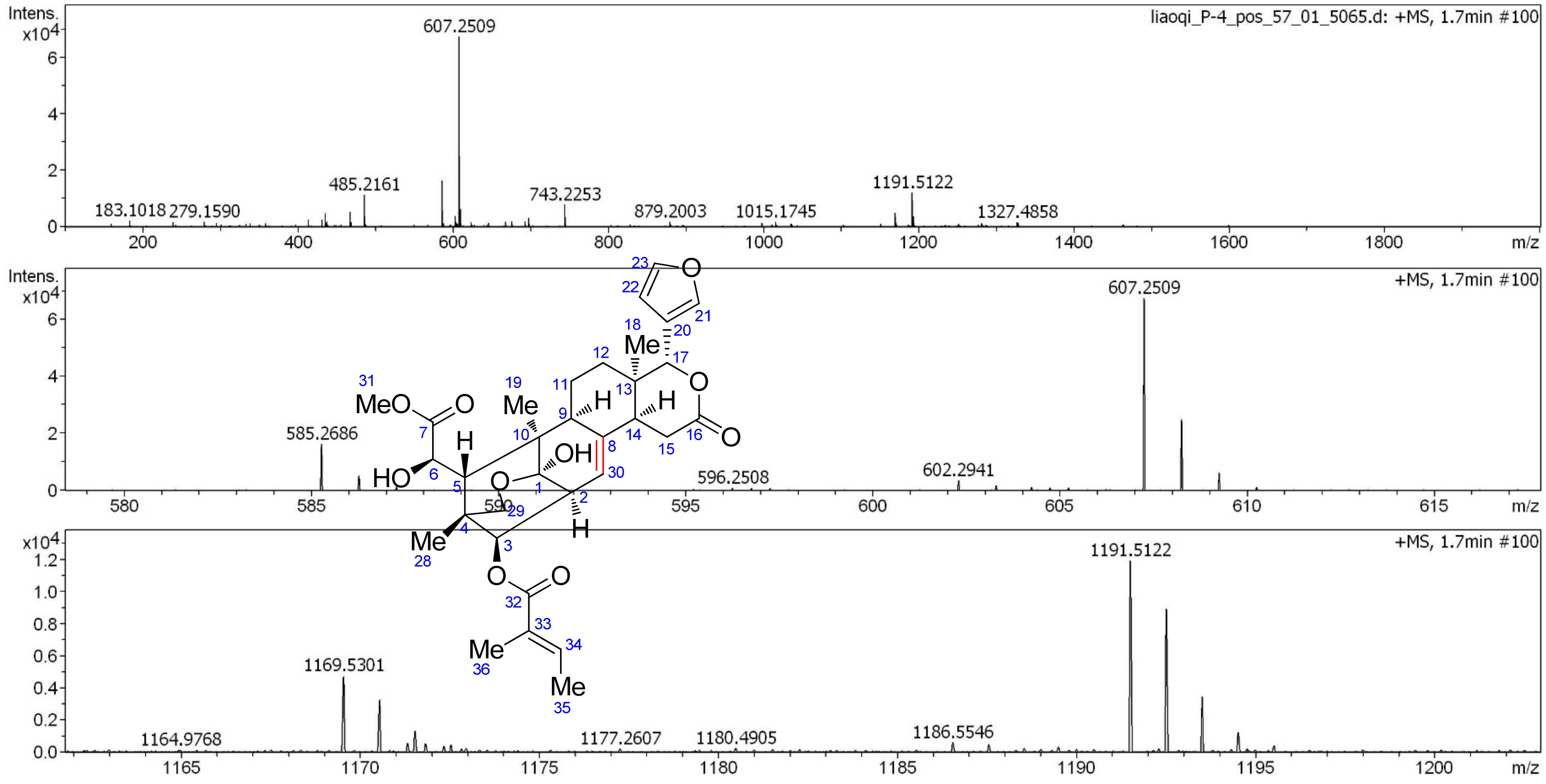

HR-ESIMS for compound **1**

Mass Spectrum SmartFormula Report

Analysis Info

Analysis Name	D:\Data\MS\data\201807\liaoqi_P-4_pos_57_01_5065.d	Acquisition Date	7/3/2018 3:44:16 PM
Method	LC_Direct Infusion_pos_100-1000mz.m	Operator	SCSIO
Sample Name	liaoqi_P-4_pos	Instrument	maXis 255552.00029
Comment			

Acquisition Parameter

Source Type	ESI	Ion Polarity	Positive	Set Nebulizer	0.4 Bar
Focus	Active	Set Capillary	4500 V	Set Dry Heater	180 °C
Scan Begin	100 m/z	Set End Plate Offset	-500 V	Set Dry Gas	4.0 l/min
Scan End	2000 m/z	Set Charging Voltage	0 V	Set Divert Valve	Waste
		Set Corona	0 nA	Set APCI Heater	0 °C

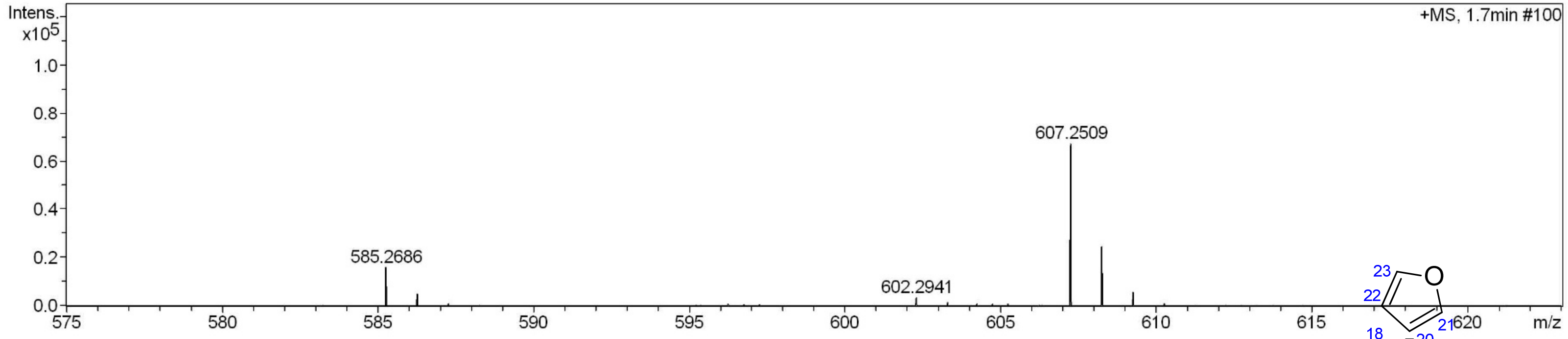

Meas. m/z	#	Ion Formula	Score	m/z	err [ppm]	err [mDa]	mSigma	rdb	e⁻ Conf	N-Rule
585.2686	1	C32H41O10	100.00	585.2694	-1.4	-0.8	18.7	12.5	even	ok
607.2509	1	C32H40NaO10	100.00	607.2514	-0.7	-0.4	8.6	12.5	even	ok
1169.5301	1	C64H81O20	100.00	1169.5316	-1.2	-1.4	13.4	24.5	even	ok
1191.5122	1	C64H80NaO20	100.00	1191.5135	-1.1	-1.3	18.4	24.5	even	ok

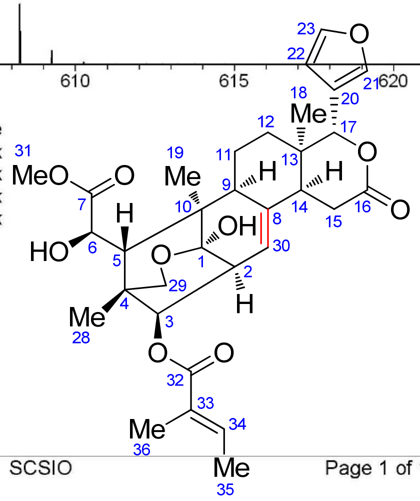

^{1}H NMR (400 MHz) spectrum of compound **1** in $CDCl_3$

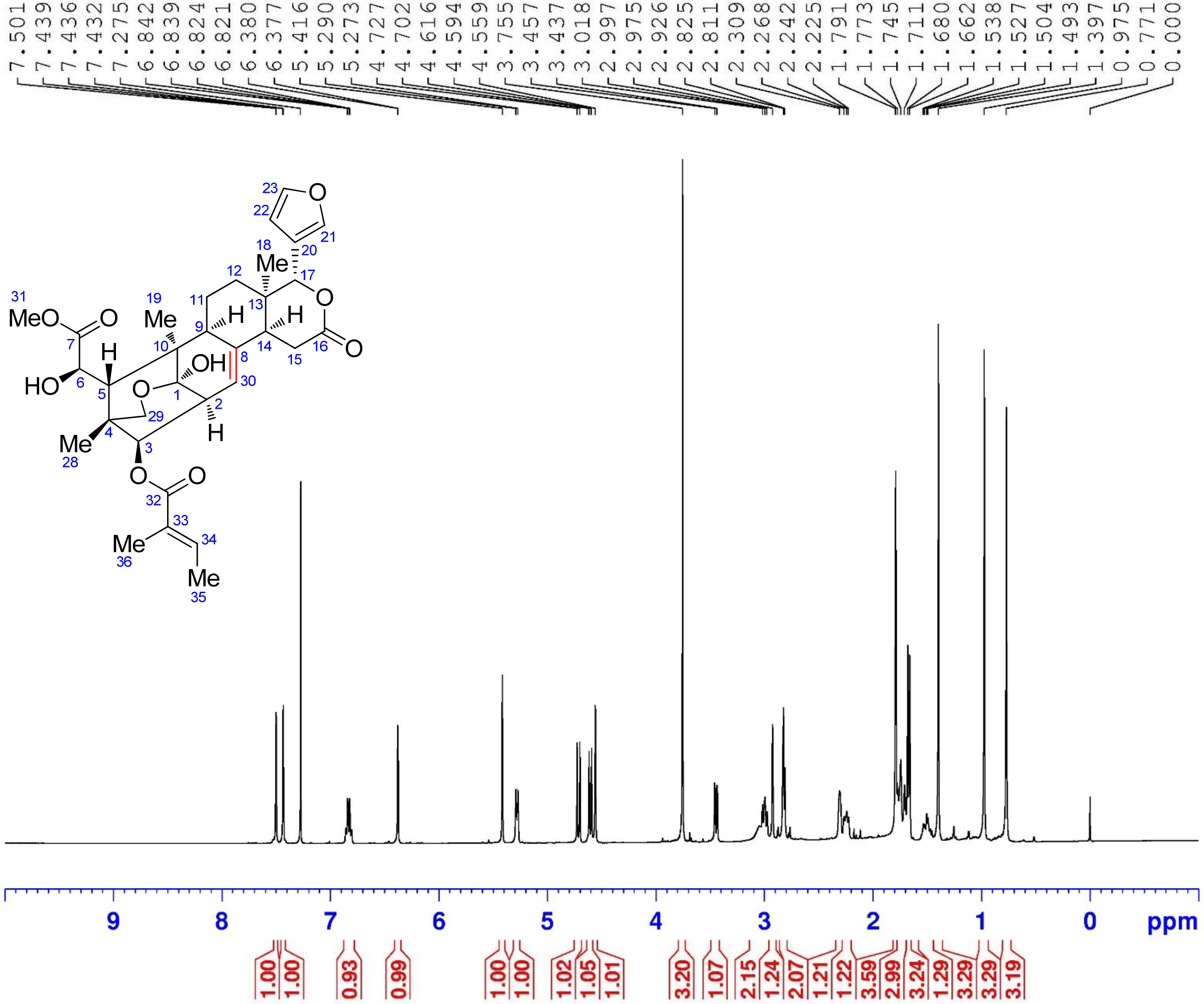


```
NAME          ZXP-R-15-3-3
EXPNO                    1
PROCNO                   1
Date_             20180508
Time                  6.06
INSTRUM              spect
PROBHD      5 mm CPPBBO BB
PULPROG               zg30
TD                   65536
SOLVENT              CDCl3
NS                      16
DS                       2
SWH               8223.685 Hz
FIDRES            0.125483 Hz
AQ               3.9846387 sec
RG                   44.55
DW                  60.800 usec
DE                   10.00 usec
TE                   297.0 K
D1              1.00000000 sec
TD0                      1

======== CHANNEL f1 ========
SFO1           400.1324710 MHz
NUC1                    1H
P1                   11.50 usec
SI                   65536
SF             400.1300037 MHz
WDW                     EM
SSB                      0
LB                    0.30 Hz
GB                       0
PC                    1.00
```

^{1}H NMR (400 MHz) spectrum of compound **1** in $CDCl_3$

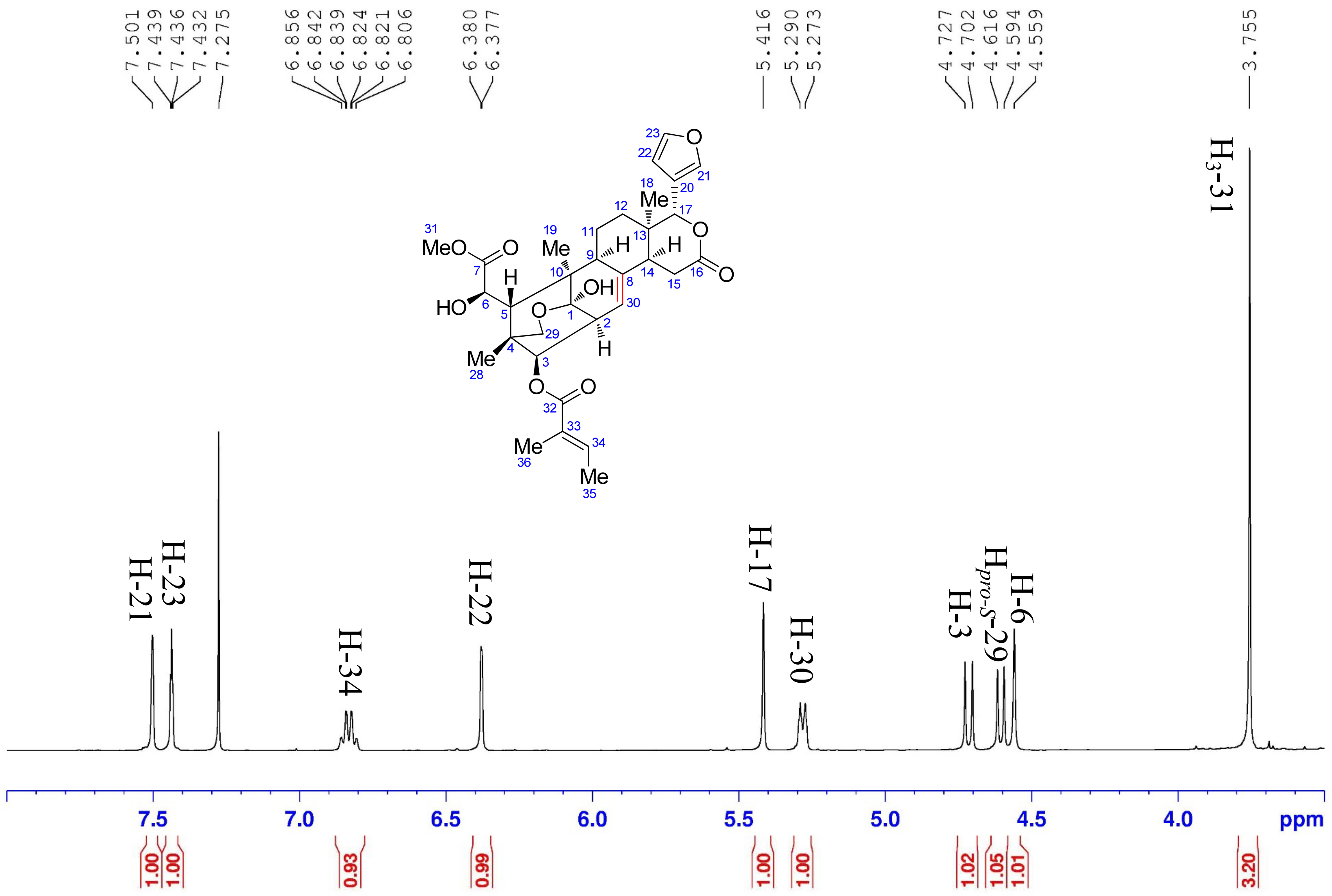

^{1}H NMR (400 MHz) spectrum of compound **1** in $CDCl_3$

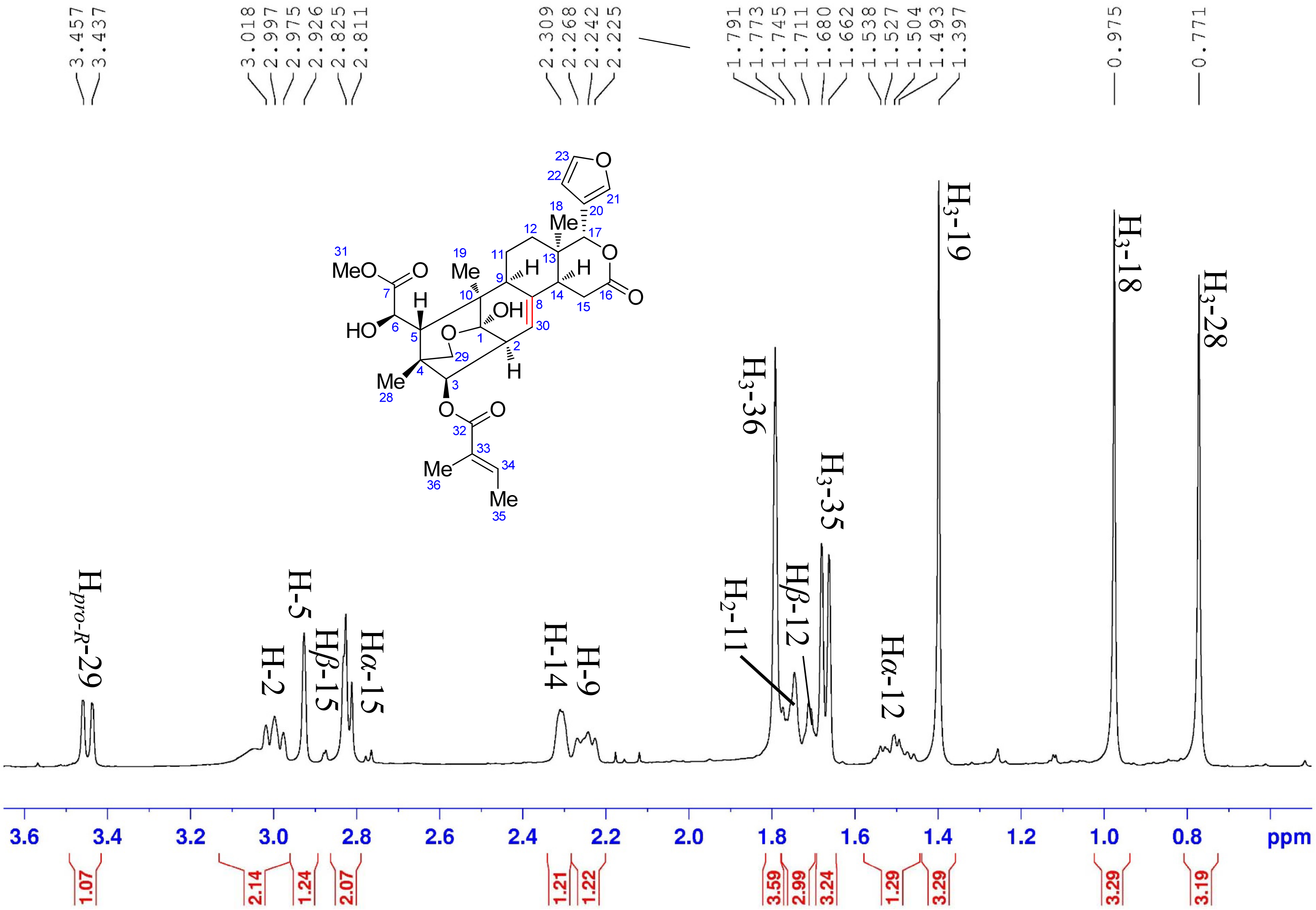

^{13}C NMR (100 MHz) spectrum of compound **1** in $CDCl_3$

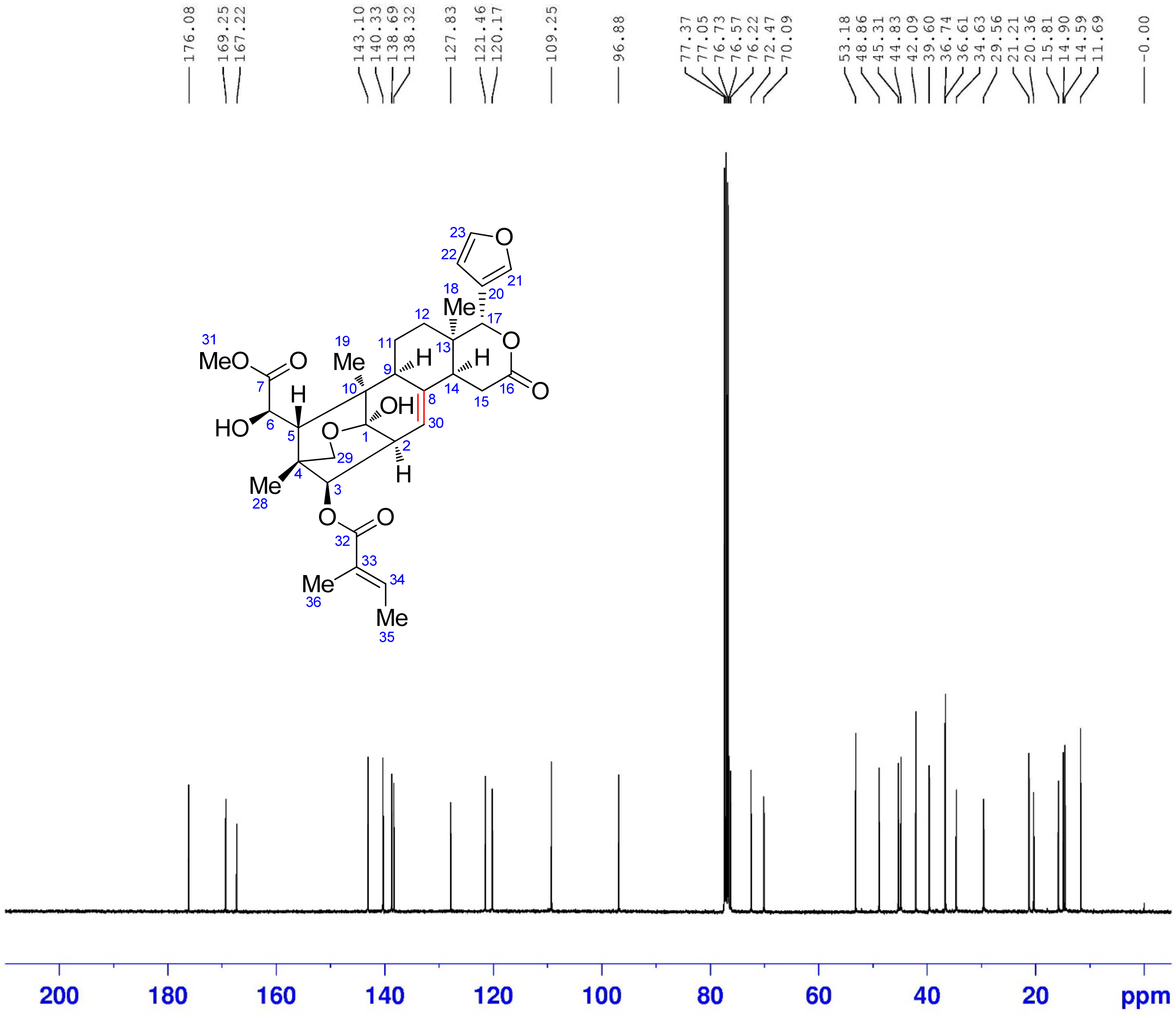


```
NAME           zxp-R-15-3-3
EXPNO                     2
PROCNO                    1
Date_              20180508
Time                   7.05
INSTRUM               spect
PROBHD    5 mm CPPBBO BB
PULPROG              zgpg30
TD                    65536
SOLVENT               CDCl3
NS                     1024
DS                        4
SWH               24038.461 Hz
FIDRES             0.366798 Hz
AQ                1.3631988 sec
RG                   130.26
DW                   20.800 usec
DE                    18.00 usec
TE                    297.0 K
D1               2.00000000 sec
D11              0.03000000 sec
TD0                       1

======== CHANNEL f1 ========
SFO1            100.6233324 MHz
NUC1                    13C
P1                    10.00 usec
SI                    32768
SF              100.6127690 MHz
WDW                      EM
SSB                       0
LB                     1.00 Hz
GB                        0
PC                     1.40
```

^{13}C NMR (100 MHz) spectrum of compound **1** in $CDCl_3$

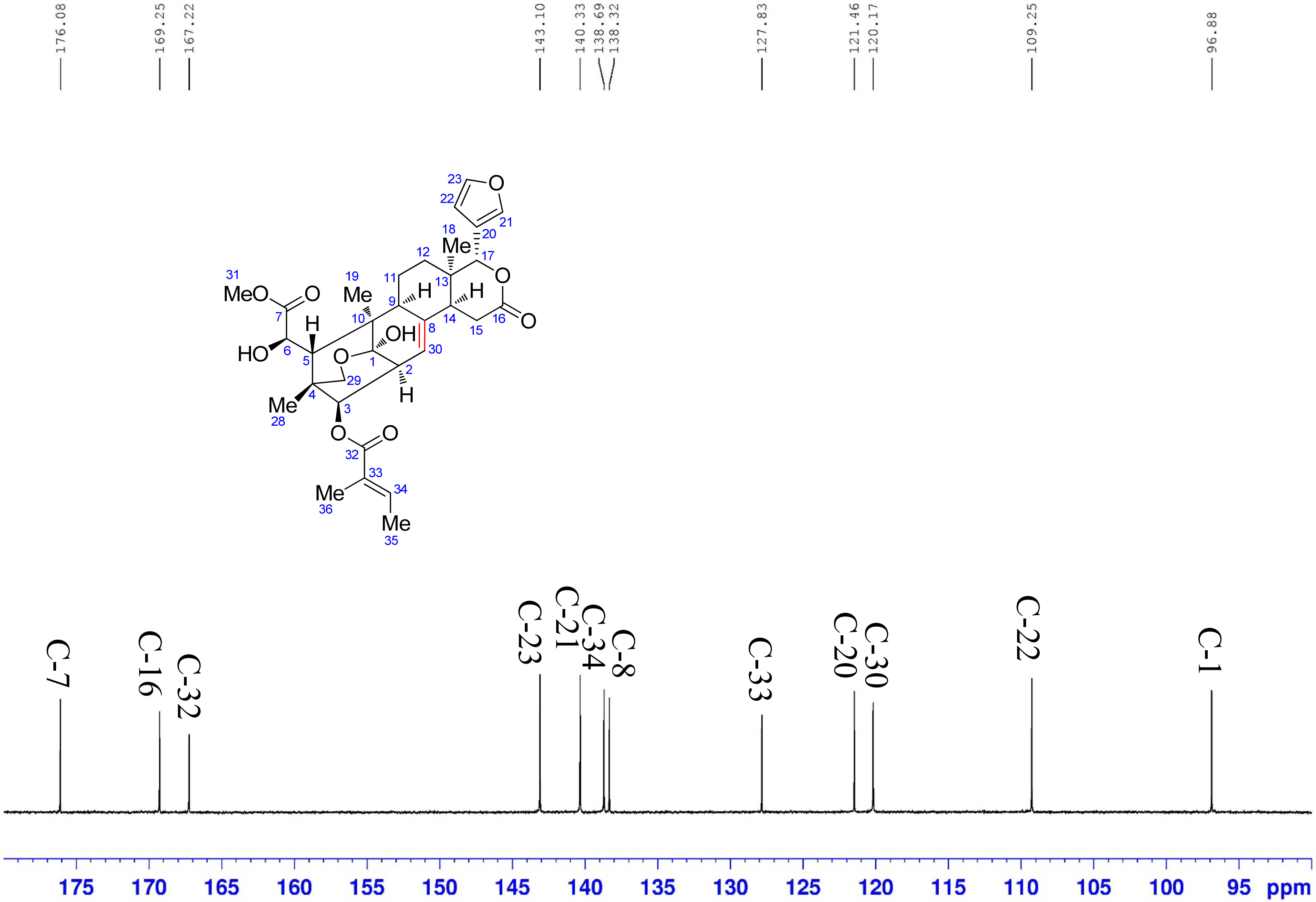

^{13}C NMR (100 MHz) spectrum of compound **1** in $CDCl_3$

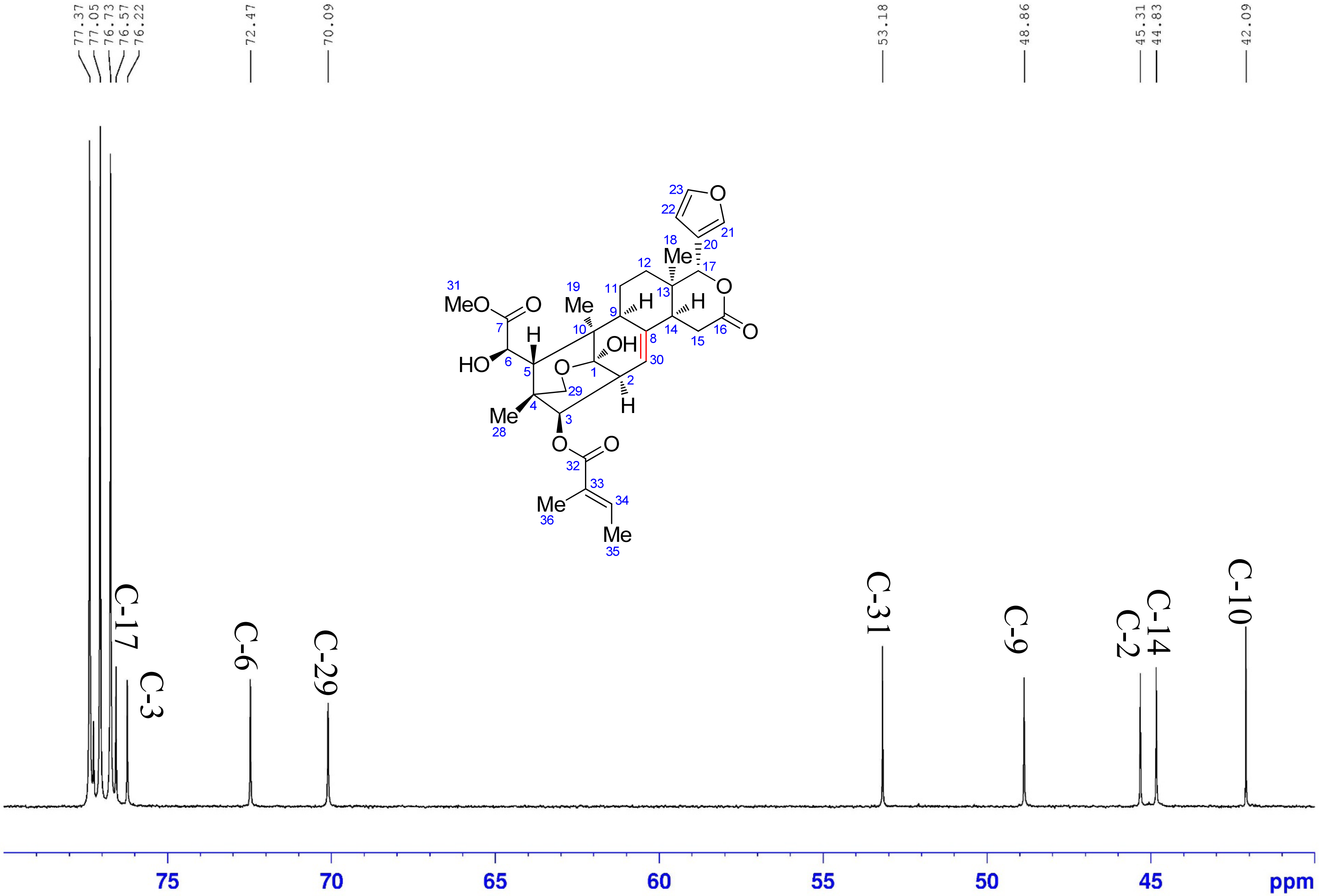

^{13}C NMR (100 MHz) spectrum of compound **1** in $CDCl_3$

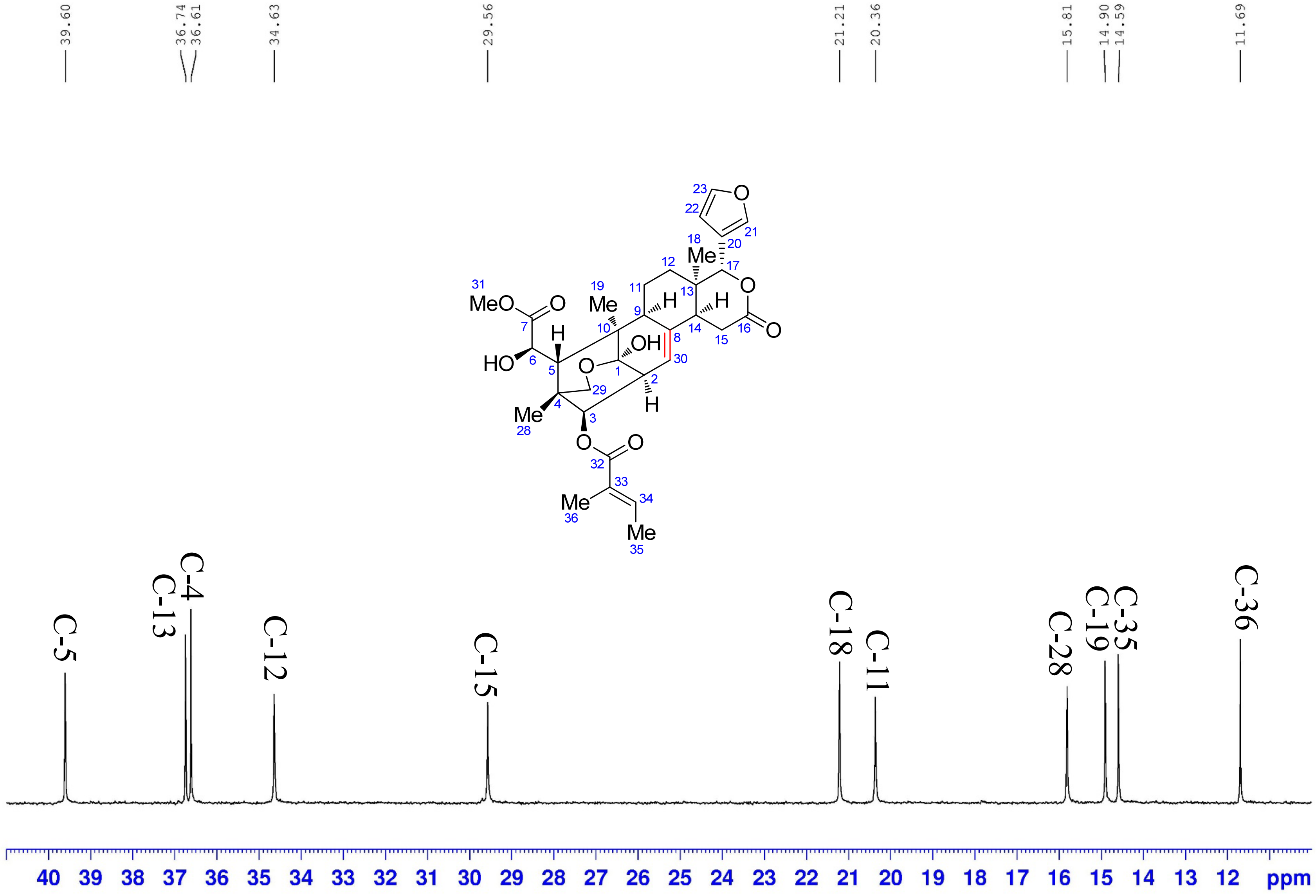

DEPT135 (100 MHz) spectrum of compound **1** in $CDCl_3$

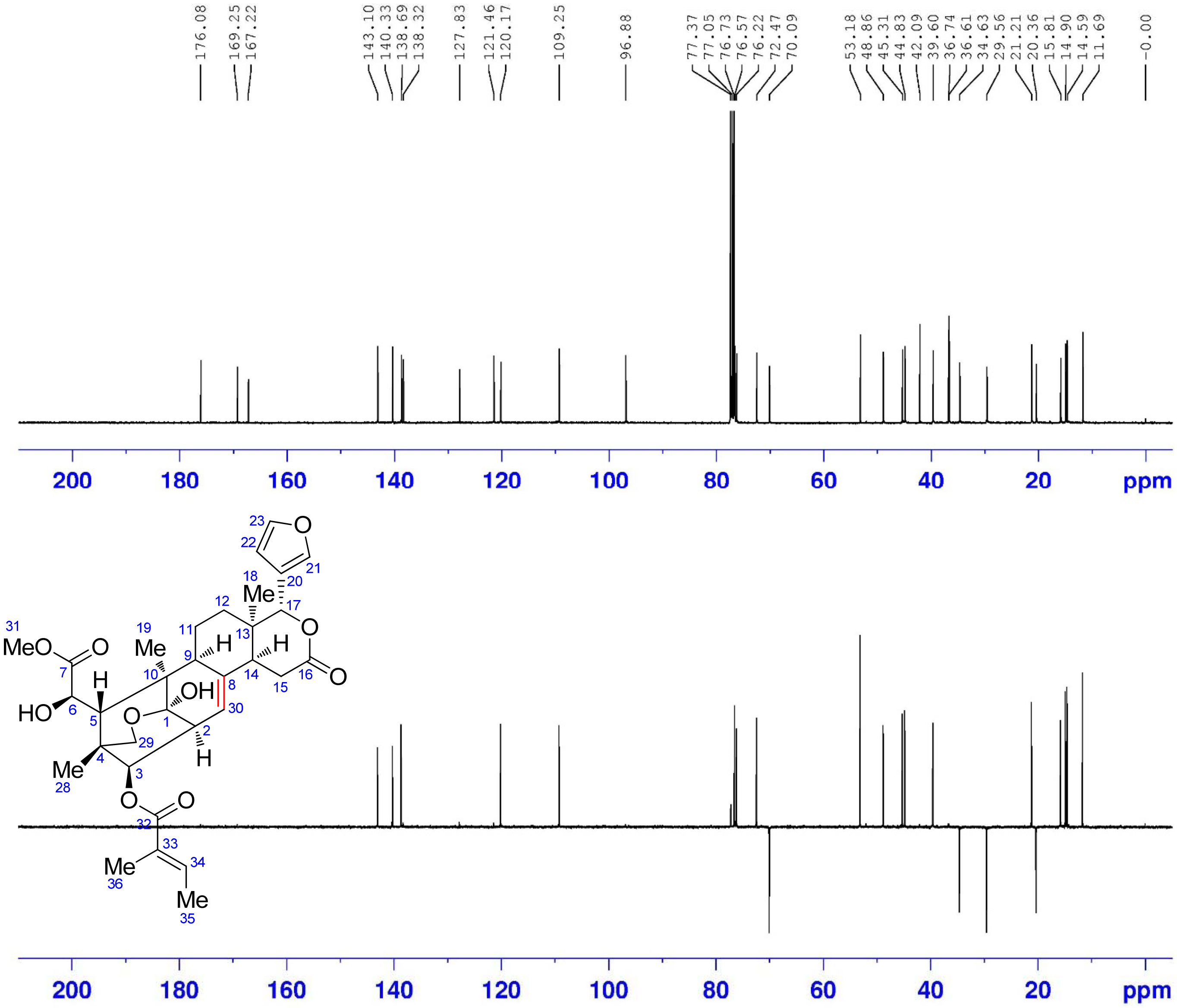

NAME	zxp-R-15-3-3
EXPNO	2
PROCNO	1
Date_	20180508
Time	7.05
INSTRUM	spect
PROBHD	5 mm CPPBBO BB
PULPROG	zgpg30
TD	65536
SOLVENT	CDCl3
NS	1024
DS	4
SWH	24038.461 Hz
FIDRES	0.366798 Hz
AQ	1.3631988 sec
RG	130.26
DW	20.800 usec
DE	18.00 usec
TE	297.0 K
D1	2.00000000 sec
D11	0.03000000 sec
TD0	1

======== CHANNEL f1 ========

SFO1	100.6233324 MHz
NUC1	13C
P1	10.00 usec
SI	32768
SF	100.6127690 MHz
WDW	EM
SSB	0
LB	1.00 Hz
GB	0
PC	1.40

DEPT135 (100 MHz) spectrum of compound **1** in $CDCl_3$

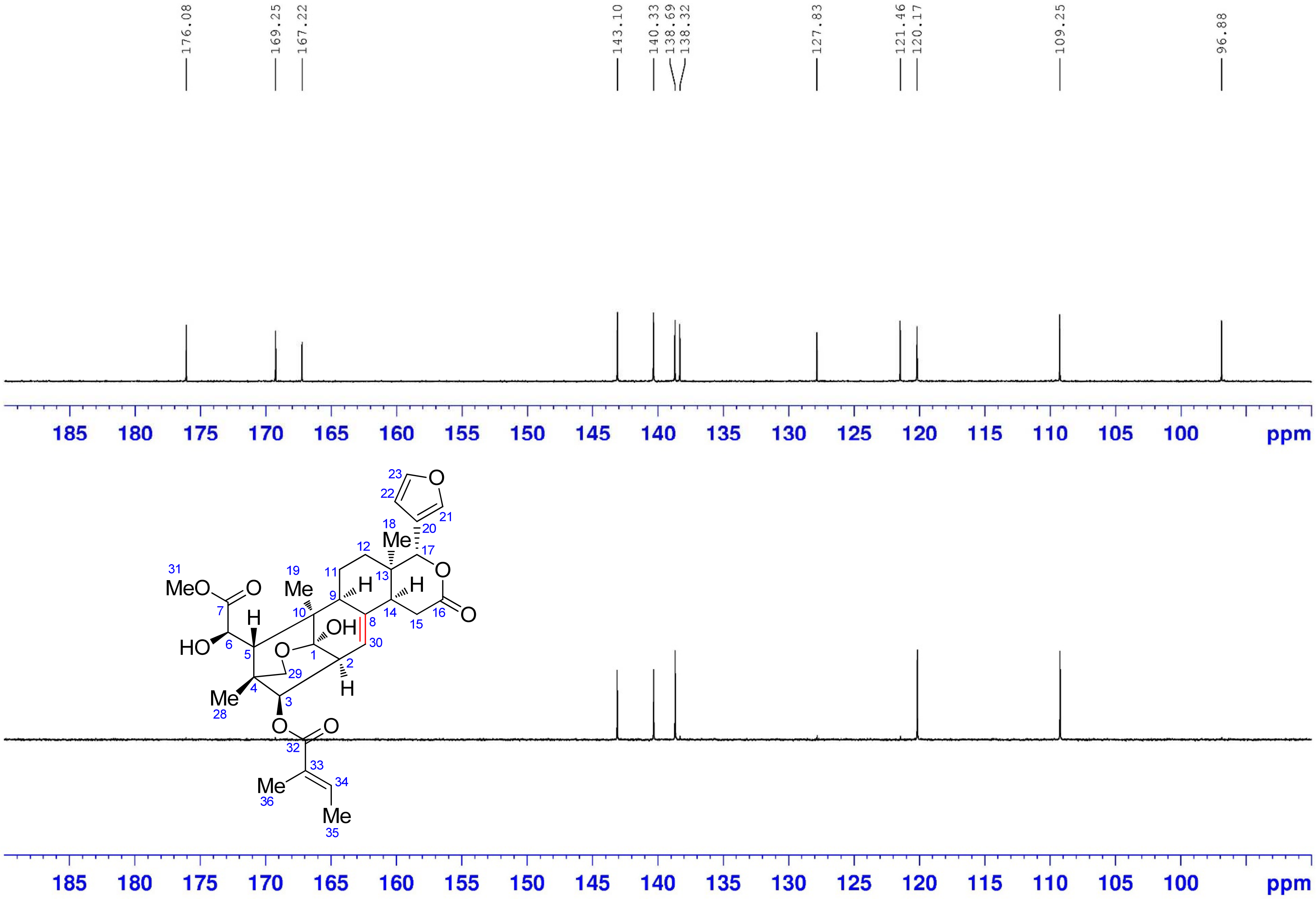

DEPT135 (100 MHz) spectrum of compound **1** in $CDCl_3$

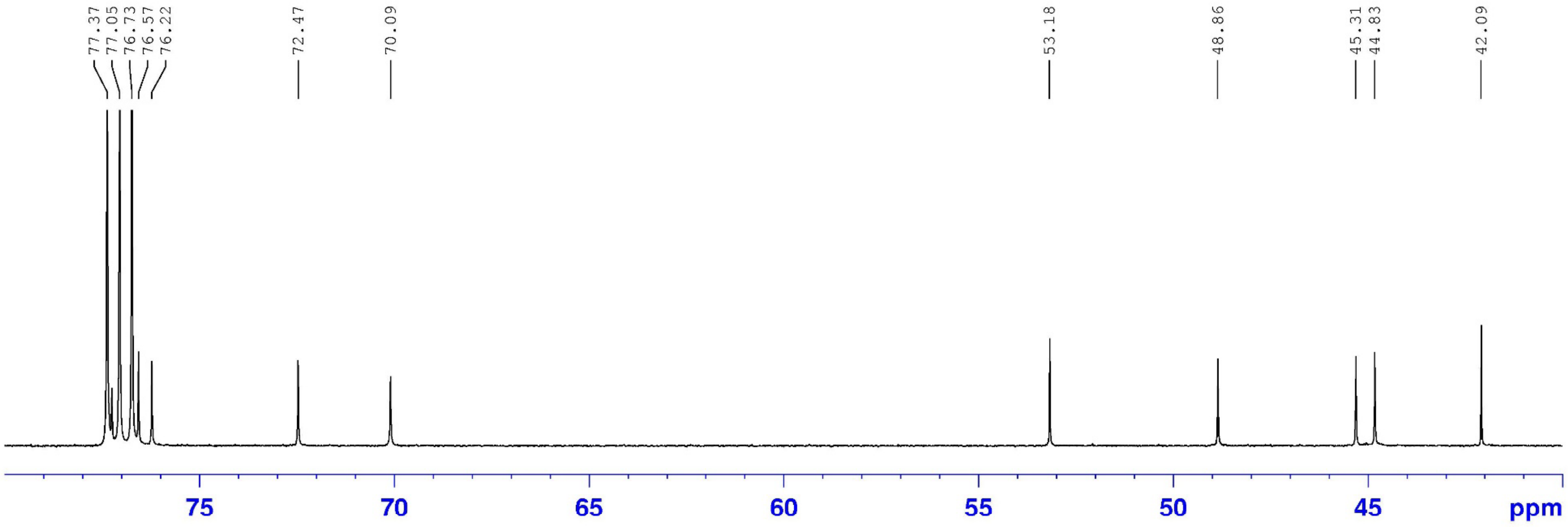

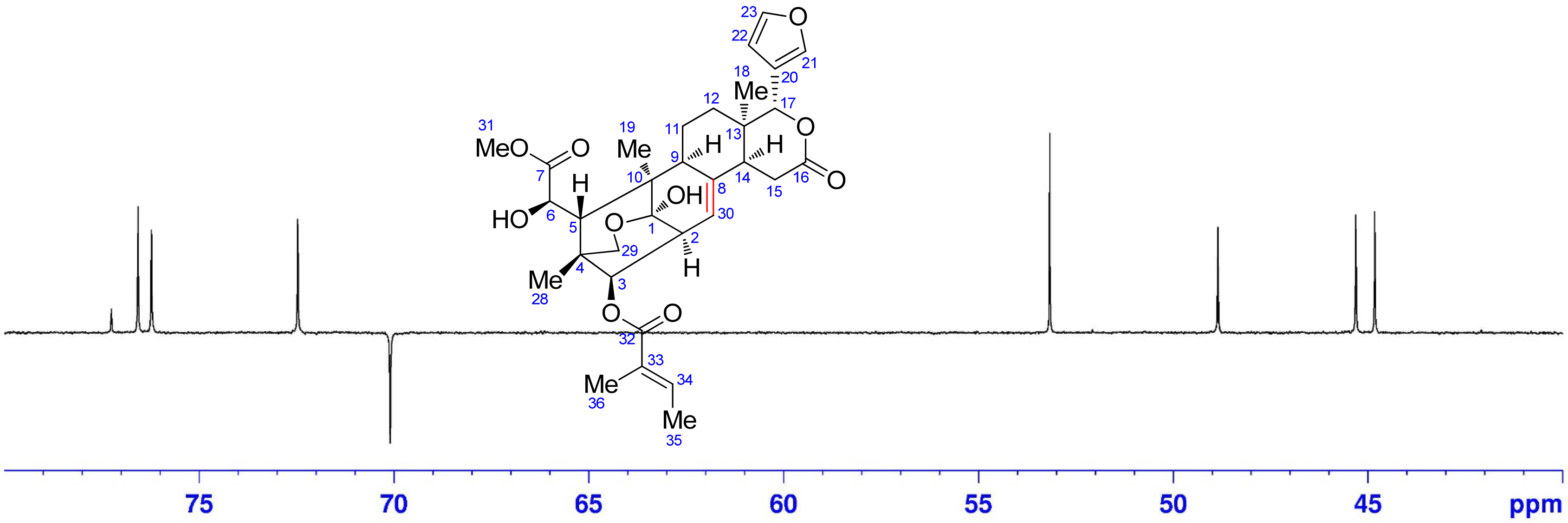

DEPT135 (100 MHz) spectrum of compound **1** in $CDCl_3$

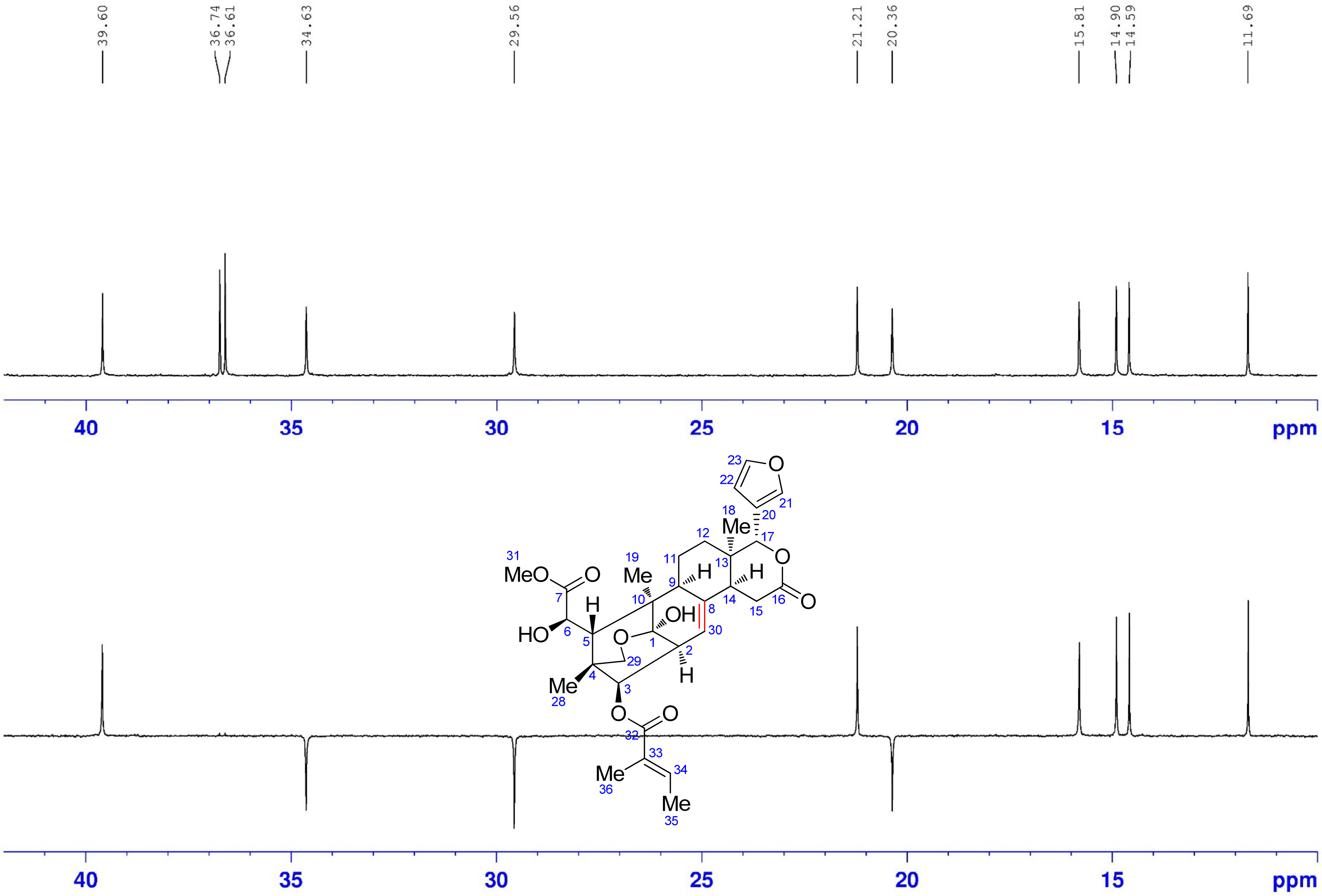

^{1}H-^{1}H COSY (400 MHz) spectrum of compound **1** in $CDCl_3$

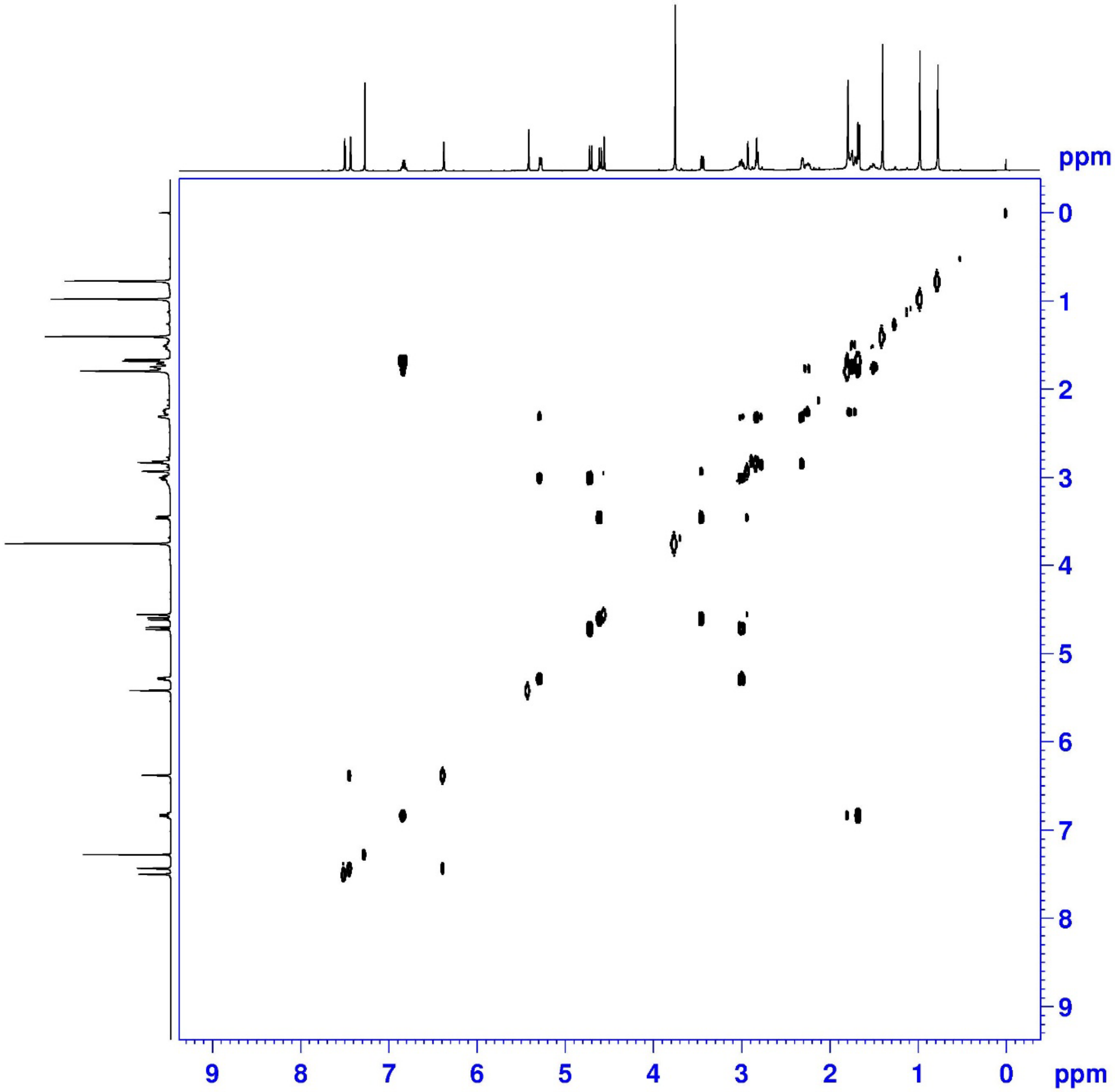

NAME	zxp-R-15-3-3	
EXPNO	4	
PROCNO	1	
Date_	20180605	
Time	22.56	
INSTRUM	spect	
PROBHD	5 mm CPPBBO BB	
PULPROG	cosygpppqf	
TD	2048	
SOLVENT	CDCl3	
NS	8	
DS	8	
SWH	3906.250	Hz
FIDRES	1.907349	Hz
AQ	0.2621940	sec
RG	91.64	
DW	128.000	usec
DE	10.00	usec
TE	297.0	K
D0	0.00000300	sec
D1	1.89678097	sec
D11	0.03000000	sec
D12	0.00002000	sec
D13	0.00000400	sec
D16	0.00020000	sec
IN0	0.00025600	sec
======== CHANNEL f1 ========		
SFO1	400.1318006	MHz
NUC1	1H	
P0	11.50	usec
P1	11.50	usec
P17	2500.00	usec
ND0	1	
TD	128	
SFO1	400.1318	MHz
FIDRES	30.517578	Hz
SW	9.762	ppm
FnMODE	QF	
SI	1024	
SF	400.1300020	MHz
WDW	QSINE	
SSB	0	
LB	0.00	Hz
GB	0	
PC	1.40	
SI	1024	
MC2	QF	
SF	400.1300017	MHz
WDW	QSINE	
SSB	0	
LB	0.00	Hz
GB	0	

^{1}H-^{1}H COSY (400 MHz) spectrum of compound **1** in $CDCl_3$

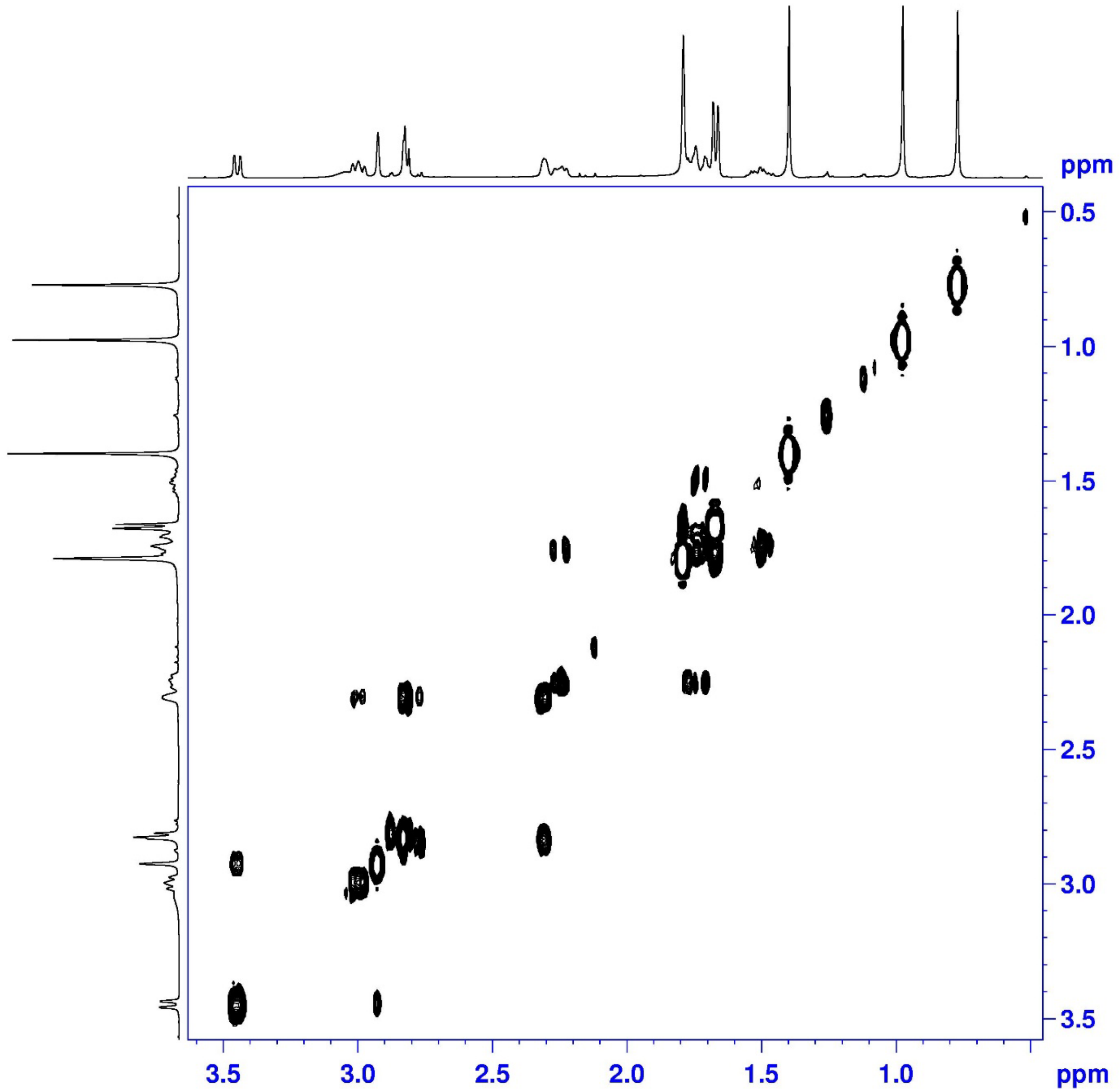

HSQC (400 MHz) spectrum of compound **1** in $CDCl_3$

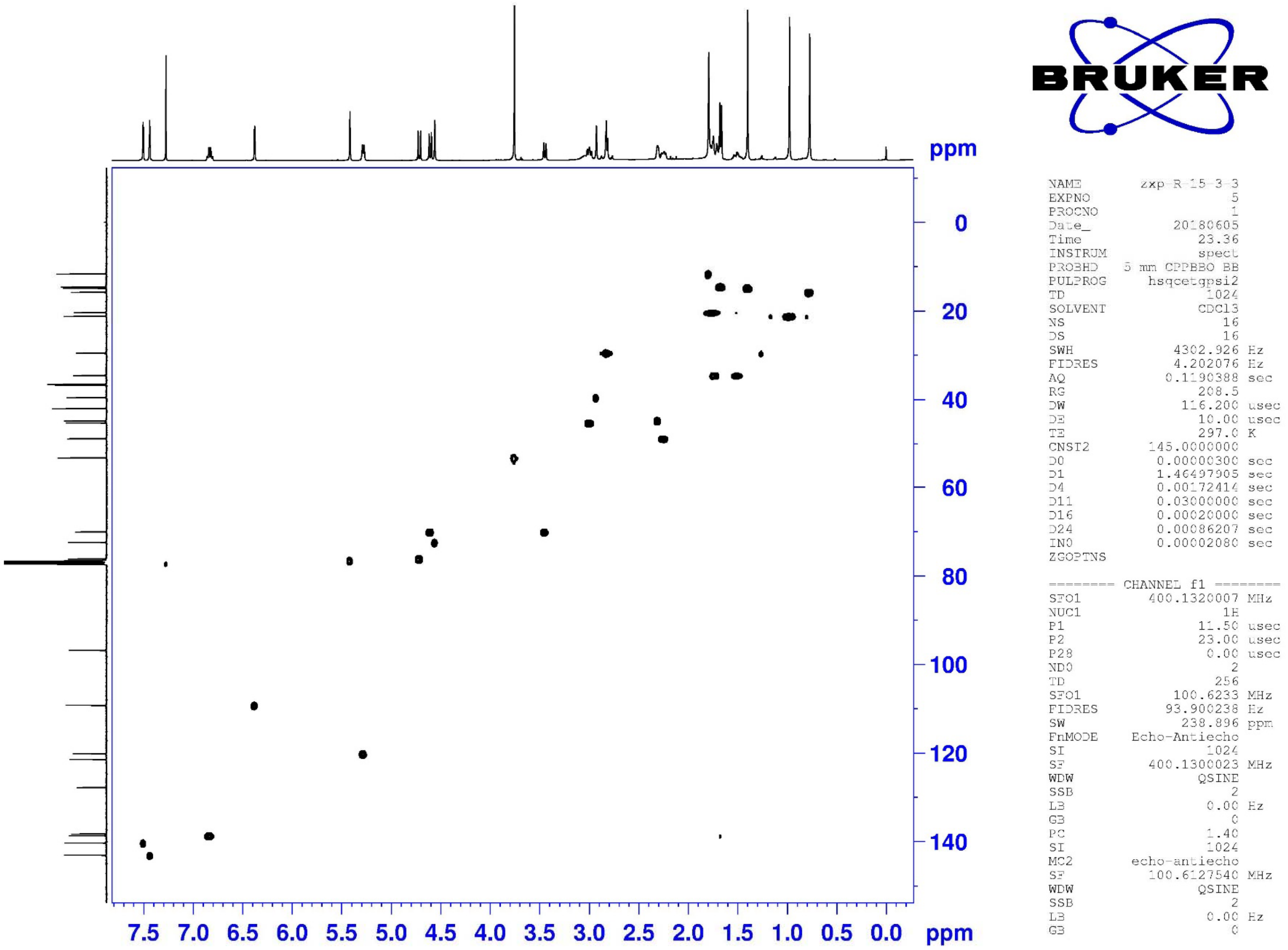

HSQC (400 MHz) spectrum of compound **1** in $CDCl_3$

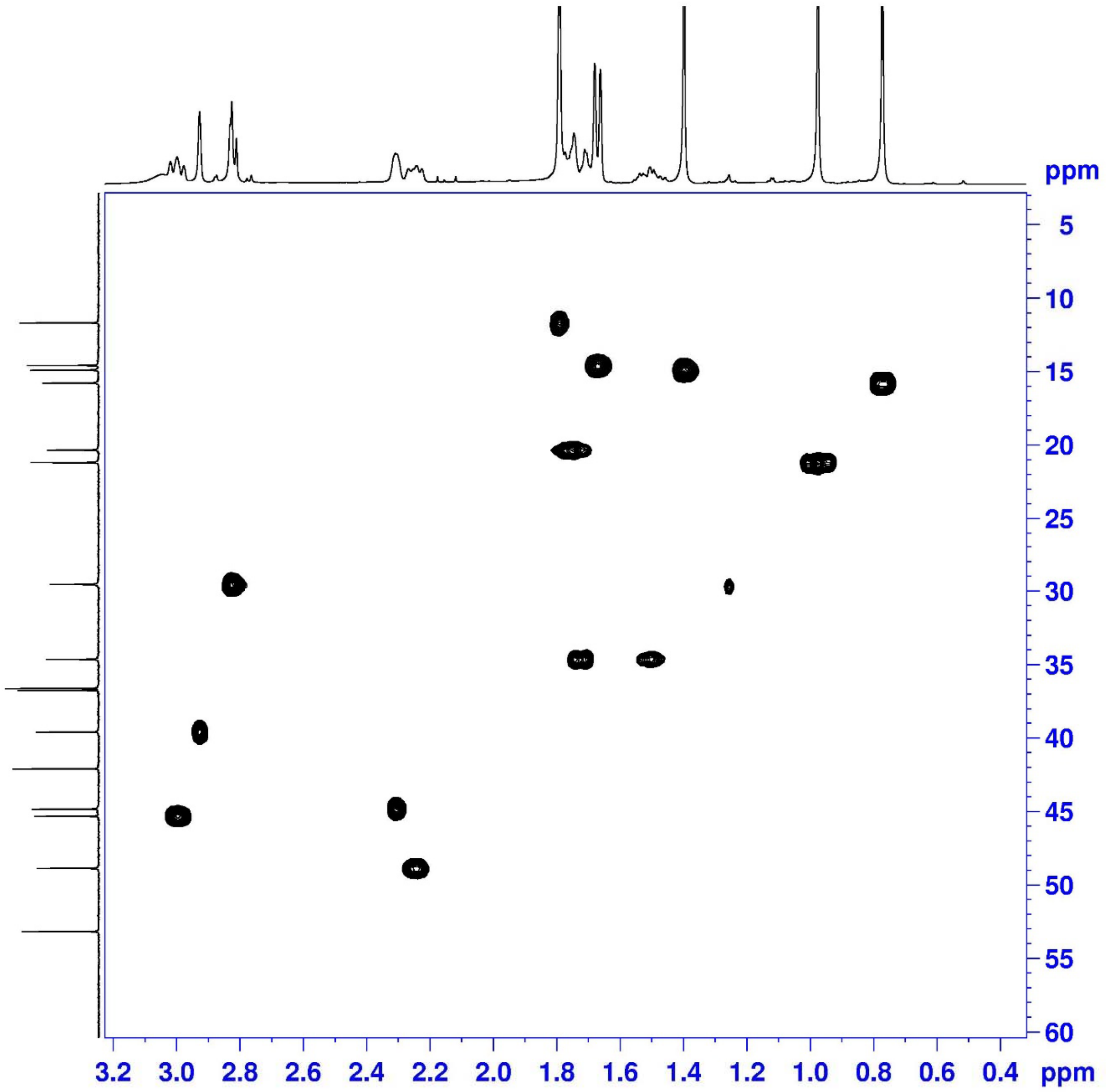

HMBC (400 MHz) spectrum of compound **1** in $CDCl_3$

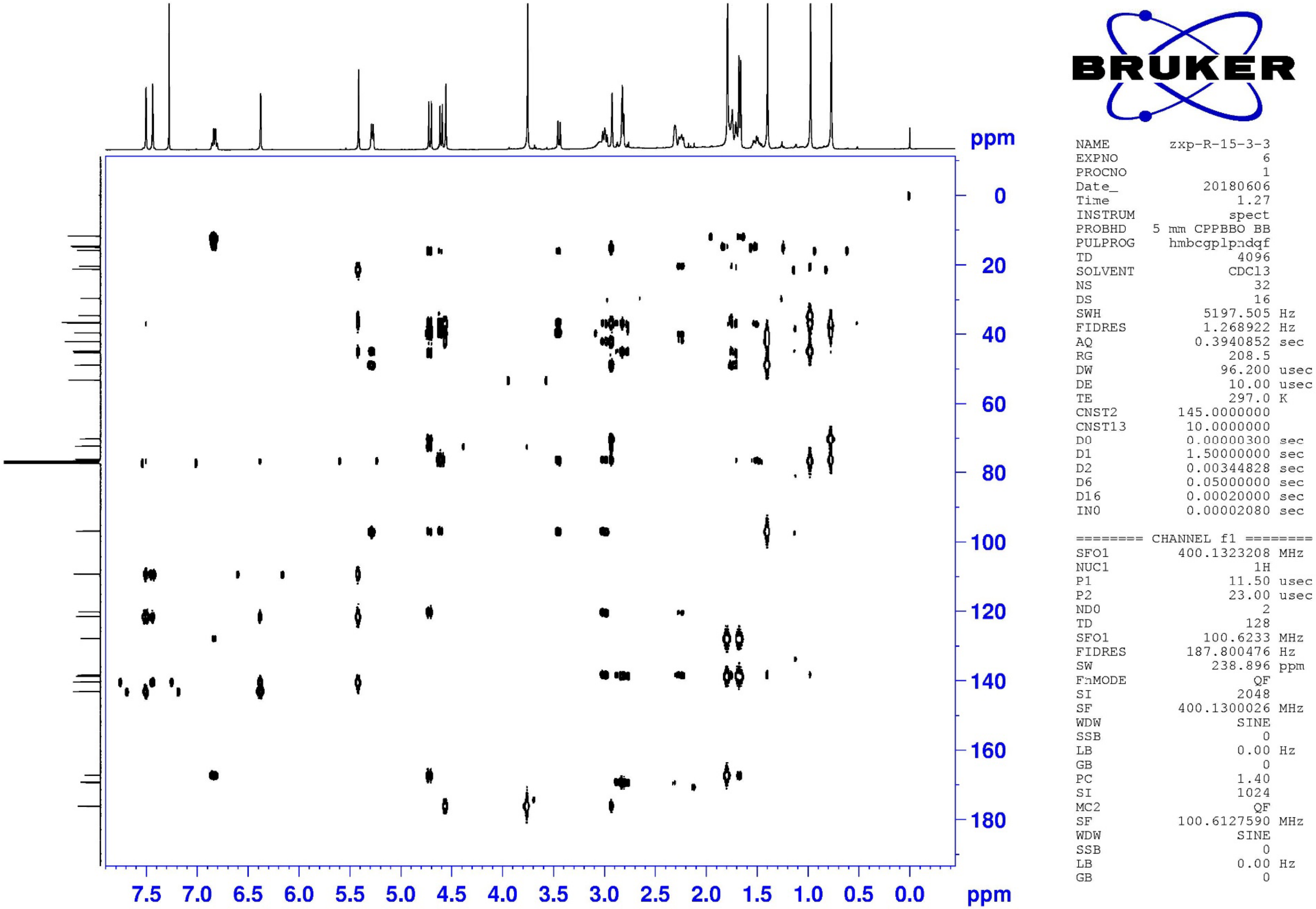

HMBC (400 MHz) spectrum of compound **1** in $CDCl_3$

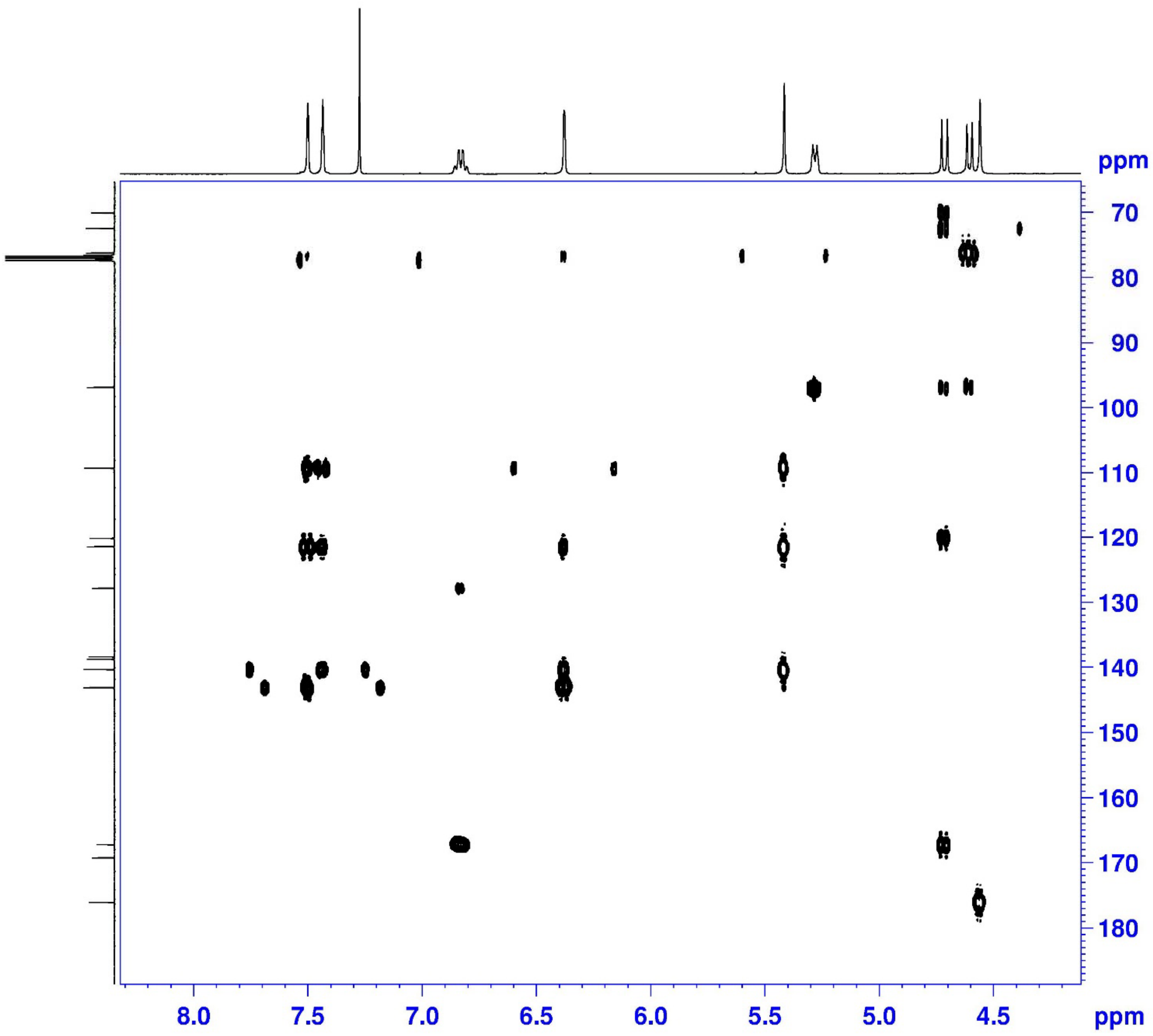

HMBC (400 MHz) spectrum of compound **1** in $CDCl_3$

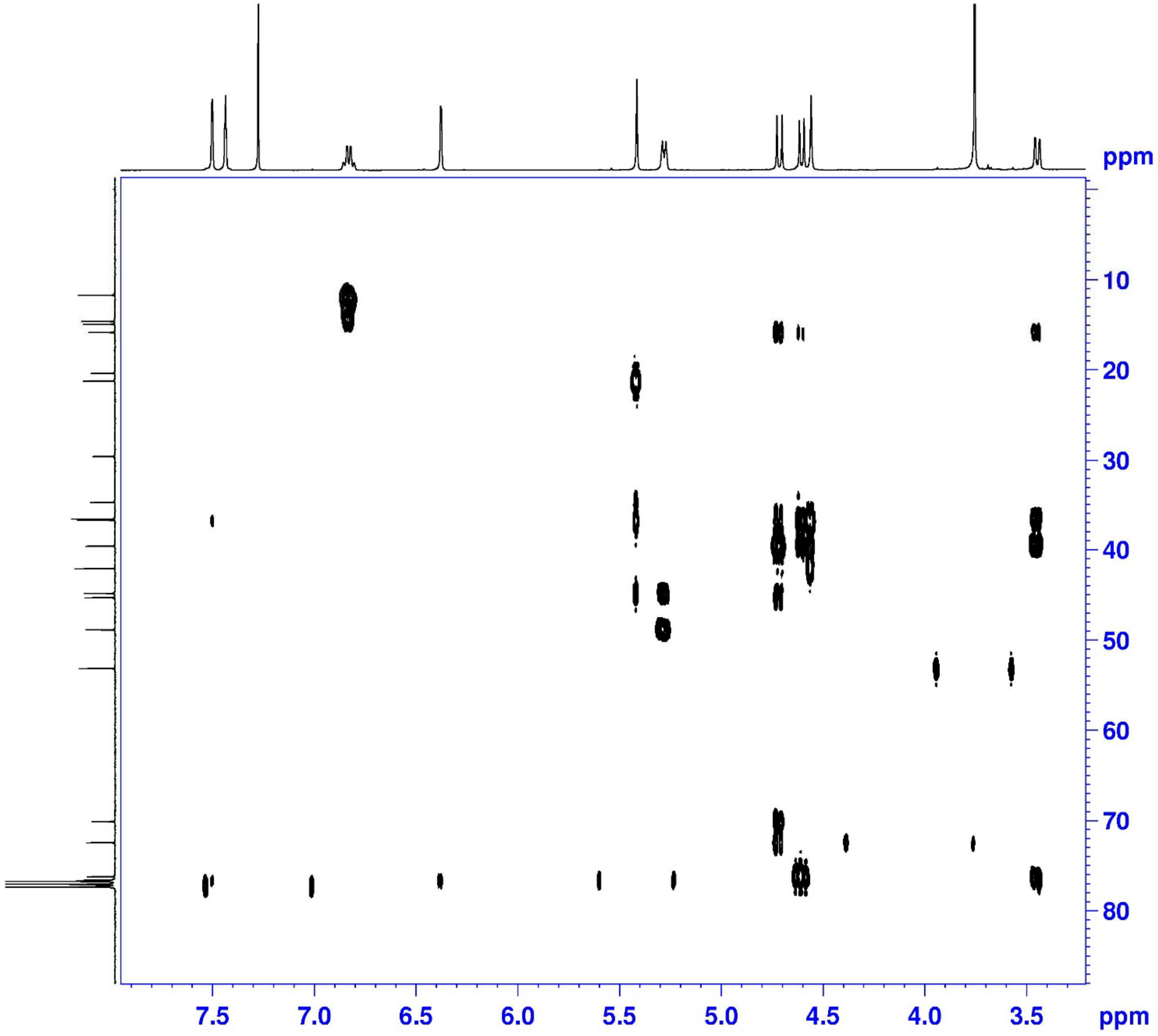

HMBC (400 MHz) spectrum of compound **1** in $CDCl_3$

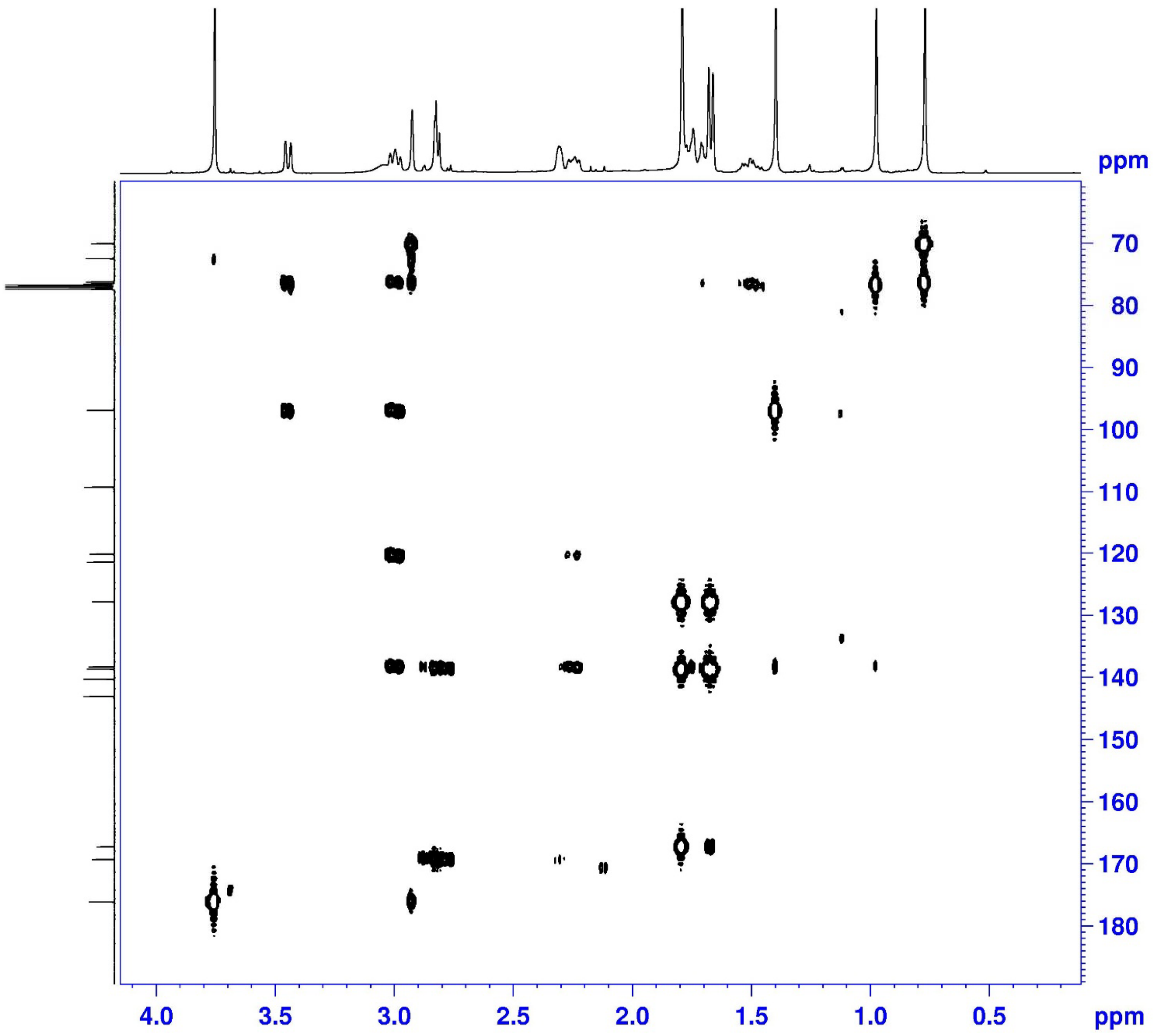

HMBC (400 MHz) spectrum of compound **1** in $CDCl_3$

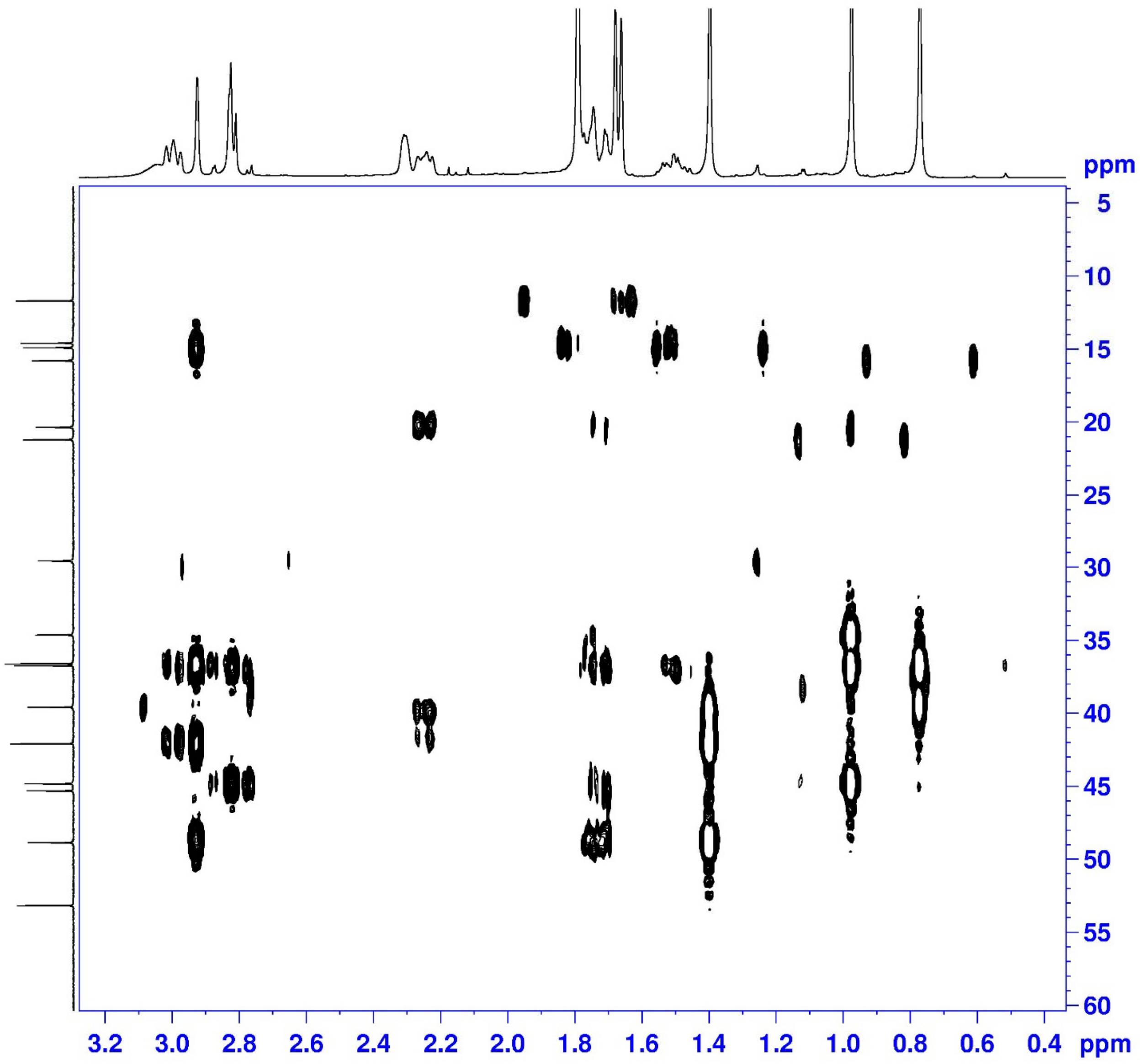

NOESY (400 MHz) spectrum of compound **1** in $CDCl_3$

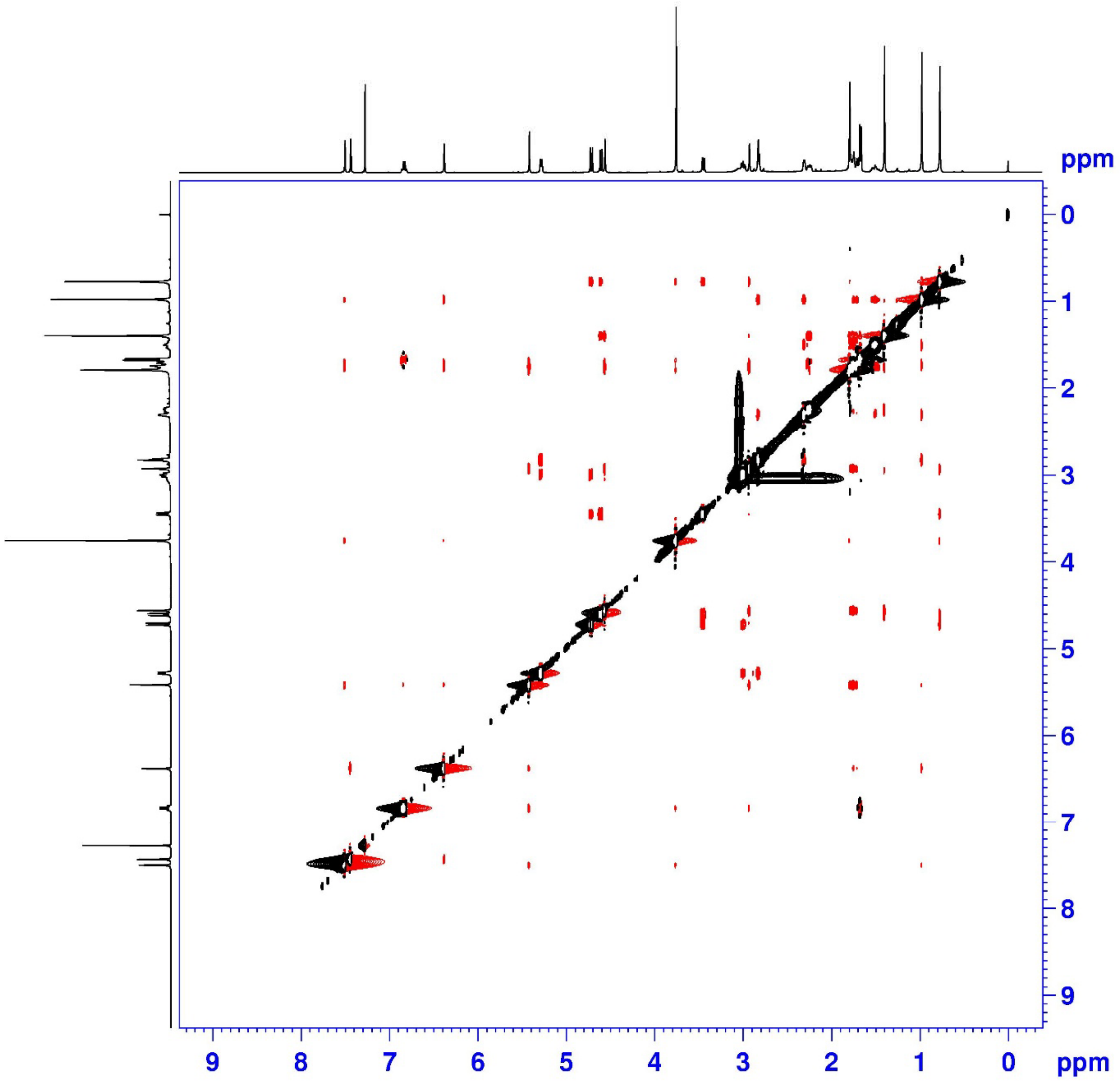

NAME	ZXP-R-15-3-3	
EXPNO	7	
PROCNO	1	
Date_	20180606	
Time	3.42	
INSTRUM	spect	
PROBHD	5 mm CPPBBO BB	
PULPROG	noesygpphpp	
TD	2048	
SOLVENT	CDCl3	
NS	16	
DS	32	
SWH	4000.000	Hz
FIDRES	1.953125	Hz
AQ	0.2560500	sec
RG	65.23	
DW	125.000	usec
DE	10.00	usec
TE	297.0	K
D0	0.00011036	sec
D1	1.99385595	sec
D8	0.30000001	sec
D11	0.03000000	sec
D12	0.00002000	sec
D16	0.00020000	sec
IN0	0.00025000	sec
======== CHANNEL f1 ========		
SFO1	400.1318006	MHz
NUC1	1H	
P1	11.50	usec
P2	23.00	usec
P17	2500.00	usec
ND0	1	
TD	256	
SFO1	400.1318	MHz
FIDRES	15.625000	Hz
SW	9.997	ppm
FnMODE	States-TPPI	
SI	1024	
SF	400.1300018	MHz
WDW	QSINE	
SSB	2	
LB	0.00	Hz
GB	0	
PC	1.00	
SI	1024	
MC2	States-TPPI	
SF	400.1300021	MHz
WDW	QSINE	
SSB	2	
LB	0.00	Hz
GB	0	

NOESY (400 MHz) spectrum of compound **1** in $CDCl_3$

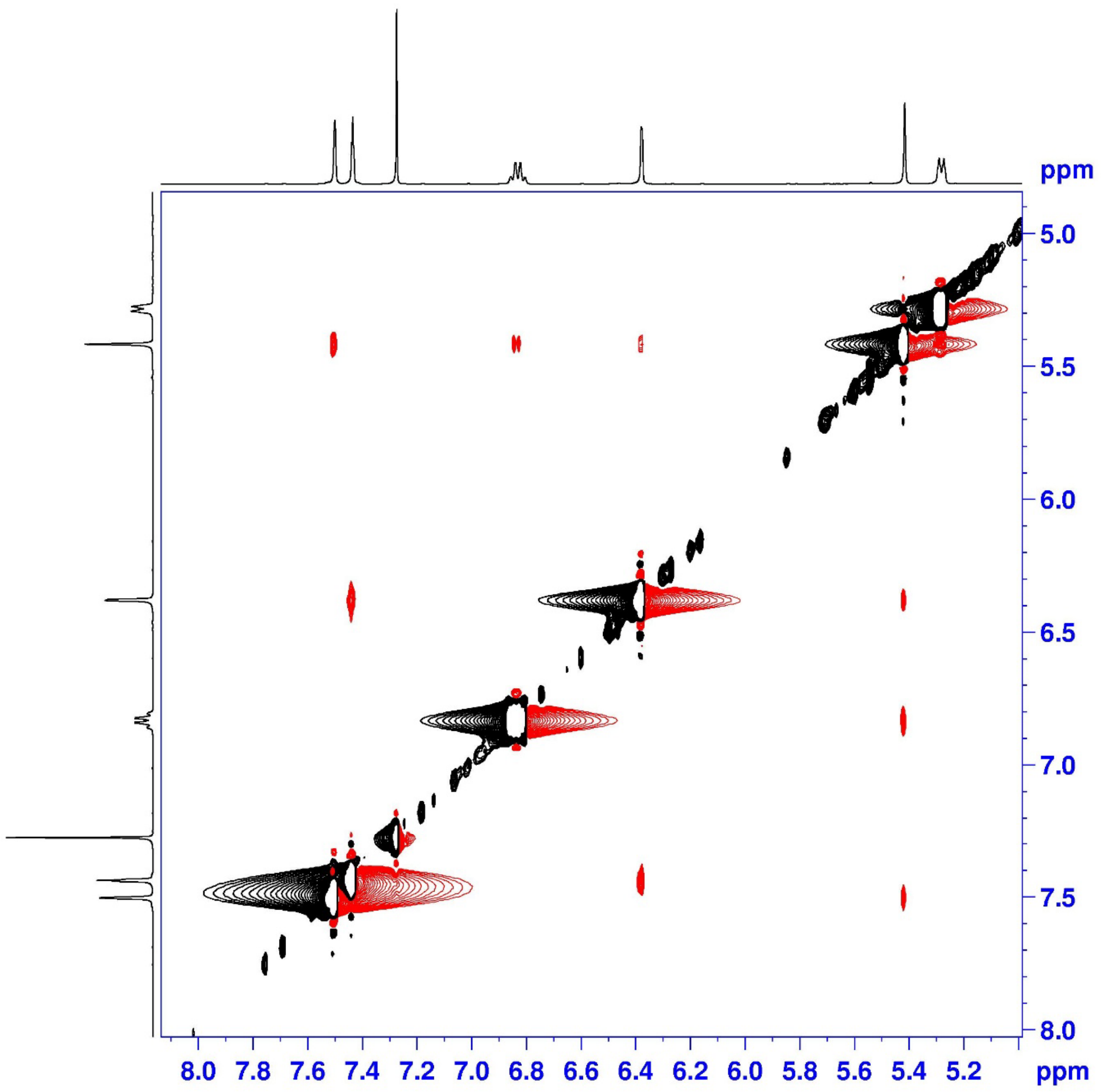

NOESY (400 MHz) spectrum of compound **1** in $CDCl_3$

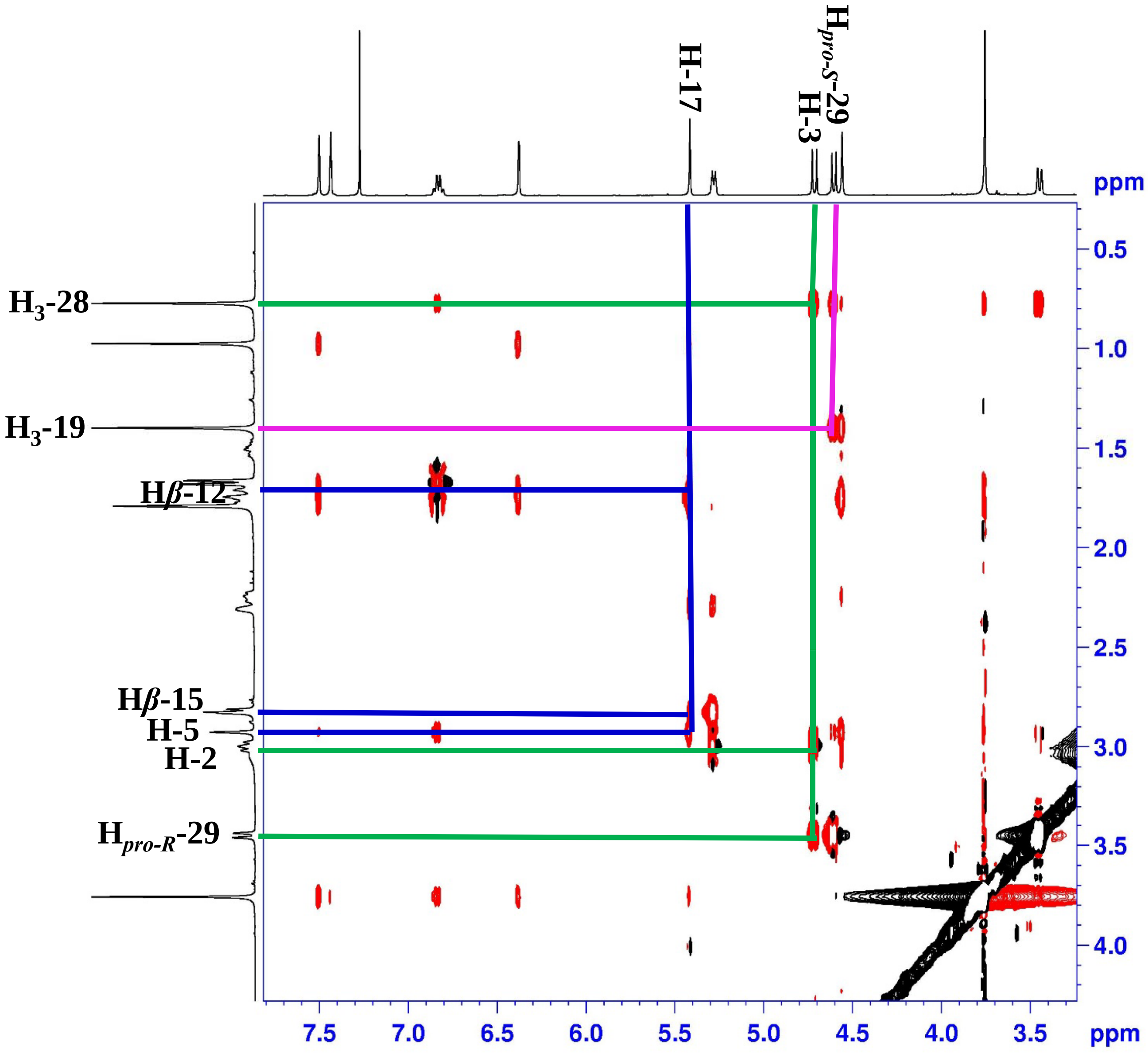

NOESY (400 MHz) spectrum of compound **1** in $CDCl_3$

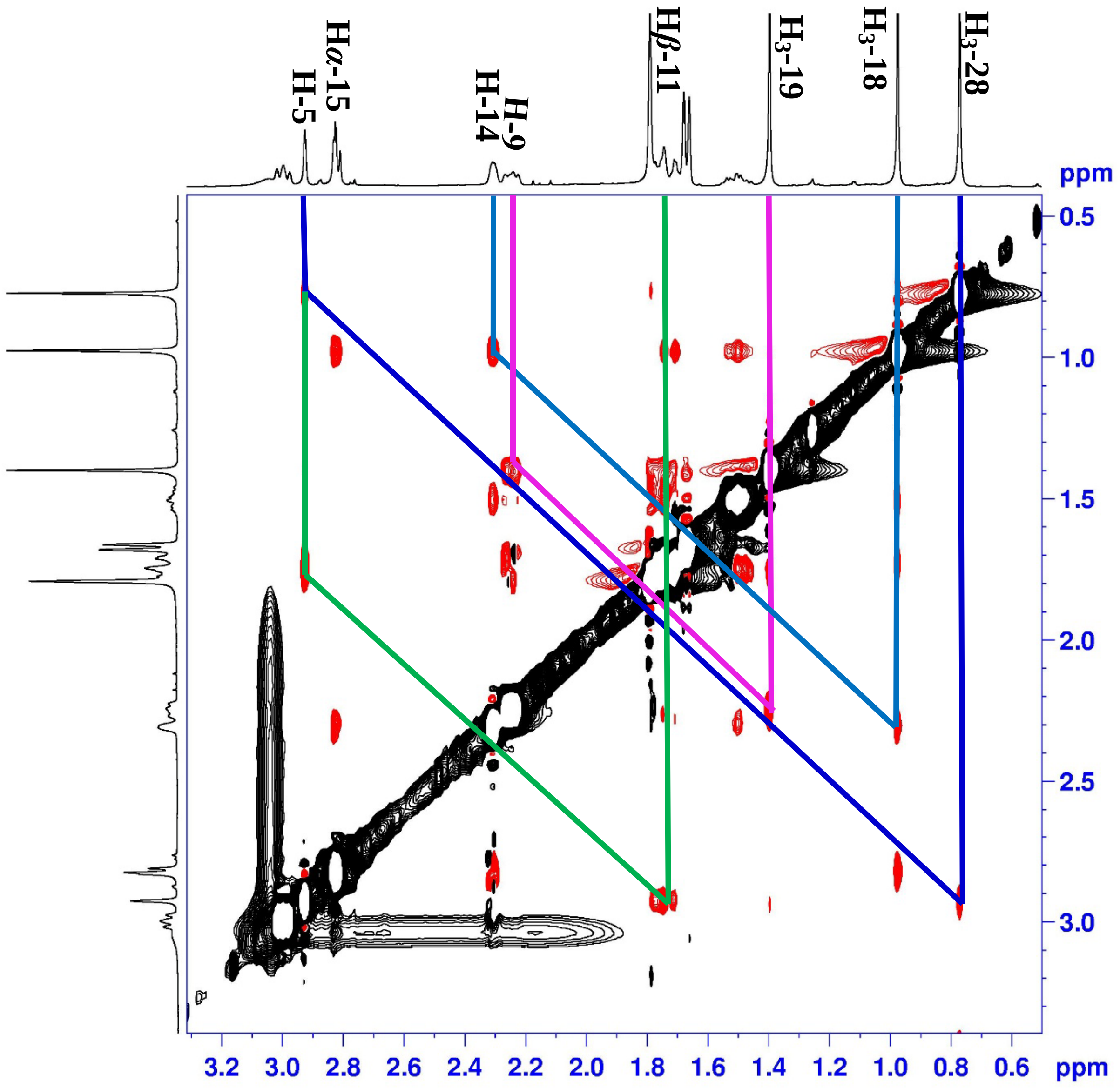

HR-ESIMS for compound **2**

Generic Display Report

**Analysis Info**		Acquisition Date	9/7/2018 5:05:23 PM
Analysis Name	D:\Data\MS\data\201809\zhouxiaopeng_ZXP-3_pos_5_01_5427.d		
Method	LC_Direct Infusion_pos_100-1000mz.m	Operator	SCSIO
Sample Name	zhouxiaopeng_ZXP-3_pos	Instrument	maXis
Comment			

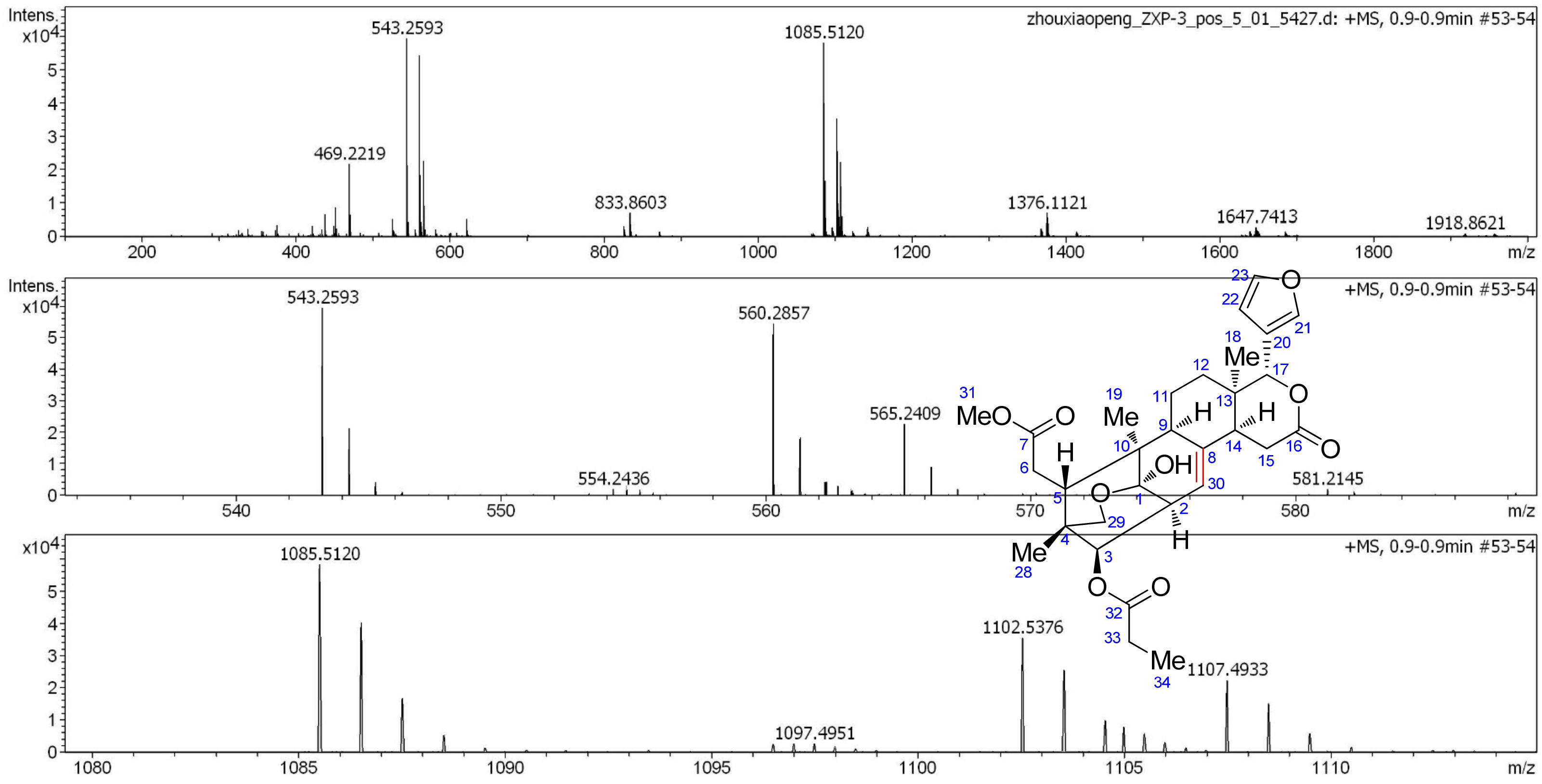

HR-ESIMS for compound **2**

Mass Spectrum SmartFormula Report

Analysis Info

Analysis Name	D:\Data\MS\data\201809\zhouxiaopeng_ZXP-3_pos_5_01_5427.d	Acquisition Date	9/7/2018 5:05:23 PM
Method	LC_Direct Infusion_pos_100-1000mz.m	Operator	SCSIO
Sample Name	zhouxiaopeng_ZXP-3_pos	Instrument	maXis 255552.00029
Comment			

Acquisition Parameter

Source Type	ESI	Ion Polarity	Positive	Set Nebulizer	0.4 Bar
Focus	Active	Set Capillary	4500 V	Set Dry Heater	180 °C
Scan Begin	100 m/z	Set End Plate Offset	-500 V	Set Dry Gas	4.0 l/min
Scan End	2000 m/z	Set Charging Voltage	0 V	Set Divert Valve	Waste
		Set Corona	0 nA	Set APCI Heater	0 °C

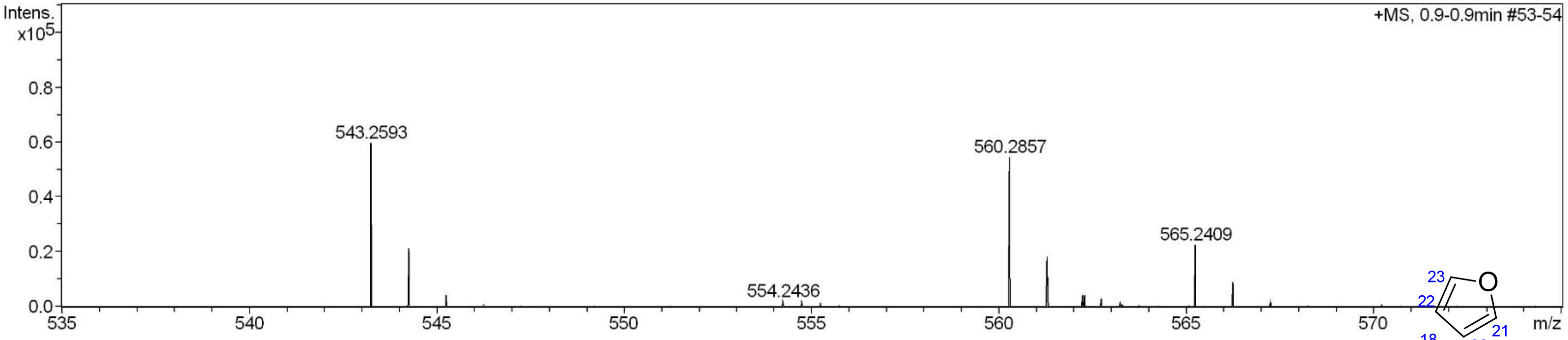

Meas. m/z	#	Ion Formula	Score	m/z	err [ppm]	err [mDa]	mSigma	rdb	e⁻ Conf	N-Rule
543.2593	1	C30H39O9	100.00	543.2589	0.7	0.4	11.9	11.5	even	ok
565.2409	1	C30H38NaO9	100.00	565.2408	-0.2	-0.1	34.2	11.5	even	ok
1085.5120	1	C60H77O18	100.00	1085.5104	1.4	1.5	20.5	22.5	even	ok
1107.4933	1	C60H76NaO18	100.00	1107.4924	-0.8	-0.9	4.6	22.5	even	ok

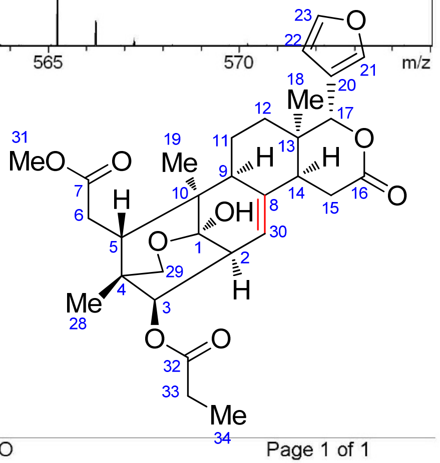

zhouxiaopeng_ZXP-3_pos_5_01_5427.d

Bruker Compass DataAnalysis 4.1 printed: 9/7/2018 5:08:48 PM by: SCSIO Page 1 of 1

^{1}H NMR (400 MHz) spectrum of compound **2** in $CDCl_3$

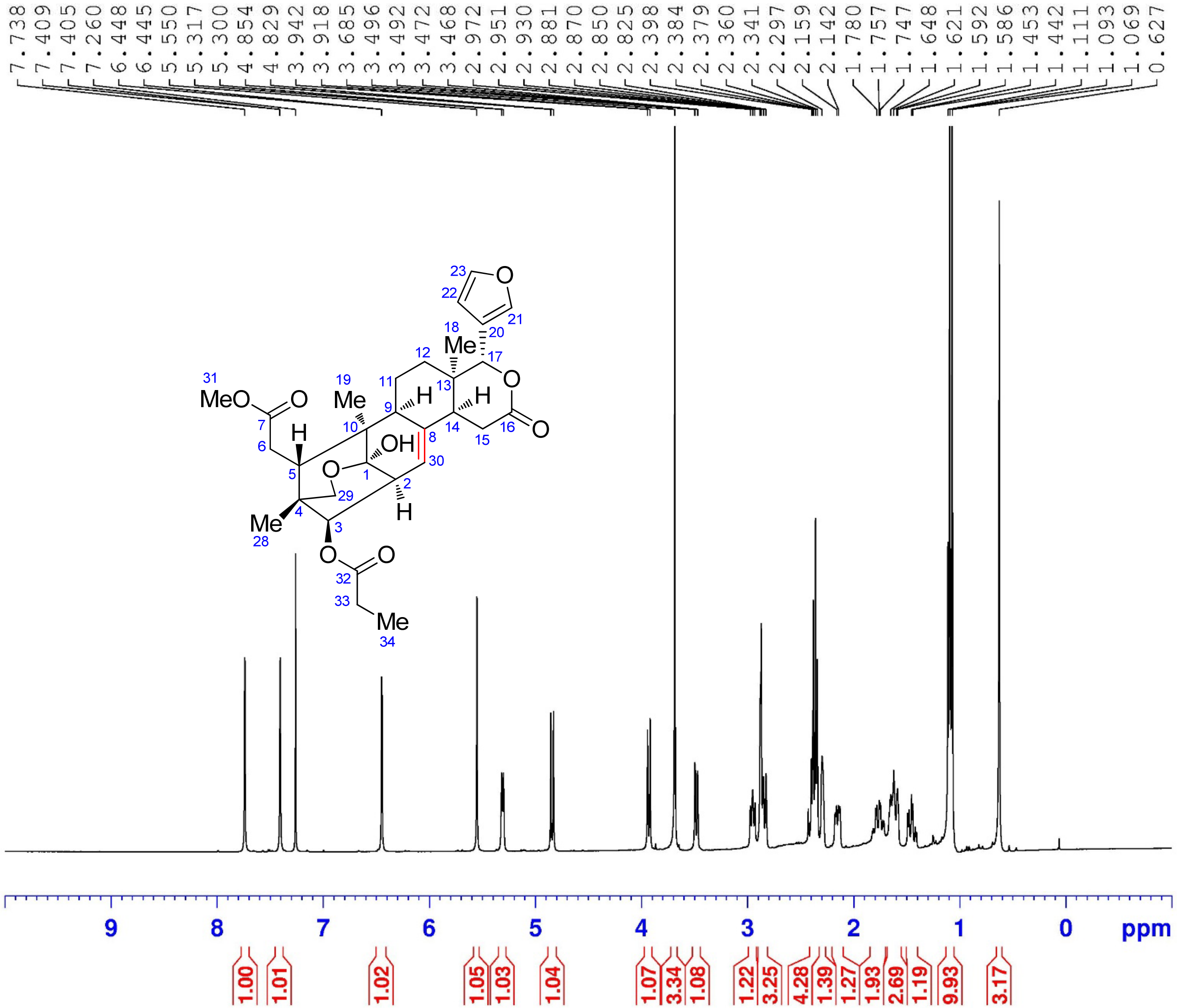

NAME ZXP-R-17-4-2-2-2
EXPNO 1
PROCNO 1
Date_ 20180818
Time 13.44
INSTRUM spect
PROBHD 5 mm CPPBBO BB
PULPROG zg30
TD 65536
SOLVENT CDCl3
NS 16
DS 2
SWH 8223.685 Hz
FIDRES 0.125483 Hz
AQ 3.9846387 sec
RG 117.37
DW 60.800 usec
DE 10.00 usec
TE 297.0 K
D1 1.00000000 sec
TD0 1

======== CHANNEL f1 ========
SFO1 400.1324710 MHz
NUC1 1H
P1 11.50 usec
SI 65536
SF 400.1300095 MHz
WDW EM
SSB 0
LB 0.30 Hz
GB 0
PC 1.00

^{1}H NMR (400 MHz) spectrum of compound **2** in $CDCl_3$

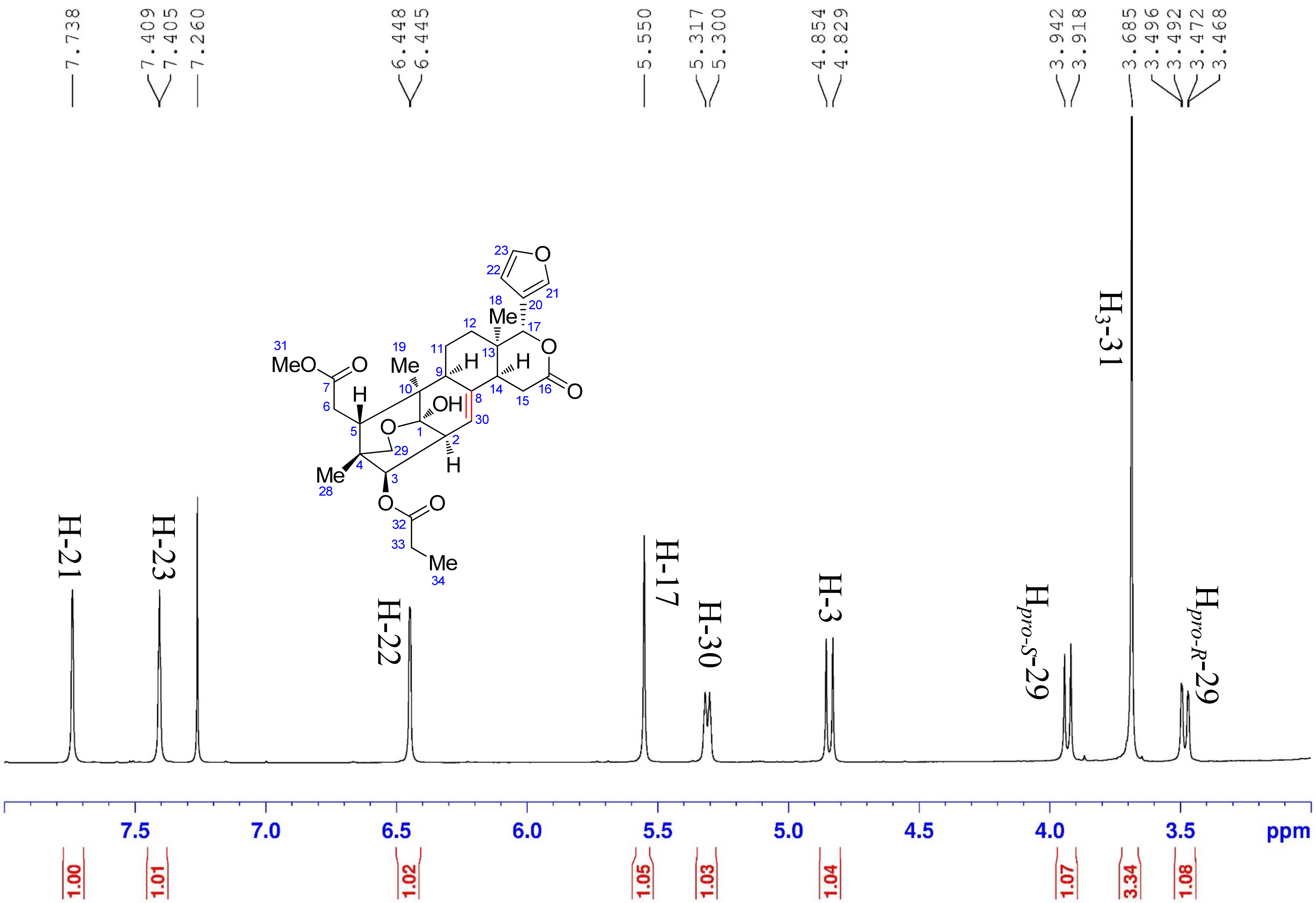

^{1}H NMR (400 MHz) spectrum of compound **2** in $CDCl_3$

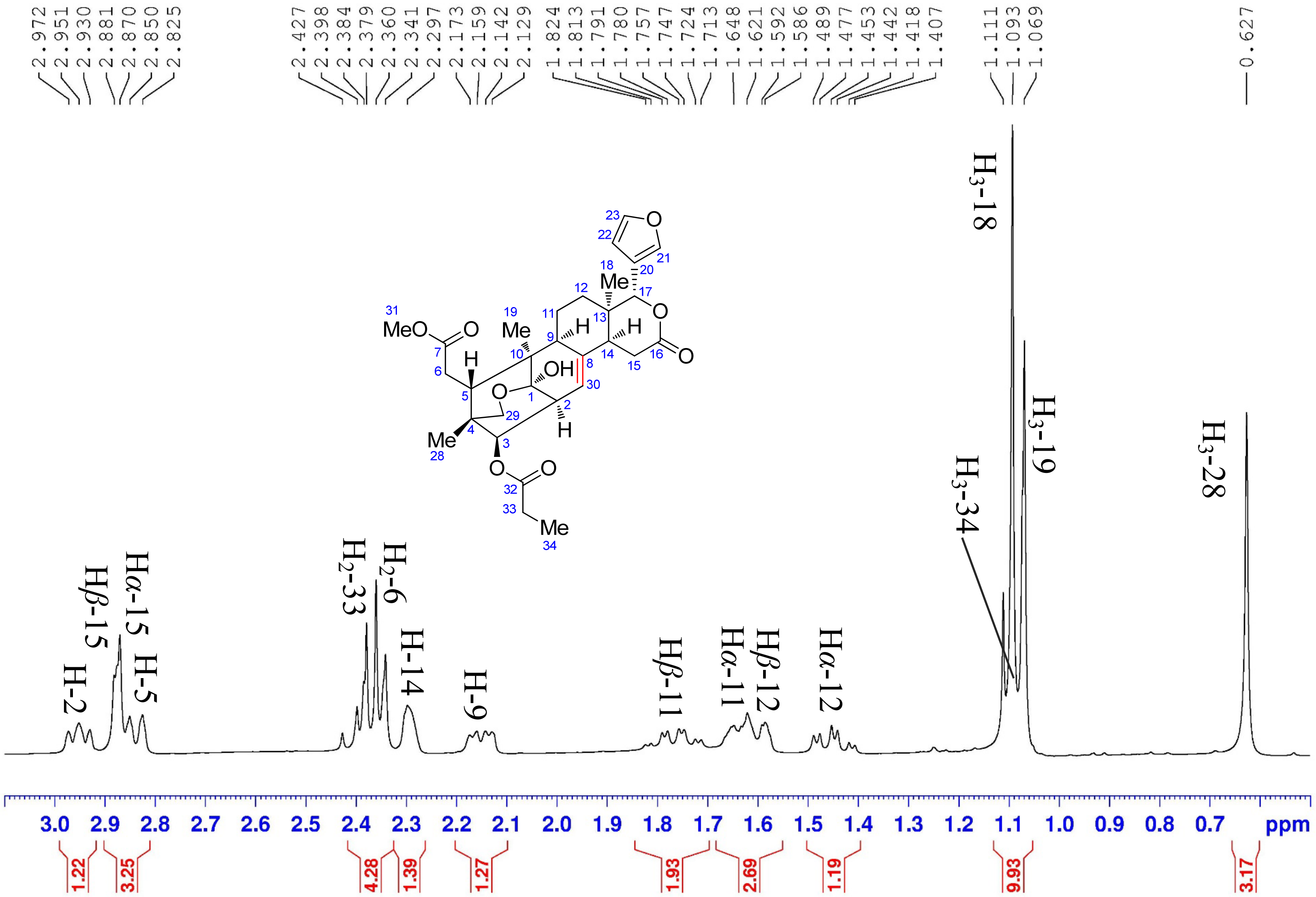

^{13}C NMR (100 MHz) spectrum of compound **2** in $CDCl_3$

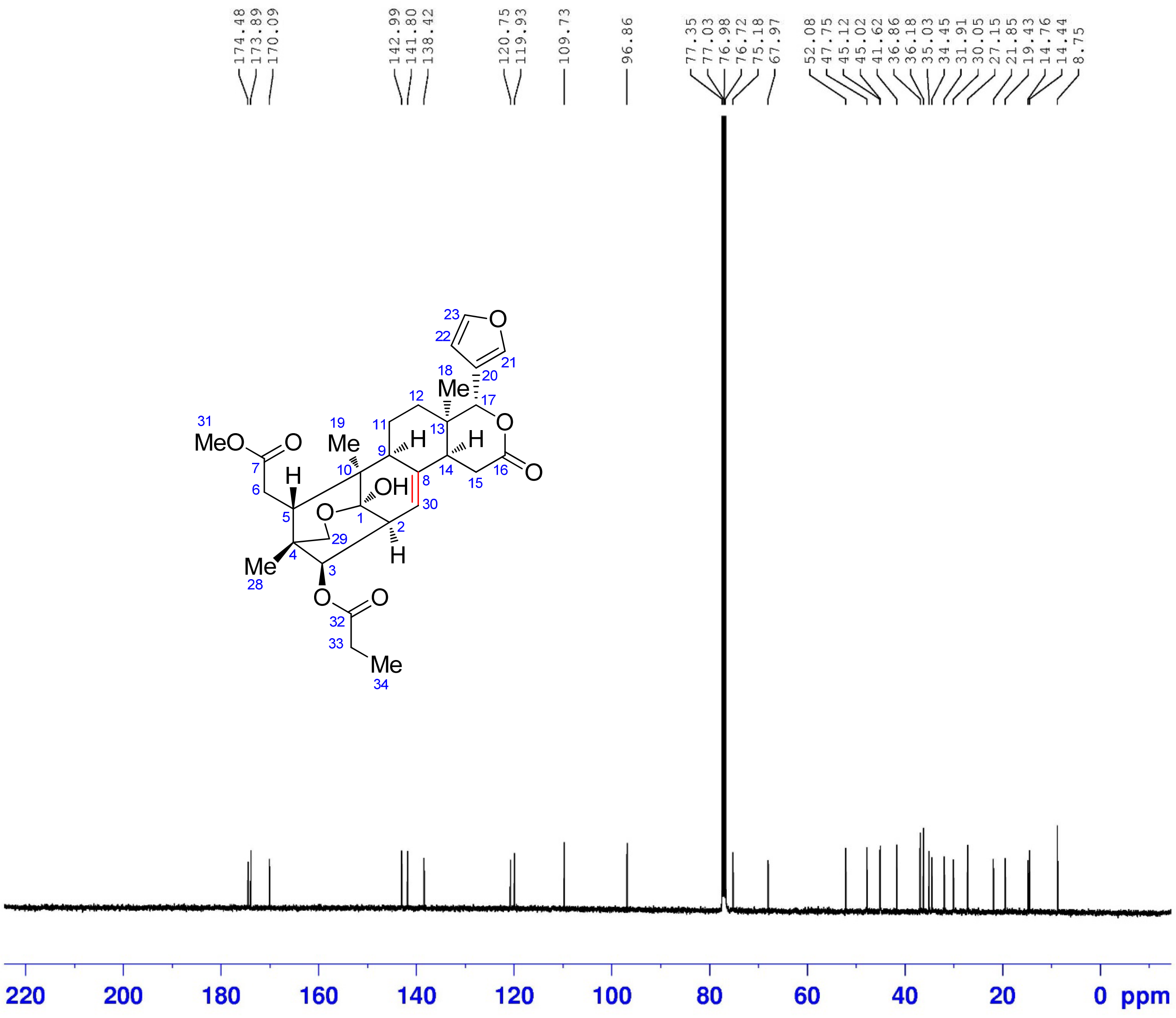

NAME	ZXP-R-17-4-2-2-2
EXPNO	2
PROCNO	1
Date_	20180818
Time	14.44
INSTRUM	spect
PROBHD	5 mm CPPBBO BB
PULPROG	zgpg30
TD	65536
SOLVENT	CDCl3
NS	1024
DS	4
SWH	24038.461 Hz
FIDRES	0.366798 Hz
AQ	1.3631988 sec
RG	130.26
DW	20.800 usec
DE	18.00 usec
TE	297.0 K
D1	2.00000000 sec
D11	0.03000000 sec
TD0	1

======== CHANNEL f1 ========

SFO1	100.6233324 MHz
NUC1	13C
P1	10.00 usec
SI	32768
SF	100.6127692 MHz
WDW	EM
SSB	0
LB	1.00 Hz
GB	0
PC	1.40

^{13}C NMR (100 MHz) spectrum of compound **2** in $CDCl_3$

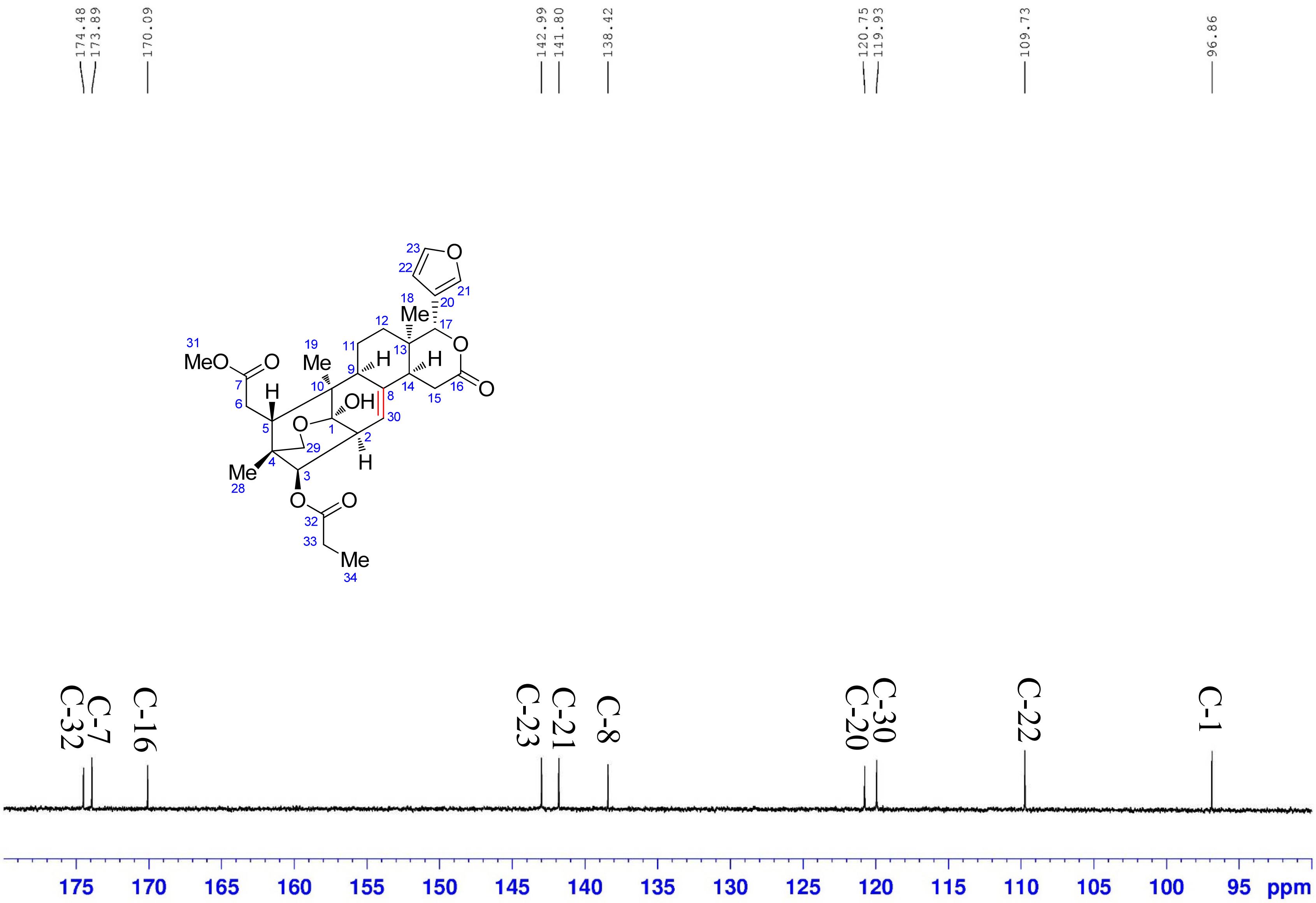

^{13}C NMR (100 MHz) spectrum of compound **2** in $CDCl_3$

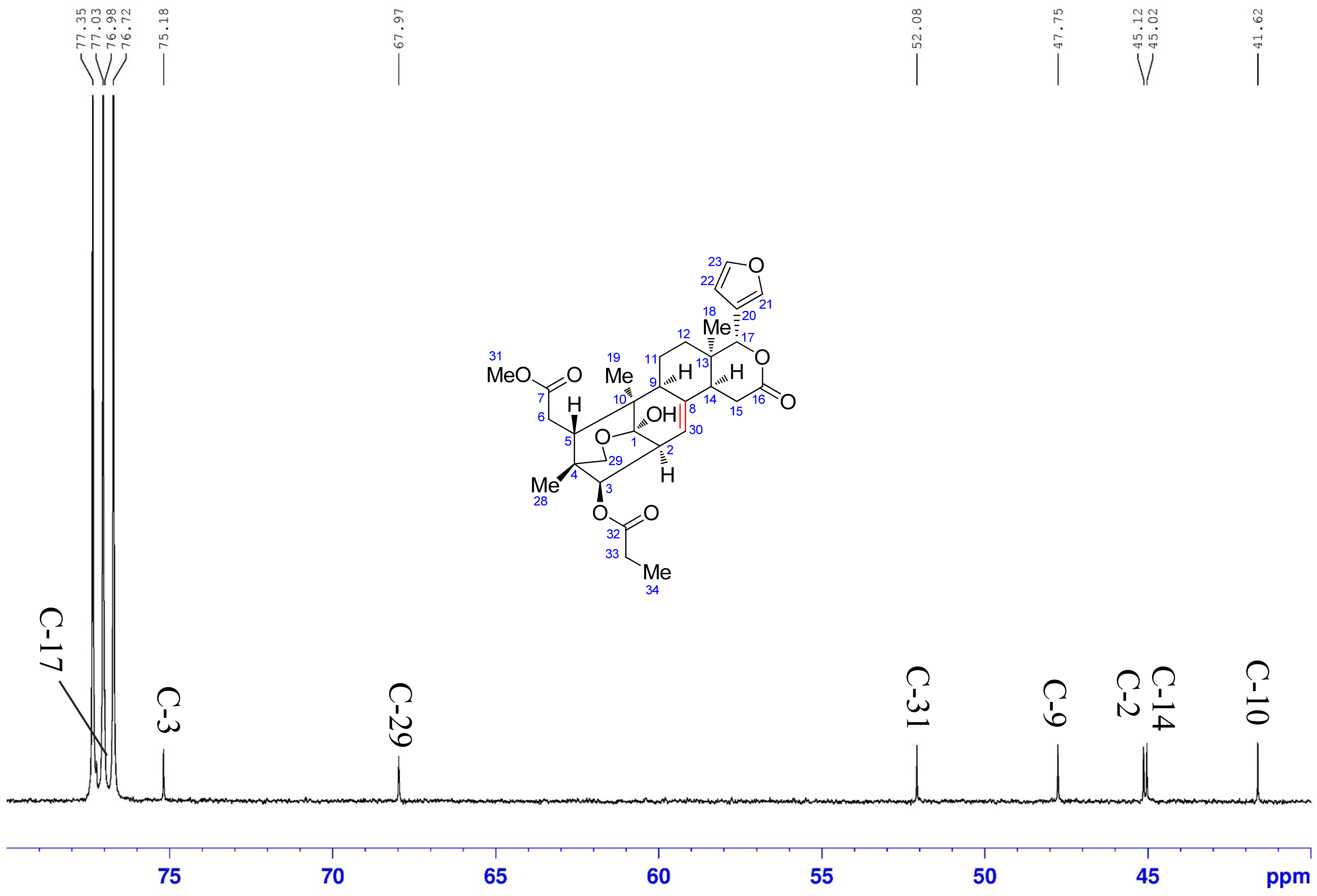

^{13}C NMR (100 MHz) spectrum of compound **2** in $CDCl_3$

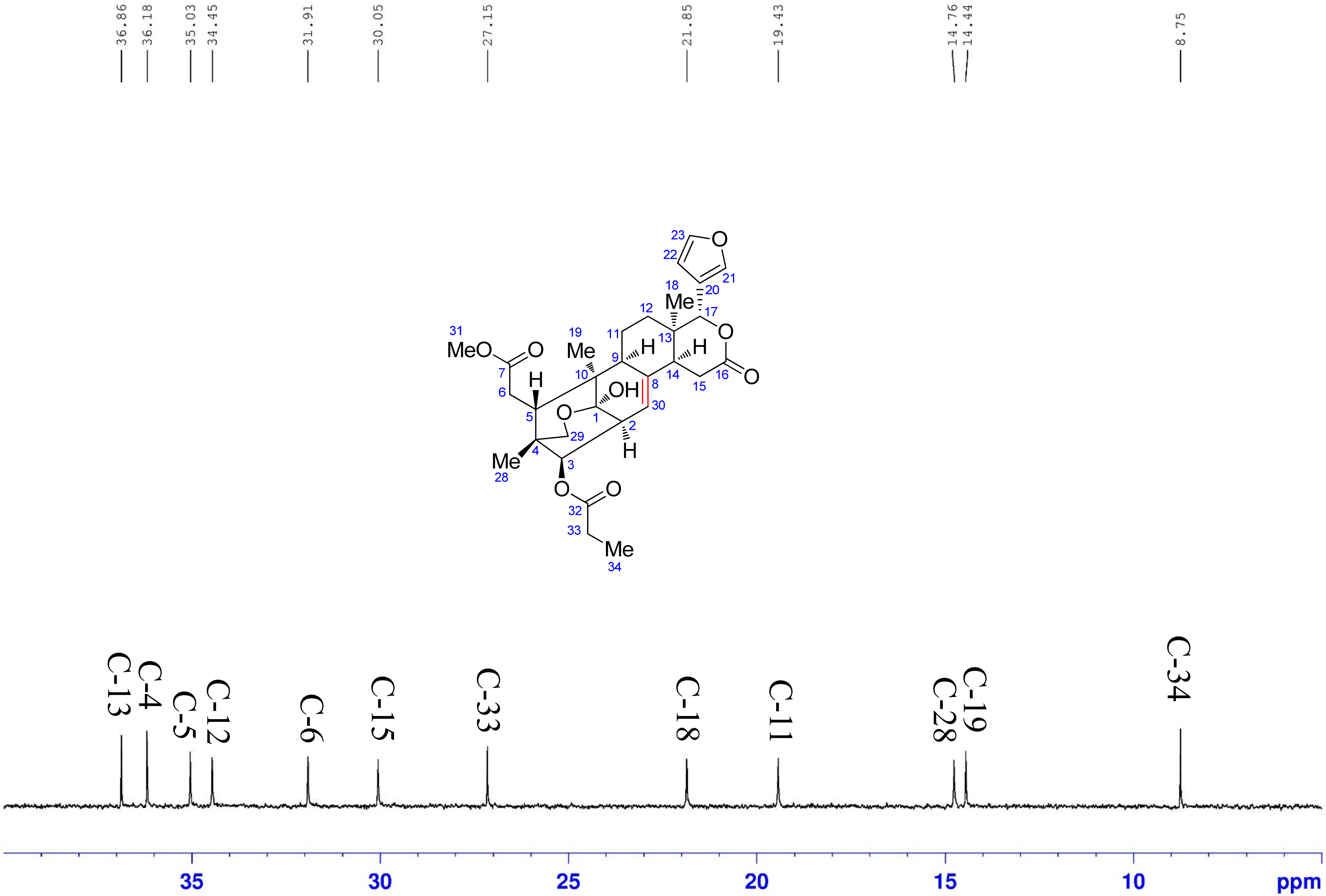

DEPT135 (100 MHz) spectrum of compound **2** in $CDCl_3$

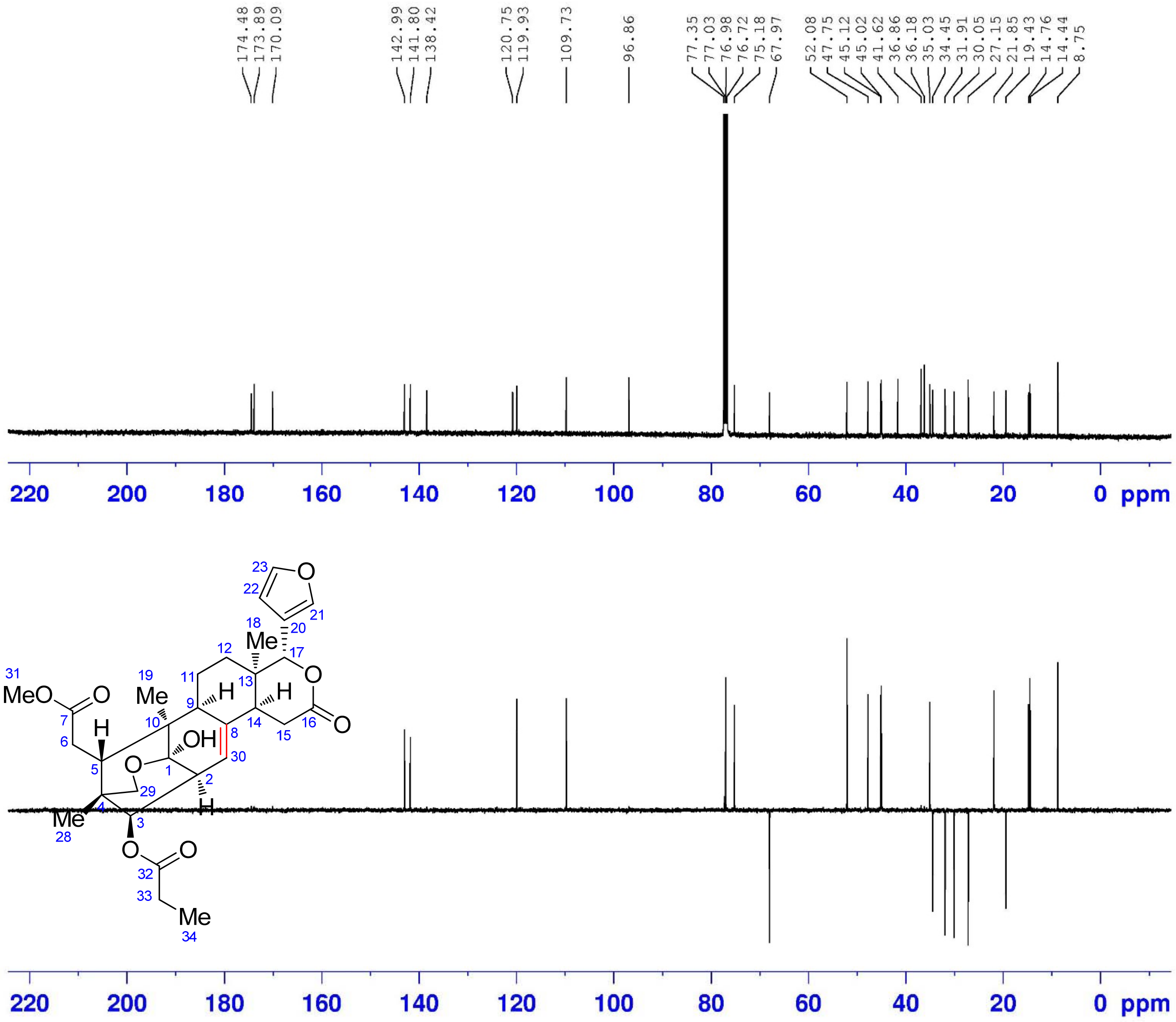

BRUKER

```
NAME       ZXP-R-17-4-2-2-2
EXPNO                     2
PROCNO                    1
Date_              20180818
Time                  14.44
INSTRUM               spect
PROBHD     5 mm CPPBBO BB
PULPROG             zgpg30
TD                    65536
SOLVENT               CDCl3
NS                     1024
DS                        4
SWH               24038.461 Hz
FIDRES             0.366798 Hz
AQ                1.3631988 sec
RG                   130.26
DW                   20.800 usec
DE                    18.00 usec
TE                    297.0 K
D1               2.00000000 sec
D11              0.03000000 sec
TD0                       1

======== CHANNEL f1 ========
SFO1            100.6233324 MHz
NUC1                    13C
P1                    10.00 usec
SI                    32768
SF              100.6127692 MHz
WDW                      EM
SSB                       0
LB                     1.00 Hz
GB                        0
PC                     1.40
```

DEPT135 (100 MHz) spectrum of compound **2** in $CDCl_3$

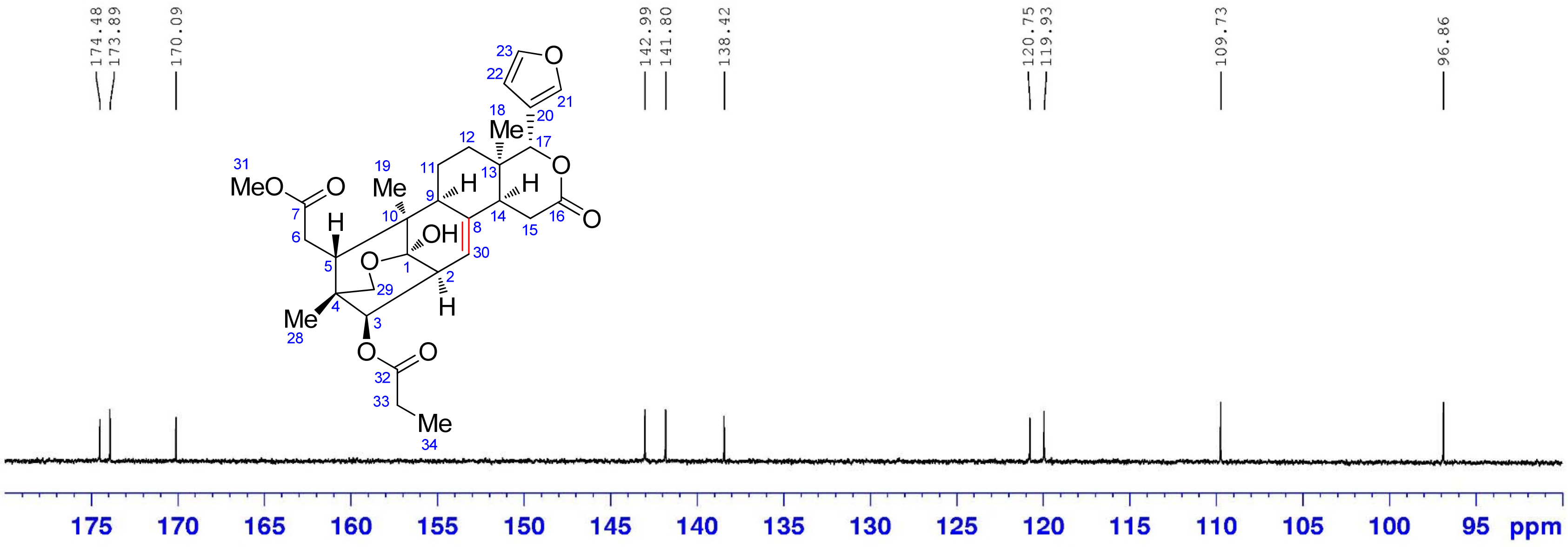

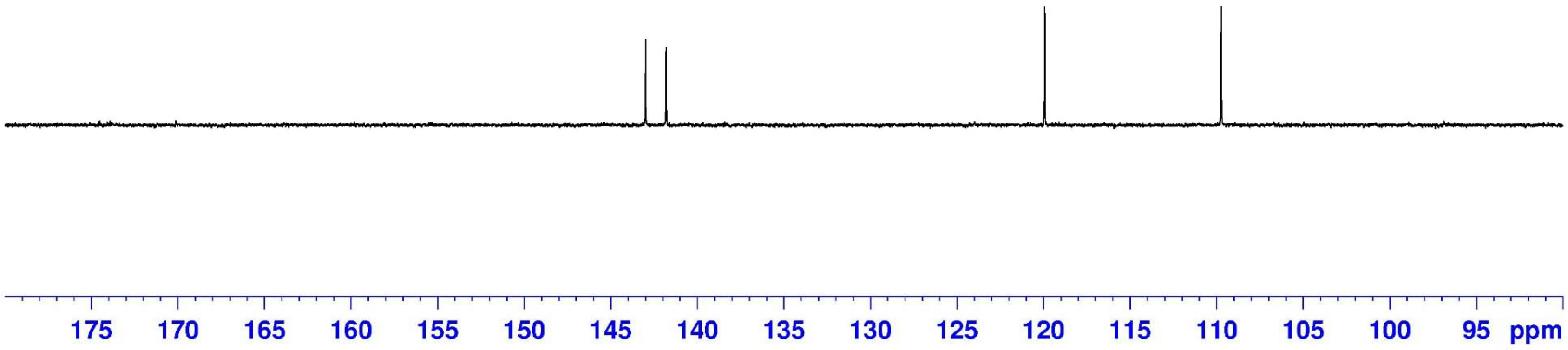

DEPT135 (100 MHz) spectrum of compound **2** in $CDCl_3$

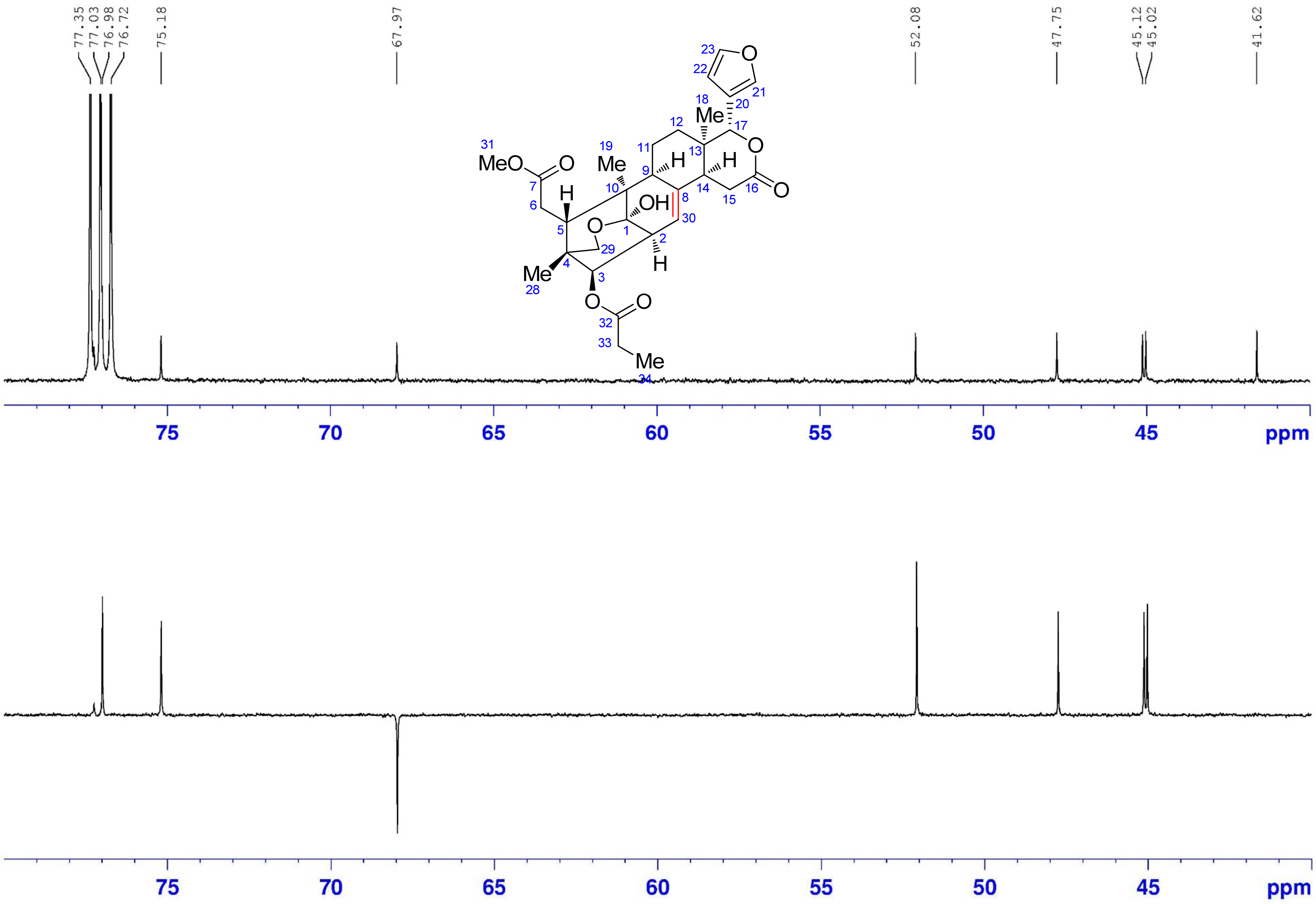

DEPT135 (100 MHz) spectrum of compound **2** in $CDCl_3$

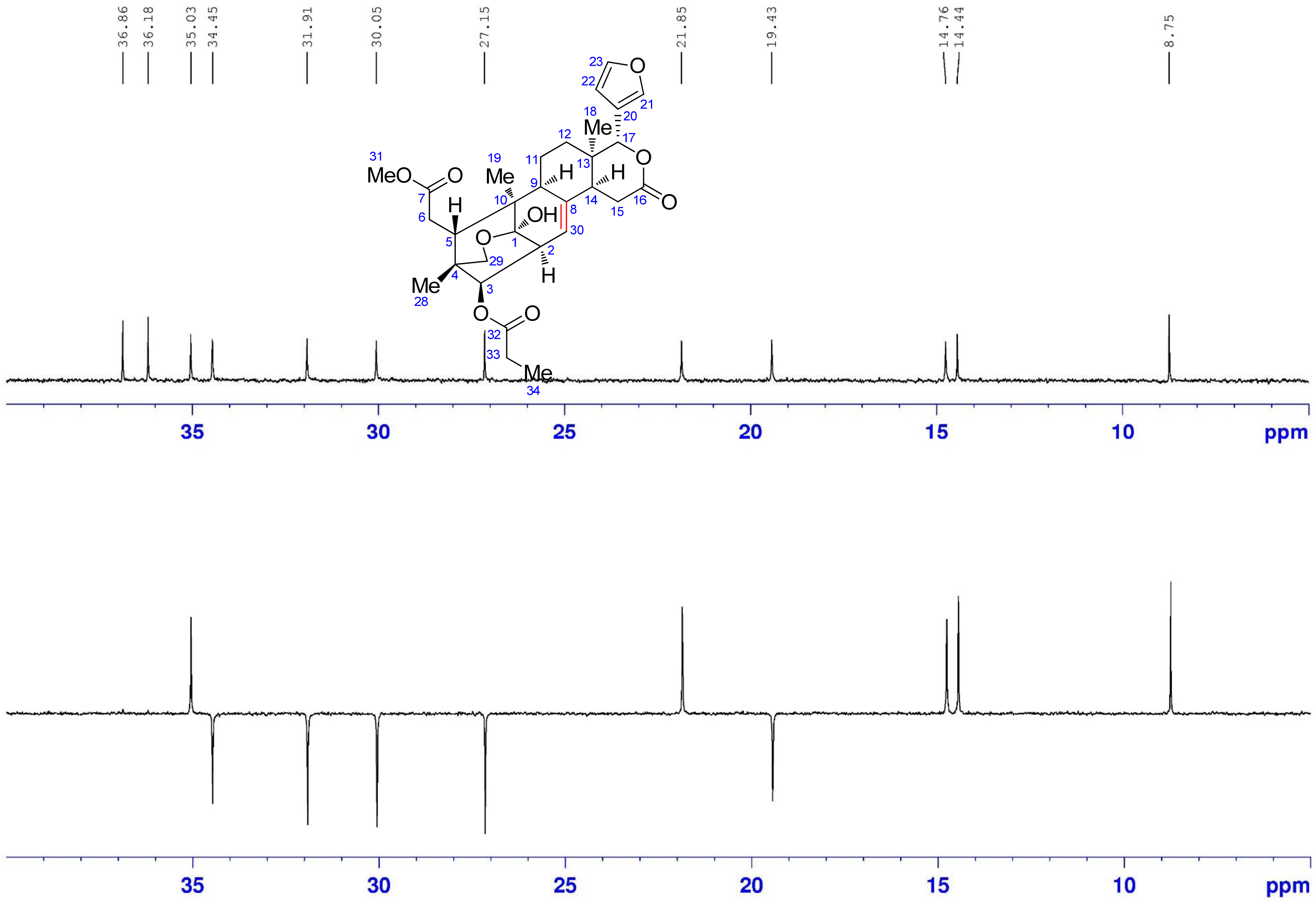

^{1}H-^{1}H COSY (400 MHz) spectrum of compound **2** in $CDCl_3$

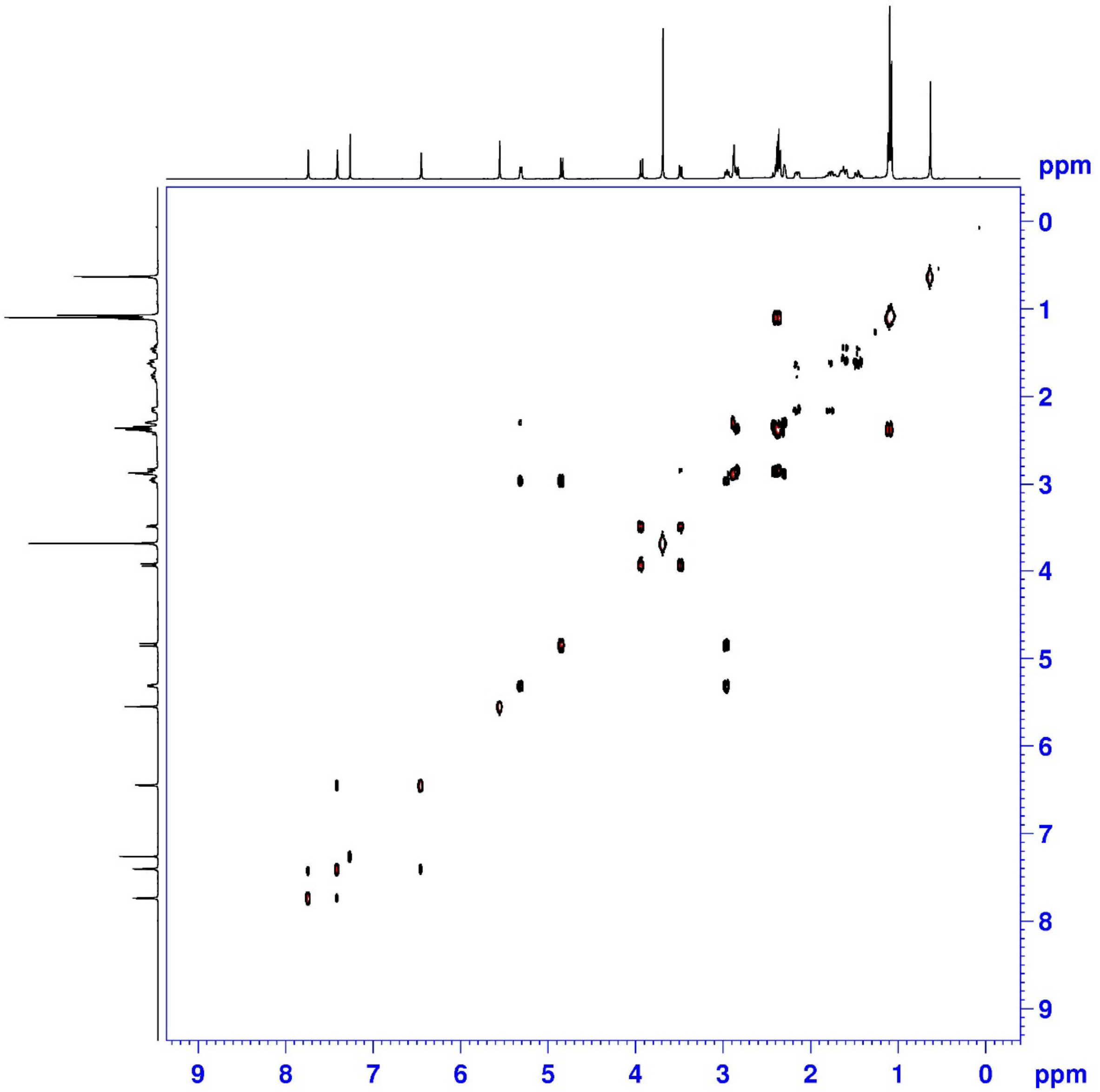


```
NAME       ZXP-R-17-4-2-2-2
EXPNO                     4
PROCNO                    1
Date_              20180821
Time                   4.42
INSTRUM               spect
PROBHD    5 mm CPPBBO BB
PULPROG          cosygpppqf
TD                     2048
SOLVENT               CDCl3
NS                        8
DS                        8
SWH                3906.250 Hz
FIDRES             1.907349 Hz
AQ                0.2621940 sec
RG                   147.94
DW                  128.000 usec
DE                    10.00 usec
TE                    297.0 K
D0               0.00000300 sec
D1               1.89678097 sec
D11              0.03000000 sec
D12              0.00002000 sec
D13              0.00000400 sec
D16              0.00020000 sec
IN0              0.00025600 sec

======== CHANNEL f1 ========
SFO1            400.1318006 MHz
NUC1                     1H
P0                    11.50 usec
P1                    11.50 usec
P17                 2500.00 usec
ND0                       1
TD                      128
SFO1               400.1318 MHz
FIDRES            30.517578 Hz
SW                    9.762 ppm
FnMODE                   QF
SI                     1024
SF              400.1300076 MHz
WDW                   QSINE
SSB                       0
LB                     0.00 Hz
GB                        0
PC                     1.40
SI                     1024
MC2                      QF
SF              400.1300060 MHz
WDW                   QSINE
SSB                       0
LB                     0.00 Hz
GB                        0
```

^{1}H-^{1}H COSY (400 MHz) spectrum of compound **2** in $CDCl_3$

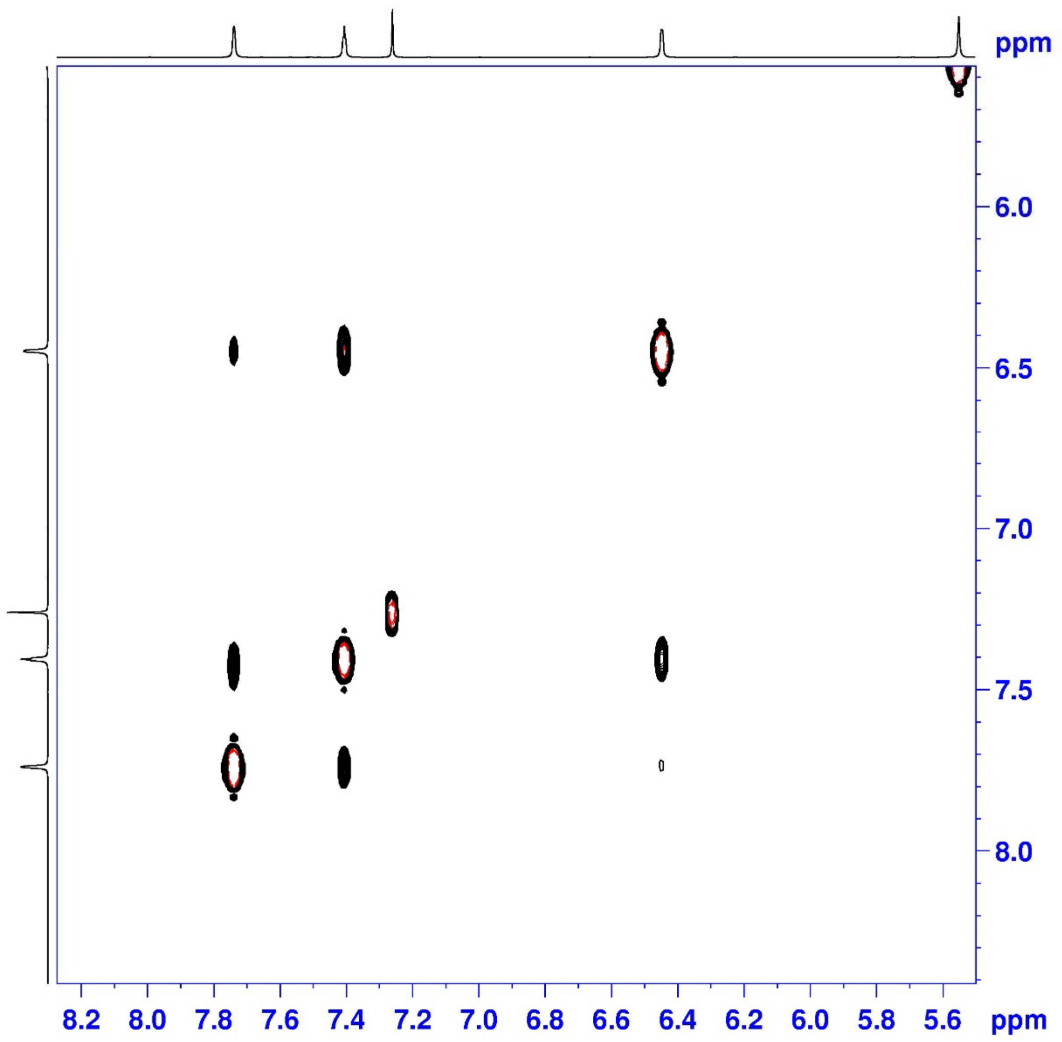

^{1}H-^{1}H COSY (400 MHz) spectrum of compound **2** in $CDCl_3$

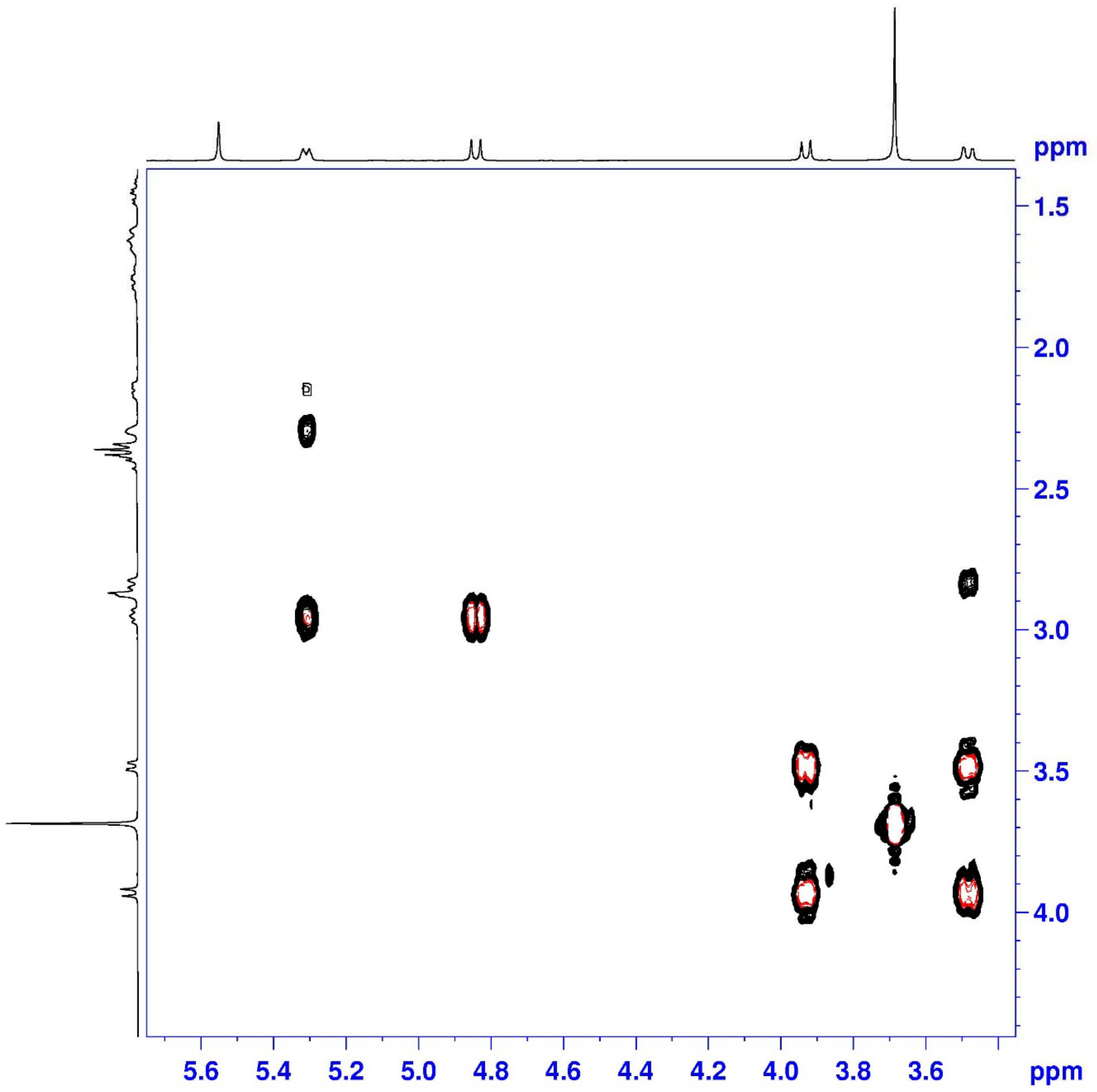

^{1}H-^{1}H COSY (400 MHz) spectrum of compound **2** in $CDCl_3$

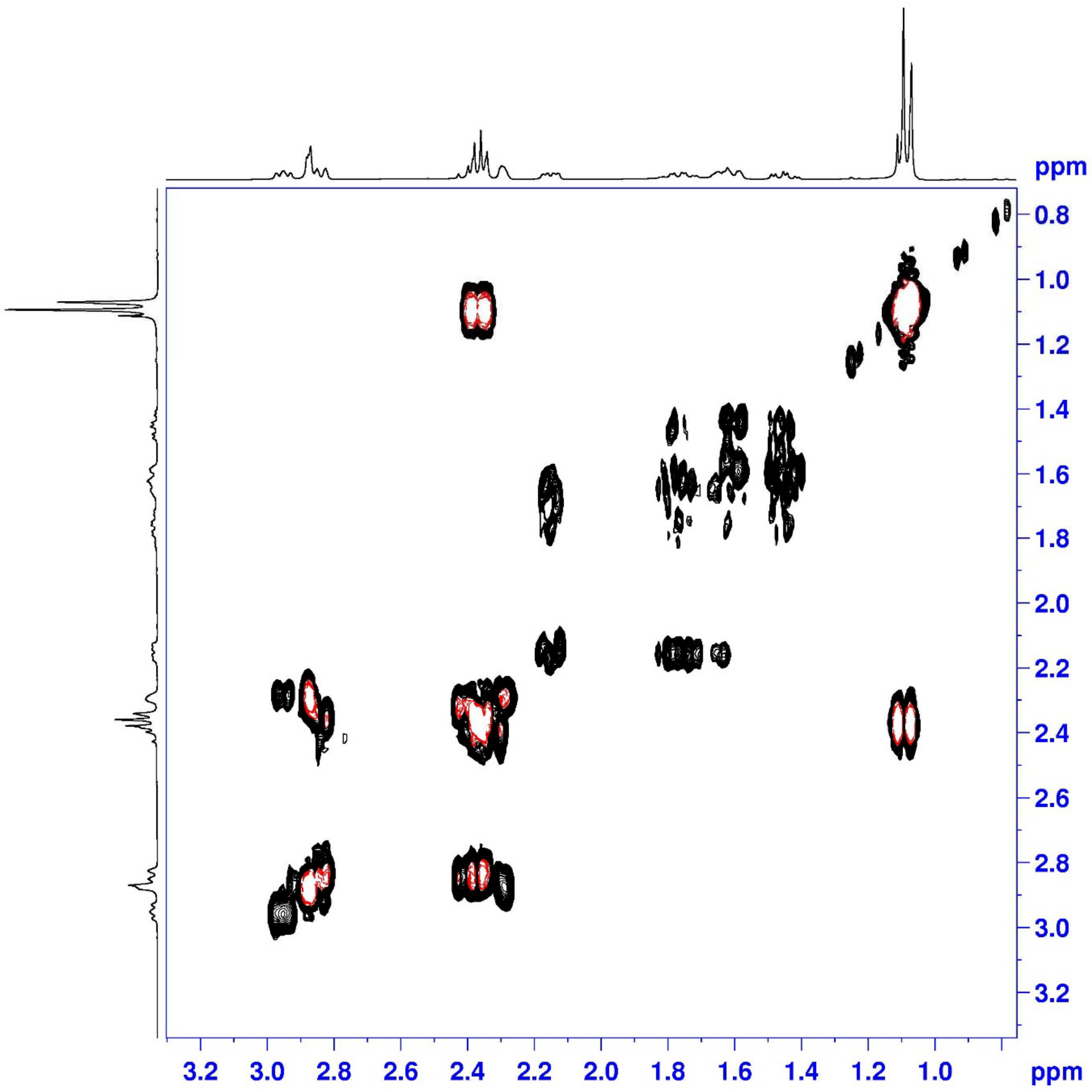

HSQC (400 MHz) spectrum of compound **2** in $CDCl_3$

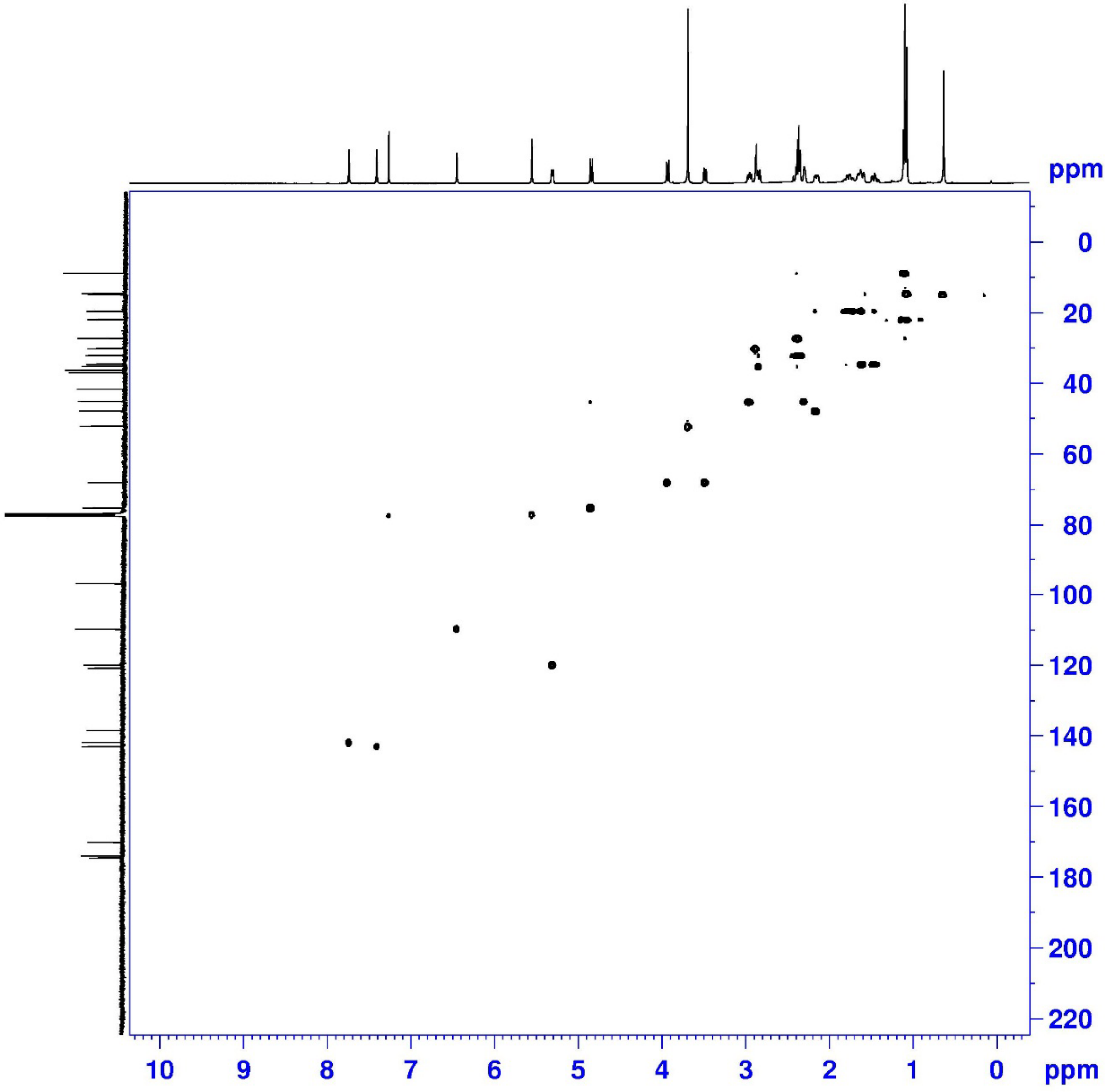


```
NAME       ZXP-R-17-4-2-2-2
EXPNO                     5
PROCNO                    1
Date_              20180821
Time                   5.21
INSTRUM               spect
PROBHD    5 mm CPPBBO BB
PULPROG          hsqcetgpsi2
TD                     1024
SOLVENT               CDCl3
NS                       16
DS                       16
SWH               4302.926 Hz
FIDRES            4.202076 Hz
AQ               0.1190388 sec
RG                    208.5
DW                  116.200 usec
DE                    10.00 usec
TE                    297.0 K
CNST2          145.0000000
D0              0.00000300 sec
D1              1.46497905 sec
D4              0.00172414 sec
D11             0.03000000 sec
D16             0.00020000 sec
D24             0.00086207 sec
IN0             0.00002080 sec
ZGOPTNS

======== CHANNEL f1 ========
SFO1           400.1320007 MHz
NUC1                    1H
P1                   11.50 usec
P2                   23.00 usec
P28                   0.00 usec
ND0                      2
TD                     256
SFO1             100.6233 MHz
FIDRES          93.900238 Hz
SW                238.896 ppm
FnMODE        Echo-Antiecho
SI                    1024
SF             400.1300074 MHz
WDW                  QSINE
SSB                      2
LB                    0.00 Hz
GB                       0
PC                    1.40
SI                    1024
MC2           echo-antiecho
SF             100.6127538 MHz
WDW                  QSINE
SSB                      2
LB                    0.00 Hz
GB                       0
```

HSQC (400 MHz) spectrum of compound **2** in $CDCl_3$

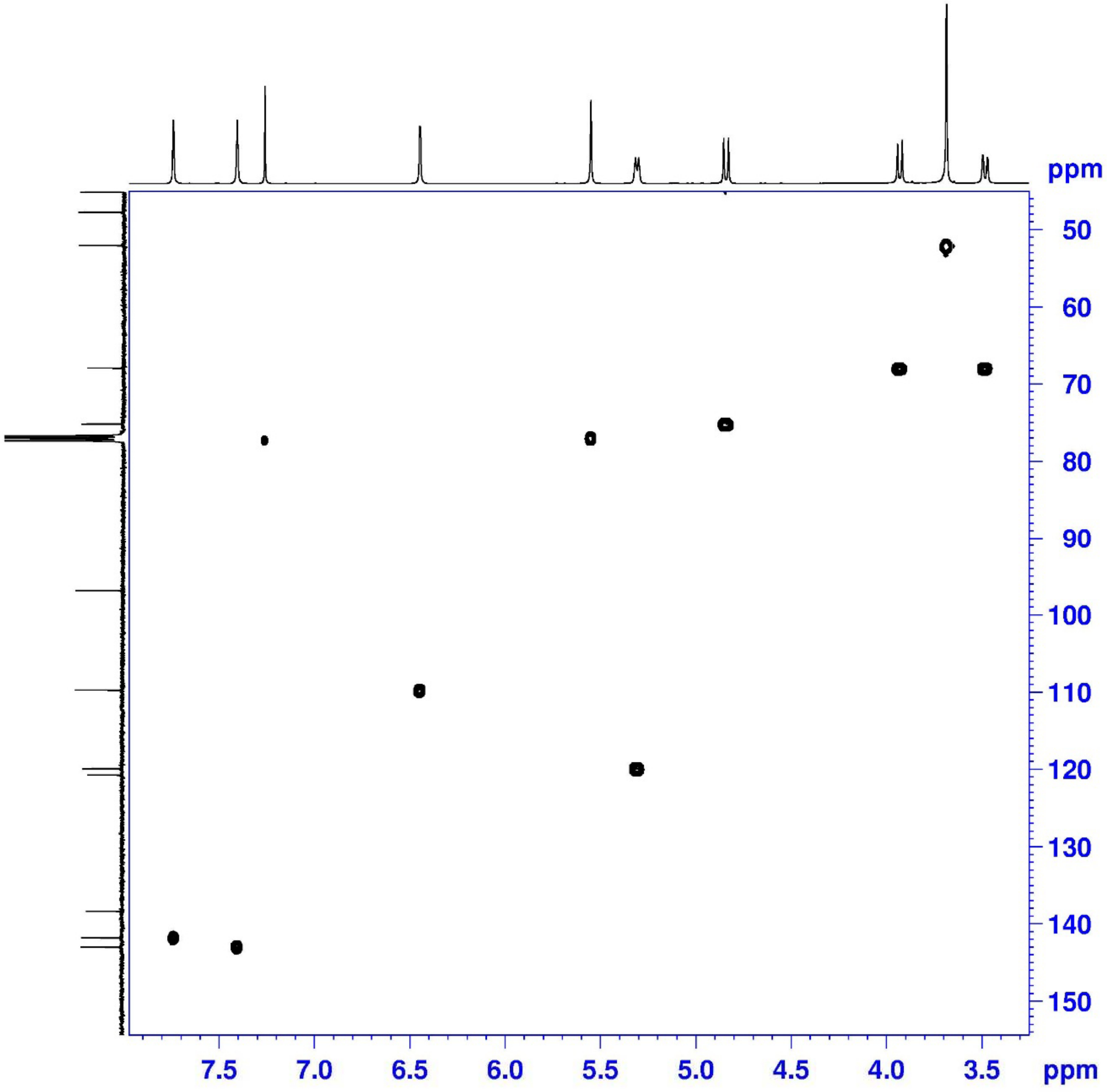

HSQC (400 MHz) spectrum of compound **2** in $CDCl_3$

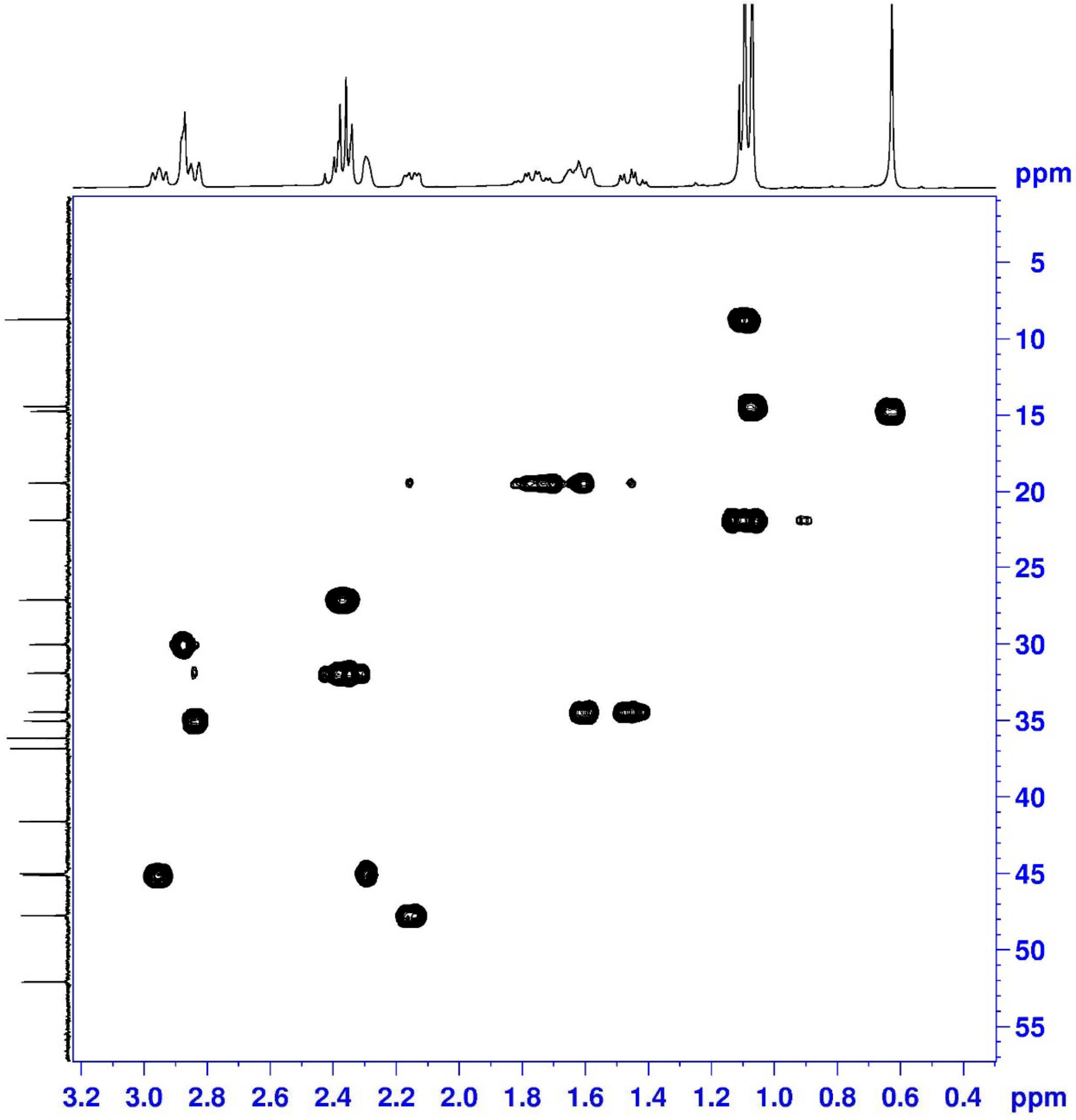

HMBC (400 MHz) spectrum of compound **2** in $CDCl_3$

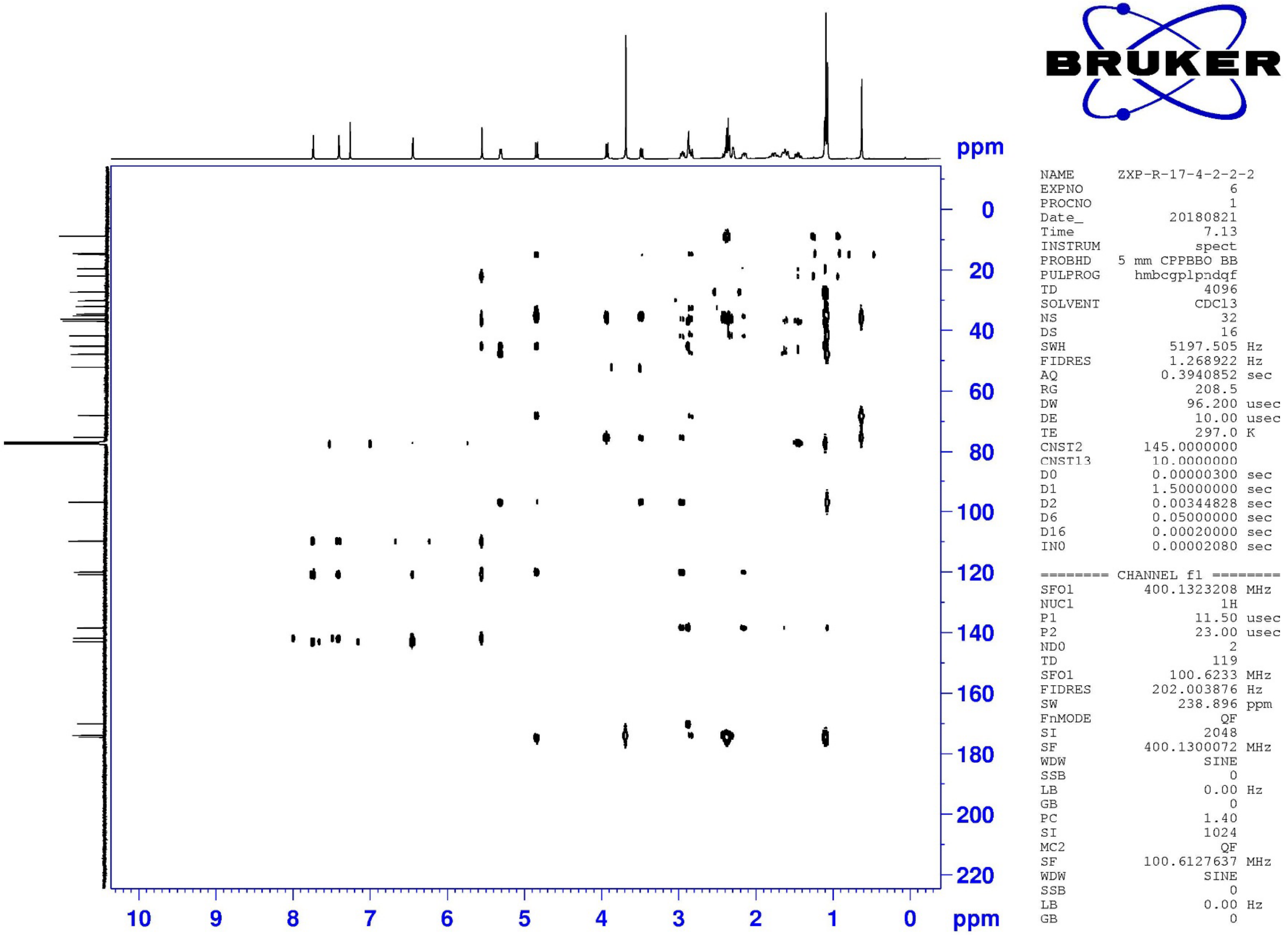

HMBC (400 MHz) spectrum of compound **2** in $CDCl_3$

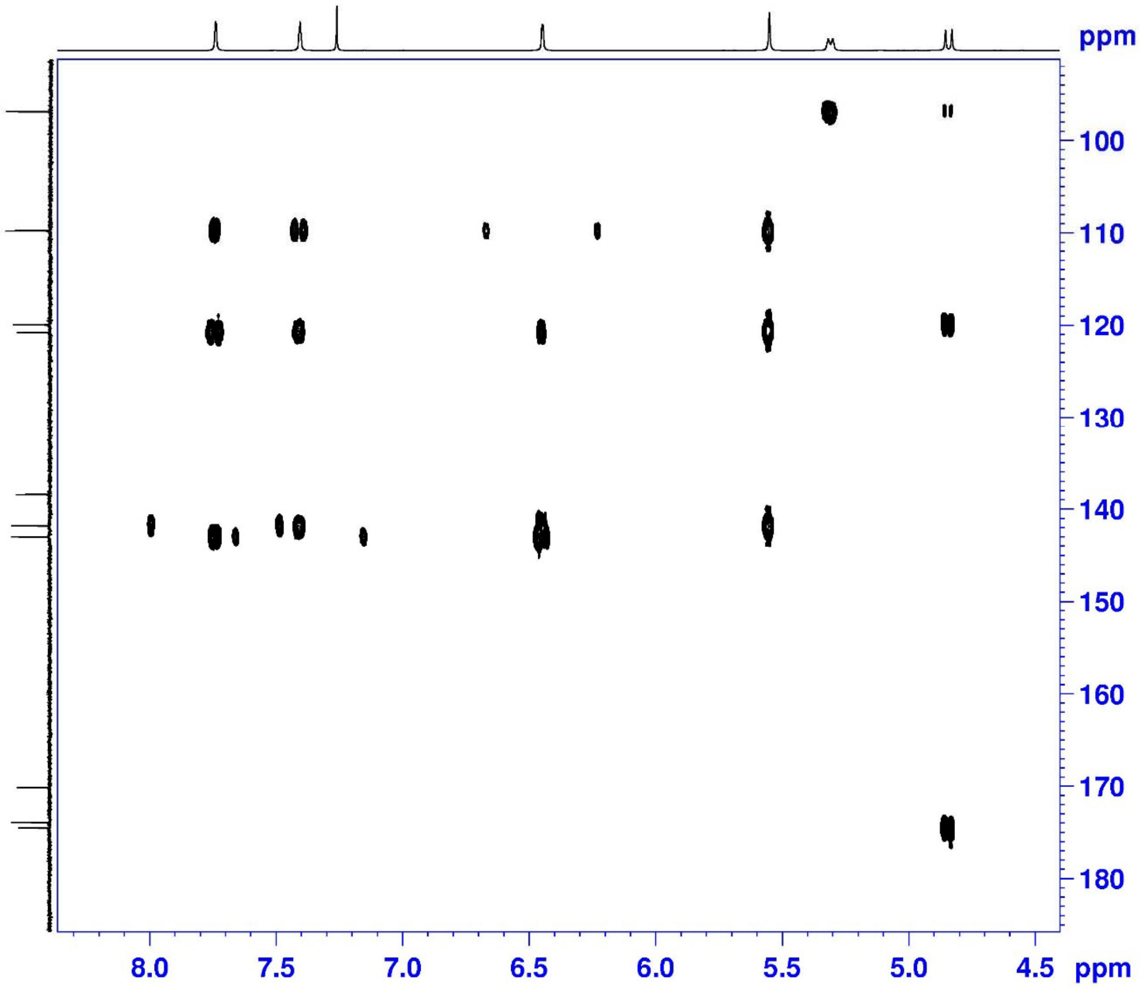

HMBC (400 MHz) spectrum of compound **2** in $CDCl_3$

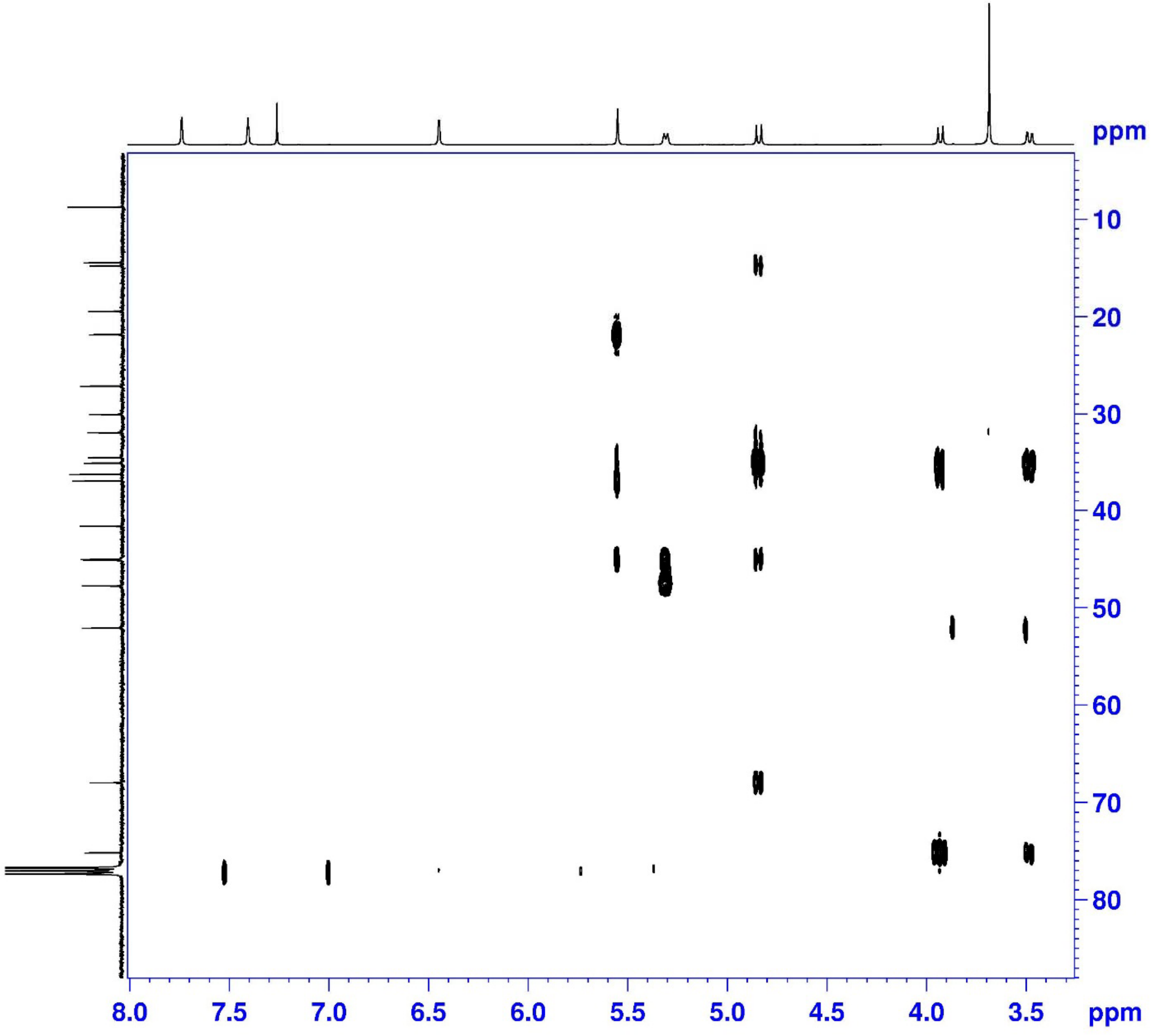

HMBC (400 MHz) spectrum of compound **2** in $CDCl_3$

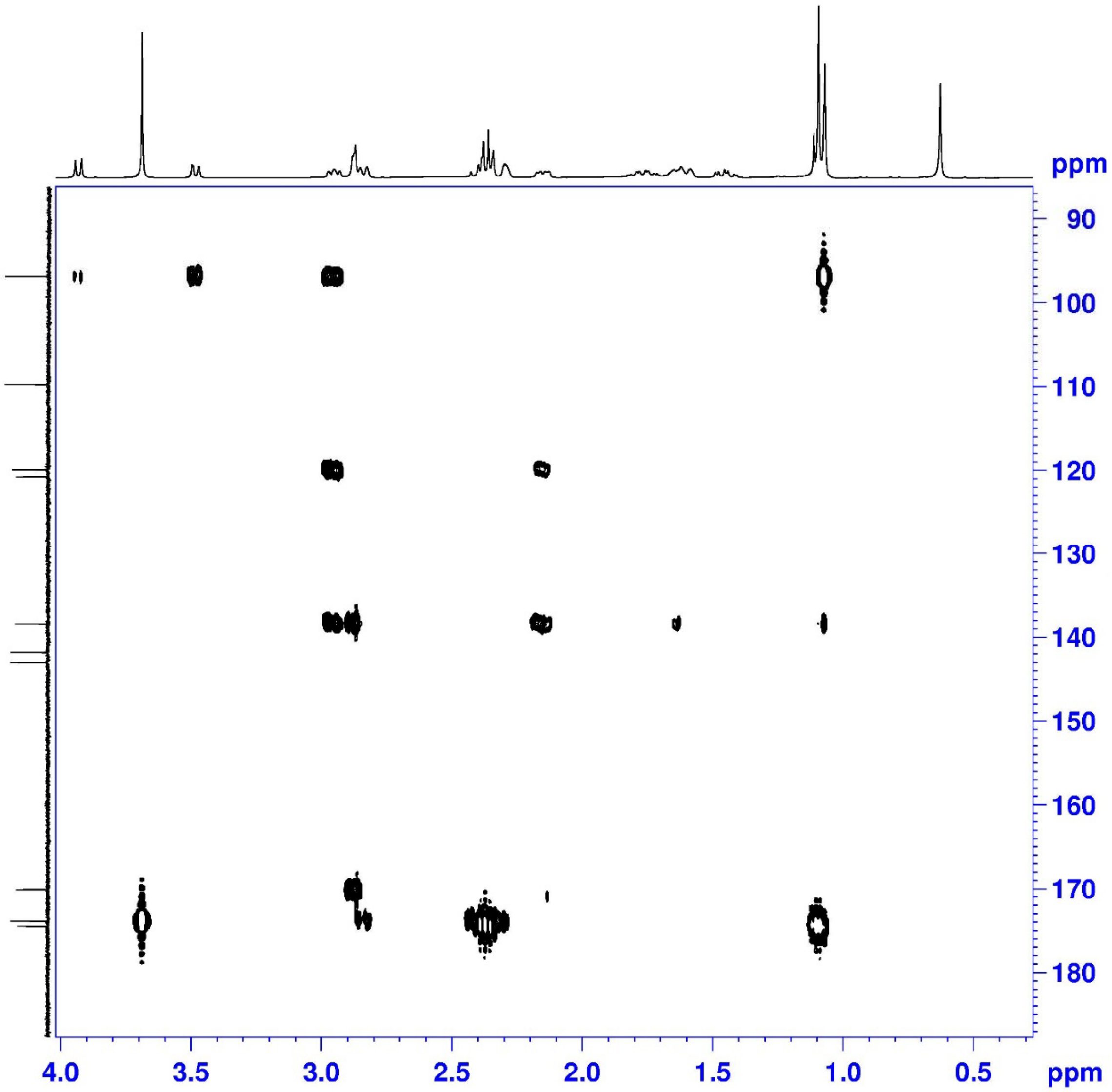

HMBC (400 MHz) spectrum of compound **2** in $CDCl_3$

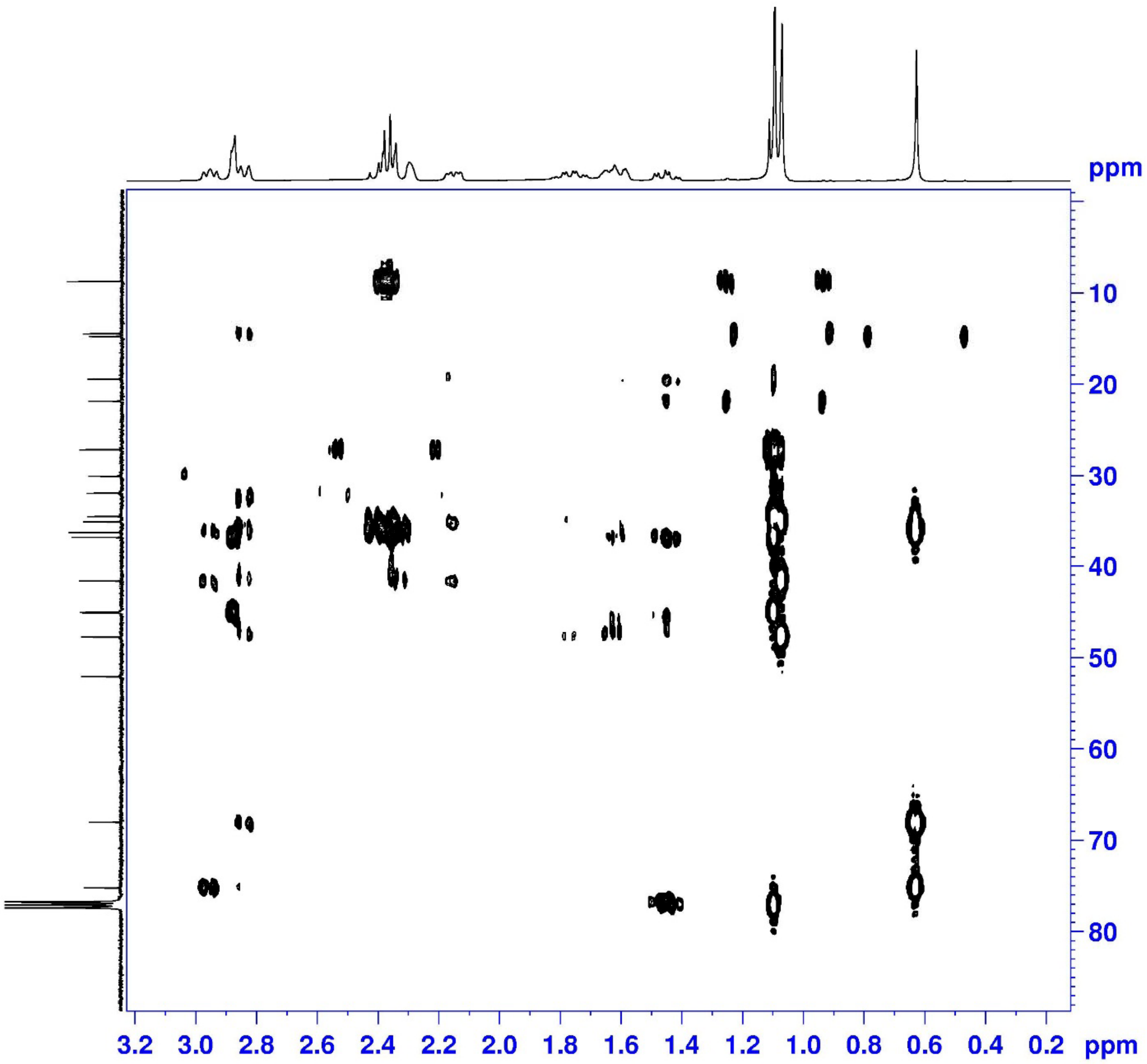

HMBC (400 MHz) spectrum of compound **2** in $CDCl_3$

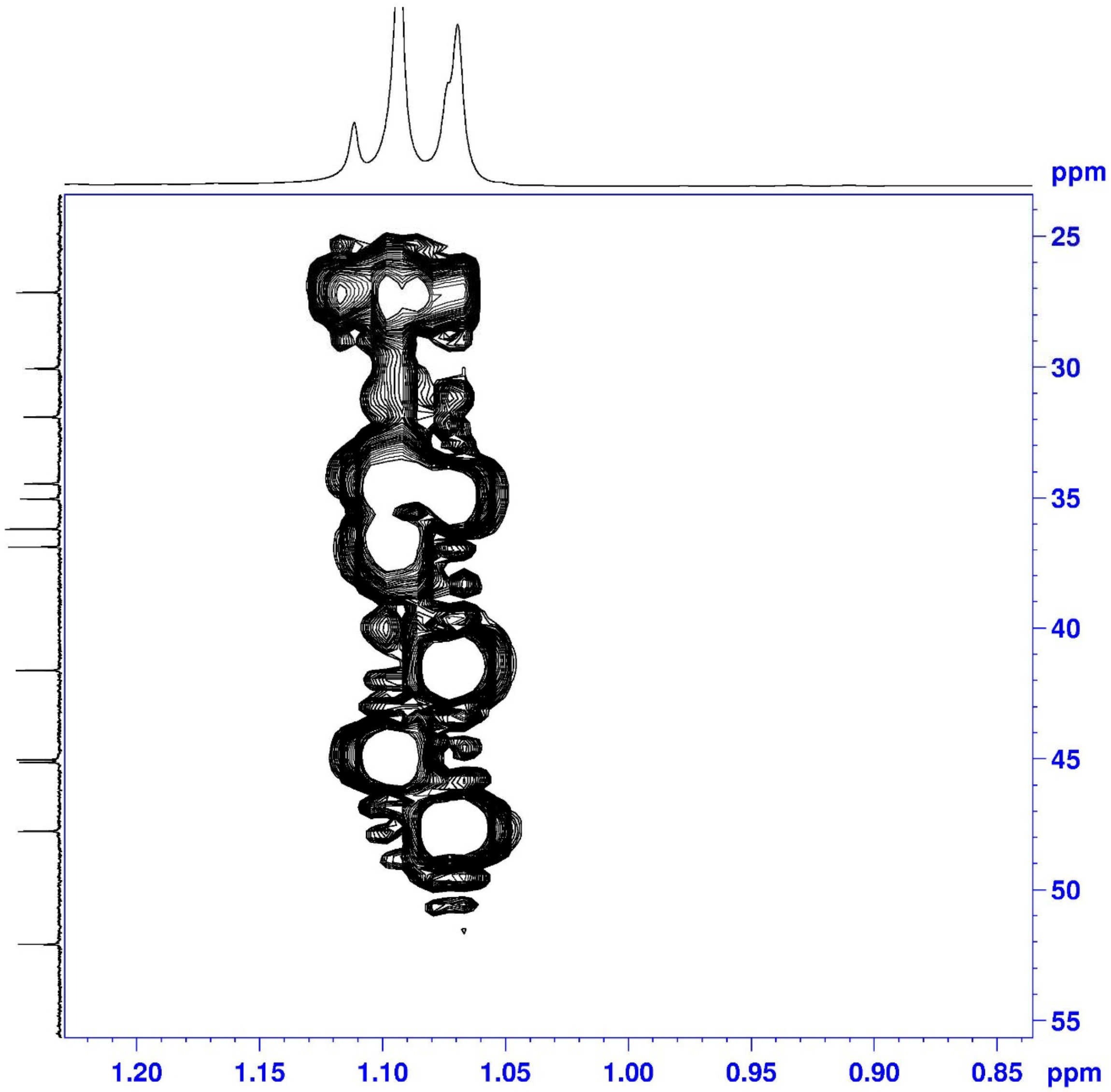

NOESY (400 MHz) spectrum of compound **2** in $CDCl_3$

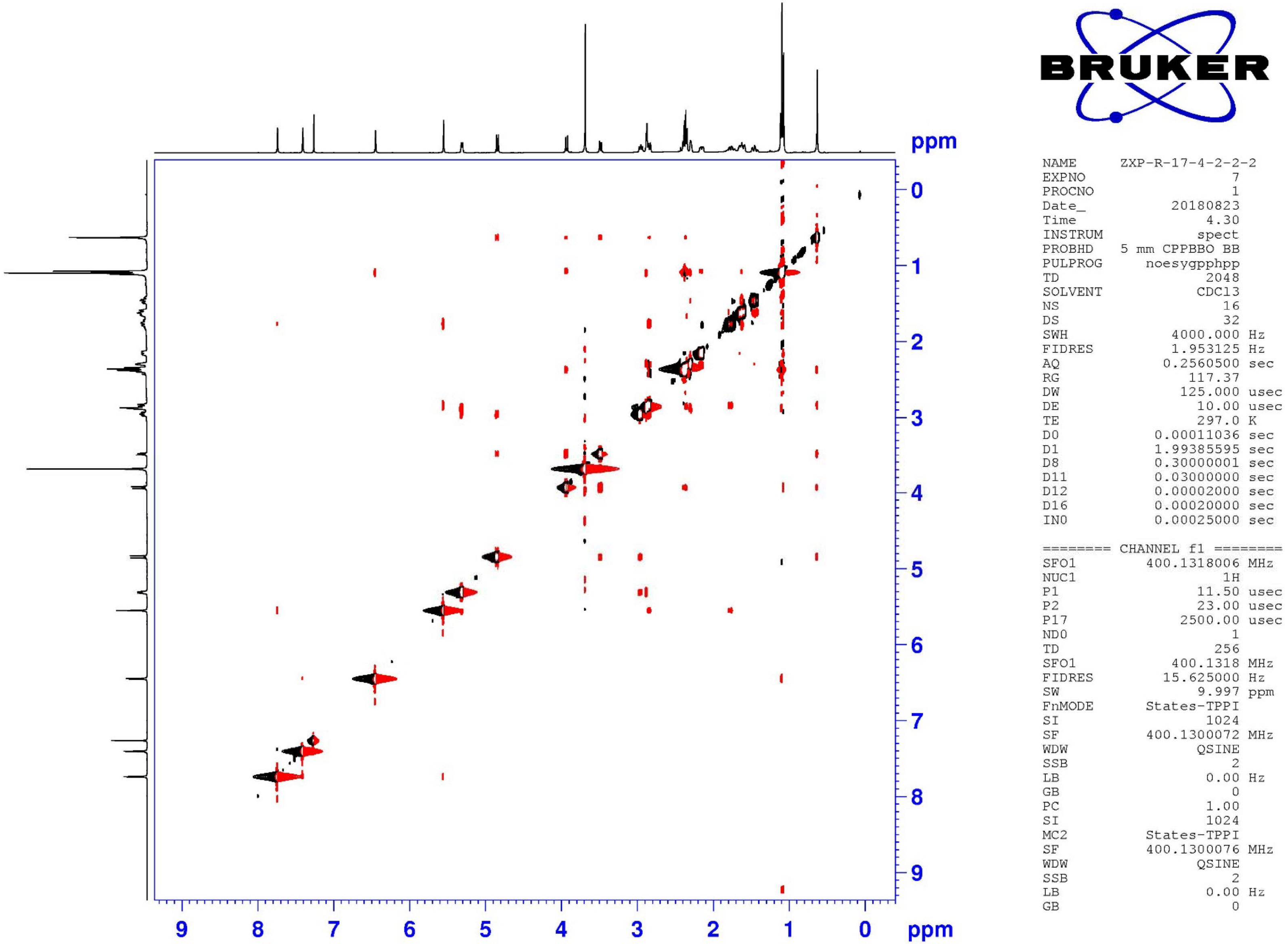

NOESY (400 MHz) spectrum of compound **2** in $CDCl_3$

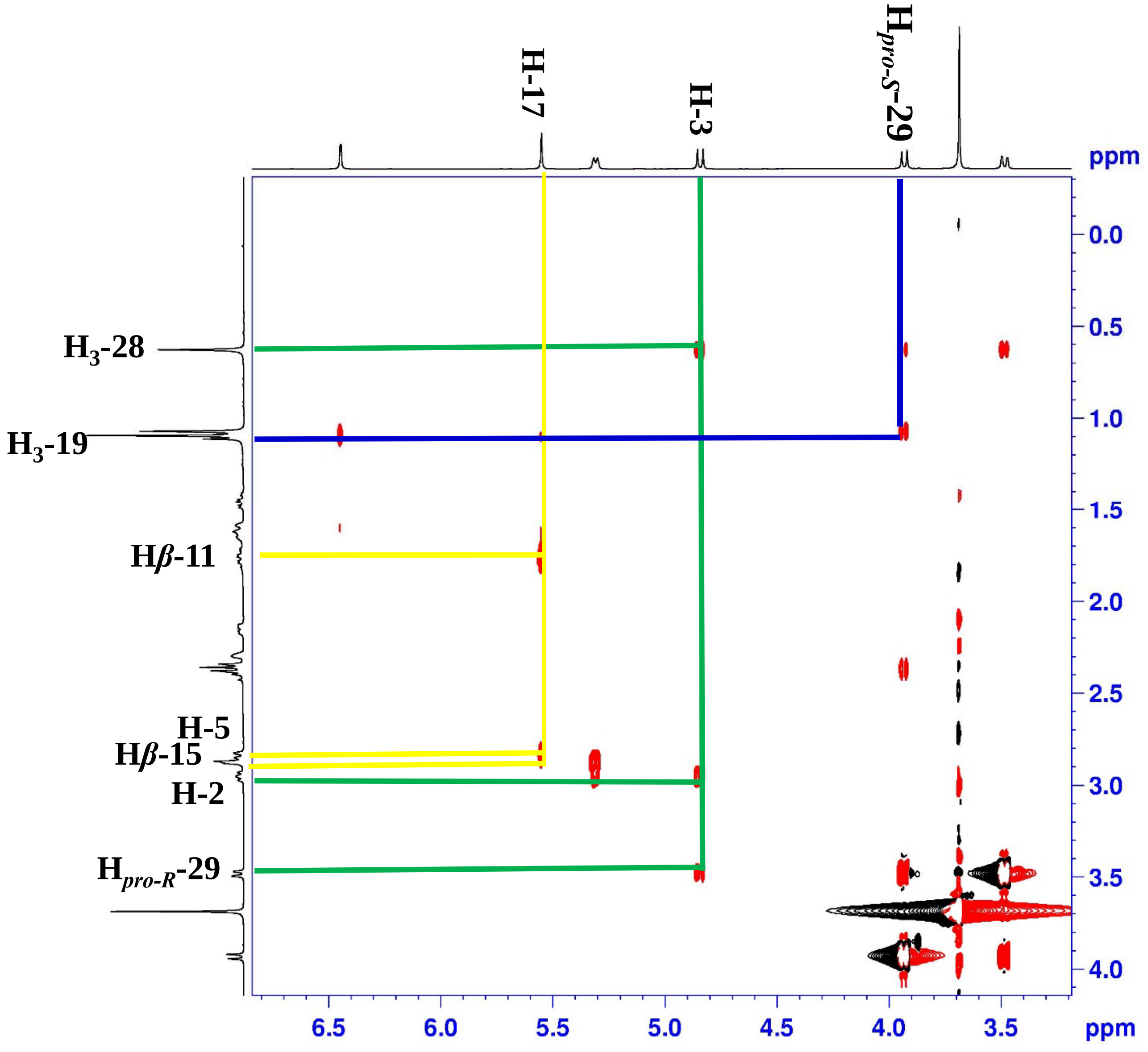

NOESY (400 MHz) spectrum of compound **2** in $CDCl_3$

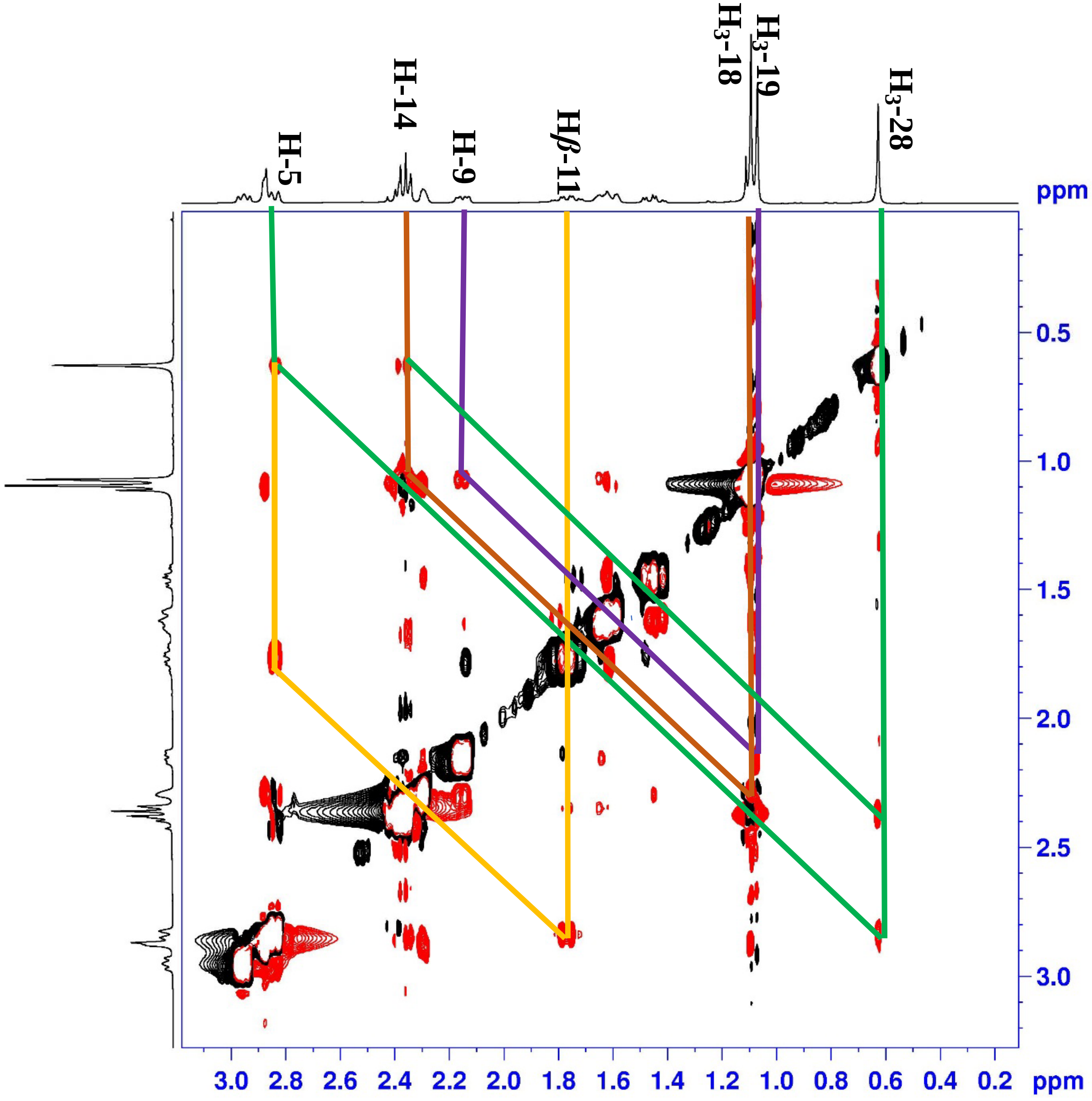

HR-ESIMS for compound **3**

Generic Display Report

Analysis Info

Analysis Name	D:\Data\MS\data\201805\liwanshan_P-18-3-1_pos_40_01_4670.d	Acquisition Date	5/4/2018 11:54:44 AM
Method	LC_Direct Infusion_pos_100-1000mz.m	Operator	SCSIO
Sample Name	liwanshan_P-18-3-1_pos	Instrument	maXis
Comment			

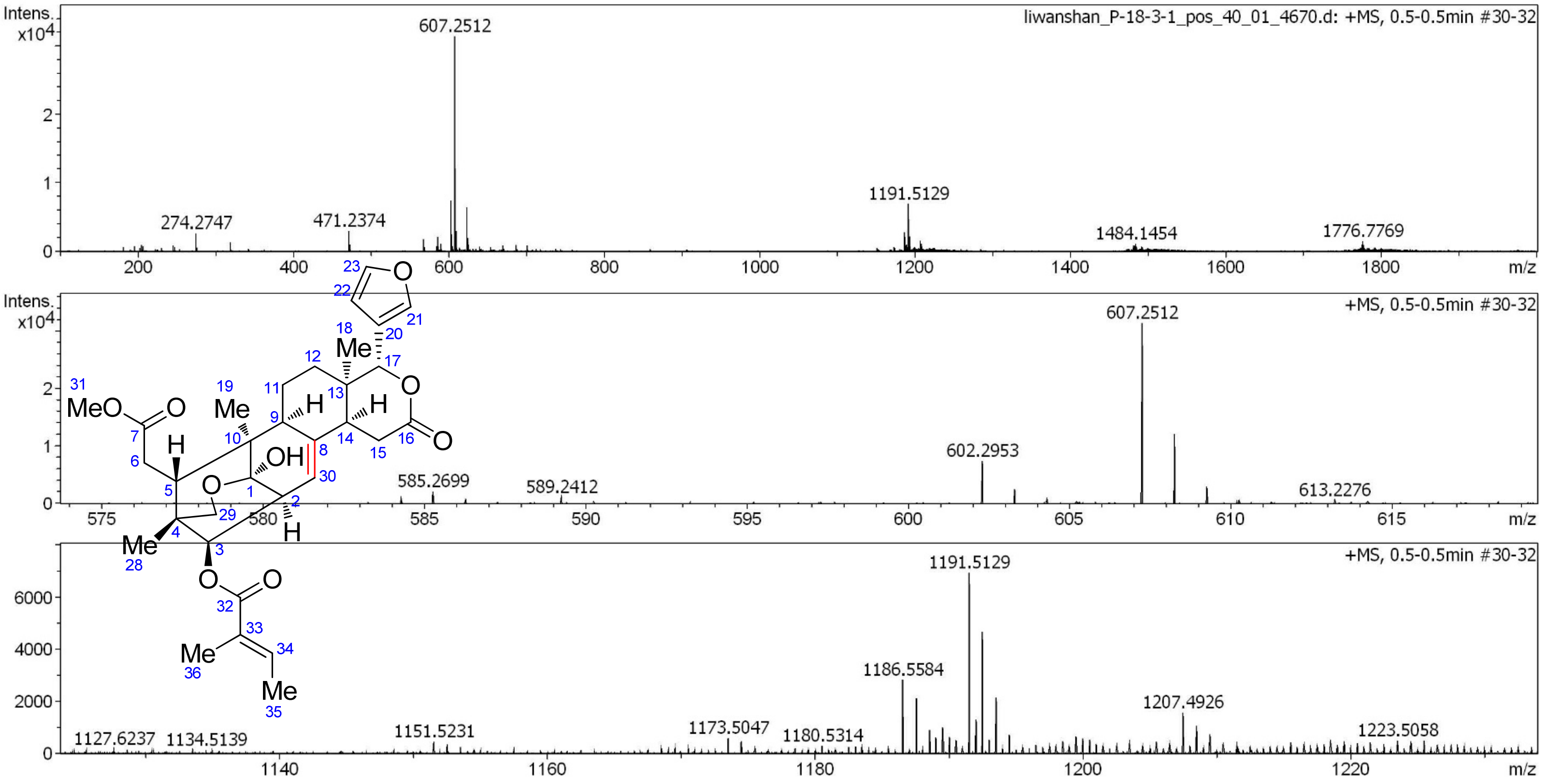

HR-ESIMS for compound **3**

Mass Spectrum SmartFormula Report

Analysis Info

Analysis Name	D:\Data\MS\data\201805\liwanshan_P-18-3-1_pos_40_01_4670.d	Acquisition Date	5/4/2018 11:54:44 AM
Method	LC_Direct Infusion_pos_100-1000mz.m	Operator	SCSIO
Sample Name	liwanshan_P-18-3-1_pos	Instrument	maXis 255552.00029
Comment			

Acquisition Parameter

Source Type	ESI	Ion Polarity	Positive	Set Nebulizer	0.4 Bar
Focus	Active	Set Capillary	4500 V	Set Dry Heater	180 °C
Scan Begin	100 m/z	Set End Plate Offset	-500 V	Set Dry Gas	4.0 l/min
Scan End	2000 m/z	Set Charging Voltage	0 V	Set Divert Valve	Waste
		Set Corona	0 nA	Set APCI Heater	0 °C

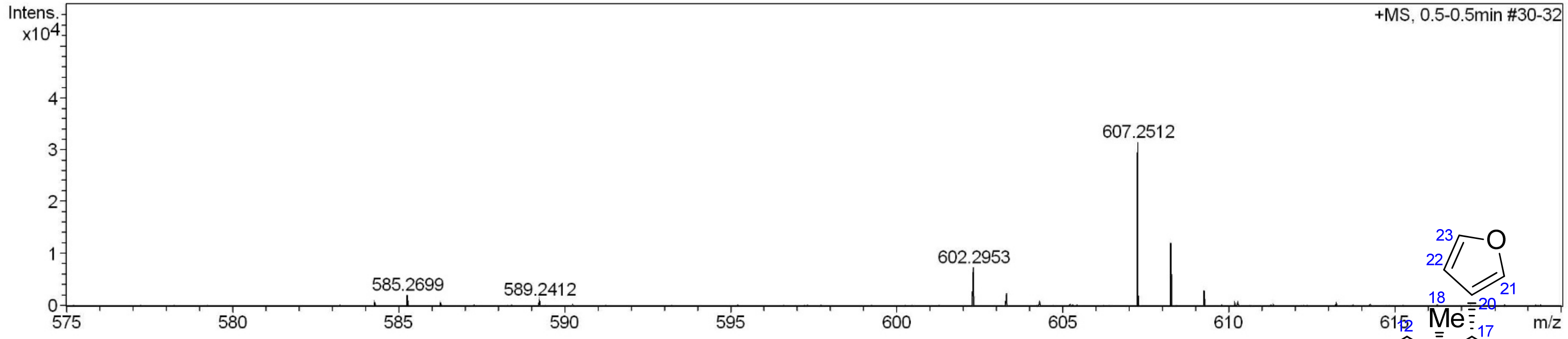

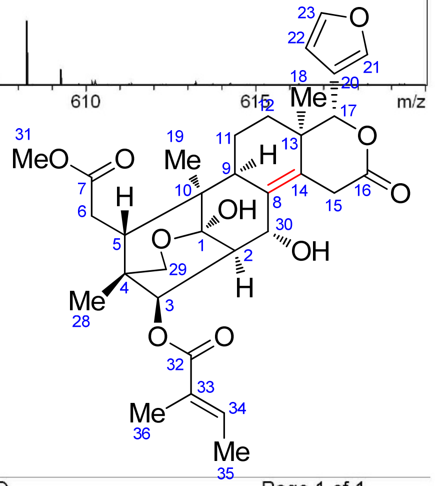

Meas. m/z	#	Ion Formula	Score	m/z	err [ppm]	err [mDa]	mSigma	rdb	e⁻ Conf	N-Rule
585.2699	1	C32H41O10	100.00	585.2694	-0.8	-0.5	30.5	12.5	even	ok
607.2512	1	C32H40NaO10	100.00	607.2514	0.3	0.2	16.6	12.5	even	ok
1191.5129	1	C64H80NaO20	100.00	1191.5135	0.6	0.7	25.9	24.5	even	ok

^{1}H NMR (400 MHz) spectrum of compound **3** in $CDCl_3$

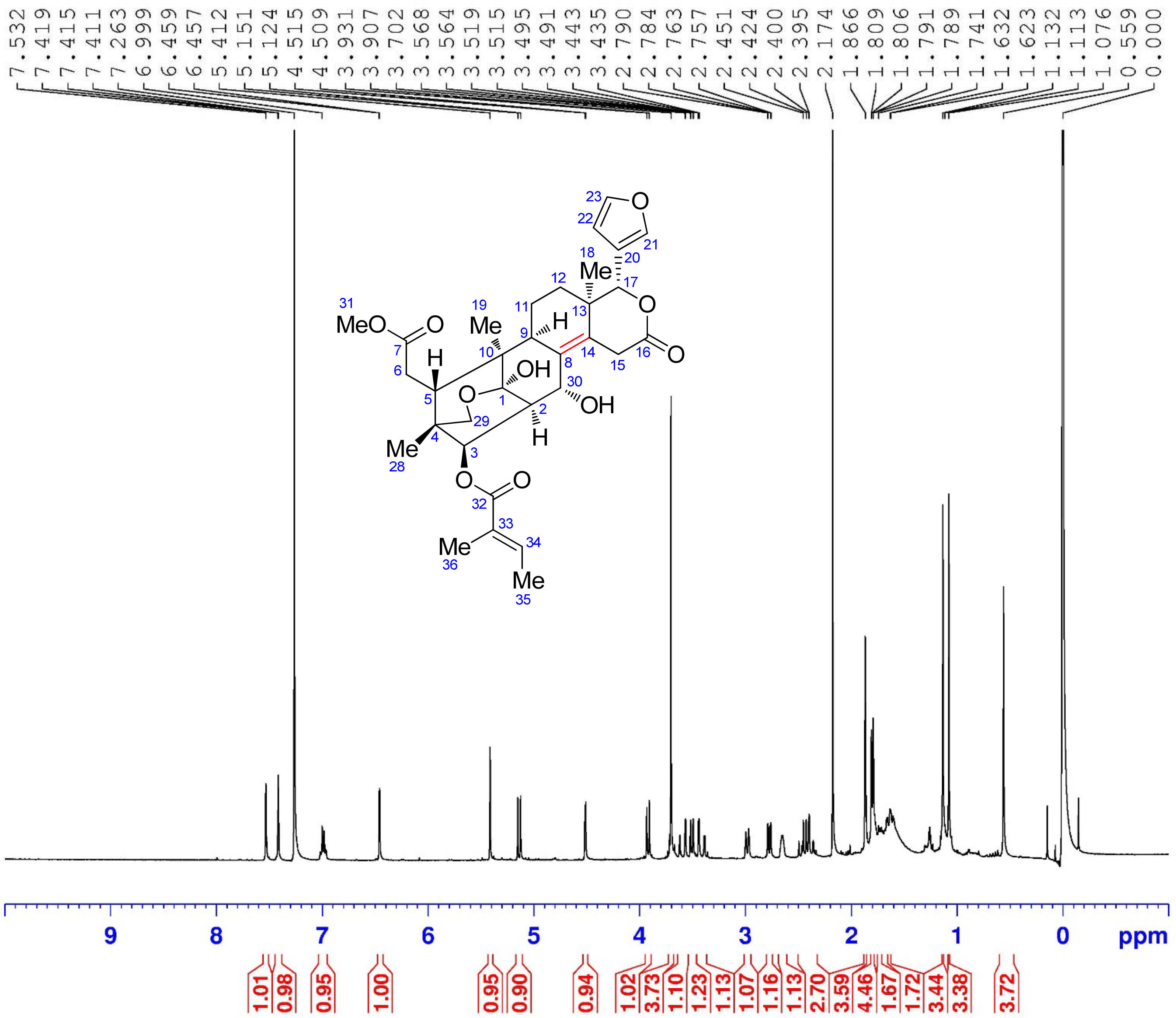


```
NAME       ZXP-R-18-3-1-3-3 new
EXPNO                     1
PROCNO                    1
Date_              20180109
Time                   8.01
INSTRUM               spect
PROBHD     5 mm CPPBBO BB
PULPROG                zg30
TD                    65536
SOLVENT               CDCl3
NS                       16
DS                        2
SWH                8223.685 Hz
FIDRES             0.125483 Hz
AQ                3.9846387 sec
RG                    208.5
DW                   60.800 usec
DE                    10.00 usec
TE                    297.0 K
D1               1.00000000 sec
TD0                       1

======== CHANNEL f1 ========
SFO1            400.1324710 MHz
NUC1                     1H
P1                    11.50 usec
SI                    65536
SF              400.1300087 MHz
WDW                      EM
SSB                       0
LB                     0.30 Hz
GB                        0
PC                     1.00
```

^{1}H NMR (400 MHz) spectrum of compound **3** in $CDCl_3$

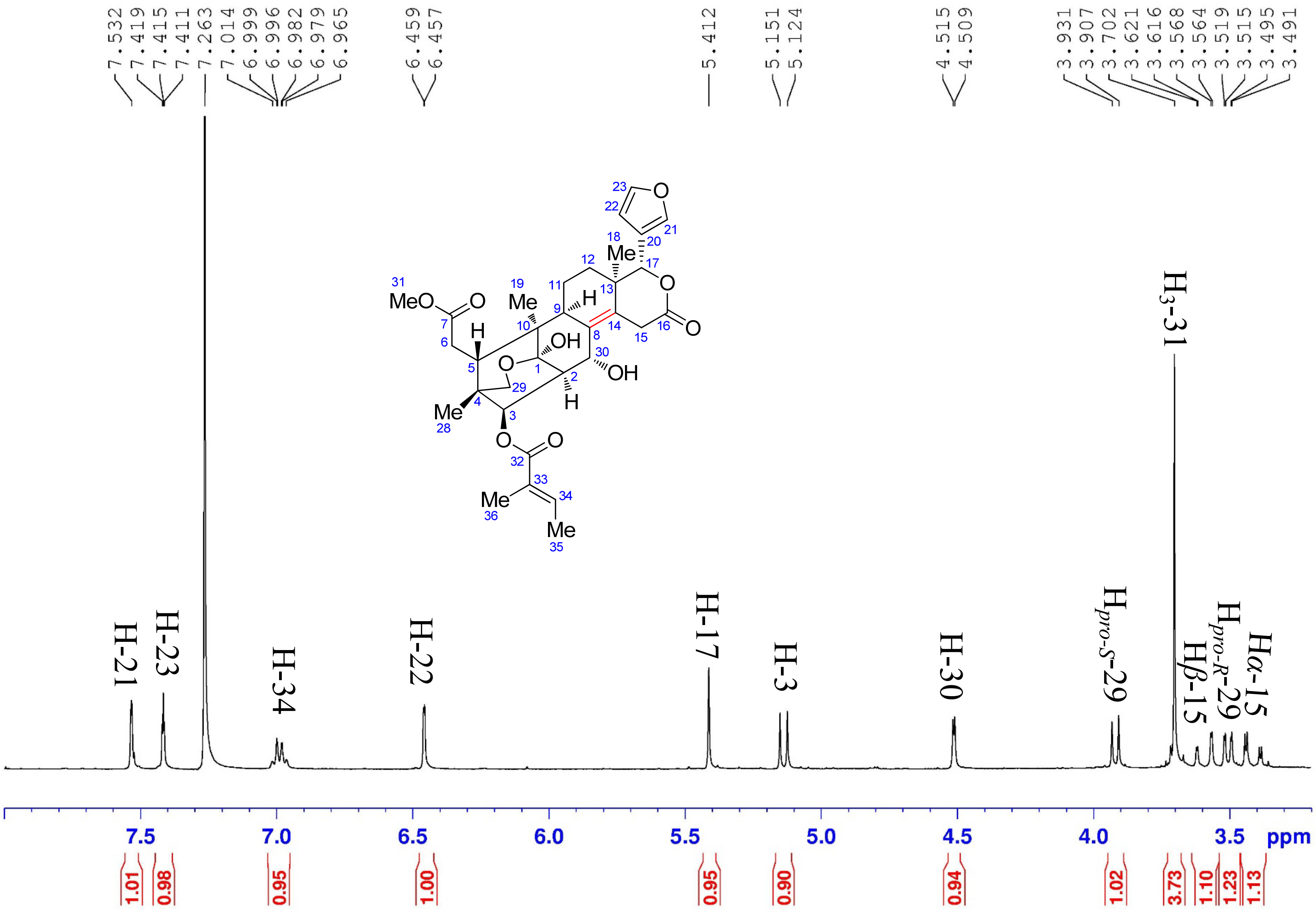

^{1}H NMR (400 MHz) spectrum of compound **3** in $CDCl_3$

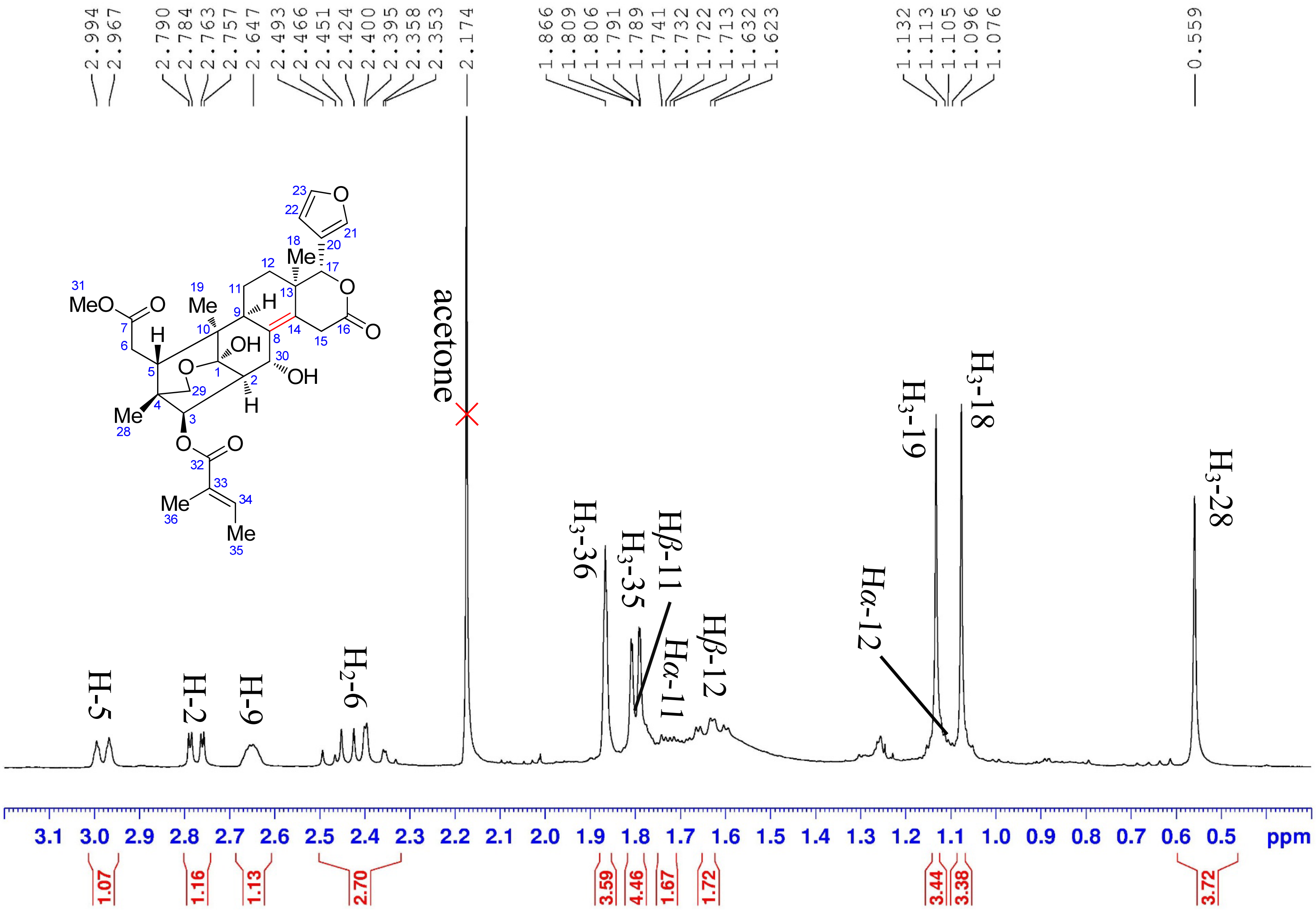

^{13}C NMR (100 MHz) spectrum of compound **3** in $CDCl_3$

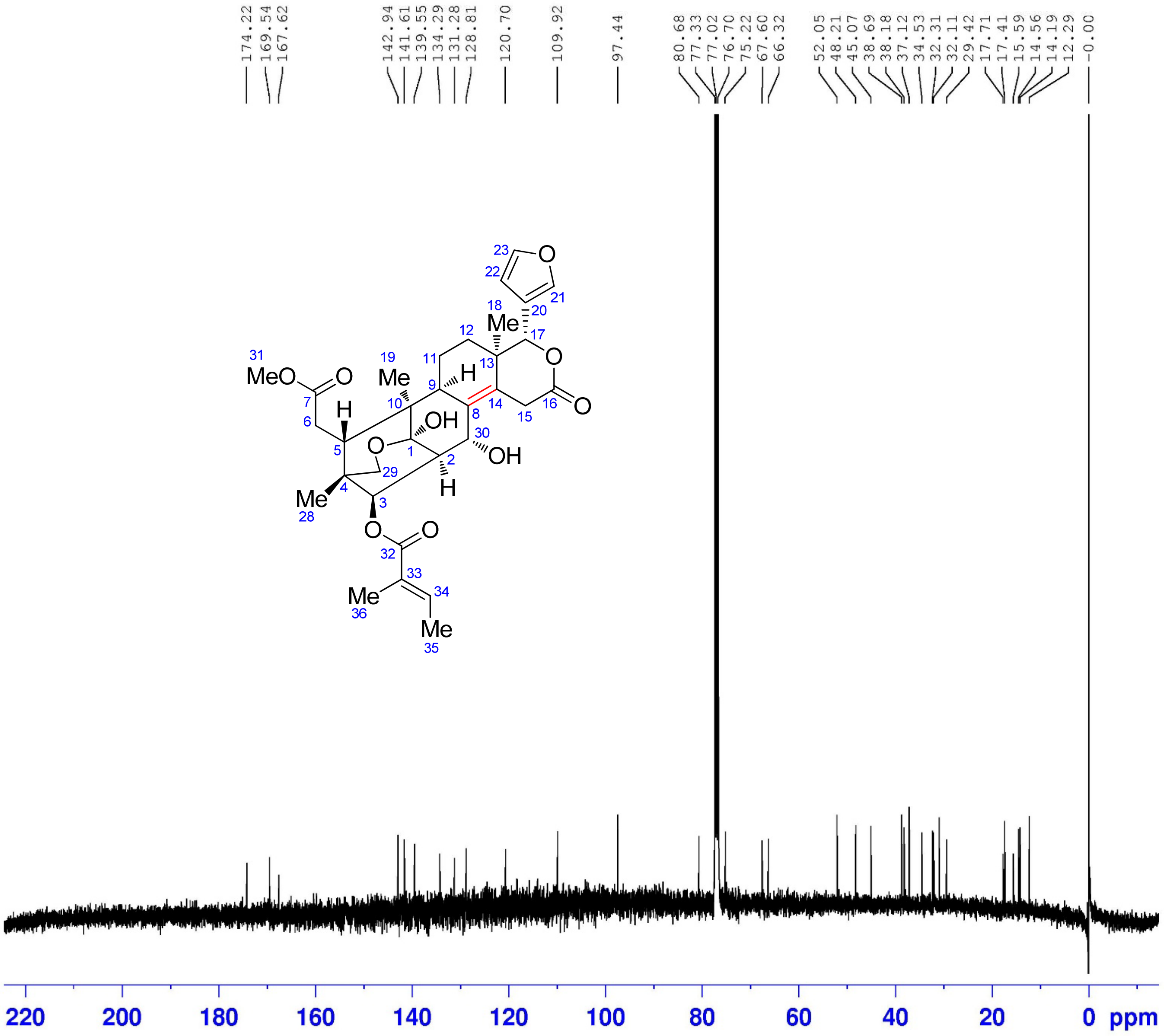

NAME	ZXP-R-18-3-1-3-3 new
EXPNO	2
PROCNO	1
Date_	20180109
Time	9.01
INSTRUM	spect
PROBHD	5 mm CPPBBO BB
PULPROG	zgpg30
TD	65536
SOLVENT	CDCl3
NS	1024
DS	4
SWH	24038.461 Hz
FIDRES	0.366798 Hz
AQ	1.3631988 sec
RG	73.92
DW	20.800 usec
DE	18.00 usec
TE	297.0 K
D1	2.00000000 sec
D11	0.03000000 sec
TD0	1

======== CHANNEL f1 ========

SFO1	100.6233324 MHz
NUC1	13C
P1	10.00 usec
SI	32768
SF	100.6127690 MHz
WDW	EM
SSB	0
LB	1.00 Hz
GB	0
PC	1.40

^{13}C NMR (100 MHz) spectrum of compound **3** in $CDCl_3$

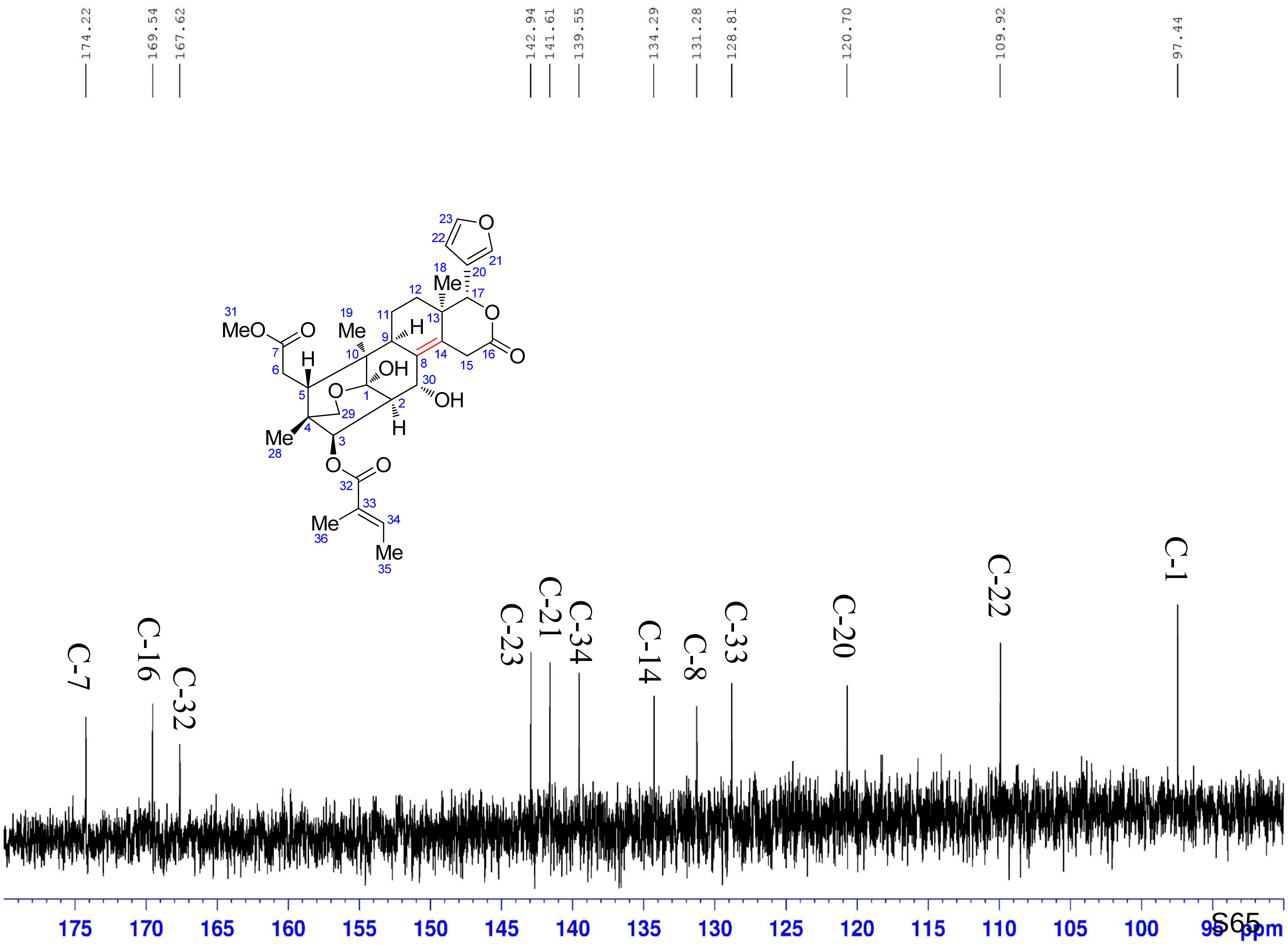

^{13}C NMR (100 MHz) spectrum of compound **3** in $CDCl_3$

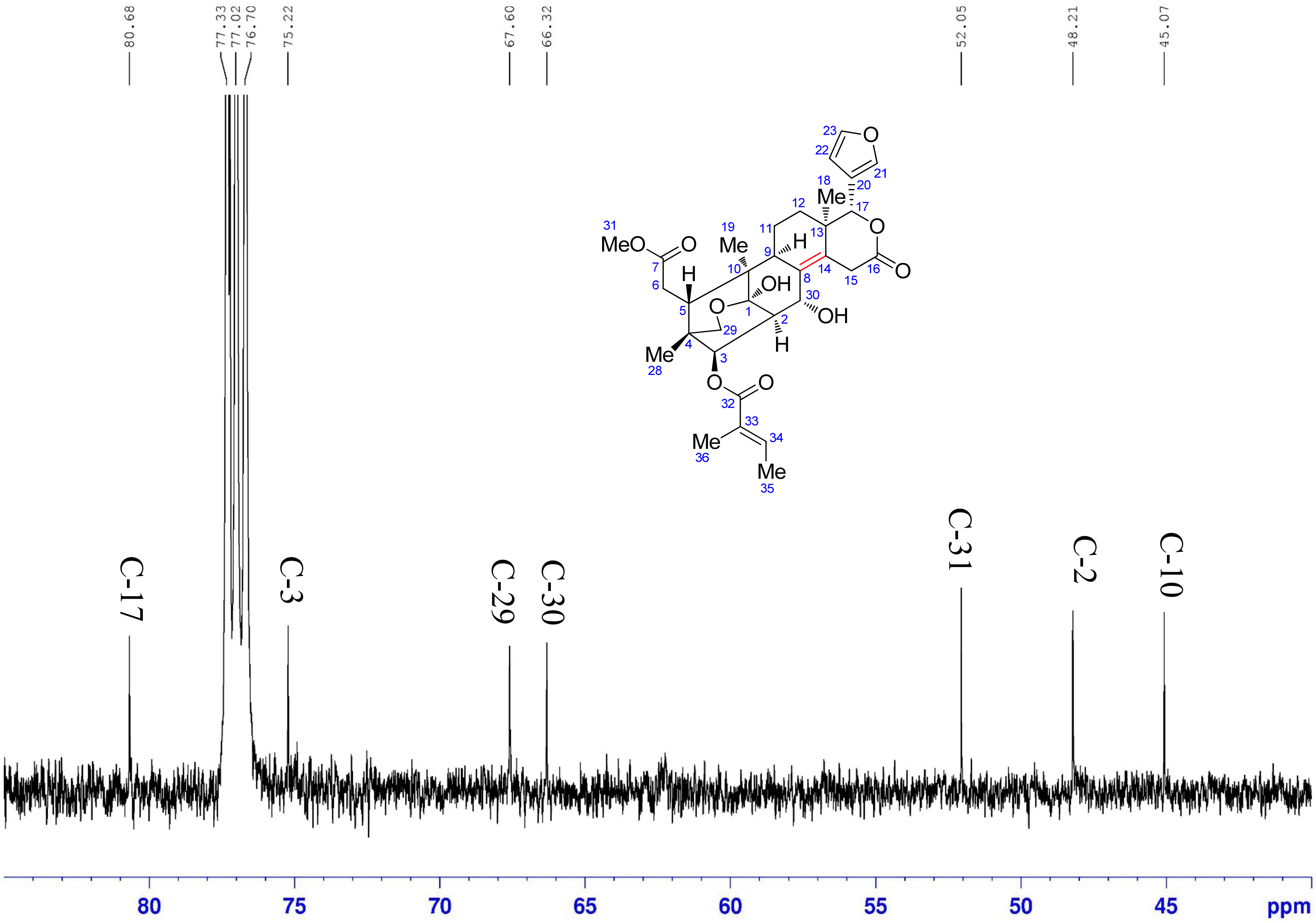

^{13}C NMR (100 MHz) spectrum of compound **3** in $CDCl_3$

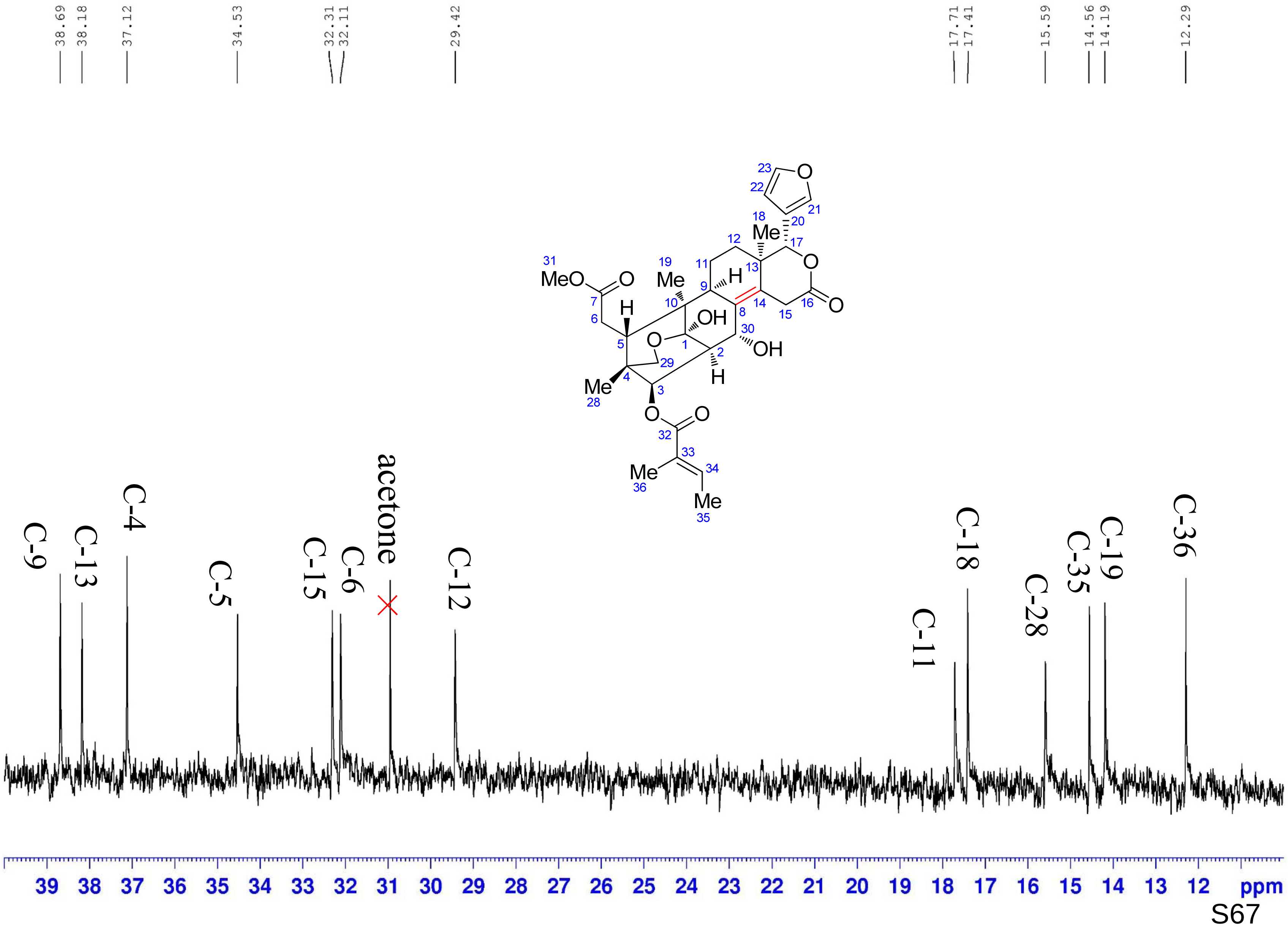

DEPT135 (100 MHz) spectrum of compound **3** in $CDCl_3$

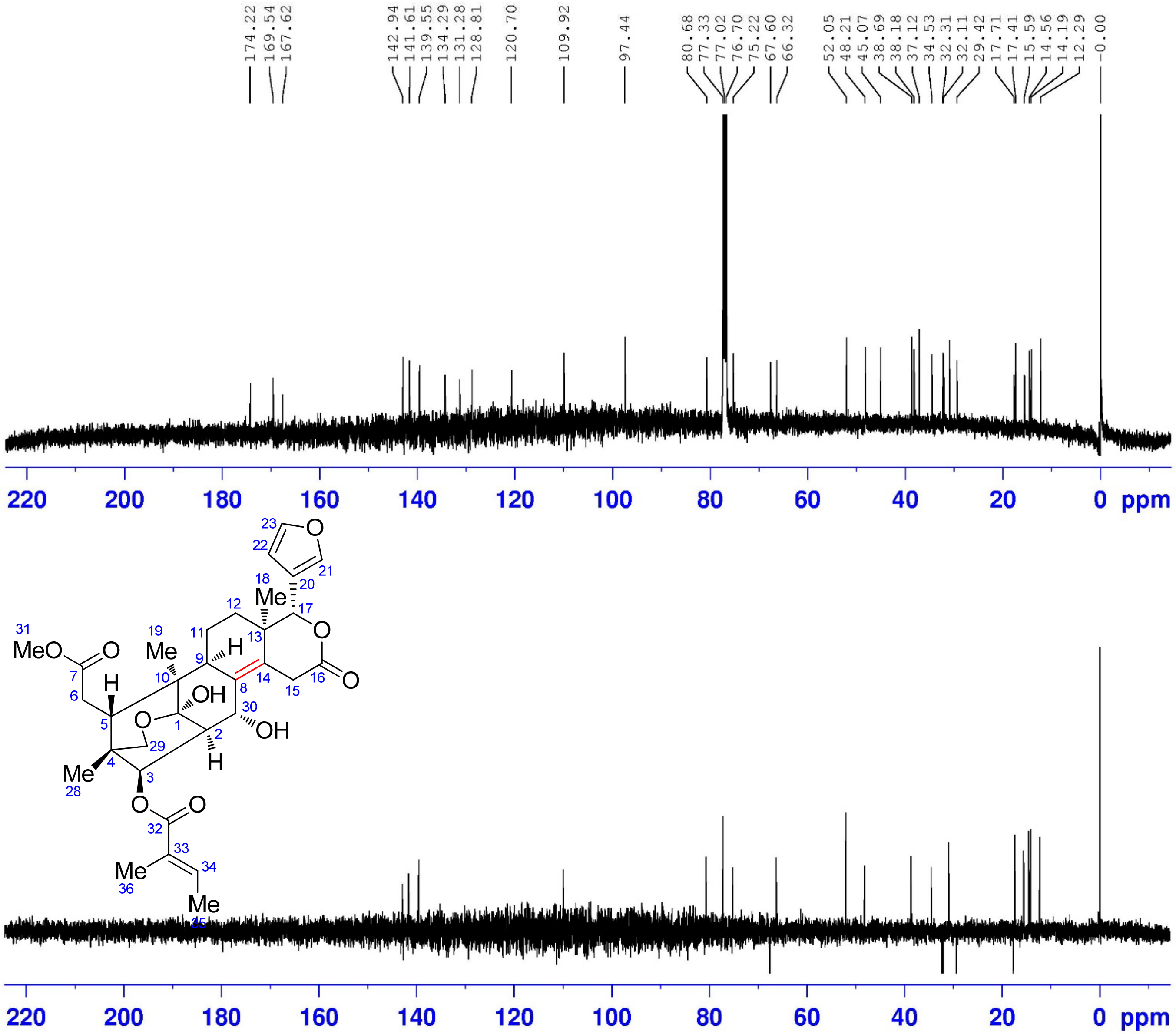

NAME	ZXP-R-18-3-1-3-3 new
EXPNO	2
PROCNO	1
Date_	20180109
Time	9.01
INSTRUM	spect
PROBHD	5 mm CPPBBO BB
PULPROG	zgpg30
TD	65536
SOLVENT	CDCl3
NS	1024
DS	4
SWH	24038.461 Hz
FIDRES	0.366798 Hz
AQ	1.3631988 sec
RG	73.92
DW	20.800 usec
DE	18.00 usec
TE	297.0 K
D1	2.00000000 sec
D11	0.03000000 sec
TD0	1

======== CHANNEL f1 ========

SFO1	100.6233324 MHz
NUC1	13C
P1	10.00 usec
SI	32768
SF	100.6127690 MHz
WDW	EM
SSB	0
LB	1.00 Hz
GB	0
PC	1.40

DEPT135 (100 MHz) spectrum of compound **3** in $CDCl_3$

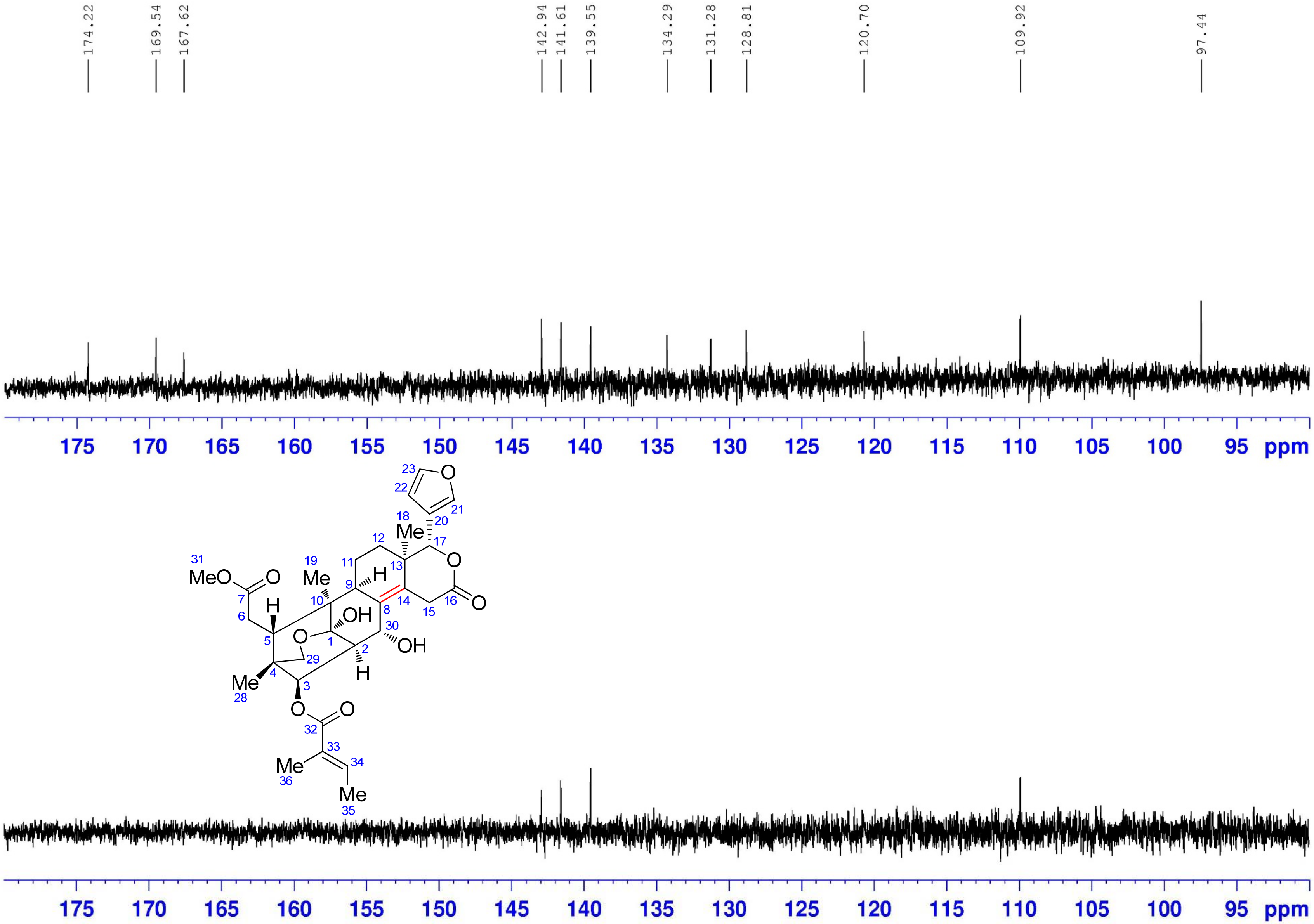

DEPT135 (100 MHz) spectrum of compound **3** in $CDCl_3$

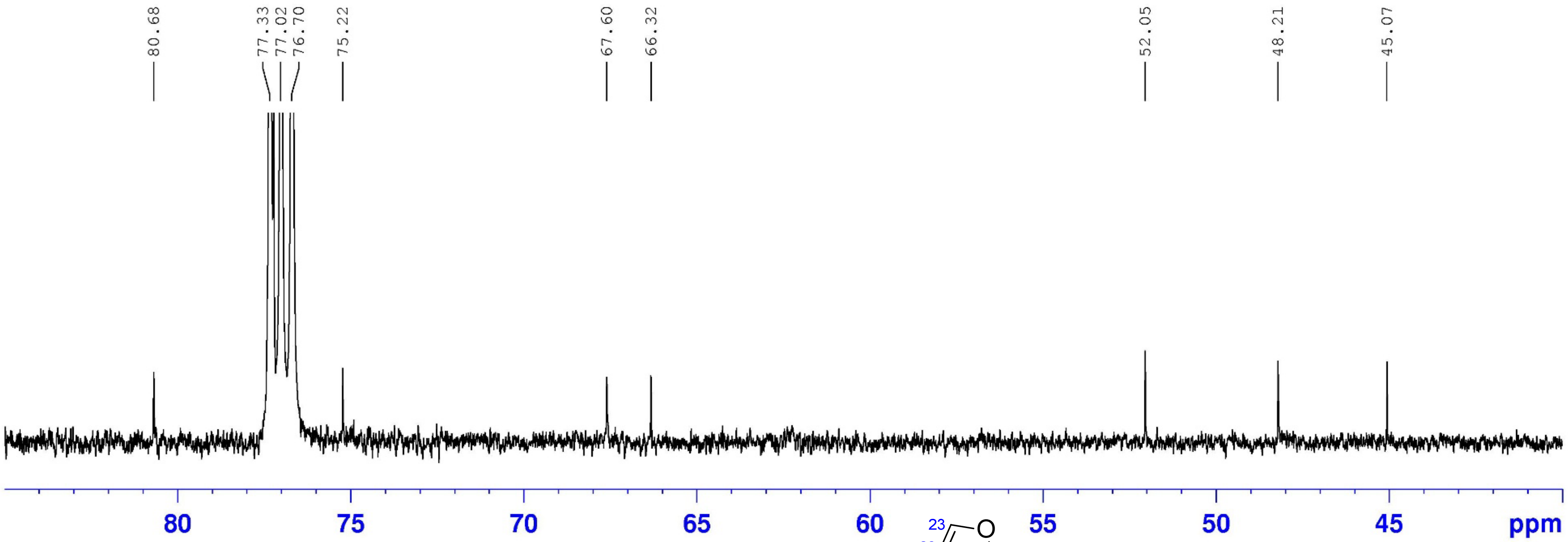

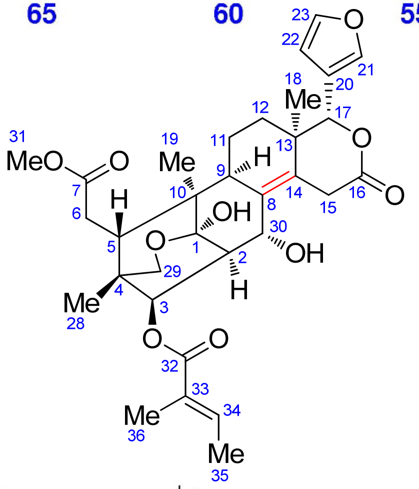

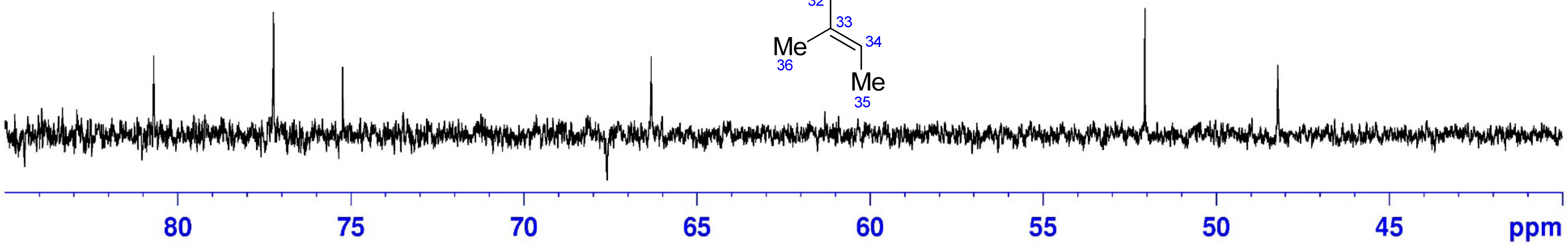

DEPT135 (100 MHz) spectrum of compound **3** in $CDCl_3$

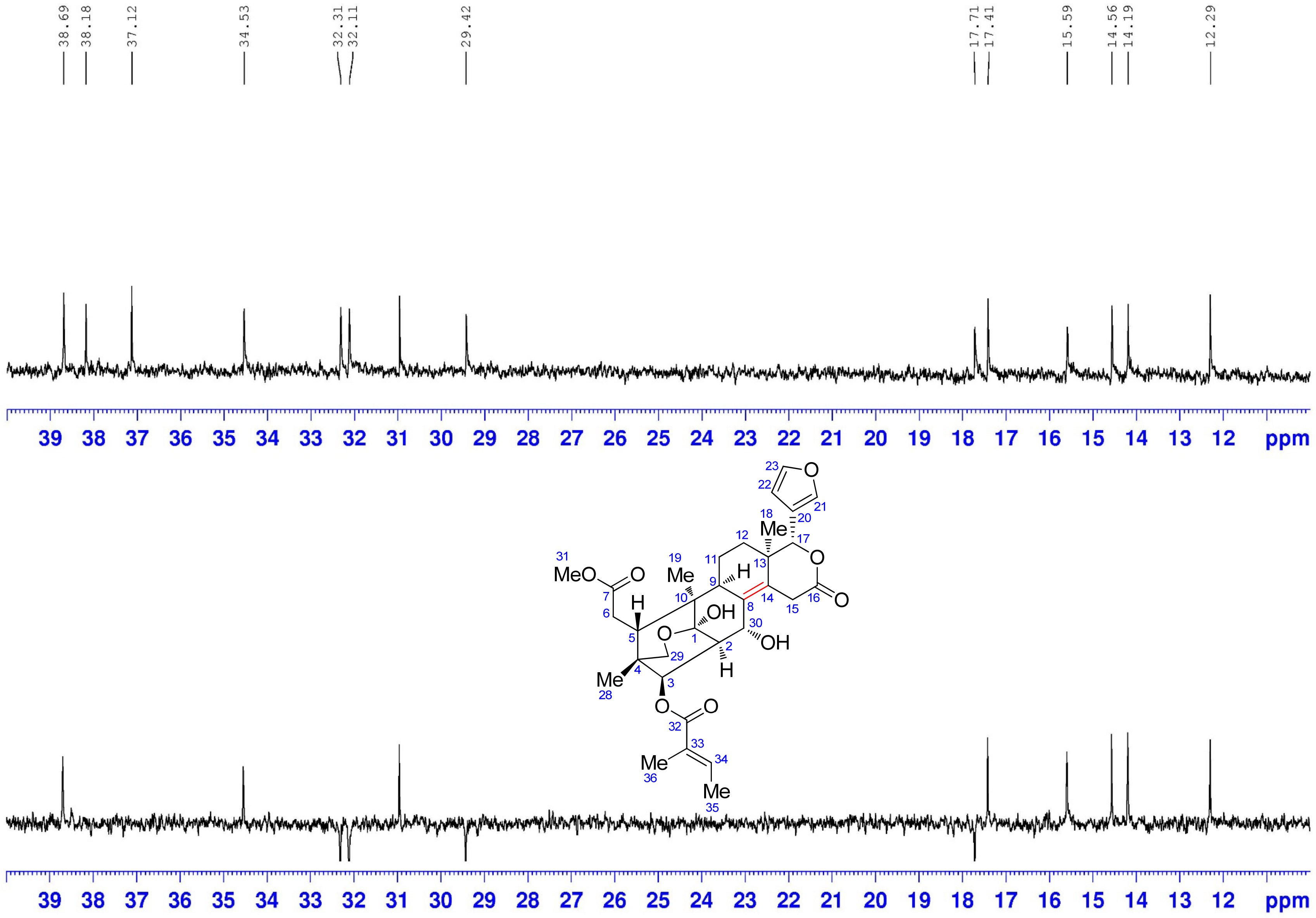

^{1}H-^{1}H COSY (400 MHz) spectrum of compound **3** in $CDCl_3$

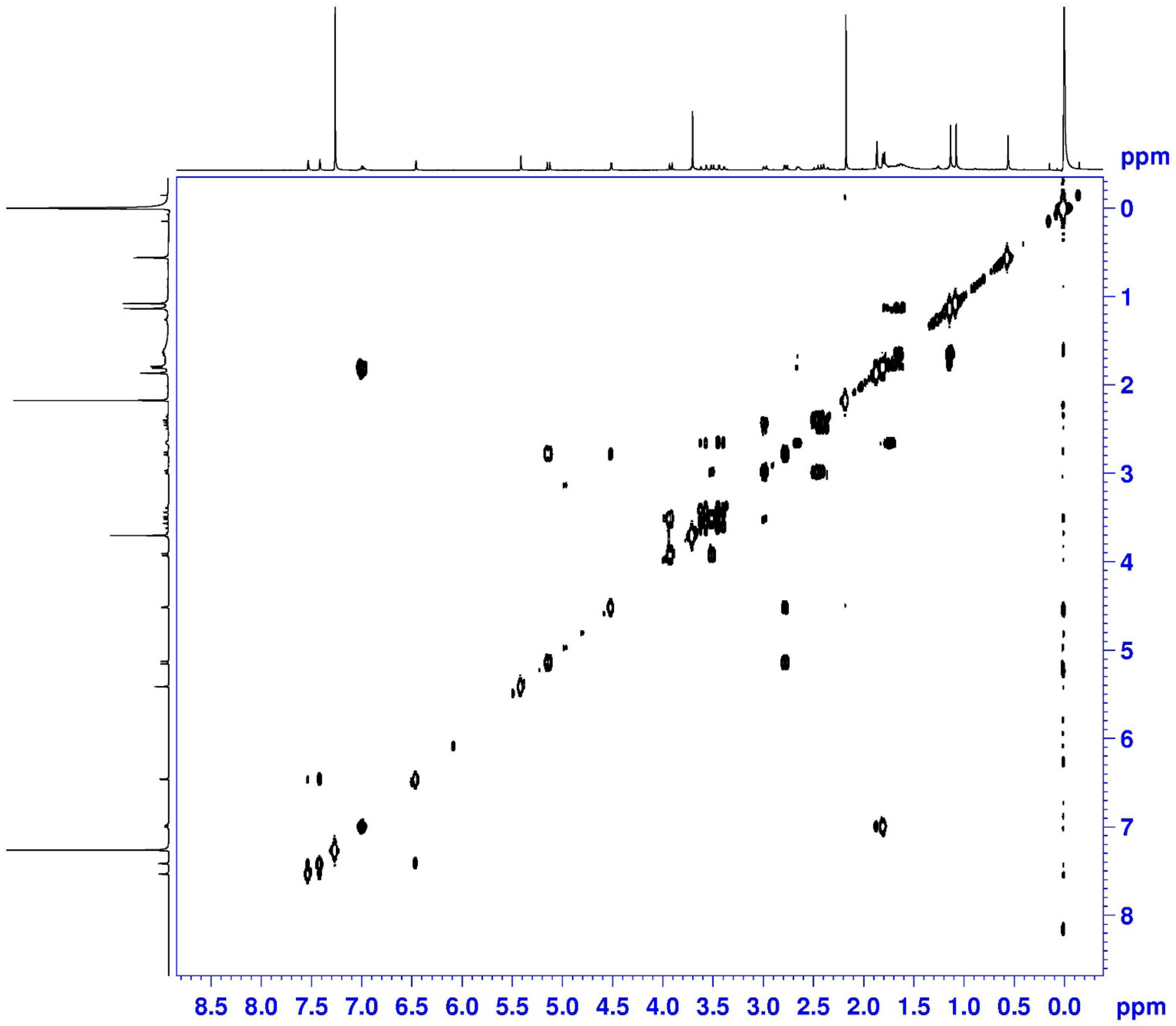


```
NAME       ZXP-R-18-3-1-3-3 new
EXPNO                 4
PROCNO                1
Date_          20180122
Time              23.49
INSTRUM           spect
PROBHD    5 mm CPPBBO BB
PULPROG       cosygpppqf
TD                 2048
SOLVENT           CDCl3
NS                    8
DS                    8
SWH            3906.250 Hz
FIDRES         1.907349 Hz
AQ            0.2621940 sec
RG                208.5
DW              128.000 usec
DE                10.00 usec
TE                297.0 K
D0           0.00000300 sec
D1           1.89678097 sec
D11          0.03000000 sec
D12          0.00002000 sec
D13          0.00000400 sec
D16          0.00020000 sec
IN0          0.00025600 sec

======== CHANNEL f1 ========
SFO1        400.1318006 MHz
NUC1                 1H
P0                11.50 usec
P1                11.50 usec
P17             2500.00 usec
ND0                   1
TD                  128
SFO1           400.1318 MHz
FIDRES        30.517578 Hz
SW                9.762 ppm
FnMODE               QF
SI                 1024
SF          400.1300064 MHz
WDW               QSINE
SSB                   0
LB                 0.00 Hz
GB                    0
PC                 1.40
SI                 1024
MC2                  QF
SF          400.1300071 MHz
WDW               QSINE
SSB                   0
LB                 0.00 Hz
```

^{1}H-^{1}H COSY (400 MHz) spectrum of compound **3** in $CDCl_3$

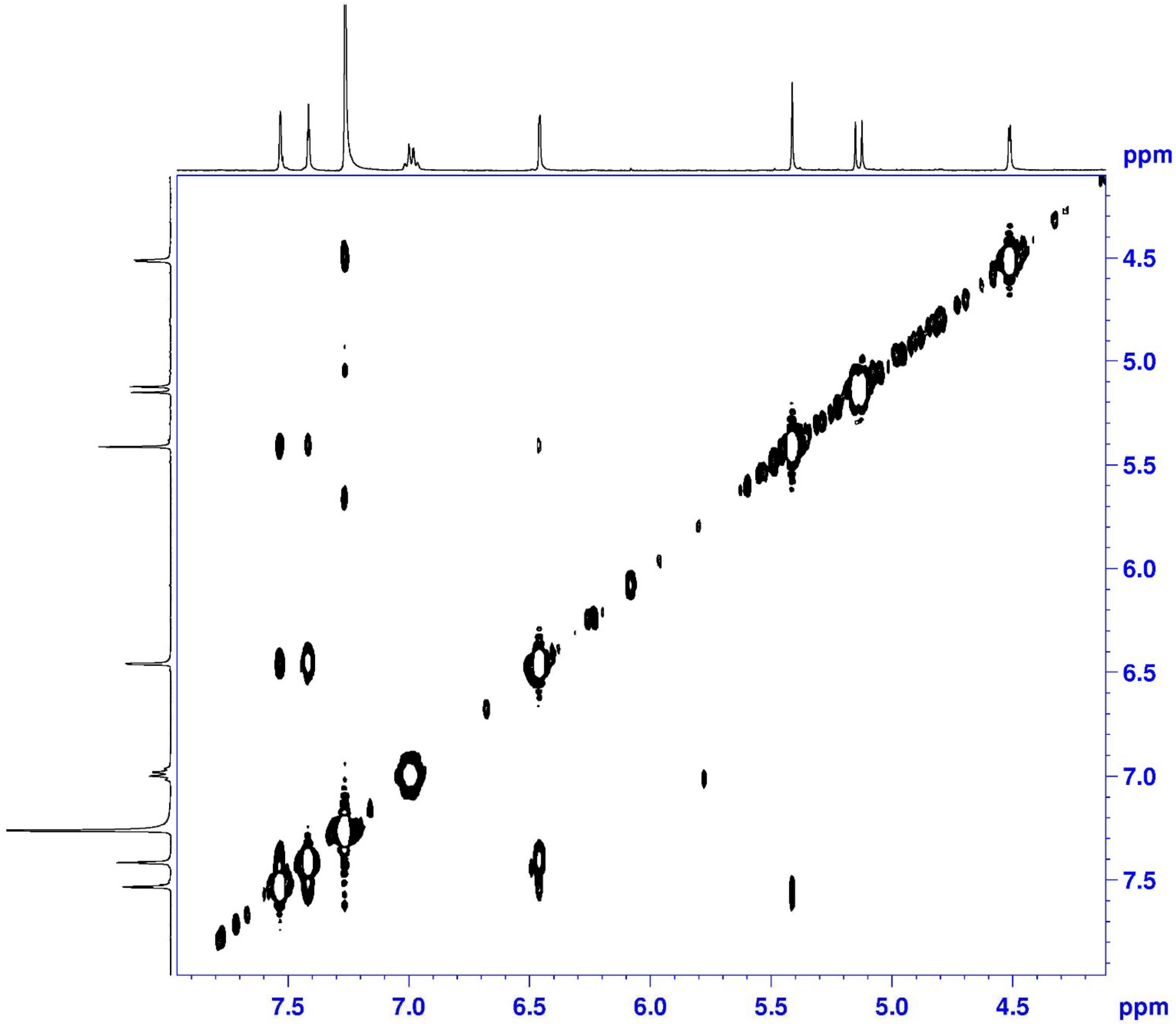

^{1}H-^{1}H COSY (400 MHz) spectrum of compound **3** in $CDCl_3$

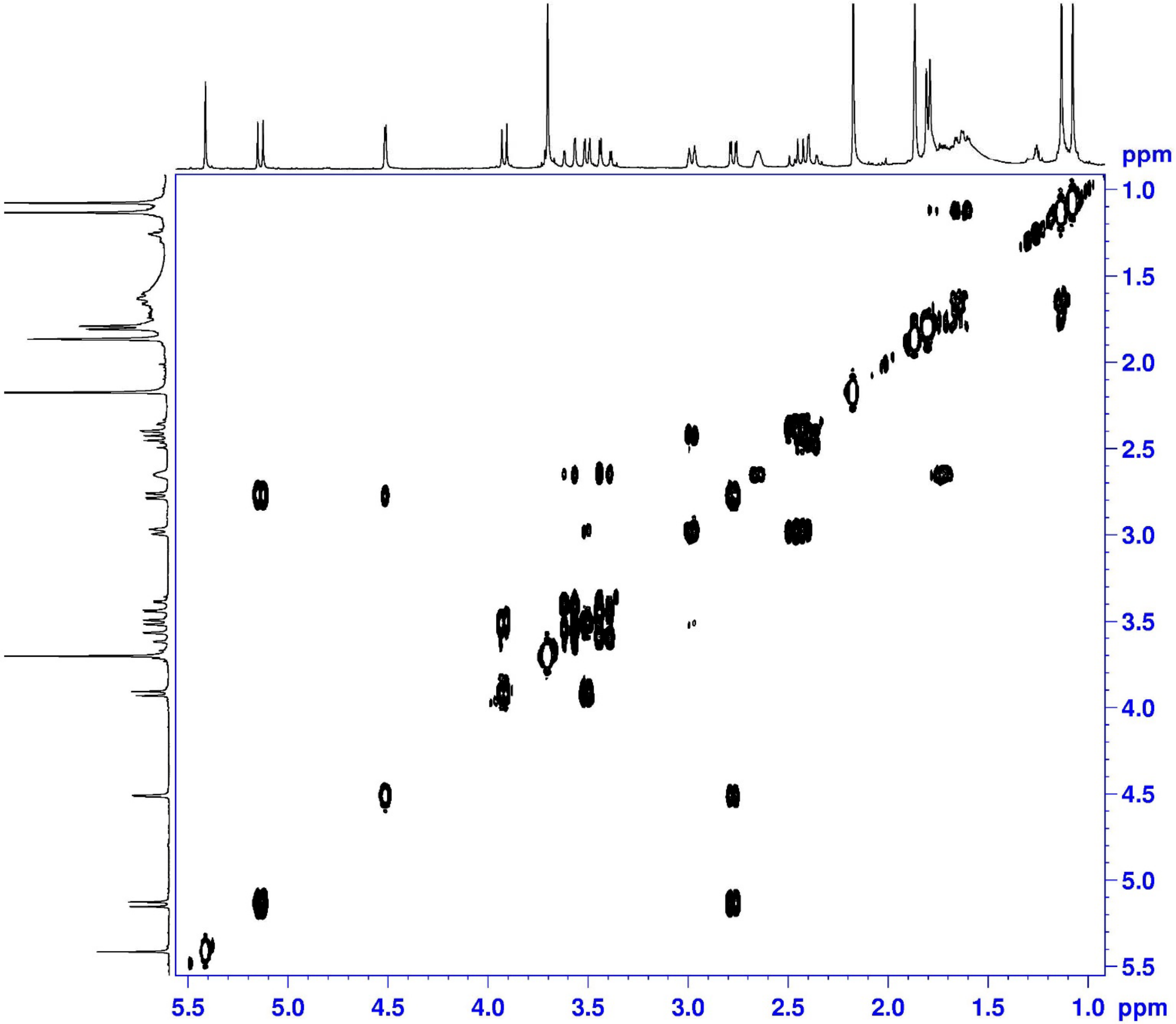

HSQC (400 MHz) spectrum of compound **3** in $CDCl_3$

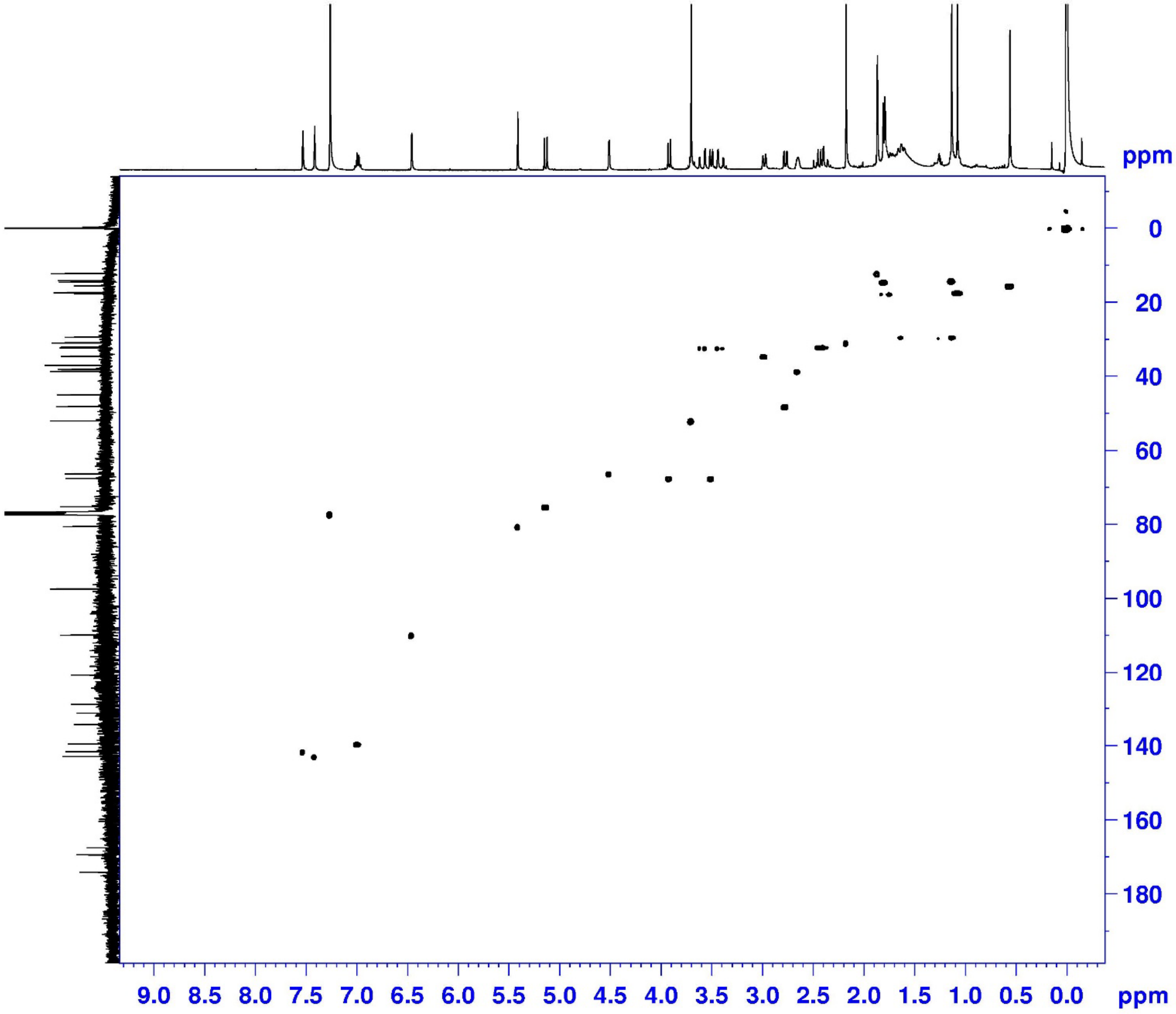


```
NAME      ZXP-R-18-3-1-3-3 new
EXPNO                    5
PROCNO                   1
Date_             20180123
Time                  0.28
INSTRUM              spect
PROBHD    5 mm CPPBBO BB
PULPROG        hsqcetgpsi2
TD                    1024
SOLVENT              CDCl3
NS                      16
DS                      16
SWH               4302.926 Hz
FIDRES            4.202076 Hz
AQ               0.1190388 sec
RG                   208.5
DW                 116.200 usec
DE                   10.00 usec
TE                   297.0 K
CNST2          145.0000000
D0              0.00000300 sec
D1              1.46497905 sec
D4              0.00172414 sec
D11             0.03000000 sec
D16             0.00020000 sec
D24             0.00086207 sec
IN0             0.00002080 sec
ZGOPTNS

======== CHANNEL f1 ========
SFO1           400.1320007 MHz
NUC1                    1H
P1                   11.50 usec
P2                   23.00 usec
P28                   0.00 usec
ND0                      2
TD                     256
SFO1              100.6233 MHz
FIDRES           93.900238 Hz
SW                 238.896 ppm
FnMODE        Echo-Antiecho
SI                    1024
SF             400.1300055 MHz
WDW                  QSINE
SSB                      2
LB                    0.00 Hz
GB                       0
PC                    1.40
SI                    1024
MC2           echo-antiecho
SF             100.6127548 MHz
WDW                  QSINE
SSB                      2
LB                    0.00 Hz
```

HSQC (400 MHz) spectrum of compound **3** in $CDCl_3$

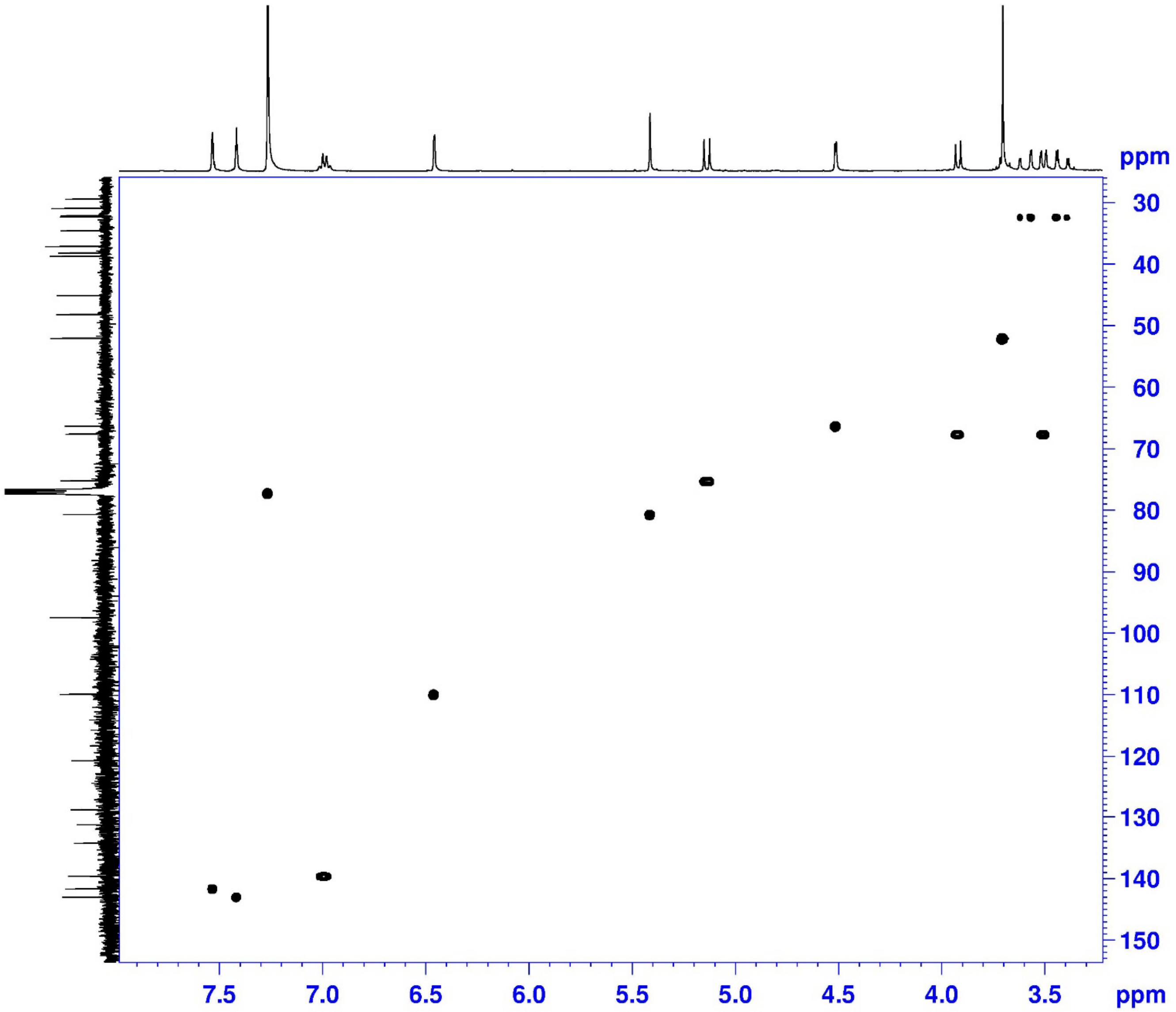

HSQC (400 MHz) spectrum of compound **3** in $CDCl_3$

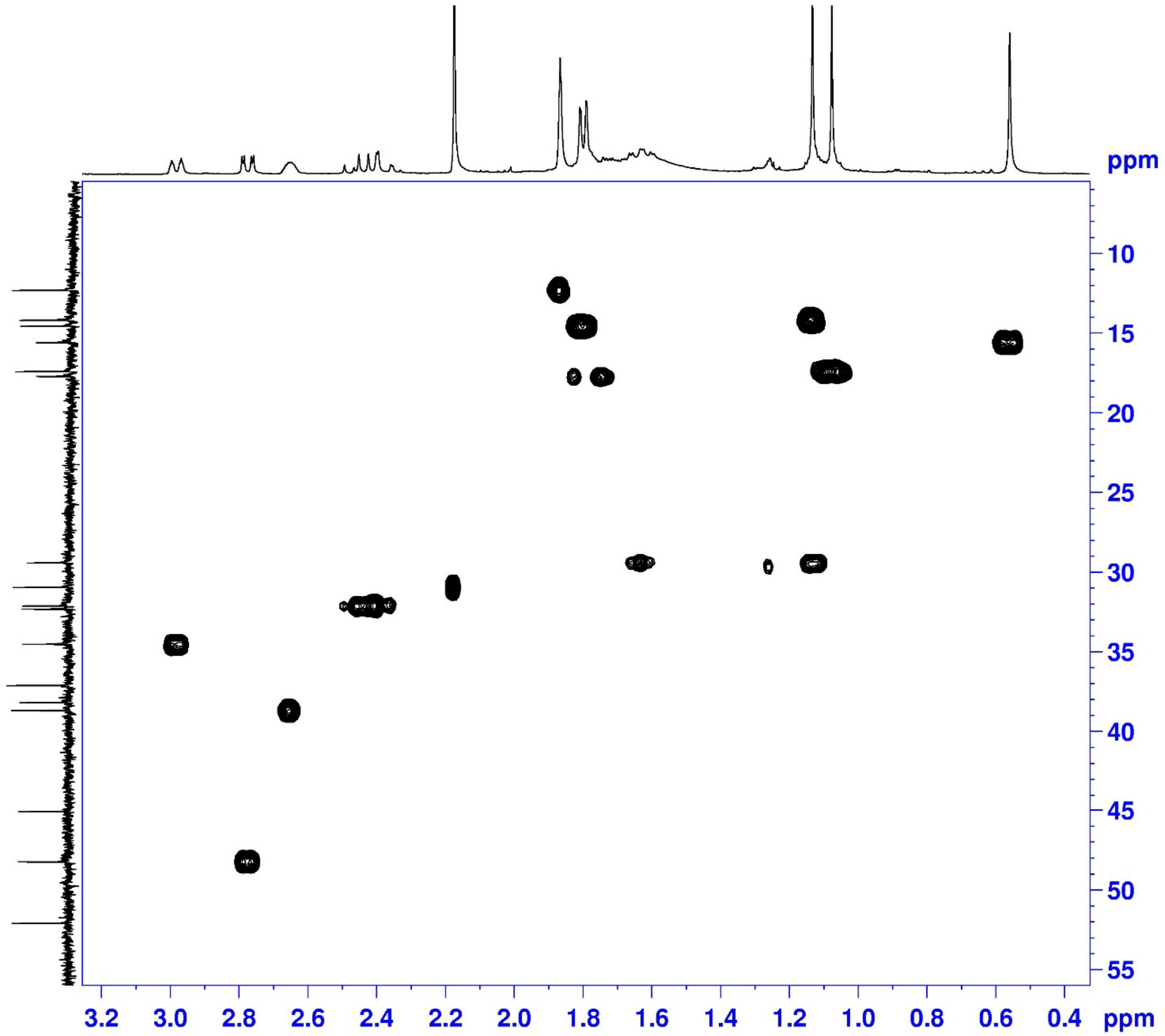

HMBC (400 MHz) spectrum of compound **3** in $CDCl_3$

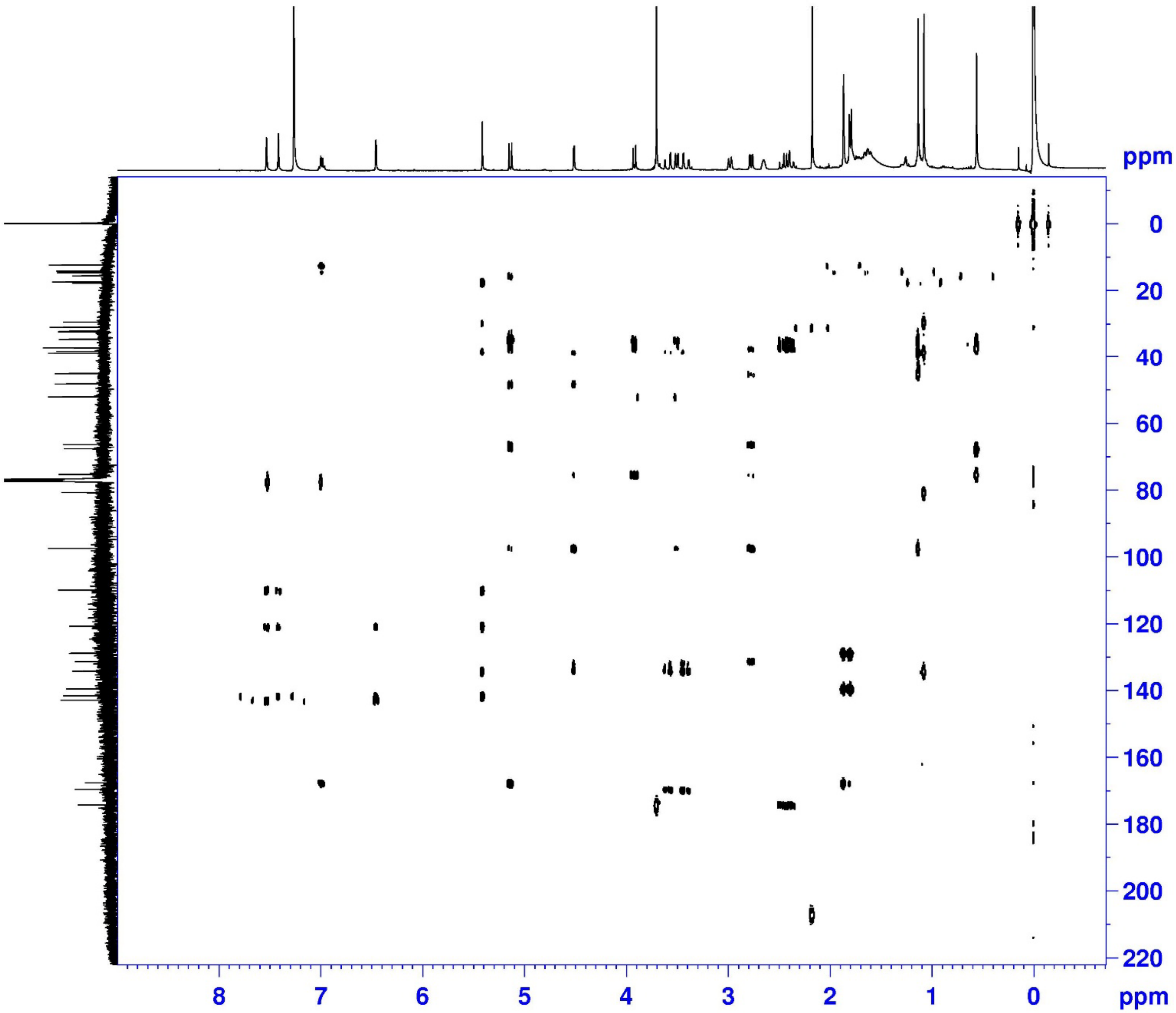

NAME	ZXP-R-18-3-1-3-3 new
EXPNO	6
PROCNO	1
Date_	20180123
Time	2.19
INSTRUM	spect
PROBHD	5 mm CPPBBO BB
PULPROG	hmbcgplpndqf
TD	4096
SOLVENT	CDCl3
NS	32
DS	16
SWH	5197.505 Hz
FIDRES	1.268922 Hz
AQ	0.3940852 sec
RG	208.5
DW	96.200 usec
DE	10.00 usec
TE	297.0 K
CNST2	145.0000000
CNST13	10.0000000
D0	0.00000300 sec
D1	1.50000000 sec
D2	0.00344828 sec
D6	0.05000000 sec
D16	0.00020000 sec
IN0	0.00002080 sec
======== CHANNEL f1 ========	
SFO1	400.1323208 MHz
NUC1	1H
P1	11.50 usec
P2	23.00 usec
ND0	2
TD	128
SFO1	100.6233 MHz
FIDRES	187.800476 Hz
SW	238.896 ppm
FnMODE	QF
SI	2048
SF	400.1300076 MHz
WDW	SINE
SSB	0
LB	0.00 Hz
GB	0
PC	1.40
SI	1024
MC2	QF
SF	100.6127574 MHz
WDW	SINE
SSB	0
LB	0.00 Hz

HMBC (400 MHz) spectrum of compound **3** in $CDCl_3$

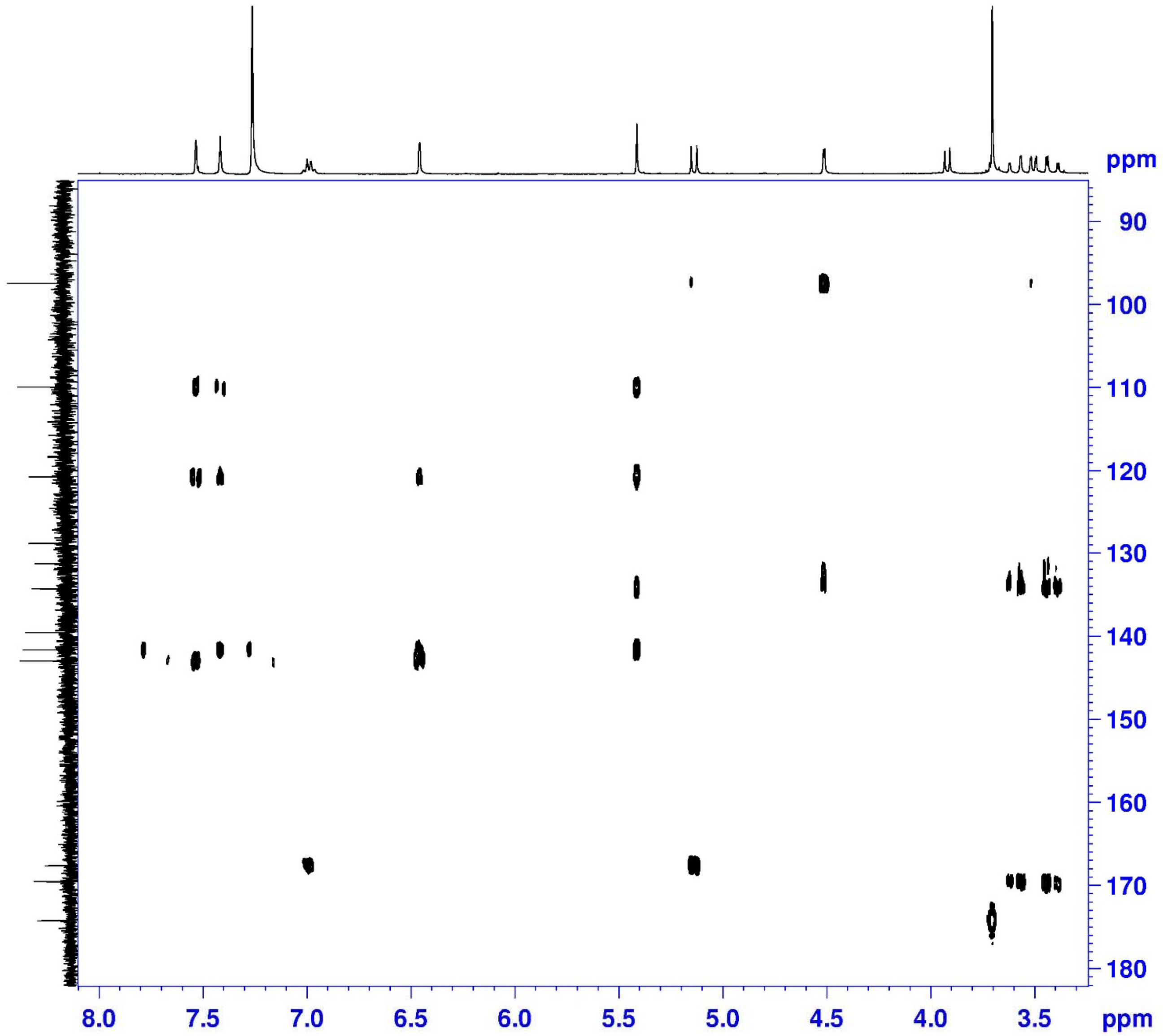

HMBC (400 MHz) spectrum of compound **3** in $CDCl_3$

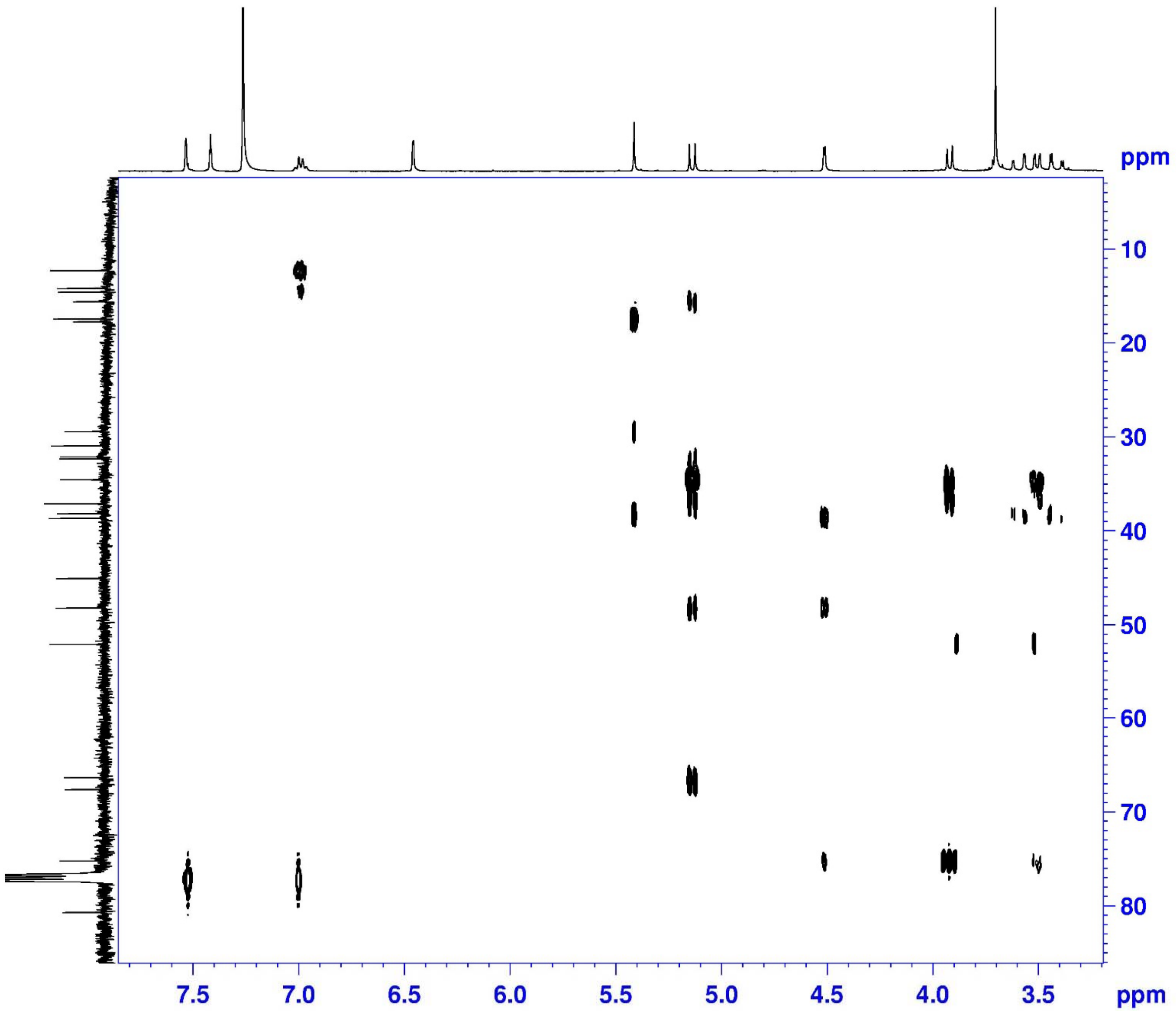

HMBC (400 MHz) spectrum of compound **3** in $CDCl_3$

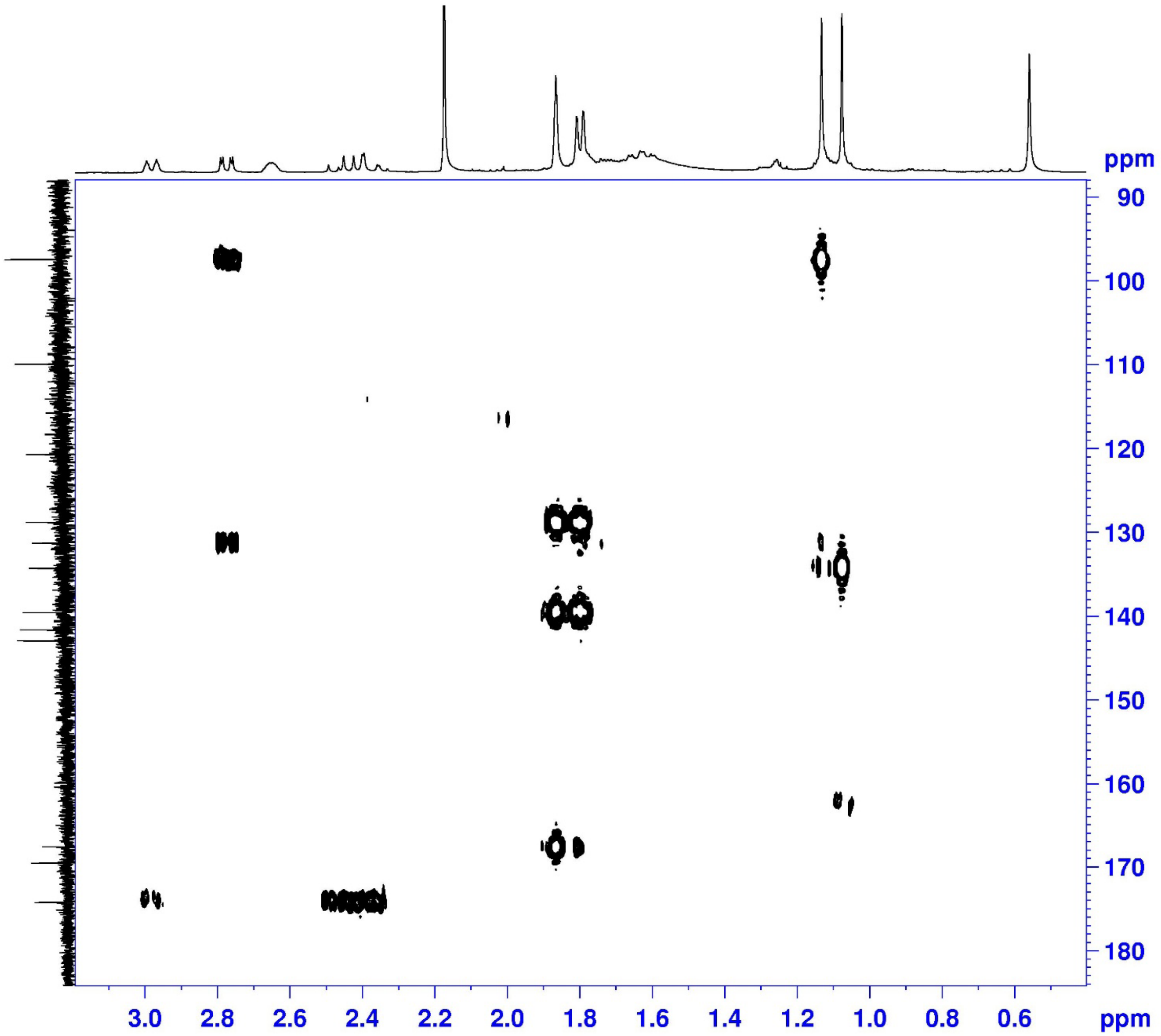

HMBC (400 MHz) spectrum of compound **3** in $CDCl_3$

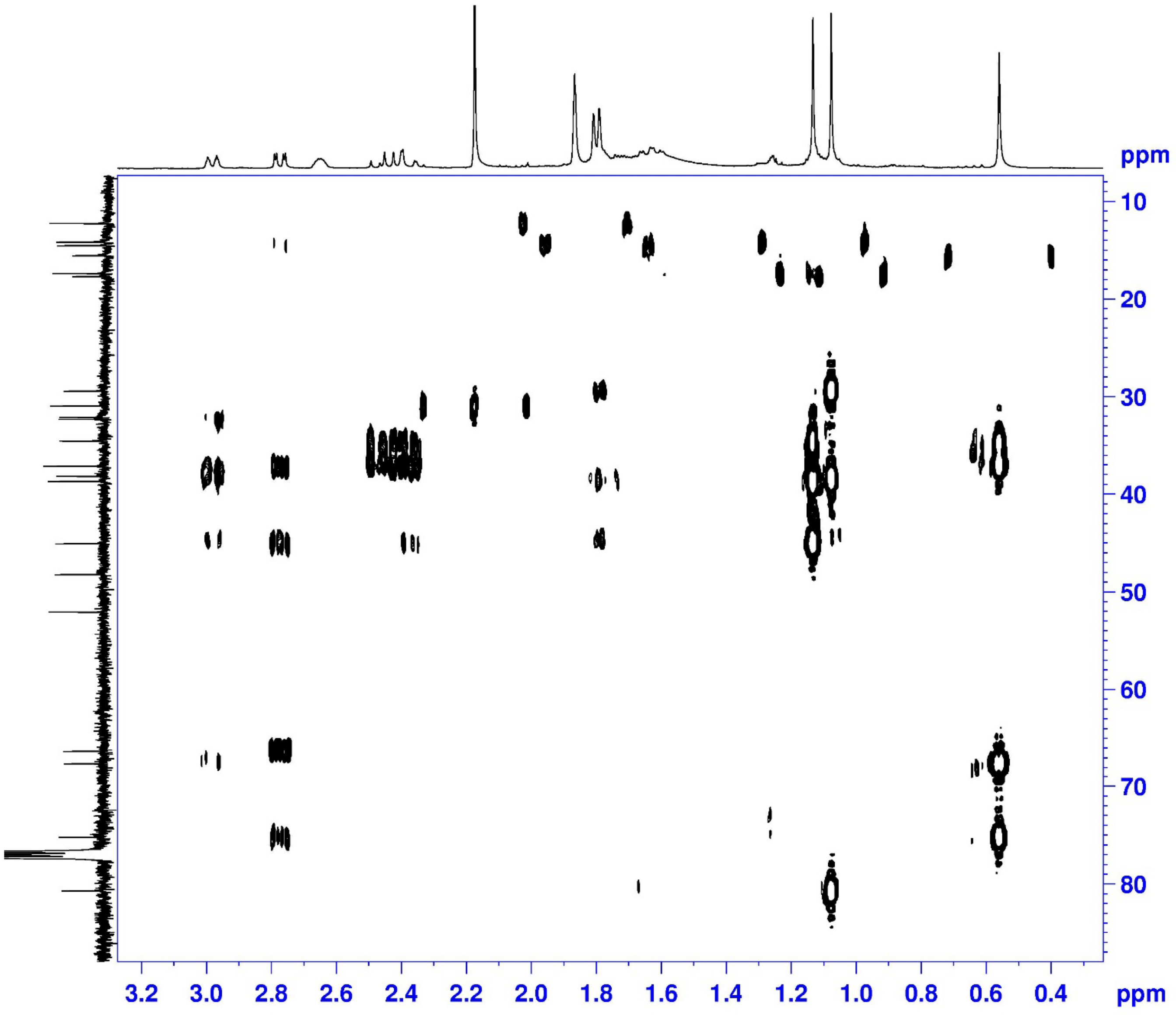

NOESY (400 MHz) spectrum of compound **3** in $CDCl_3$

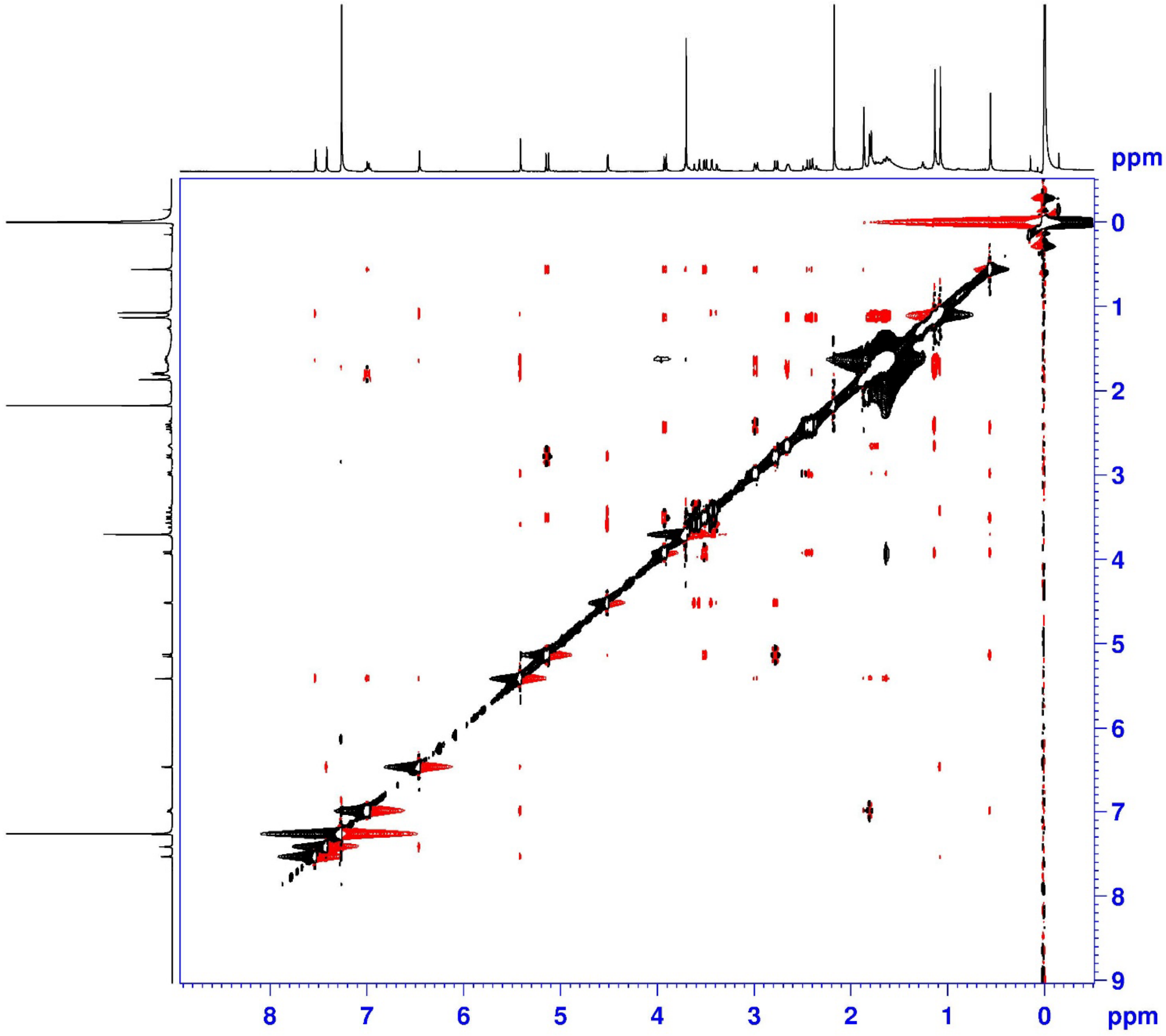


```
NAME        ZXP-R-18-3-1-3-3 new
EXPNO                      7
PROCNO                     1
Date_               20180123
Time                    4.35
INSTRUM                spect
PROBHD      5 mm CPPBBO BB
PULPROG        noesygpphpp
TD                      2048
SOLVENT                CDCl3
NS                        16
DS                        32
SWH                 4000.000 Hz
FIDRES              1.953125 Hz
AQ                 0.2560500 sec
RG                     208.5
DW                   125.000 usec
DE                     10.00 usec
TE                     297.0 K
D0                0.00011036 sec
D1                1.99385595 sec
D8                0.30000001 sec
D11               0.03000000 sec
D12               0.00002000 sec
D16               0.00020000 sec
IN0               0.00025000 sec

======== CHANNEL f1 ========
SFO1             400.1318006 MHz
NUC1                      1H
P1                     11.50 usec
P2                     23.00 usec
P17                  2500.00 usec
ND0                        1
TD                       256
SFO1                400.1318 MHz
FIDRES             15.625000 Hz
SW                     9.997 ppm
FnMODE           States-TPPI
SI                      1024
SF               400.1300062 MHz
WDW                    QSINE
SSB                        2
LB                      0.00 Hz
GB                         0
PC                      1.00
SI                      1024
MC2              States-TPPI
SF               400.1300070 MHz
WDW                    QSINE
SSB                        2
LB                      0.00 Hz
GB                         0
```

NOESY (400 MHz) spectrum of compound **3** in $CDCl_3$

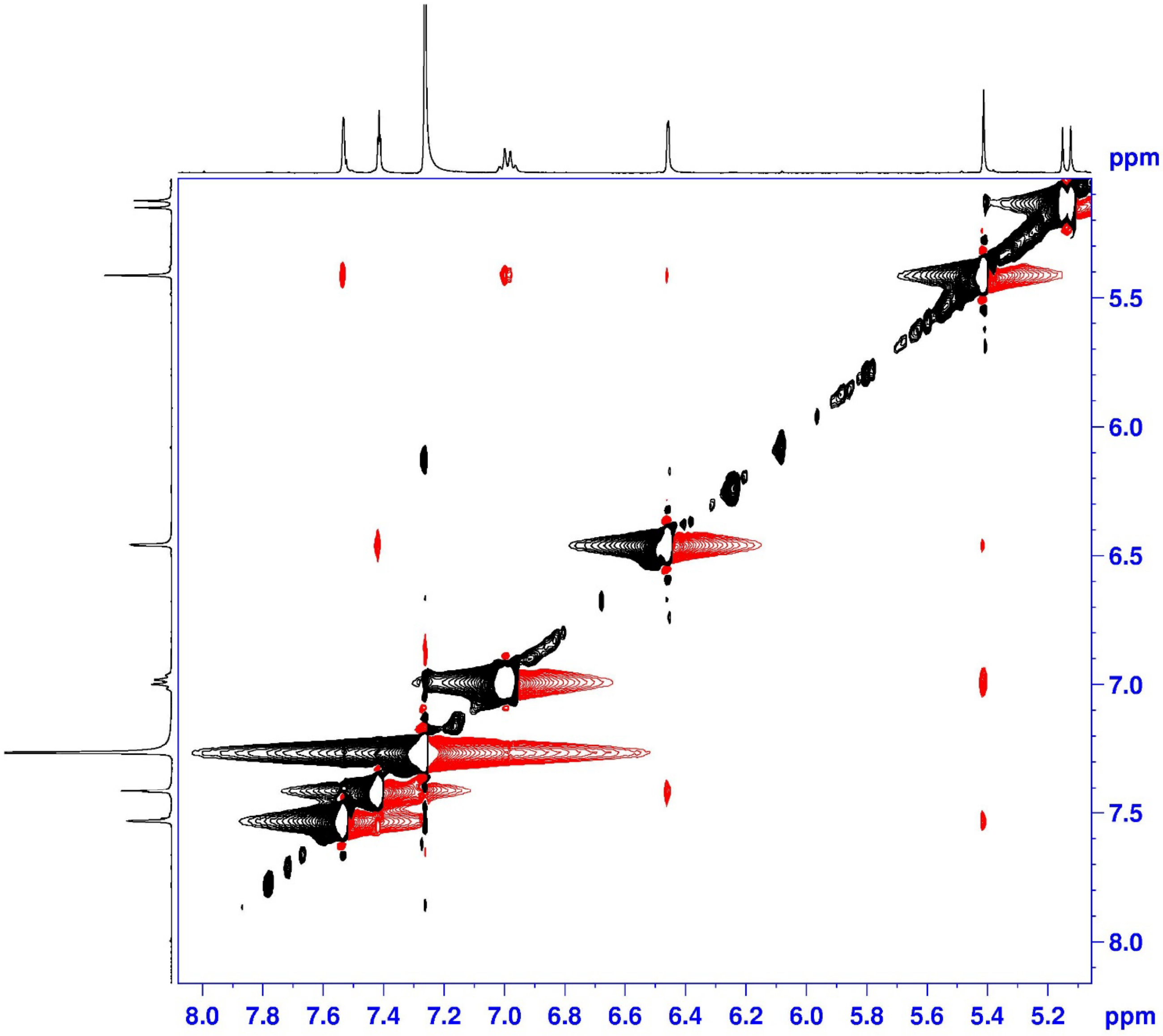

NOESY (400 MHz) spectrum of compound **3** in $CDCl_3$

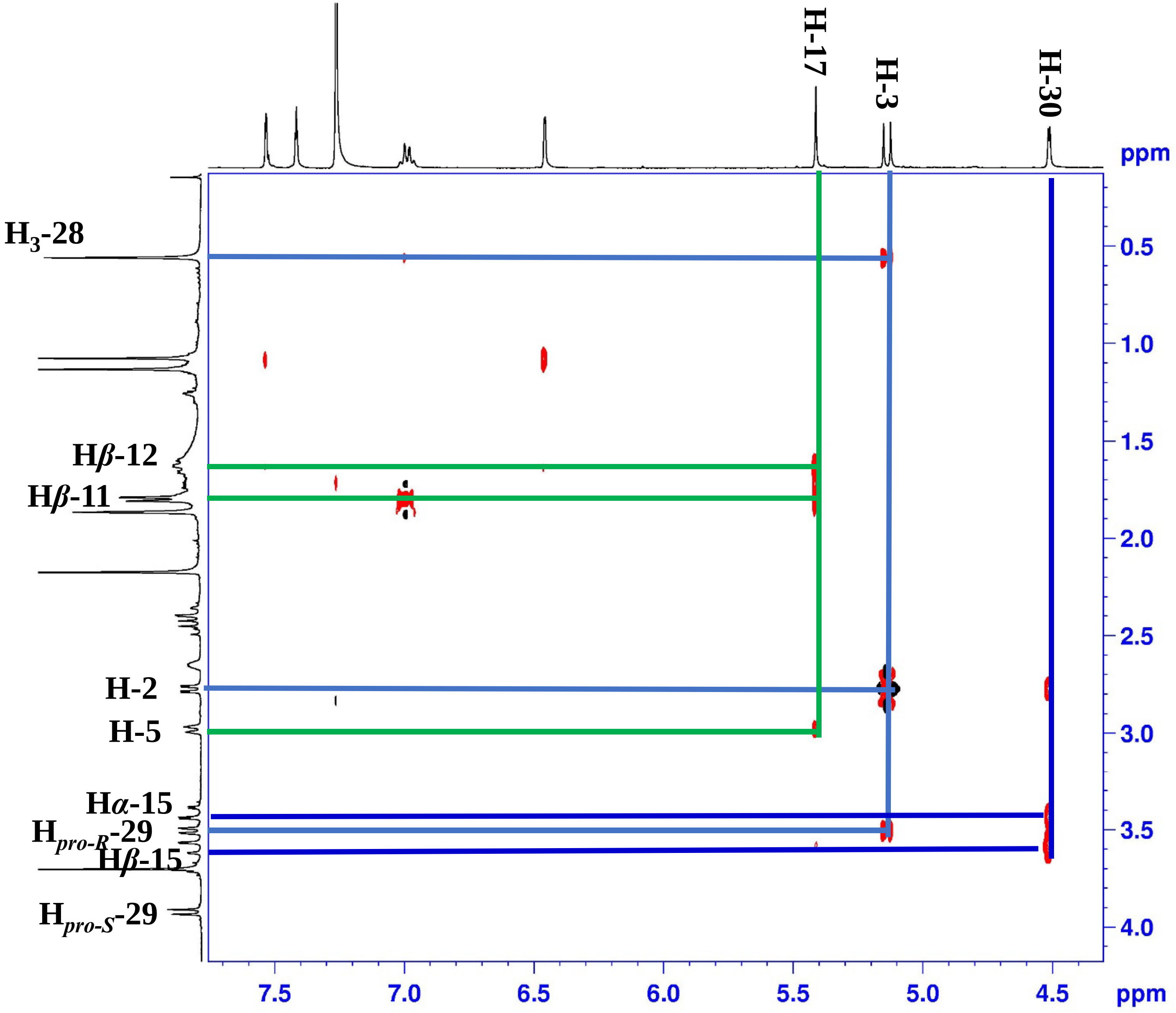

NOESY (400 MHz) spectrum of compound **3** in $CDCl_3$

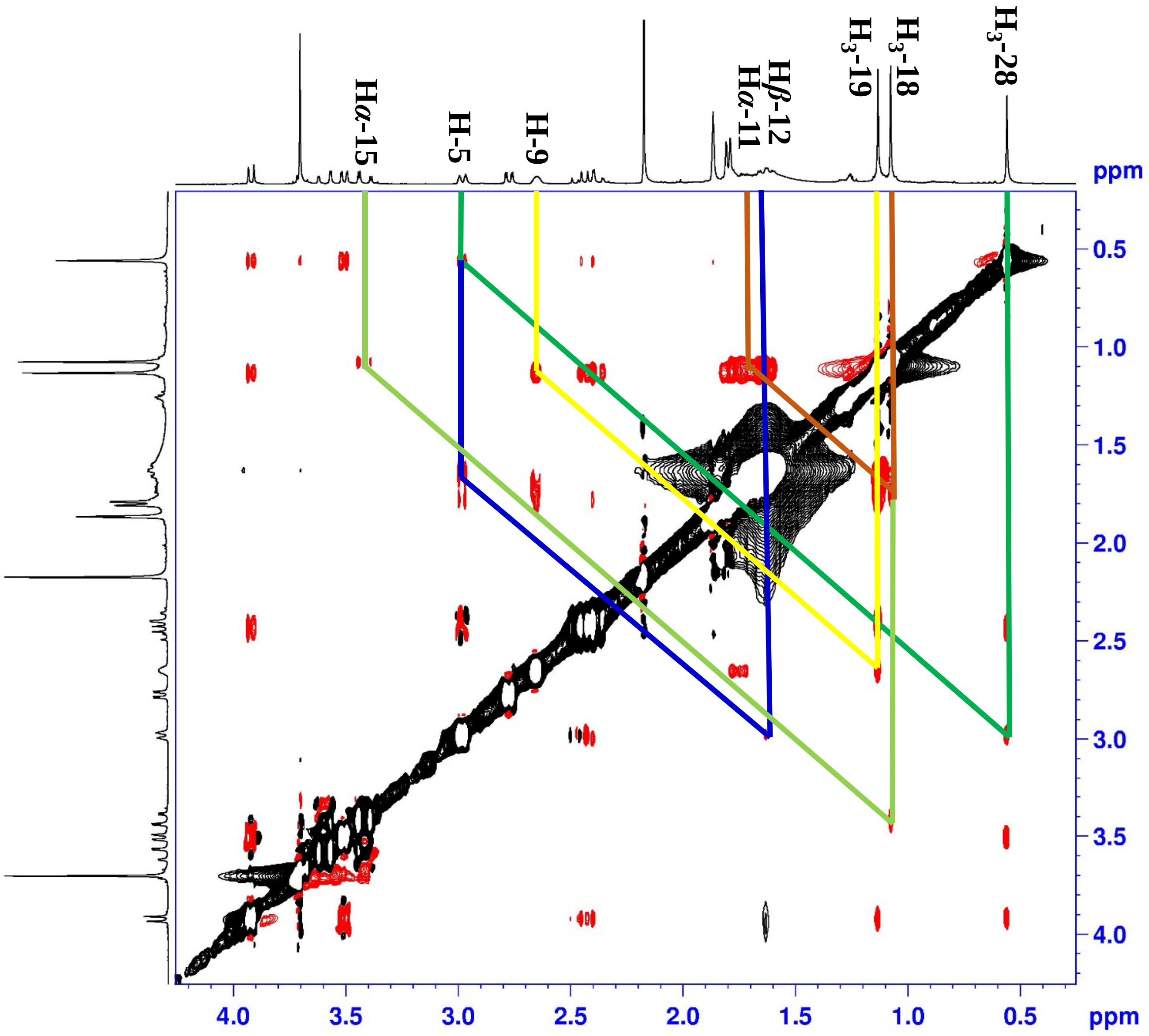

HR-ESIMS for compound **4**

Generic Display Report

Analysis Info

Analysis Name	D:\Data\MS\data\201809\zhouxiaopeng_ZXP-1_pos_3_01_5425.d
Method	LC_Direct Infusion_pos_100-1000mz.m
Sample Name	zhouxiaopeng_ZXP-1_pos
Comment	

Acquisition Date	9/7/2018 4:58:32 PM
Operator	SCSIO
Instrument	maXis

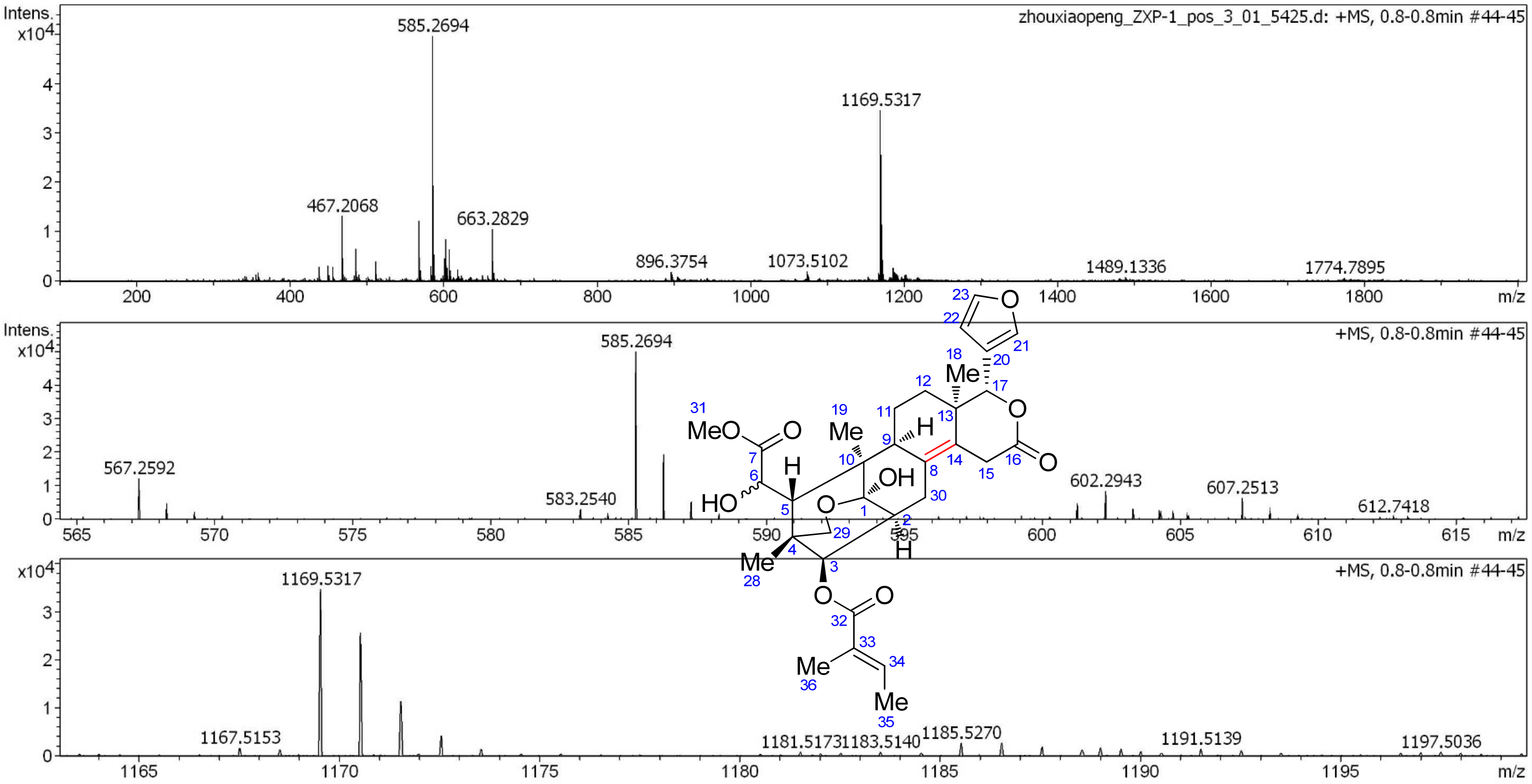

HR-ESIMS for compound **4**

Mass Spectrum SmartFormula Report

Analysis Info

Analysis Name	D:\Data\MS\data\201809\zhouxiaopeng_ZXP-1_pos_3_01_5425.d	Acquisition Date	9/7/2018 4:58:32 PM
Method	LC_Direct Infusion_pos_100-1000mz.m	Operator	SCSIO
Sample Name	zhouxiaopeng_ZXP-1_pos	Instrument	maXis 255552.00029
Comment			

Acquisition Parameter

Source Type	ESI	Ion Polarity	Positive	Set Nebulizer	0.4 Bar
Focus	Active	Set Capillary	4500 V	Set Dry Heater	180 °C
Scan Begin	100 m/z	Set End Plate Offset	-500 V	Set Dry Gas	4.0 l/min
Scan End	2000 m/z	Set Charging Voltage	0 V	Set Divert Valve	Waste
		Set Corona	0 nA	Set APCI Heater	0 °C

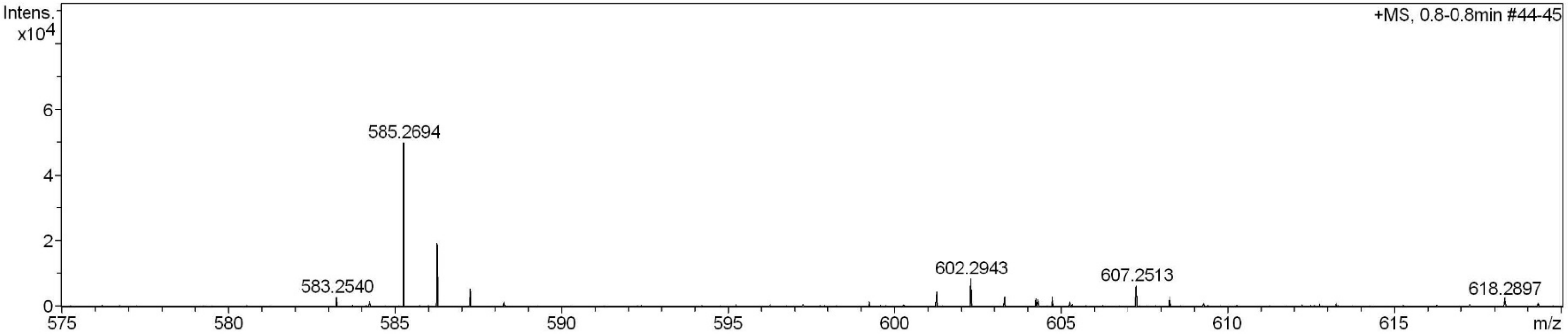

Meas. m/z	#	Ion Formula	Score	m/z	err [ppm]	err [mDa]	mSigma	rdb	e⁻ Conf	N-Rule
585.2694	1	C32H41O10	100.00	585.2694	-0.1	-0.1	22.1	12.5	even	ok
607.2513	1	C32H40NaO10	100.00	607.2514	-0.1	-0.0	38.7	12.5	even	ok
1169.5317	1	C64H81O20	100.00	1169.5316	-0.1	-0.1	26.9	24.5	even	ok

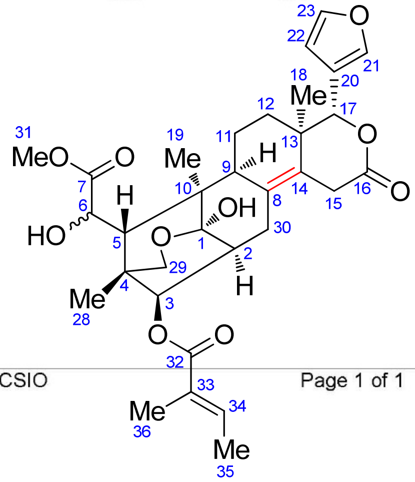

zhouxiaopeng_ZXP-1_pos_3_01_5425.d

^{1}H NMR (400 MHz) spectrum of compound **4** in $CDCl_3$

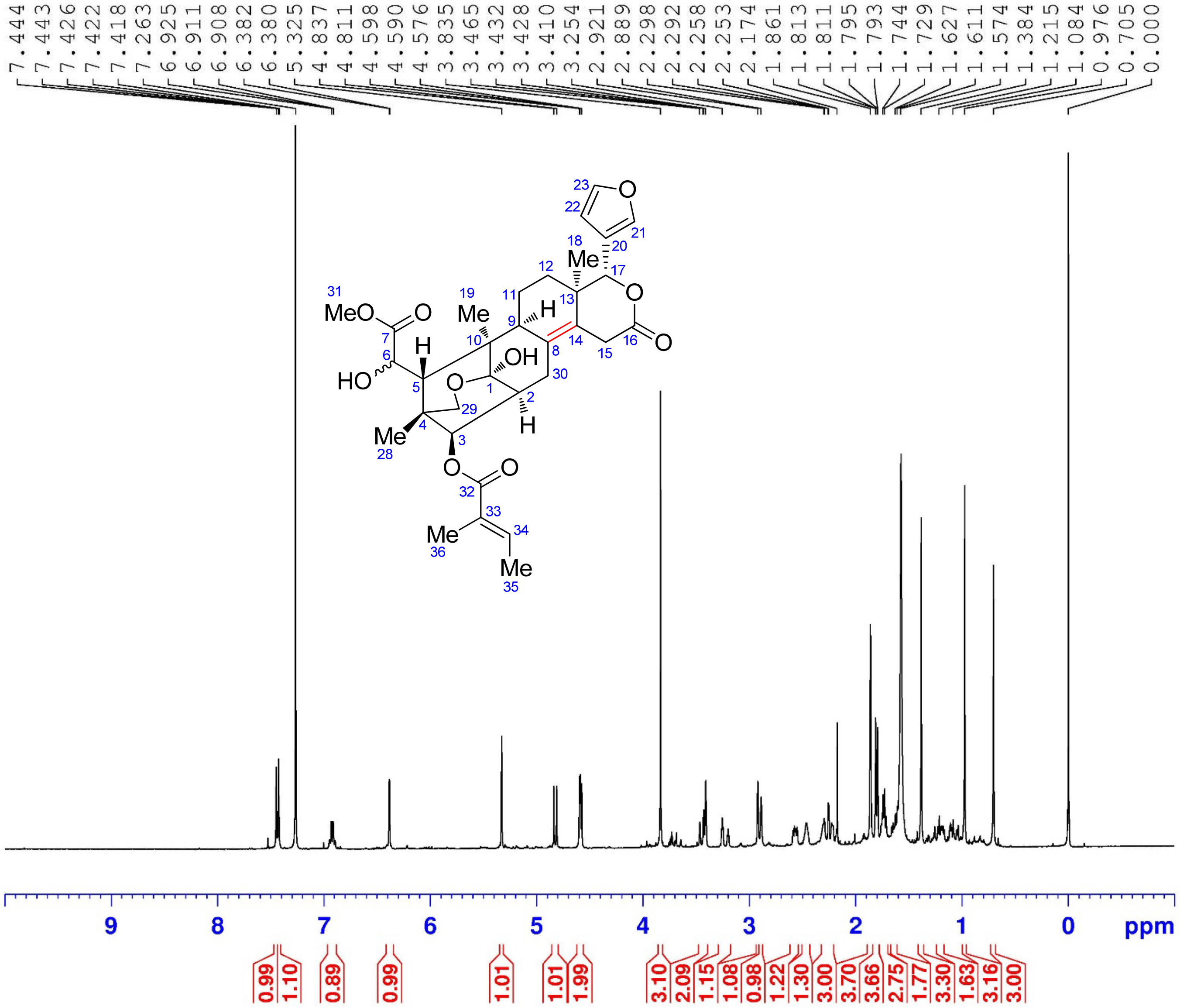


```
NAME            ZXP-R-17-2-1-2
EXPNO                        1
PROCNO                       1
Date_                 20180704
Time                      6.26
INSTRUM                  spect
PROBHD      5 mm CPPBBO BB
PULPROG                   zg30
TD                       65536
SOLVENT                  CDCl3
NS                          16
DS                           2
SWH                   8223.685 Hz
FIDRES                0.125483 Hz
AQ                   3.9846387 sec
RG                       208.5
DW                      60.800 usec
DE                       10.00 usec
TE                       297.0 K
D1                  1.00000000 sec
TD0                          1

======== CHANNEL f1 ========
SFO1               400.1324710 MHz
NUC1                        1H
P1                       11.50 usec
SI                       65536
SF                 400.1300088 MHz
WDW                         EM
SSB                          0
LB                        0.30 Hz
GB                           0
PC                        1.00
```

^{1}H NMR (400 MHz) spectrum of compound **4** in $CDCl_3$

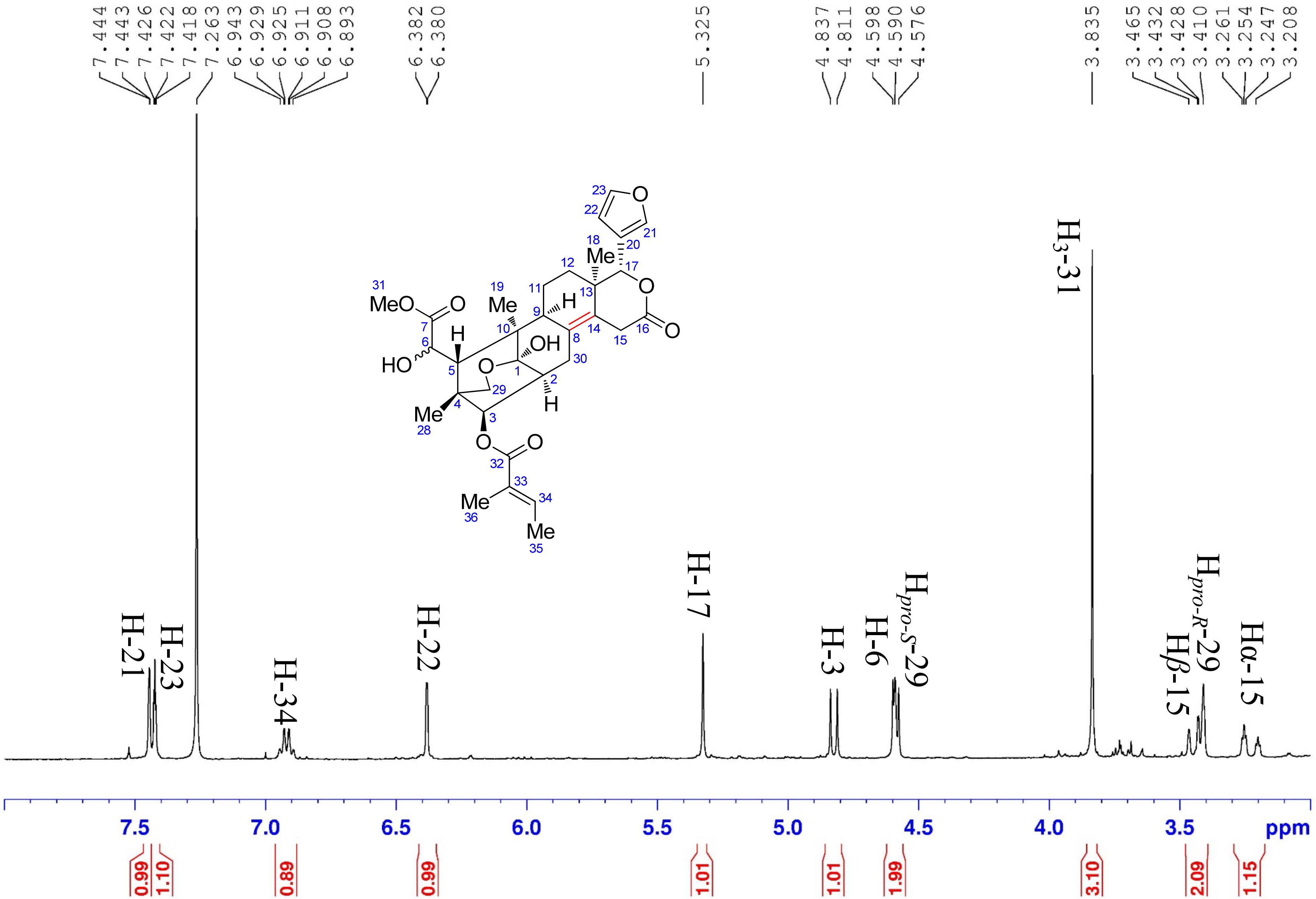

^{1}H NMR (400 MHz) spectrum of compound **4** in $CDCl_3$

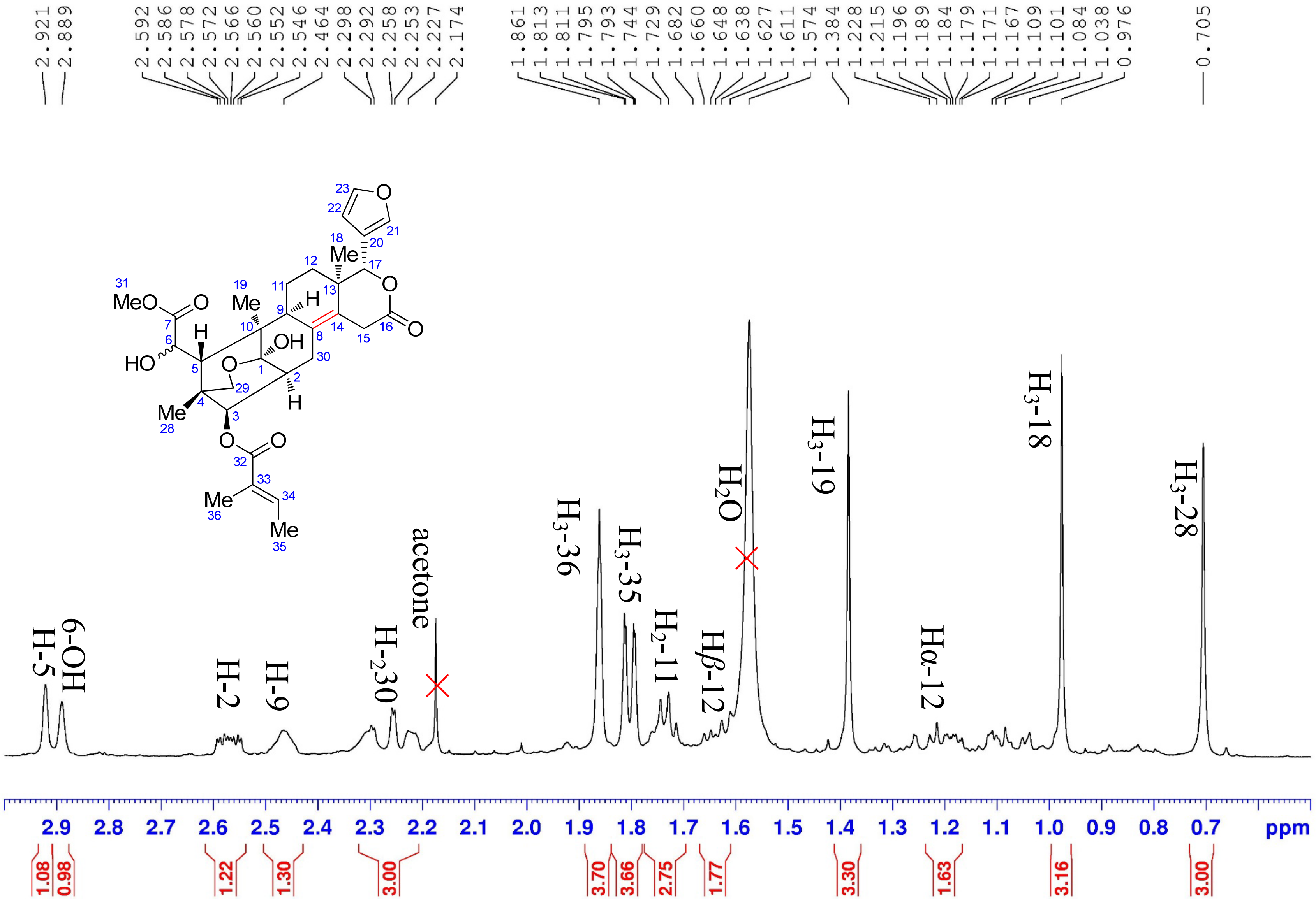

^{13}C NMR (100 MHz) spectrum of compound **4** in $CDCl_3$

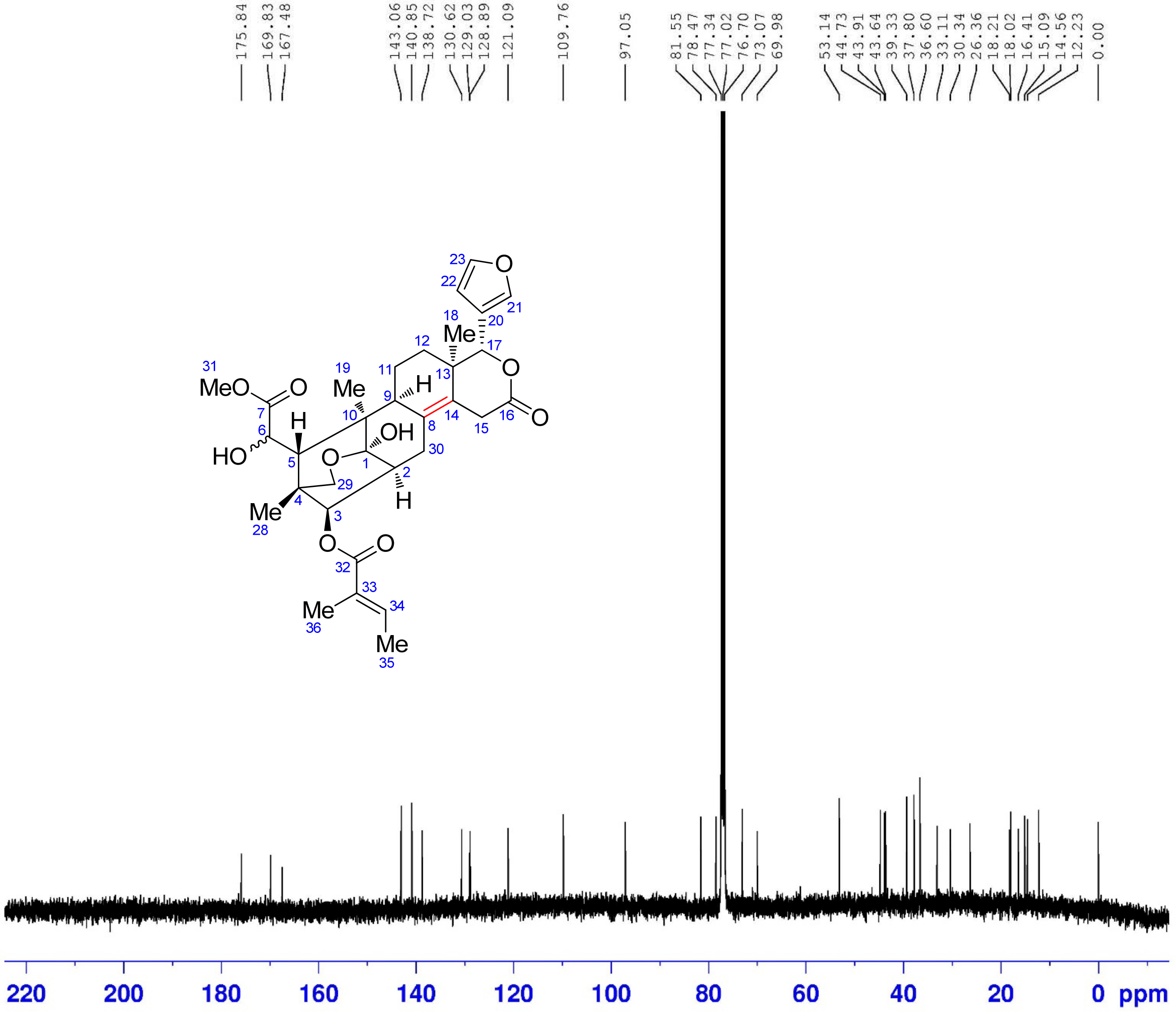

BRUKER

NAME	ZXP-R-17-2-1-2
EXPNO	2
PROCNO	1
Date_	20180704
Time	7.26
INSTRUM	spect
PROBHD	5 mm CPPBBO BB
PULPROG	zgpg30
TD	65536
SOLVENT	CDCl3
NS	1024
DS	4
SWH	24038.461 Hz
FIDRES	0.366798 Hz
AQ	1.3631988 sec
RG	147.94
DW	20.800 usec
DE	18.00 usec
TE	297.0 K
D1	2.00000000 sec
D11	0.03000000 sec
TD0	1

======== CHANNEL f1 ========

SFO1	100.6233324 MHz
NUC1	13C
P1	10.00 usec
SI	32768
SF	100.6127689 MHz
WDW	EM
SSB	0
LB	1.00 Hz
GB	0
PC	1.40

^{13}C NMR (100 MHz) spectrum of compound **4** in $CDCl_3$

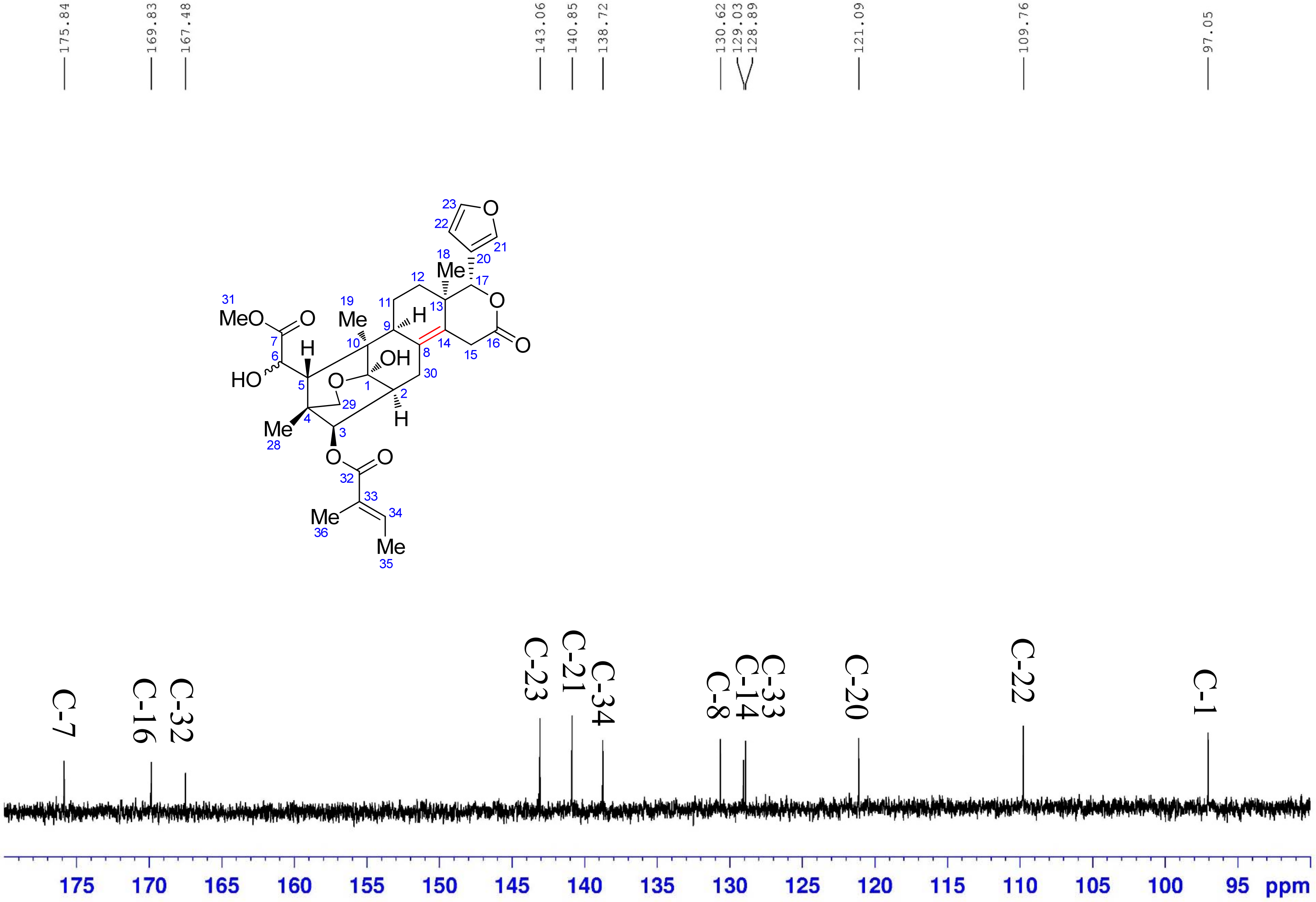

^{13}C NMR (100 MHz) spectrum of compound **4** in $CDCl_3$

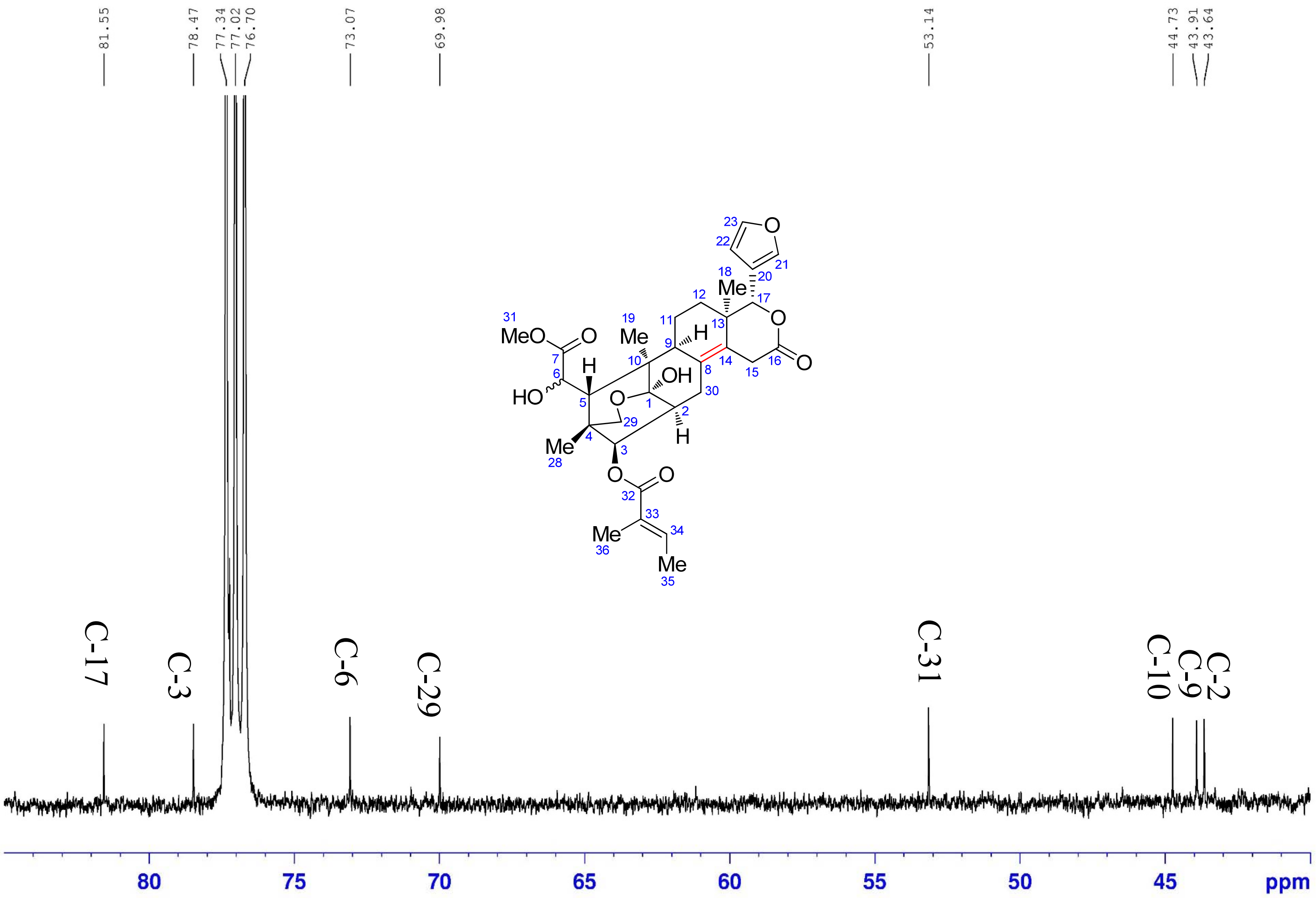

^{13}C NMR (100 MHz) spectrum of compound **4** in $CDCl_3$

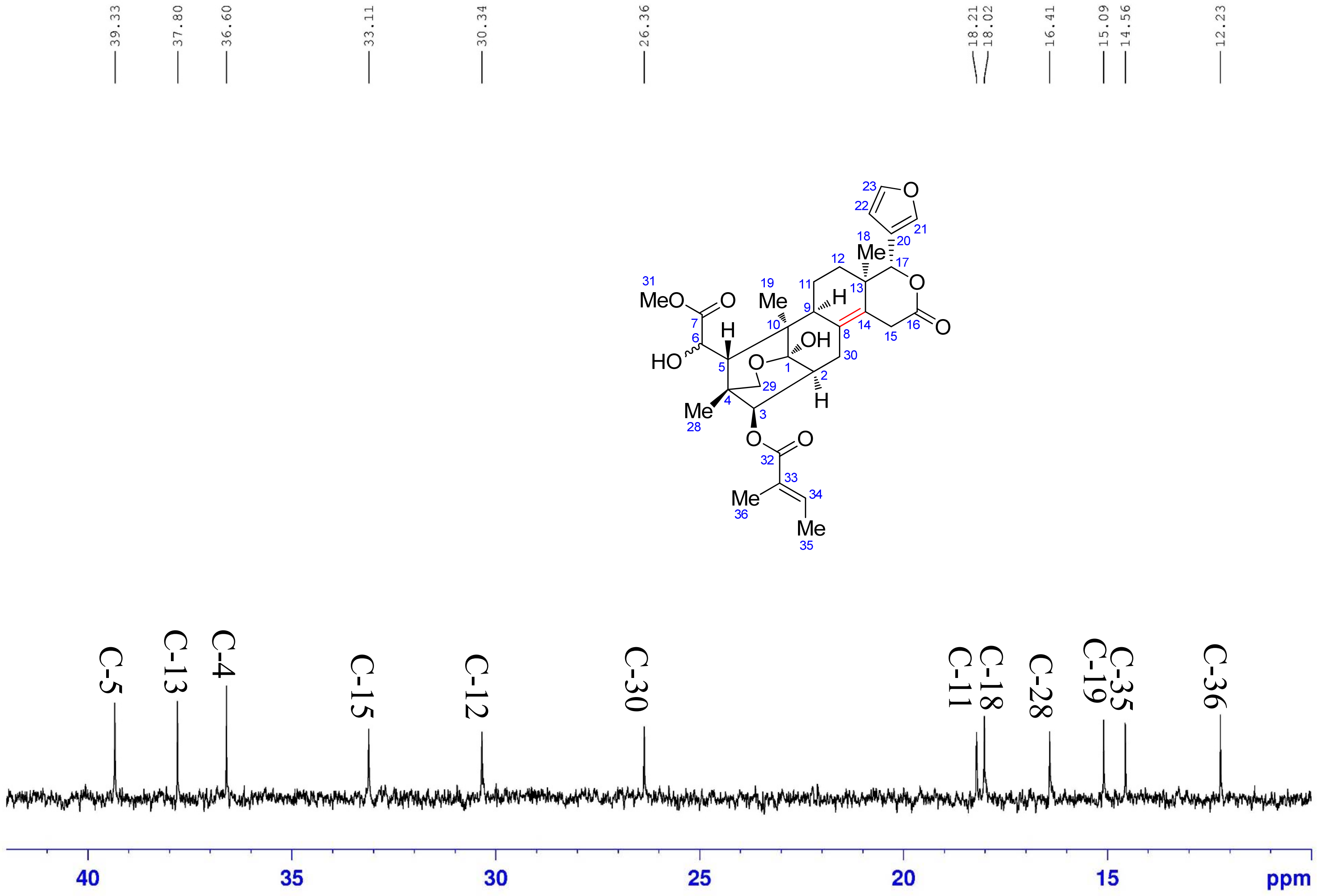

DEPT135 (100 MHz) spectrum of compound **4** in $CDCl_3$

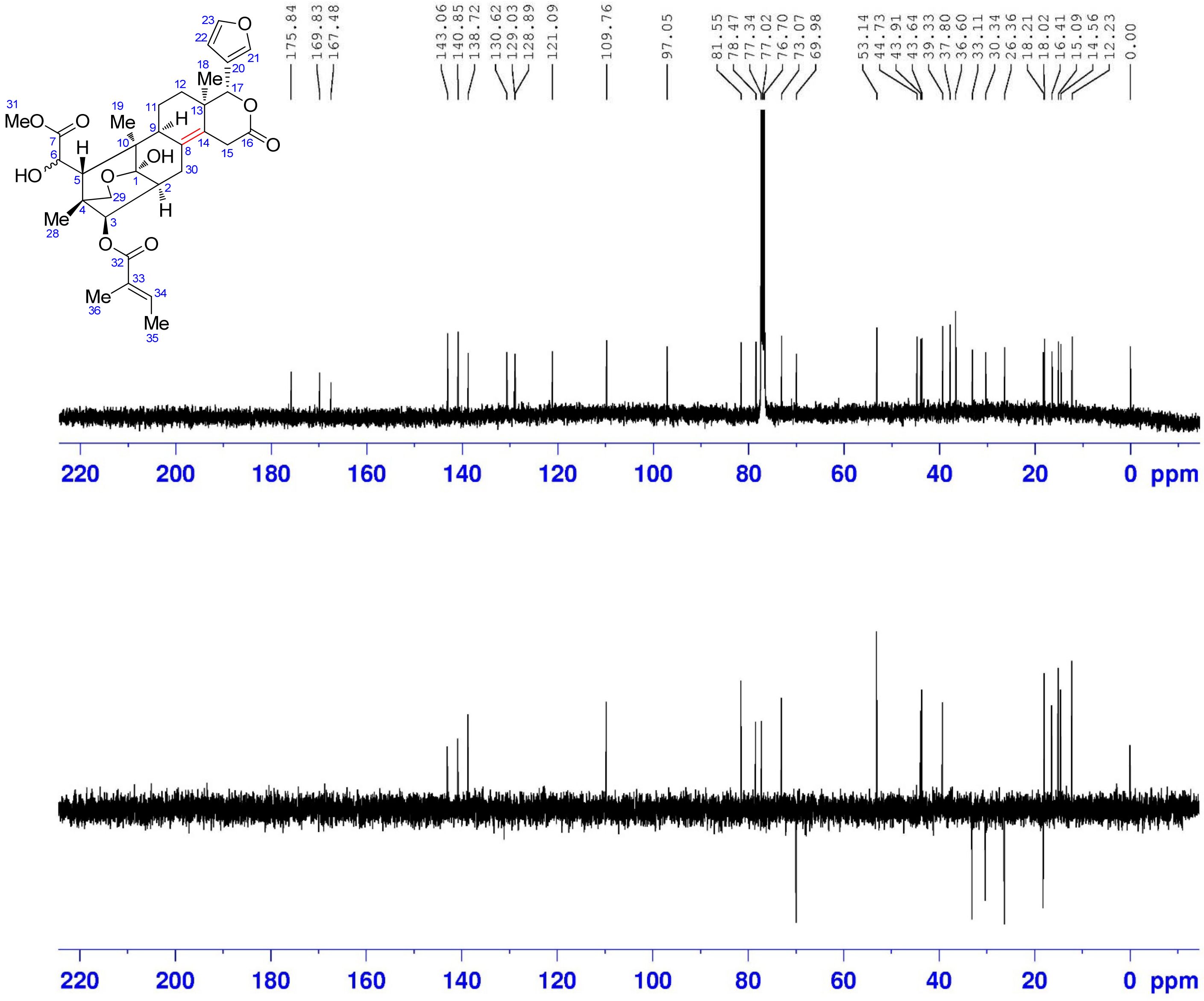

BRUKER

```
NAME       ZXP-R-17-2-1-2
EXPNO                   2
PROCNO                  1
Date_            20180704
Time                 7.26
INSTRUM             spect
PROBHD     5 mm CPPBBO BB
PULPROG           zgpg30
TD                 65536
SOLVENT            CDCl3
NS                  1024
DS                     4
SWH            24038.461 Hz
FIDRES          0.366798 Hz
AQ             1.3631988 sec
RG                147.94
DW                20.800 usec
DE                 18.00 usec
TE                 297.0 K
D1            2.00000000 sec
D11           0.03000000 sec
TD0                    1

======== CHANNEL f1 ========
SFO1         100.6233324 MHz
NUC1                 13C
P1                 10.00 usec
SI                 32768
SF           100.6127689 MHz
WDW                   EM
SSB                    0
LB                  1.00 Hz
GB                     0
PC                  1.40
```

DEPT135 (100 MHz) spectrum of compound **4** in $CDCl_3$

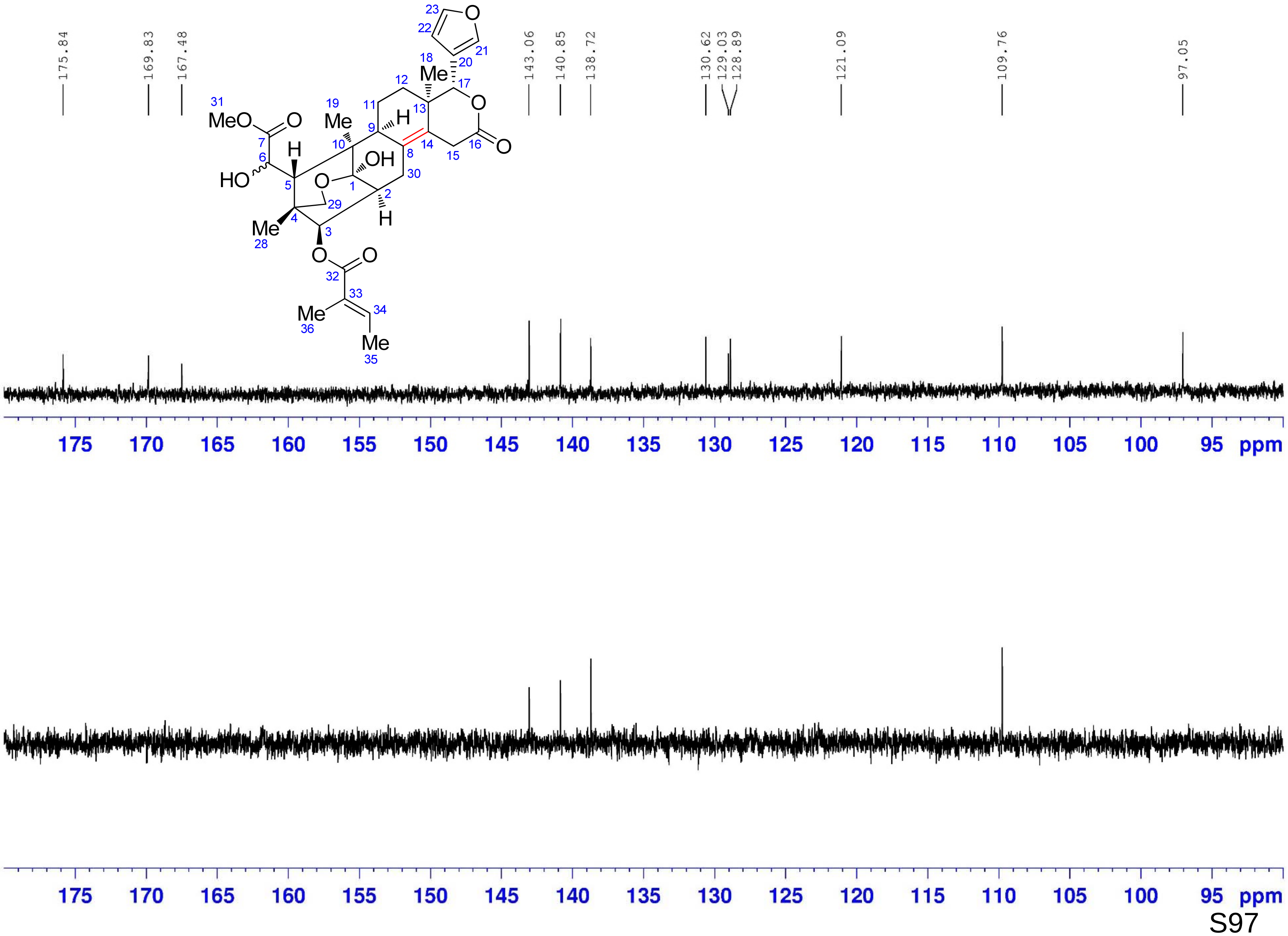

DEPT135 (100 MHz) spectrum of compound **4** in $CDCl_3$

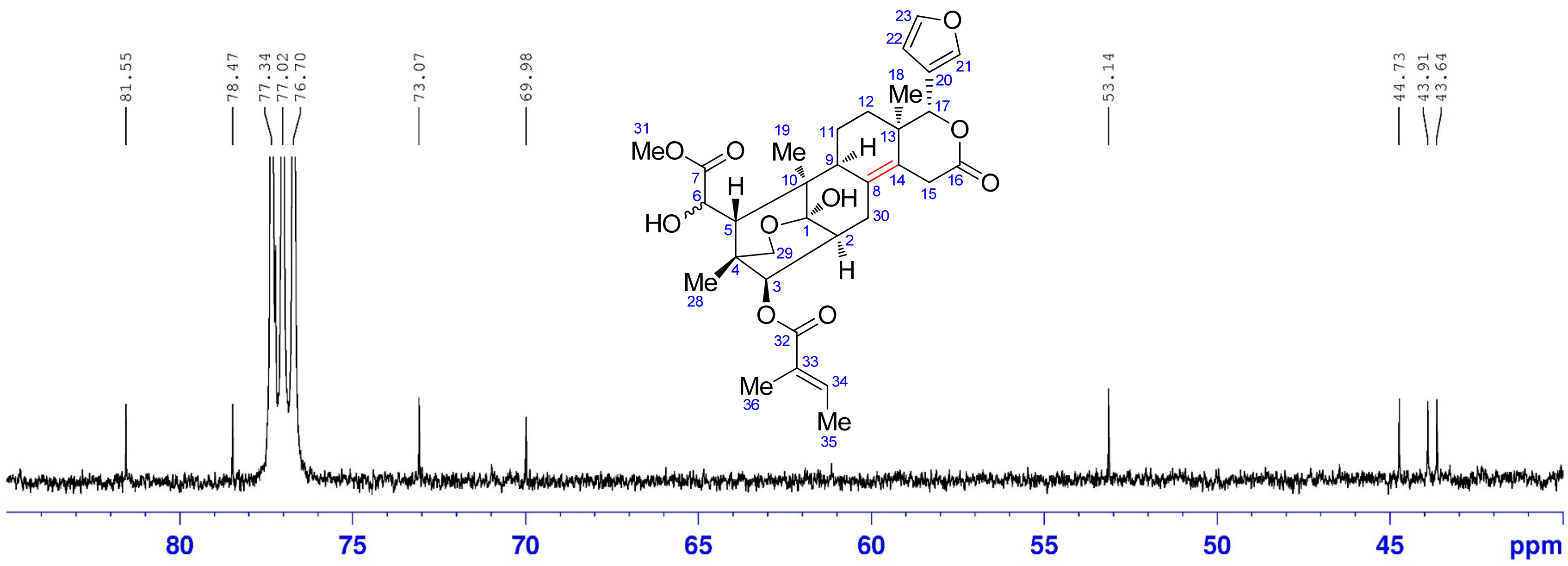

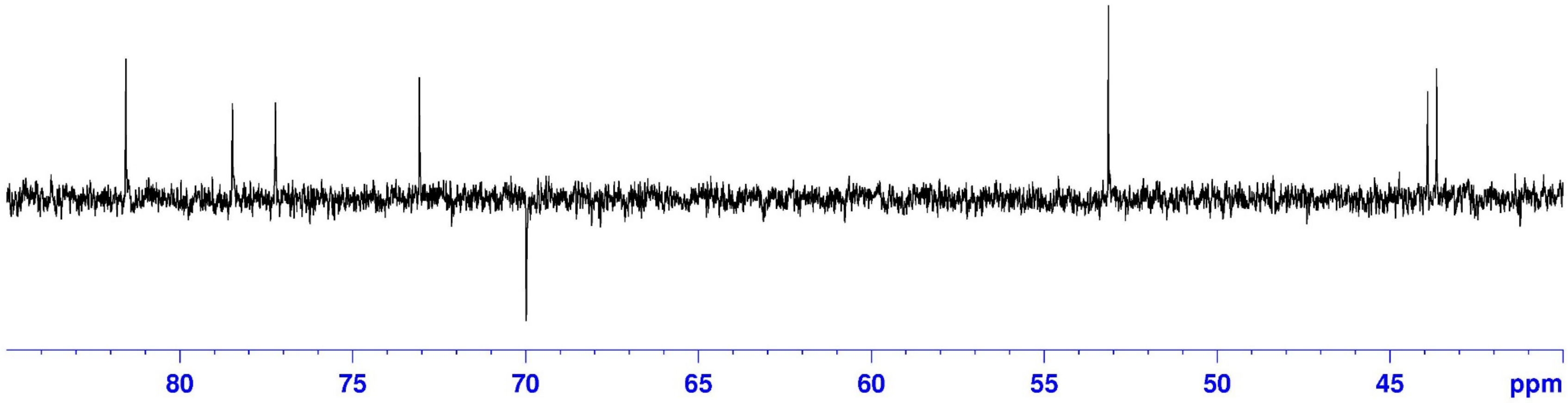

DEPT135 (100 MHz) spectrum of compound **4** in $CDCl_3$

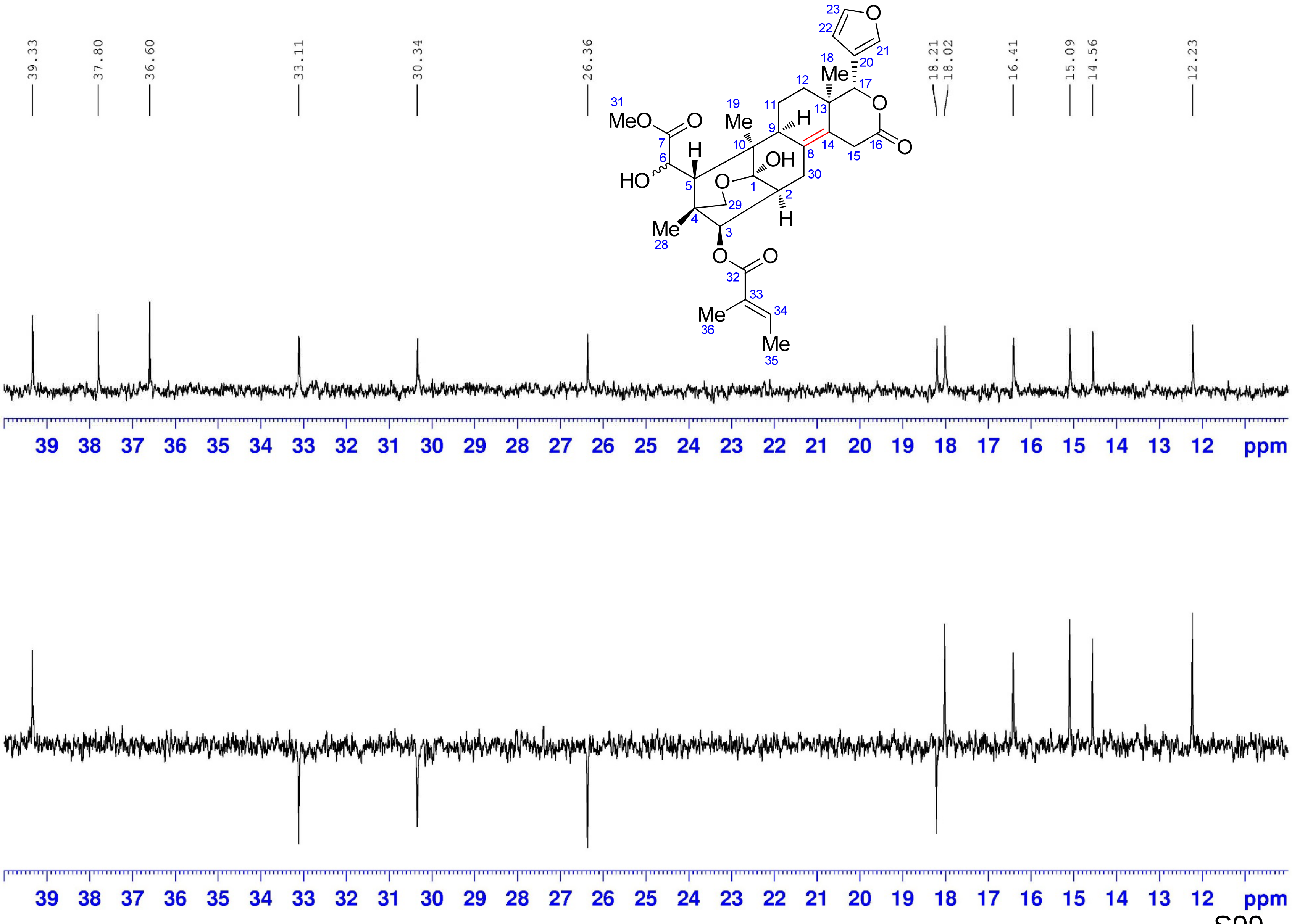

^{1}H-^{1}H COSY (400 MHz) spectrum of compound **4** in $CDCl_3$

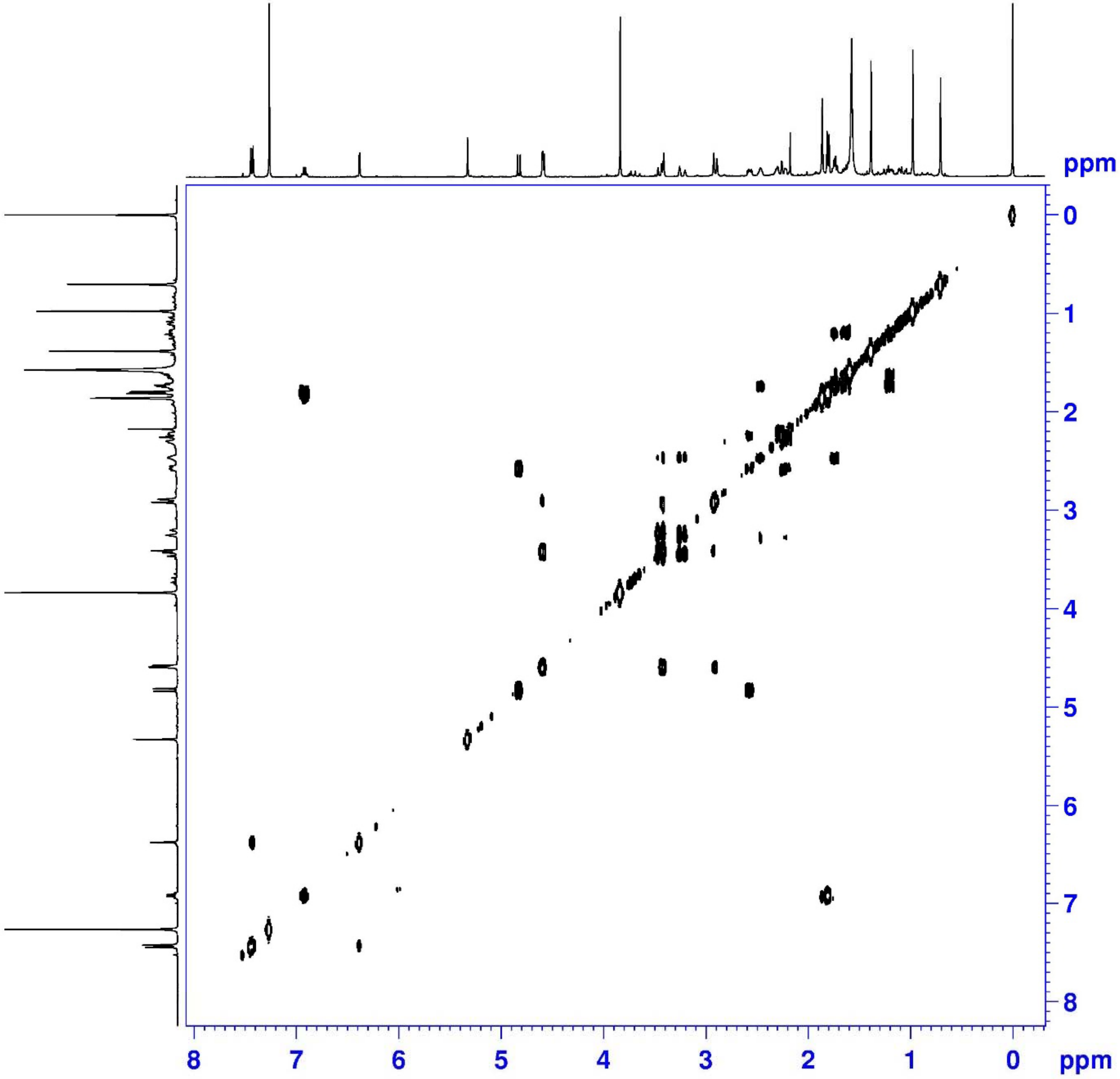

NAME	ZXP-R-17-2-1-2	
EXPNO	4	
PROCNO	1	
Date_	20180707	
Time	17.34	
INSTRUM	spect	
PROBHD	5 mm CPPBBO BB	
PULPROG	cosygpppqf	
TD	2048	
SOLVENT	CDCl3	
NS	8	
DS	8	
SWH	3906.250	Hz
FIDRES	1.907349	Hz
AQ	0.2621940	sec
RG	208.5	
DW	128.000	usec
DE	10.00	usec
TE	297.0	K
D0	0.00000300	sec
D1	1.89678097	sec
D11	0.03000000	sec
D12	0.00002000	sec
D13	0.00000400	sec
D16	0.00020000	sec
IN0	0.00025600	sec
======== CHANNEL f1 ========		
SFO1	400.1318006	MHz
NUC1	1H	
P0	11.50	usec
P1	11.50	usec
P17	2500.00	usec
ND0	1	
TD	128	
SFO1	400.1318	MHz
FIDRES	30.517578	Hz
SW	9.762	ppm
FnMODE	QF	
SI	1024	
SF	400.1300060	MHz
WDW	QSINE	
SSB	0	
LB	0.00	Hz
GB	0	
PC	1.40	
SI	1024	
MC2	QF	
SF	400.1300049	MHz
WDW	QSINE	
SSB	0	
LB	0.00	Hz
GB	0	

^{1}H-^{1}H COSY (400 MHz) spectrum of compound **4** in $CDCl_3$

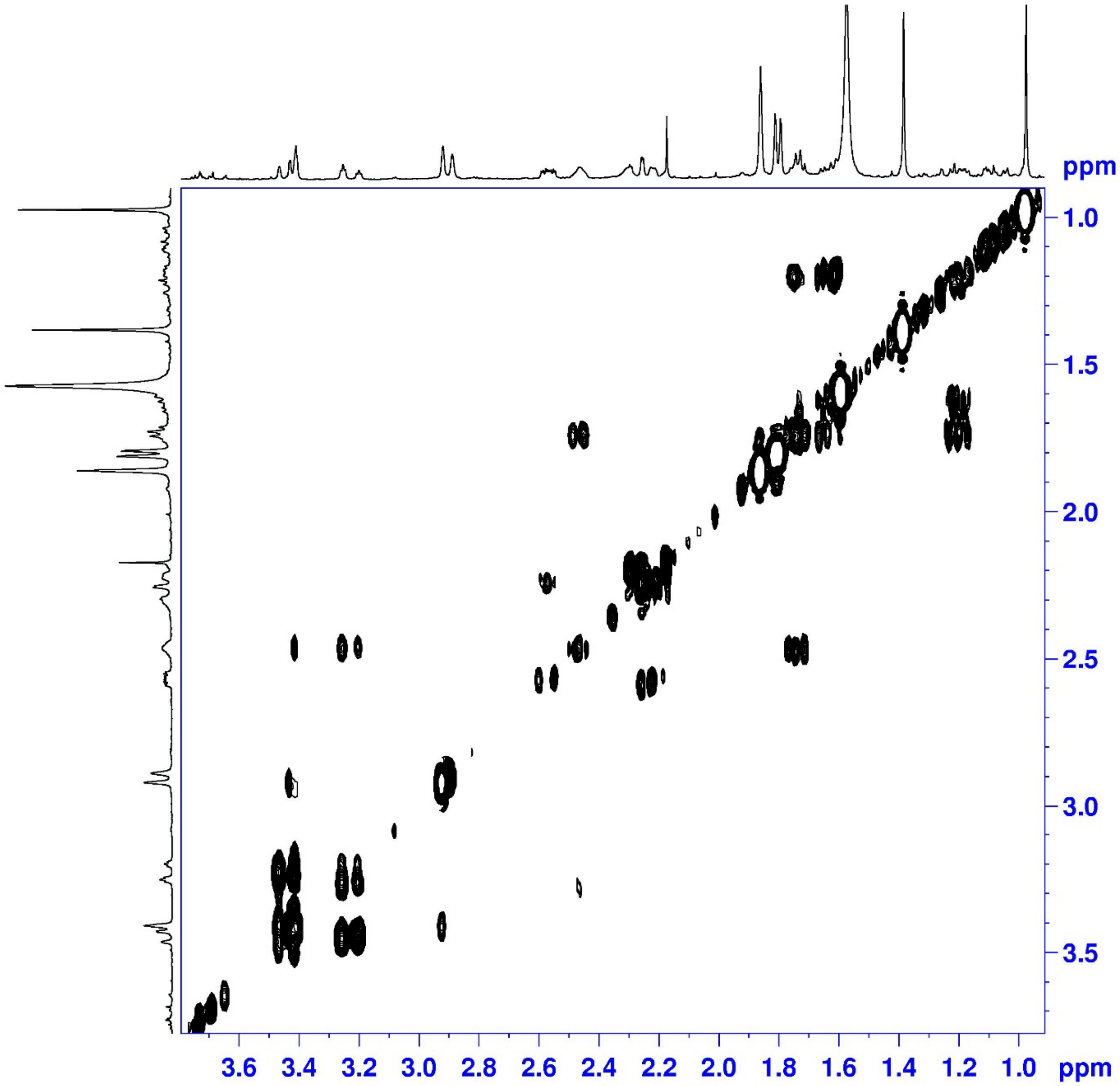

HSQC (400 MHz) spectrum of compound **4** in $CDCl_3$

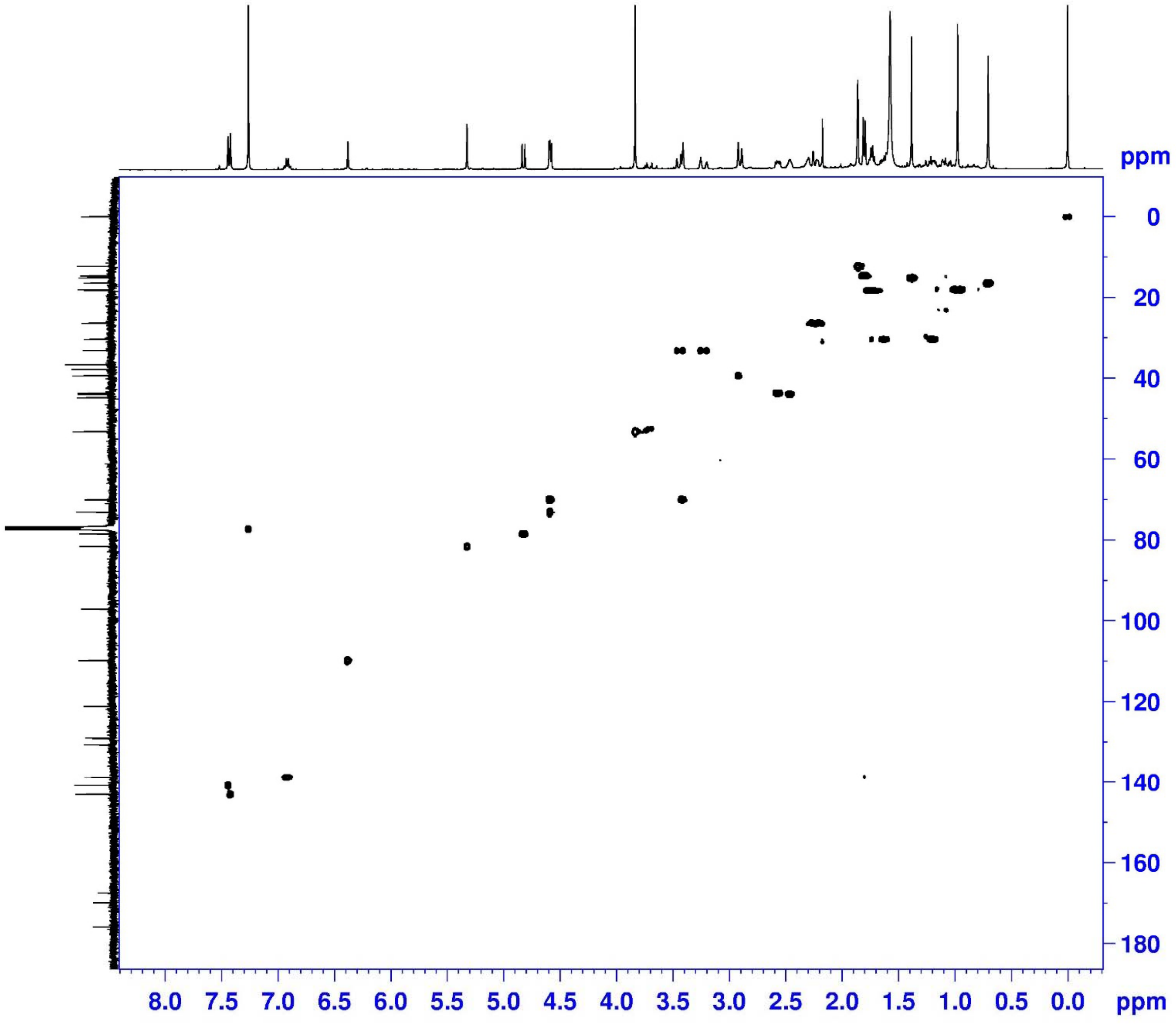


```
NAME      ZXP-R-17-2-1-2
EXPNO                  5
PROCNO                 1
Date_           20180707
Time               18.14
INSTRUM            spect
PROBHD   5 mm CPPBBO BB
PULPROG      hsqcetgpsi2
TD                  1024
SOLVENT            CDCl3
NS                    16
DS                    16
SWH             4302.926 Hz
FIDRES          4.202076 Hz
AQ             0.1190388 sec
RG                 208.5
DW               116.200 usec
DE                 10.00 usec
TE                 297.0 K
CNST2        145.0000000
D0            0.00000300 sec
D1            1.46497905 sec
D4            0.00172414 sec
D11           0.03000000 sec
D16           0.00020000 sec
D24           0.00086207 sec
IN0           0.00002080 sec
ZGOPTNS

======== CHANNEL f1 ========
SFO1         400.1320007 MHz
NUC1                  1H
P1                 11.50 usec
P2                 23.00 usec
P28                 0.00 usec
ND0                    2
TD                   256
SFO1            100.6233 MHz
FIDRES         93.900238 Hz
SW               238.896 ppm
FnMODE       Echo-Antiecho
SI                  1024
SF           400.1300068 MHz
WDW                QSINE
SSB                    2
LB                  0.00 Hz
GB                     0
PC                  1.40
SI                  1024
MC2          echo-antiecho
SF           100.6127562 MHz
WDW                QSINE
SSB                    2
LB                  0.00 Hz
GB                     0
```

HSQC (400 MHz) spectrum of compound **4** in $CDCl_3$

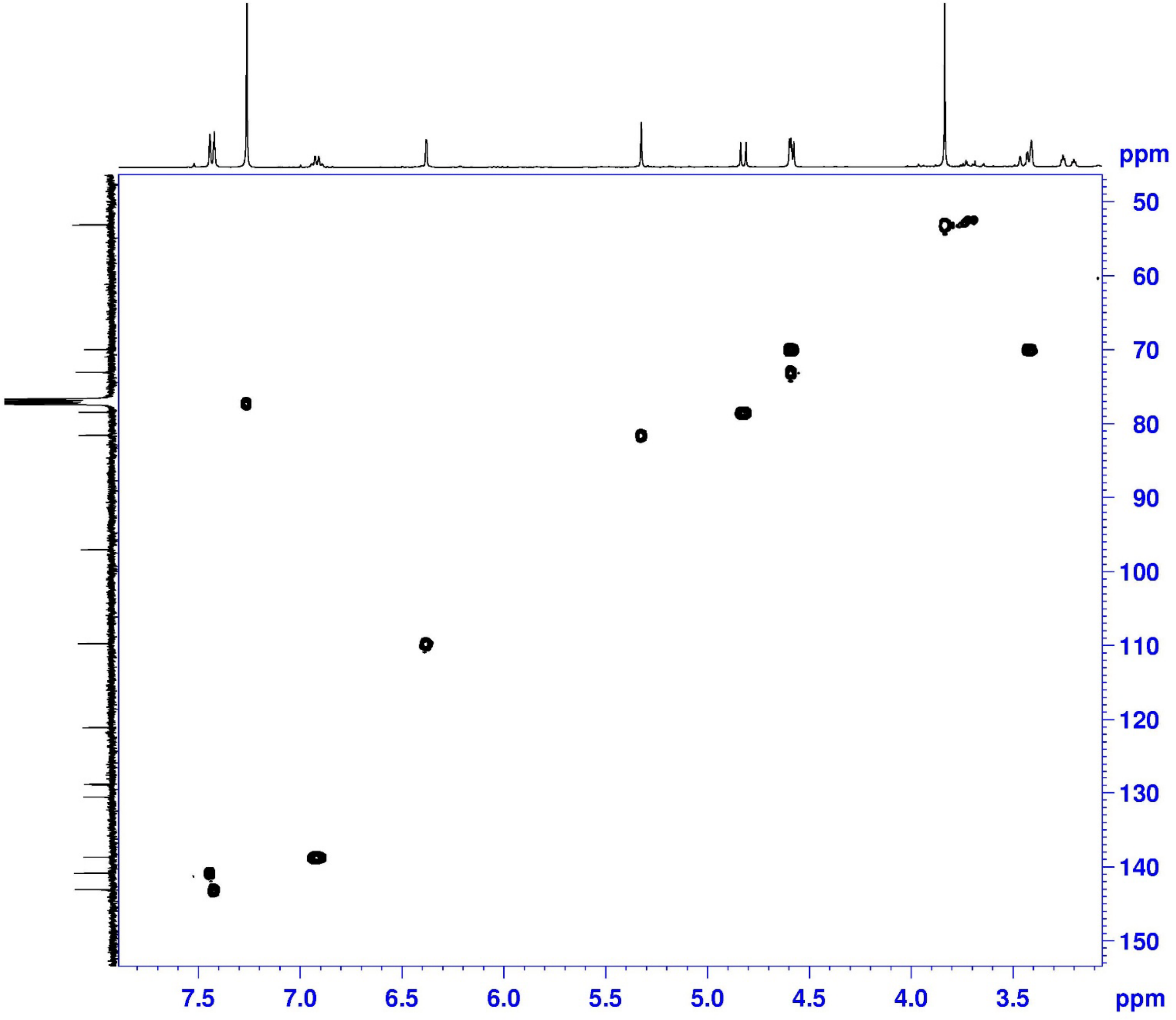

HSQC (400 MHz) spectrum of compound **4** in $CDCl_3$

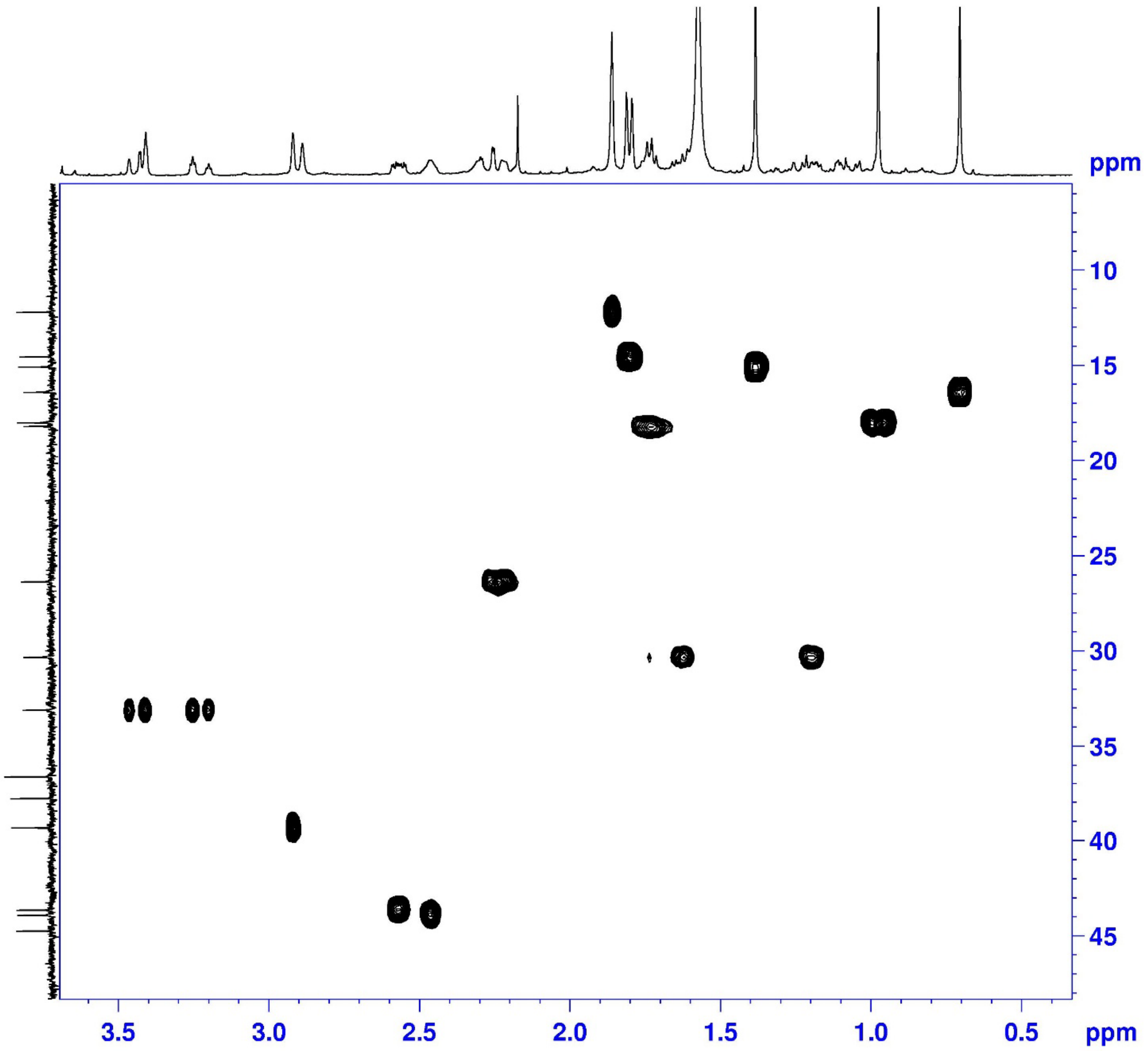

HMBC (400 MHz) spectrum of compound **4** in $CDCl_3$

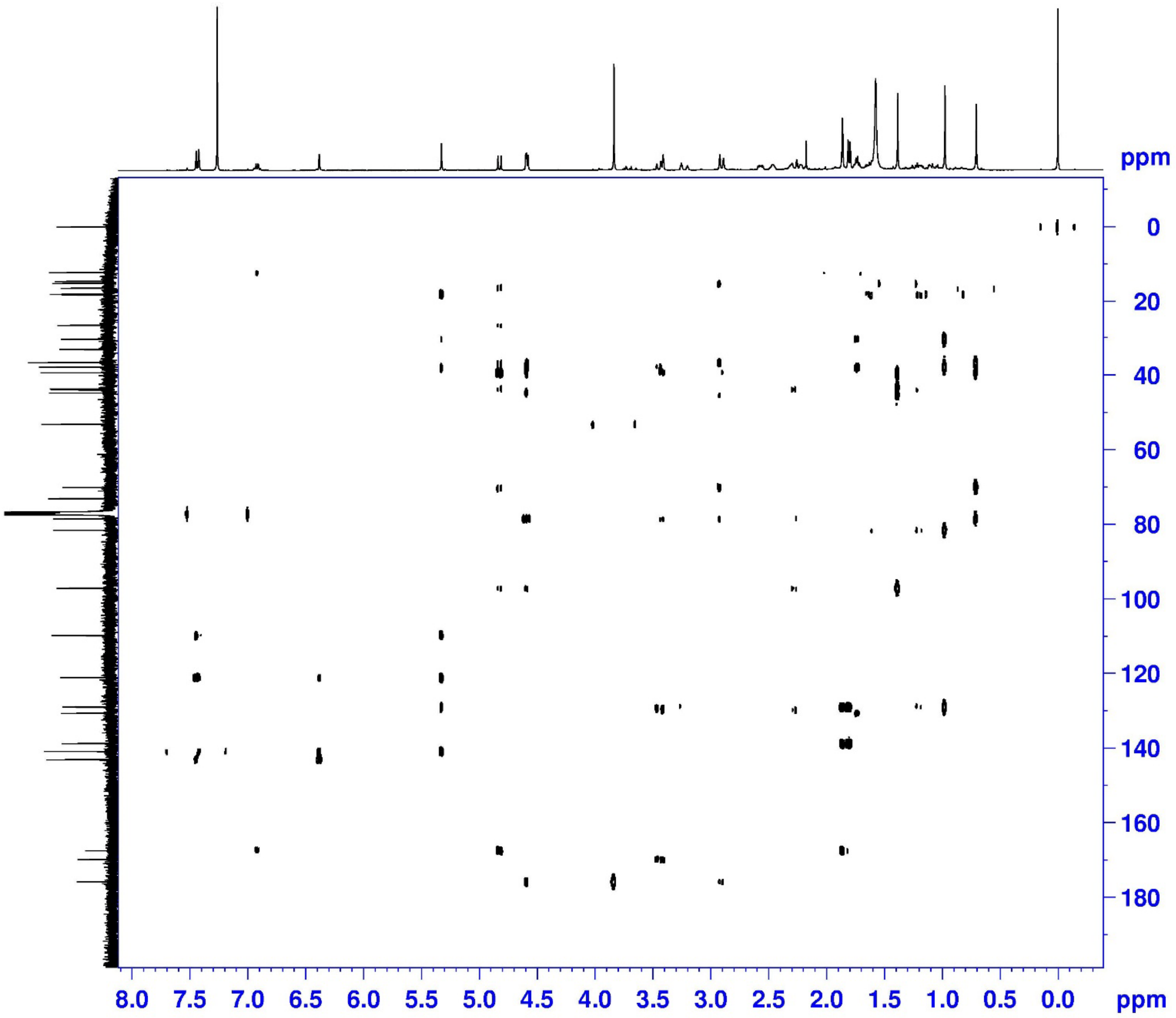

HMBC (400 MHz) spectrum of compound **4** in $CDCl_3$

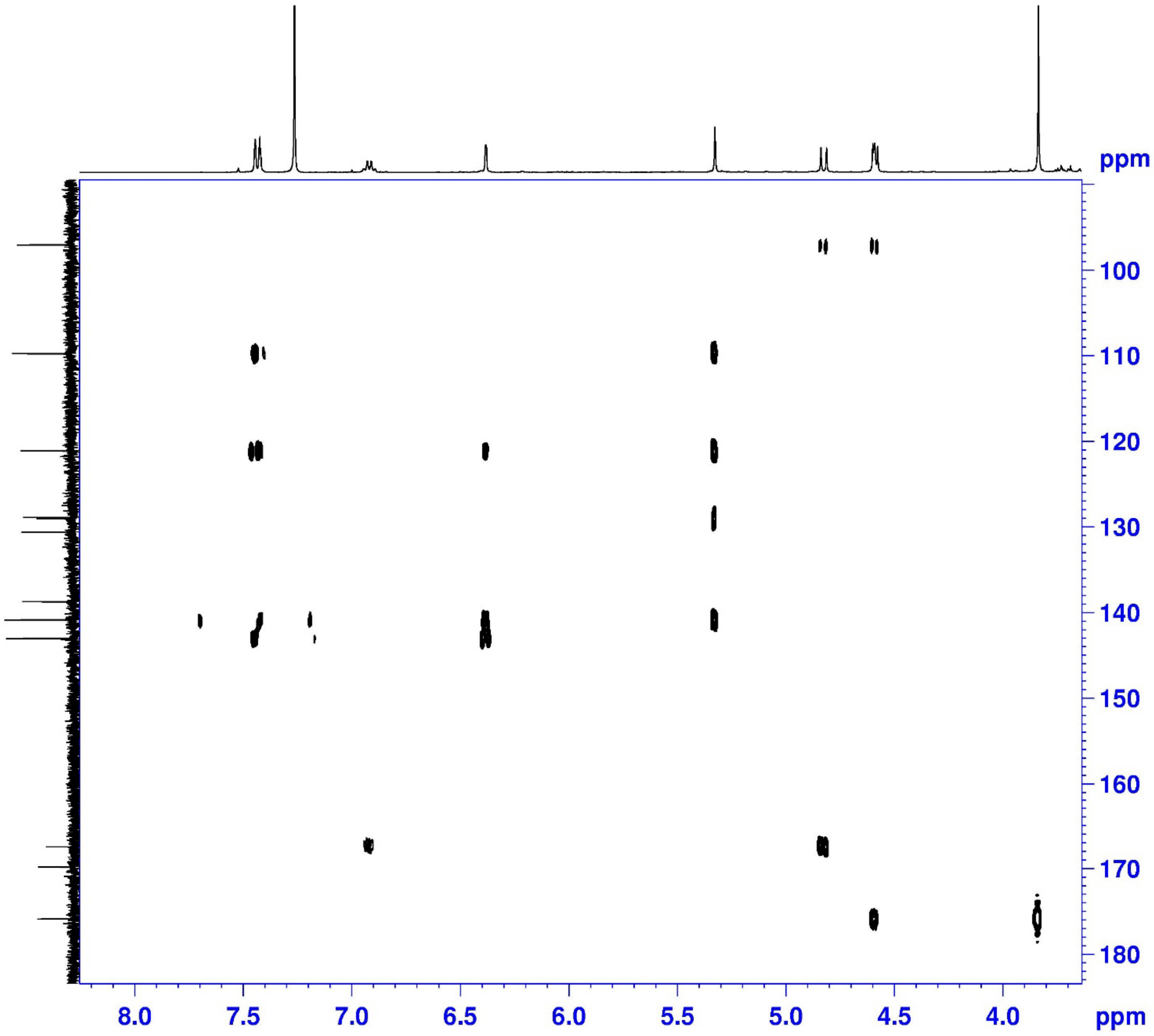

HMBC (400 MHz) spectrum of compound **4** in $CDCl_3$

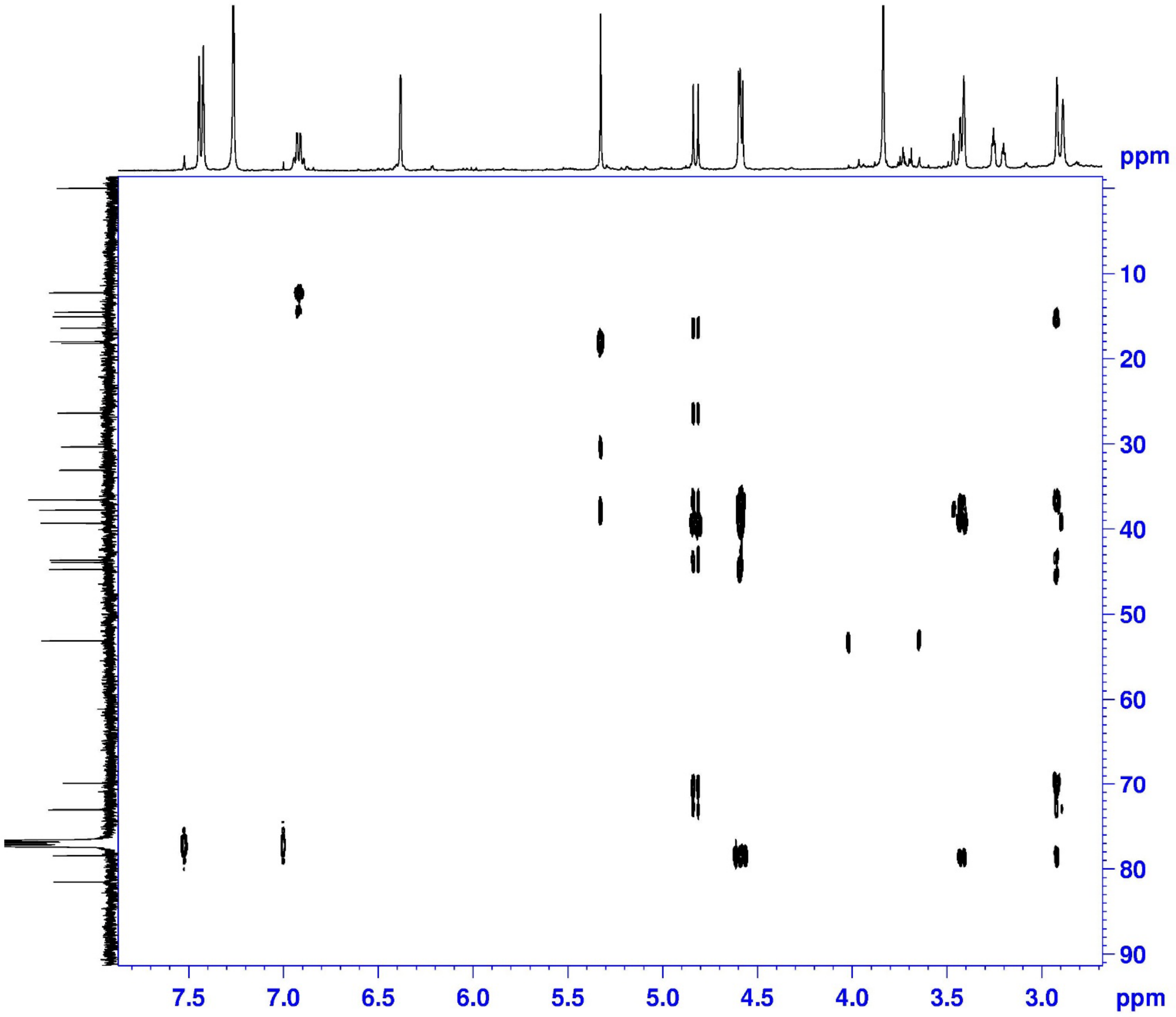

HMBC (400 MHz) spectrum of compound **4** in $CDCl_3$

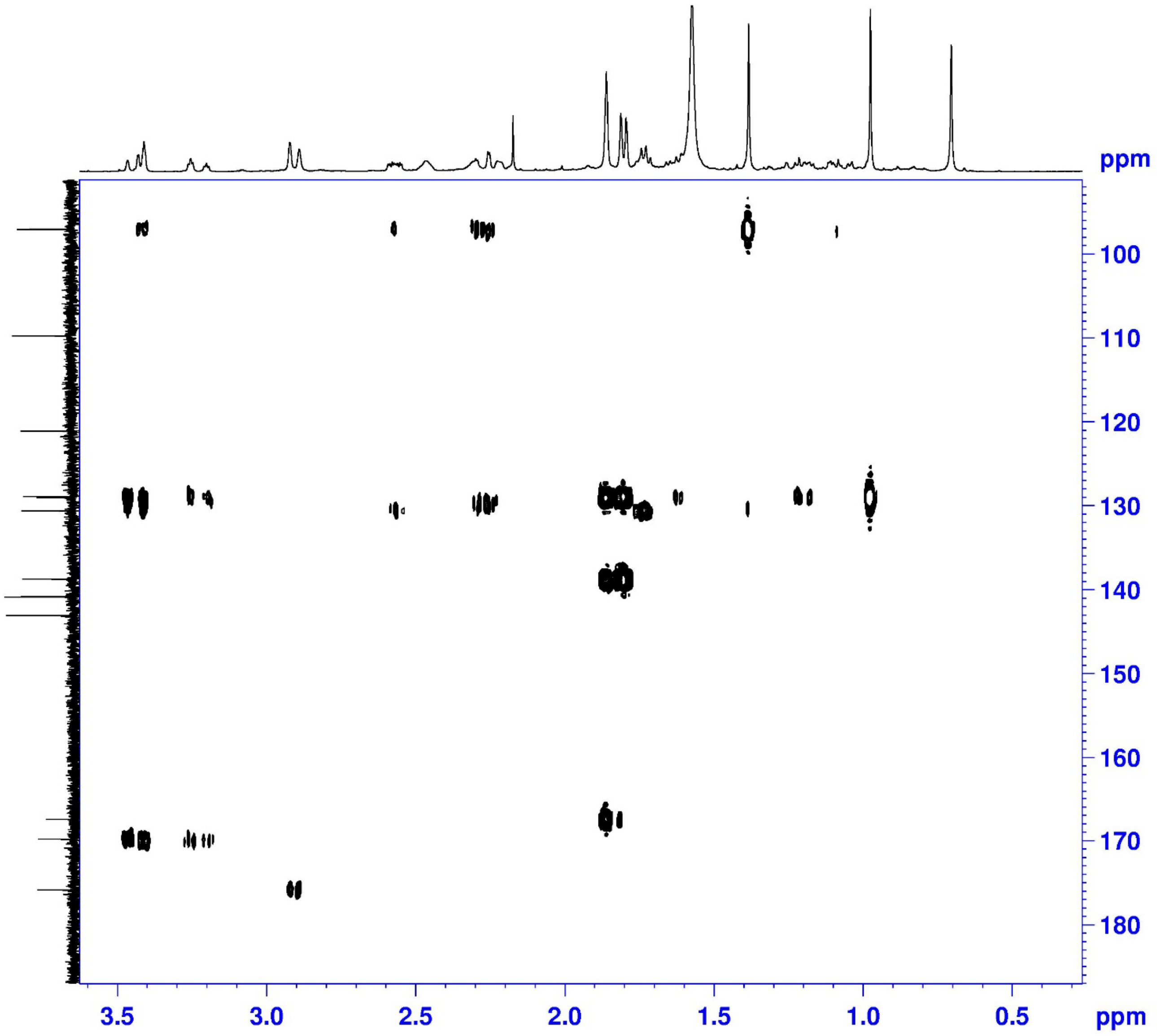

HMBC (400 MHz) spectrum of compound **4** in $CDCl_3$

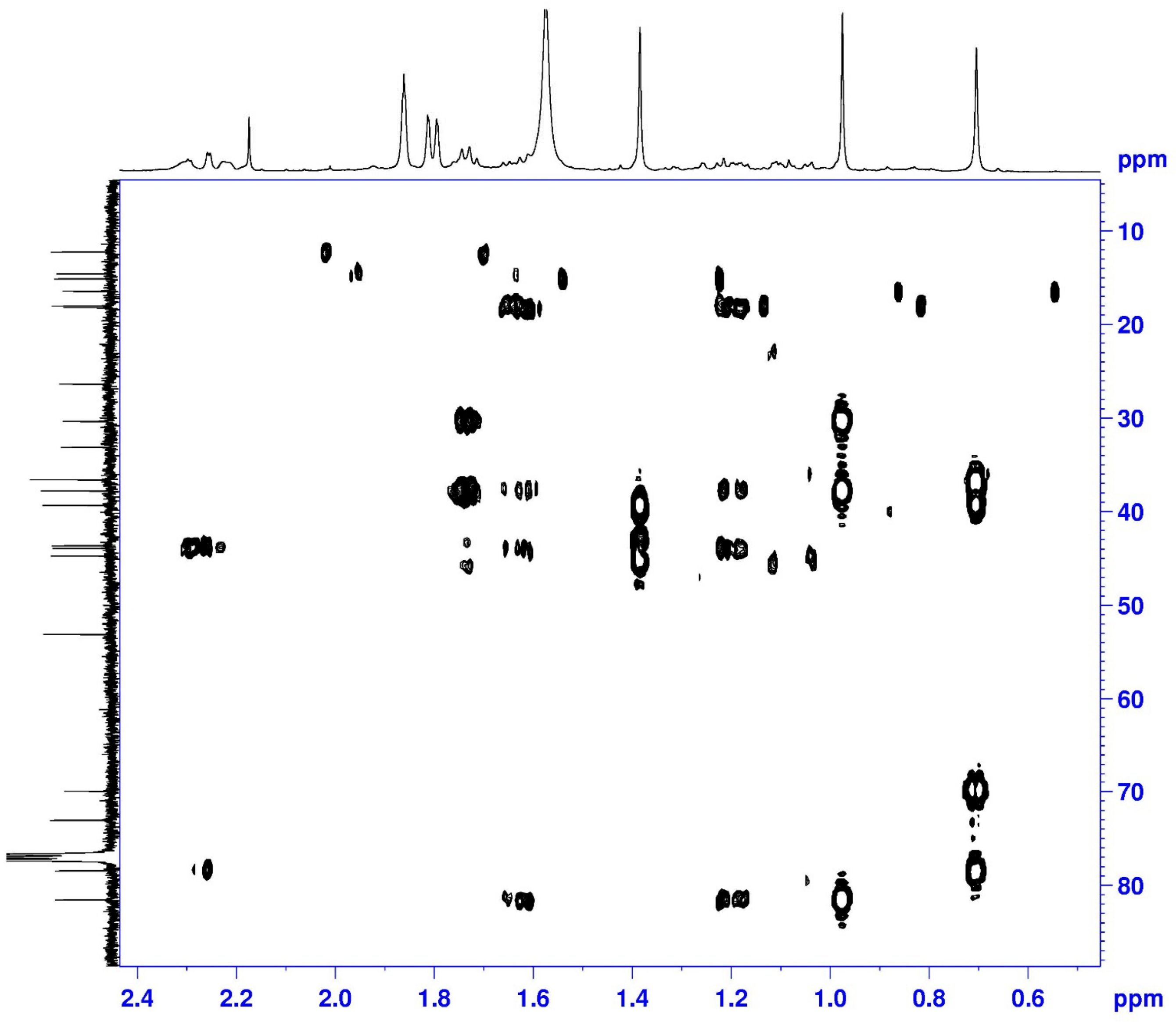

NOESY (400 MHz) spectrum of compound **4** in $CDCl_3$

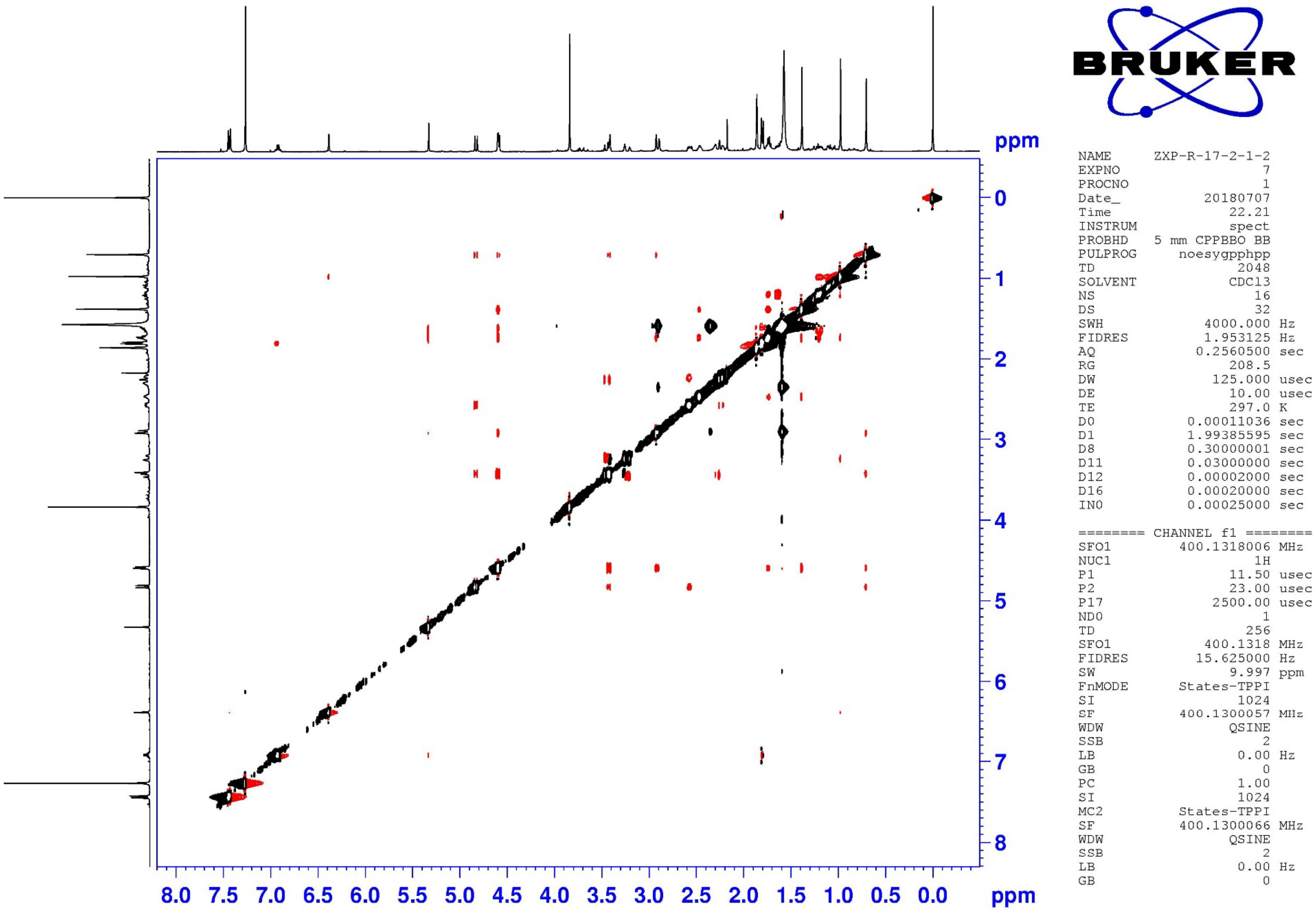

NOESY (400 MHz) spectrum of compound **4** in $CDCl_3$

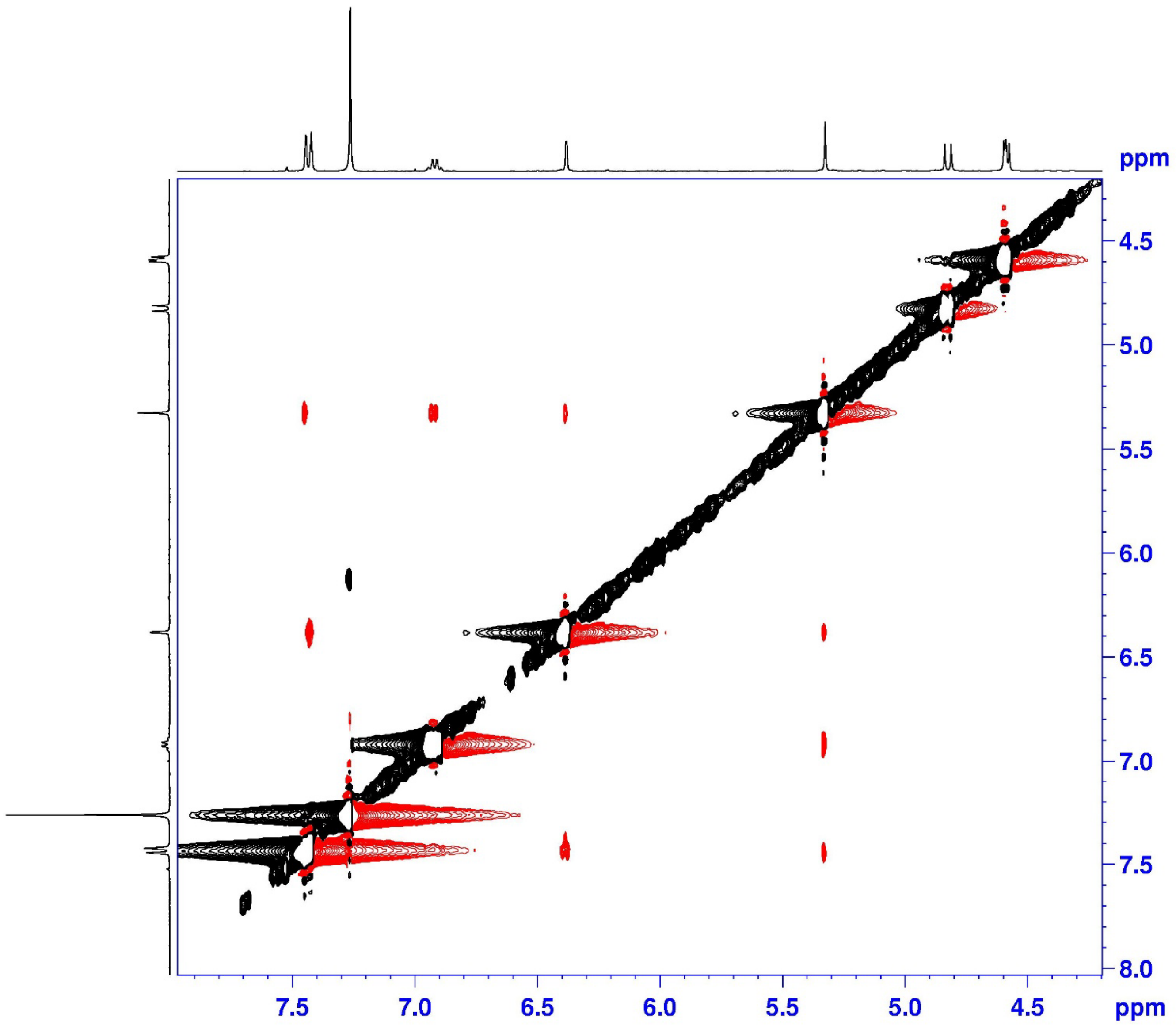

NOESY (400 MHz) spectrum of compound **4** in $CDCl_3$

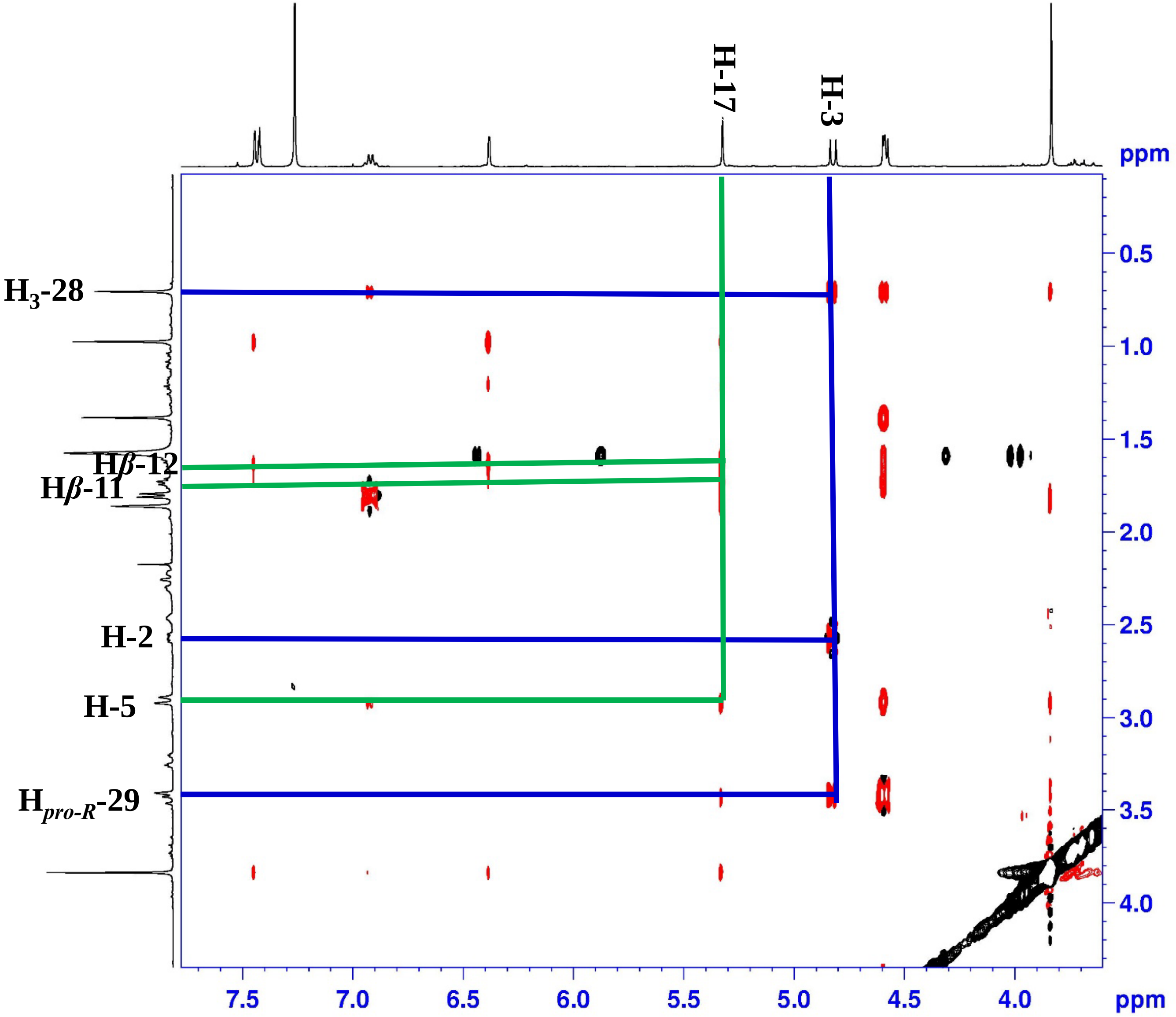

NOESY (400 MHz) spectrum of compound **4** in $CDCl_3$

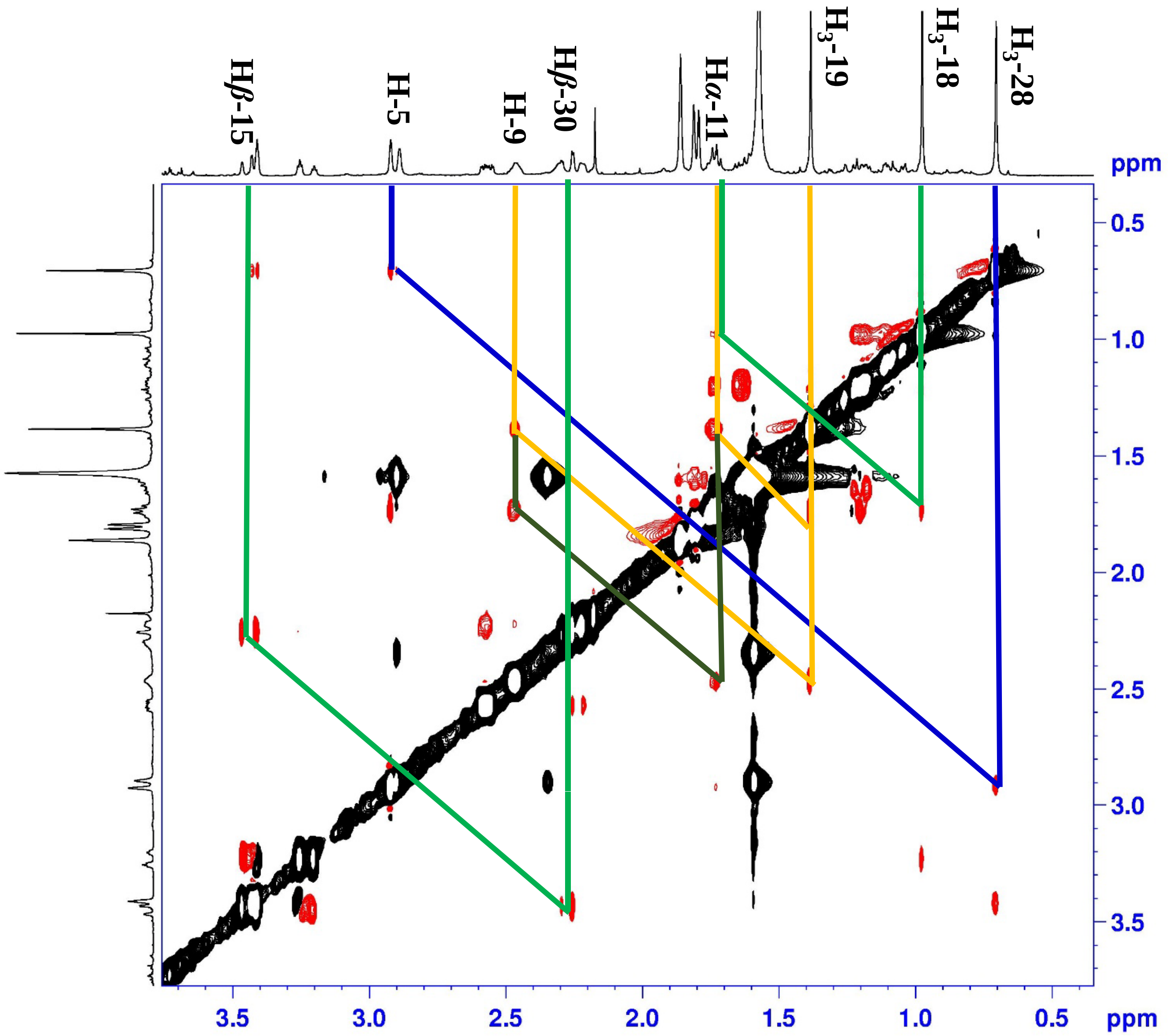

HR-ESIMS for compound **5**

Generic Display Report

Analysis Info

Analysis Name	D:\Data\MS\data\201805\liwanshan_P-18-2-1_pos_37_01_4667.d	Acquisition Date	5/4/2018 11:44:10 AM
Method	LC_Direct Infusion_pos_100-1000mz.m	Operator	SCSIO
Sample Name	liwanshan_P-18-2-1_pos	Instrument	maXis
Comment			

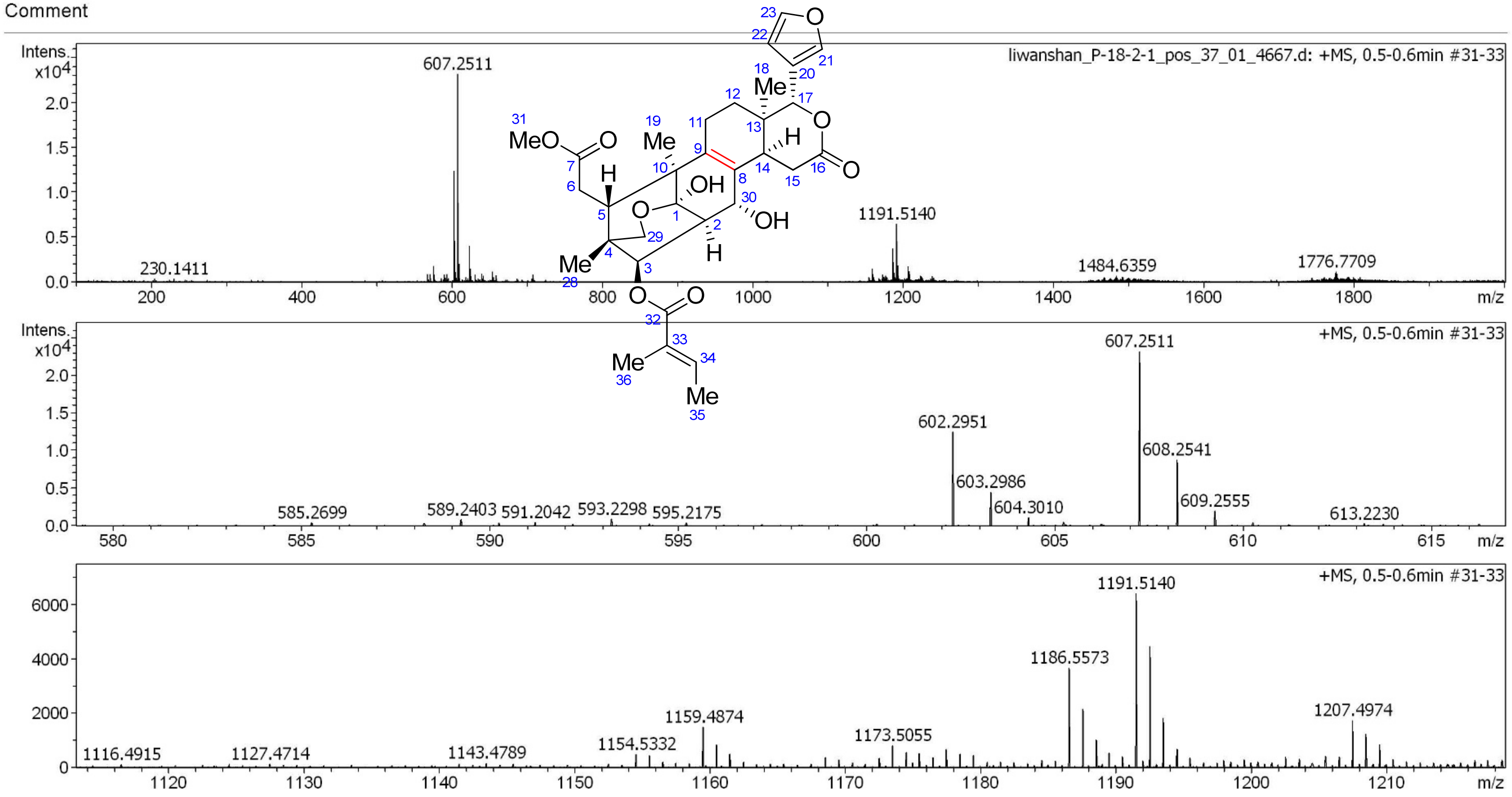

HR-ESIMS for compound **5**

Mass Spectrum SmartFormula Report

Analysis Info

Analysis Name	D:\Data\MS\data\201805\liwanshan_P-18-2-1_pos_37_01_4667.d	Acquisition Date	5/4/2018 11:44:10 AM	
Method	LC_Direct Infusion_pos_100-1000mz.m	Operator	SCSIO	
Sample Name	liwanshan_P-18-2-1_pos	Instrument	maXis	255552.00029
Comment				

Acquisition Parameter

Source Type	ESI	Ion Polarity	Positive	Set Nebulizer	0.4 Bar
Focus	Active	Set Capillary	4500 V	Set Dry Heater	180 °C
Scan Begin	100 m/z	Set End Plate Offset	-500 V	Set Dry Gas	4.0 l/min
Scan End	2000 m/z	Set Charging Voltage	0 V	Set Divert Valve	Waste
		Set Corona	0 nA	Set APCI Heater	0 °C

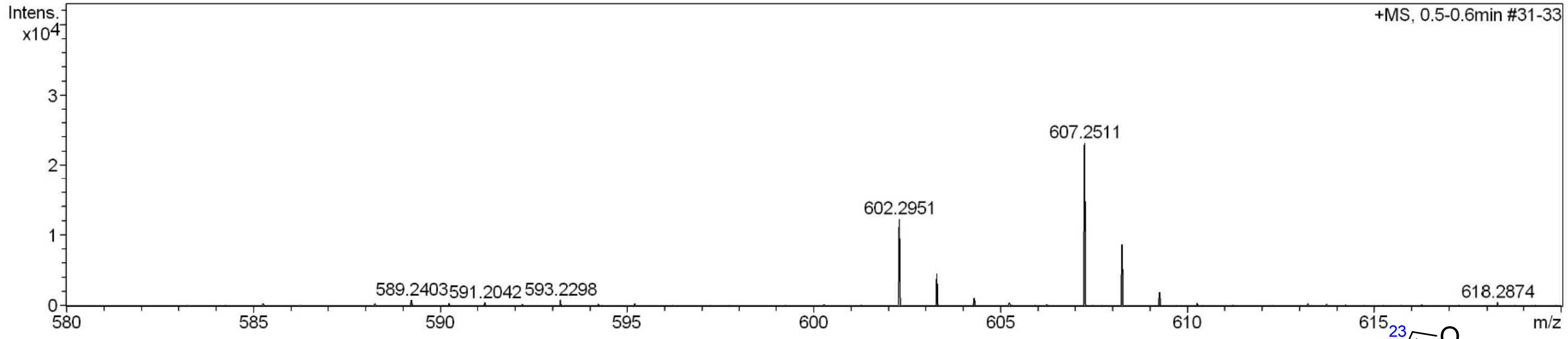

Meas. m/z	#	Ion Formula	Score	m/z	err [ppm]	err [mDa]	mSigma	rdb	e⁻ Conf	N-Rule
585.2699	1	C32H41O10	100.00	585.2694	-0.9	-0.5	371.8	12.5	even	ok
602.2951	1	C32H44NO10	100.00	602.2960	-1.4	-0.9	4.6	11.5	even	ok
607.2511	1	C32H40NaO10	100.00	607.2514	0.4	0.2	12.7	12.5	even	ok
1191.5140	1	C64H80NaO20	100.00	1191.5135	0.4	0.5	13.9	24.5	even	ok

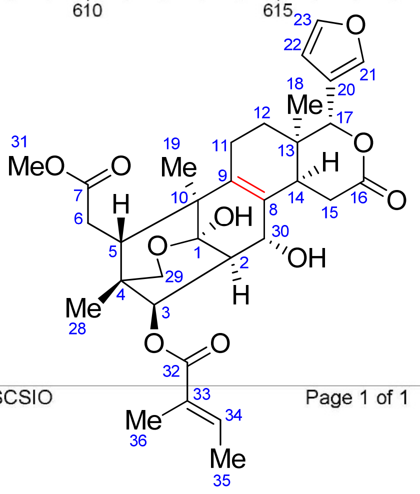

^{1}H NMR (400 MHz) spectrum of compound **5** in $CDCl_3$

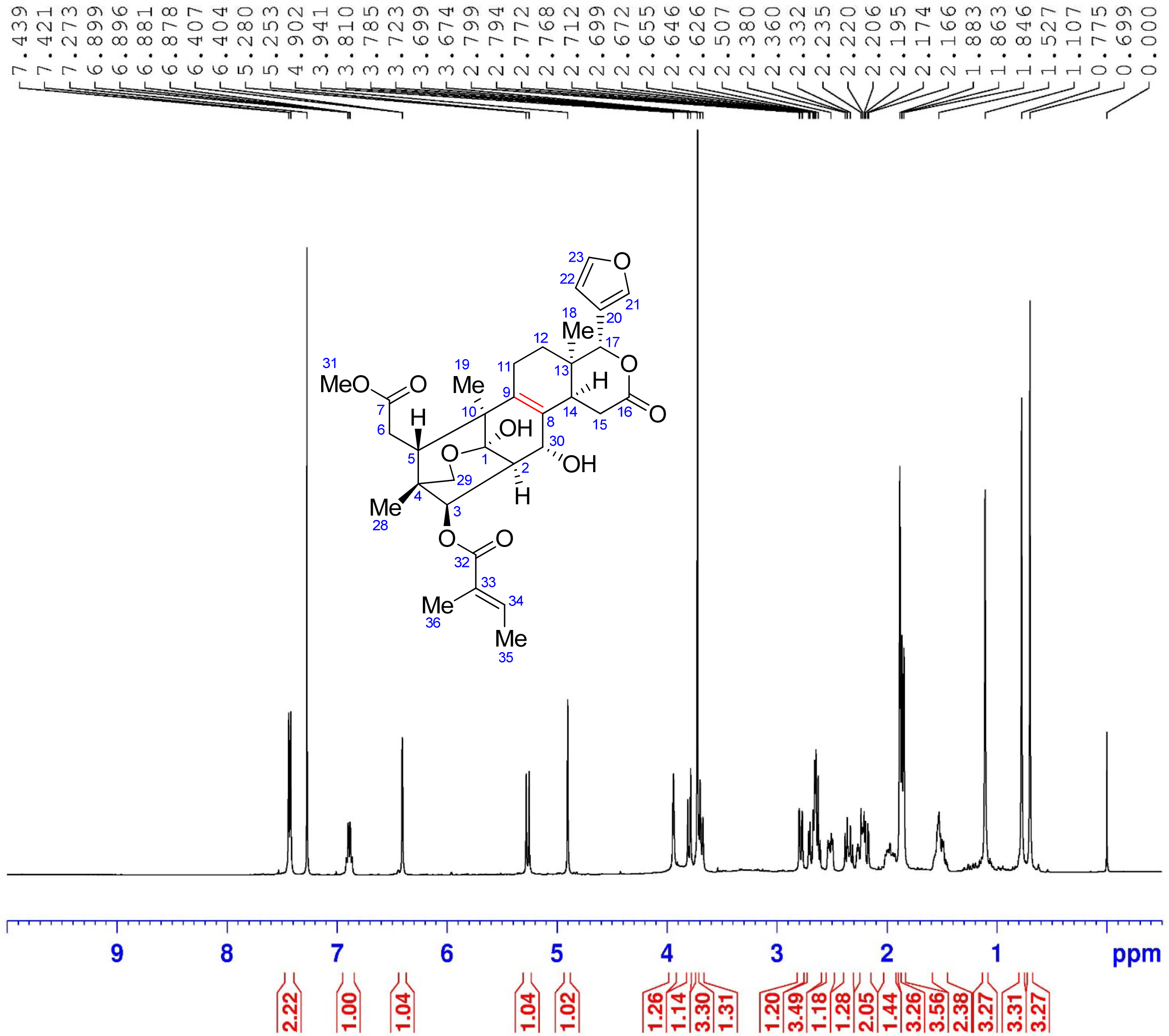

NAME	ZXP-R-18-2-2-2 new
EXPNO	1
PROCNO	1
Date_	20171206
Time	22.34
INSTRUM	spect
PROBHD	5 mm CPPBBO BB
PULPROG	zg30
TD	65536
SOLVENT	CDCl3
NS	16
DS	2
SWH	8223.685 Hz
FIDRES	0.125483 Hz
AQ	3.9846387 sec
RG	85.34
DW	60.800 usec
DE	10.00 usec
TE	297.0 K
D1	1.00000000 sec
TD0	1

======== CHANNEL f1 ========

SFO1	400.1324710 MHz
NUC1	1H
P1	11.50 usec
SI	65536
SF	400.1300045 MHz
WDW	EM
SSB	0
LB	0.30 Hz
GB	0
PC	1.00

^{1}H NMR (400 MHz) spectrum of compound **5** in $CDCl_3$

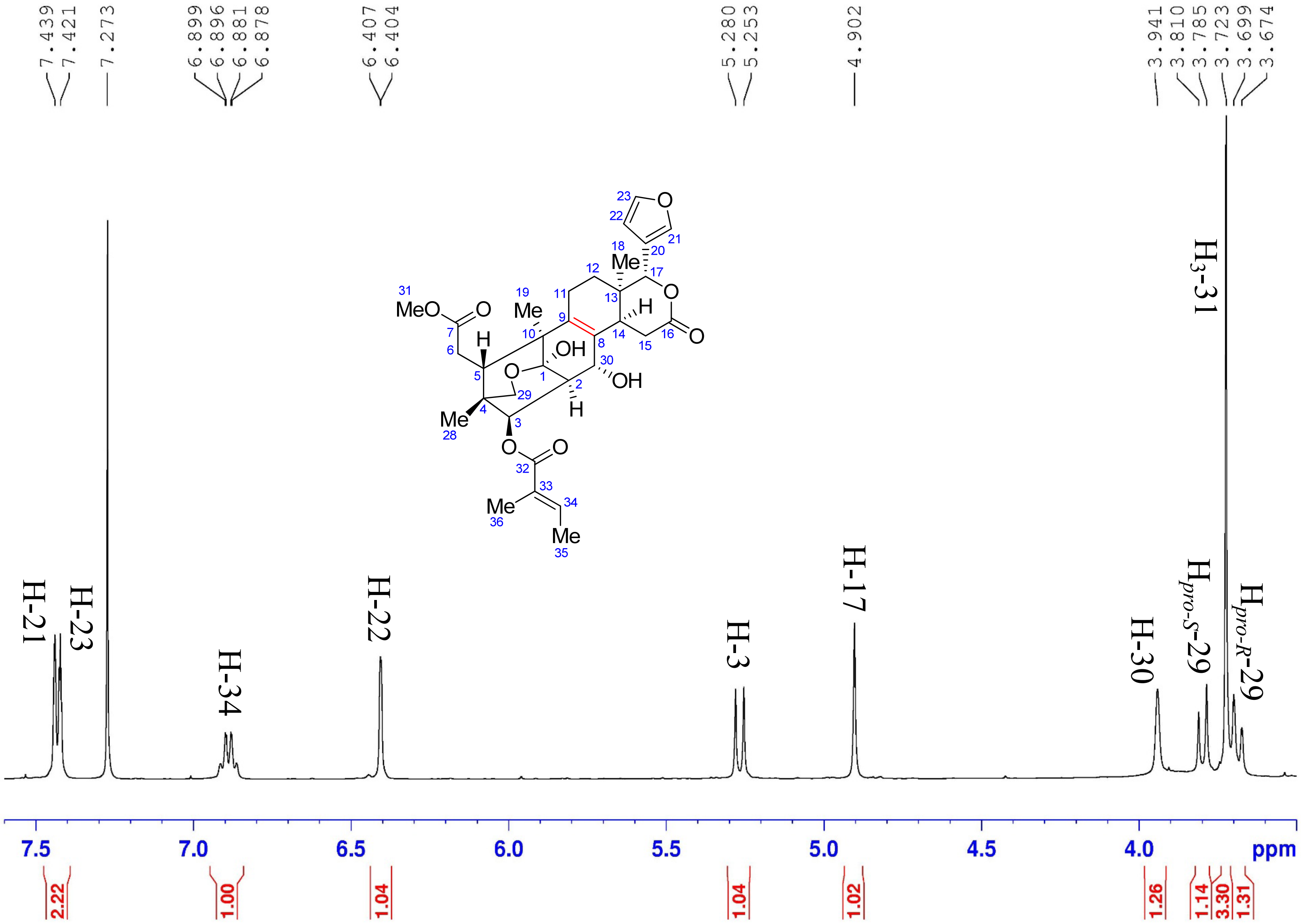

^{1}H NMR (400 MHz) spectrum of compound **5** in $CDCl_3$

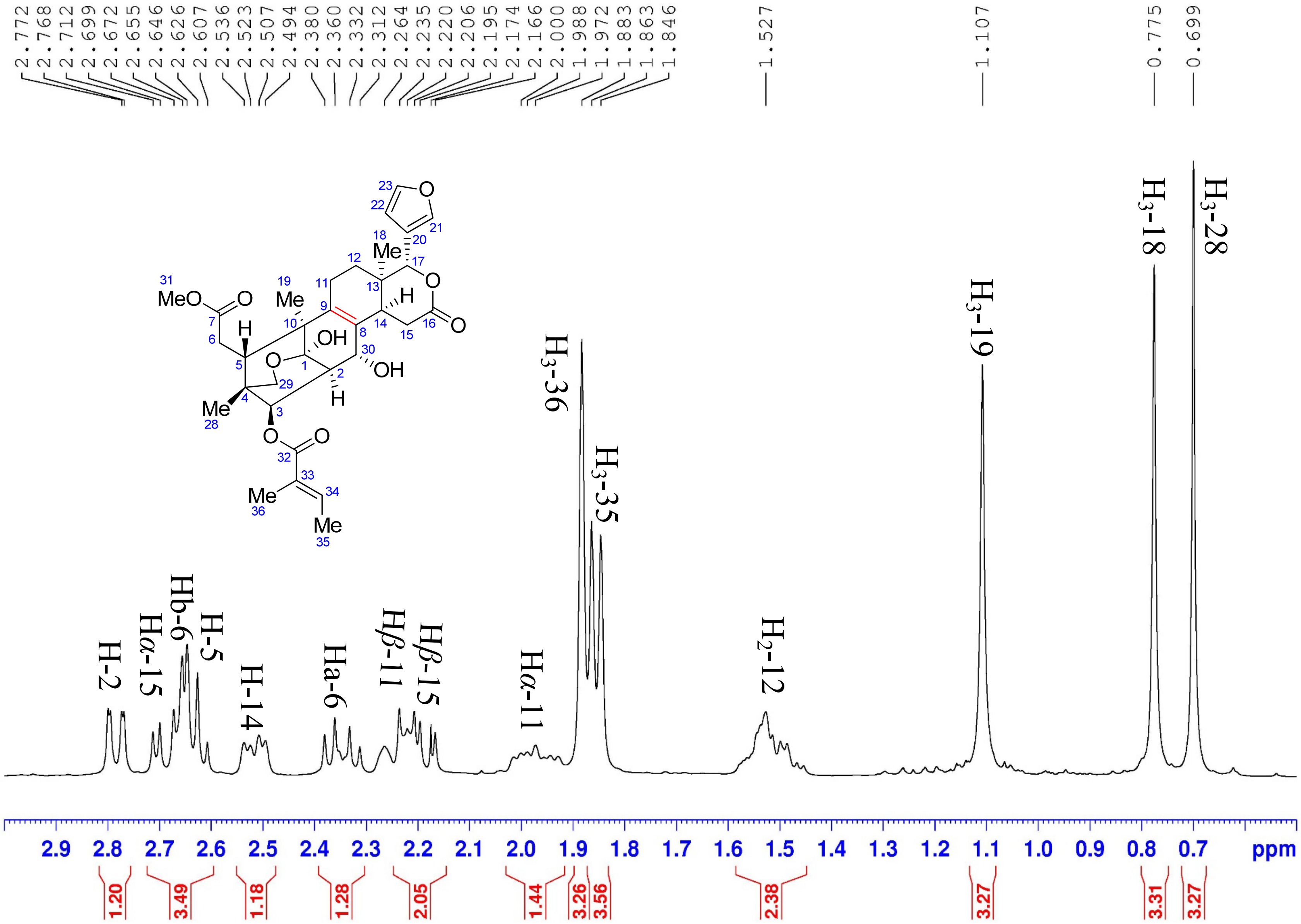

^{13}C NMR (100 MHz) spectrum of compound **5** in $CDCl_3$

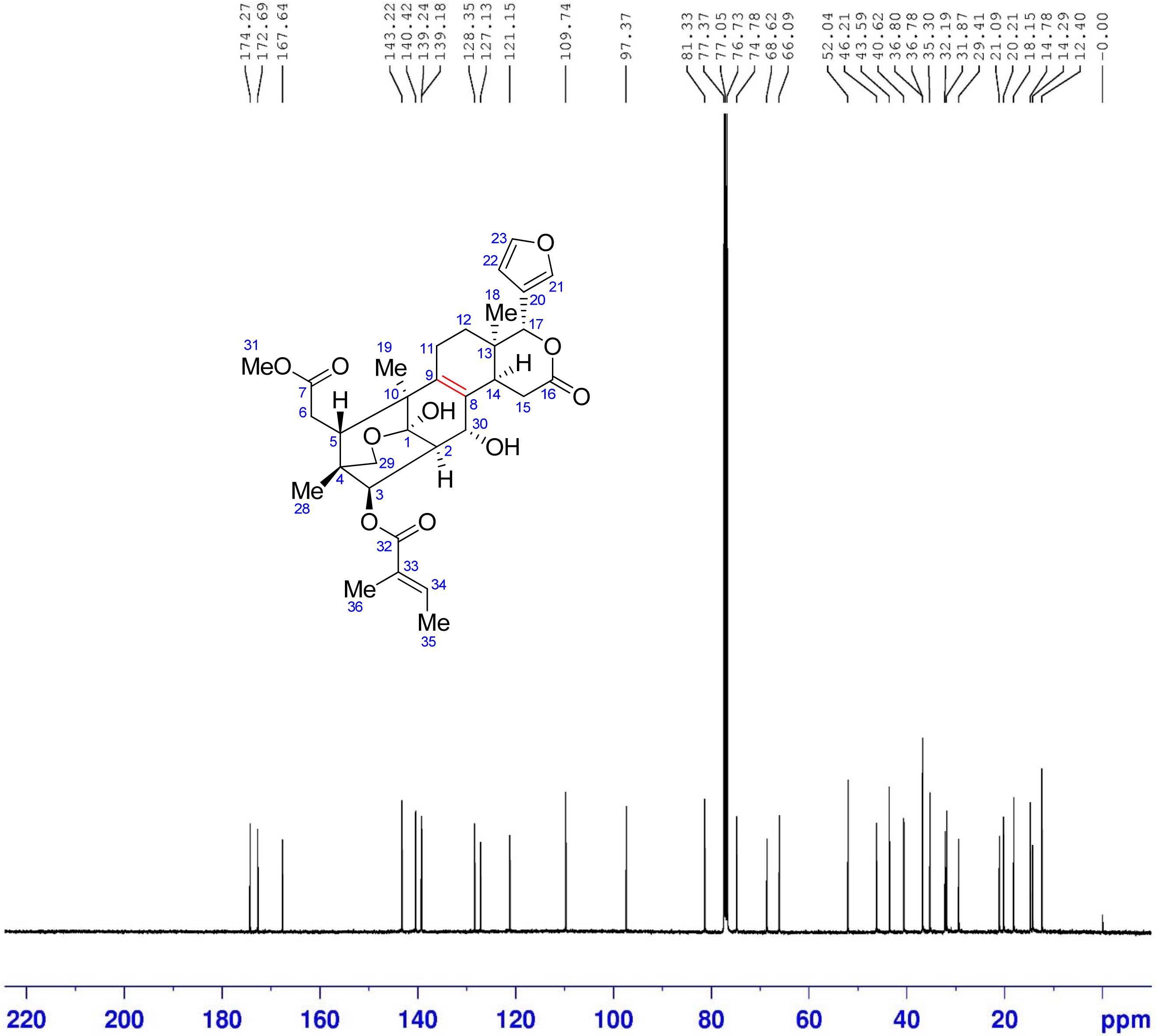

NAME	ZXP-R-18-2-2-2 new
EXPNO	2
PROCNO	1
Date_	20171206
Time	23.34
INSTRUM	spect
PROBHD	5 mm CPPBBO BB
PULPROG	zgpg30
TD	65536
SOLVENT	CDCl3
NS	1024
DS	4
SWH	24038.461 Hz
FIDRES	0.366798 Hz
AQ	1.3631988 sec
RG	117.37
DW	20.800 usec
DE	18.00 usec
TE	297.0 K
D1	2.00000000 sec
D11	0.03000000 sec
TD0	1

======== CHANNEL f1 ========

SFO1	100.6233324 MHz
NUC1	13C
P1	10.00 usec
SI	32768
SF	100.6127689 MHz
WDW	EM
SSB	0
LB	1.00 Hz
GB	0
PC	1.40

^{13}C NMR (100 MHz) spectrum of compound **5** in $CDCl_3$

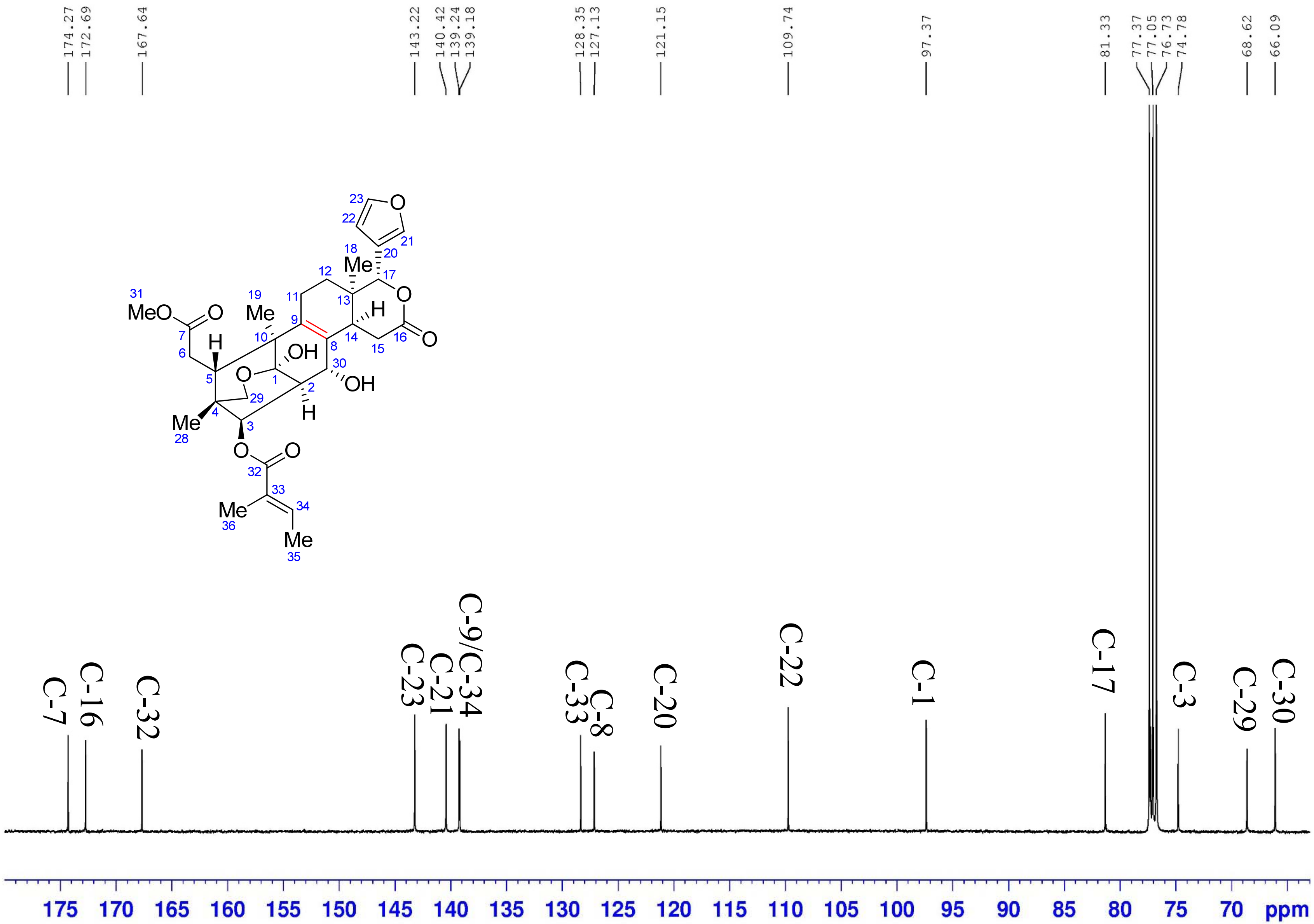

^{13}C NMR (100 MHz) spectrum of compound **5** in $CDCl_3$

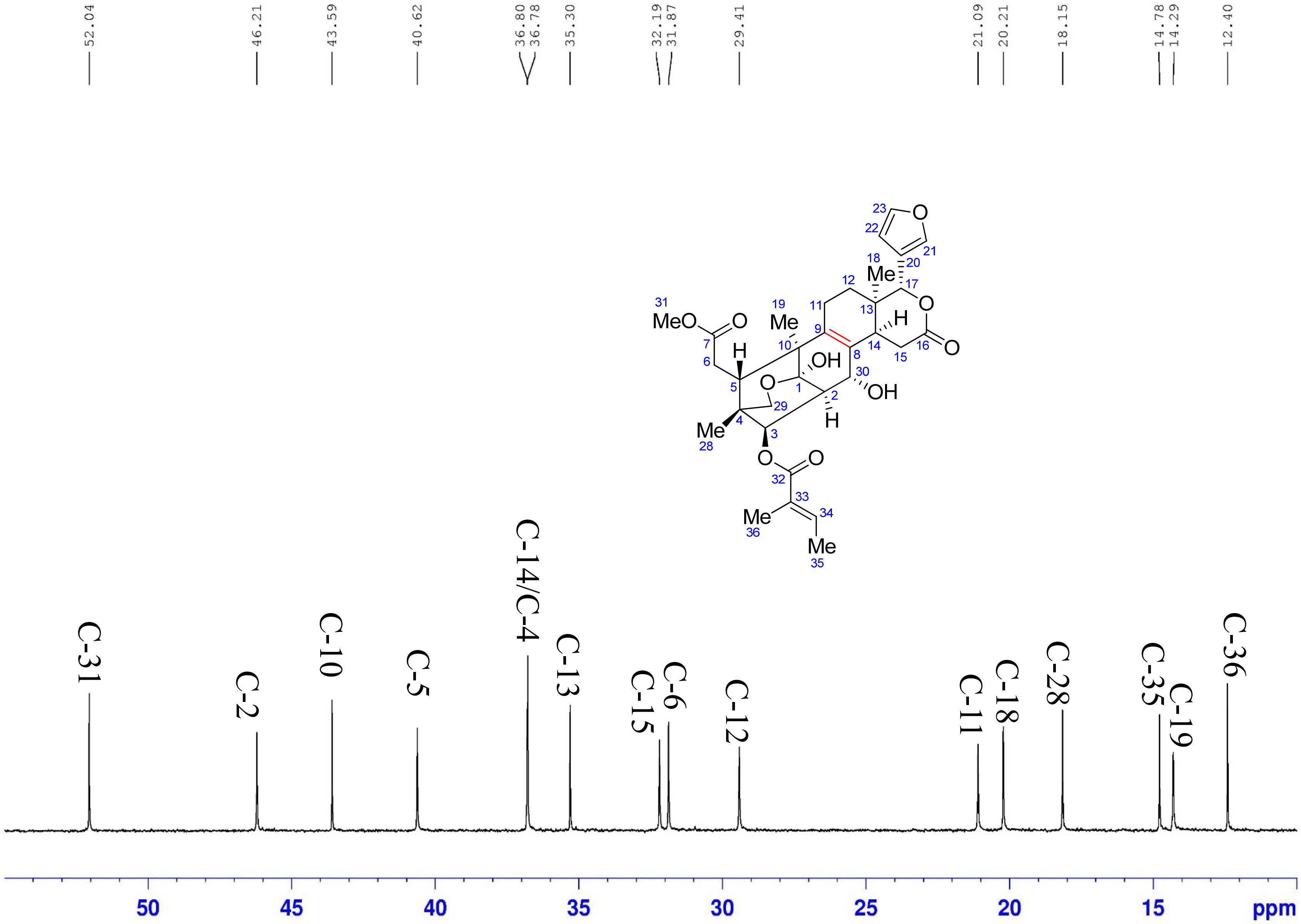

DEPT135 (100 MHz) spectrum of compound **5** in $CDCl_3$

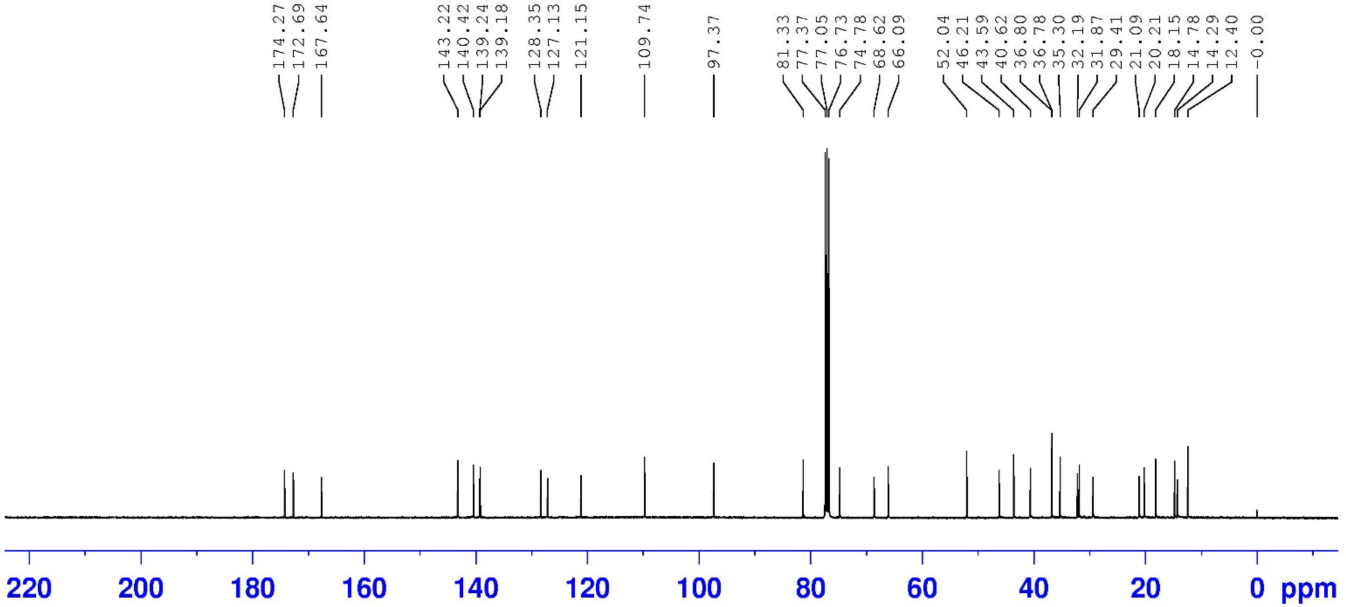


```
NAME        ZXP-R-18-2-2-2 new
EXPNO                    2
PROCNO                   1
Date_             20171206
Time                 23.34
INSTRUM              spect
PROBHD     5 mm CPPBBO BB
PULPROG            zgpg30
TD                   65536
SOLVENT              CDCl3
NS                    1024
DS                       4
SWH              24038.461 Hz
FIDRES            0.366798 Hz
AQ               1.3631988 sec
RG                  117.37
DW                  20.800 usec
DE                   18.00 usec
TE                   297.0 K
D1              2.00000000 sec
D11             0.03000000 sec
TD0                      1

======== CHANNEL f1 ========
SFO1           100.6233324 MHz
NUC1                   13C
P1                   10.00 usec
SI                   32768
SF             100.6127689 MHz
WDW                     EM
SSB                      0
LB                    1.00 Hz
GB                       0
PC                    1.40
```

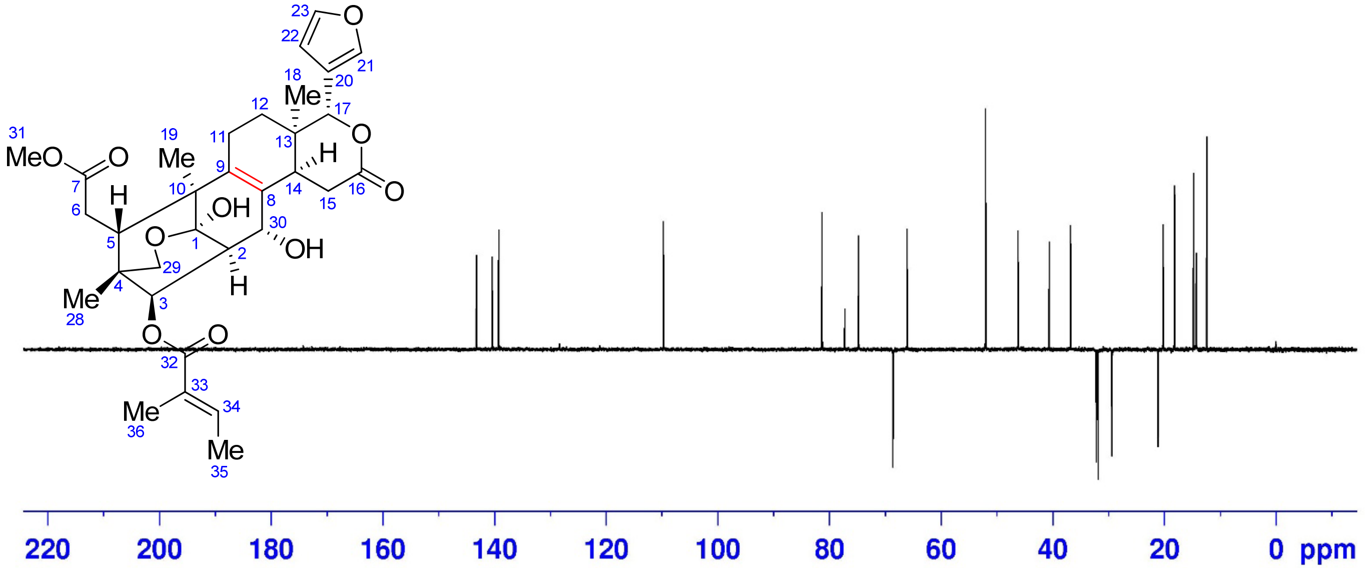

DEPT135 (100 MHz) spectrum of compound **5** in $CDCl_3$

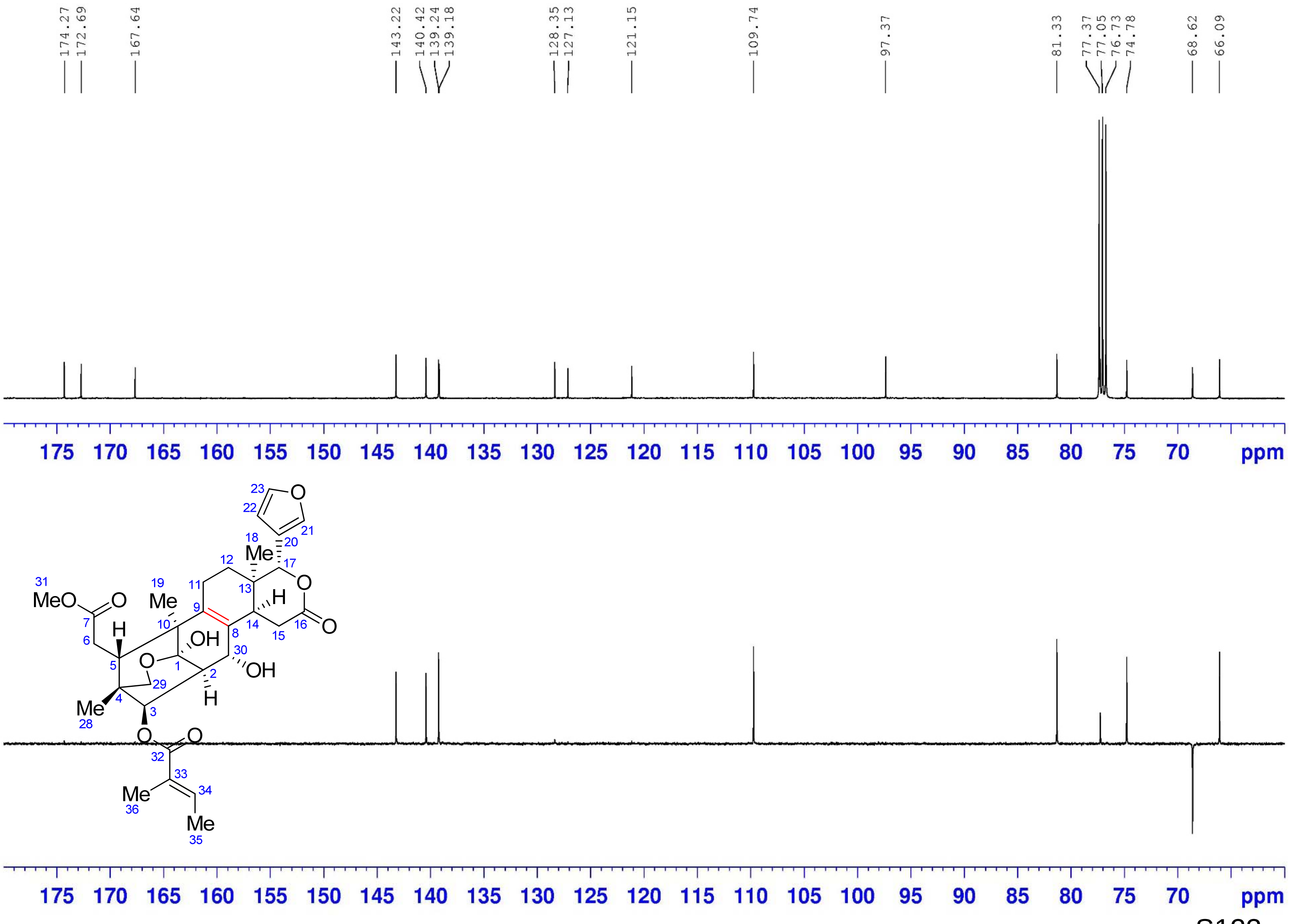

S123

DEPT135 (100 MHz) spectrum of compound **5** in $CDCl_3$

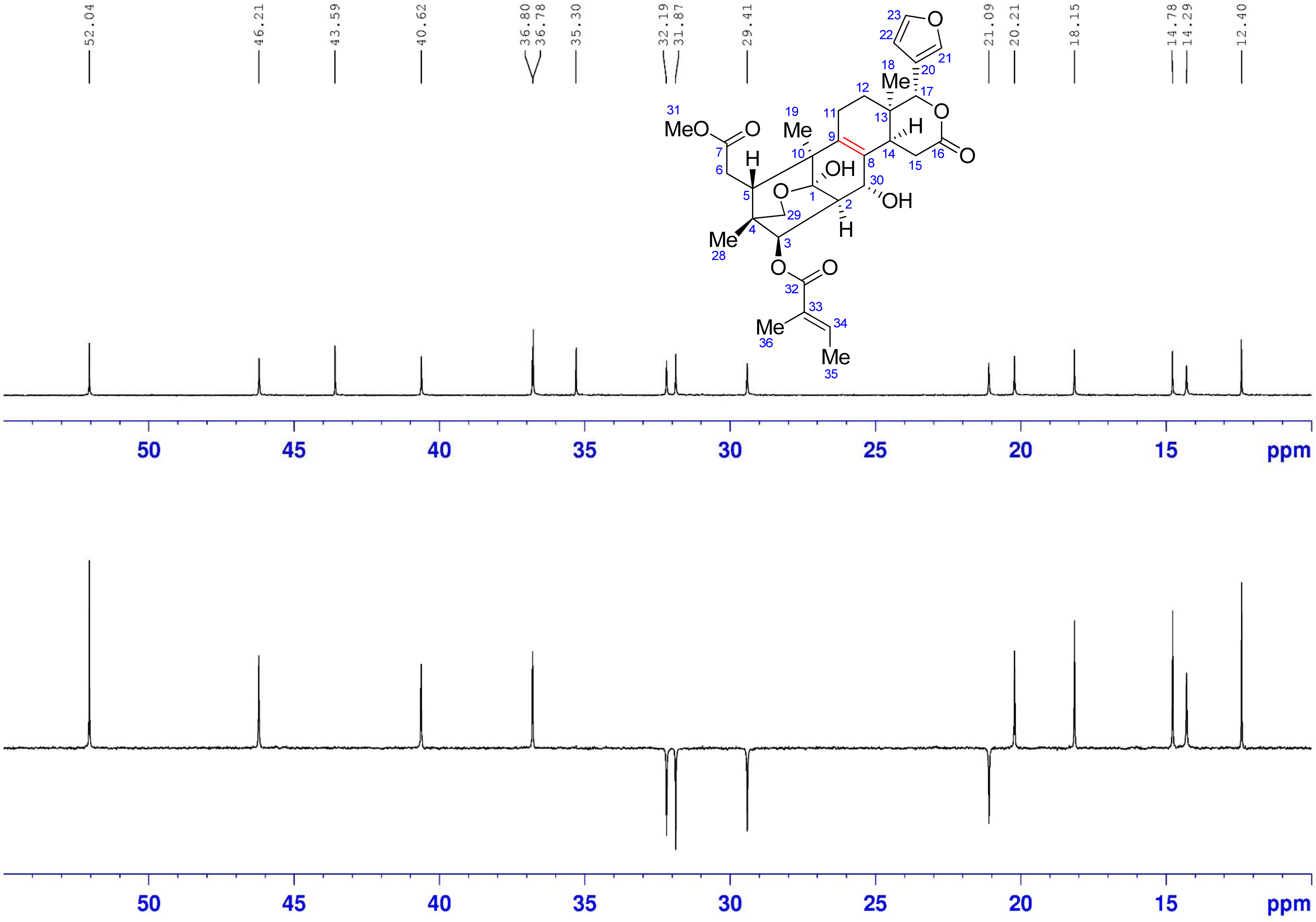

^{1}H-^{1}H COSY (400 MHz) spectrum of compound **5** in $CDCl_3$

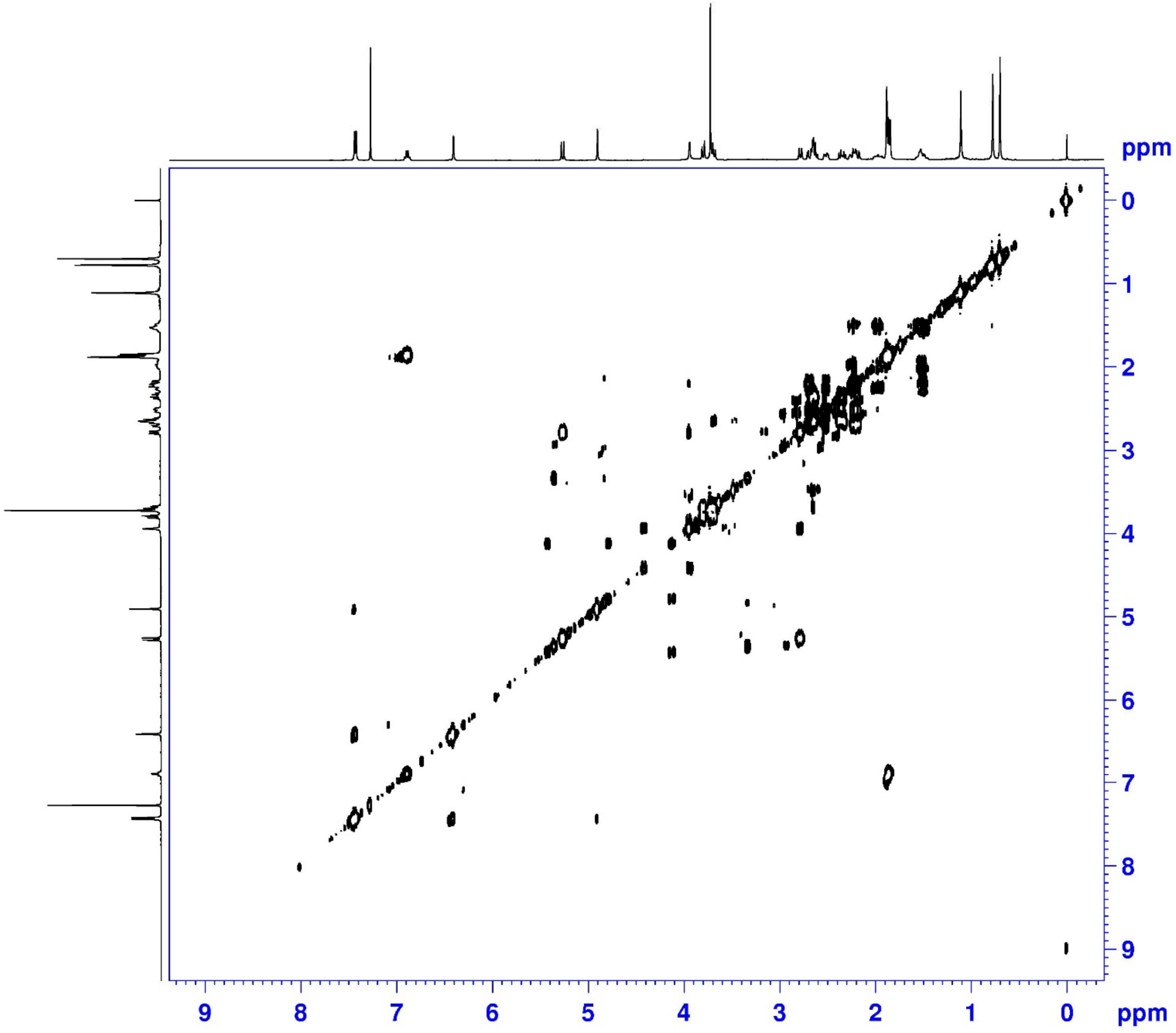


```
NAME        ZXP-R-18-2-2-2 new
EXPNO                    4
PROCNO                   1
Date_             20180110
Time                  4.22
INSTRUM              spect
PROBHD     5 mm CPPBBO BB
PULPROG         cosygpppqf
TD                    2048
SOLVENT              CDCl3
NS                       8
DS                       8
SWH               3906.250 Hz
FIDRES            1.907349 Hz
AQ               0.2621940 sec
RG                   91.64
DW                 128.000 usec
DE                   10.00 usec
TE                   297.0 K
D0              0.00000300 sec
D1              1.89678097 sec
D11             0.03000000 sec
D12             0.00002000 sec
D13             0.00000400 sec
D16             0.00020000 sec
IN0             0.00025600 sec

======== CHANNEL f1 ========
SFO1           400.1318006 MHz
NUC1                    1H
P0                   11.50 usec
P1                   11.50 usec
P17                2500.00 usec
ND0                      1
TD                     128
SFO1              400.1318 MHz
FIDRES           30.517578 Hz
SW                   9.762 ppm
FnMODE                  QF
SI                    1024
SF             400.1300024 MHz
WDW                  QSINE
SSB                      0
LB                    0.00 Hz
GB                       0
PC                    1.40
SI                    1024
MC2                     QF
SF             400.1300019 MHz
WDW                  QSINE
SSB                      0
LB                    0.00 Hz
GB                       0
```

^{1}H-^{1}H COSY (400 MHz) spectrum of compound **5** in $CDCl_3$

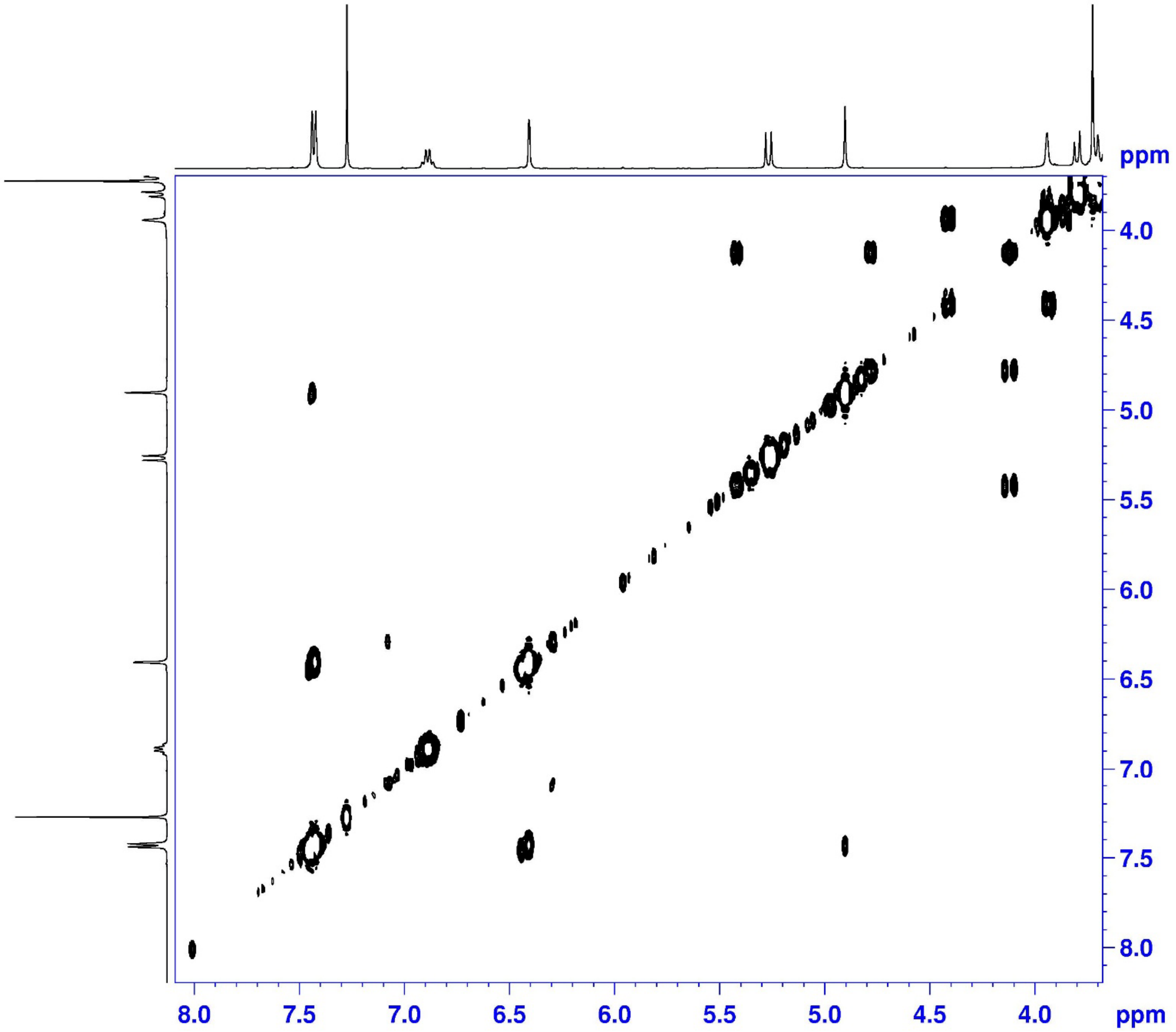

^1H-^1H COSY (400 MHz) spectrum of compound **5** in $CDCl_3$

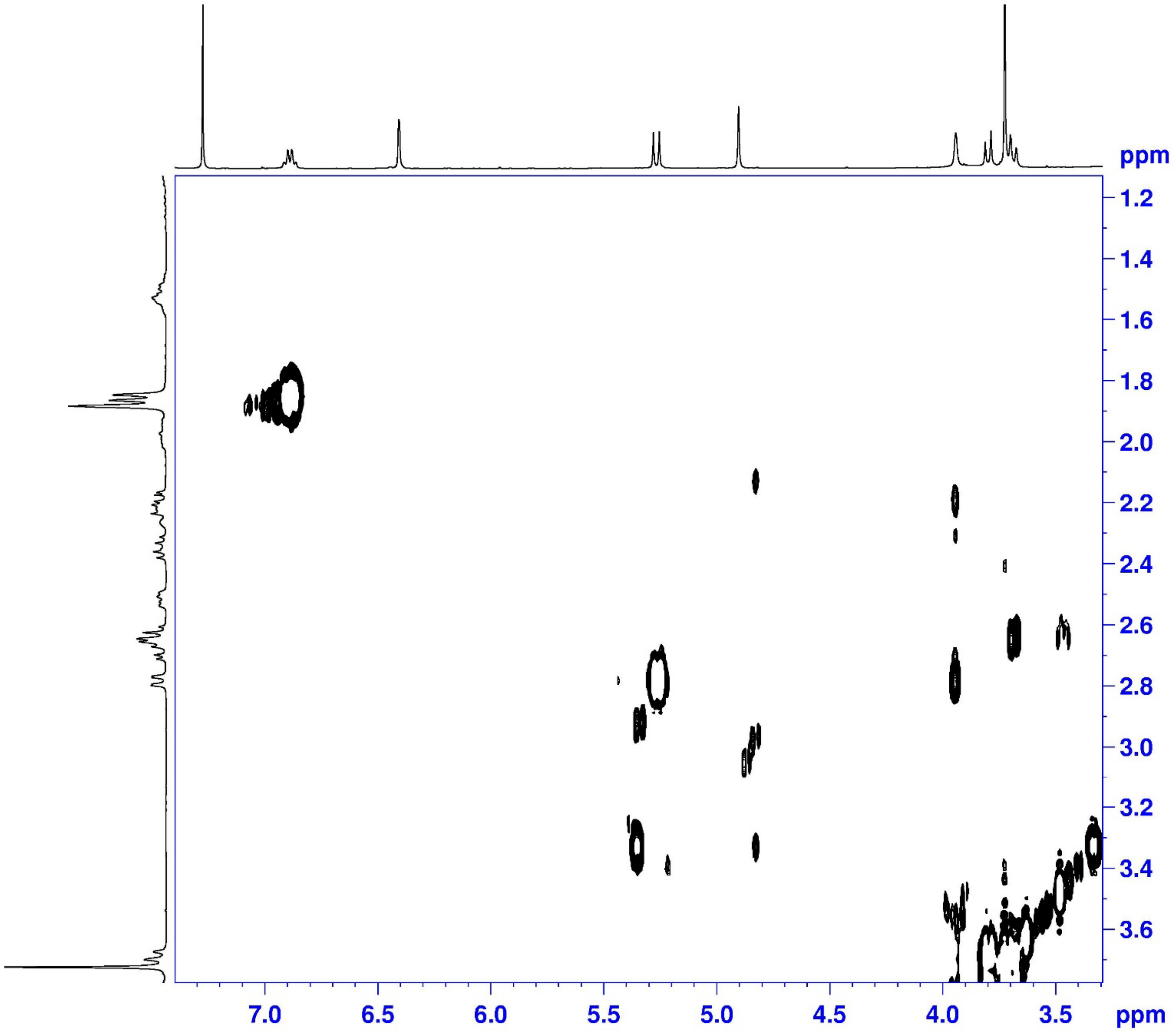

^1H-^1H COSY (400 MHz) spectrum of compound **5** in $CDCl_3$

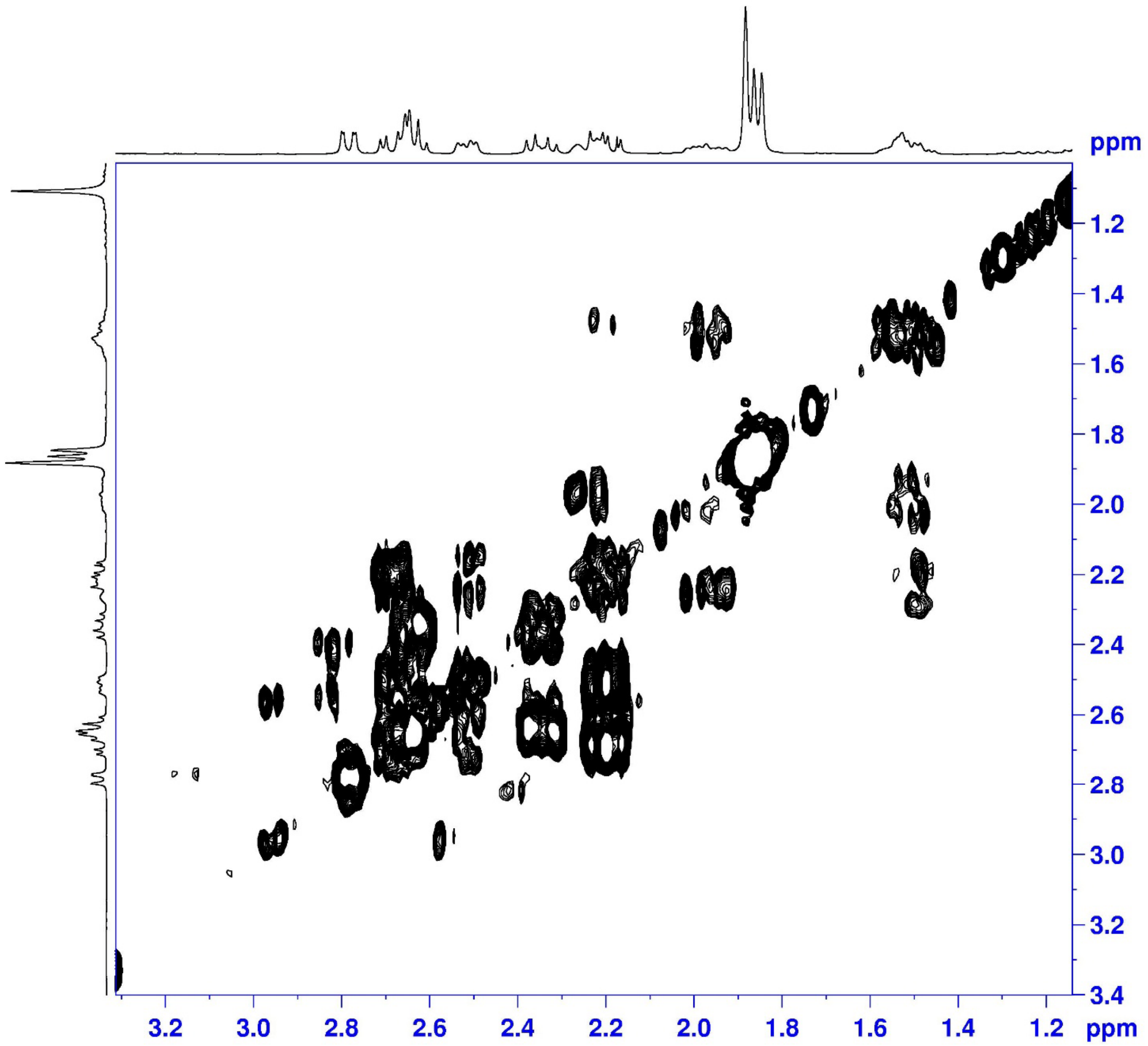

HSQC (400 MHz) spectrum of compound **5** in $CDCl_3$

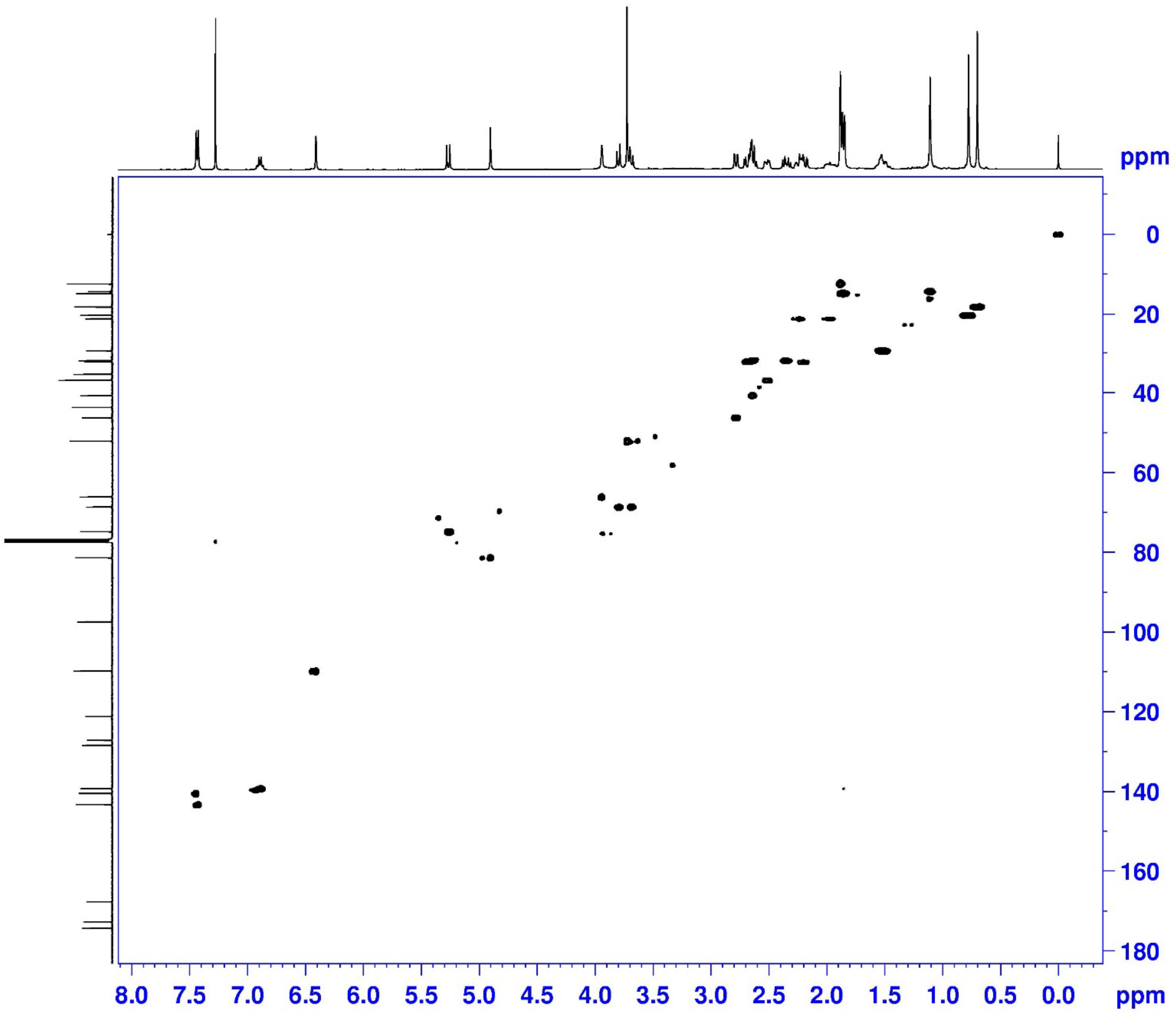


```
NAME      ZXP-R-18-2-2-2 new
EXPNO                    5
PROCNO                   1
Date_             20180110
Time                  5.01
INSTRUM              spect
PROBHD     5 mm CPPBBO BB
PULPROG        hsqcetgpsi2
TD                    1024
SOLVENT              CDCl3
NS                      16
DS                      16
SWH               4302.926 Hz
FIDRES            4.202076 Hz
AQ               0.1190388 sec
RG                   208.5
DW                 116.200 usec
DE                   10.00 usec
TE                   297.0 K
CNST2          145.0000000
D0              0.00000300 sec
D1              1.46497905 sec
D4              0.00172414 sec
D11             0.03000000 sec
D16             0.00020000 sec
D24             0.00086207 sec
IN0             0.00002080 sec
ZGOPTNS

======== CHANNEL f1 ========
SFO1          400.1320007 MHz
NUC1                    1H
P1                   11.50 usec
P2                   23.00 usec
P28                   0.00 usec
ND0                      2
TD                     256
SFO1              100.6233 MHz
FIDRES           93.900238 Hz
SW                 238.896 ppm
FnMODE        Echo-Antiecho
SI                    1024
SF            400.1300025 MHz
WDW                  QSINE
SSB                      2
LB                    0.00 Hz
GB                       0
PC                    1.40
SI                    1024
MC2           echo-antiecho
SF            100.6127564 MHz
WDW                  QSINE
SSB                      2
LB                    0.00 Hz
GB                       0
```

HSQC (400 MHz) spectrum of compound **5** in $CDCl_3$

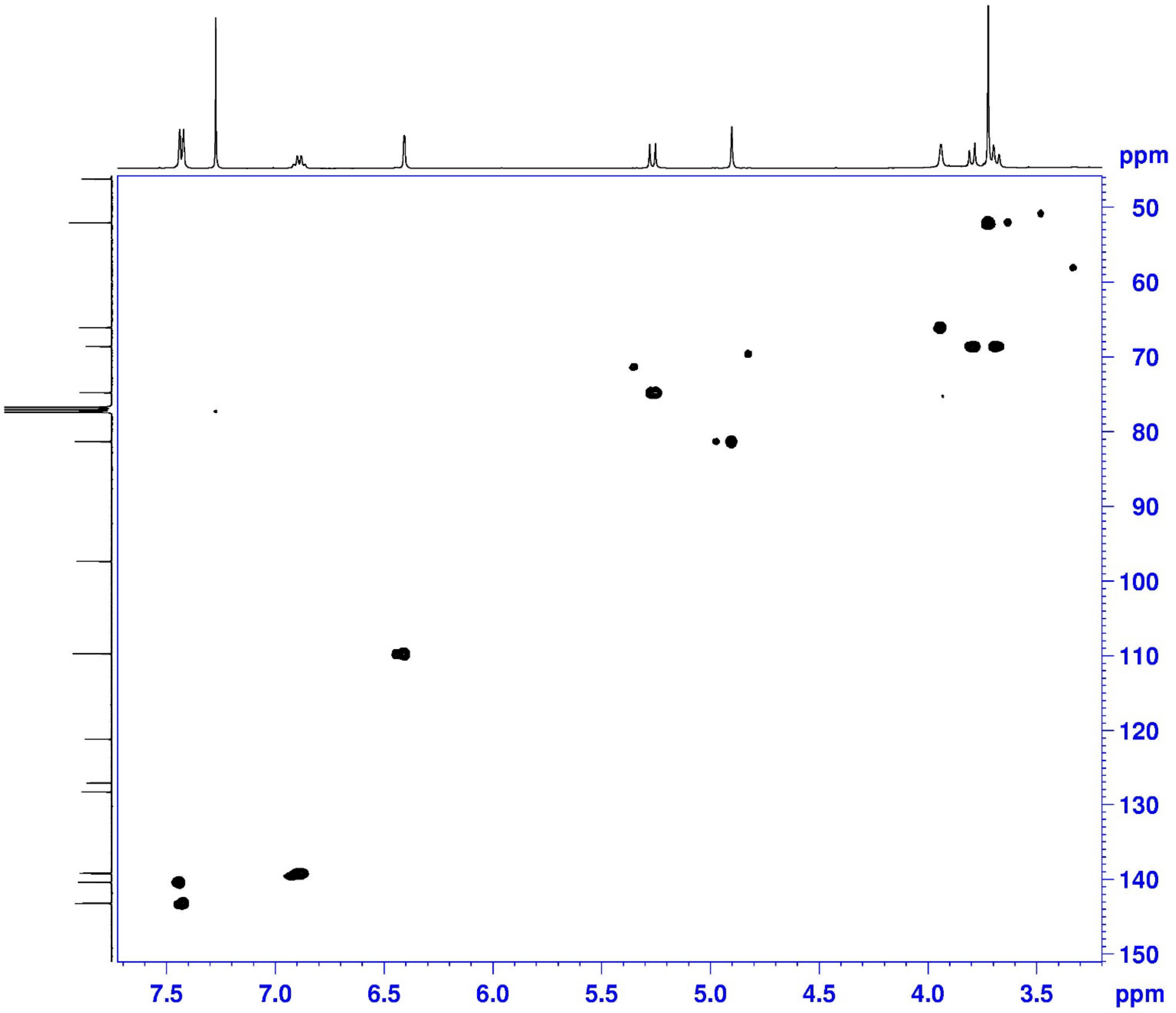

HSQC (400 MHz) spectrum of compound **5** in $CDCl_3$

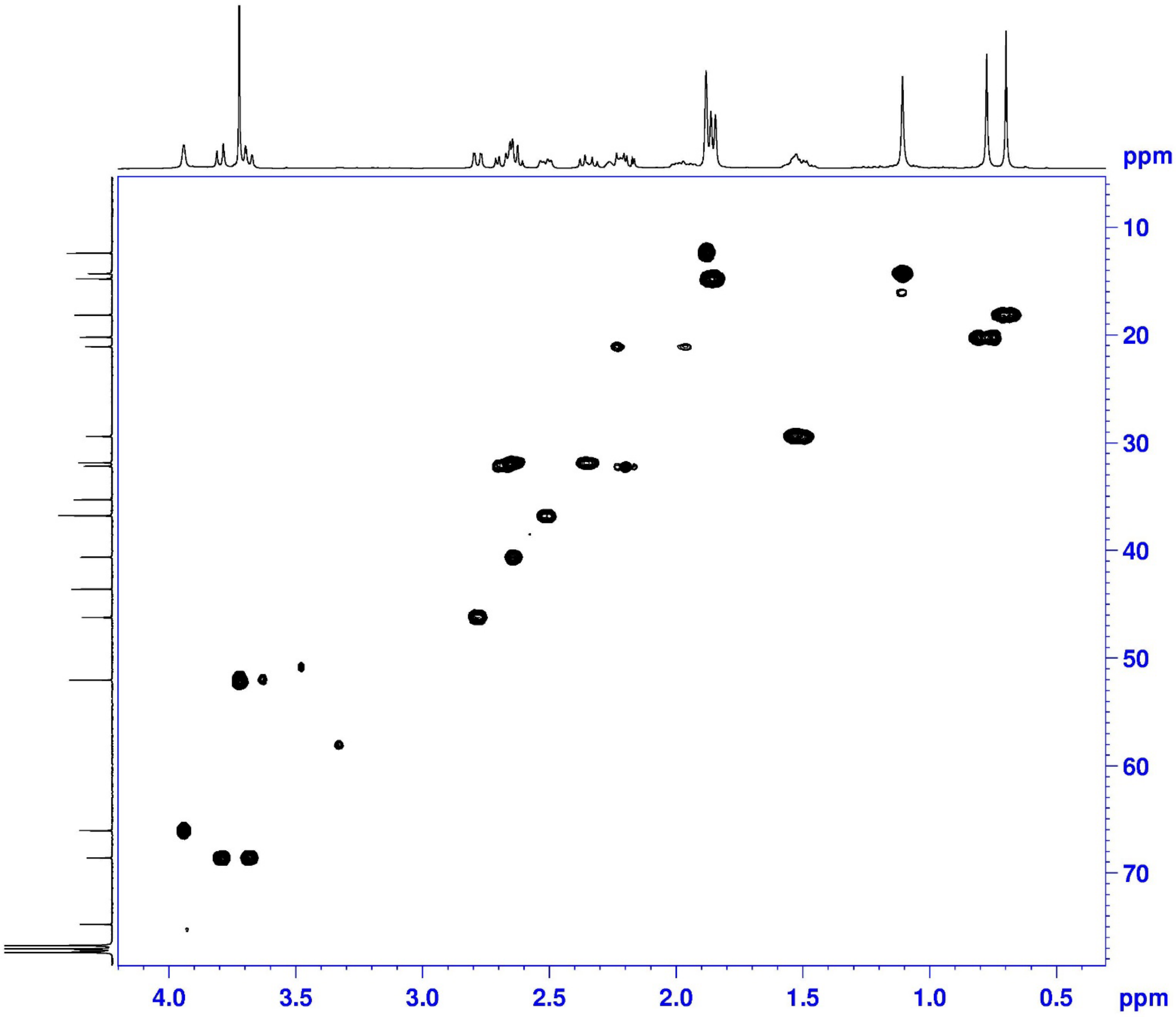

HMBC (400 MHz) spectrum of compound **5** in $CDCl_3$

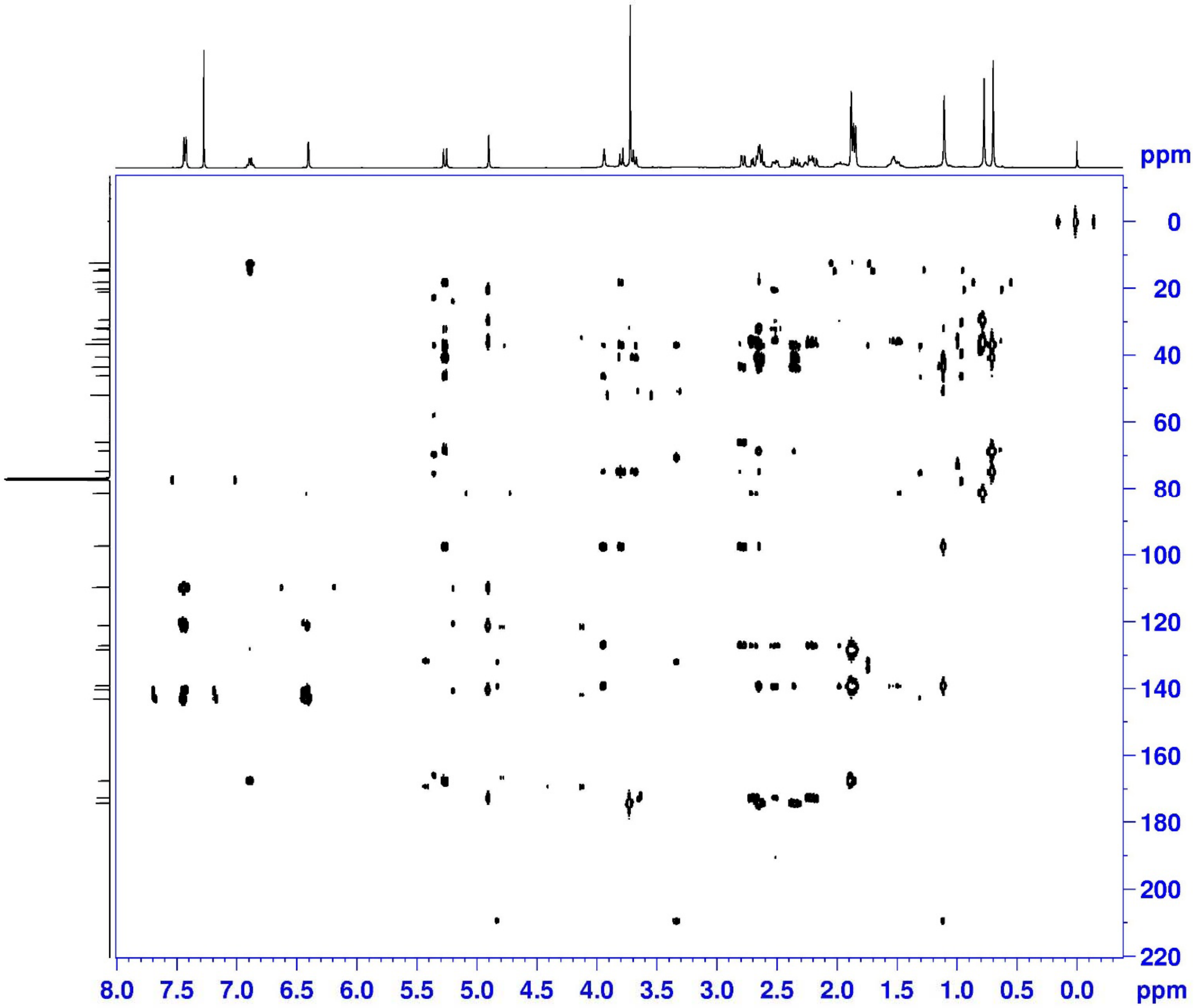


```
NAME       ZXP-R-18-2-2-2 new
EXPNO                    6
PROCNO                   1
Date_             20180110
Time                  6.53
INSTRUM              spect
PROBHD    5 mm CPPBBO BB
PULPROG       hmbcgplpndqf
TD                    4096
SOLVENT              CDCl3
NS                      32
DS                      16
SWH               5197.505 Hz
FIDRES            1.268922 Hz
AQ               0.3940852 sec
RG                   208.5
DW                  96.200 usec
DE                   10.00 usec
TE                   297.0 K
CNST2          145.0000000
CNST13          10.0000000
D0              0.00000300 sec
D1              1.50000000 sec
D2              0.00344828 sec
D6              0.05000000 sec
D16             0.00020000 sec
IN0             0.00002080 sec

======== CHANNEL f1 ========
SFO1           400.1323208 MHz
NUC1                    1H
P1                   11.50 usec
P2                   23.00 usec
ND0                      2
TD                     128
SFO1              100.6233 MHz
FIDRES          187.800476 Hz
SW                 238.896 ppm
FnMODE                  QF
SI                    2048
SF             400.1300026 MHz
WDW                   SINE
SSB                      0
LB                    0.00 Hz
GB                       0
PC                    1.40
SI                    1024
MC2                     QF
SF             100.6127604 MHz
WDW                   SINE
SSB                      0
LB                    0.00 Hz
GB                       0
```

HMBC (400 MHz) spectrum of compound **5** in $CDCl_3$

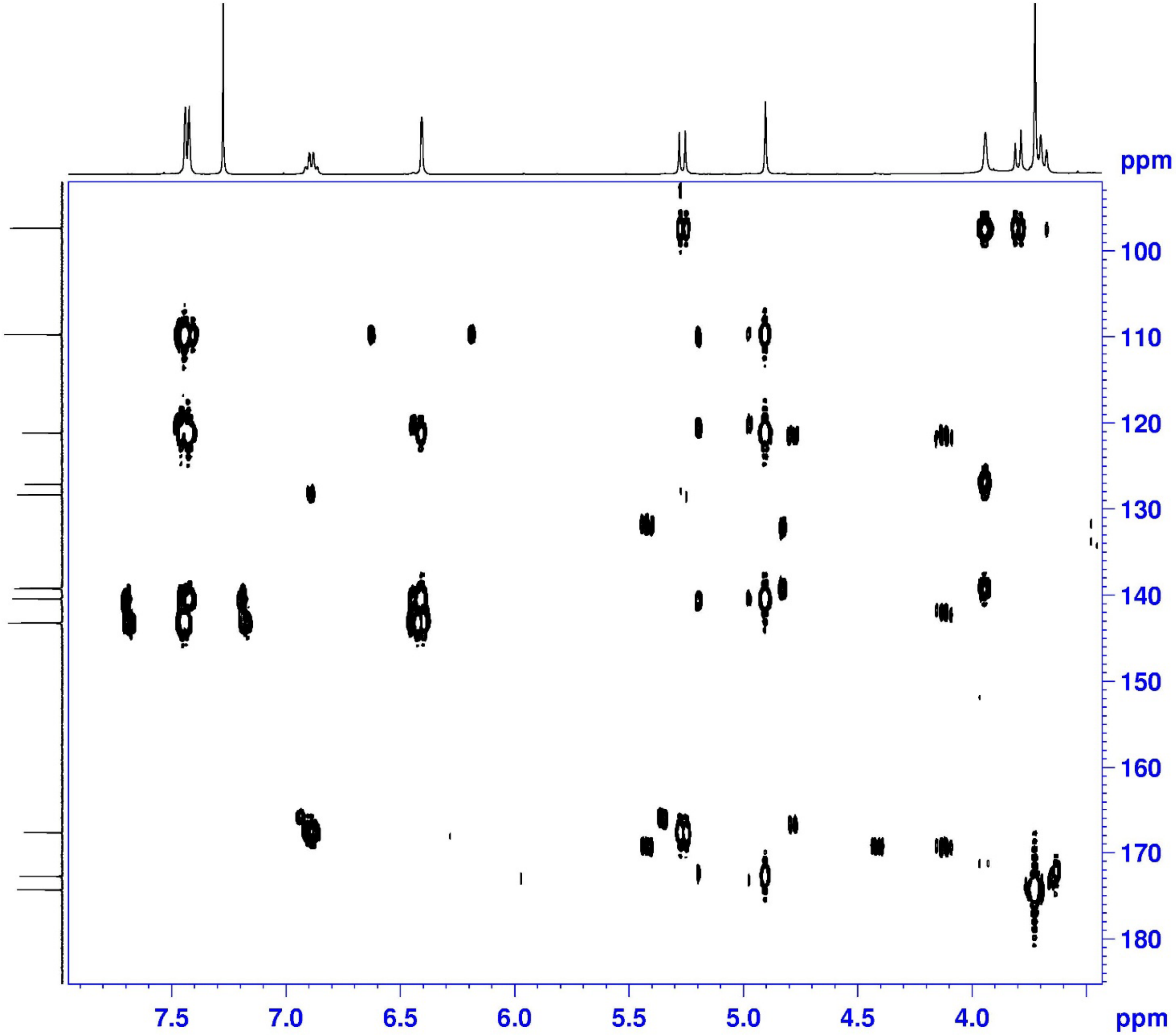

HMBC (400 MHz) spectrum of compound **5** in $CDCl_3$

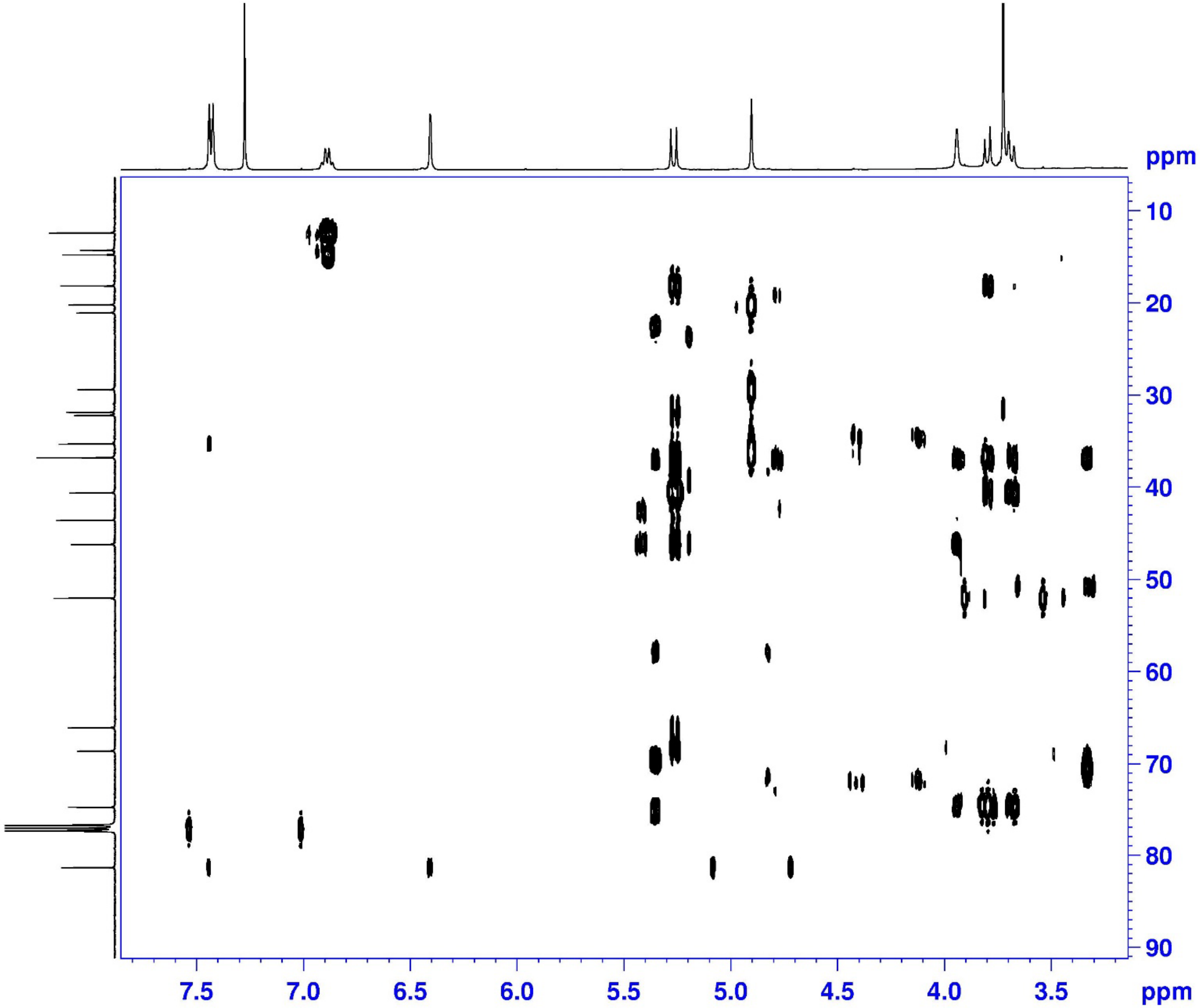

HMBC (400 MHz) spectrum of compound **5** in $CDCl_3$

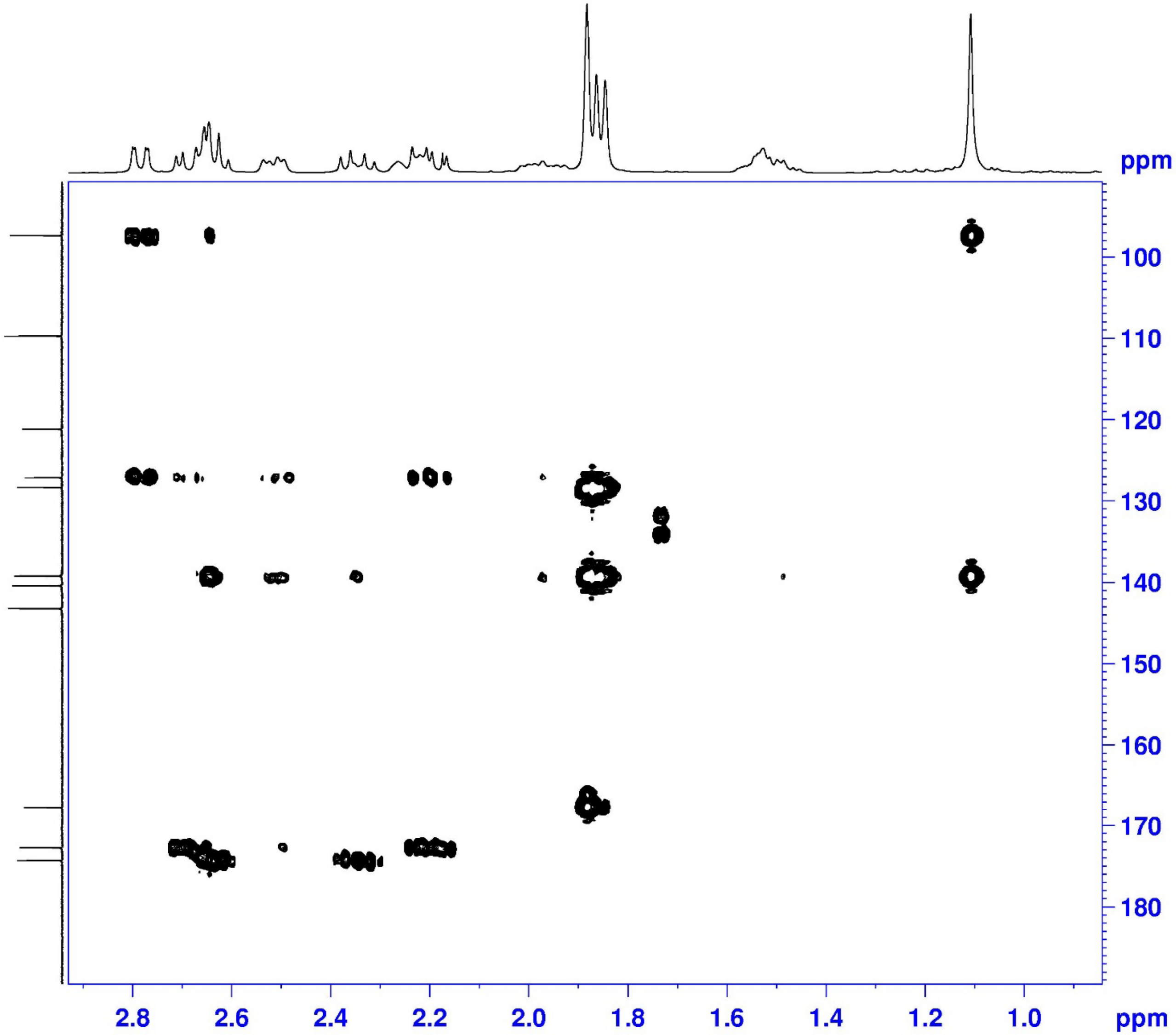

HMBC (400 MHz) spectrum of compound **5** in $CDCl_3$

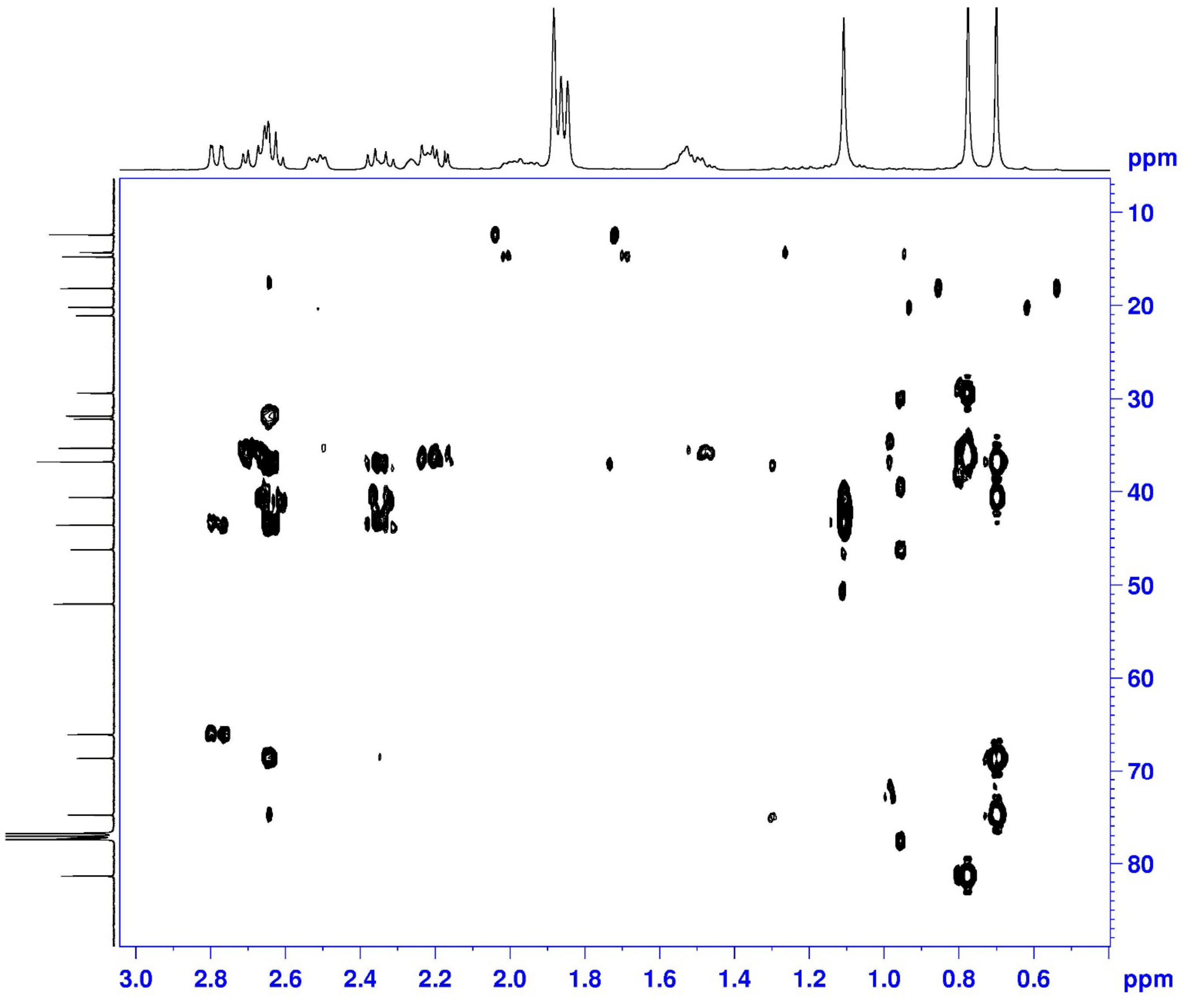

NOESY (400 MHz) spectrum of compound **5** in $CDCl_3$

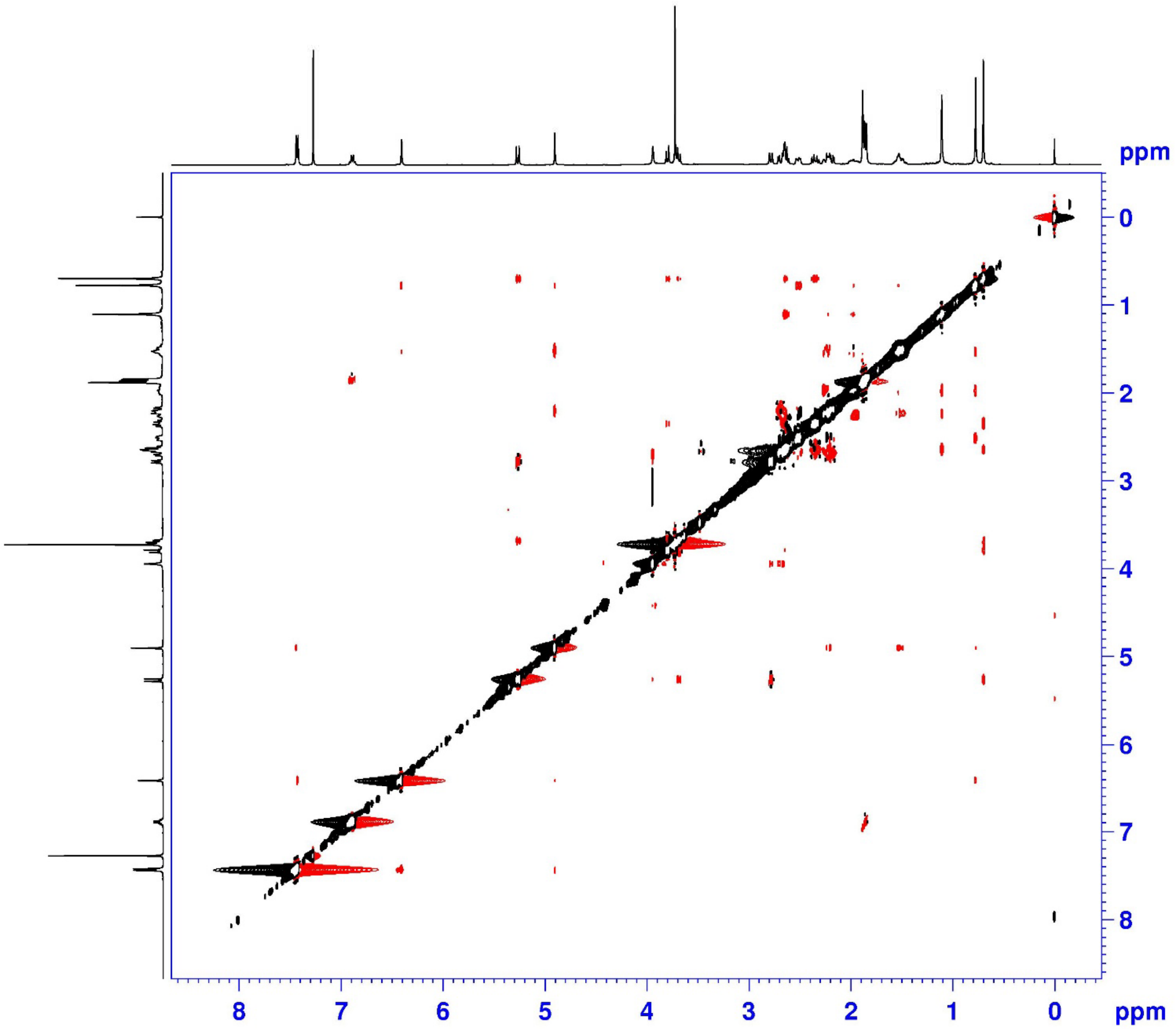


```
NAME       ZXP-R-18-2-2-2 new
EXPNO                     7
PROCNO                    1
Date_              20180110
Time                   9.08
INSTRUM               spect
PROBHD     5 mm CPPBBO BB
PULPROG        noesygpphpp
TD                     2048
SOLVENT               CDCl3
NS                       16
DS                       32
SWH             4000.000 Hz
FIDRES          1.953125 Hz
AQ             0.2560500 sec
RG                    85.34
DW               125.000 usec
DE                 10.00 usec
TE                 297.0 K
D0            0.00011036 sec
D1            1.99385595 sec
D8            0.30000001 sec
D11           0.03000000 sec
D12           0.00002000 sec
D16           0.00020000 sec
IN0           0.00025000 sec

======== CHANNEL f1 ========
SFO1         400.1318006 MHz
NUC1                     1H
P1                 11.50 usec
P2                 23.00 usec
P17              2500.00 usec
ND0                       1
TD                      256
SFO1            400.1318 MHz
FIDRES         15.625000 Hz
SW                 9.997 ppm
FnMODE          States-TPPI
SI                     1024
SF           400.1300018 MHz
WDW                   QSINE
SSB                       2
LB                  0.00 Hz
GB                        0
PC                     1.00
SI                     1024
MC2             States-TPPI
SF           400.1300029 MHz
WDW                   QSINE
SSB                       2
LB                  0.00 Hz
GB                        0
```

NOESY (400 MHz) spectrum of compound **5** in $CDCl_3$

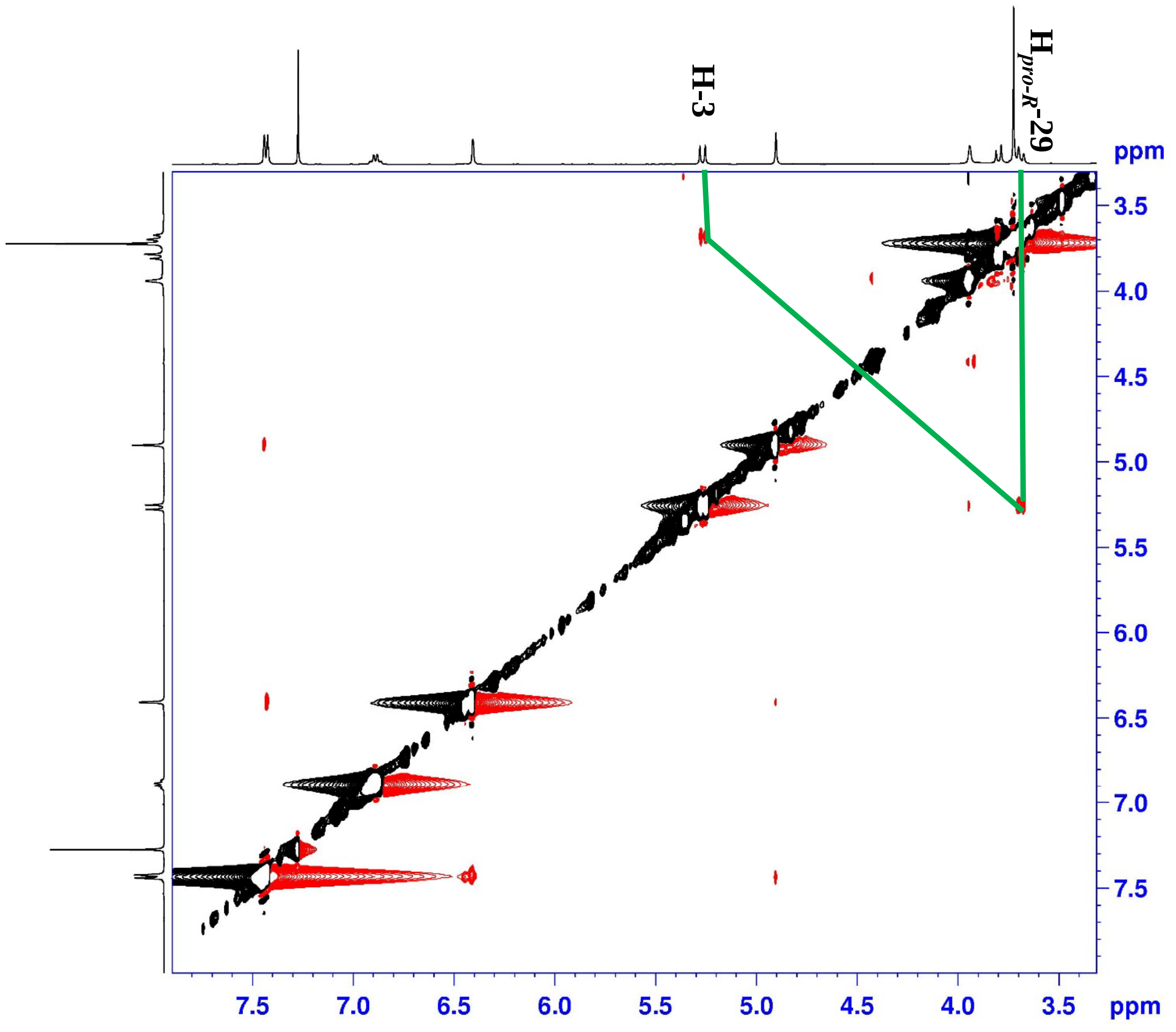

NOESY (400 MHz) spectrum of compound **5** in $CDCl_3$

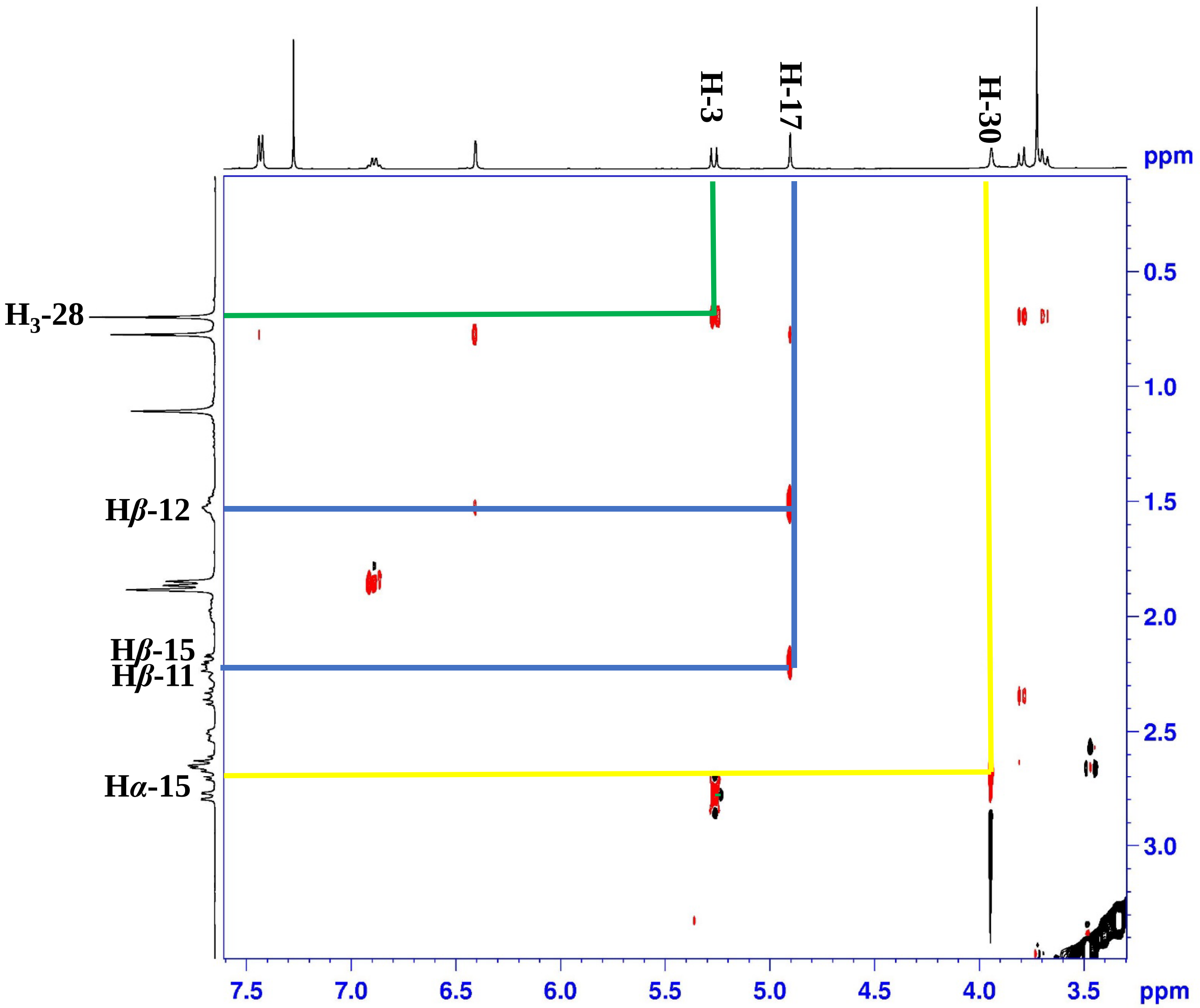

NOESY (400 MHz) spectrum of compound **5** in $CDCl_3$

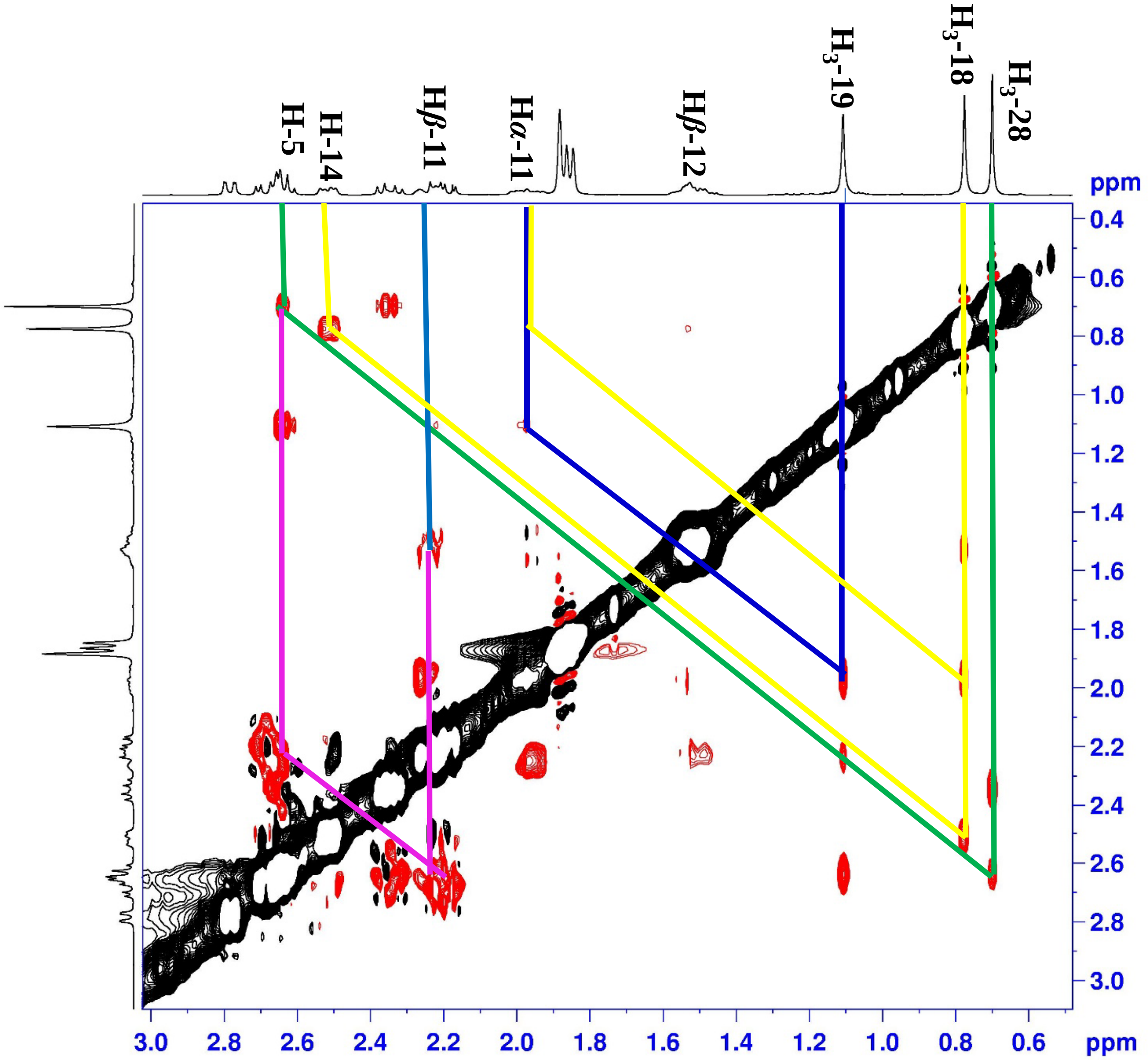

Supplement: Supplementary file 1 [file marinedrugs-16-00434-s001.zip › SupportingInformation-files/SI-I.pdf]
