# Supplementary material for: Limonoids Containing a C1–O–C29 Moiety: Isolation, Structural Modification, and Antiviral Activity
Source: Mar Drugs. 2018 Nov 4;16(11):434. doi: 10.3390/md16110434 (PMC6266505; doi:10.3390/md16110434)

Supporting Information-II

Copies of ESIMS, 1D and 2D NMR spectra of compounds 7–10, 8a–8i, 9a–9b, and 10a–10c.

Table of Contents:

1. LR-ESIMS for compound **7** ········ S6
2. ^{1}H NMR (400 MHz) spectrum of compound **7** in CD_3OD ········ S7-S10
3. ^{13}C NMR (100 MHz) spectrum of compound **7** in CD_3OD ········ S11-S14
4. DEPT 135 spectrum of compound **7** in CD_3OD ········ S15-S18
5. LR-ESIMS for compound **8** ········ S19
6. ^{1}H NMR (400 MHz) spectrum of compound **8** in $CDCl_3$ ········ S20-S23
7. ^{13}C NMR (100 MHz) spectrum of compound **8** in $CDCl_3$ ········ S24-S26
8. DEPT 135 spectrum of compound **8** in $CDCl_3$ ········ S27-S30
9. HR-ESIMS for compound **8a** ········ S31-S32
10. ^{1}H NMR (400 MHz) spectrum of compound **8a** in $CDCl_3$ ········ S33-S35
11. ^{13}C NMR (100 MHz) spectrum of compound **8a** in $CDCl_3$ ········ S36-S39
12. DEPT 135 spectrum of compound **8a** in $CDCl_3$ ········ S40-S43
13. HR-ESIMS for compound **8b** ········ S44-S45
14. ^{1}H NMR (400 MHz) spectrum of compound **8b** in $CDCl_3$ ········ S46-S48
15. ^{13}C NMR (100 MHz) spectrum of compound **8b** in $CDCl_3$ ········ S49-S52
16. DEPT 135 spectrum of compound **8b** in $CDCl_3$ ········ S53-S56
17. LR-ESIMS for compound **8c** ········ S57
18. ^{1}H NMR (400 MHz) spectrum of compound **8c** in $CDCl_3$ ········ S58-S60
19. ^{13}C NMR (100 MHz) spectrum of compound **8c** in $CDCl_3$ ········ S61-S64
20. DEPT 135 spectrum of compound **8c** in $CDCl_3$ ········ S65-S68
21. HR-ESIMS for compound **8d** ········ S69-S70
22. ^{1}H NMR (400 MHz) spectrum of compound **8d** in $CDCl_3$ ········ S71-S73

23. ^{13}C NMR (100 MHz) spectrum of compound **8d** in $CDCl_3$ ····· S74-S77
24. DEPT 135 spectrum of compound **8d** in CDCl3 ····· S78-S81
25. HR-ESIMS for compound **8e** ····· S82-S83
26. 1H NMR (400 MHz) spectrum of compound **8e** in CDCl3 ····· S84-S86
27. 13C NMR (100 MHz) spectrum of compound **8e** in CDCl3 ····· S87-S91
28. DEPT 135 spectrum of compound **8e** in CDCl3 ····· S92-S95
29. HR-ESIMS for compound **8f** ····· S96-S97
30. 1H NMR (400 MHz) spectrum of compound **8f** in CDCl3 ····· S98-S100
31. 13C NMR (100 MHz) spectrum of compound **8f** in CDCl3 ····· S101-S104
32. DEPT 135 spectrum of compound **8f** in CDCl3 ····· S105-S108
33. HR-ESIMS for compound **8g** ····· S109-S110
34. 1H NMR (400 MHz) spectrum of compound **8g** in CDCl3 ····· S111-S113
35. 13C NMR (100 MHz) spectrum of compound **8g** in CDCl3 ····· S114-S117
36. DEPT 135 spectrum of compound **8g** in CDCl3 ····· S118-S122
37. HR-ESIMS for compound **8h** ····· S123-S124
38. 1H NMR (400 MHz) spectrum of compound **8h** in CDCl3 ····· S125-S127
39. 13C NMR (100 MHz) spectrum of compound **8h** in CDCl3 ····· S128-S131
40. DEPT 135 spectrum of compound **8h** in CDCl3 ····· S132-S135
41. HR-ESIMS for compound **8i** ····· S136-S137
42. 1H NMR (400 MHz) spectrum of compound **8i** in CDCl3 ····· S138-S140
43. 13C NMR (100 MHz) spectrum of compound **8i** in CDCl3 ····· S141-S144
44. DEPT 135 spectrum of compound **8i** in CDCl3 ····· S145-S149
45. LR-ESIMS for compound **9** ····· S150

46. ^{1}H NMR (400 MHz) spectrum of compound **9** in $CDCl_3$ ··························· S151-S154
47. ^{13}C NMR (100 MHz) spectrum of compound **9** in $CDCl_3$ ························ S155-S158
48. DEPT 135 spectrum of compound **9** in $CDCl_3$ ·· S159-S164
49. LR-ESIMS for compound **9a** ··· S165
50. ^{1}H NMR (400 MHz) spectrum of compound **9a** in $CDCl_3$ ························ S166-S168
51. ^{13}C NMR (100 MHz) spectrum of compound **9a** in $CDCl_3$ ······················ S169-S171
52. DEPT 135 spectrum of compound **9a** in $CDCl_3$ ······································ S172-S175
53. LR-ESIMS for compound **9b** ··· S176
54. ^{1}H NMR (400 MHz) spectrum of compound **9b** in $CDCl_3$ ························ S177-S180
55. ^{13}C NMR (100 MHz) spectrum of compound **9b** in $CDCl_3$ ······················ S181-S183
56. DEPT 135 spectrum of compound **9b** in $CDCl_3$ ······································ S184-S187
57. LR-ESIMS for compound **10** ··· S188
58. ^{1}H NMR (400 MHz) spectrum of compound **10** in $(CD_3)_2CO$ ···················· S189-S192
59. ^{13}C NMR (100 MHz) spectrum of compound **10** in $(CD_3)_2CO$ ·················· S193-S196
60. DEPT 135 spectrum of compound **10** in $(CD_3)_2CO$ ·································· S197-S199
61. LR-ESIMS for compound **10a** ··· S200
62. ^{1}H NMR (400 MHz) spectrum of compound **10a** in $(CD_3)_2CO$ ·················· S201-S203
63. ^{13}C NMR (100 MHz) spectrum of compound **10a** in $(CD_3)_2CO$ ················ S204-S206
64. DEPT 135 spectrum of compound **10a** in $(CD_3)_2CO$ ································ S207-S209
65. Positive LR-ESIMS for compound **10b** ··· S210
66. ^{1}H NMR (400 MHz) spectrum of compound **10b** in $(CD_3)_2CO$ ·················· S211-S213
67. ^{13}C NMR (100 MHz) spectrum of compound **10b** in $(CD_3)_2CO$ ················ S214-S216
68. DEPT 135 spectrum of compound **10b** in $(CD_3)_2CO$ ································ S217-S219

61. LR-ESIMS for compound **10c**······S220
62. ^{1}H NMR (400 MHz) spectrum of compound **10c** in $(CD_3)_2CO$······S221-S223
63. ^{13}C NMR (100 MHz) spectrum of compound **10c** in $(CD_3)_2CO$······S224-S226
64. DEPT 135 spectrum of compound **10c** in $(CD_3)_2CO$······S227-S229

LR-ESIMS for compound **7**

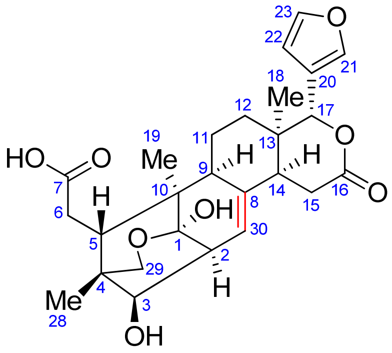

7

Chemical Formula: $C_{26}H_{32}O_8$
Exact Mass: 472.20972

Generic Display Report

Analysis Info
Analysis Name D:\MS\RJL\180915\62-2018-09-1520-31-45.D
Method DEF_LC.M
Sample Name 62
Comment

Acquisition D 2018/9/15 20:32:00
Operator
Instrument LCMSD

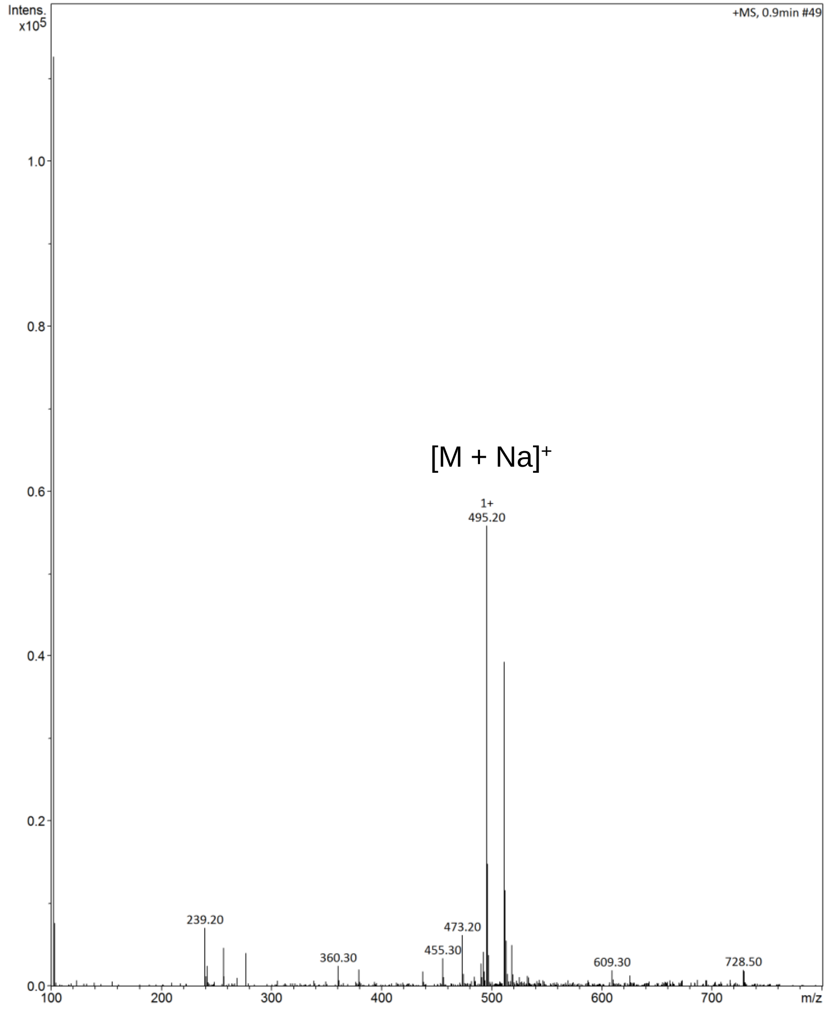

Bruker Compass DataAnalysis

^1H NMR (400 MHz) spectrum of **7** in CD_3OD

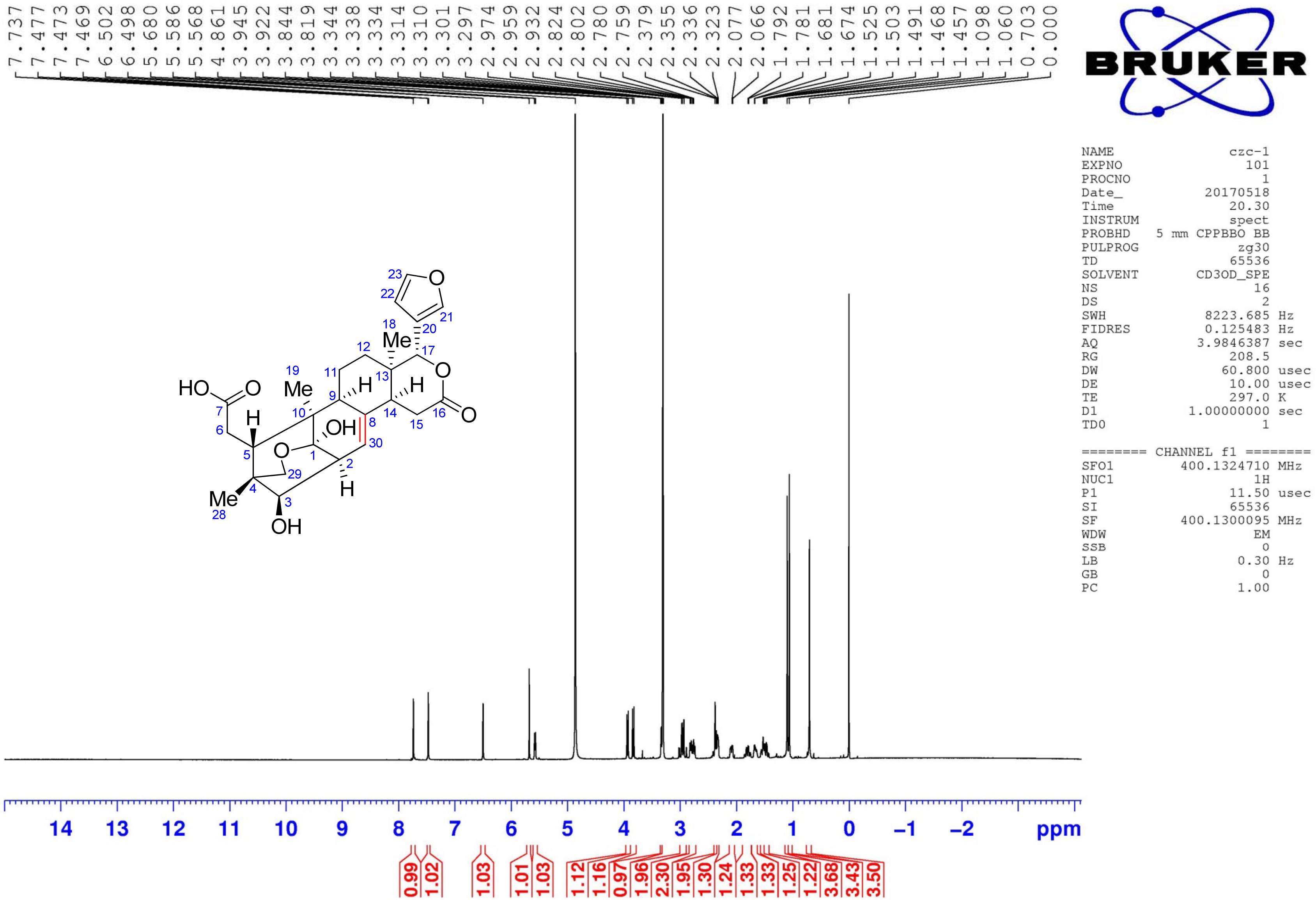

^{1}H NMR (400 MHz) spectrum of **7** in CD_3OD

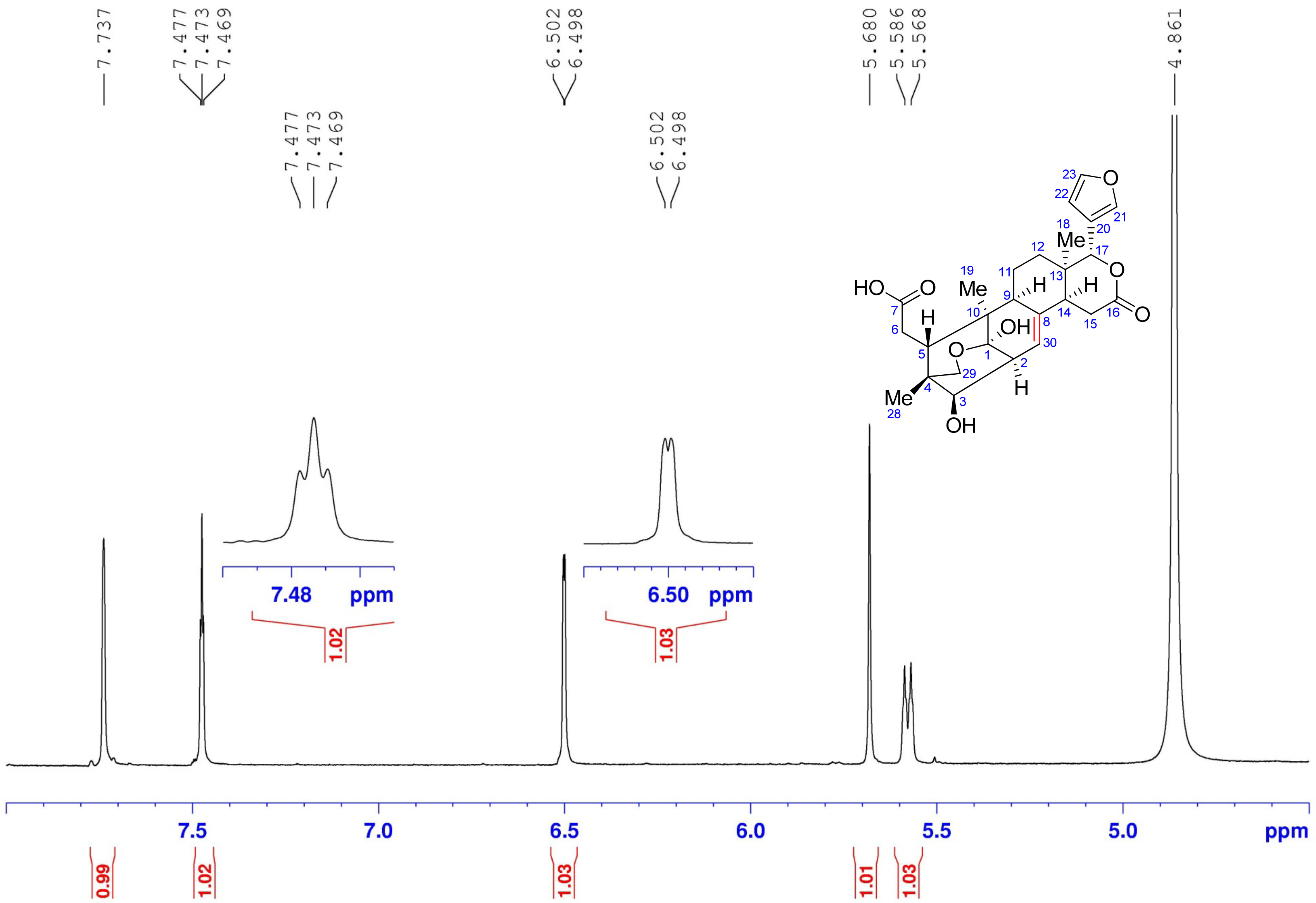

^{1}H NMR (400 MHz) spectrum of **7** in CD_3OD

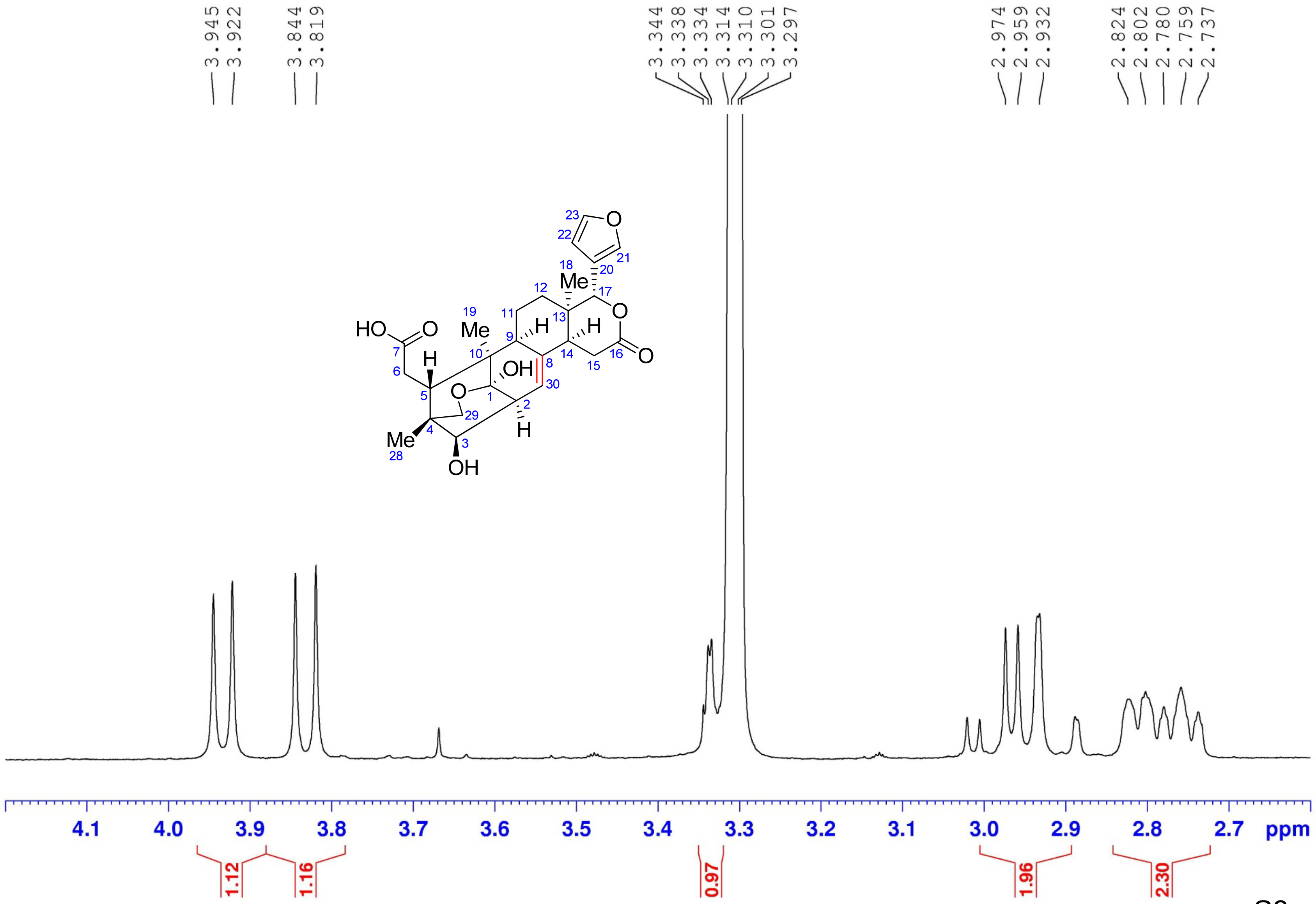

^{1}H NMR (400 MHz) spectrum of **7** in CD_3OD

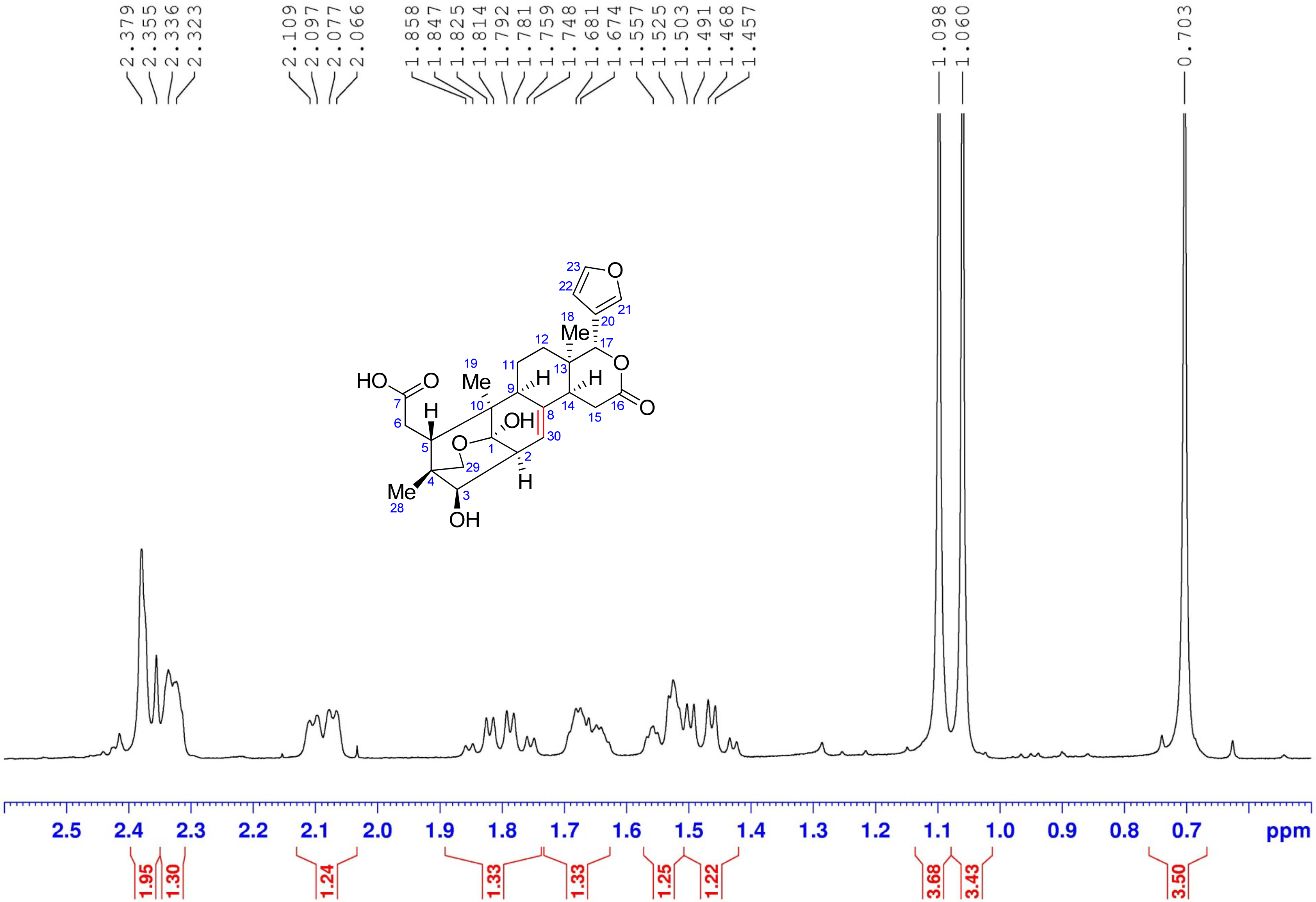

^{13}C NMR (100 MHz) spectrum of **7** in CD_3OD

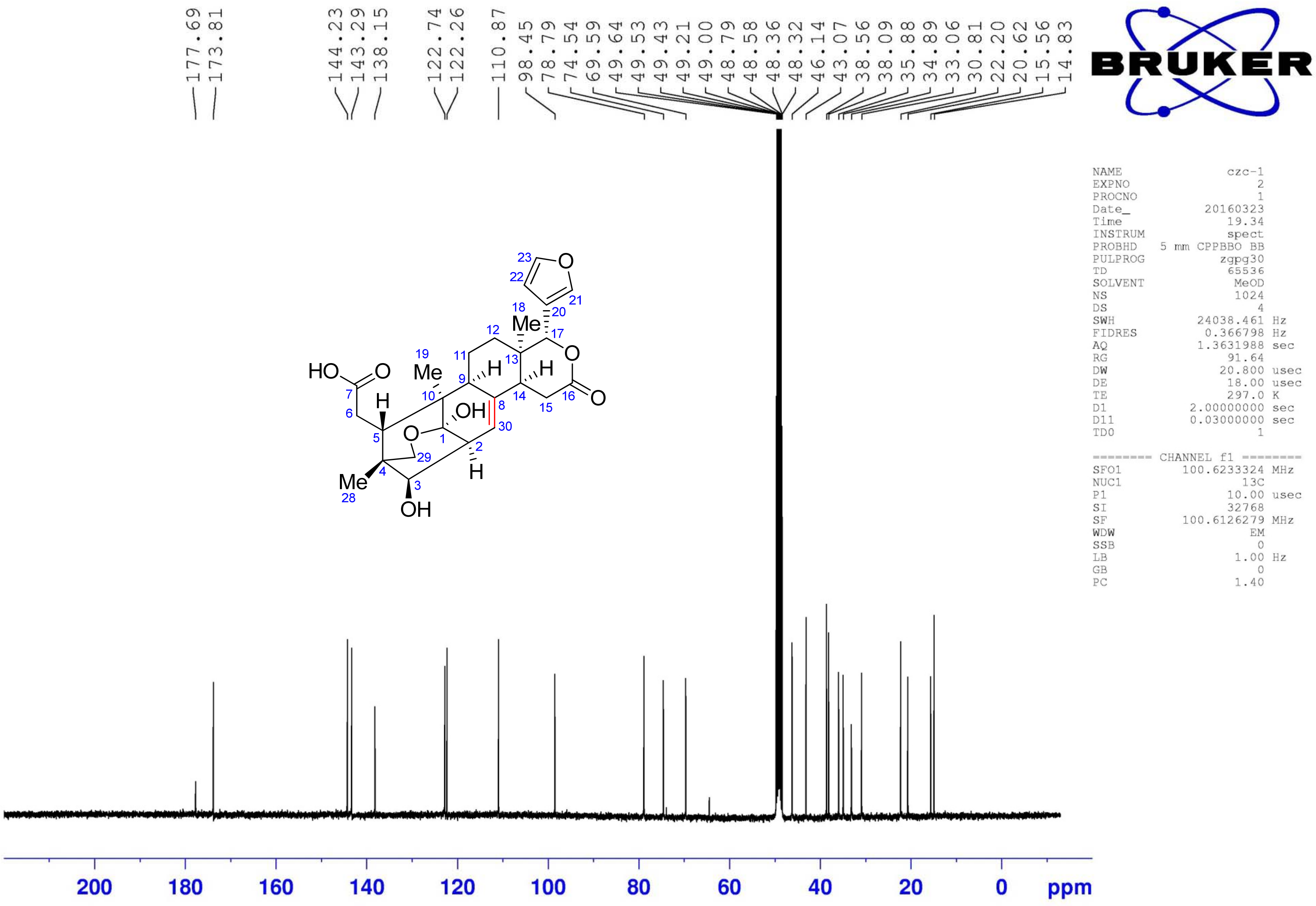

^{13}C NMR (100 MHz) spectrum of **7** in CD_3OD

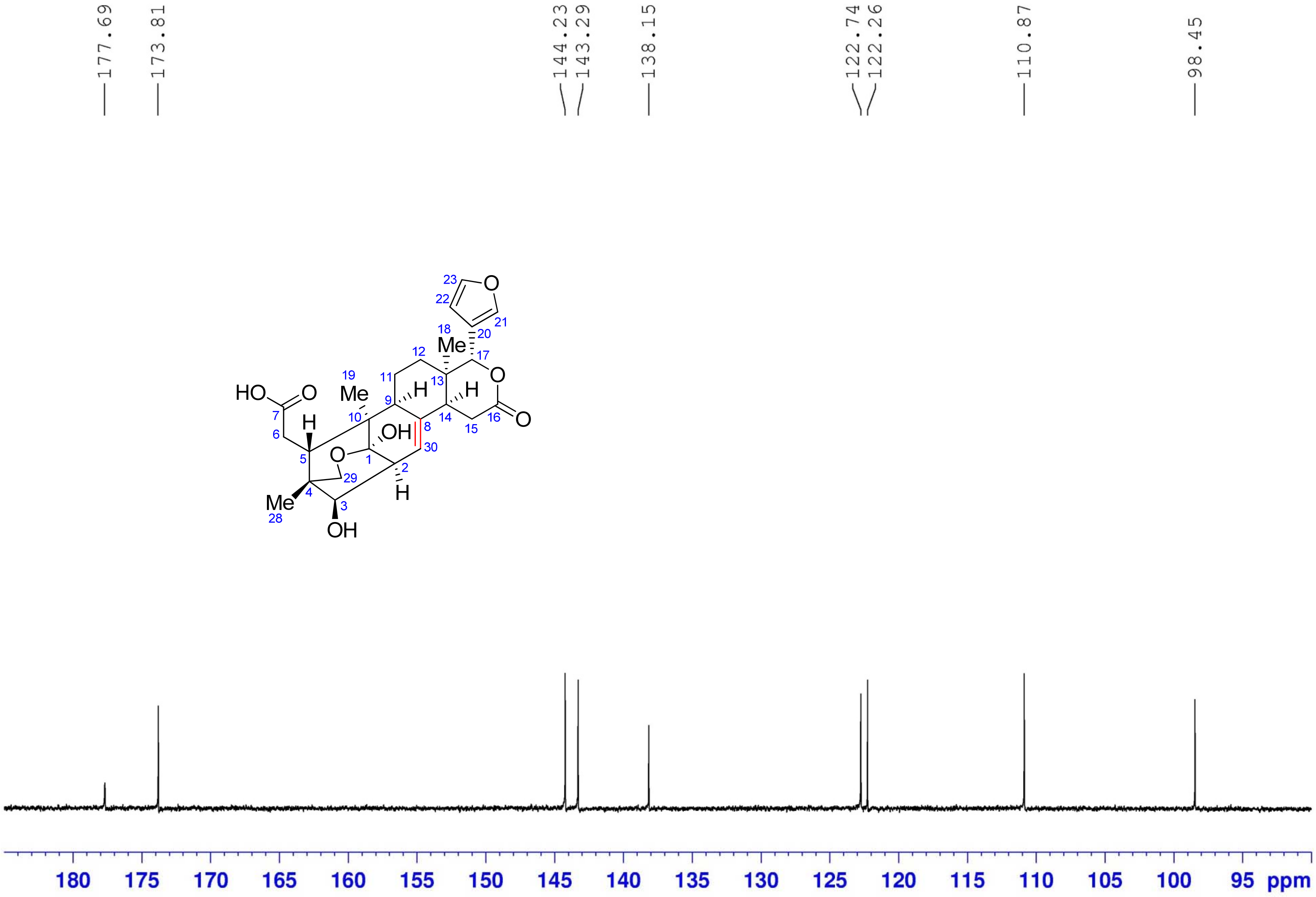

^{13}C NMR (100 MHz) spectrum of **7** in CD_3OD

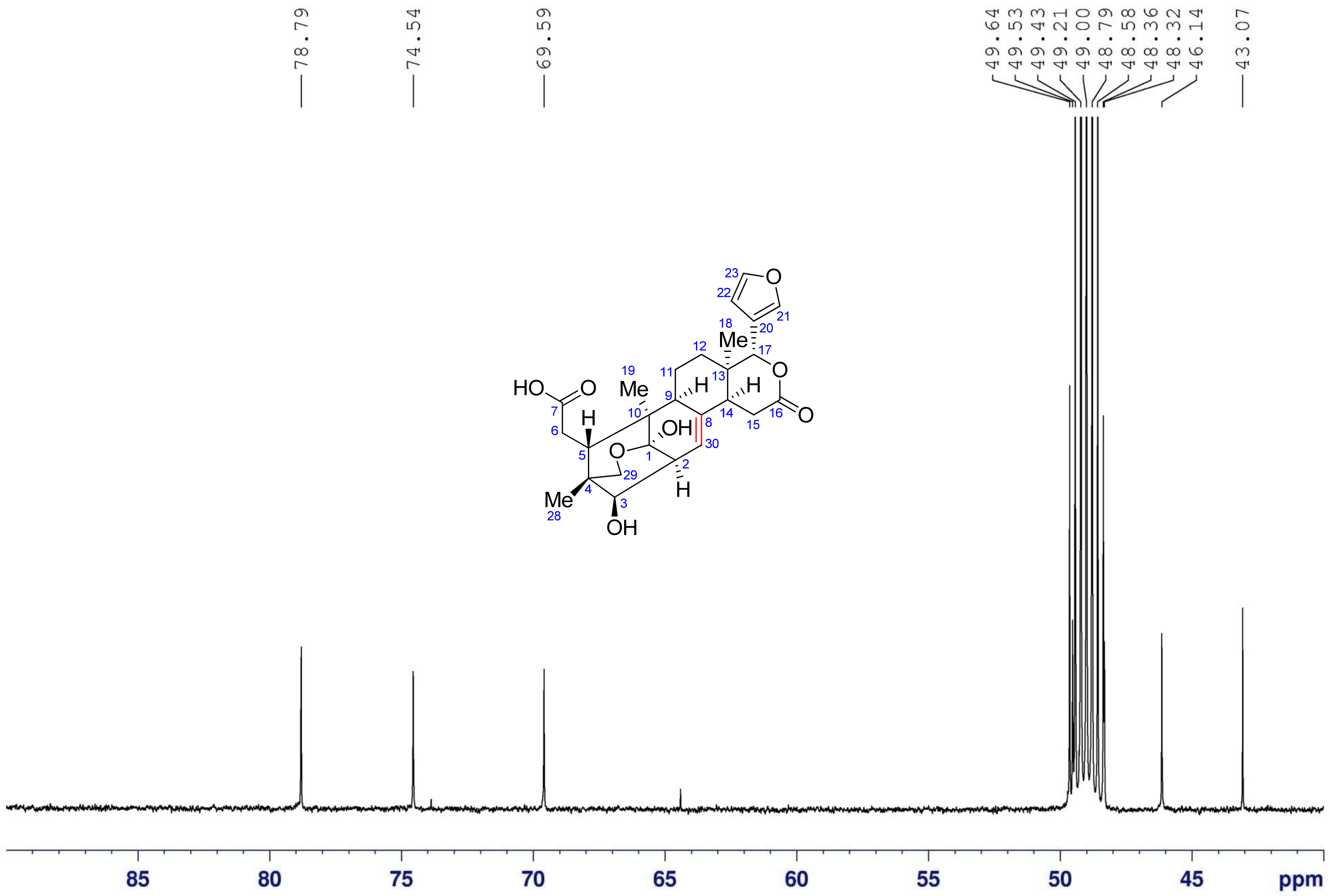

^{13}C NMR (100 MHz) spectrum of **7** in CD_3OD

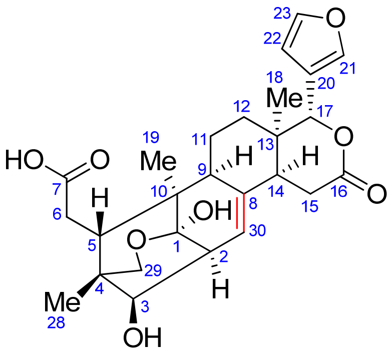

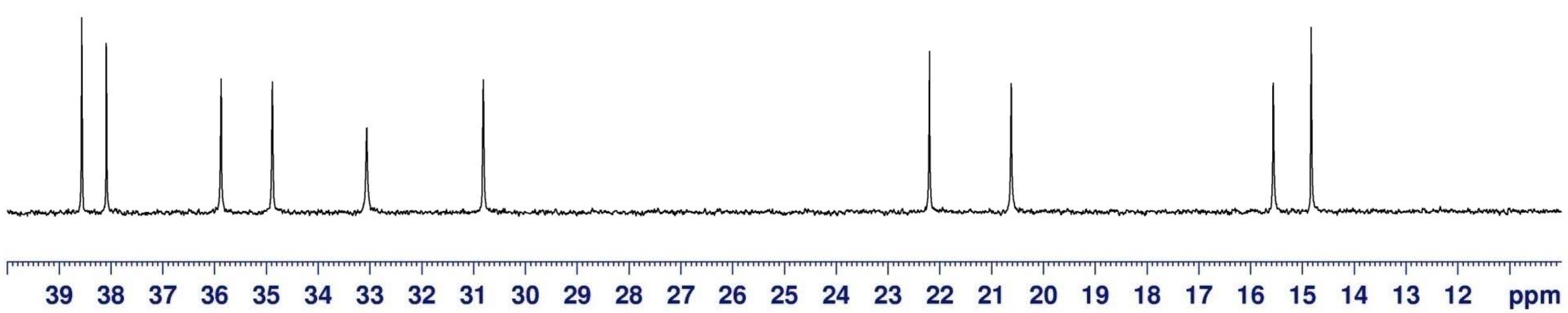

DEPT 135 spectrum of **7** in CD_3OD

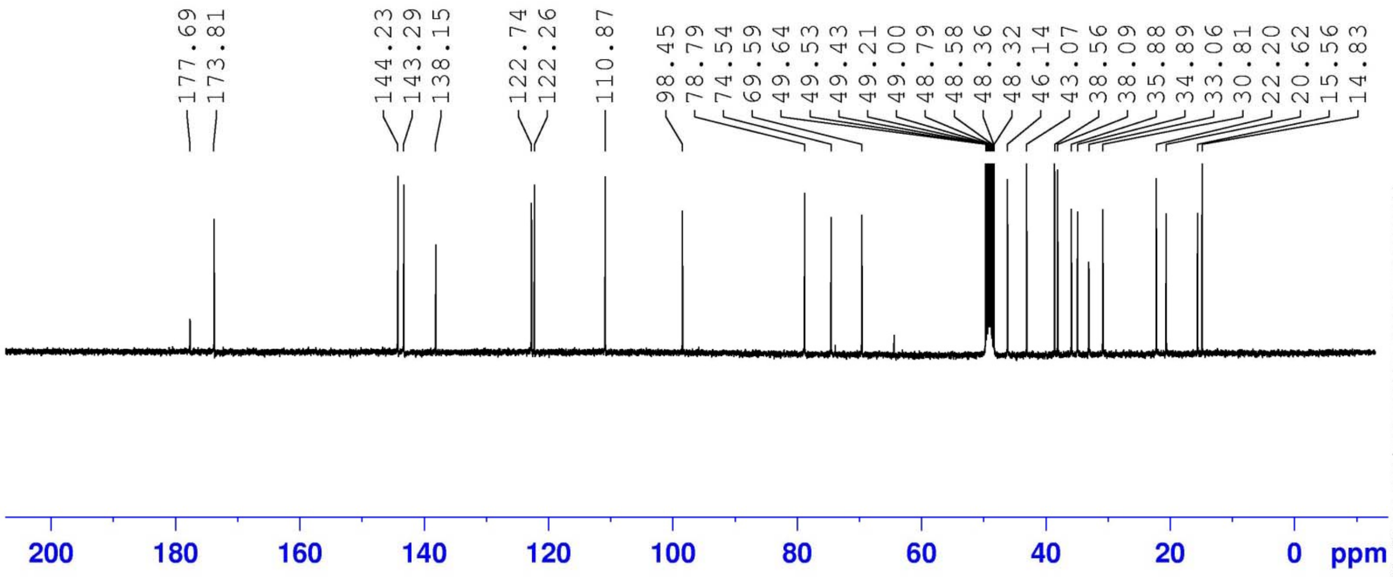

NAME	czc-1
EXPNO	3
PROCNO	1
Date_	20160323
Time	19.52
INSTRUM	spect
PROBHD	5 mm CPPBBO BB
PULPROG	deptsp135
TD	65536
SOLVENT	MeOD
NS	300
DS	4
SWH	24038.461 Hz
FIDRES	0.366798 Hz
AQ	1.3631988 sec
RG	130.26
DW	20.800 usec
DE	18.00 usec
TE	297.0 K
CNST2	145.0000000
D1	2.00000000 sec
D2	0.00344828 sec
D12	0.00002000 sec
TD0	1

======== CHANNEL f1 ========

SFO1	100.6233324 MHz
NUC1	13C
P1	10.00 usec
P13	2000.00 usec
SI	32768
SF	100.6126286 MHz
WDW	EM
SSB	0
LB	1.00 Hz
GB	0
PC	1.40

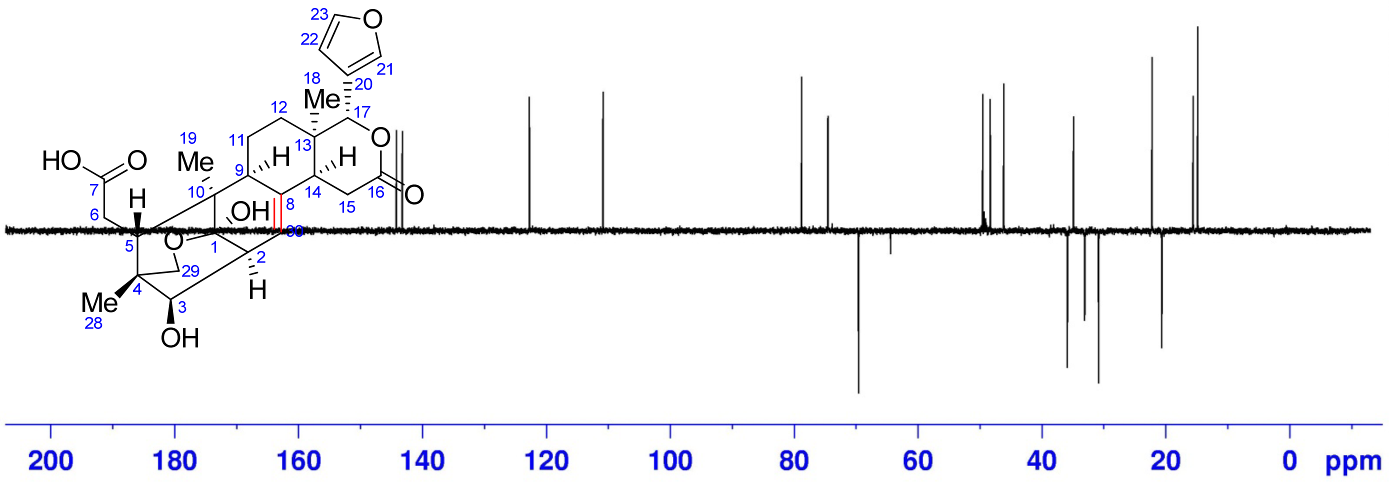

DEPT 135 spectrum of **7** in CD_3OD

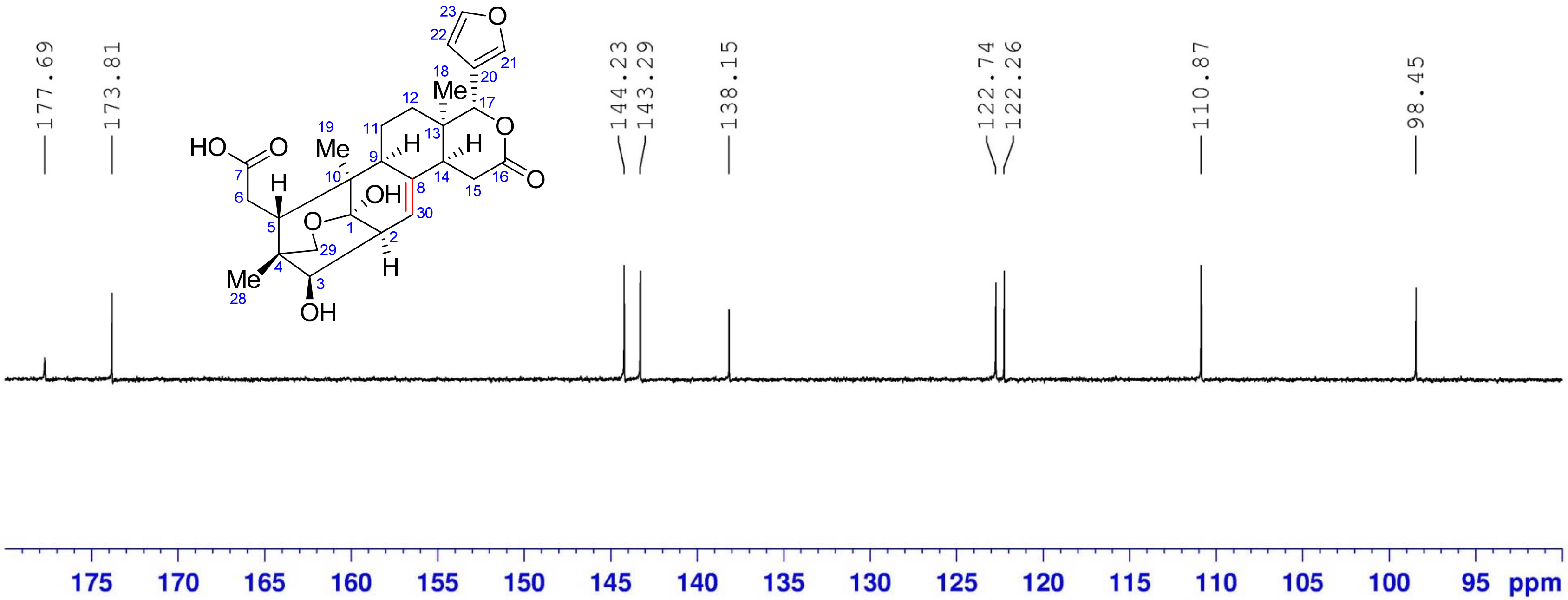

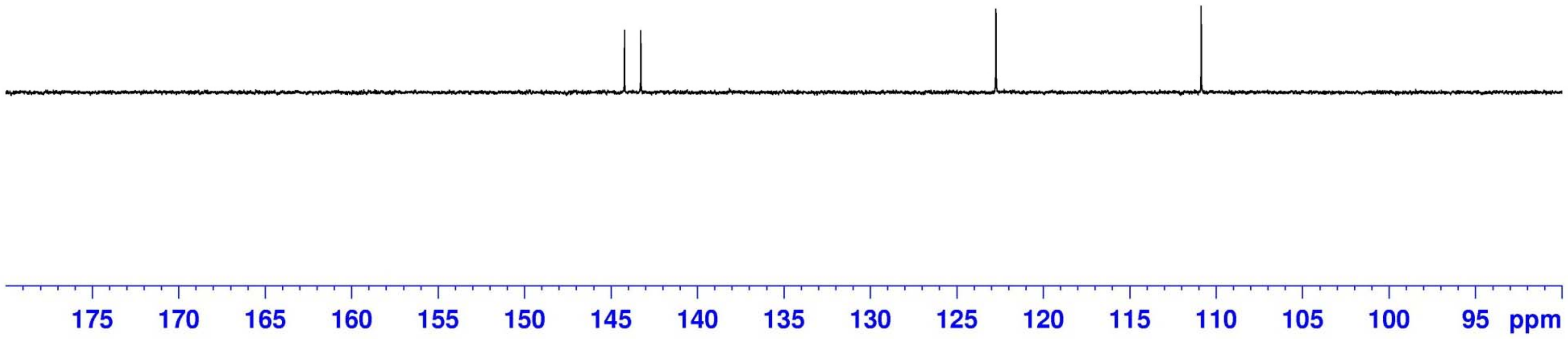

DEPT 135 spectrum of **7** in CD_3OD

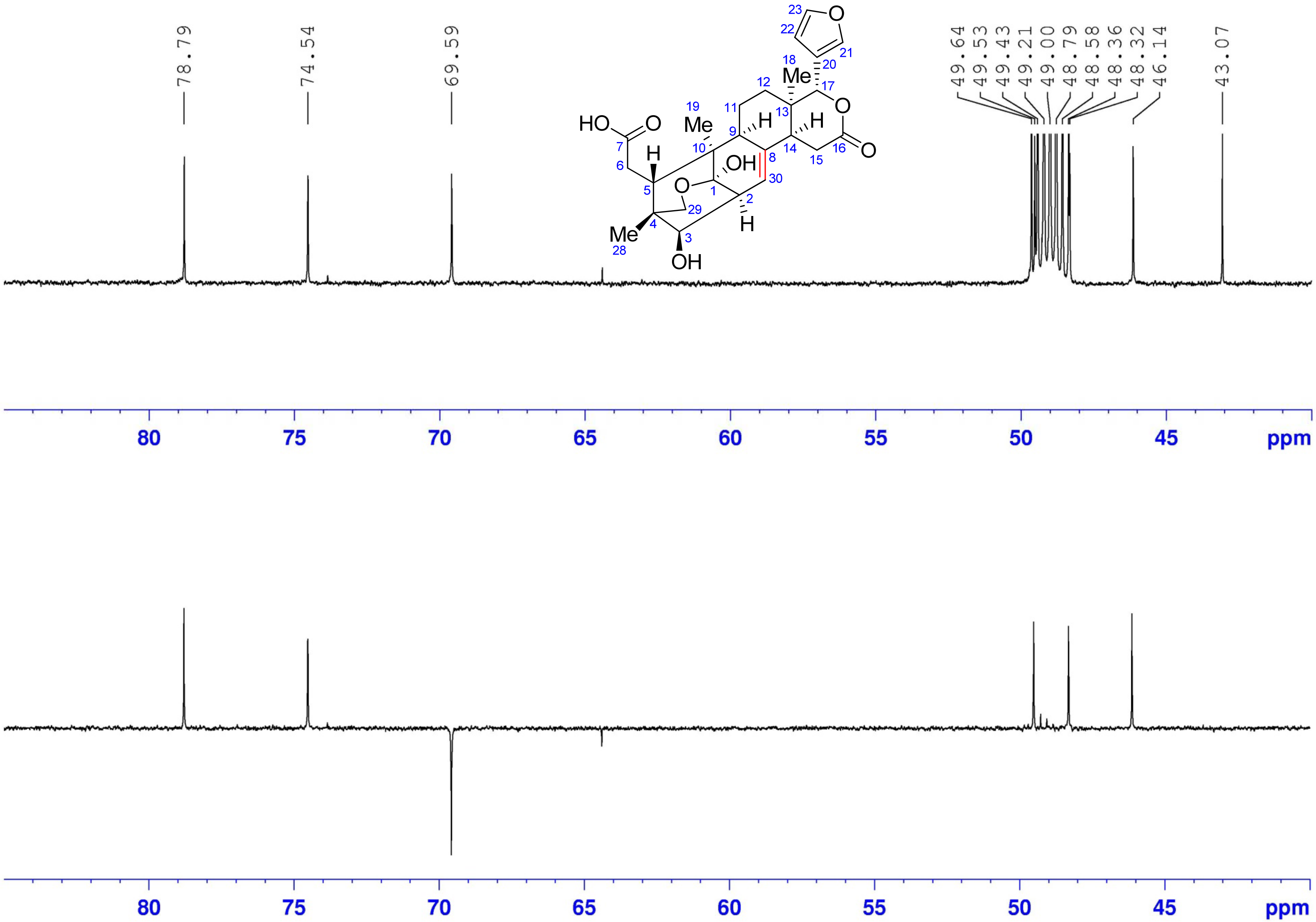

DEPT 135 spectrum of **7** in CD_3OD

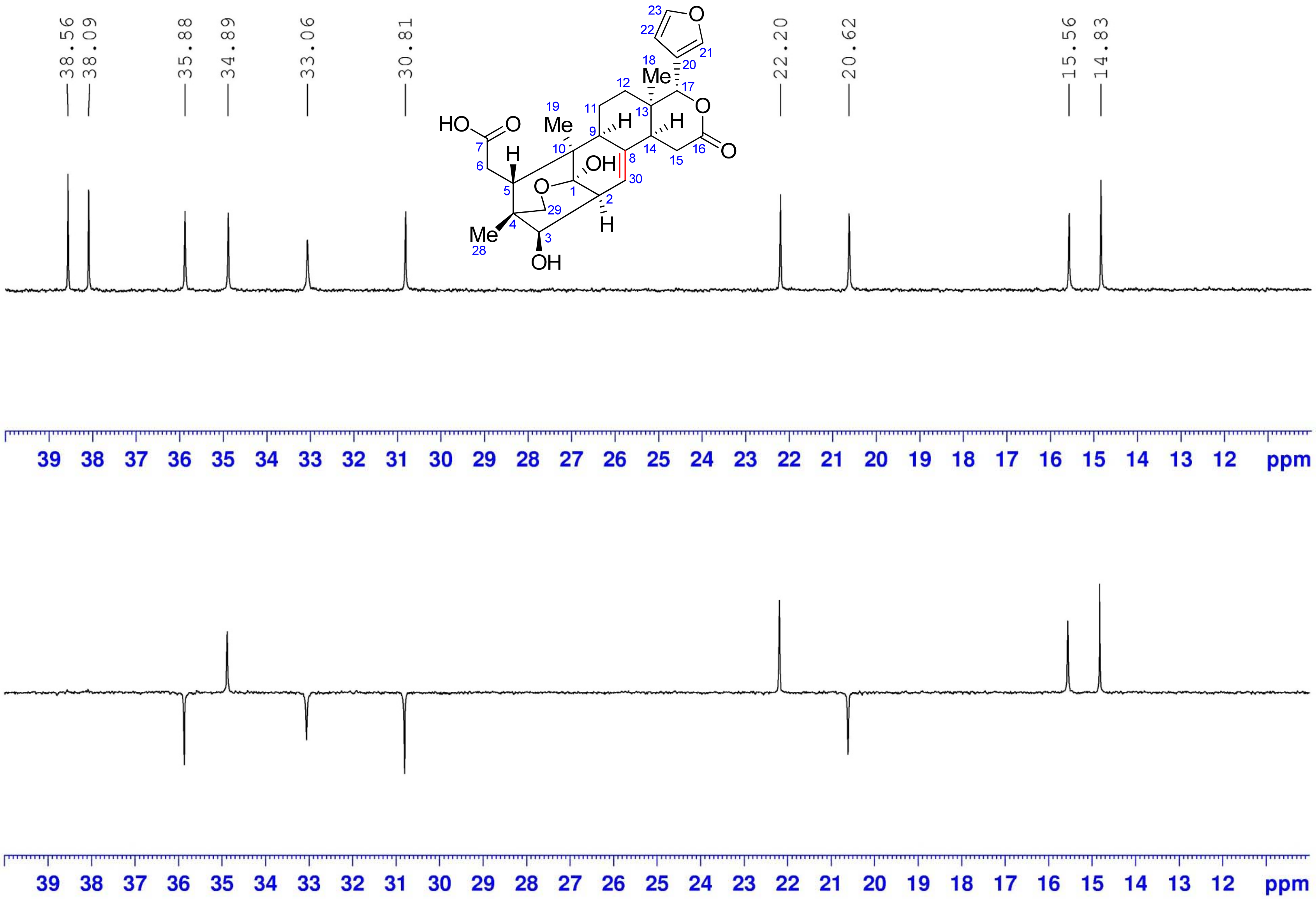

LR-ESIMS for compound **8**

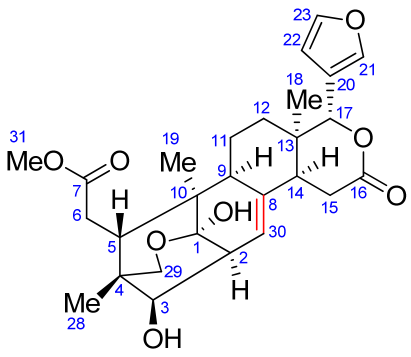

8

Chemical Formula: $C_{27}H_{34}O_8$
Exact Mass: 486.22537

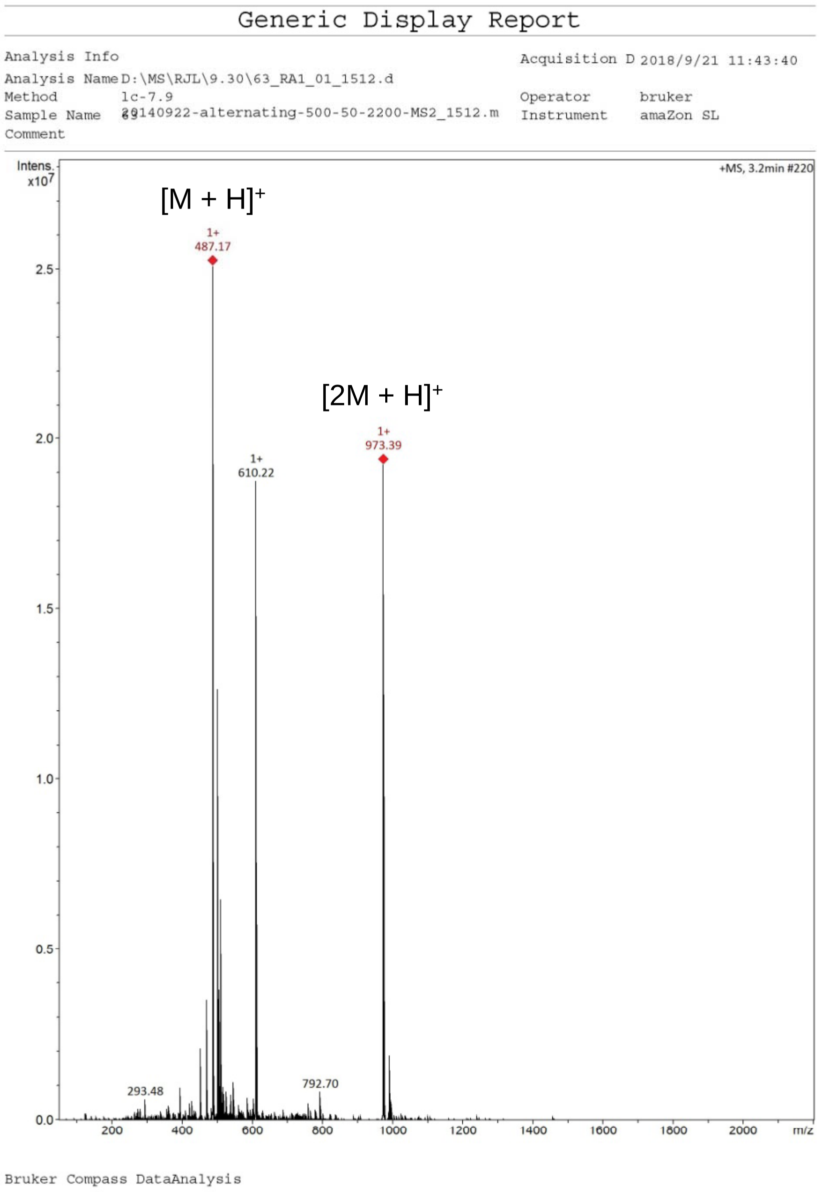

^1H NMR (400 MHz) spectrum of **8** in $CDCl_3$

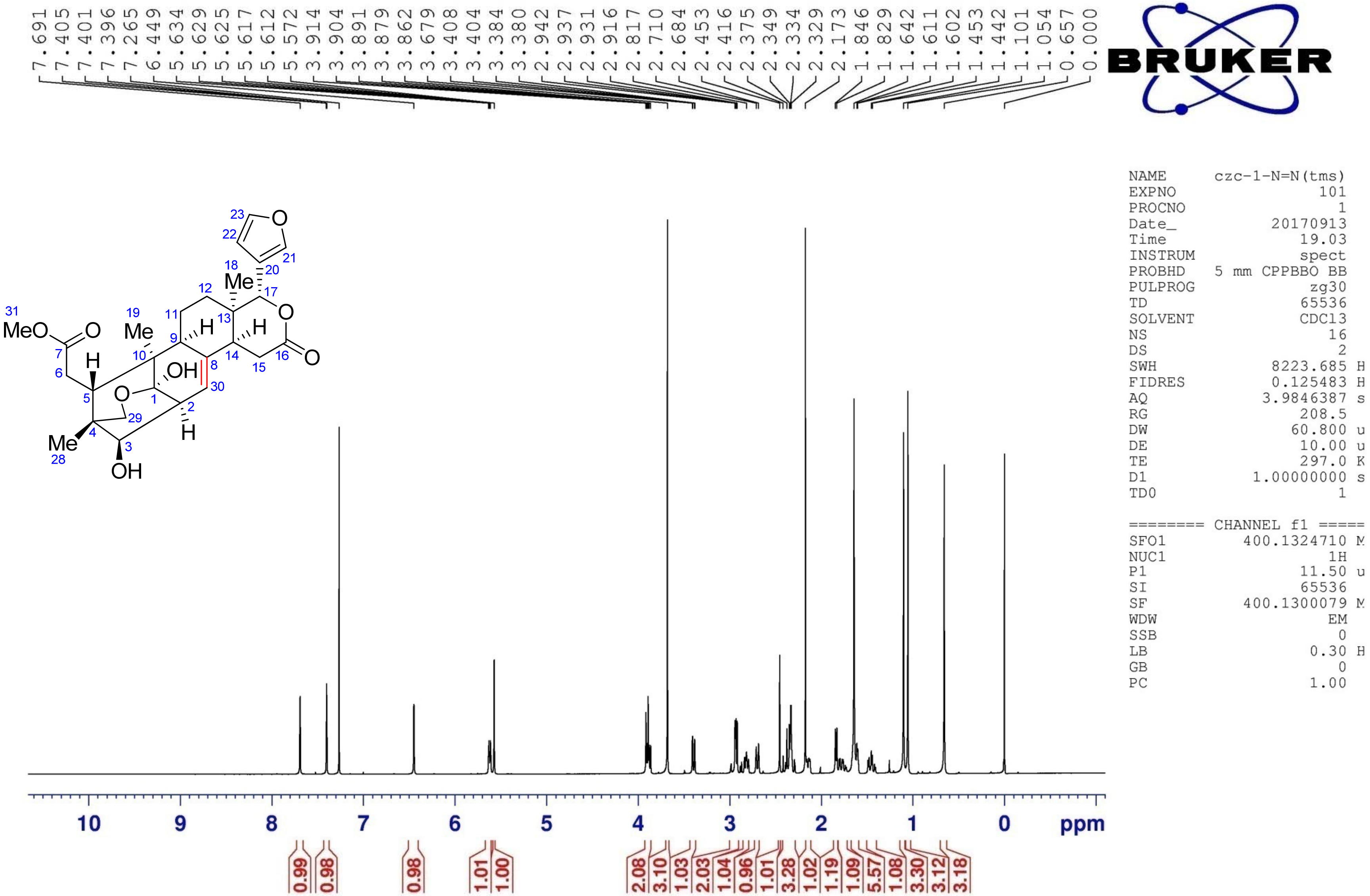

^{1}H NMR (400 MHz) spectrum of **8** in $CDCl_3$

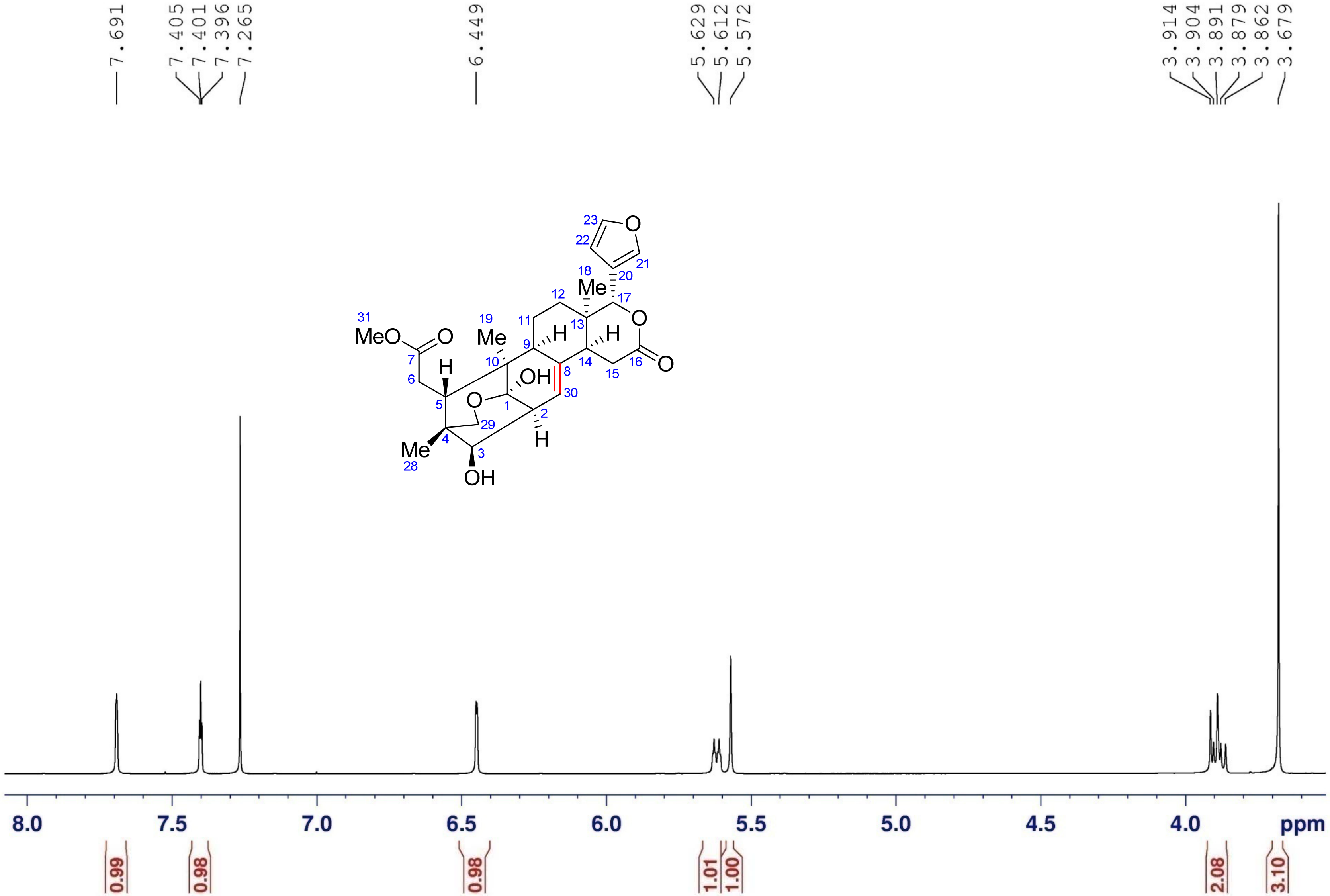

^{1}H NMR (400 MHz) spectrum of **8** in $CDCl_3$

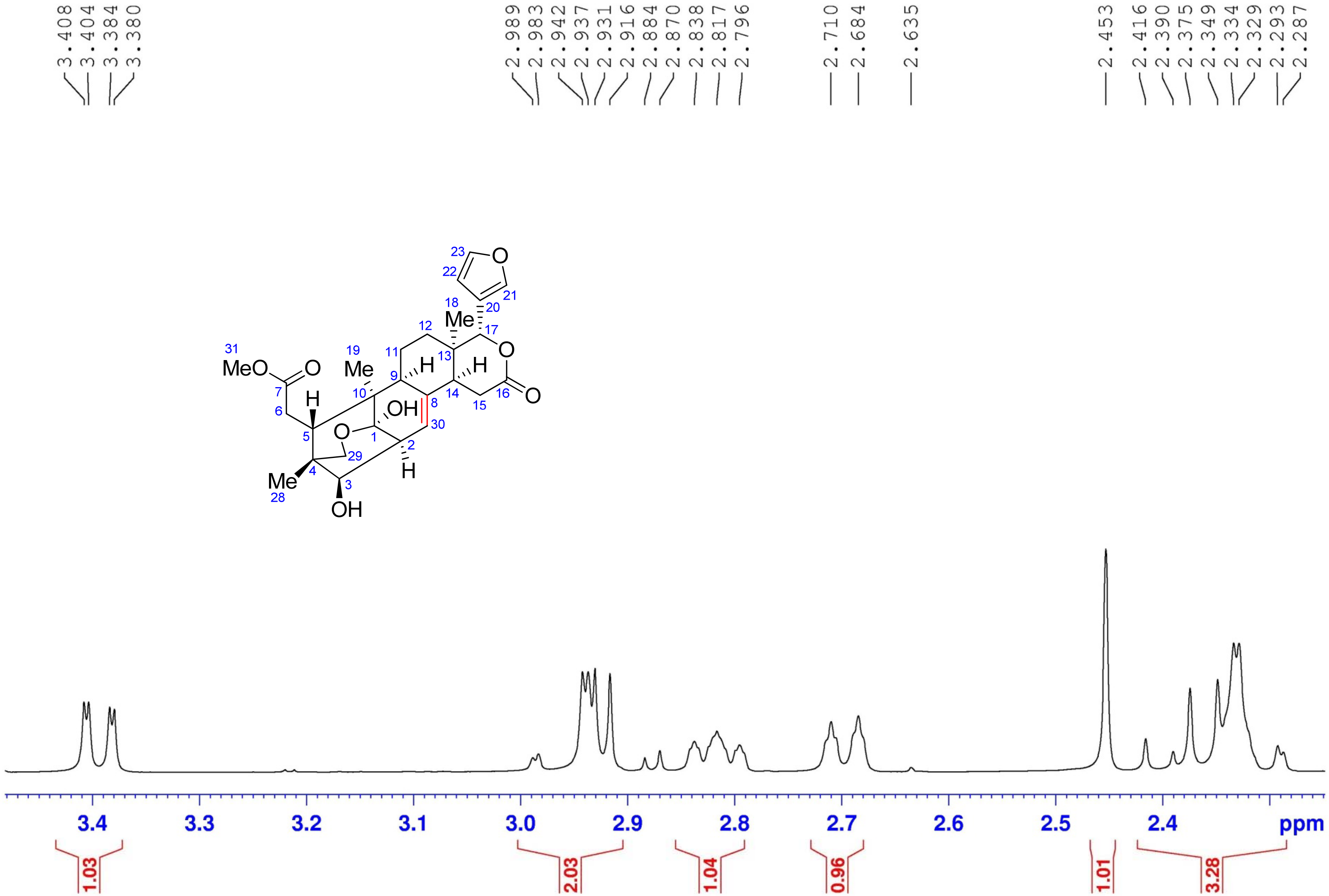

^{1}H NMR (400 MHz) spectrum of **8** in $CDCl_3$

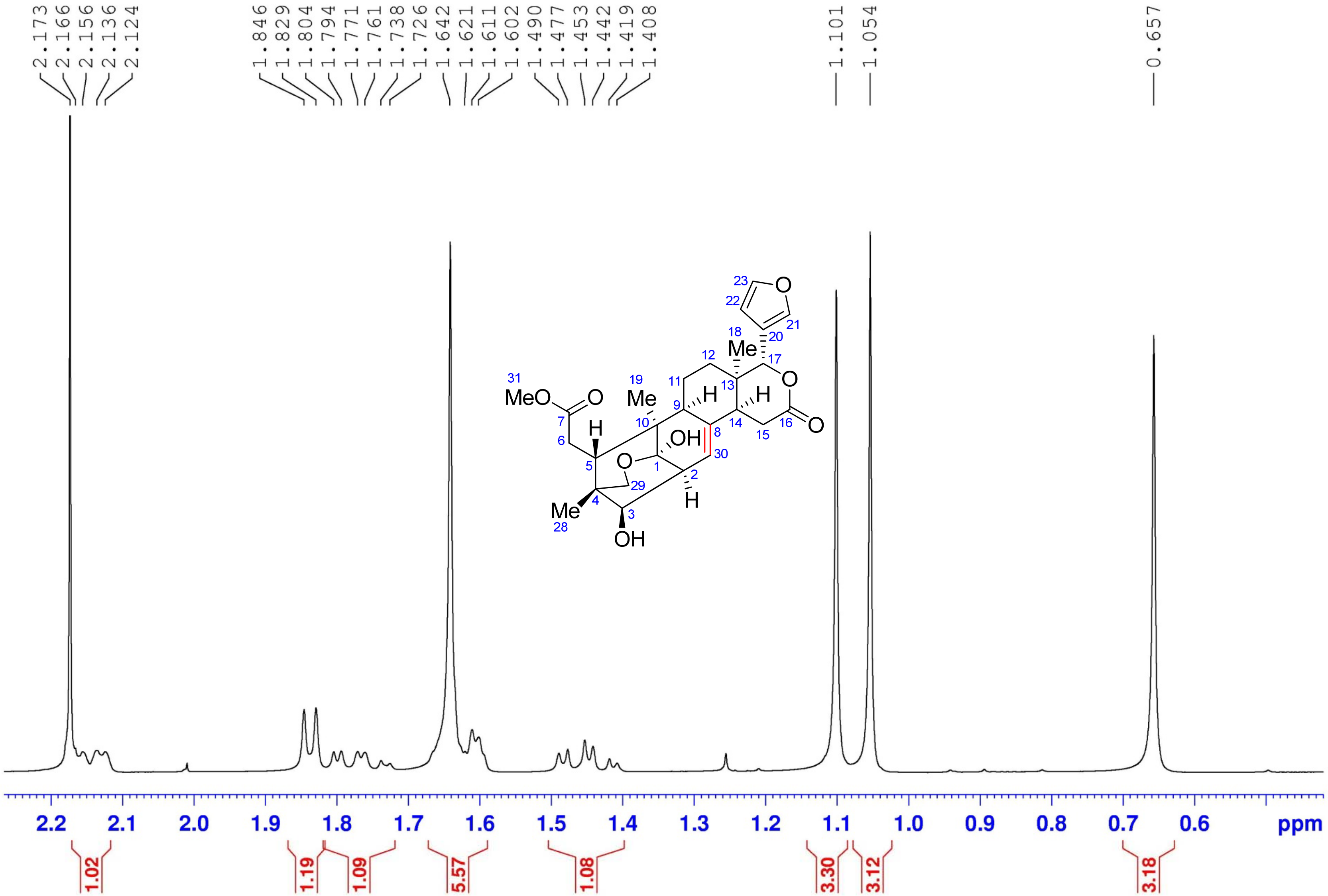

^{13}C NMR (100 MHz) spectrum of **8** in $CDCl_3$

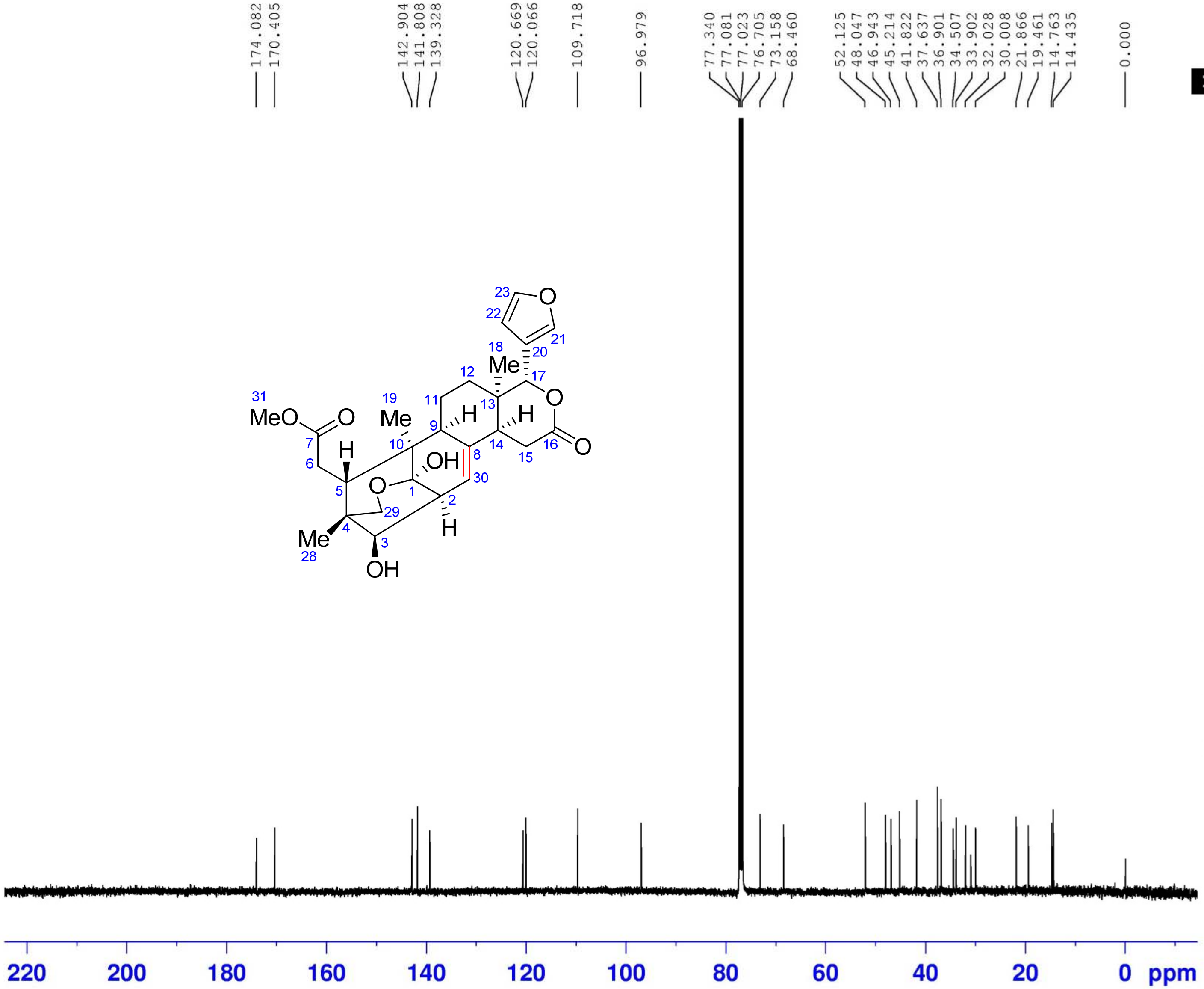

NAME	czc-1-N=N(tms)
EXPNO	2
PROCNO	1
Date_	20170905
Time	20.18
INSTRUM	spect
PROBHD	5 mm CPPBBO BB
PULPROG	zgpg30
TD	65536
SOLVENT	CDCl3
NS	1024
DS	4
SWH	24038.461 H
FIDRES	0.366798 H
AQ	1.3631988 s
RG	85.34
DW	20.800 u
DE	18.00 u
TE	297.0 K
D1	2.00000000 s
D11	0.03000000 s
TD0	1
======== CHANNEL f1 =====	
SFO1	100.6233324 M
NUC1	13C
P1	10.00 u
SI	32768
SF	100.6127690 M
WDW	EM
SSB	0
LB	1.00 H
GB	0
PC	1.40

^{13}C NMR (100 MHz) spectrum of **8** in $CDCl_3$

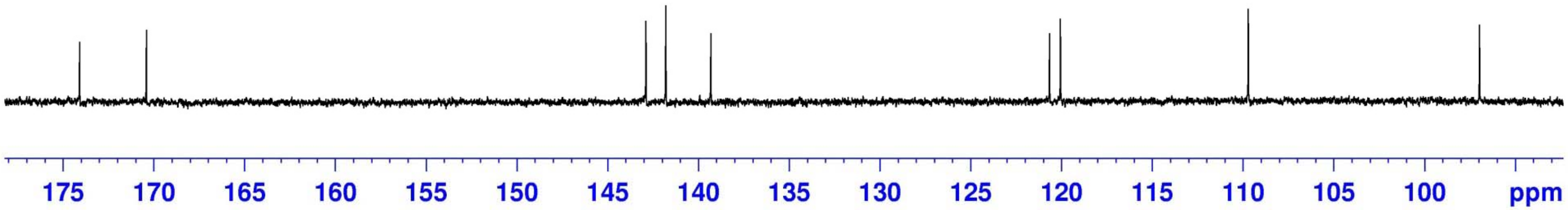

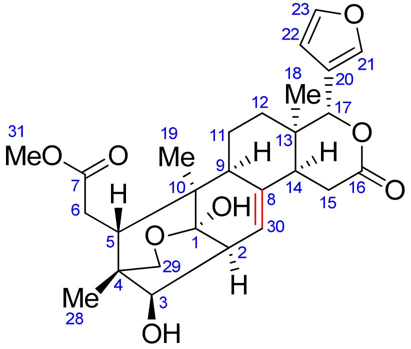

^{13}C NMR (100 MHz) spectrum of **8** in $CDCl_3$

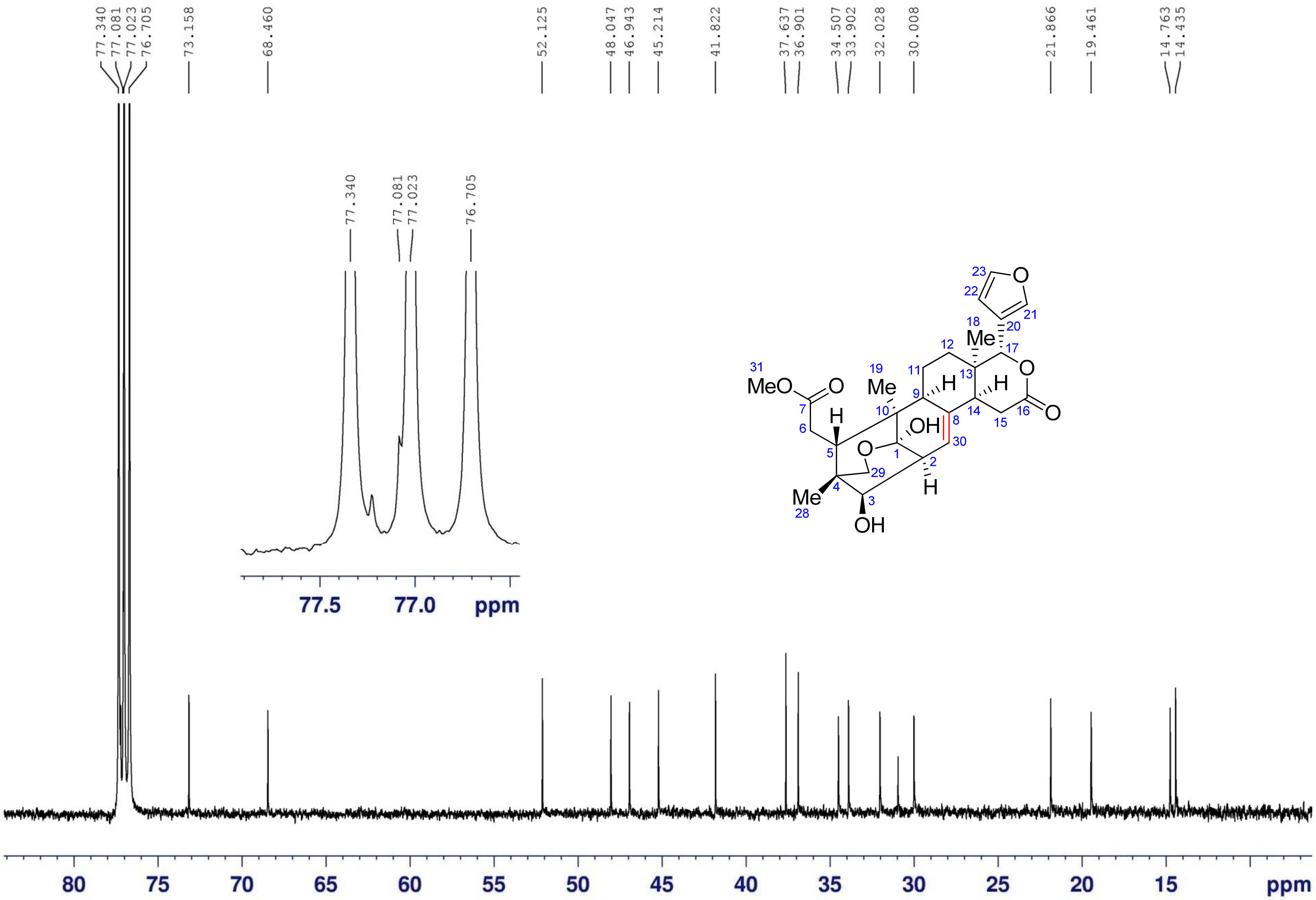

DEPT 135 spectrum of **8** in $CDCl_3$

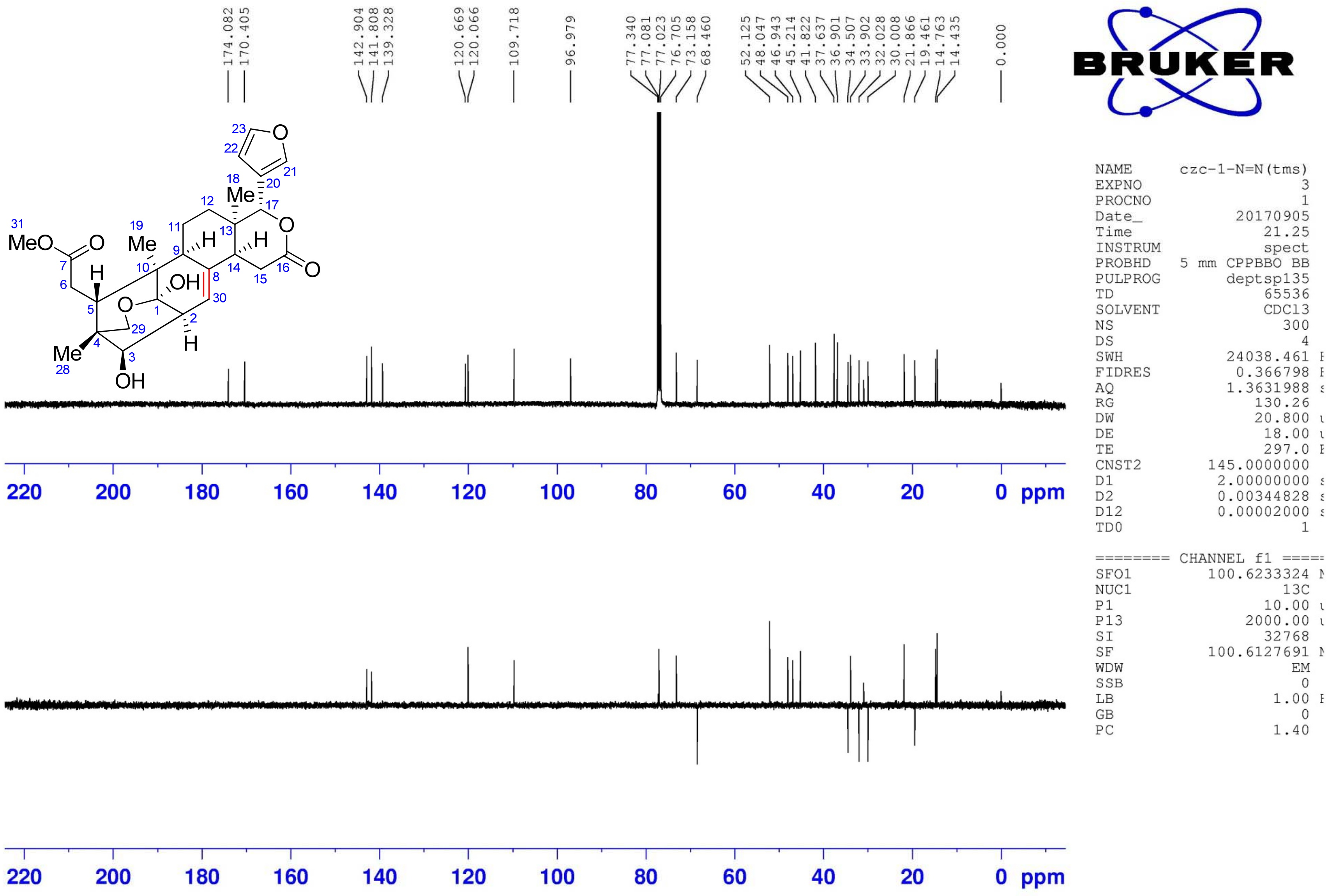

DEPT 135 spectrum of **8** in $CDCl_3$

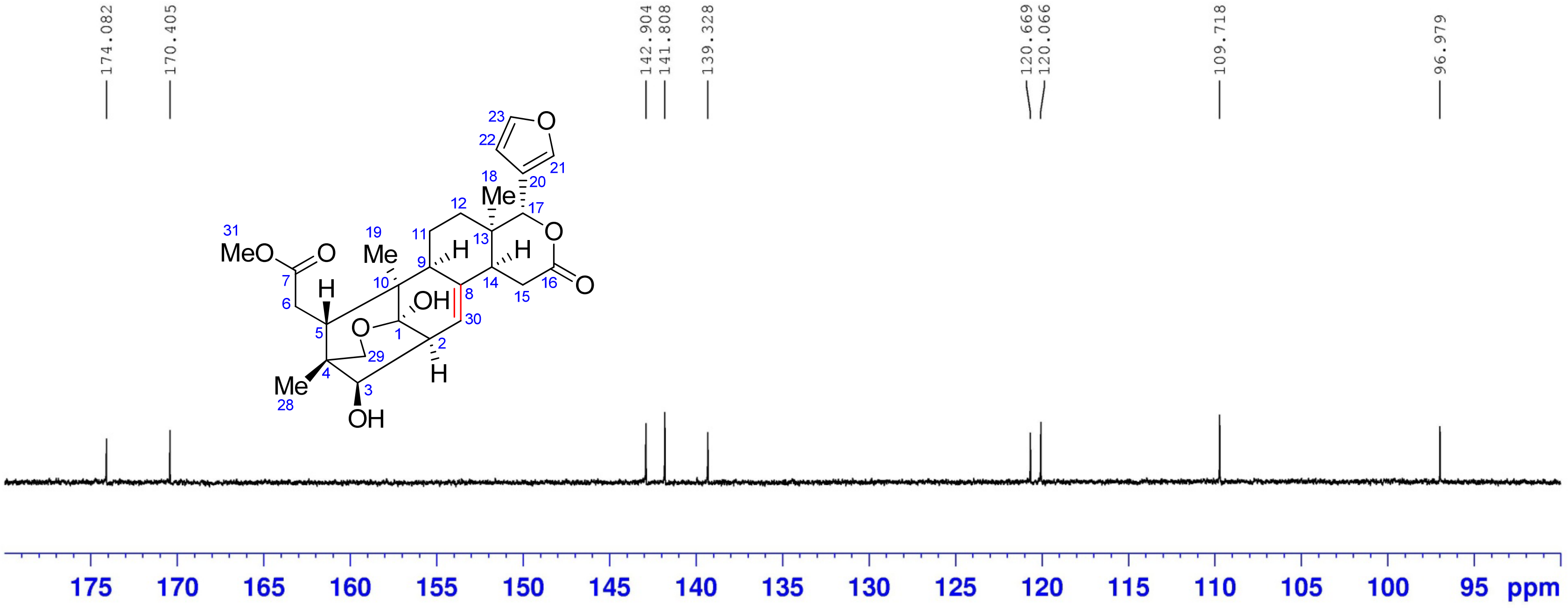

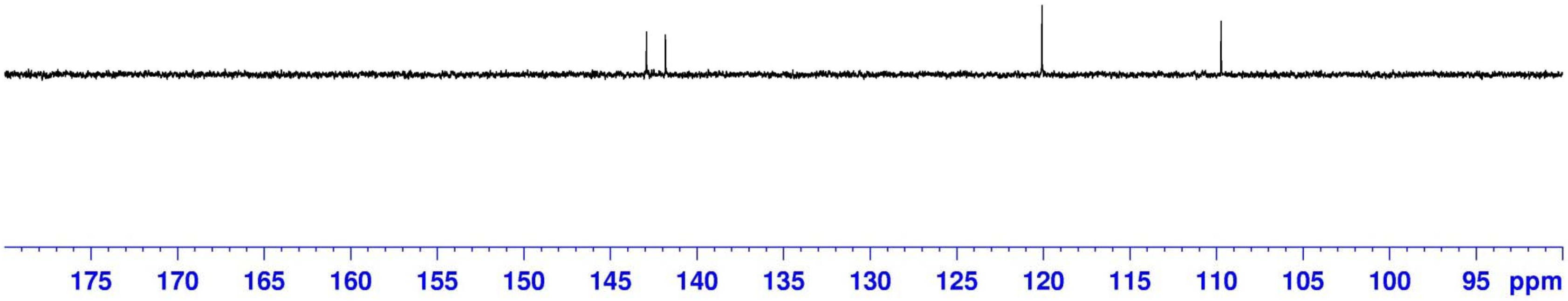

DEPT 135 spectrum of **8** in $CDCl_3$

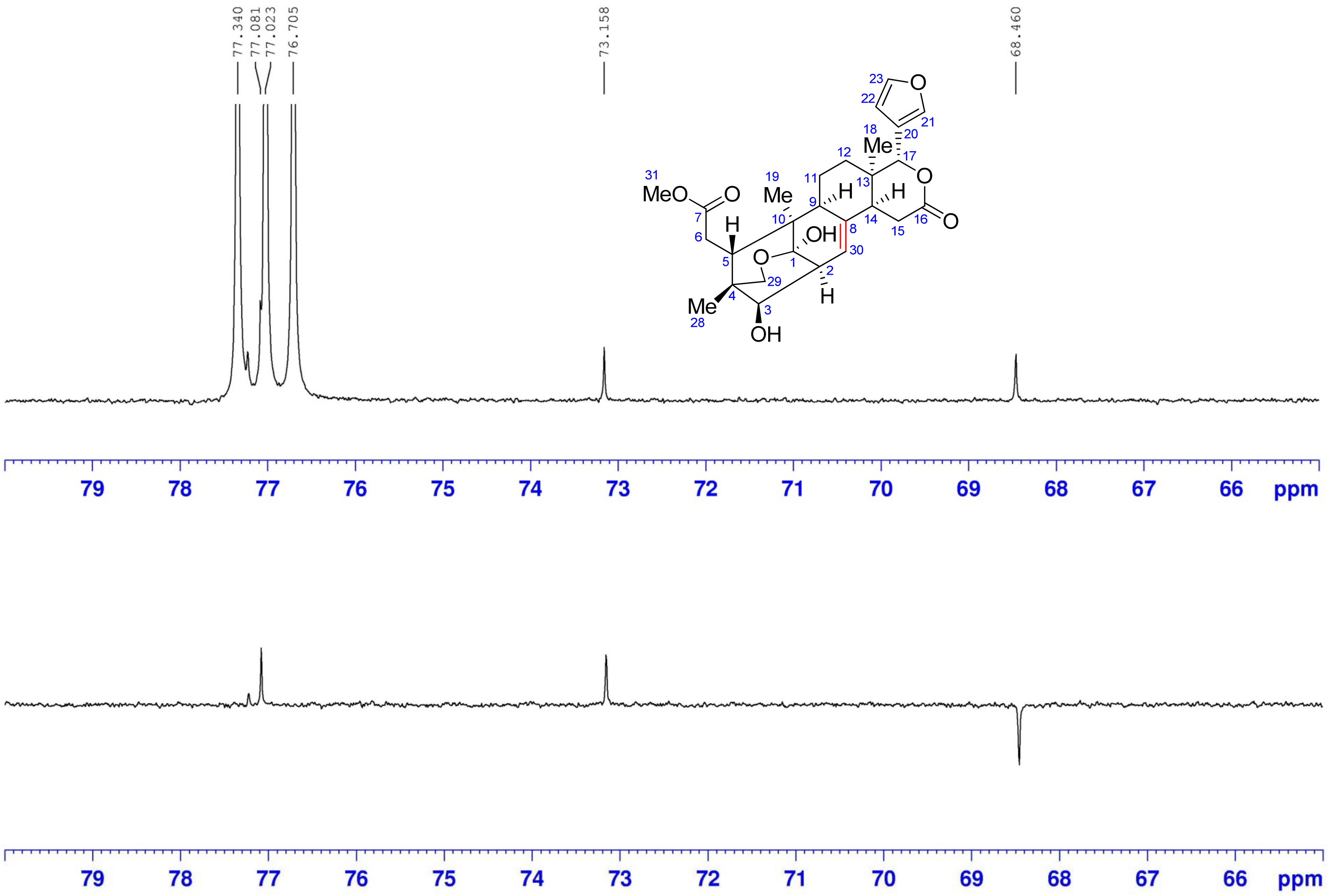

DEPT 135 spectrum of **8** in $CDCl_3$

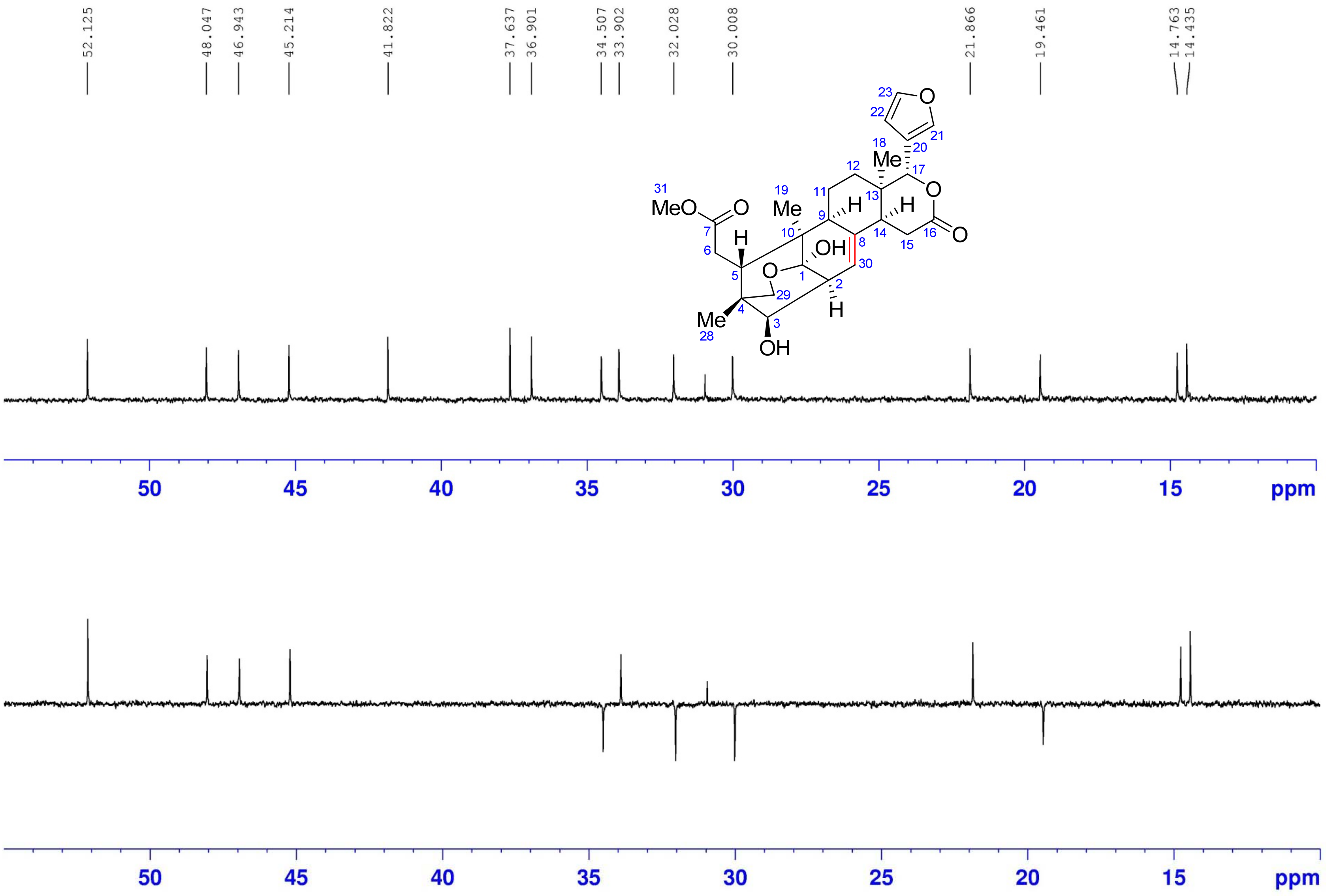

HR-ESIMS for compound **8a**

Generic Display Report

Analysis Info

Analysis Name	D:\Data\MS\data\201809\liwanshan_WSD-72_pos_18_01_5579.d	Acquisition Date	9/30/2018 6:19:21 PM
Method	LC_Direct Infusion_pos_100-1000mz.m	Operator	SCSIO
Sample Name	liwanshan_WSD-72_pos	Instrument	maXis
Comment			

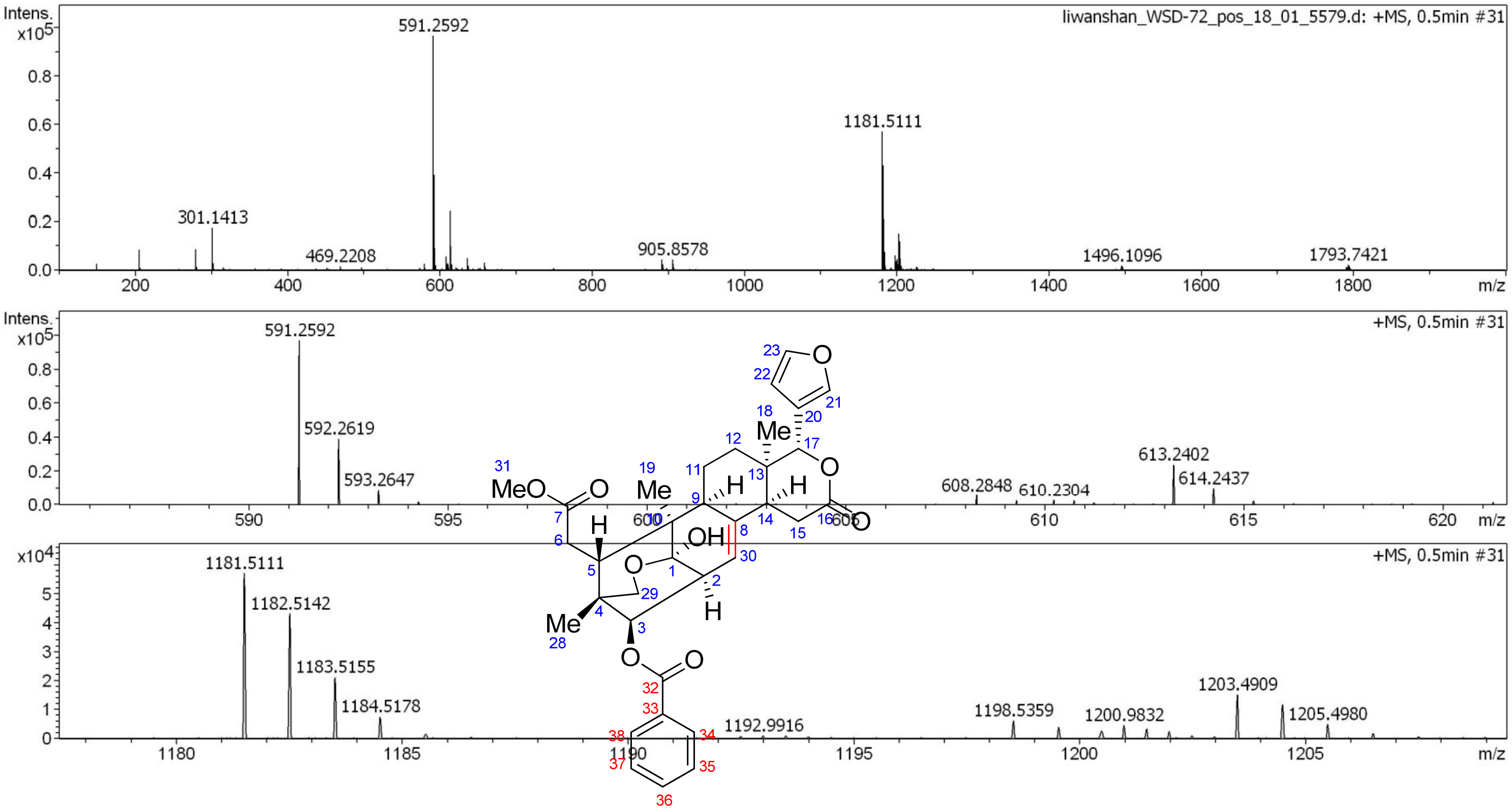

HR-ESIMS for compound **8a**

Mass Spectrum SmartFormula Report

Analysis Info

Analysis Name	D:\Data\MS\data\201809\liwanshan_WSD-72_pos_18_01_5579.d	Acquisition Date	9/30/2018 6:19:21 PM
Method	LC_Direct Infusion_pos_100-1000mz.m	Operator	SCSIO
Sample Name	liwanshan_WSD-72_pos	Instrument	maXis 255552.00029
Comment			

Acquisition Parameter

Source Type	ESI	Ion Polarity	Positive	Set Nebulizer	0.4 Bar
Focus	Active	Set Capillary	4500 V	Set Dry Heater	180 °C
Scan Begin	100 m/z	Set End Plate Offset	-500 V	Set Dry Gas	4.0 l/min
Scan End	2000 m/z	Set Charging Voltage	0 V	Set Divert Valve	Waste
		Set Corona	0 nA	Set APCI Heater	0 °C

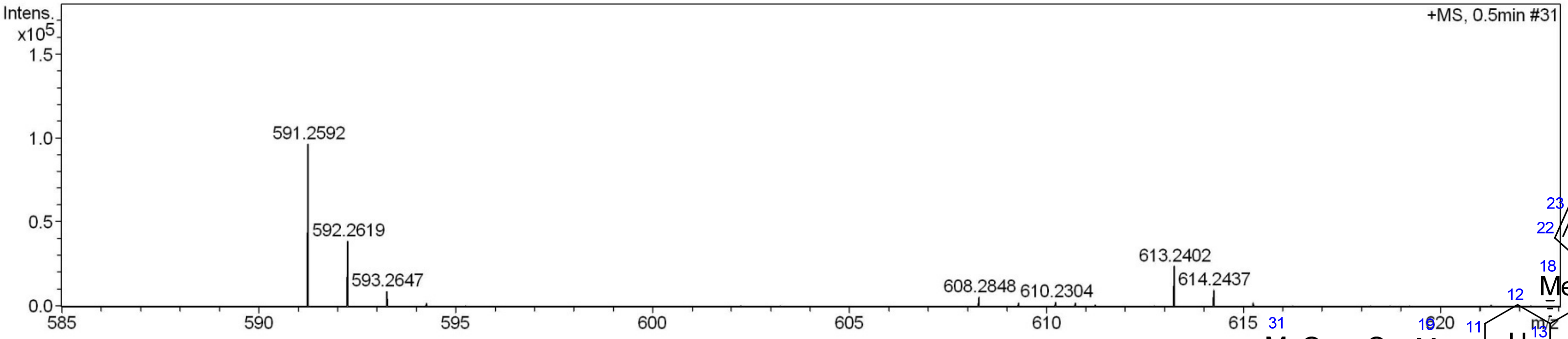

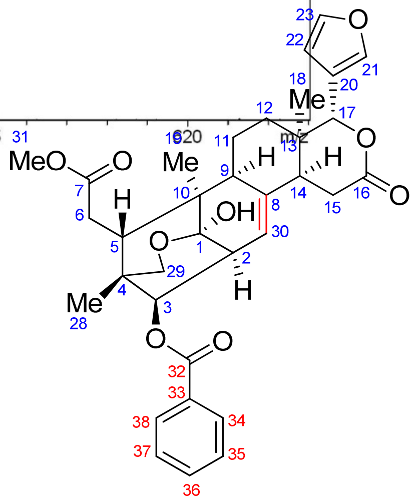

Meas. m/z	#	Ion Formula	Score	m/z	err [ppm]	err [mDa]	mSigma	rdb	e⁻ Conf	N-Rule
591.2592	1	C34H39O9	100.00	591.2589	0.6	0.4	15.4	15.5	even	ok
613.2402	1	C34H38NaO9	100.00	613.2408	1.1	0.6	17.7	15.5	even	ok
1181.5111	1	C68H77O18	100.00	1181.5104	0.5	0.6	27.1	30.5	even	ok
1203.4909	1	C68H76NaO18	100.00	1203.4924	1.3	1.5	15.7	30.5	even	ok

liwanshan_WSD-72_pos_18_01_5579.d

^{1}H NMR (400 MHz) spectrum of **8a** in $CDCl_3$

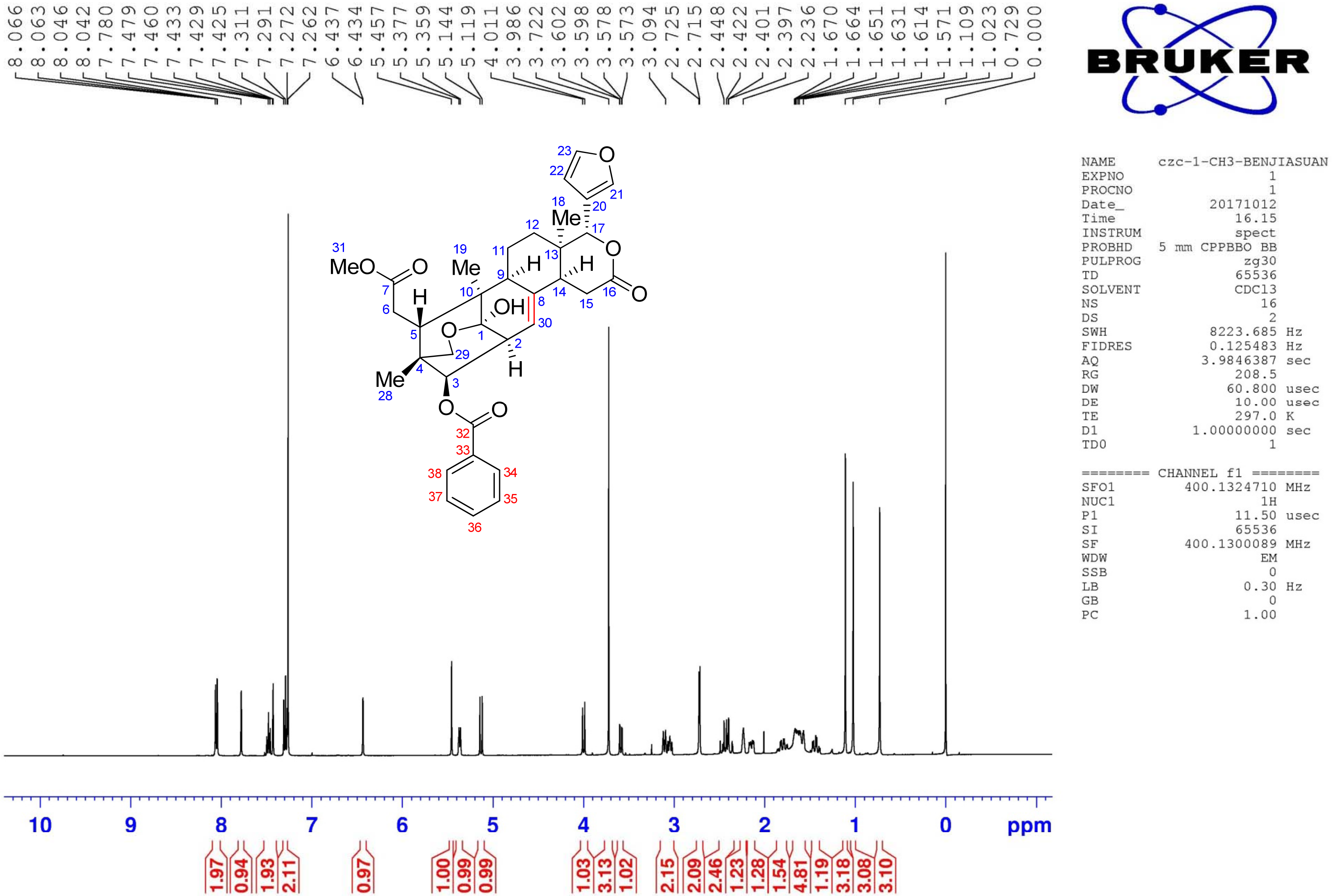

^{1}H NMR (400 MHz) spectrum of **8a** in $CDCl_3$

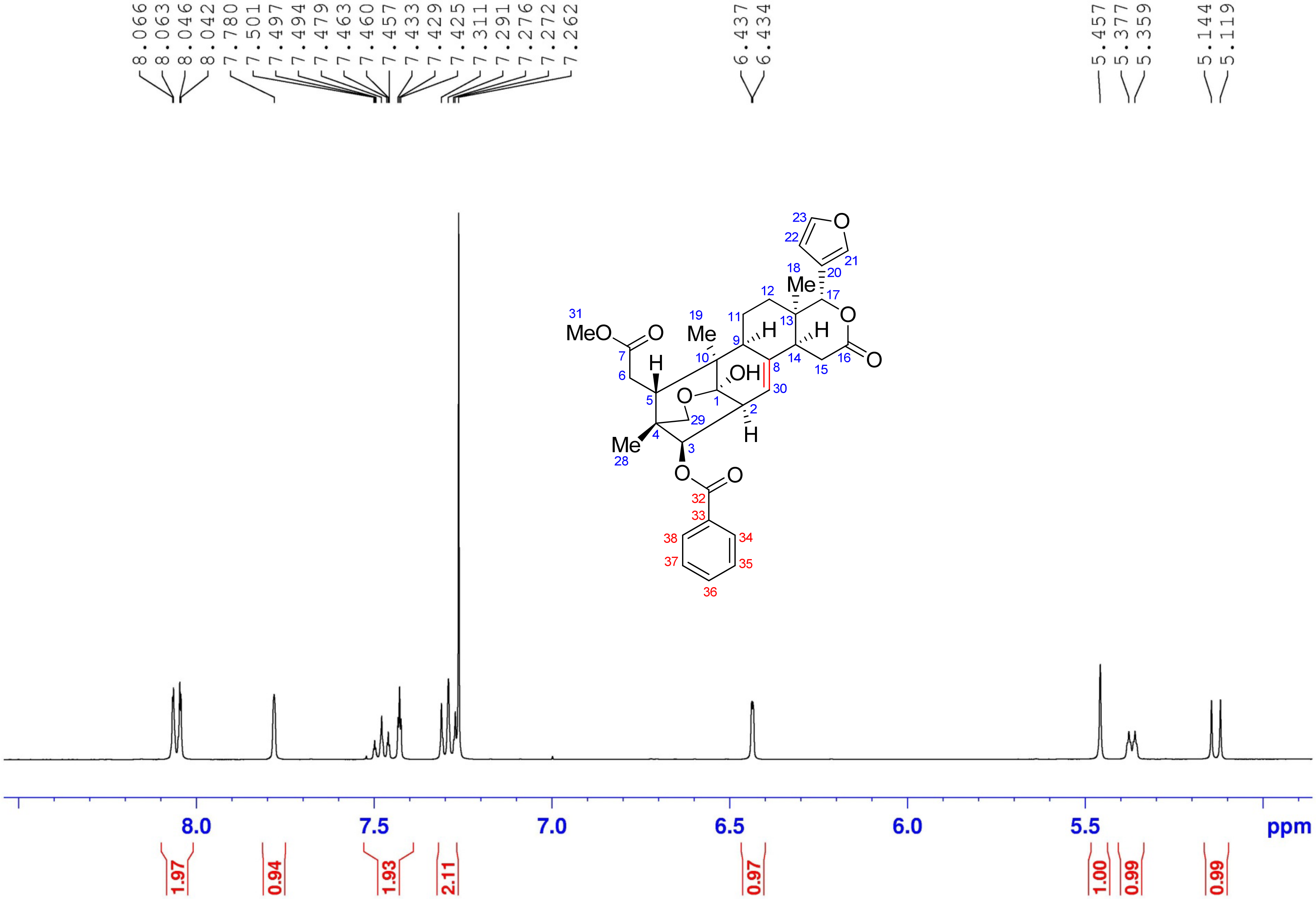

^{1}H NMR (400 MHz) spectrum of **8a** in $CDCl_3$

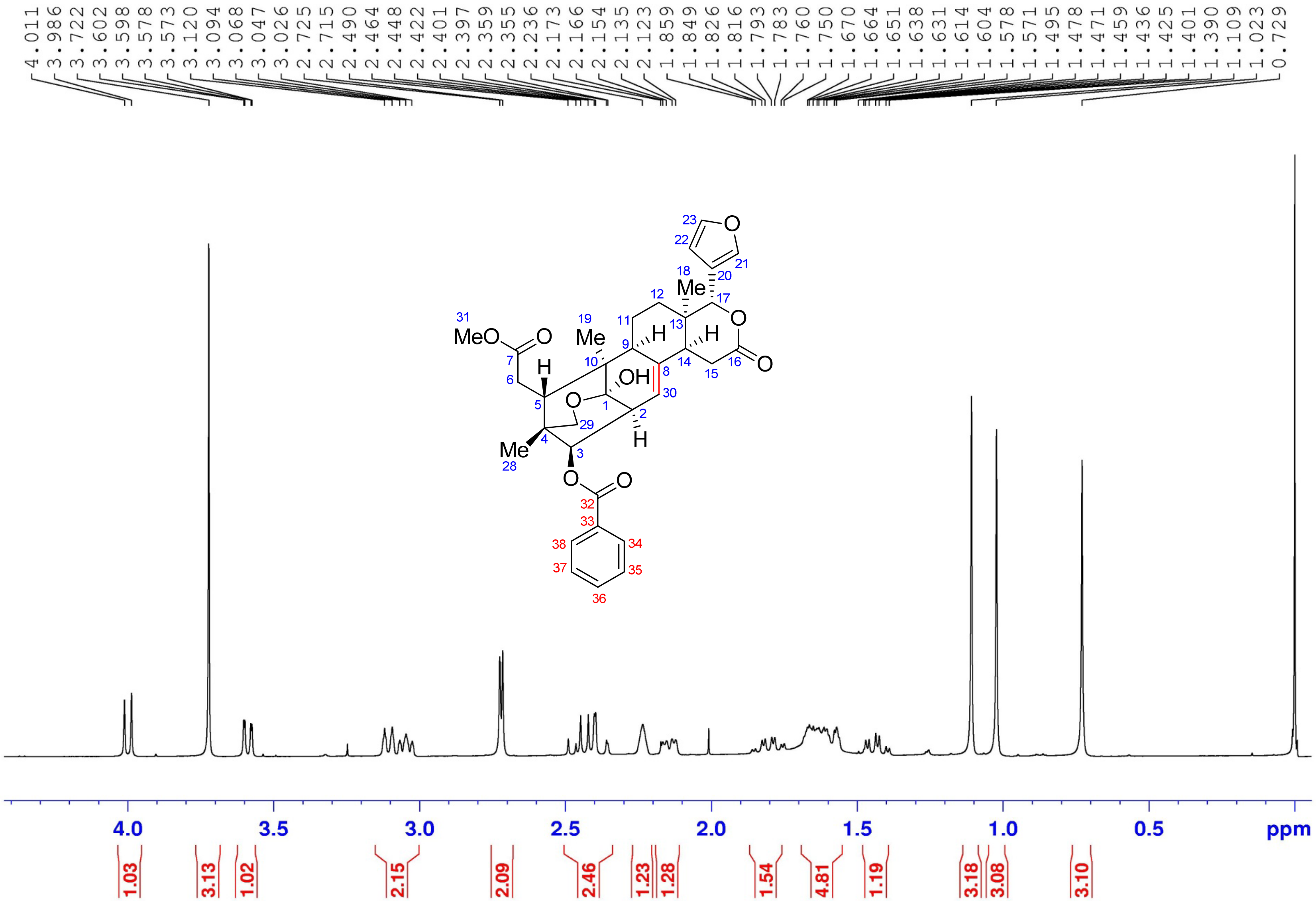

^{13}C NMR (100 MHz) spectrum of **8a** in $CDCl_3$

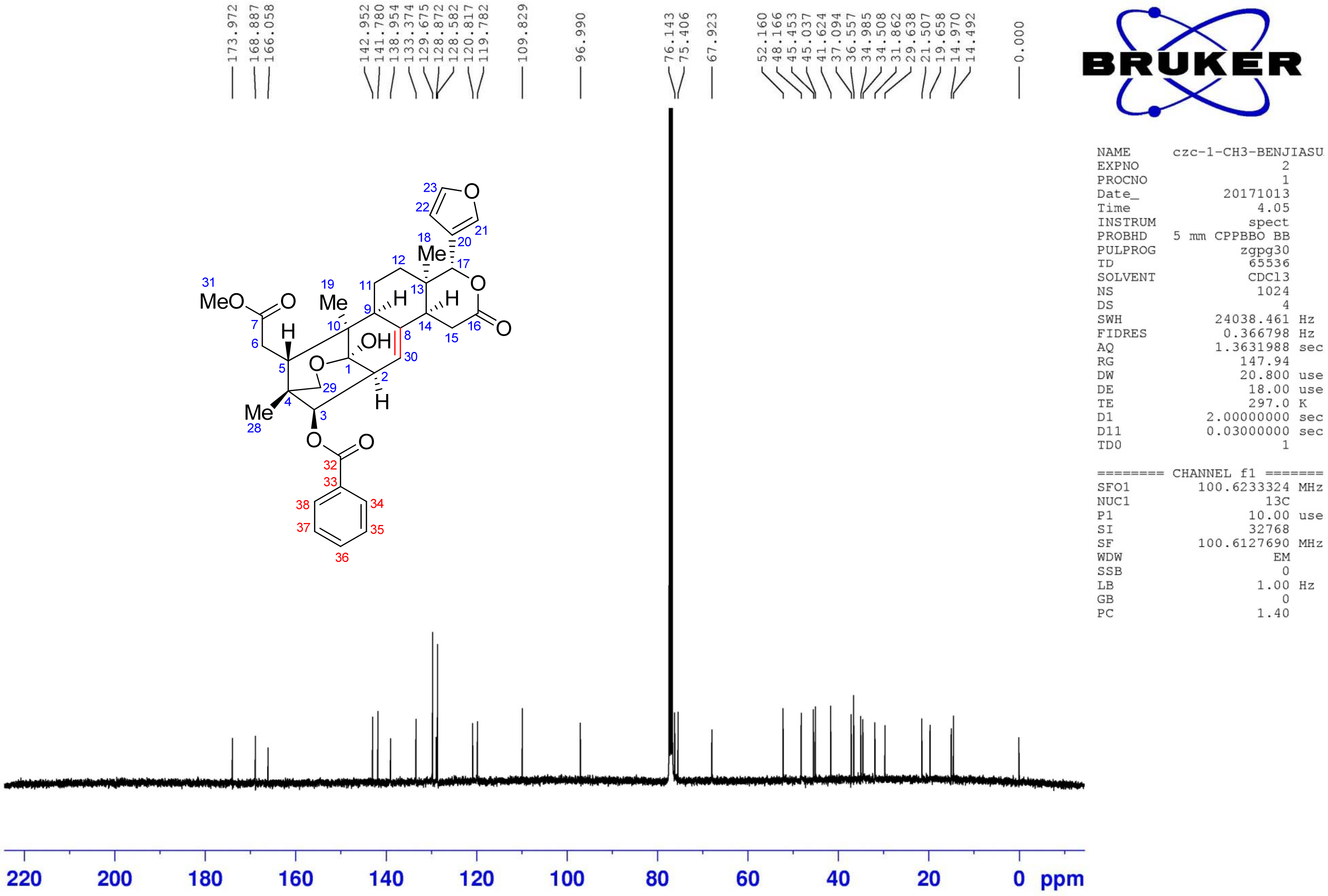


```
NAME       czc-1-CH3-BENJIASU
EXPNO                       2
PROCNO                      1
Date_                20171013
Time                     4.05
INSTRUM                 spect
PROBHD      5 mm CPPBBO BB
PULPROG               zgpg30
TD                      65536
SOLVENT                 CDCl3
NS                       1024
DS                          4
SWH                 24038.461 Hz
FIDRES               0.366798 Hz
AQ                  1.3631988 sec
RG                     147.94
DW                     20.800 use
DE                      18.00 use
TE                      297.0 K
D1                 2.00000000 sec
D11                0.03000000 sec
TD0                         1

======== CHANNEL f1 =======
SFO1            100.6233324 MHz
NUC1                      13C
P1                      10.00 use
SI                      32768
SF              100.6127690 MHz
WDW                        EM
SSB                         0
LB                       1.00 Hz
GB                          0
PC                       1.40
```

^{13}C NMR (100 MHz) spectrum of **8a** in $CDCl_3$

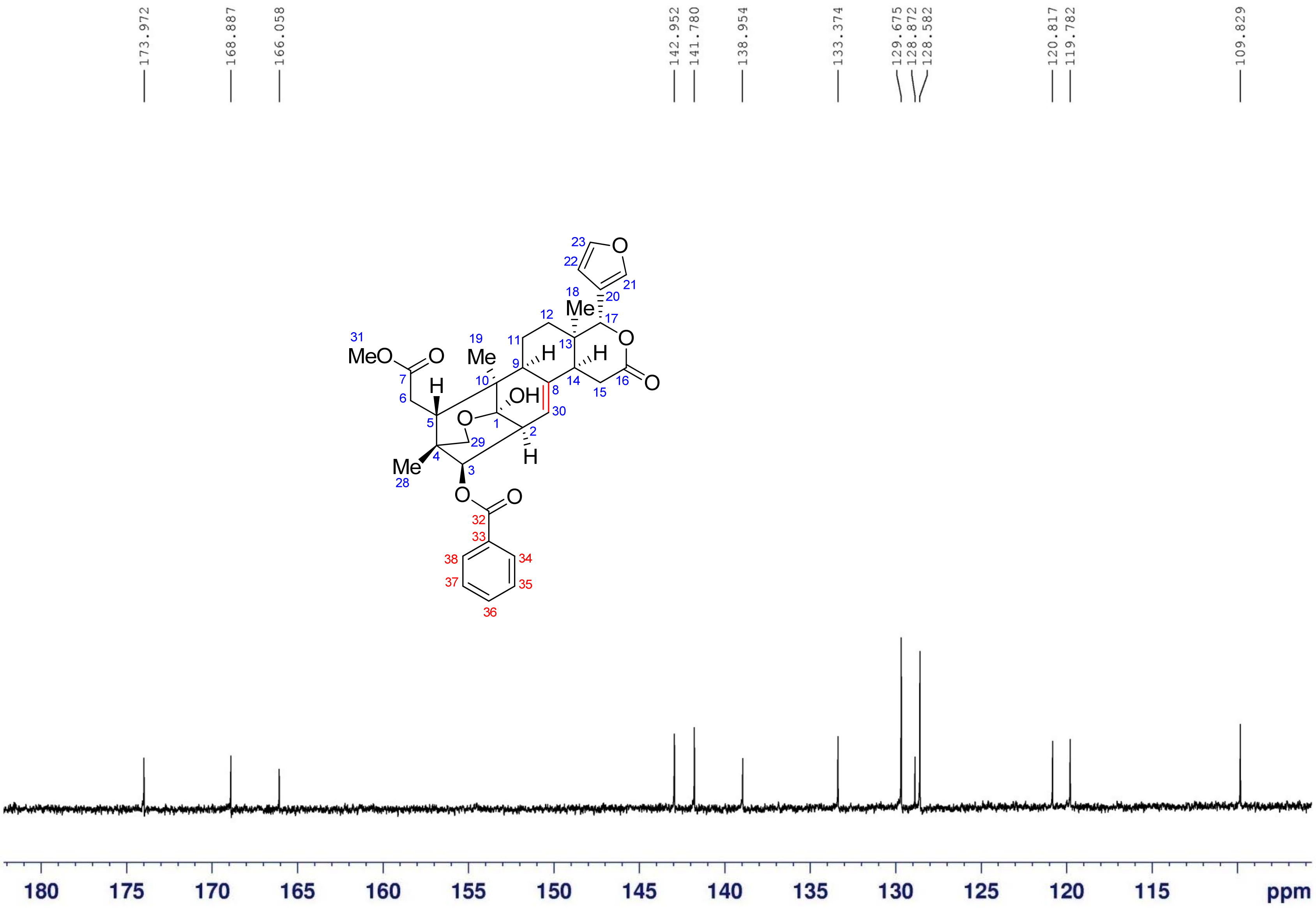

^{13}C NMR (100 MHz) spectrum of **8a** in $CDCl_3$

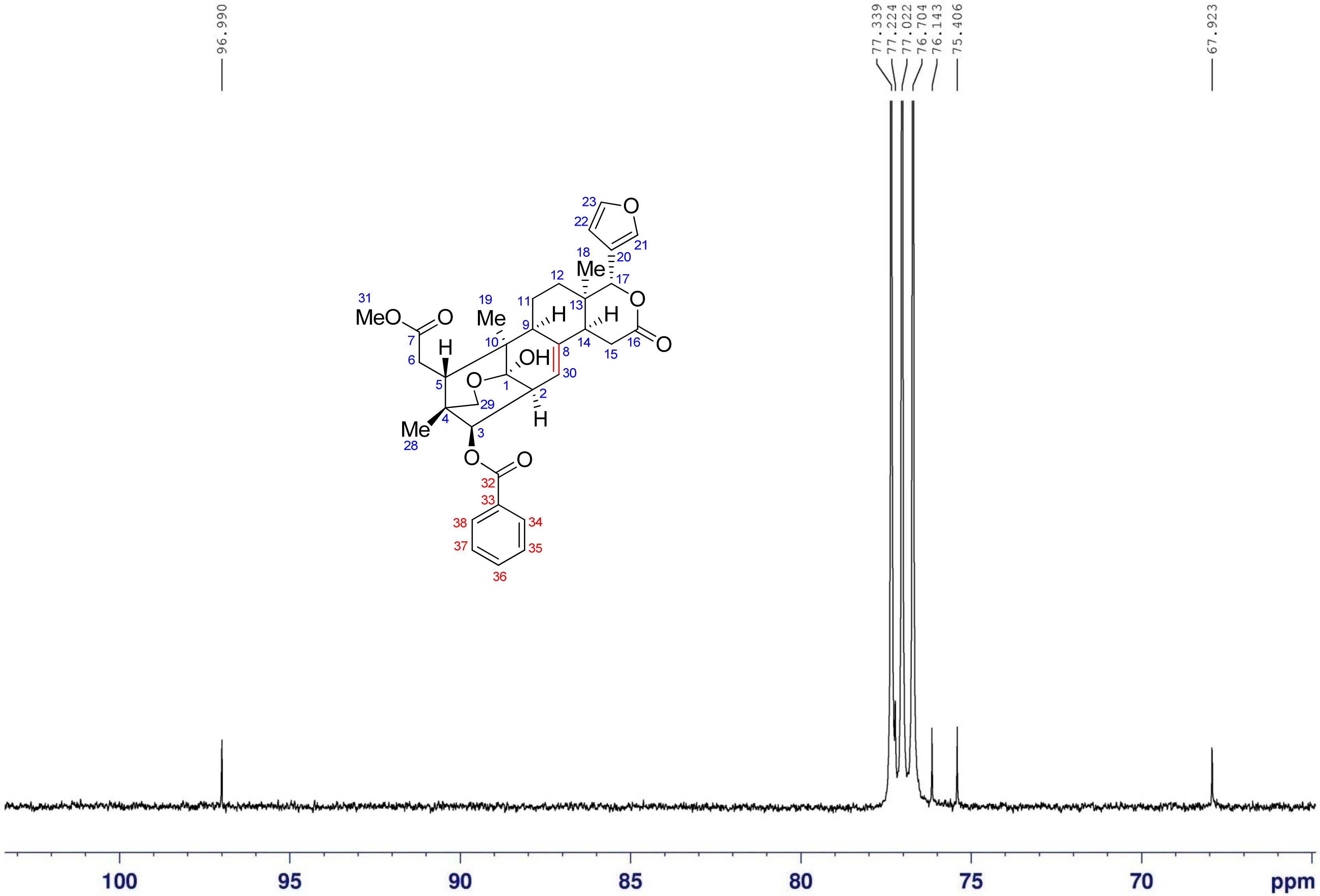

^{13}C NMR (100 MHz) spectrum of **8a** in $CDCl_3$

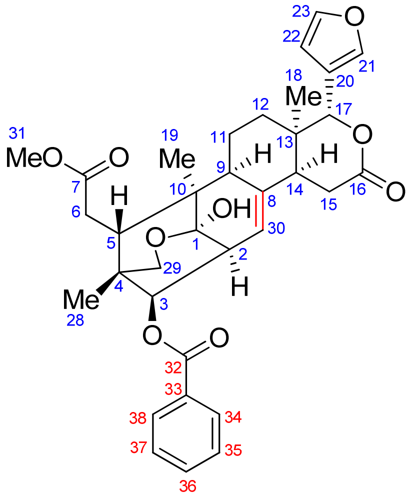

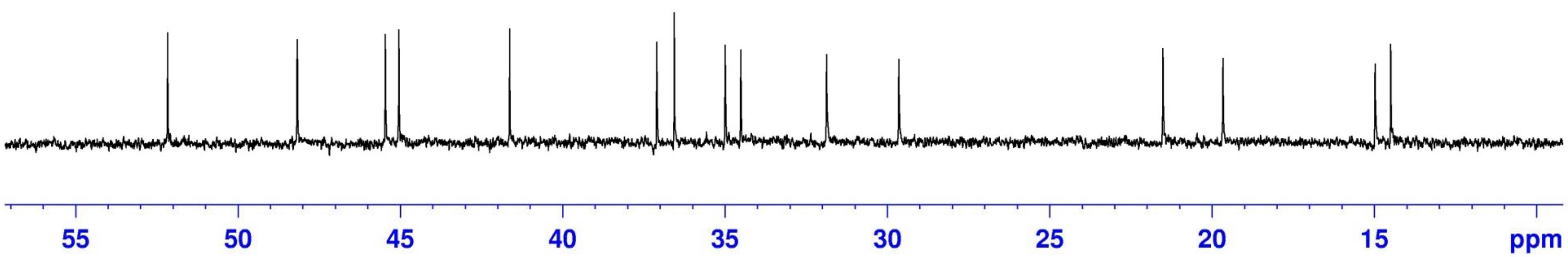

DEPT 135 spectrum of **8a** in $CDCl_3$

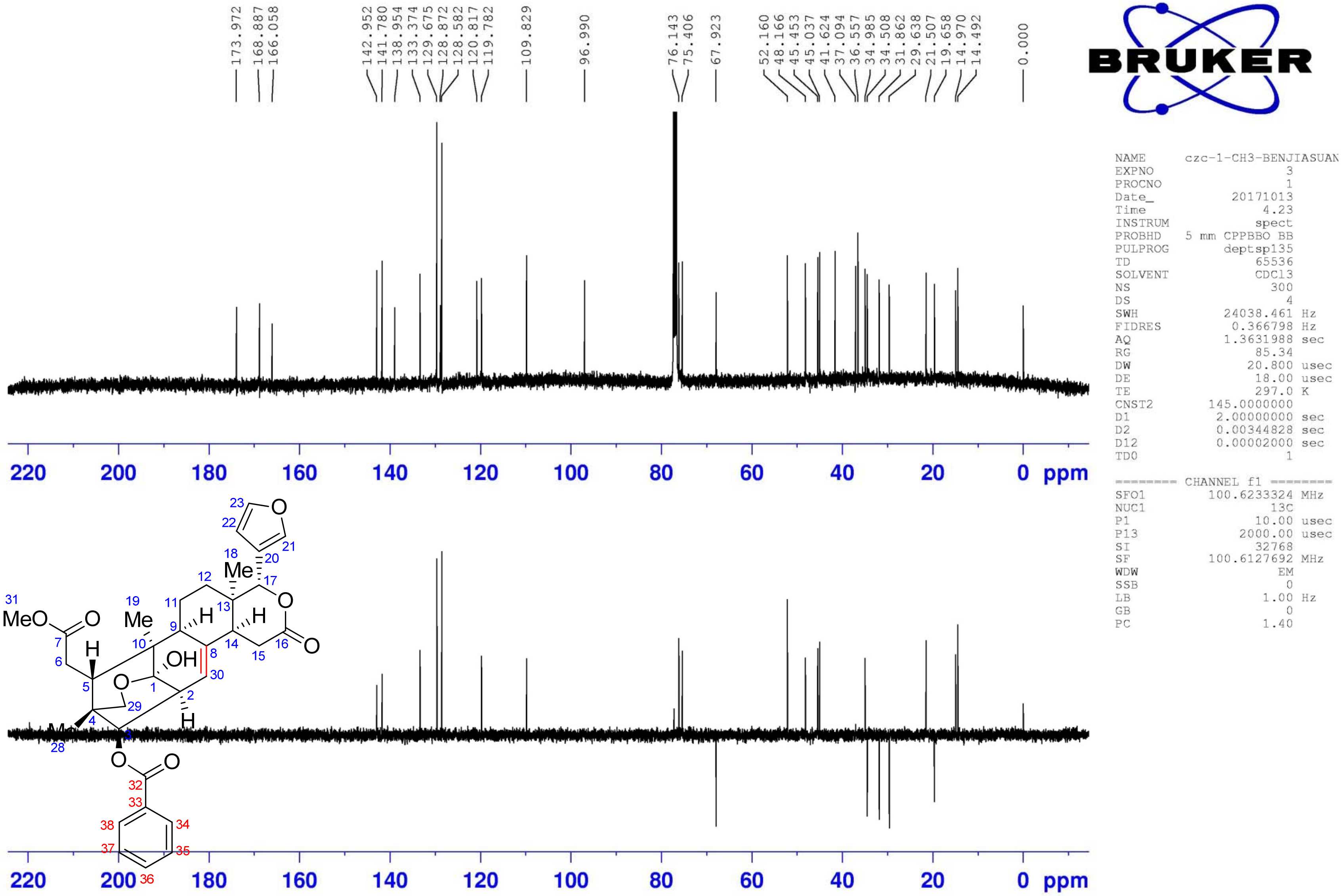

DEPT 135 spectrum of **8a** in $CDCl_3$

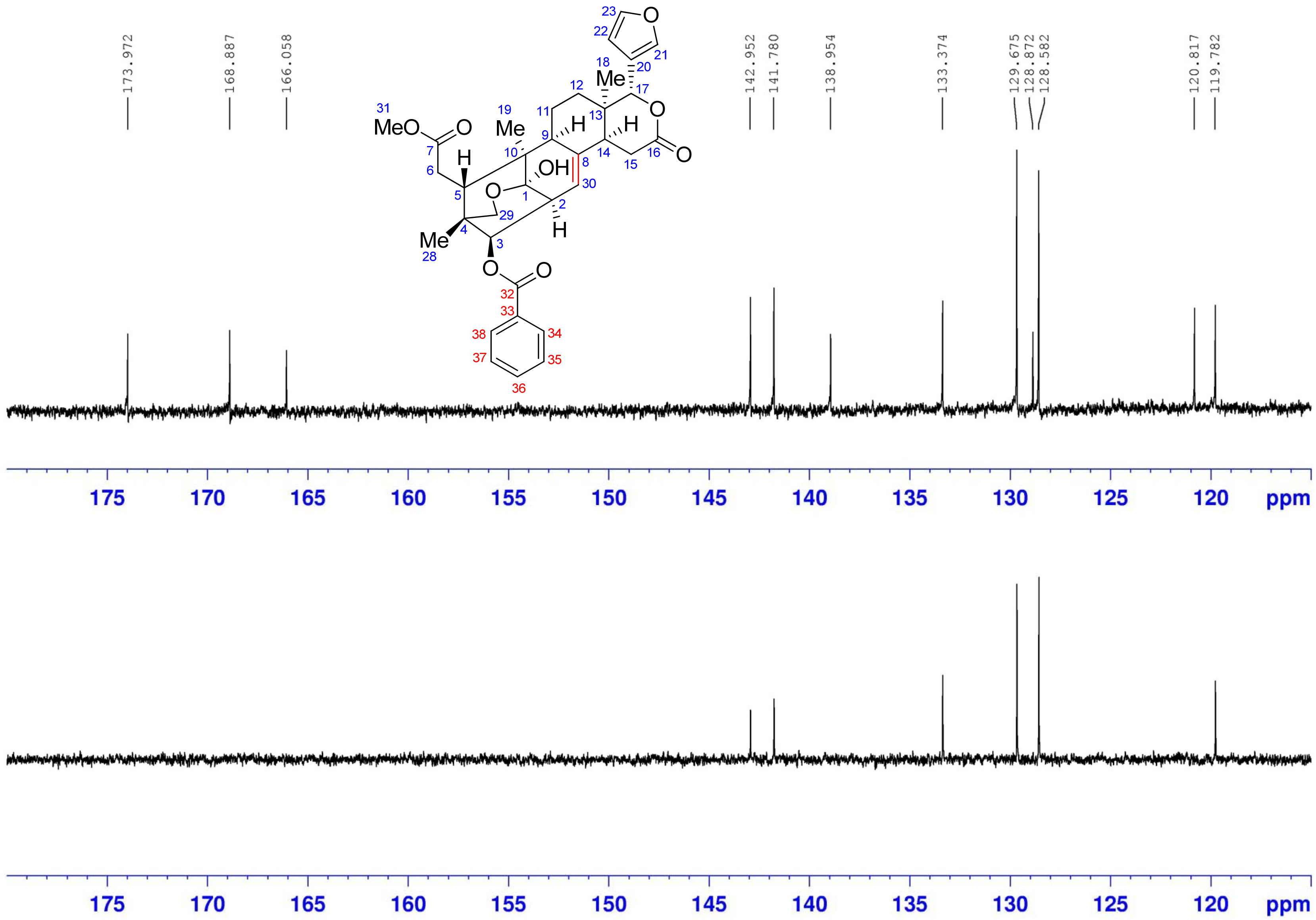

DEPT 135 spectrum of **8a** in $CDCl_3$

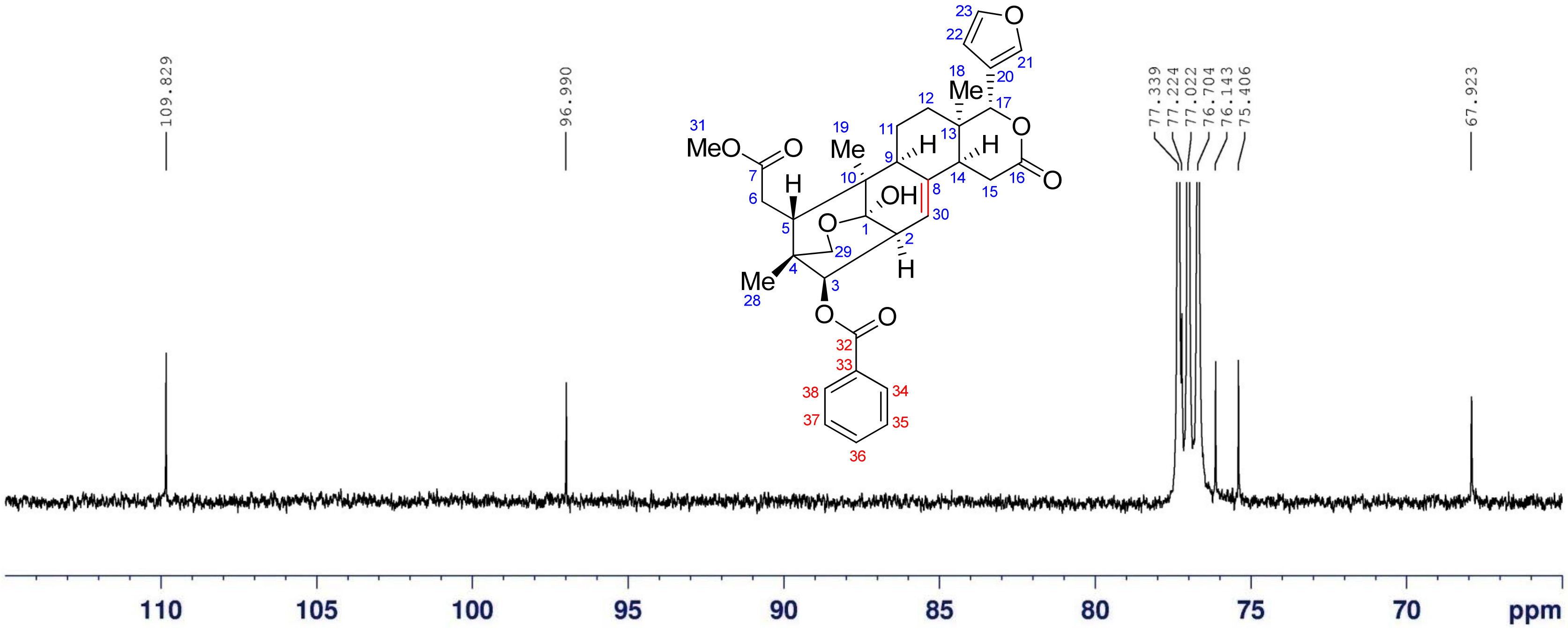

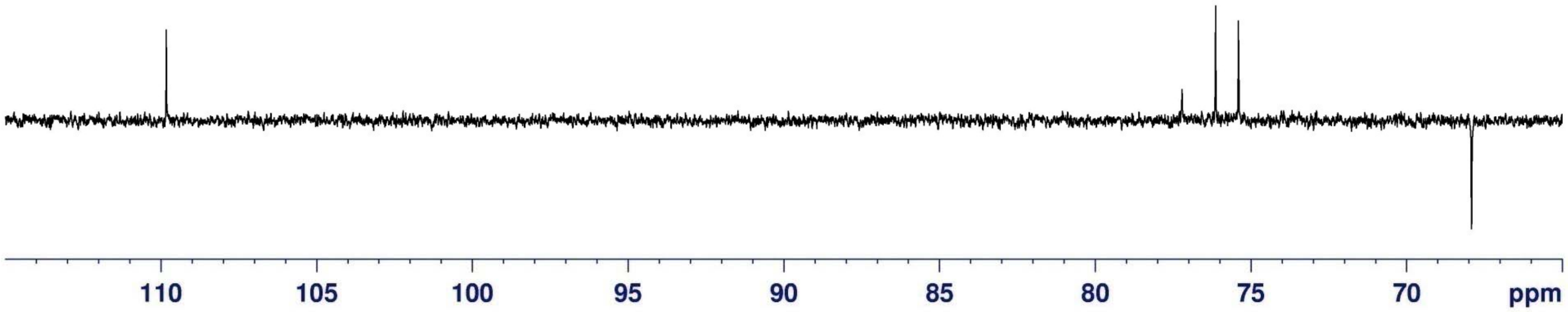

DEPT 135 spectrum of **8a** in $CDCl_3$

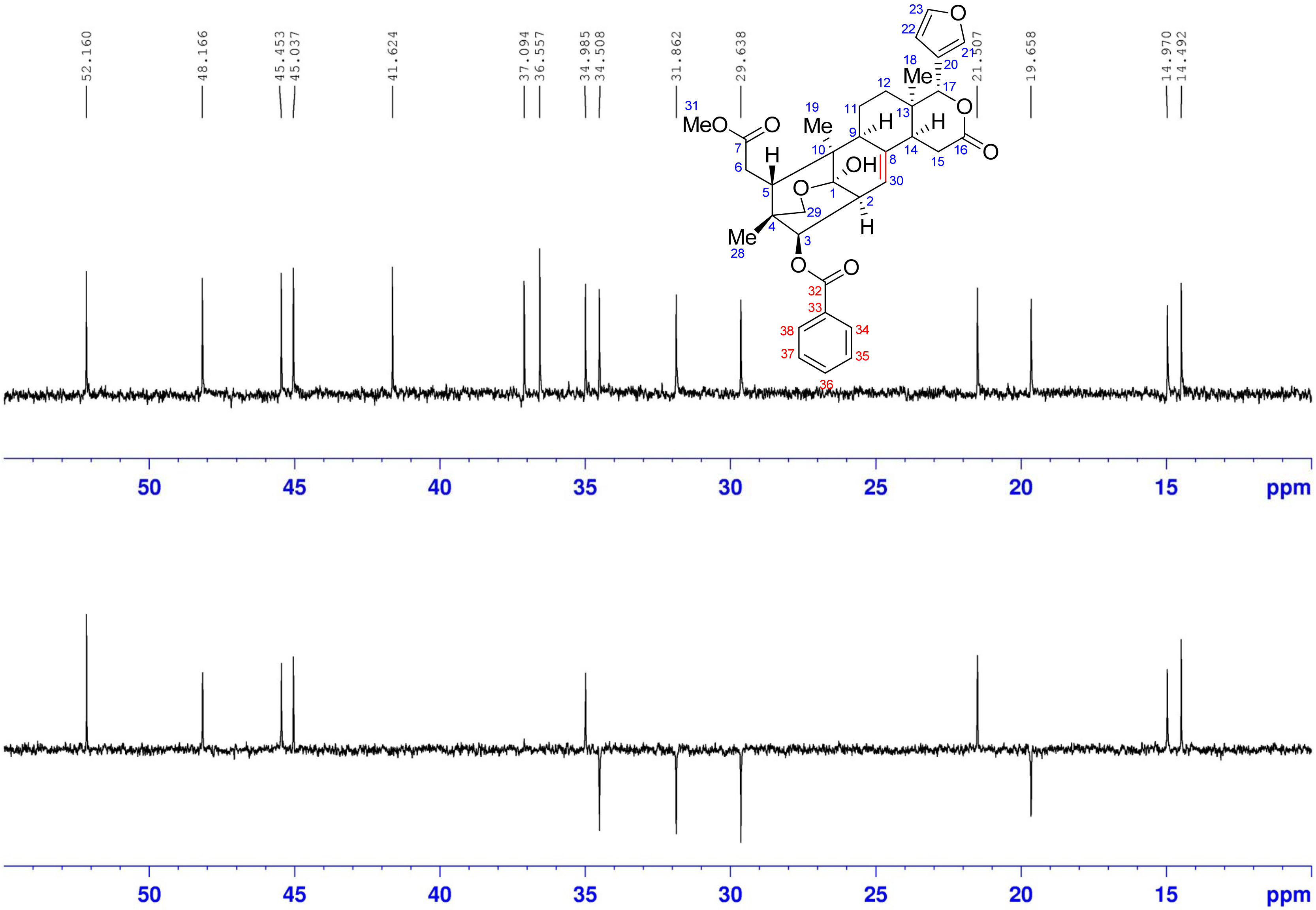

HR-ESIMS for compound **8b**

Generic Display Report

Analysis Info

Analysis Name	D:\Data\MS\data\201809\liwanshan_WSD-69_pos_15_01_5576.d	Acquisition Date	9/30/2018 6:08:56 PM
Method	LC_Direct Infusion_pos_100-1000mz.m	Operator	SCSIO
Sample Name	liwanshan_WSD-69_pos	Instrument	maXis
Comment			

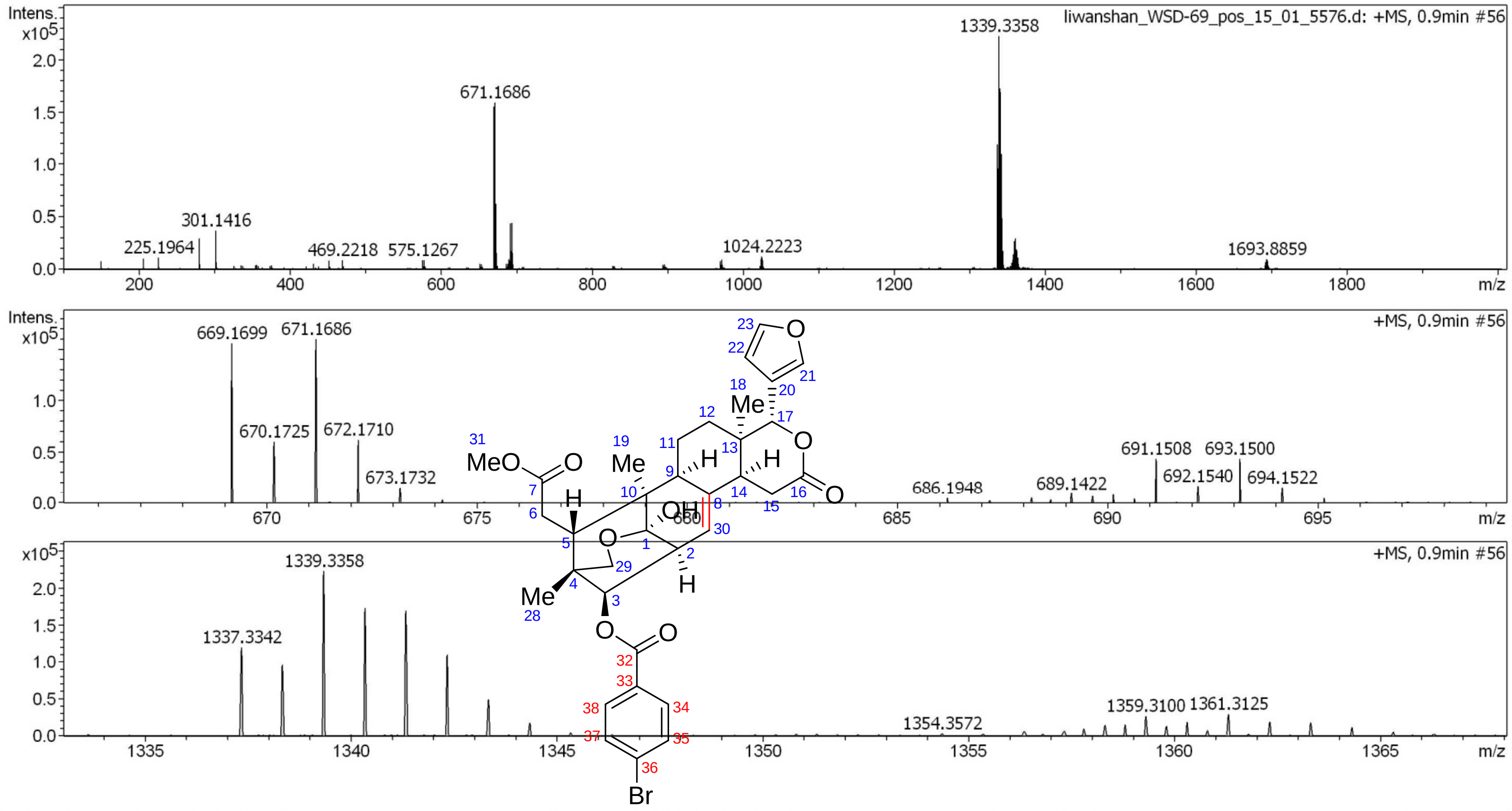

Bruker Compass DataAnalysis 4.1 printed: 9/30/2018 6:23:49 PM by: SCSIO Page 1 of 1

HR-ESIMS for compound **8b**

Mass Spectrum SmartFormula Report

Analysis Info

Analysis Name	D:\Data\MS\data\201809\liwanshan_WSD-69_pos_15_01_5576.d	Acquisition Date	9/30/2018 6:08:56 PM
Method	LC_Direct Infusion_pos_100-1000mz.m	Operator	SCSIO
Sample Name	liwanshan_WSD-69_pos	Instrument	maXis 255552.00029
Comment			

Acquisition Parameter

Source Type	ESI	Ion Polarity	Positive	Set Nebulizer	0.4 Bar
Focus	Active	Set Capillary	4500 V	Set Dry Heater	180 °C
Scan Begin	100 m/z	Set End Plate Offset	-500 V	Set Dry Gas	4.0 l/min
Scan End	2000 m/z	Set Charging Voltage	0 V	Set Divert Valve	Waste
		Set Corona	0 nA	Set APCI Heater	0 °C

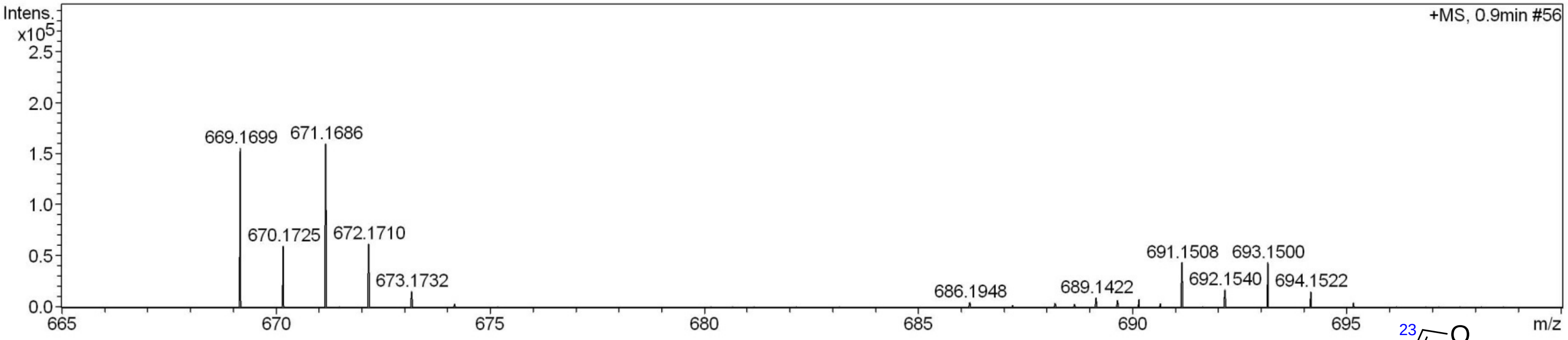

Meas. m/z	#	Ion Formula	Score	m/z	err [ppm]	err [mDa]	mSigma	rdb	e⁻ Conf	N-Rule
669.1699	1	C34H38BrO9	100.00	669.1694	0.8	0.5	21.4	15.5	even	ok
691.1508	1	C34H37BrNaO9	100.00	691.1513	0.8	0.5	28.4	15.5	even	ok
1337.3342	1	C68H75Br2O18	100.00	1337.3315	2.0	2.7	70.3	30.5	even	ok

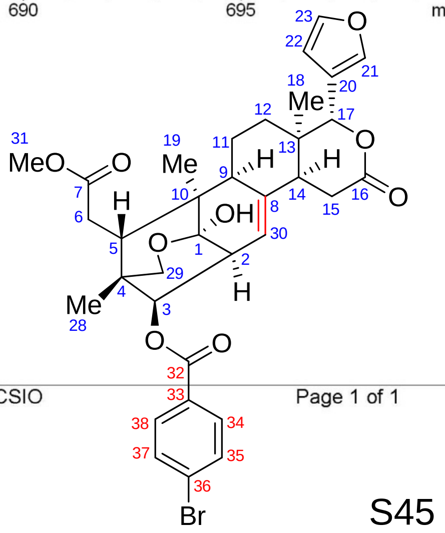

liwanshan_WSD-69_pos_15_01_5576.d

Bruker Compass DataAnalysis 4.1 printed: 9/30/2018 6:23:15 PM by: SCSIO Page 1 of 1

^1H NMR (400 MHz) spectrum of **8b** in $CDCl_3$

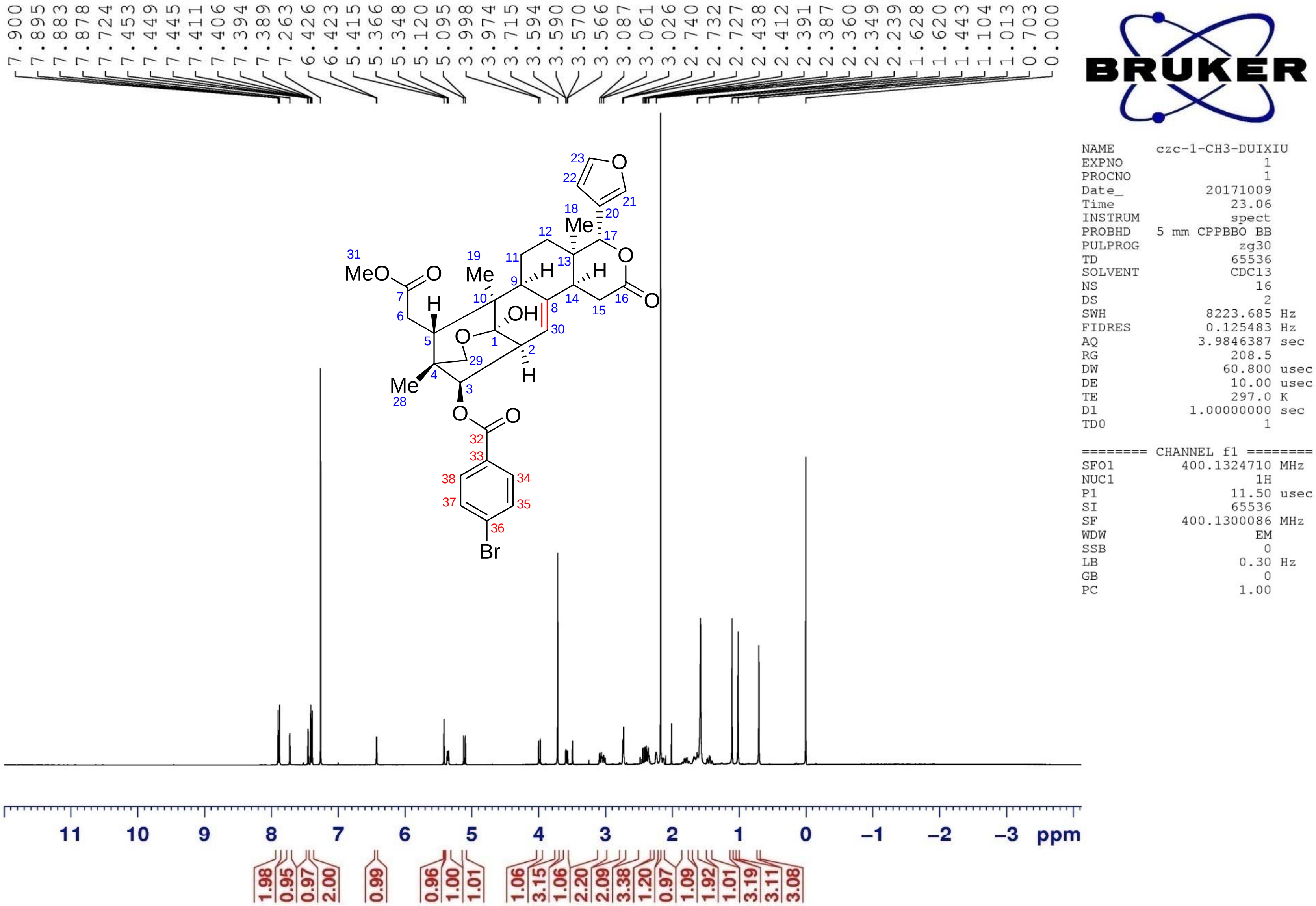

NAME	czc-1-CH3-DUIXIU
EXPNO	1
PROCNO	1
Date_	20171009
Time	23.06
INSTRUM	spect
PROBHD	5 mm CPPBBO BB
PULPROG	zg30
TD	65536
SOLVENT	CDCl3
NS	16
DS	2
SWH	8223.685 Hz
FIDRES	0.125483 Hz
AQ	3.9846387 sec
RG	208.5
DW	60.800 usec
DE	10.00 usec
TE	297.0 K
D1	1.00000000 sec
TD0	1

======== CHANNEL f1 ========

SFO1	400.1324710 MHz
NUC1	1H
P1	11.50 usec
SI	65536
SF	400.1300086 MHz
WDW	EM
SSB	0
LB	0.30 Hz
GB	0
PC	1.00

^{1}H NMR (400 MHz) spectrum of **8b** in $CDCl_3$

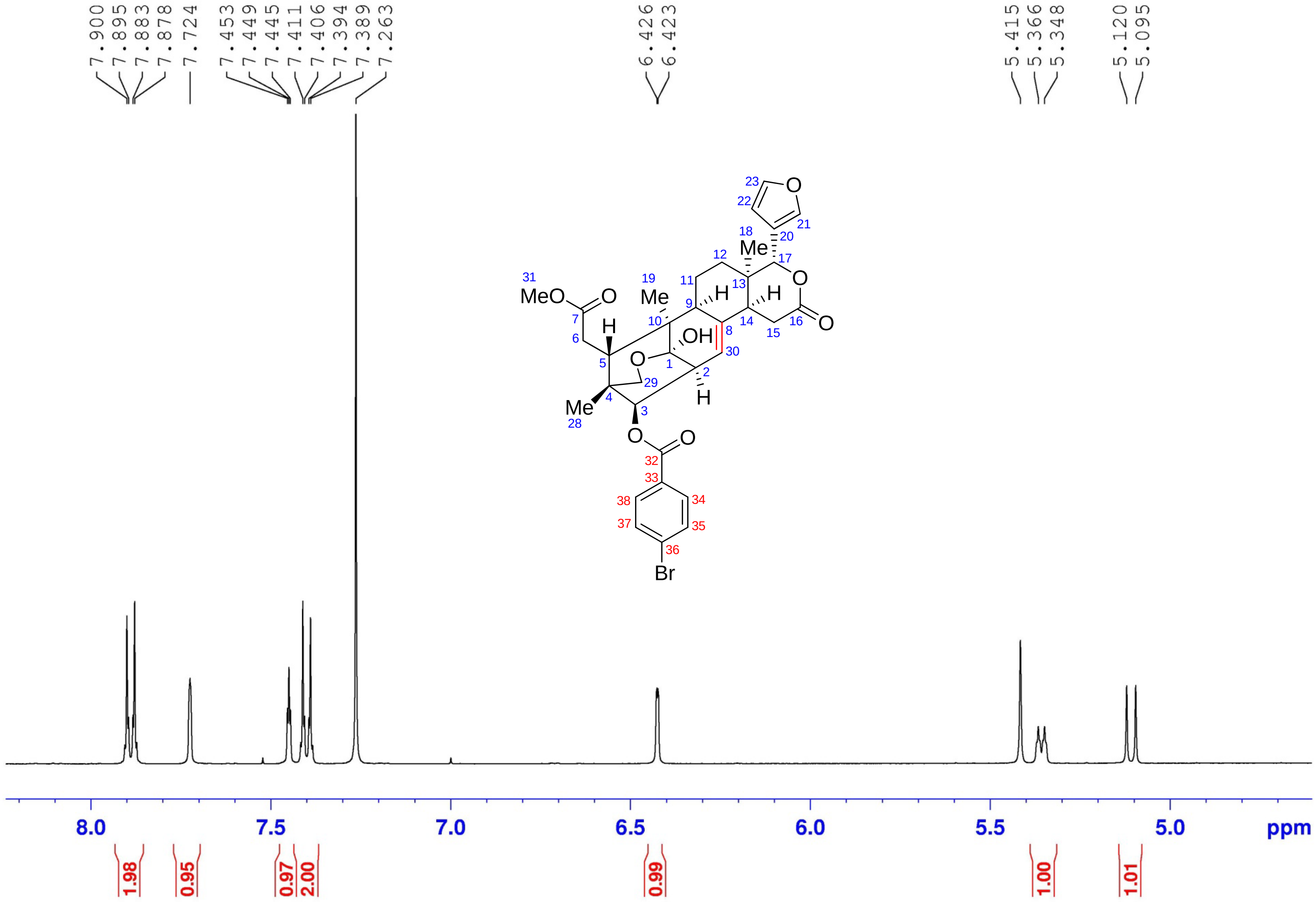

^1H NMR (400 MHz) spectrum of **8b** in $CDCl_3$

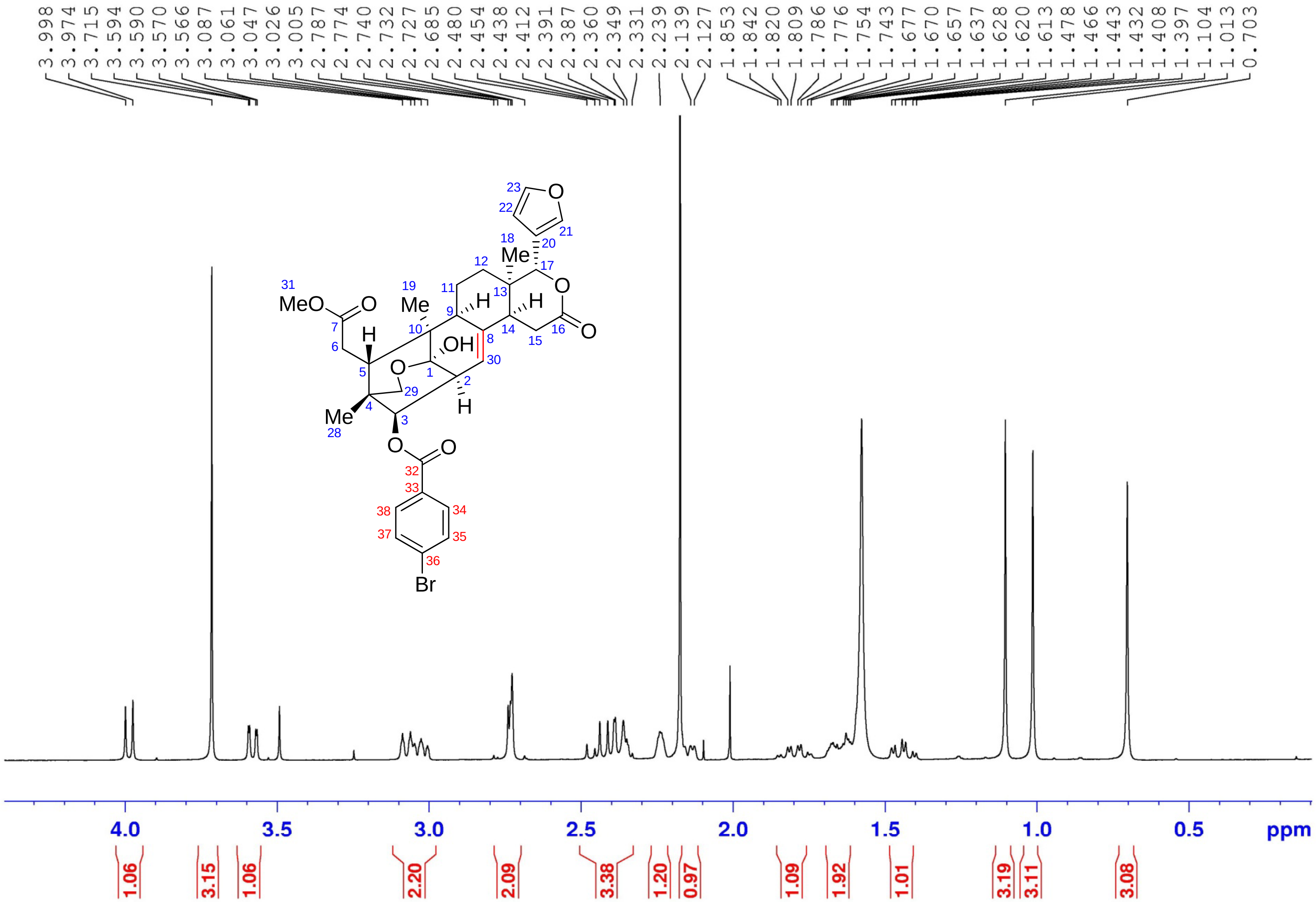

^{13}C NMR (100 MHz) spectrum of **8b** in $CDCl_3$

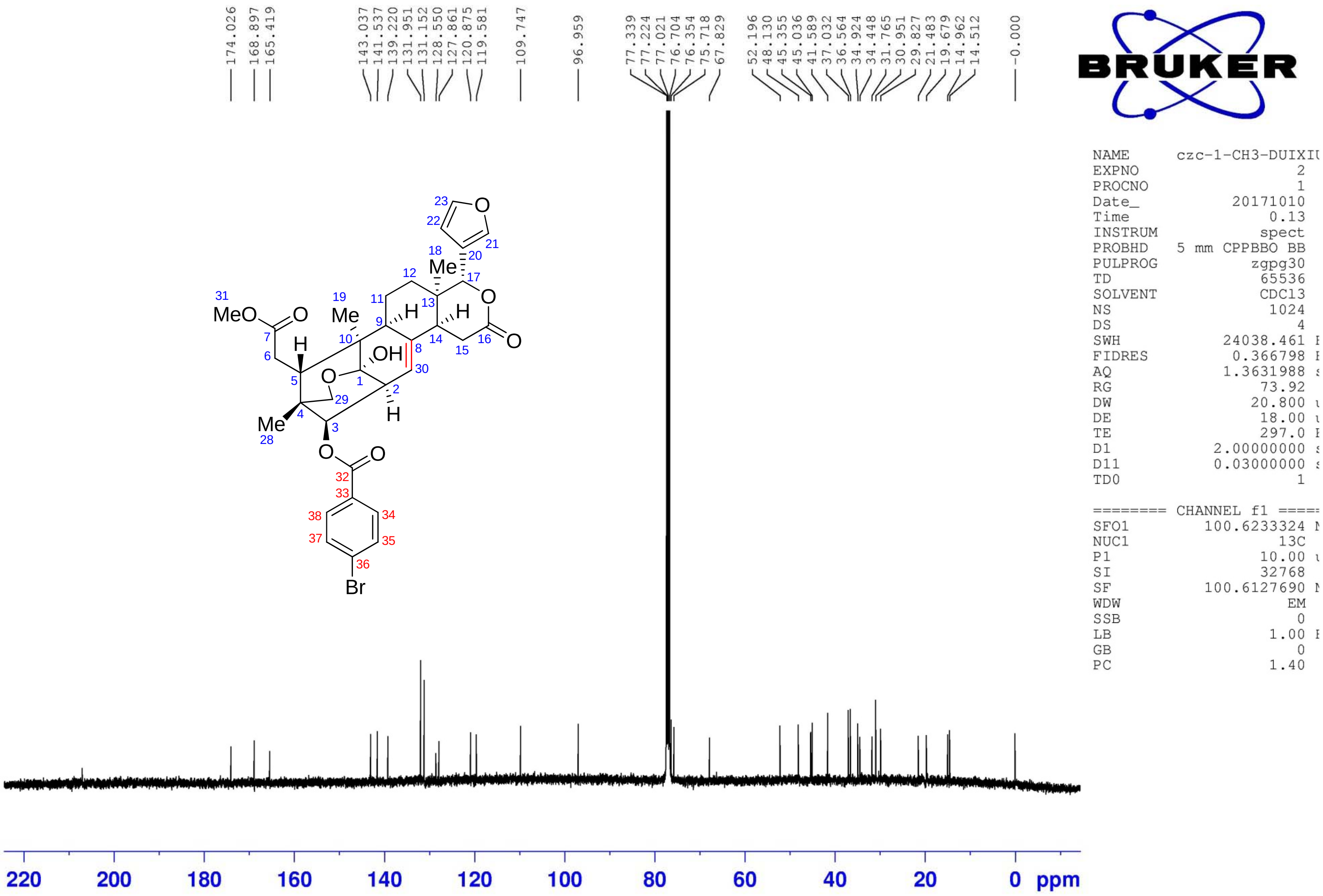

^{13}C NMR (100 MHz) spectrum of **8b** in $CDCl_3$

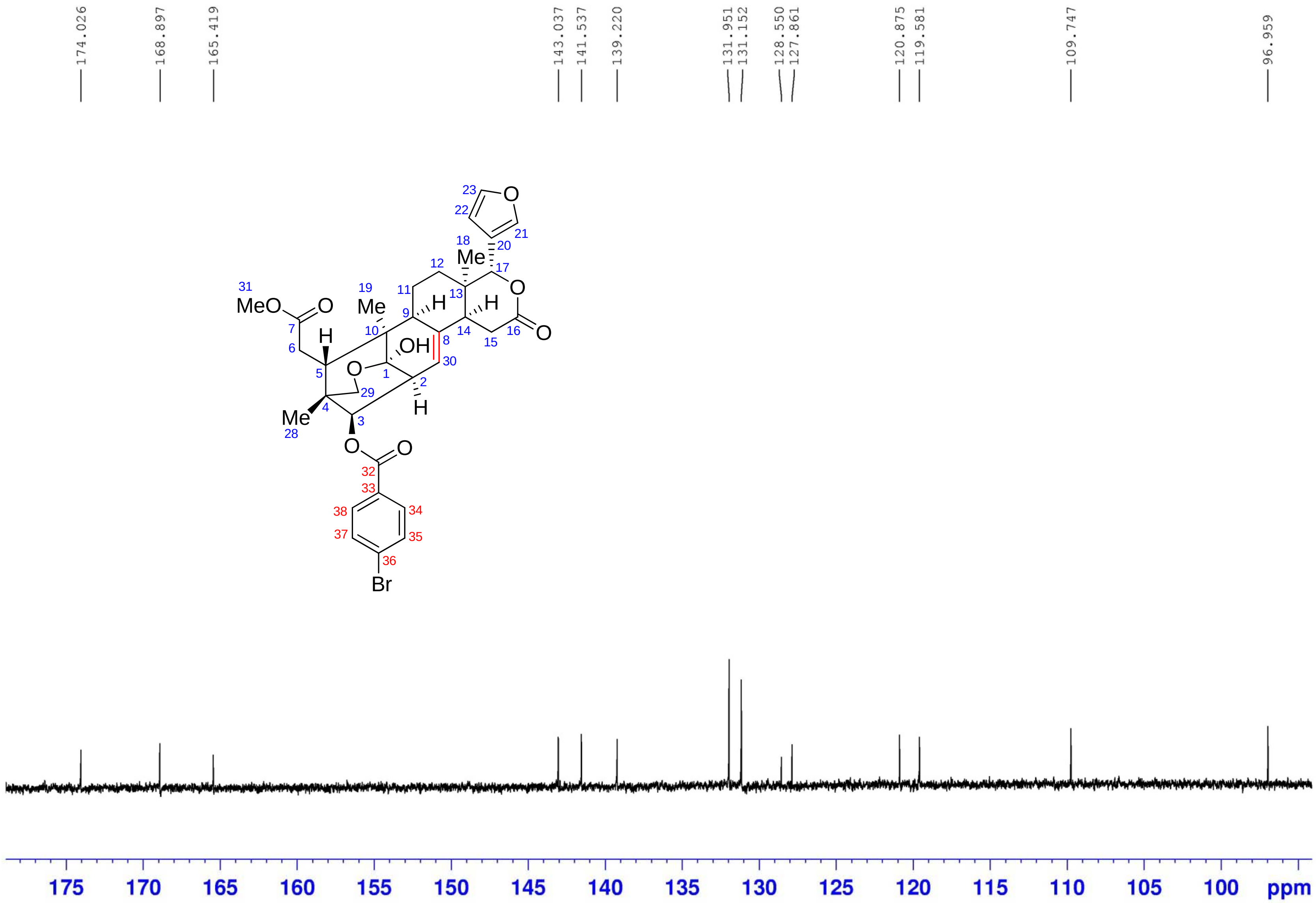

^{13}C NMR (100 MHz) spectrum of **8b** in $CDCl_3$

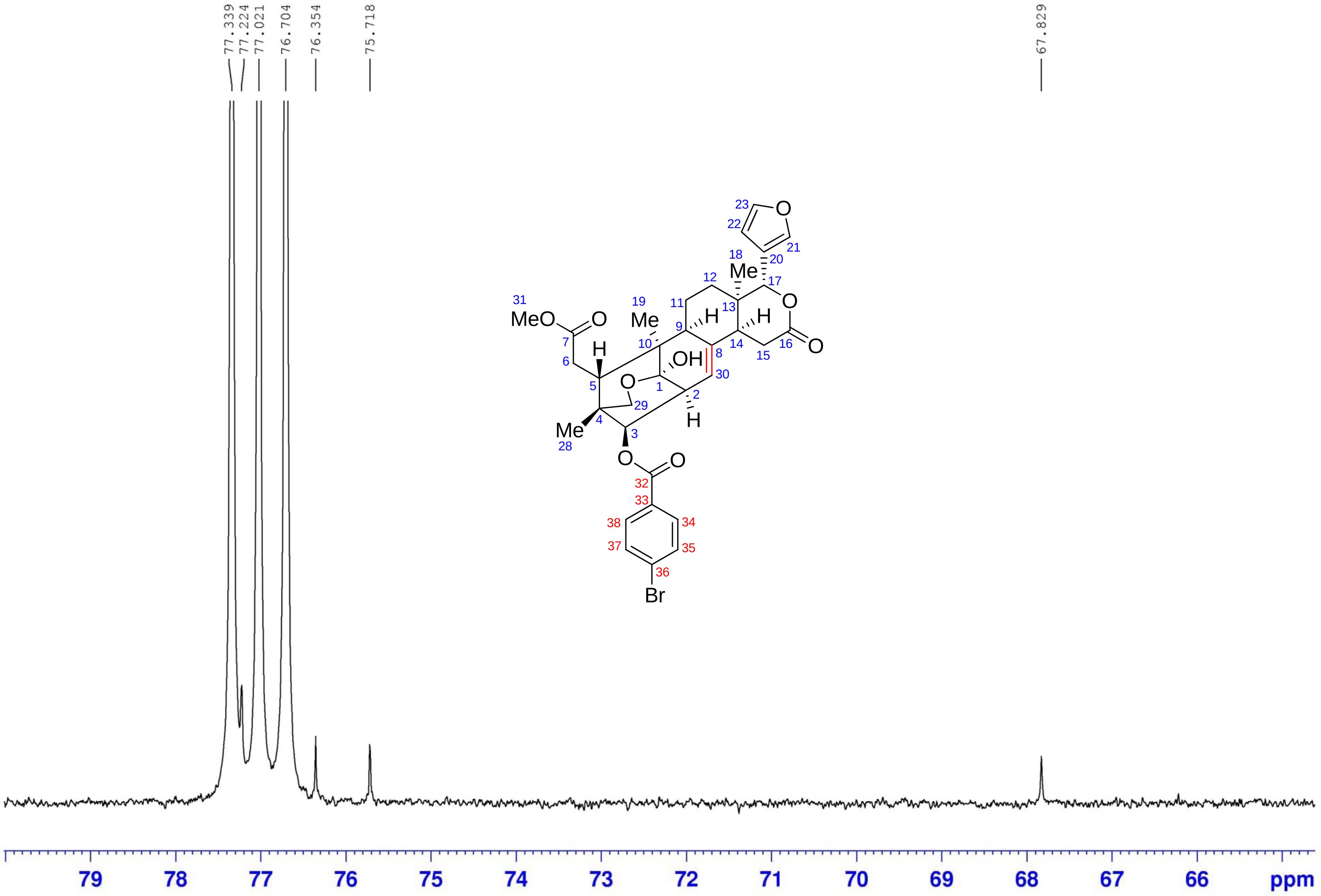

^{13}C NMR (100 MHz) spectrum of **8b** in $CDCl_3$

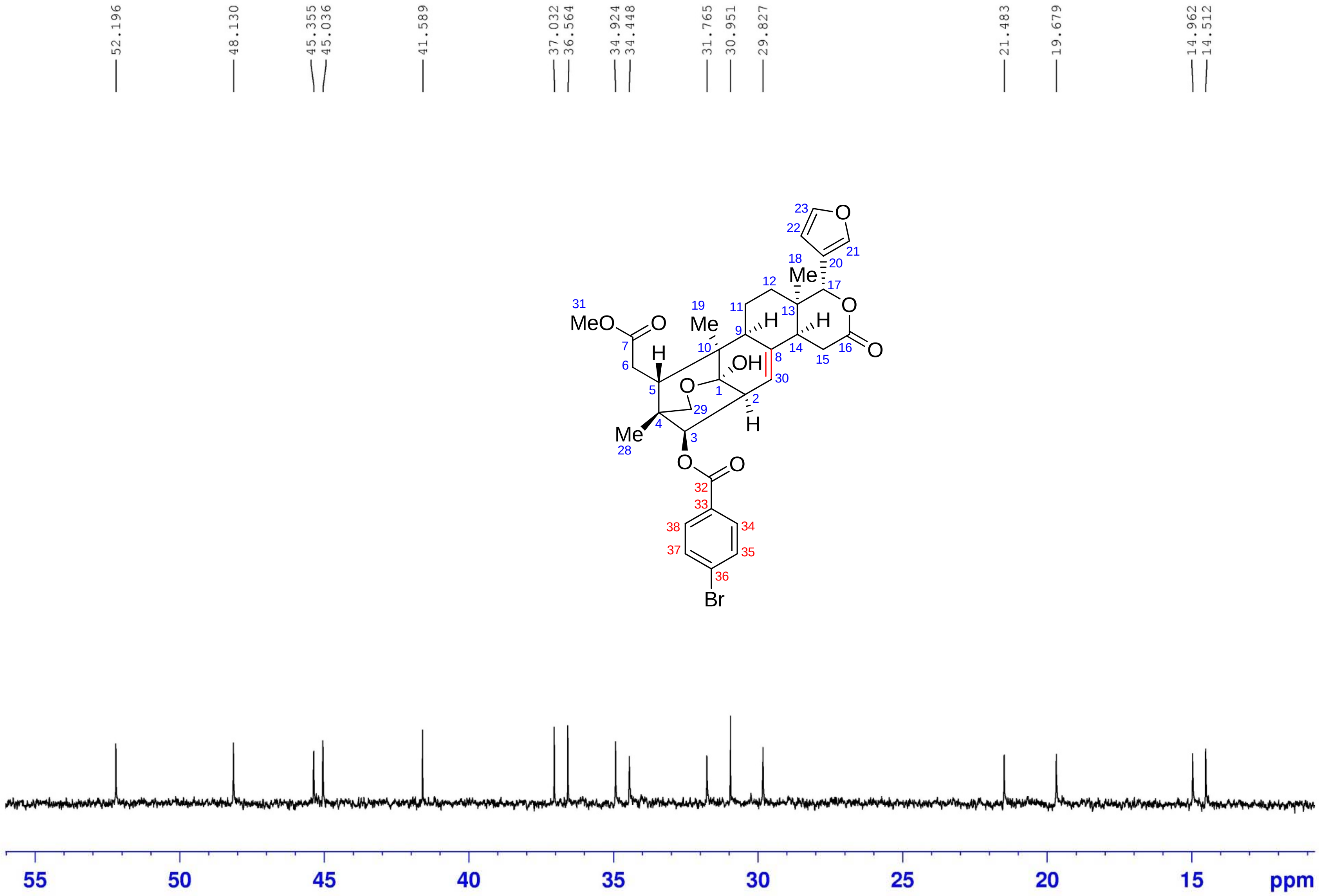

DEPT 135 spectrum of **8b** in $CDCl_3$

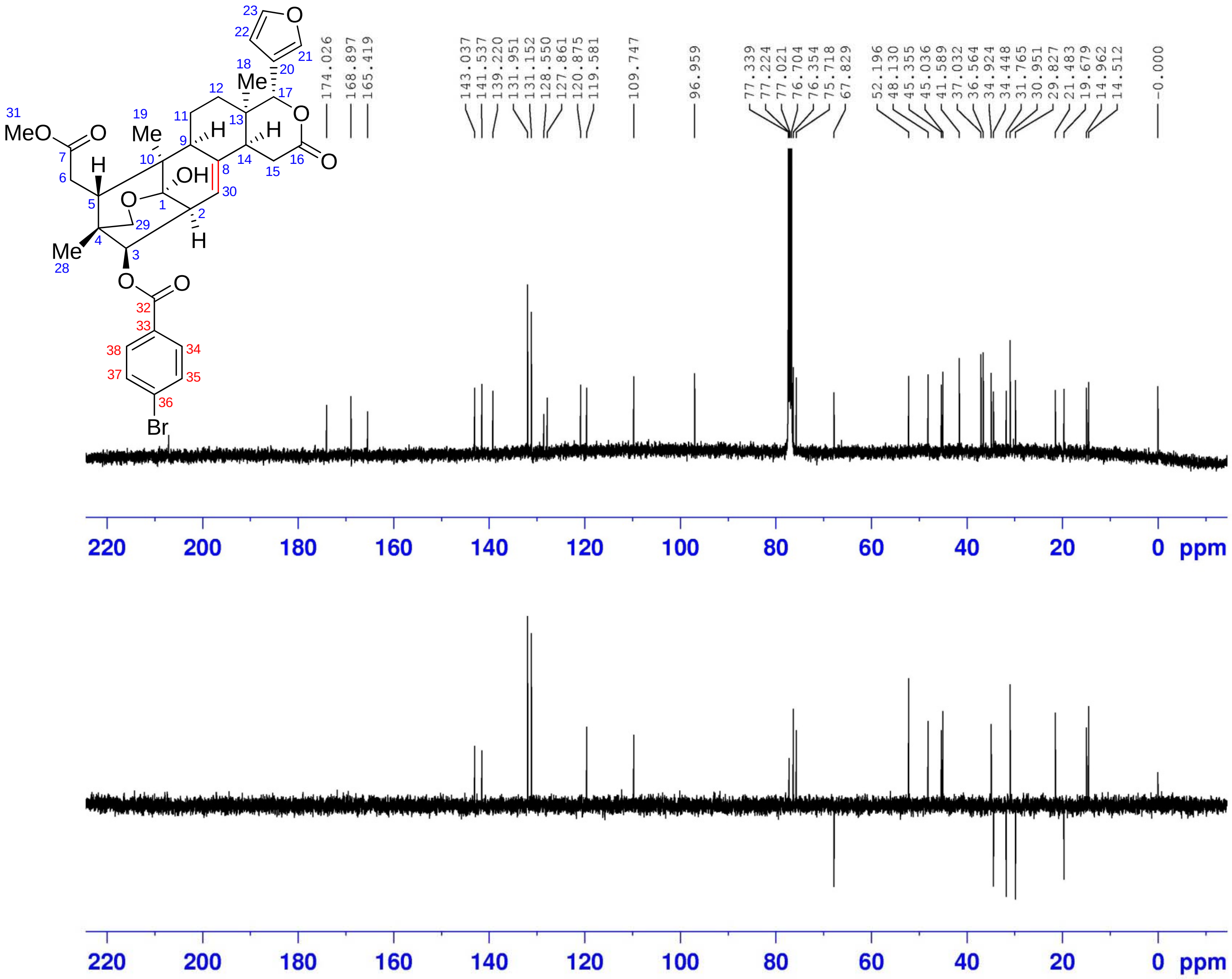

NAME	czc-1-CH3-DUIXI
EXPNO	3
PROCNO	1
Date_	20171010
Time	0.31
INSTRUM	spect
PROBHD	5 mm CPPBBO BB
PULPROG	deptsp135
TD	65536
SOLVENT	CDCl3
NS	300
DS	4
SWH	24038.461
FIDRES	0.366798
AQ	1.3631988
RG	130.26
DW	20.800
DE	18.00
TE	297.0
CNST2	145.0000000
D1	2.00000000
D2	0.00344828
D12	0.00002000
TD0	1

======== CHANNEL f1 ====

SFO1	100.6233324
NUC1	13C
P1	10.00
P13	2000.00
SI	32768
SF	100.6127693
WDW	EM
SSB	0
LB	1.00
GB	0
PC	1.40

DEPT 135 spectrum of **8b** in $CDCl_3$

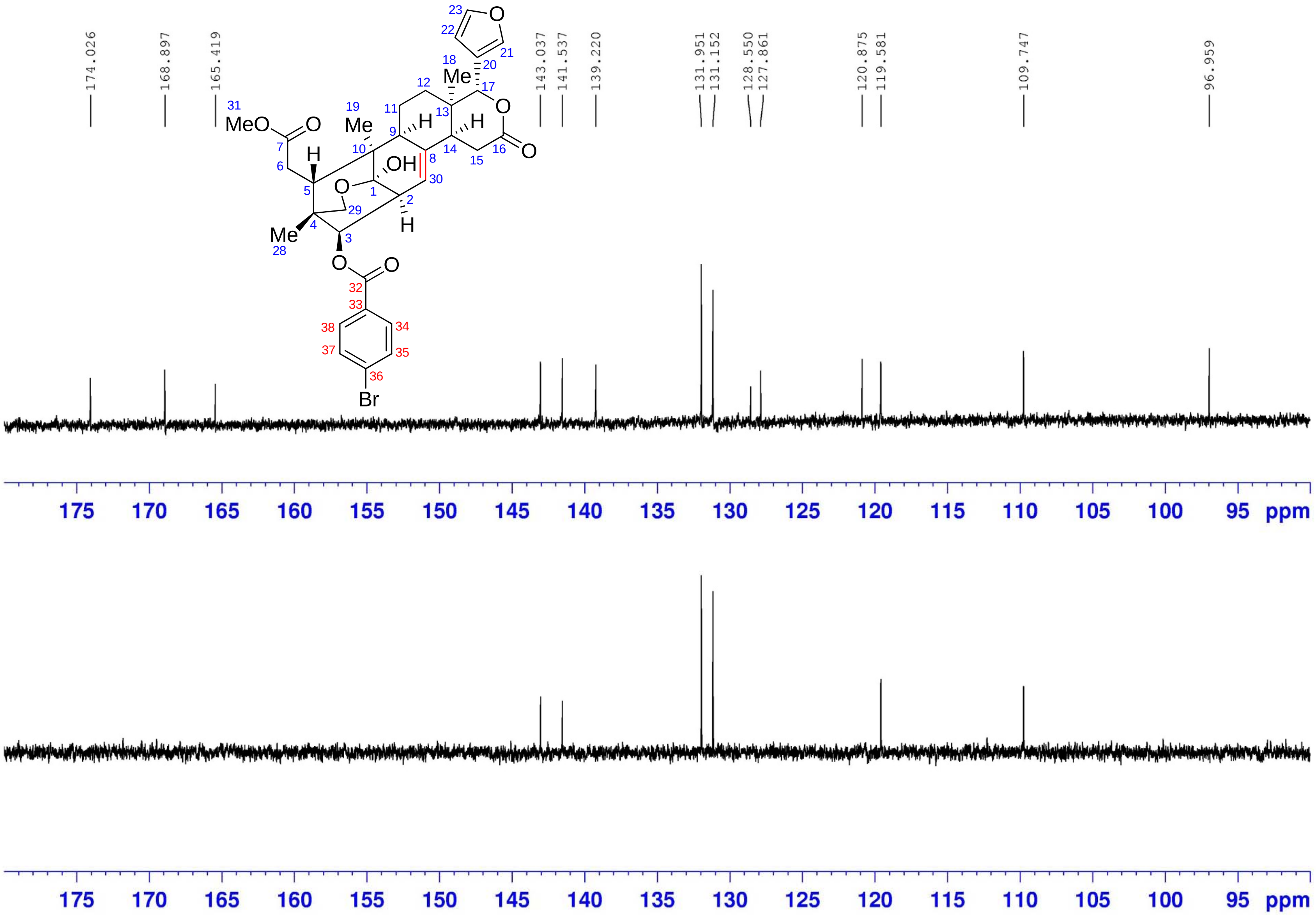

DEPT 135 spectrum of **8b** in $CDCl_3$

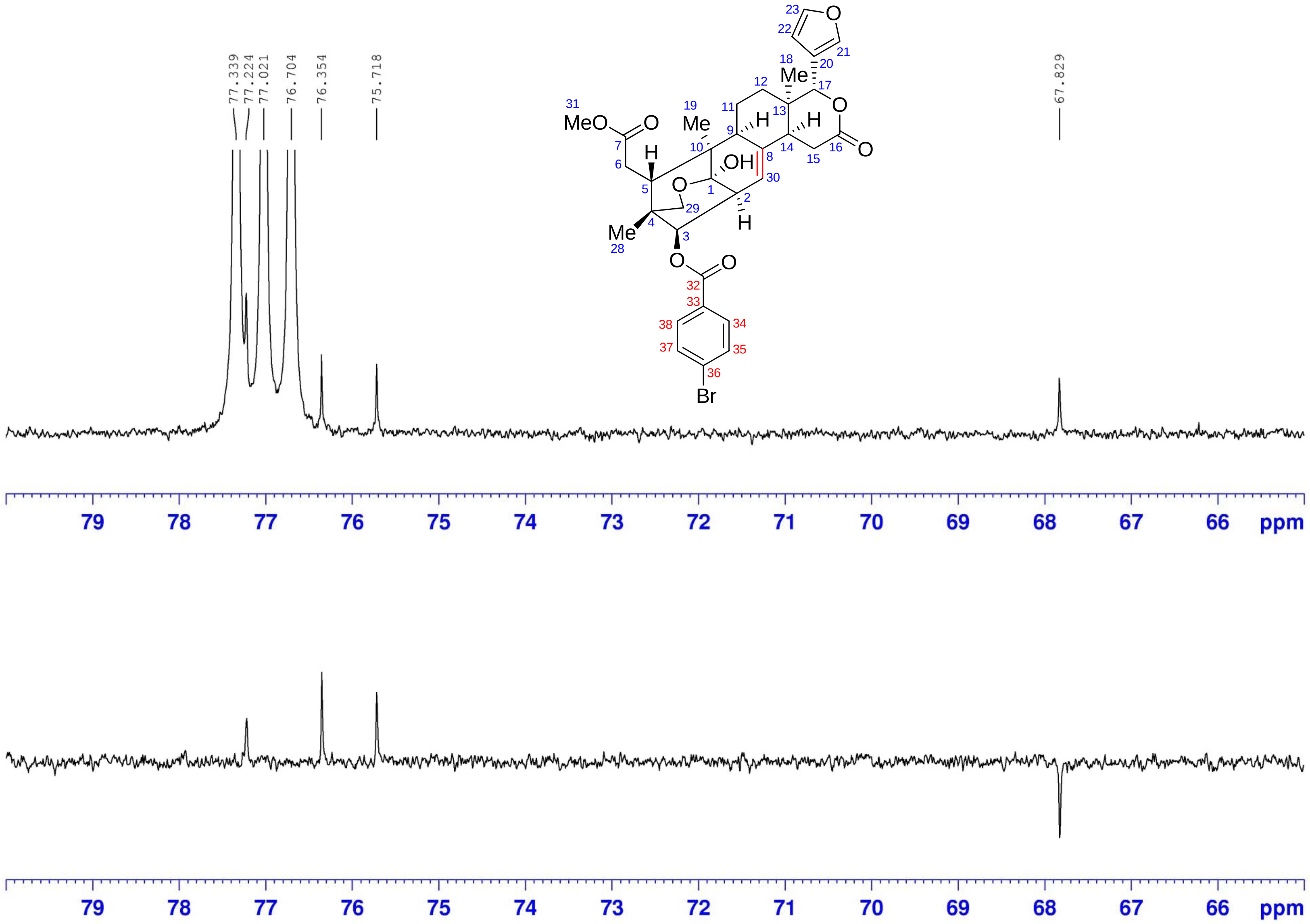

DEPT 135 spectrum of **8b** in $CDCl_3$

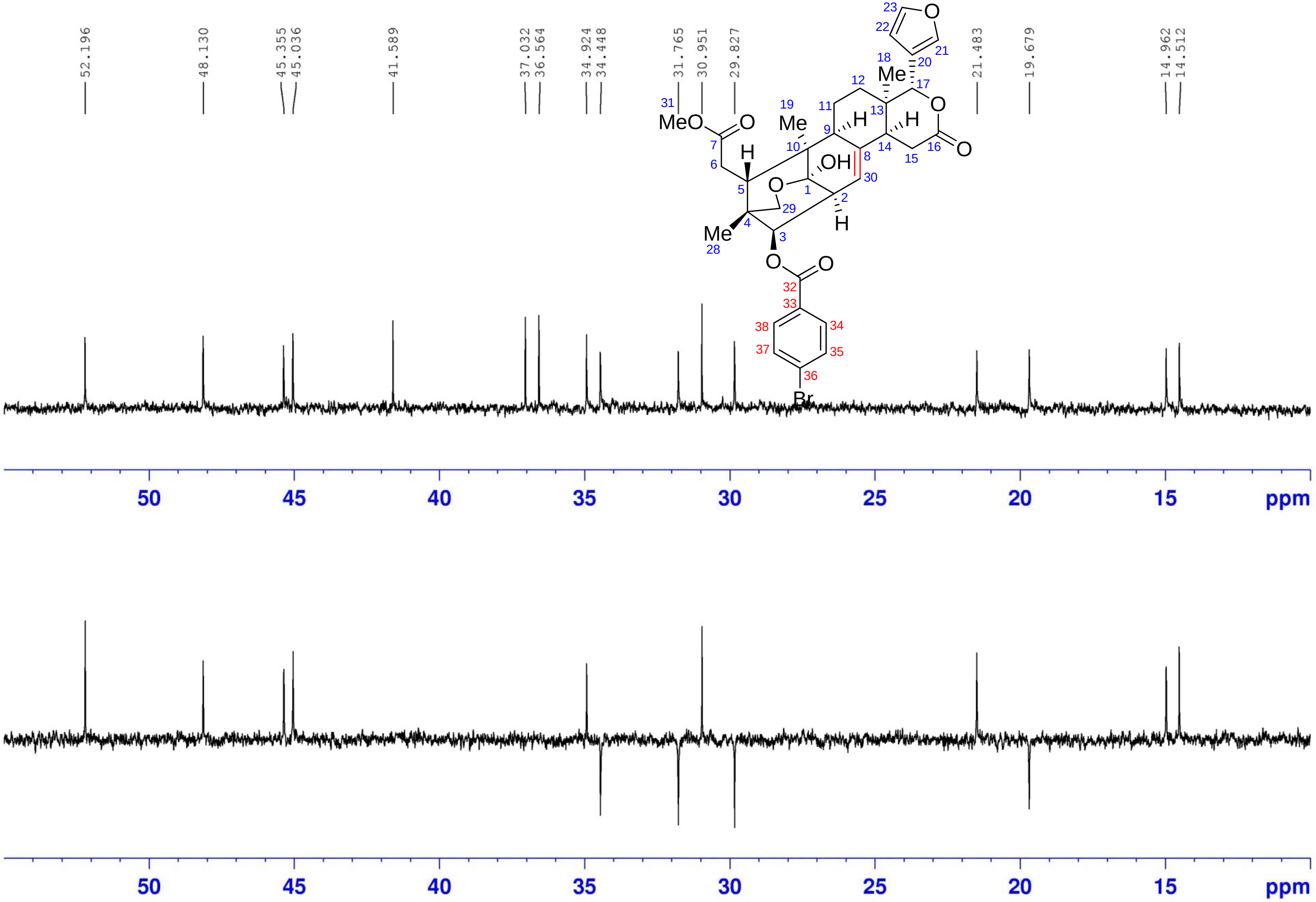

LR-ESIMS for compound **8c**

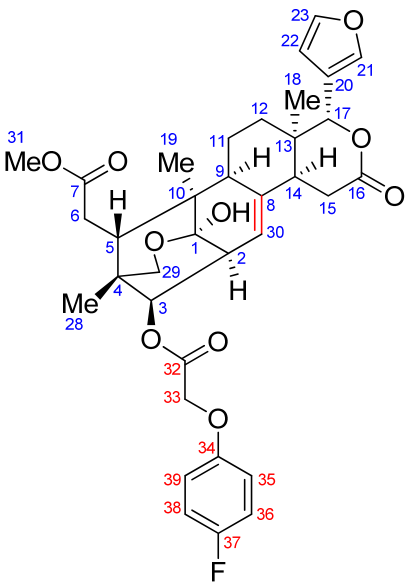

Generic Display Report

Analysis Info Acquisition D 2017/10/20 10:48:50

Analysis Name H:\Data-MS-20180909\liwanshan\CZC-1-CH3 4-F ben yang yi suan.d

Method DEF_MS.M Operator bdal

Sample Name CZC-1-CH3 4-F ben yang yi suan Instrument amaZon SL

Comment

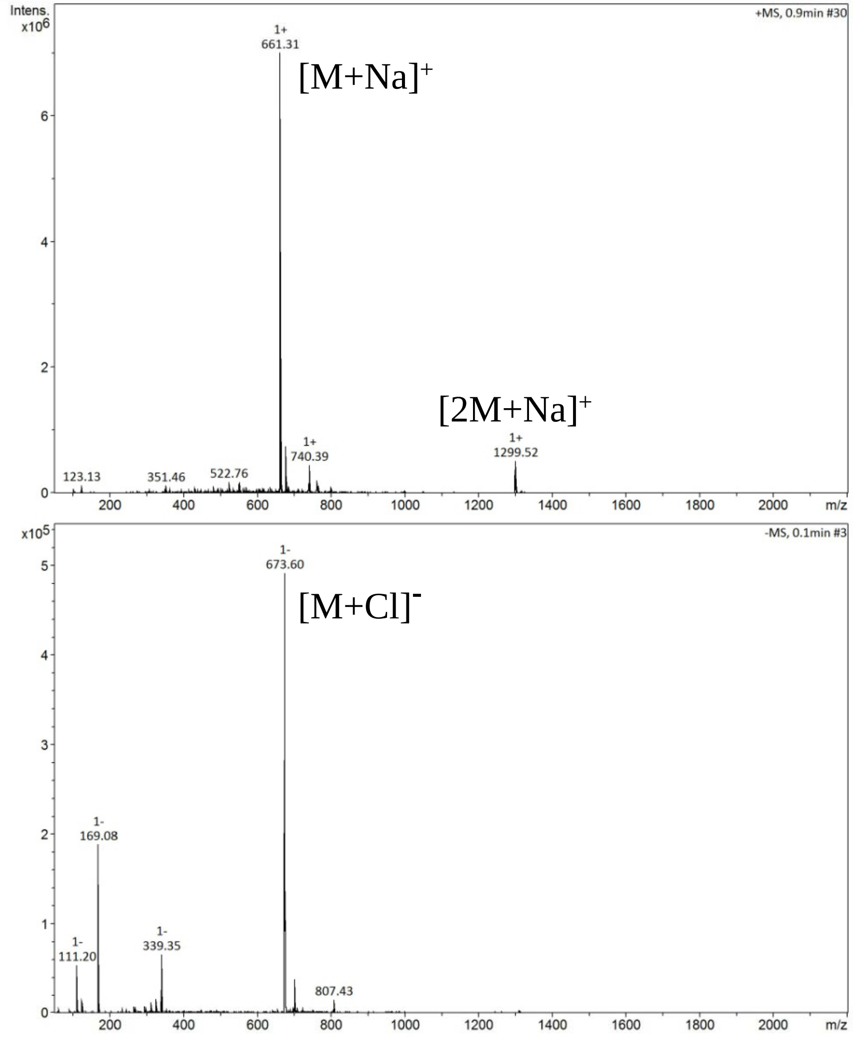

Bruker Compass DataAnalysis

^{1}H NMR (400 MHz) spectrum of **8c** in $CDCl_3$

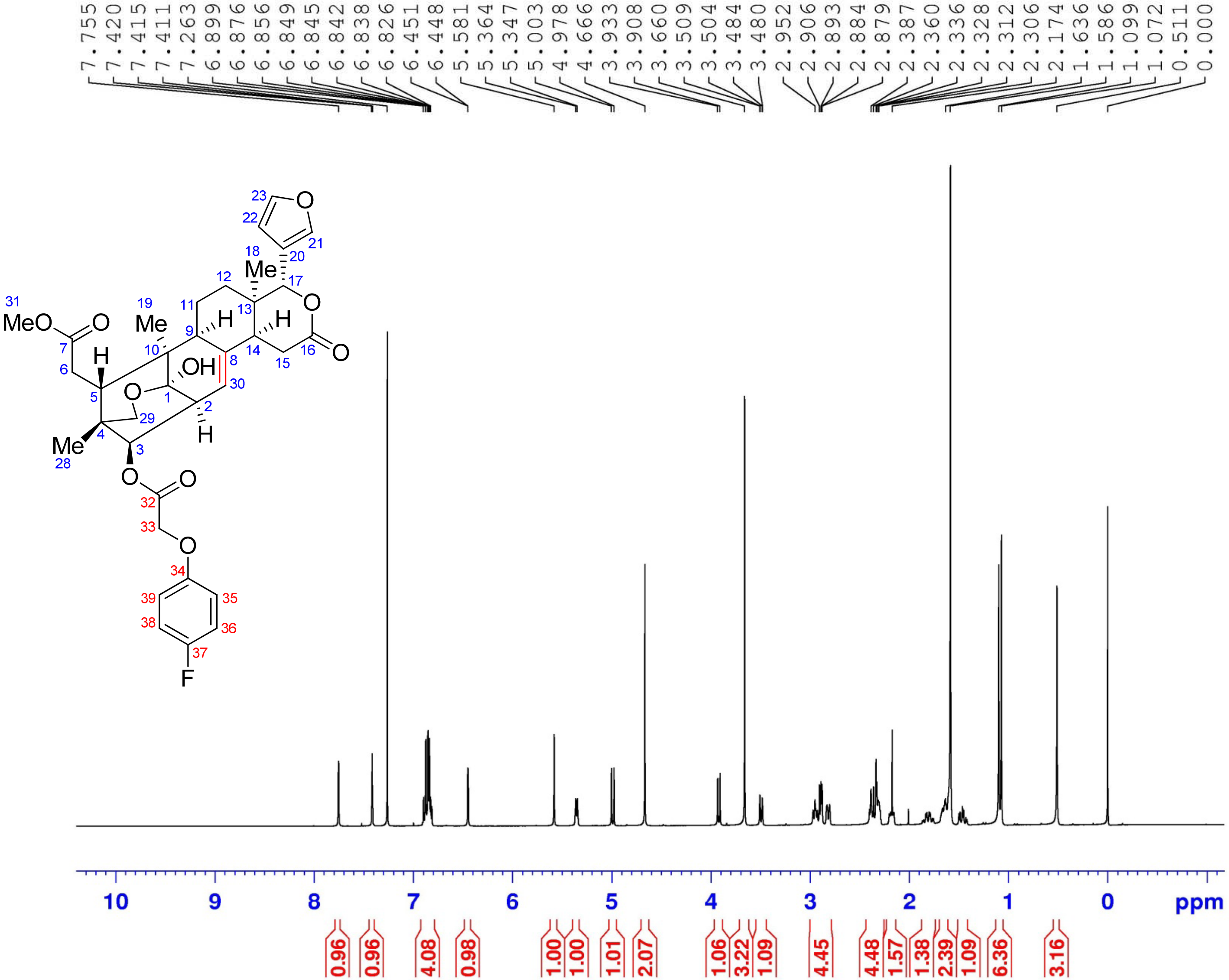


```
NAME      czc-1-CH3-4-F-YISU
EXPNO                  1
PROCNO                 1
Date_           20171017
Time               14.25
INSTRUM            spect
PROBHD   5 mm CPPBBO BB
PULPROG             zg30
TD                 65536
SOLVENT            CDCl3
NS                    16
DS                     2
SWH             8223.685 Hz
FIDRES          0.125483 Hz
AQ             3.9846387 sec
RG                 208.5
DW                60.800 use
DE                 10.00 use
TE                 297.0 K
D1            1.00000000 sec
TD0                    1

======== CHANNEL f1 =======
SFO1         400.1324710 MHz
NUC1                  1H
P1                 11.50 use
SI                 65536
SF           400.1300084 MHz
WDW                   EM
SSB                    0
LB                  0.30 Hz
GB                     0
PC                  1.00
```

^1H NMR (400 MHz) spectrum of **8c** in $CDCl_3$

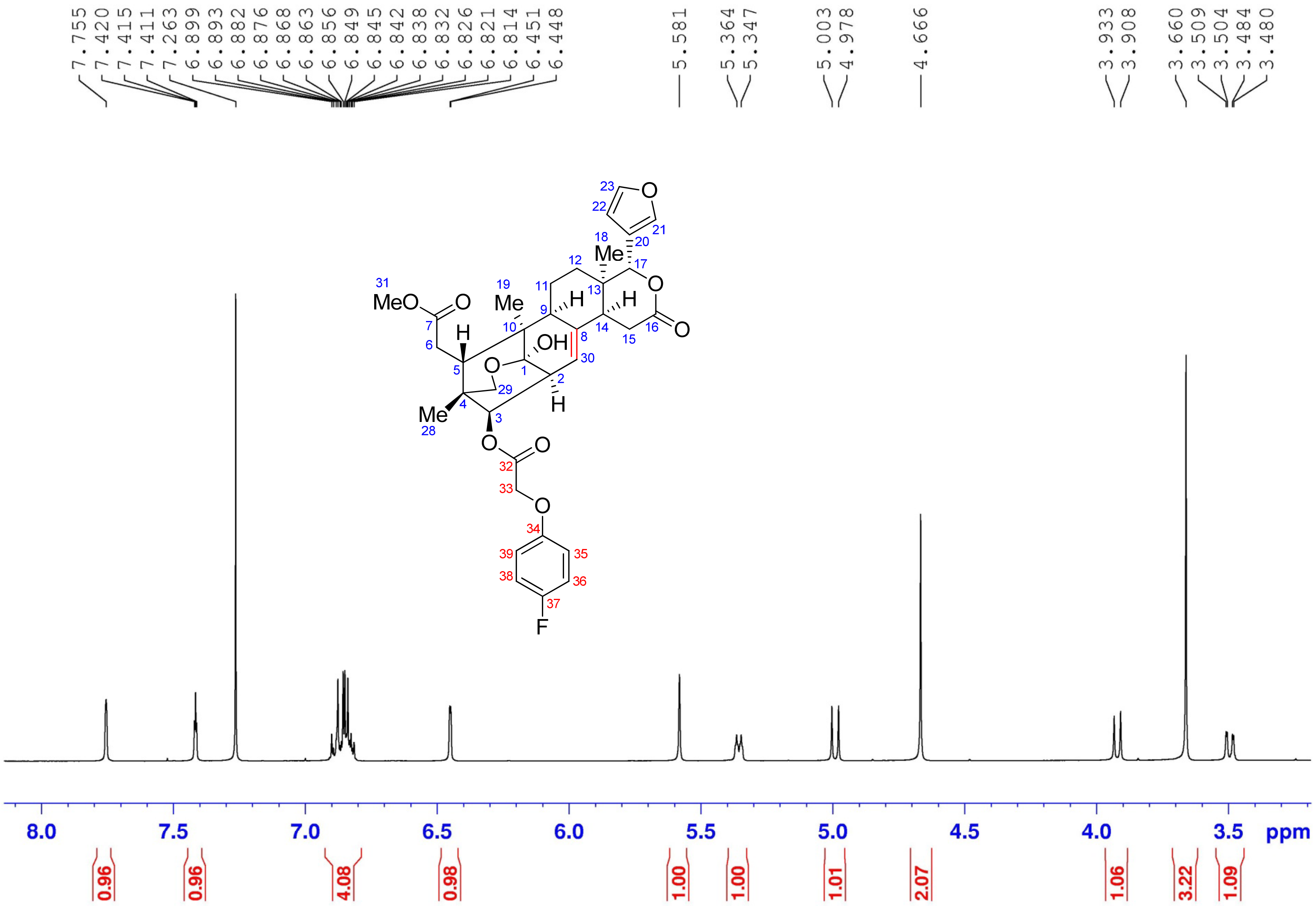

^{1}H NMR (400 MHz) spectrum of **8c** in $CDCl_3$

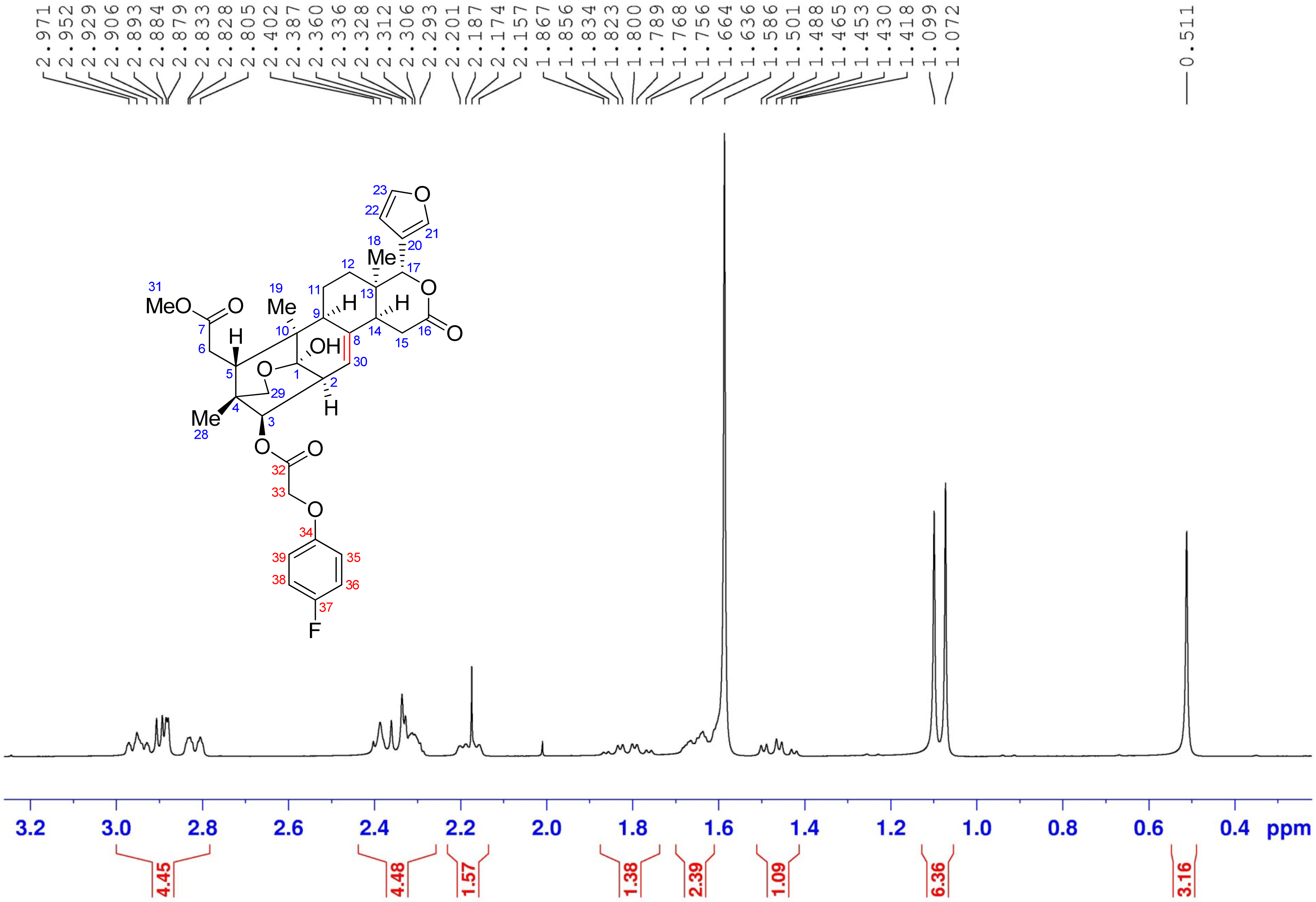

^{13}C NMR (100 MHz) spectrum of **8c** in $CDCl_3$

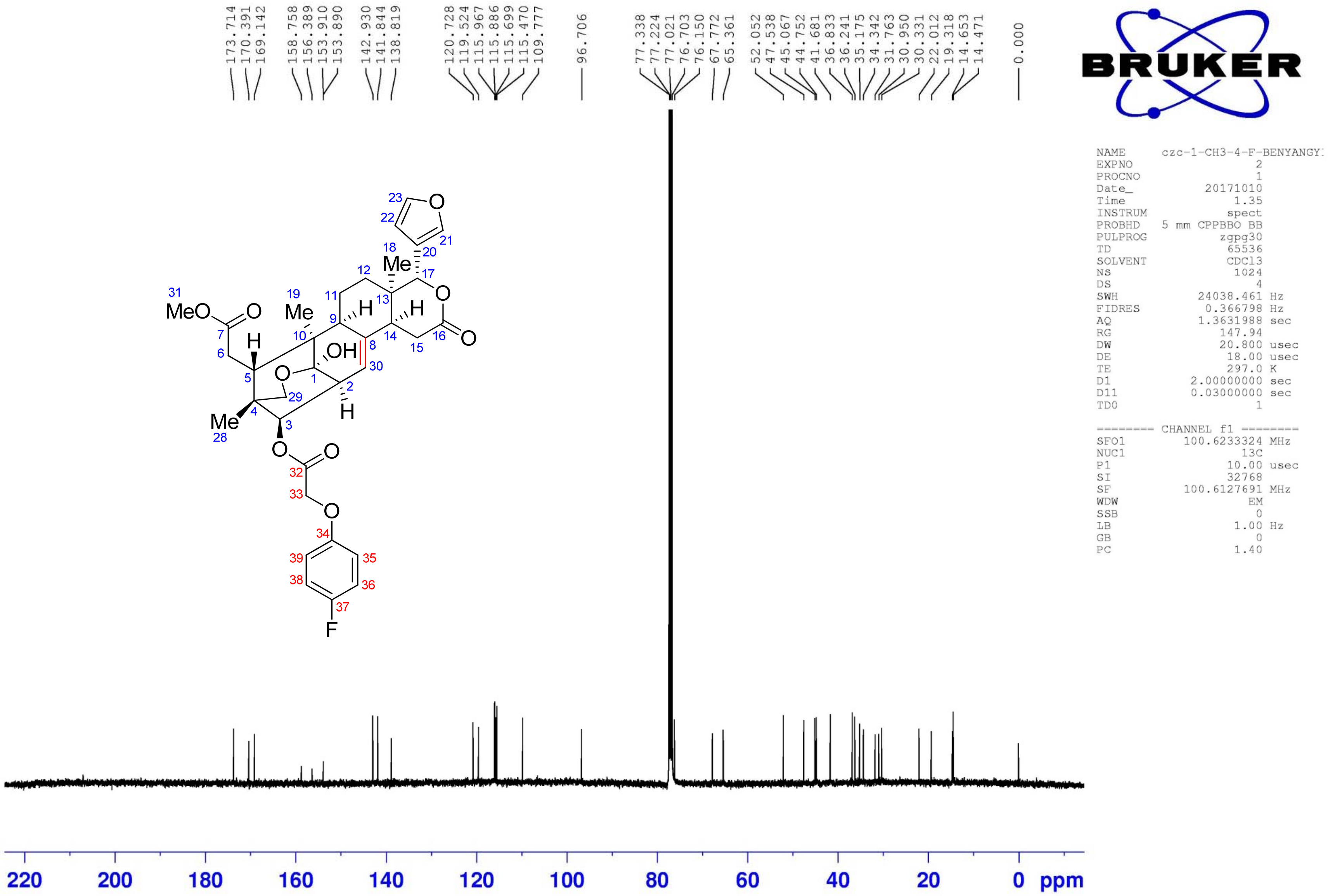

^{13}C NMR (100 MHz) spectrum of **8c** in $CDCl_3$

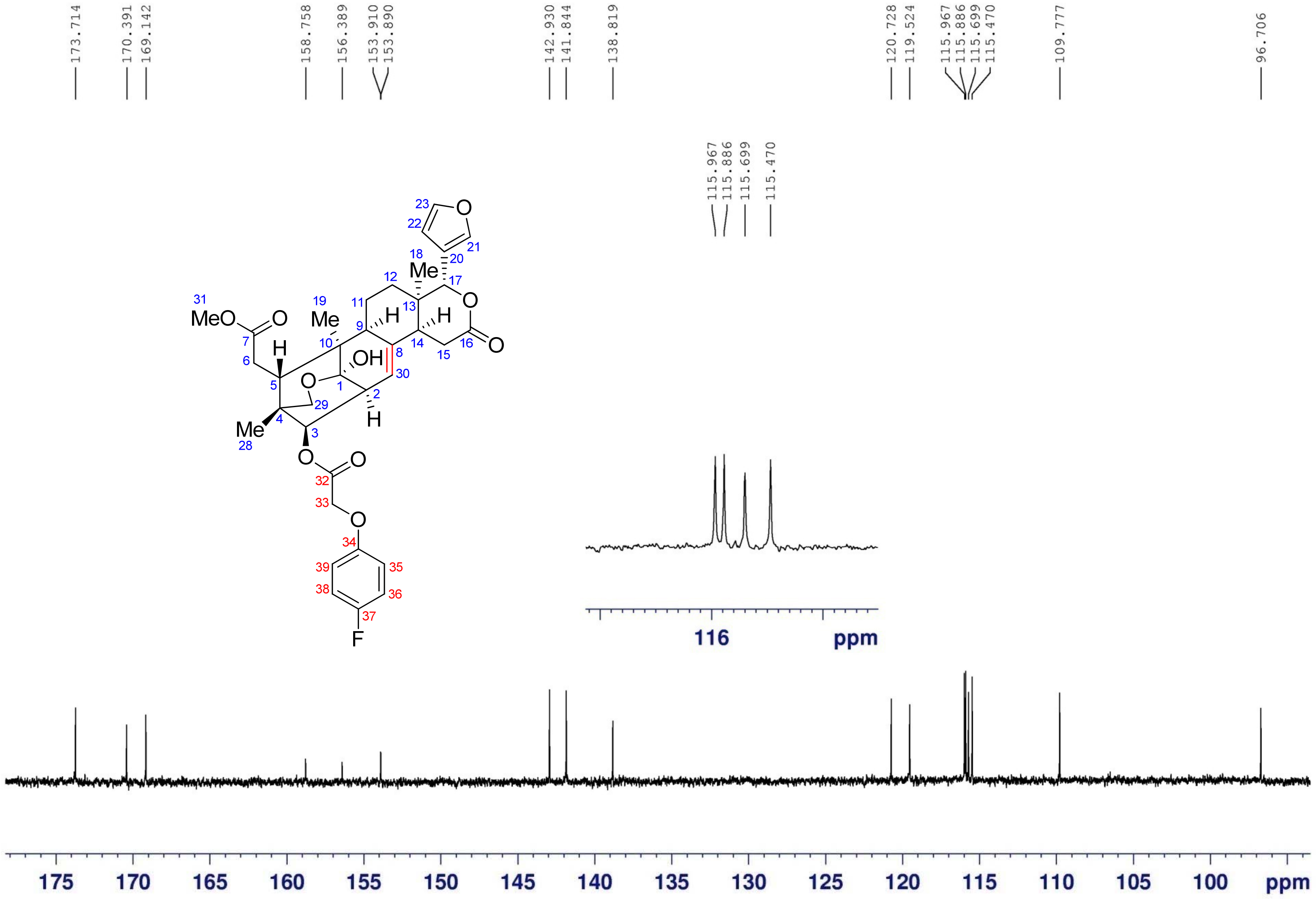

^{13}C NMR (100 MHz) spectrum of **8c** in $CDCl_3$

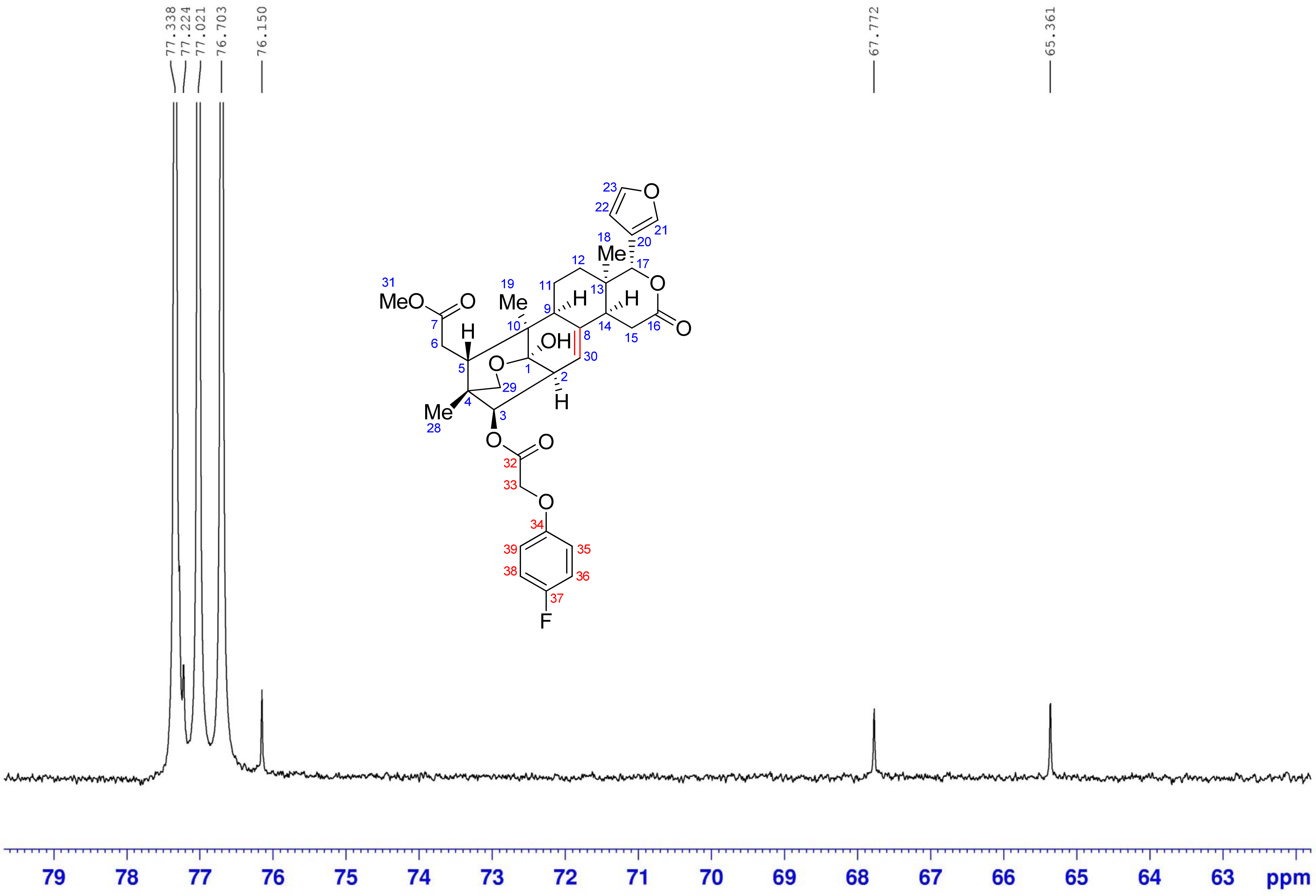

^{13}C NMR (100 MHz) spectrum of **8c** in $CDCl_3$

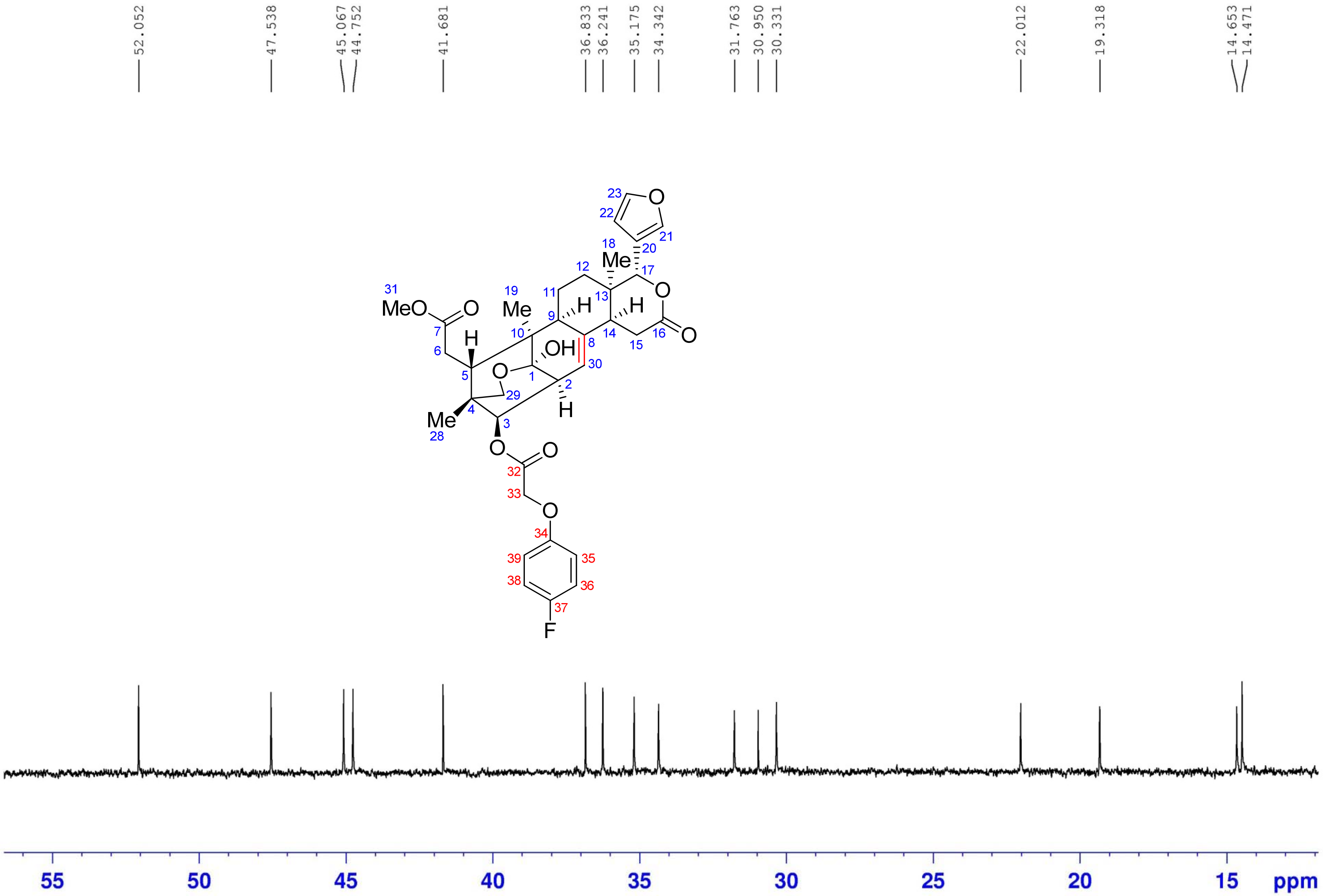

DEPT 135 spectrum of **8c** in $CDCl_3$

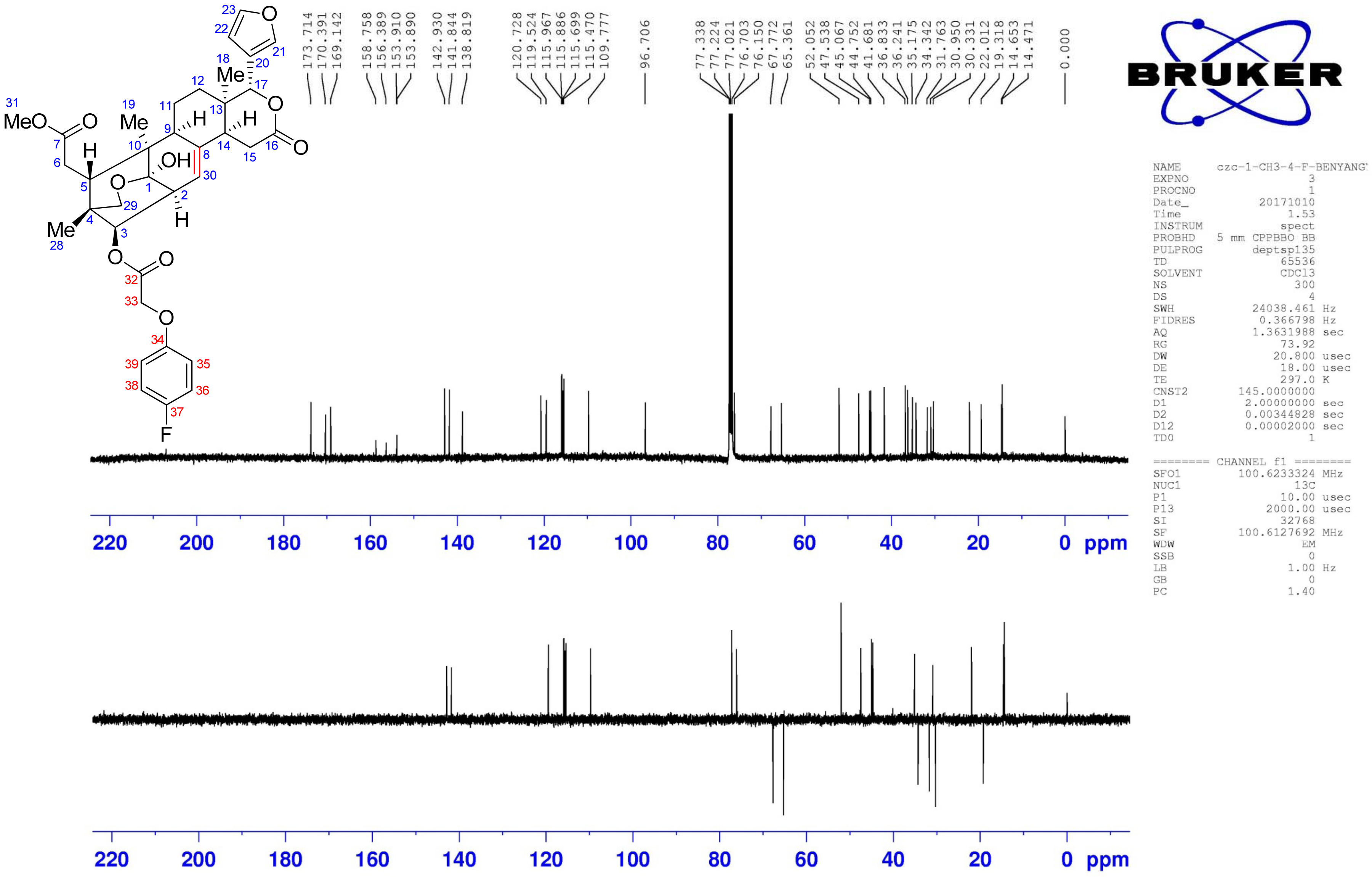

DEPT 135 spectrum of **8c** in $CDCl_3$

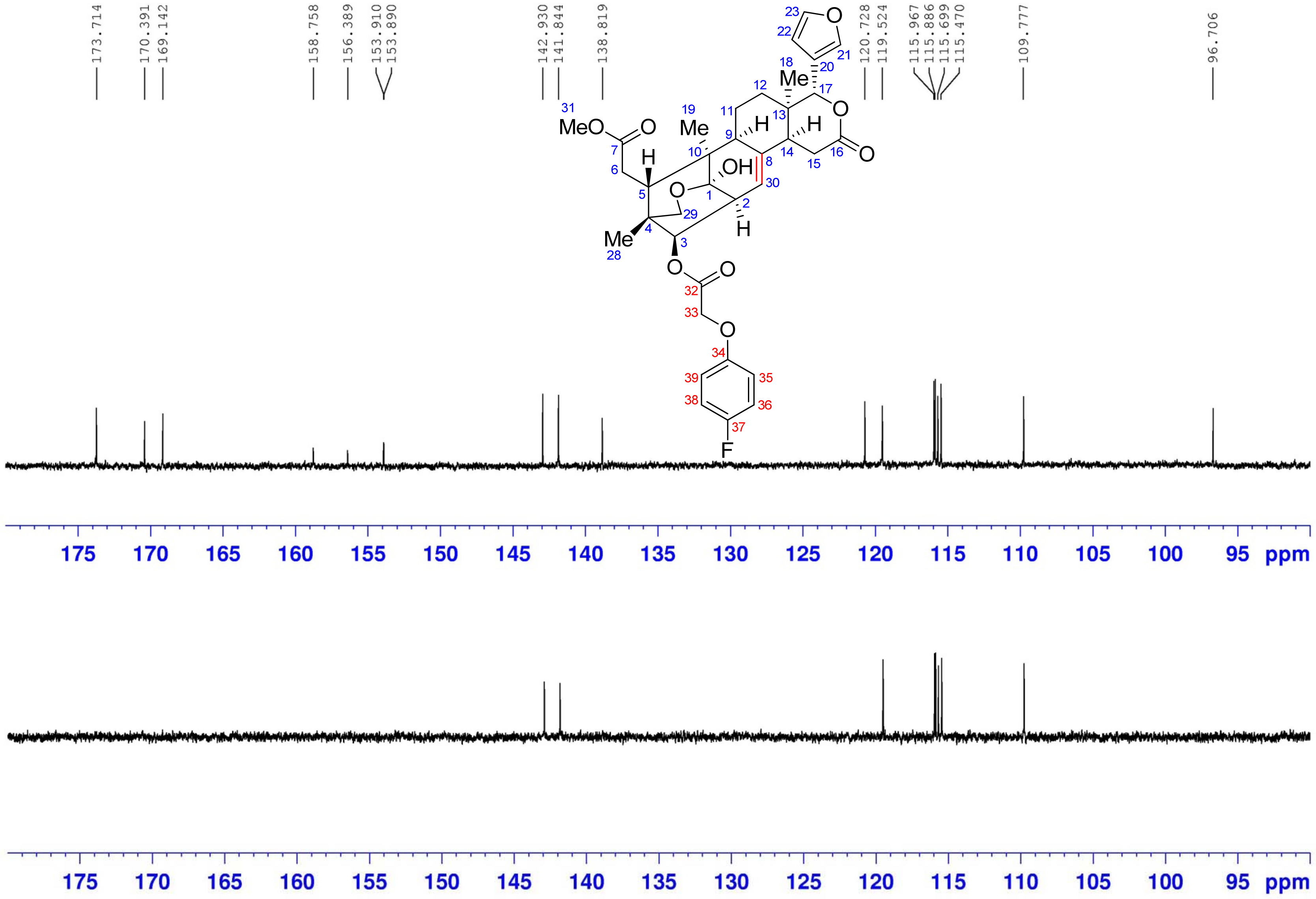

DEPT 135 spectrum of **8c** in $CDCl_3$

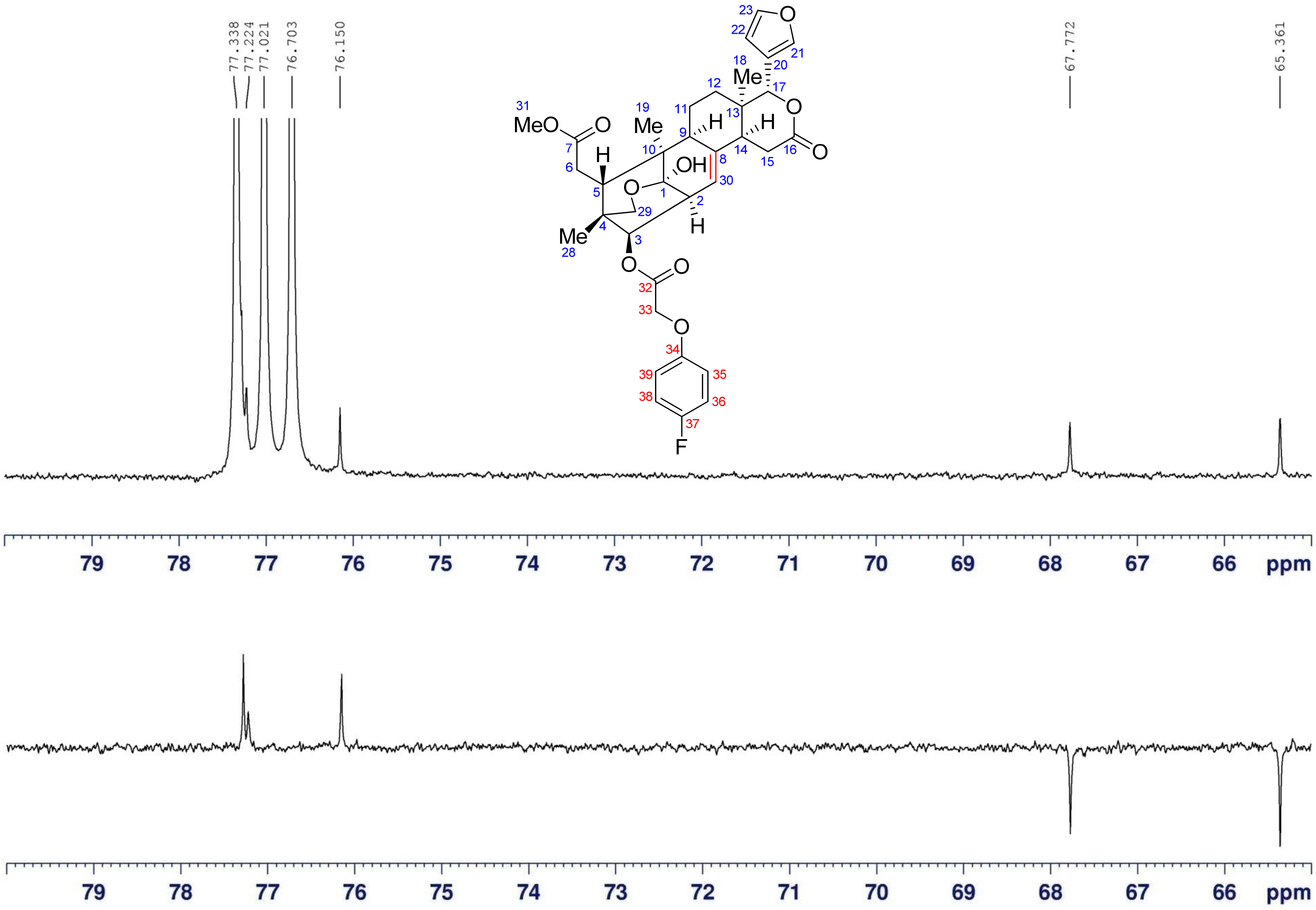

DEPT 135 spectrum of **8c** in $CDCl_3$

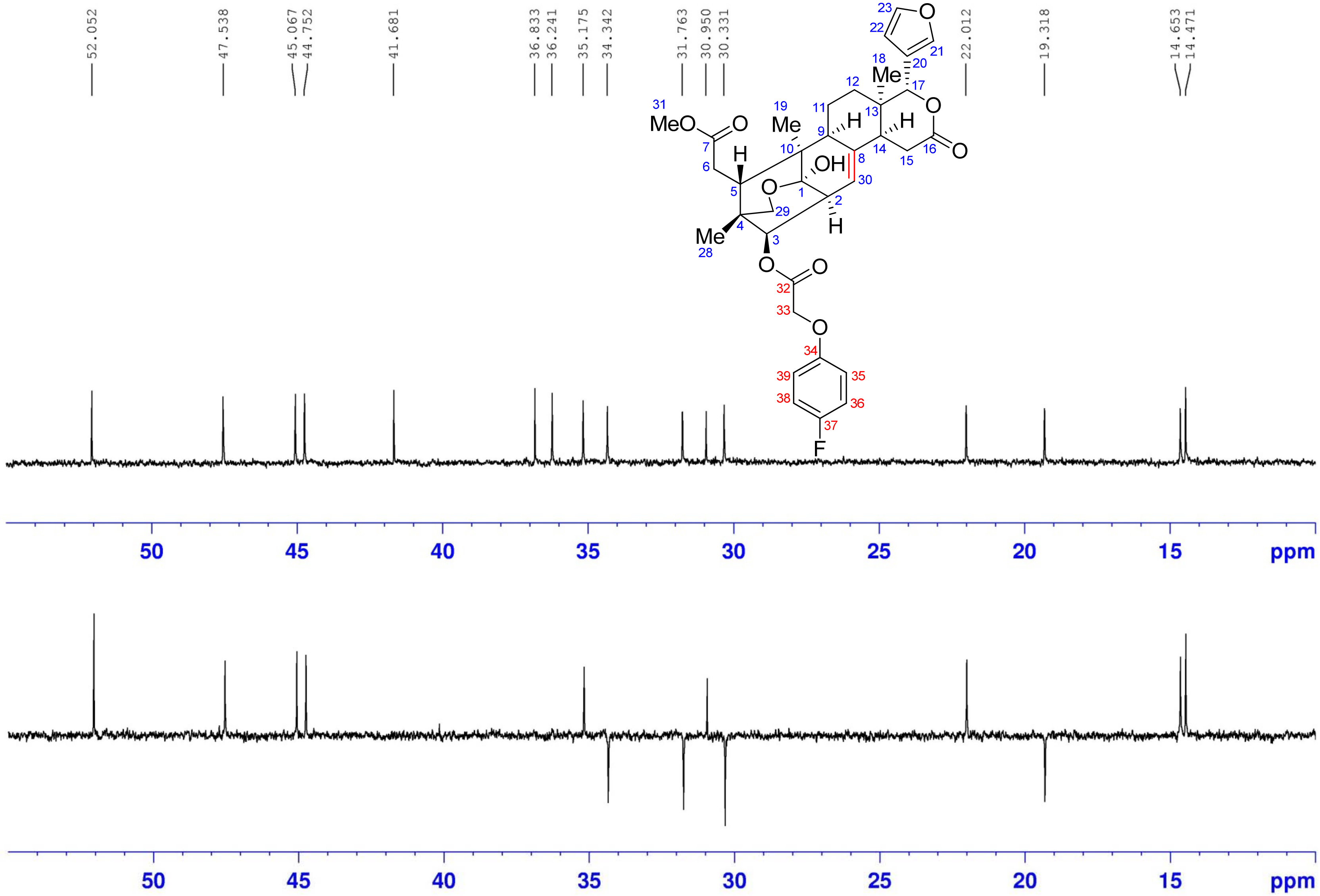

HR-ESIMS for compound **8d**

Generic Display Report

Analysis Info

Analysis Name	D:\Data\MS\data\201809\liwanshan_WSD-68_pos_14_01_5575.d	Acquisition Date	9/30/2018 6:05:28 PM
Method	LC_Direct Infusion_pos_100-1000mz.m	Operator	SCSIO
Sample Name	liwanshan_WSD-68_pos	Instrument	maXis
Comment			

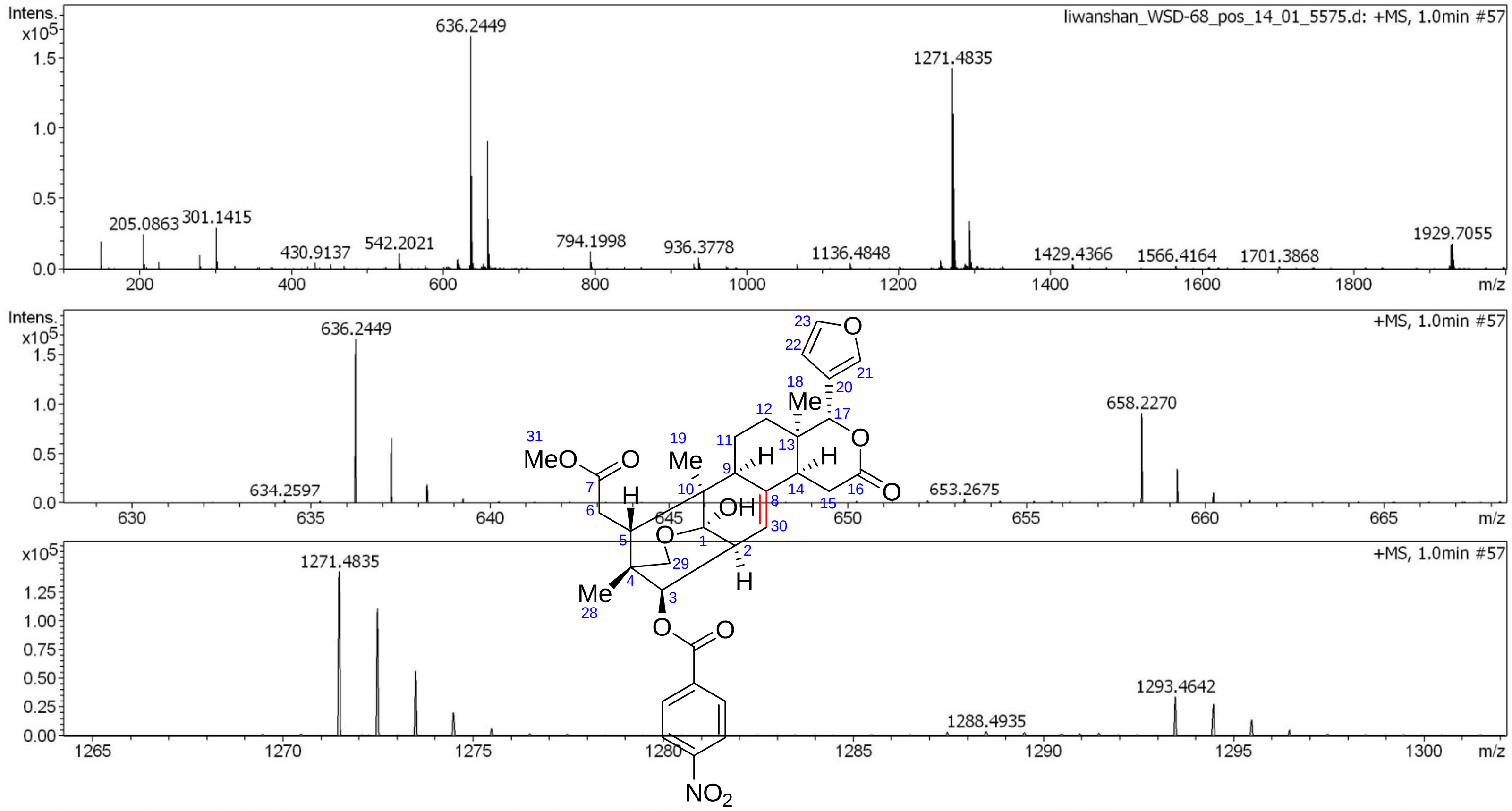

HR-ESIMS for compound **8d**

Mass Spectrum SmartFormula Report

Analysis Info

Analysis Name	D:\Data\MS\data\201809\liwanshan_WSD-68_pos_14_01_5575.d	Acquisition Date	9/30/2018 6:05:28 PM
Method	LC_Direct Infusion_pos_100-1000mz.m	Operator	SCSIO
Sample Name	liwanshan_WSD-68_pos	Instrument	maXis 255552.00029
Comment			

Acquisition Parameter

Source Type	ESI	Ion Polarity	Positive	Set Nebulizer	0.4 Bar
Focus	Active	Set Capillary	4500 V	Set Dry Heater	180 °C
Scan Begin	100 m/z	Set End Plate Offset	-500 V	Set Dry Gas	4.0 l/min
Scan End	2000 m/z	Set Charging Voltage	0 V	Set Divert Valve	Waste
		Set Corona	0 nA	Set APCI Heater	0 °C

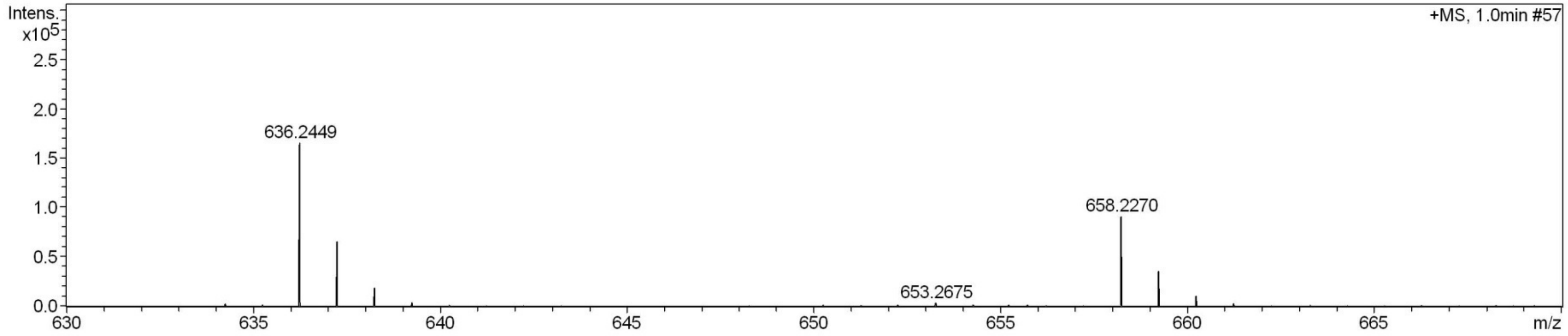

Meas. m/z	#	Ion Formula	Score	m/z	err [ppm]	err [mDa]	mSigma	rdb	e⁻ Conf	N-Rule
636.2449	1	C34H38NO11	100.00	636.2439	-1.5	-0.9	16.8	16.5	even	ok
658.2270	1	C34H37NNaO11	100.00	658.2259	-1.6	-1.1	14.2	16.5	even	ok
1271.4835	1	C68H75N2O22	77.91	1271.4806	-2.3	-2.9	33.3	32.5	even	ok
1293.4642	1	C68H74N2NaO22	100.00	1293.4625	-1.3	-1.7	41.6	32.5	even	ok

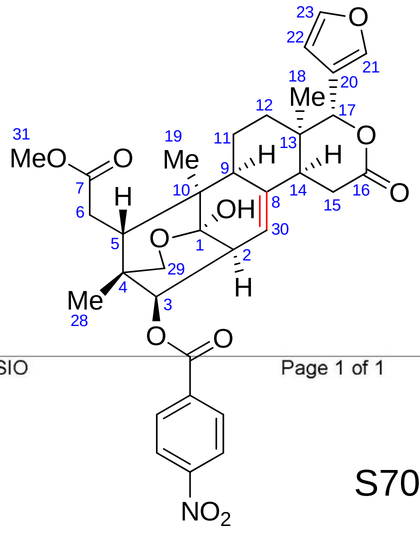

^{1}H NMR (400 MHz) spectrum of **8d** in $CDCl_3$

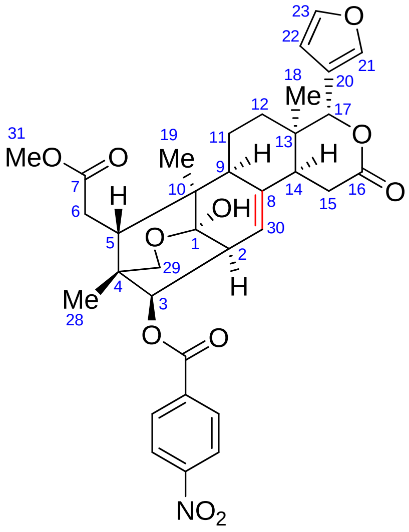

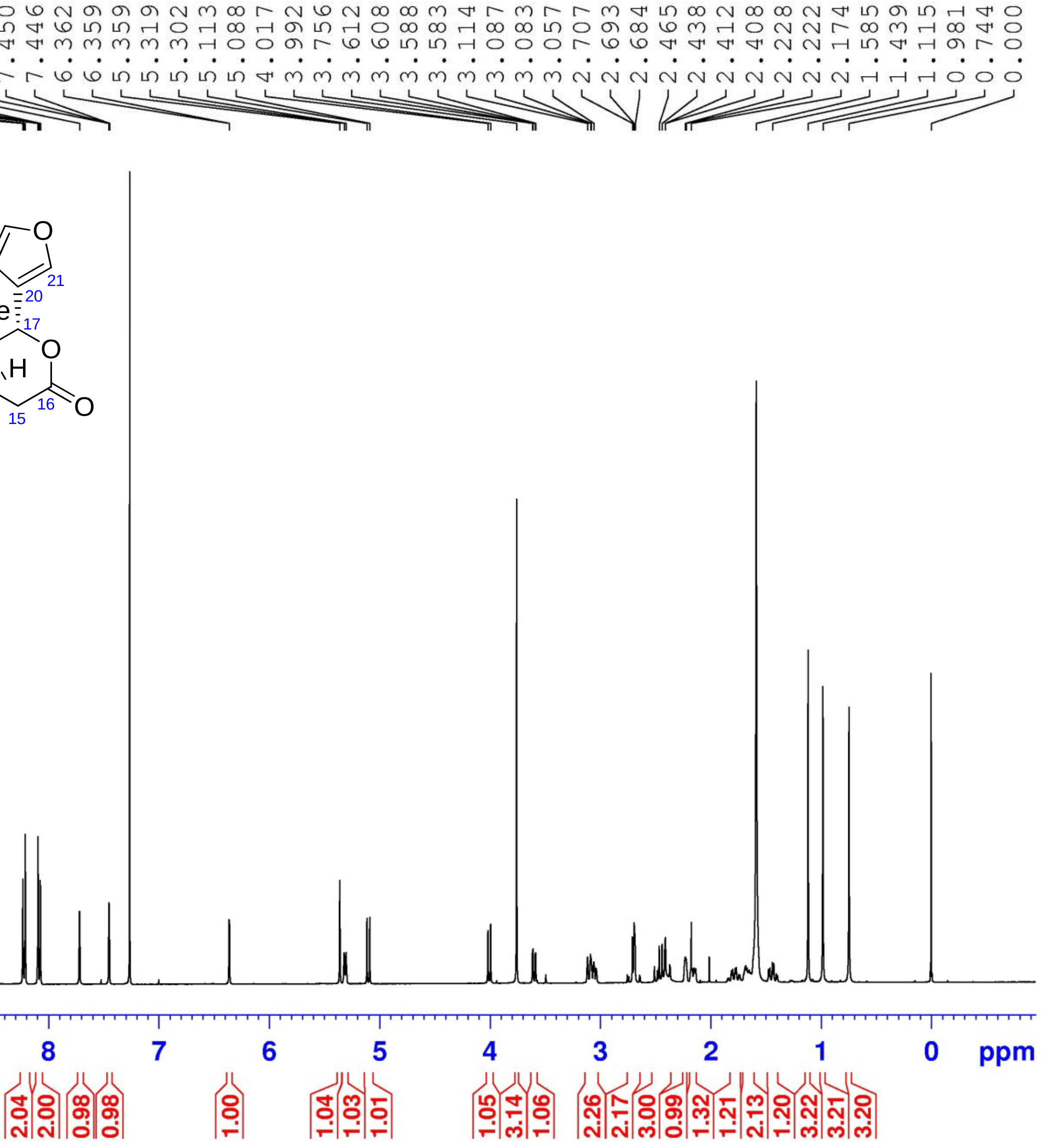

NAME	czc-1-CH3-duixia
EXPNO	1
PROCNO	1
Date_	20170922
Time	23.09
INSTRUM	spect
PROBHD	5 mm CPPBBO BB
PULPROG	zg30
TD	65536
SOLVENT	CDCl3
NS	16
DS	2
SWH	8223.685 H
FIDRES	0.125483 H
AQ	3.9846387 s
RG	208.5
DW	60.800 u
DE	10.00 u
TE	297.0 K
D1	1.00000000 s
TD0	1

======== CHANNEL f1 =====

SFO1	400.1324710 M
NUC1	1H
P1	11.50 u
SI	65536
SF	400.1300083 M
WDW	EM
SSB	0
LB	0.30 H
GB	0
PC	1.00

^{1}H NMR (400 MHz) spectrum of **8d** in $CDCl_3$

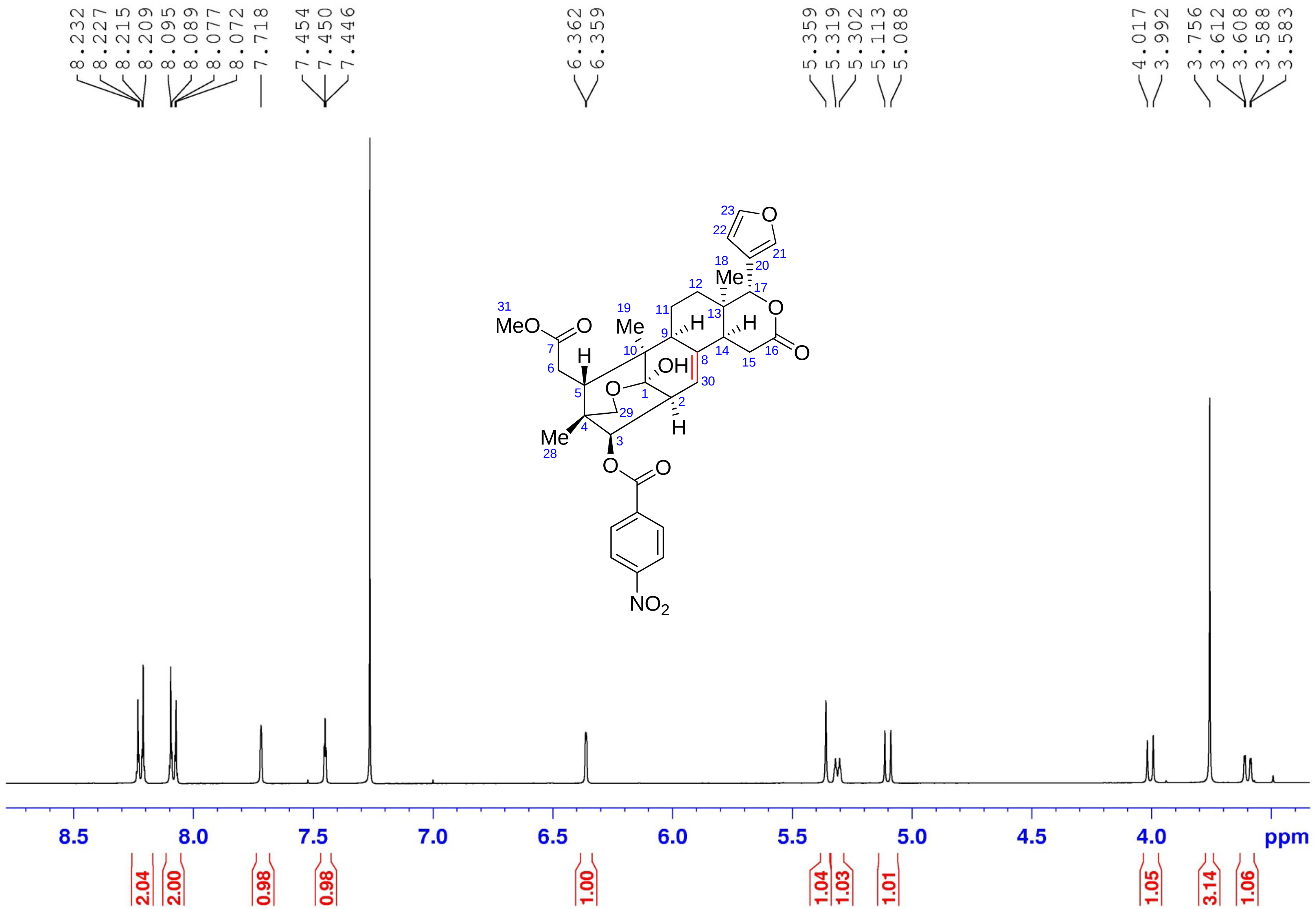

^1H NMR (400 MHz) spectrum of **8d** in $CDCl_3$

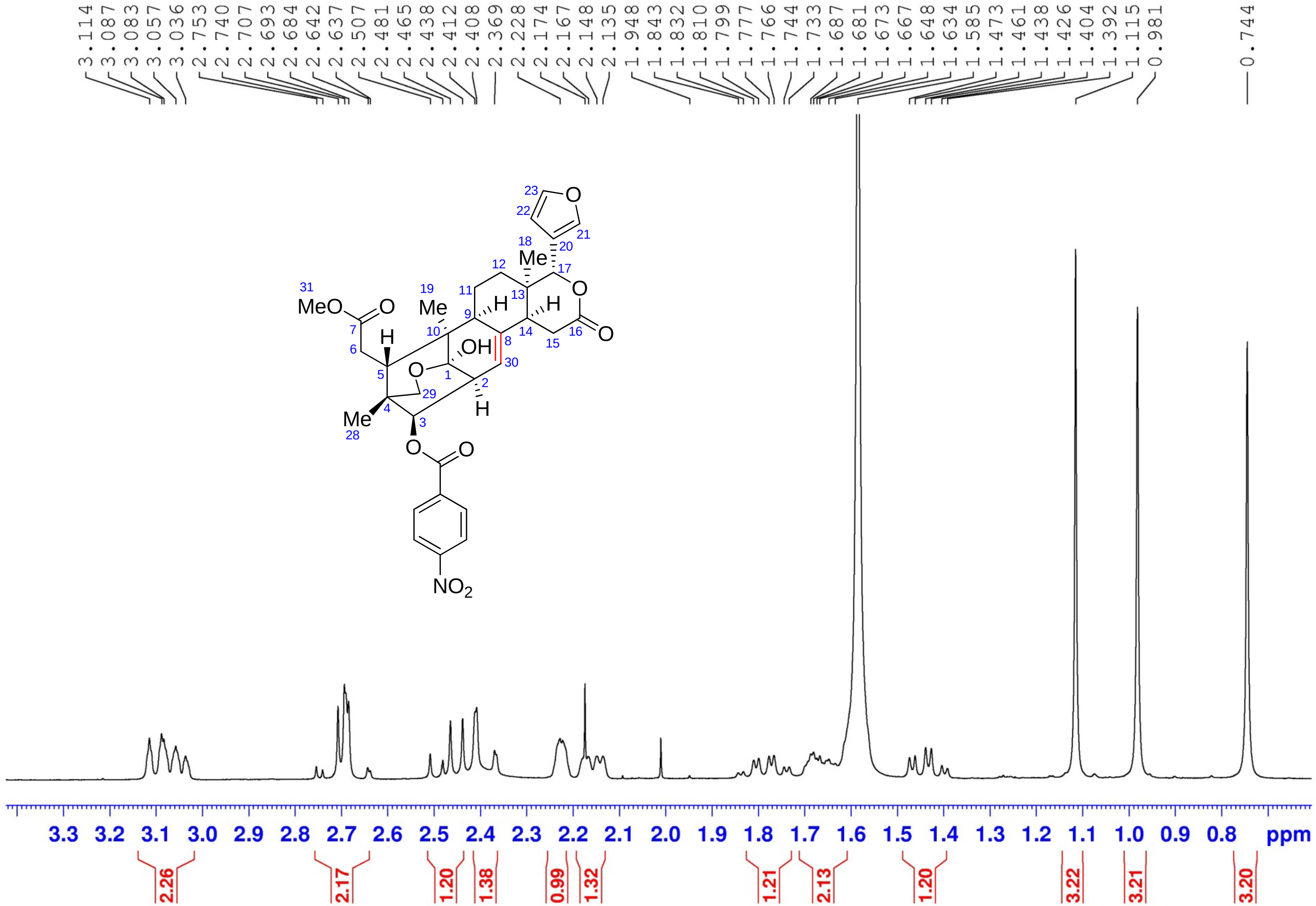

^{13}C NMR (100 MHz) spectrum of **8d** in $CDCl_3$

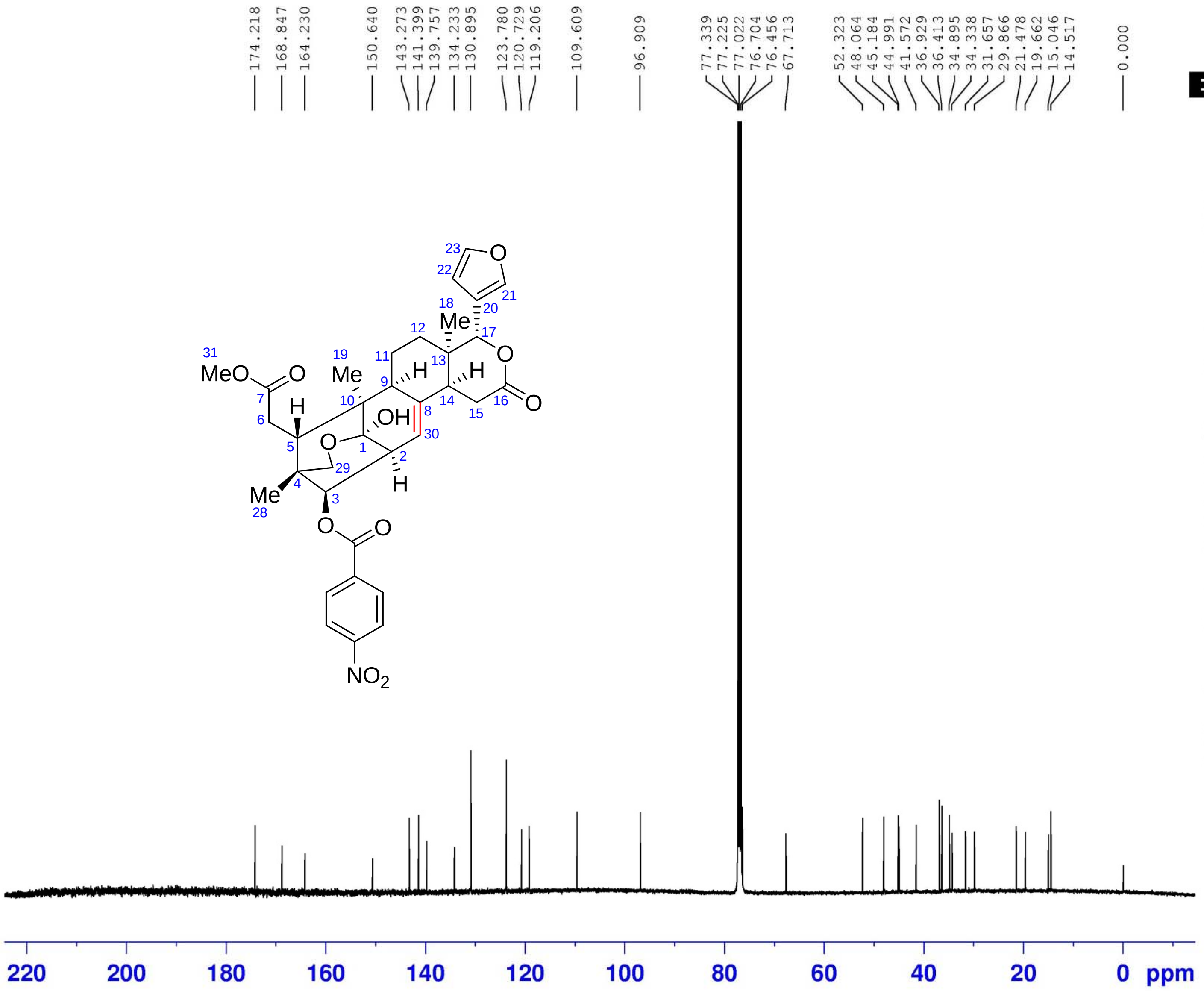

NAME	czc-1-CH3-duixia
EXPNO	2
PROCNO	1
Date_	20170923
Time	8.42
INSTRUM	spect
PROBHD	5 mm CPPBBO BB
PULPROG	zgpg30
TD	65536
SOLVENT	CDCl3
NS	10000
DS	4
SWH	24038.461 H
FIDRES	0.366798 H
AQ	1.3631988 s
RG	130.26
DW	20.800 u
DE	18.00 u
TE	297.0 K
D1	2.00000000 s
D11	0.03000000 s
TD0	1

======== CHANNEL f1 =====

SFO1	100.6233324 M
NUC1	13C
P1	10.00 u
SI	32768
SF	100.6127690 M
WDW	EM
SSB	0
LB	1.00 H
GB	0
PC	1.40

^{13}C NMR (100 MHz) spectrum of **8d** in $CDCl_3$

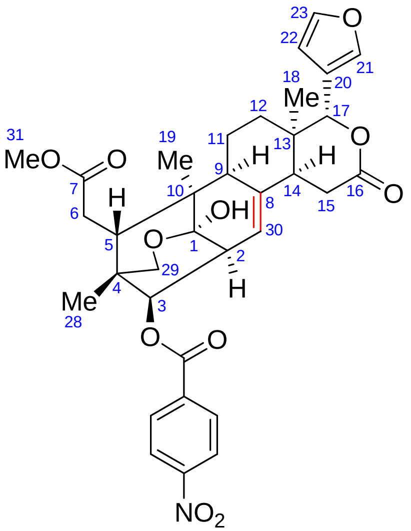

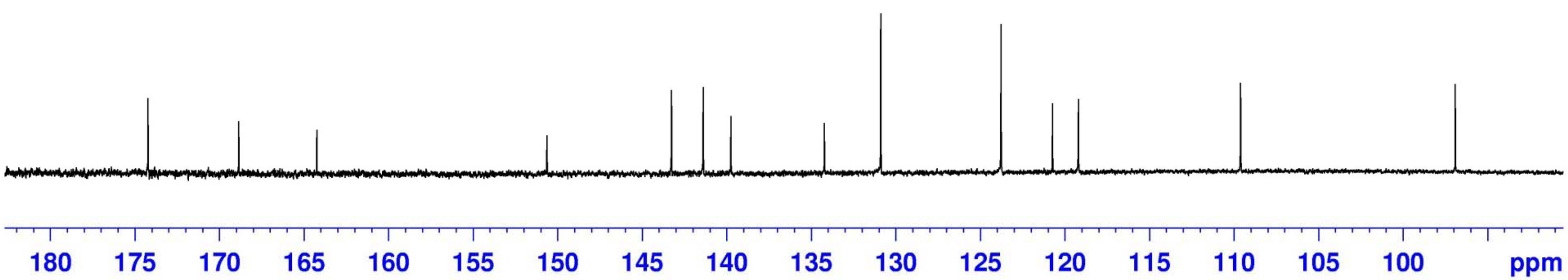

^{13}C NMR (100 MHz) spectrum of **8d** in $CDCl_3$

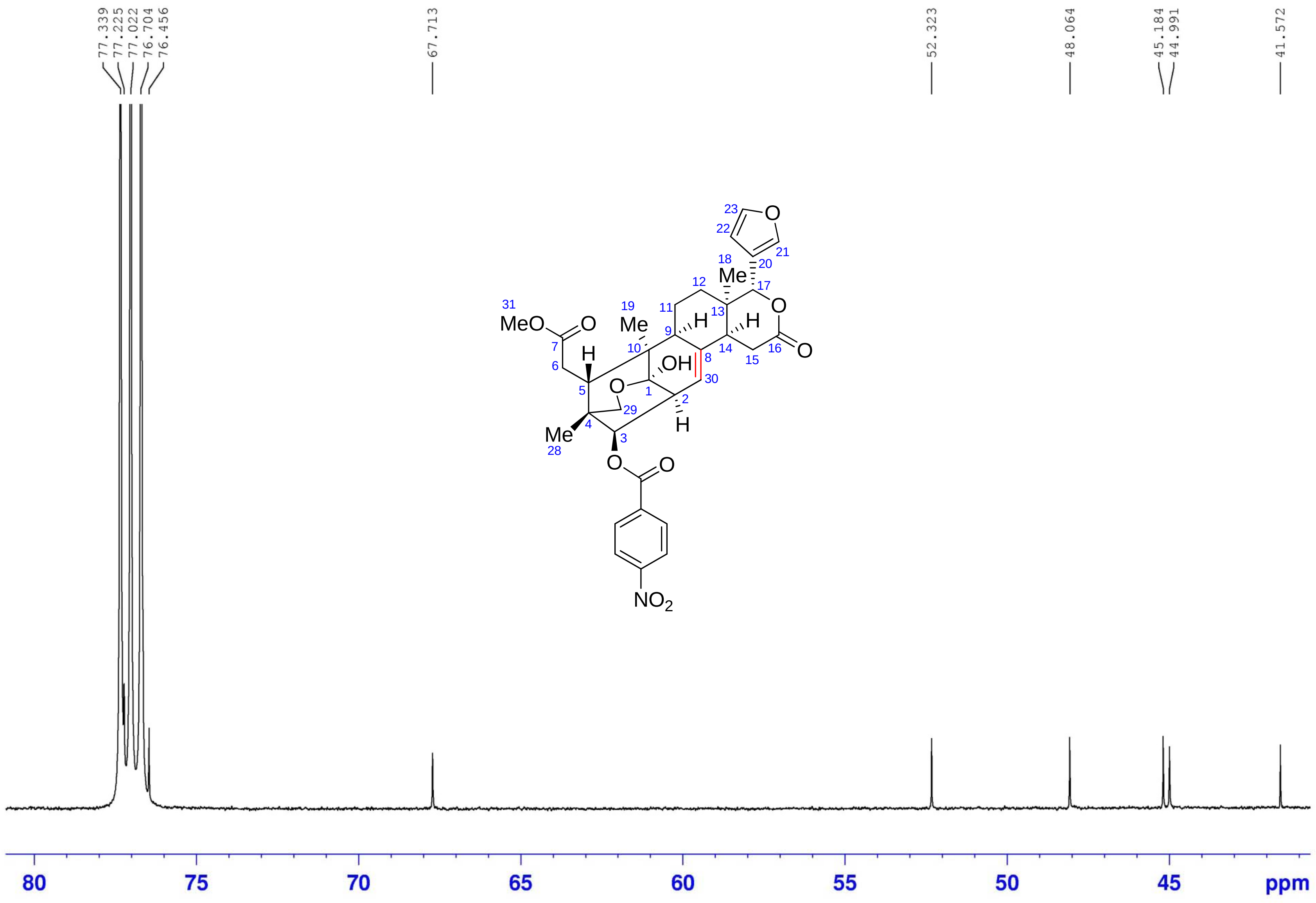

^{13}C NMR (100 MHz) spectrum of **8d** in $CDCl_3$

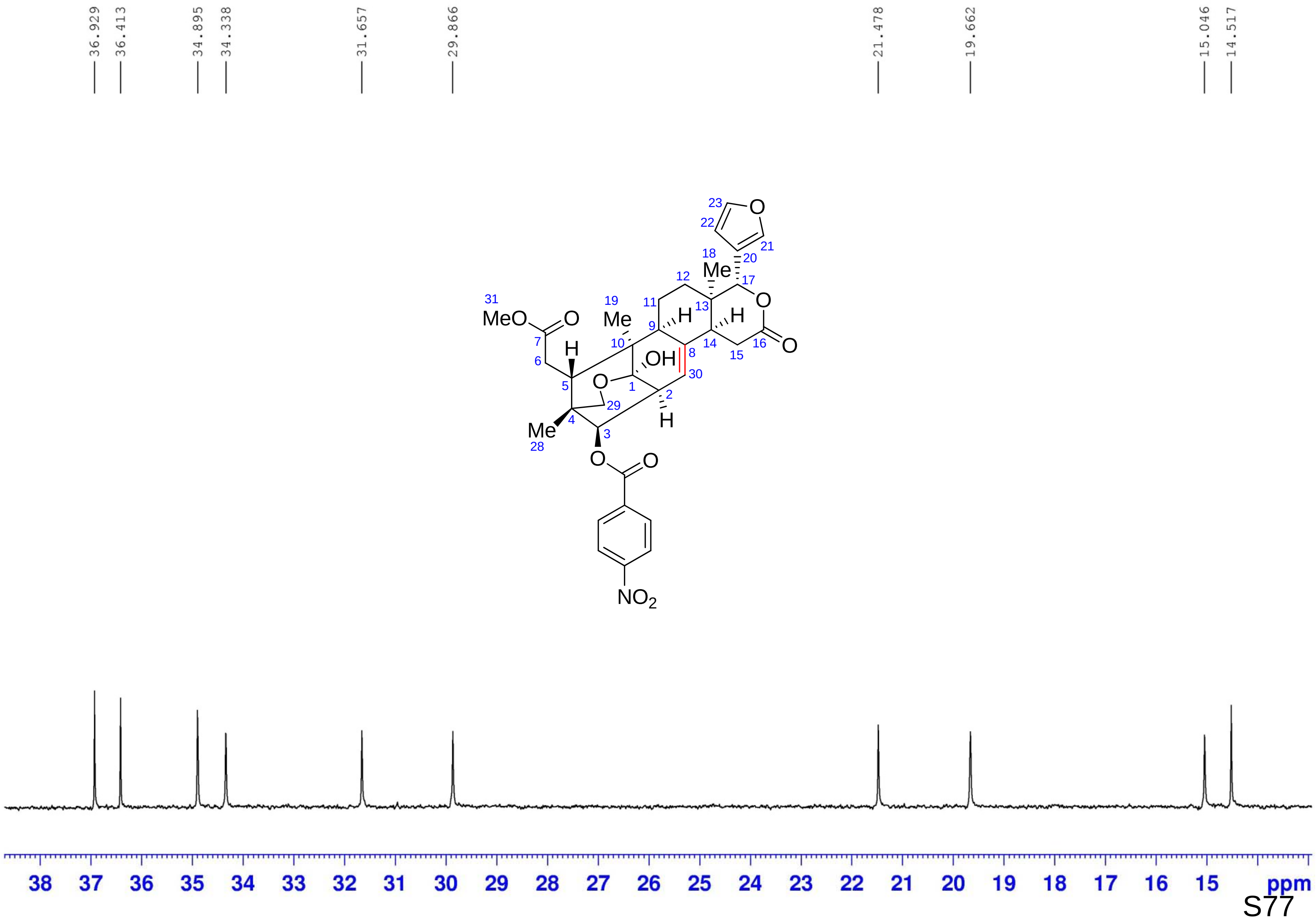

DEPT 135 spectrum of **8d** in $CDCl_3$

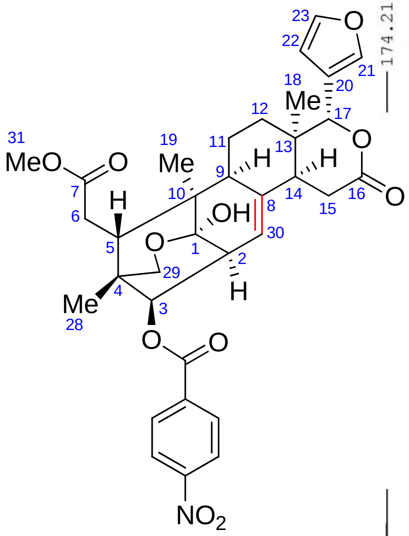

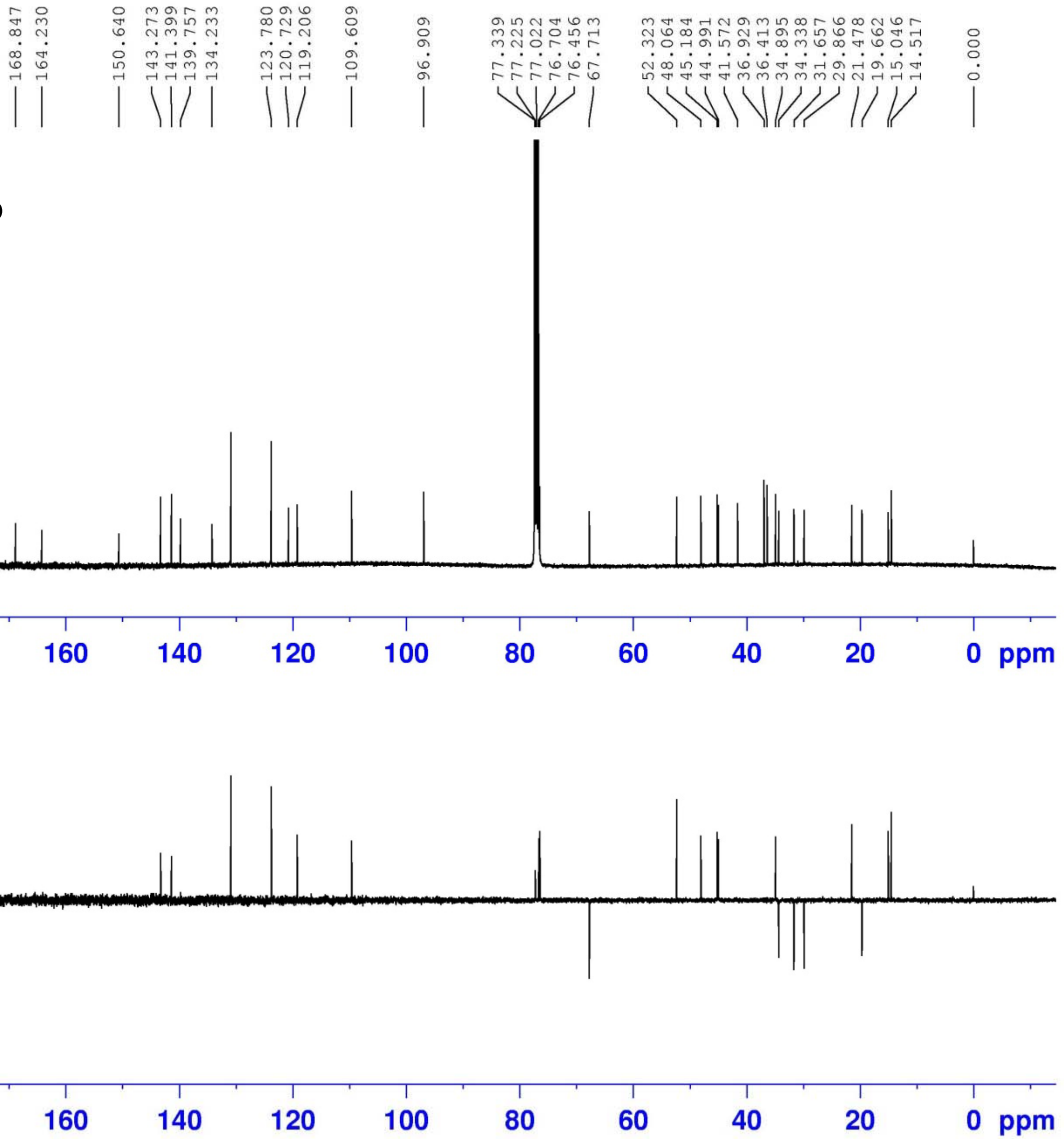

NAME	czc-1-CH3-duixia
EXPNO	3
PROCNO	1
Date_	20170923
Time	11.06
INSTRUM	spect
PROBHD	5 mm CPPBBO BB
PULPROG	deptsp135
TD	65536
SOLVENT	CDCl3
NS	2500
DS	4
SWH	24038.461 H
FIDRES	0.366798 H
AQ	1.3631988 s
RG	130.26
DW	20.800 u
DE	18.00 u
TE	297.0 K
CNST2	145.0000000
D1	2.00000000 s
D2	0.00344828 s
D12	0.00002000 s
TD0	1
======== CHANNEL f1 =====	
SFO1	100.6233324 M
NUC1	13C
P1	10.00 u
P13	2000.00 u
SI	32768
SF	100.6127691 M
WDW	EM
SSB	0
LB	1.00 H
GB	0
PC	1.40

DEPT 135 spectrum of **8d** in $CDCl_3$

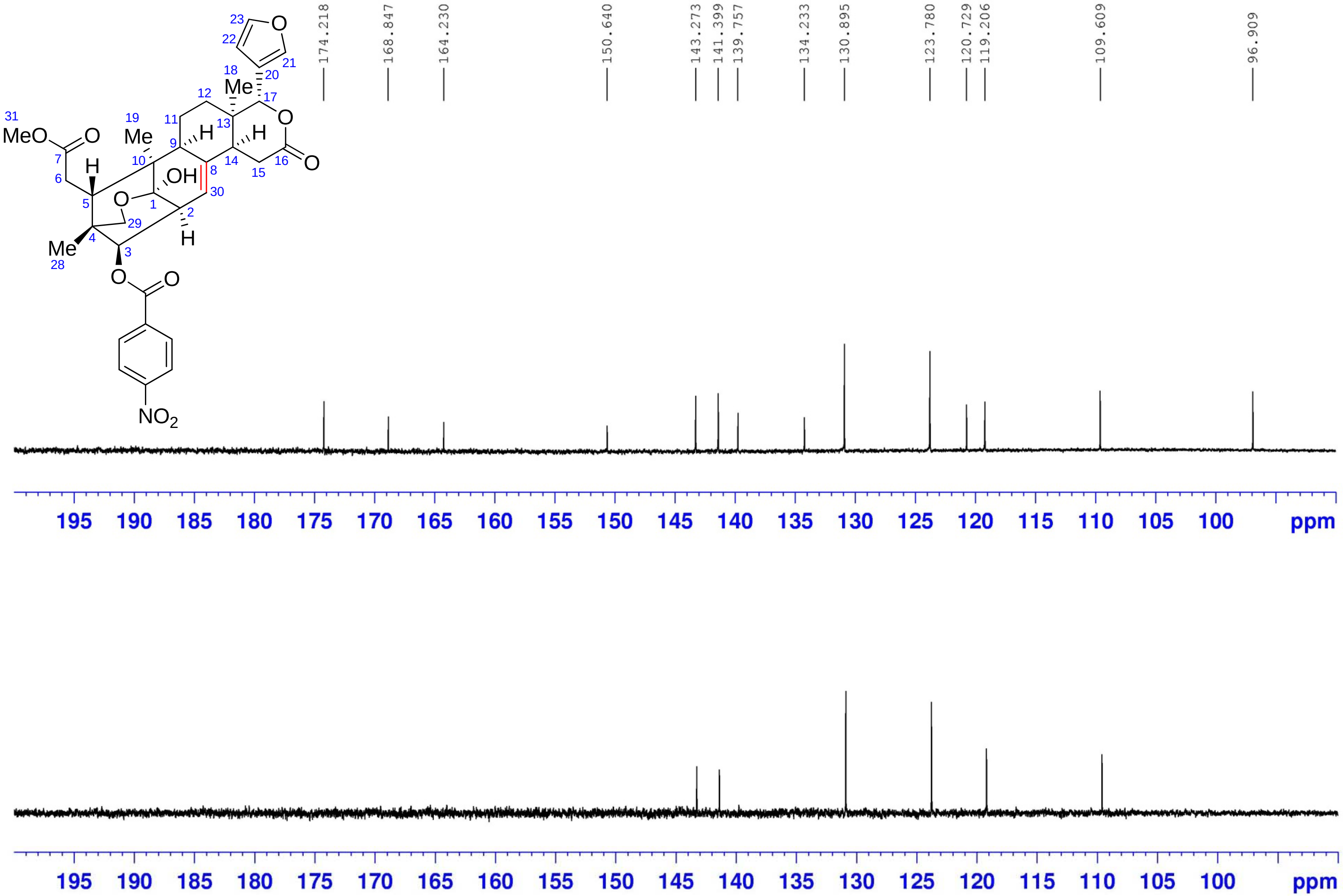

DEPT 135 spectrum of **8d** in $CDCl_3$

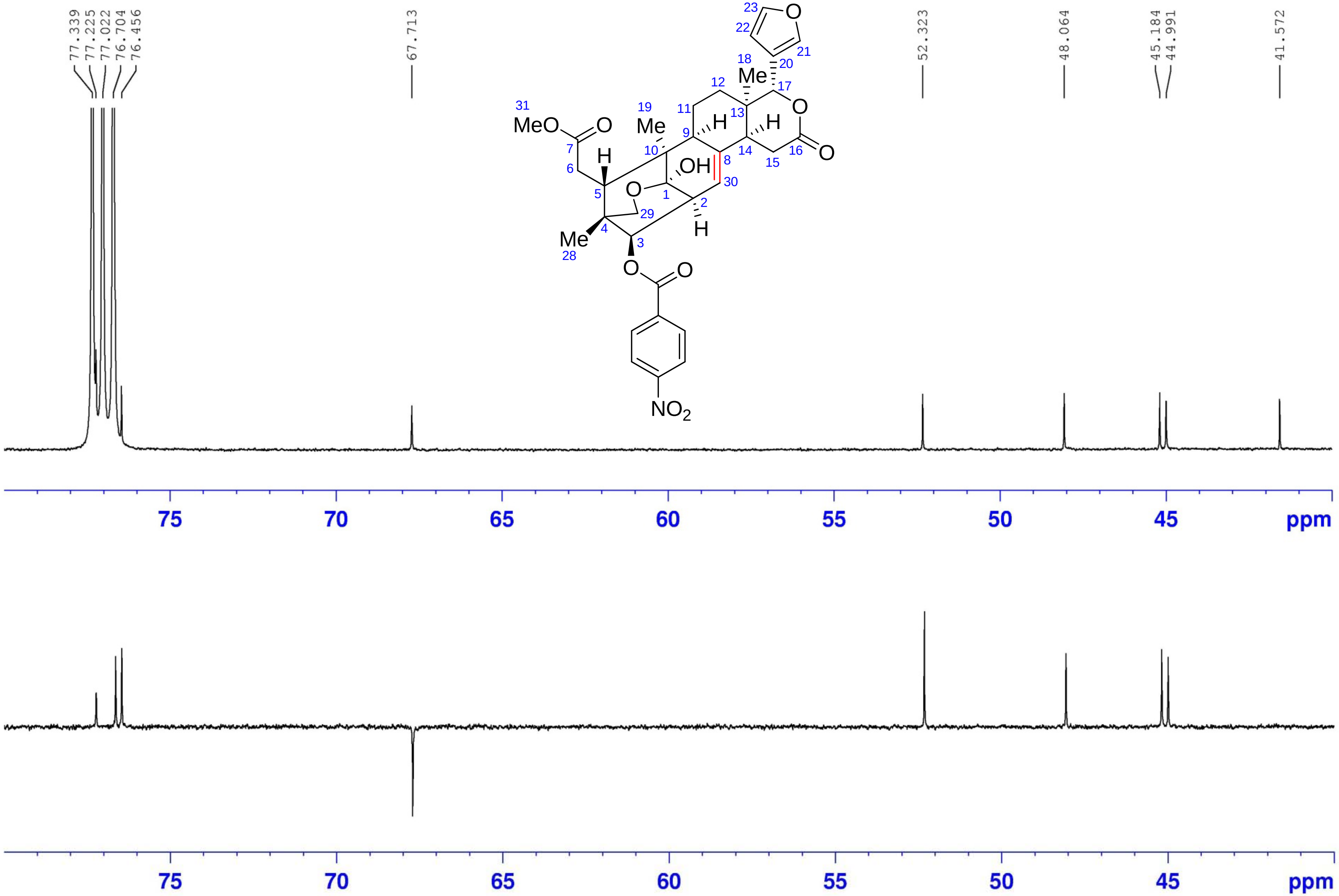

DEPT 135 spectrum of **8d** in $CDCl_3$

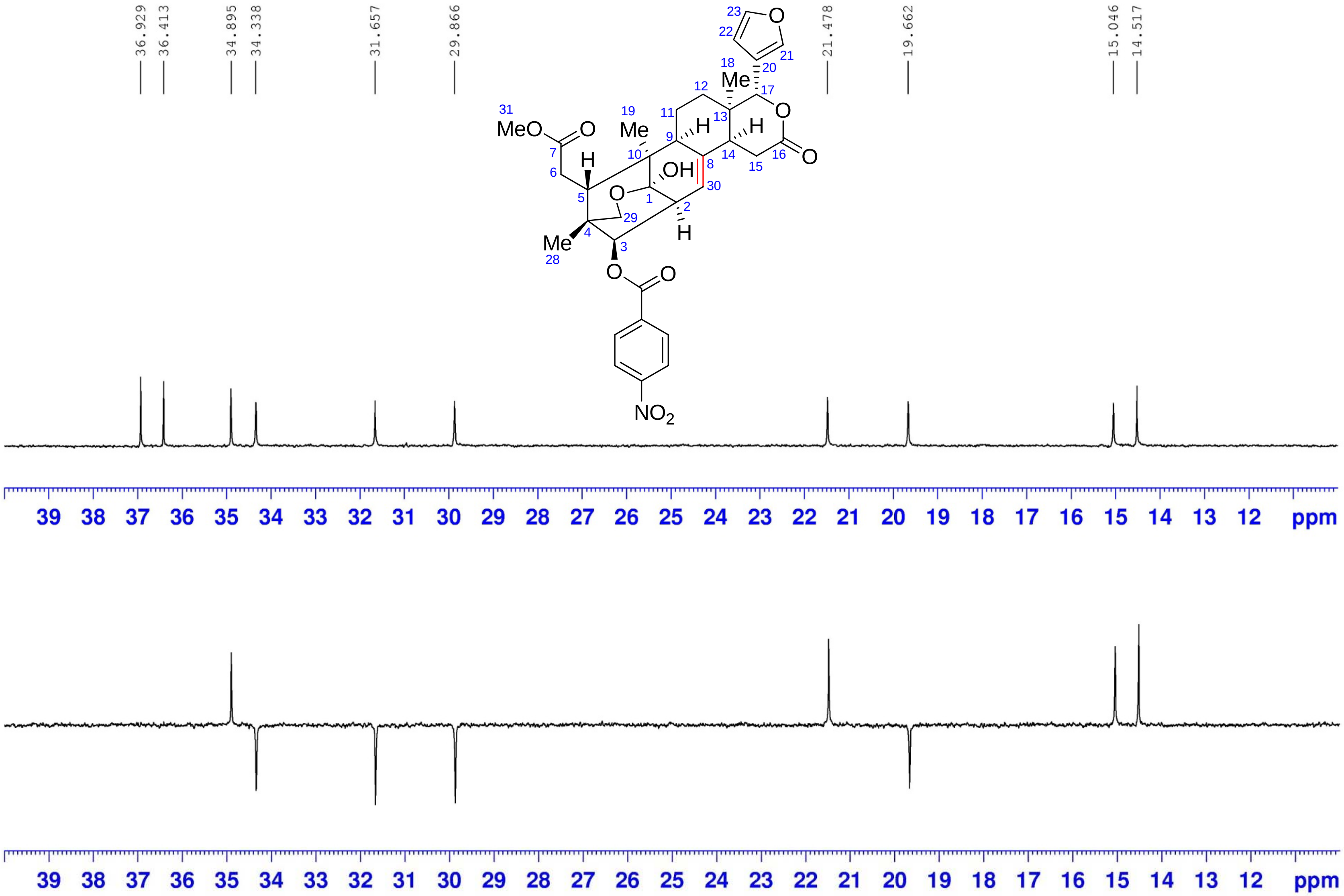

HR-ESIMS for compound **8e**

Generic Display Report

Analysis Info

Analysis Name	D:\Data\MS\data\201809\liwanshan_WSD-70_pos_16_01_5577.d	Acquisition Date	9/30/2018 6:12:23 PM
Method	LC_Direct Infusion_pos_100-1000mz.m	Operator	SCSIO
Sample Name	liwanshan_WSD-70_pos	Instrument	maXis
Comment			

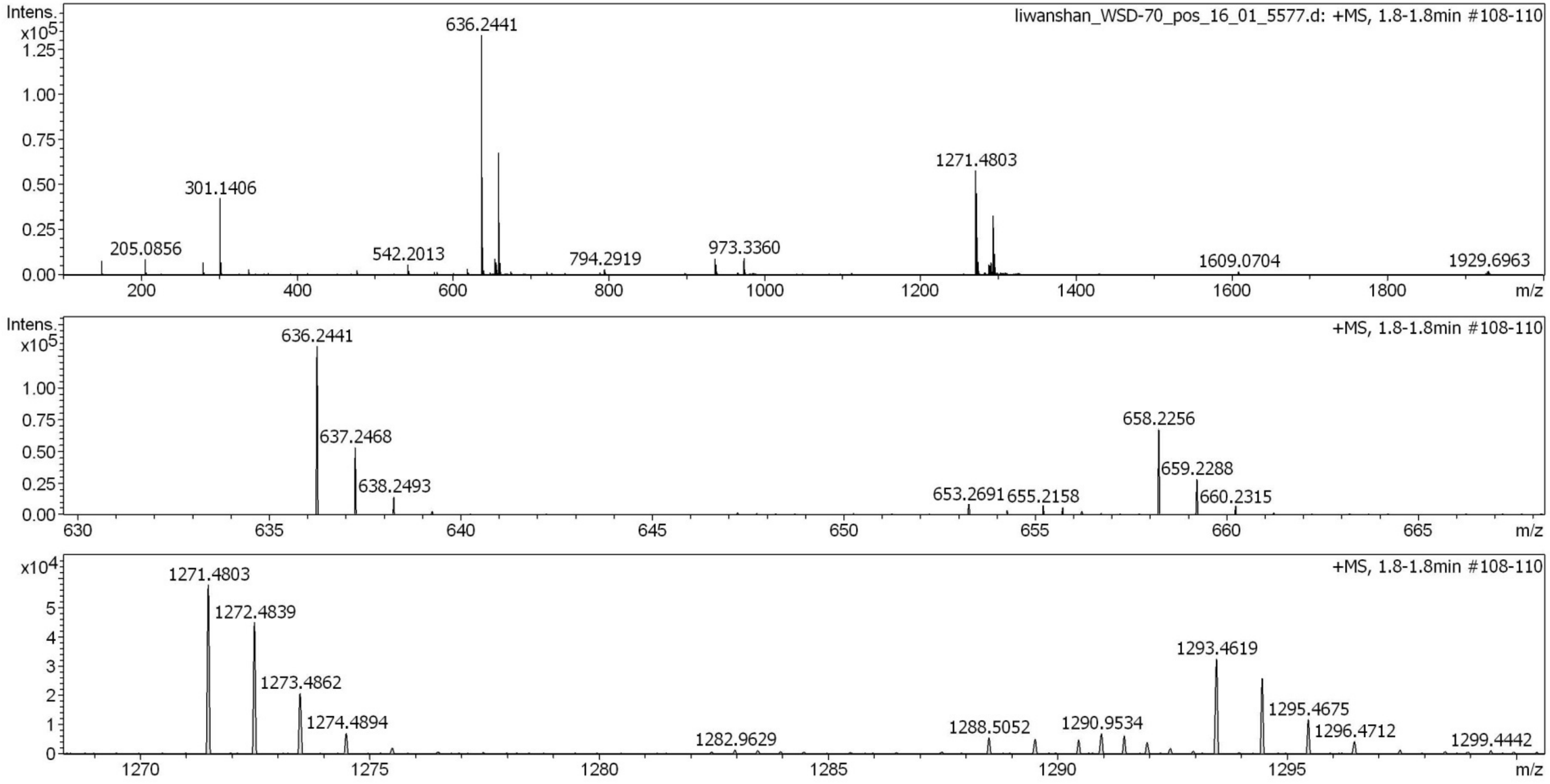

Bruker Compass DataAnalysis 4.1 printed: 9/30/2018 6:27:48 PM by: SCSIO Page 1 of 1

HR-ESIMS for compound **8e**

Mass Spectrum SmartFormula Report

Analysis Info

Analysis Name	D:\Data\MS\data\201809\liwanshan_WSD-70_pos_16_01_5577.d	Acquisition Date	9/30/2018 6:12:23 PM
Method	LC_Direct Infusion_pos_100-1000mz.m	Operator	SCSIO
Sample Name	liwanshan_WSD-70_pos	Instrument	maXis 255552.00029
Comment			

Acquisition Parameter

Source Type	ESI	Ion Polarity	Positive	Set Nebulizer	0.4 Bar
Focus	Active	Set Capillary	4500 V	Set Dry Heater	180 °C
Scan Begin	100 m/z	Set End Plate Offset	-500 V	Set Dry Gas	4.0 l/min
Scan End	2000 m/z	Set Charging Voltage	0 V	Set Divert Valve	Waste
		Set Corona	0 nA	Set APCI Heater	0 °C

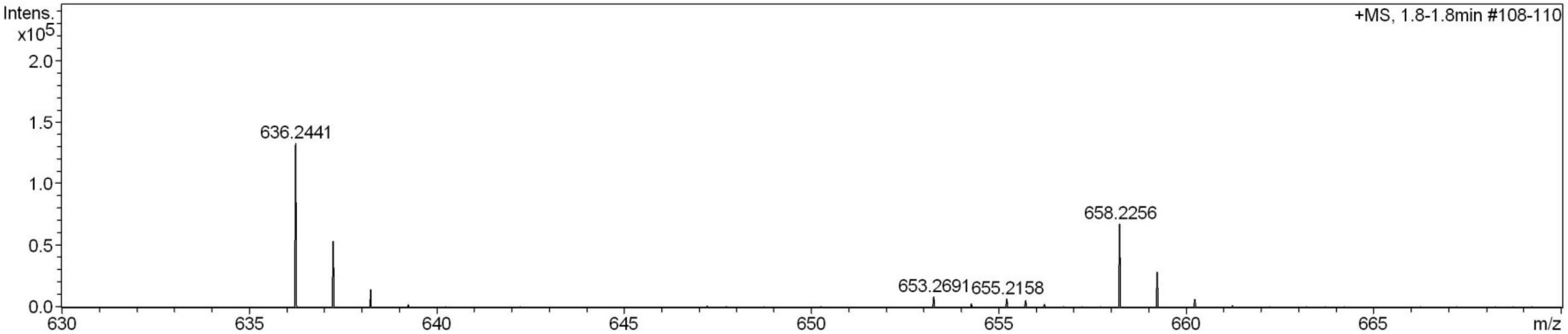

Meas. m/z	#	Ion Formula	Score	m/z	err [ppm]	err [mDa]	mSigma	rdb	e⁻ Conf	N-Rule
636.2441	1	C34H38NO11	100.00	636.2439	0.3	0.2	16.1	16.5	even	ok
658.2256	1	C34H37NNaO11	100.00	658.2259	0.5	0.3	22.4	16.5	even	ok
1271.4803	1	C68H75N2O22	100.00	1271.4806	-0.2	-0.3	15.4	32.5	even	ok
1293.4619	1	C68H74N2NaO22	100.00	1293.4625	-0.5	-0.7	23.2	32.5	even	ok

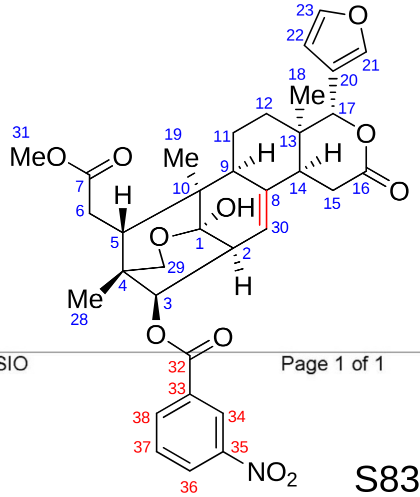

^1H NMR (400 MHz) spectrum of **8e** in $CDCl_3$

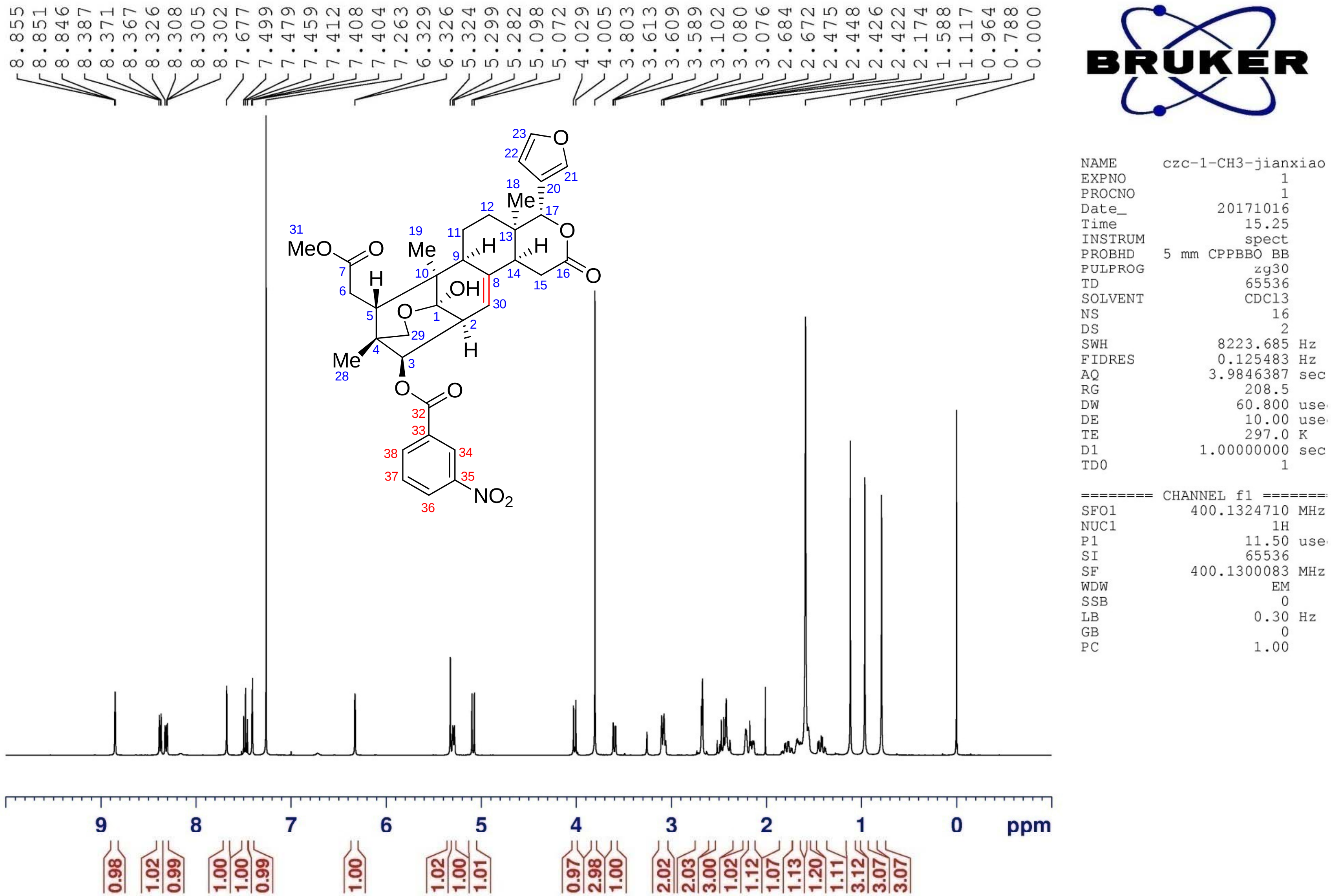

^1H NMR (400 MHz) spectrum of **8e** in $CDCl_3$

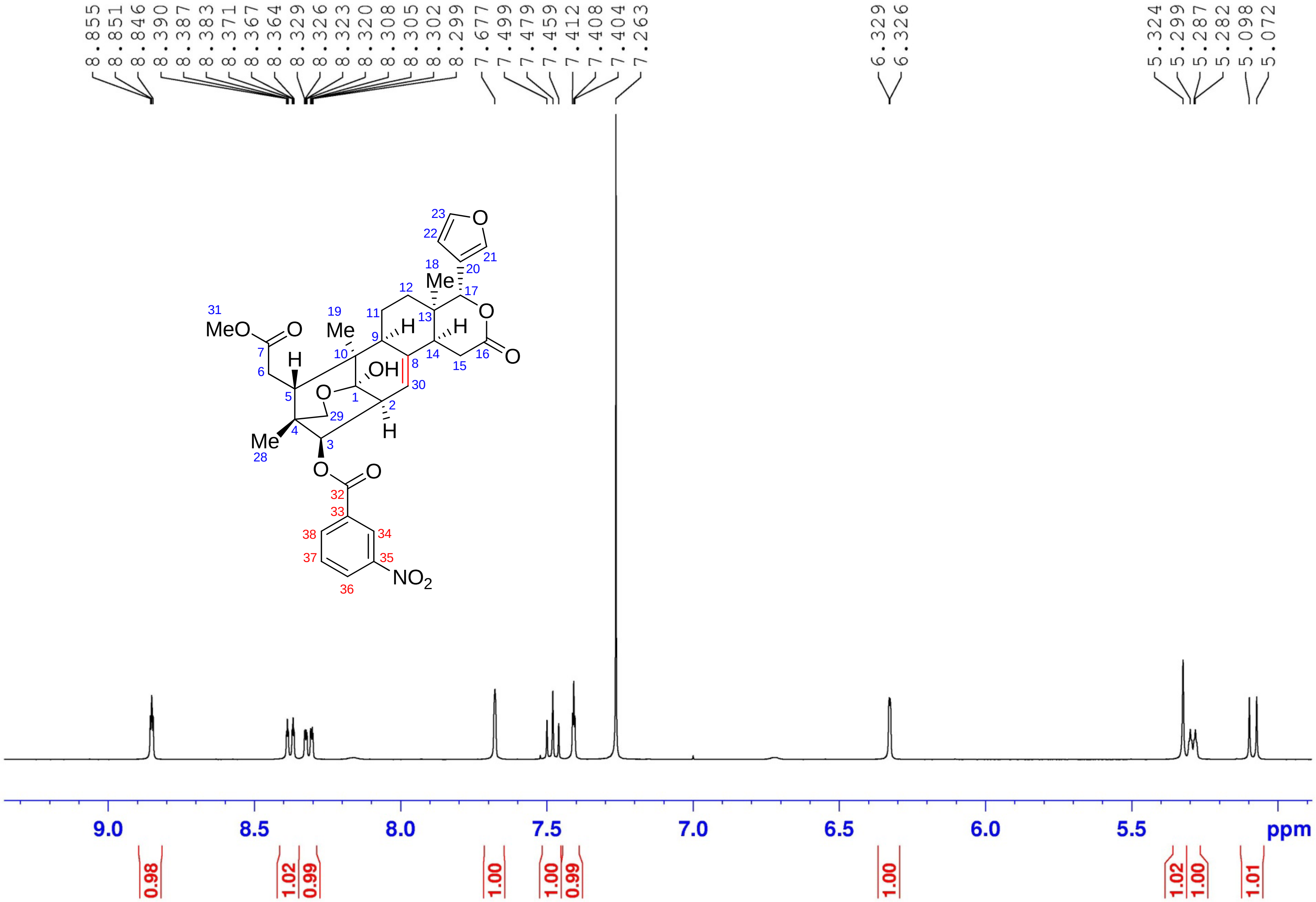

^1H NMR (400 MHz) spectrum of **8e** in $CDCl_3$

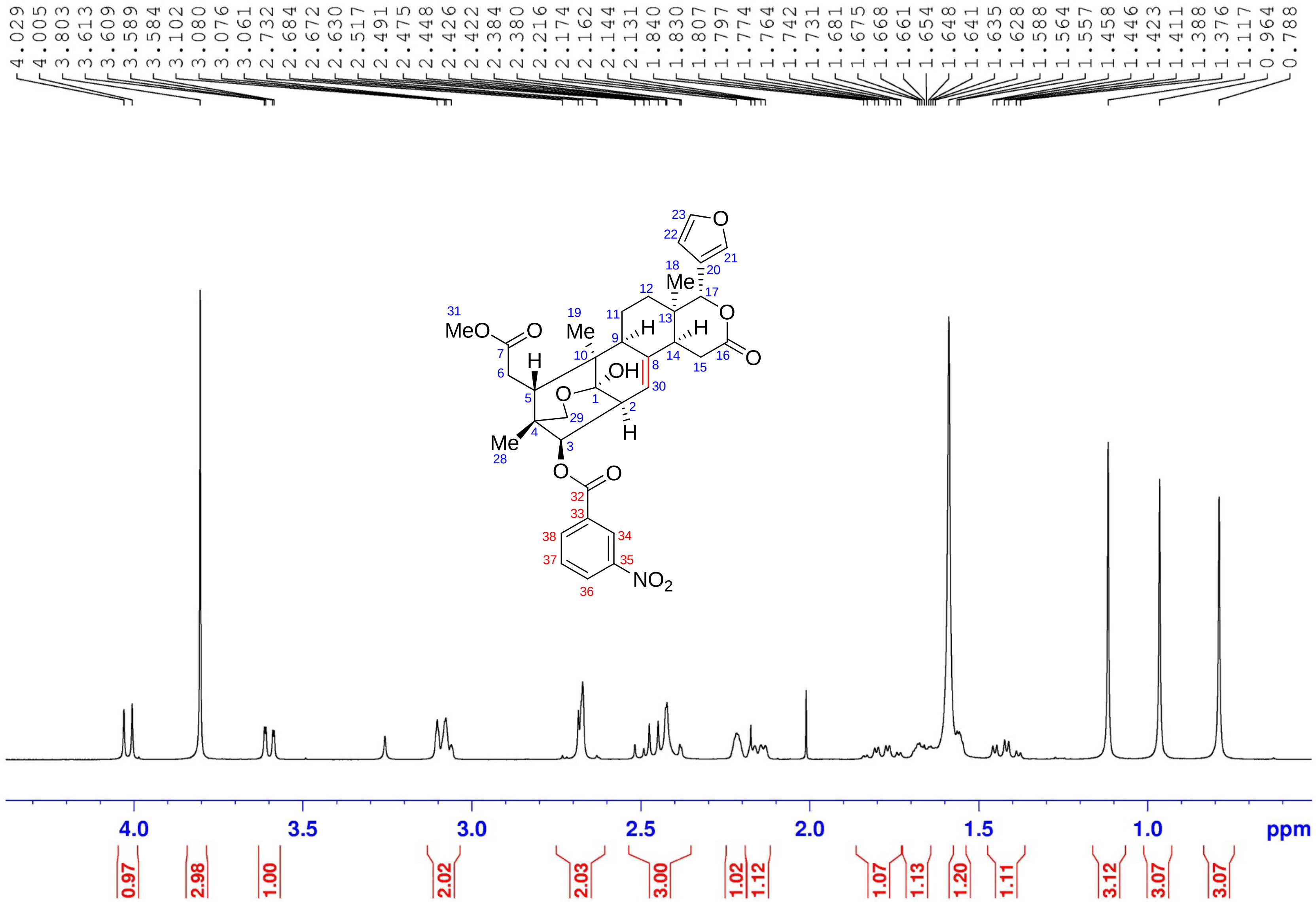

^{13}C NMR (100 MHz) spectrum of **8e** in $CDCl_3$

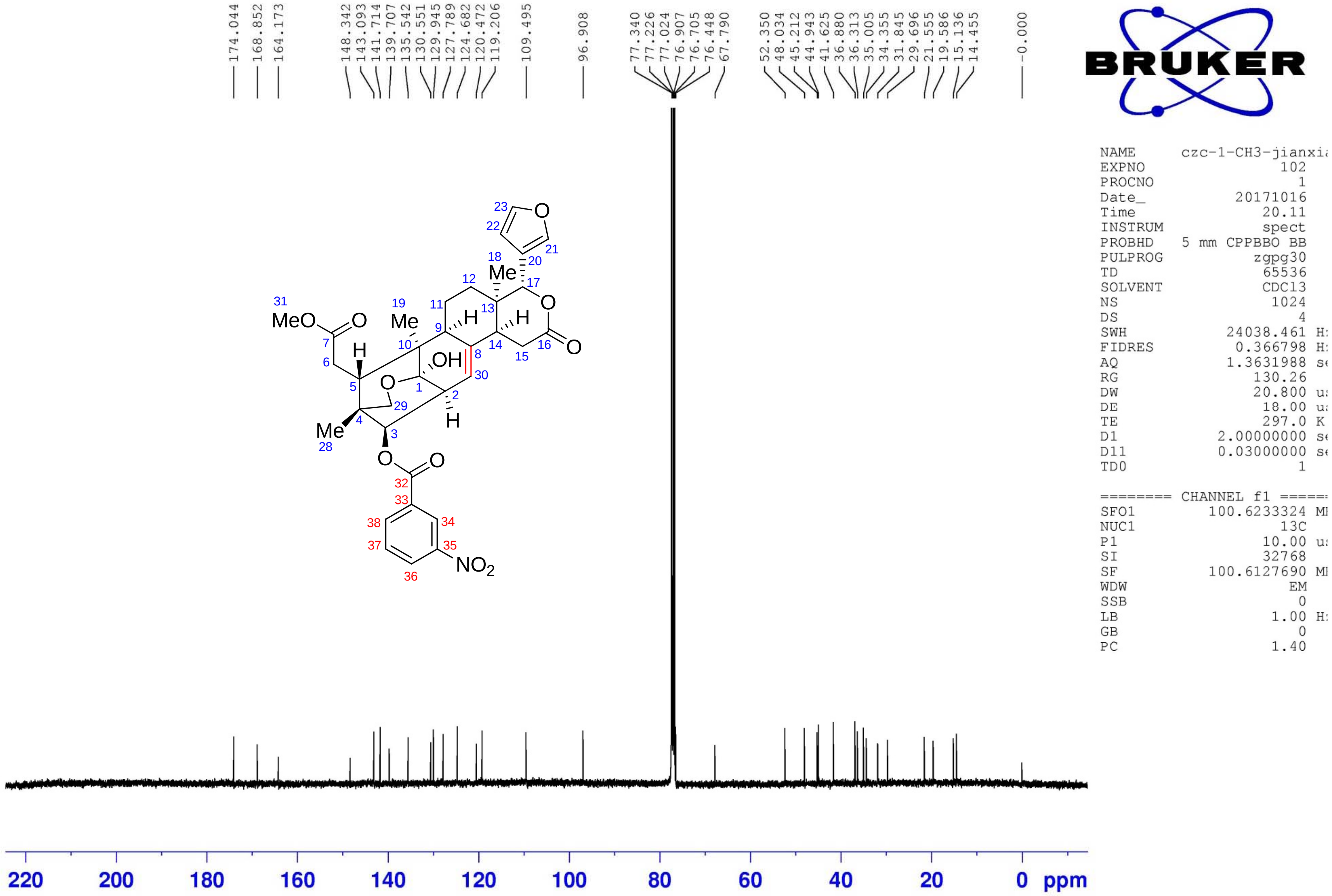


```
NAME       czc-1-CH3-jianxi
EXPNO                   102
PROCNO                    1
Date_              20171016
Time                  20.11
INSTRUM               spect
PROBHD      5 mm CPPBBO BB
PULPROG             zgpg30
TD                    65536
SOLVENT               CDCl3
NS                     1024
DS                        4
SWH               24038.461 H
FIDRES             0.366798 H
AQ                1.3631988 s
RG                   130.26
DW                   20.800 u
DE                    18.00 u
TE                    297.0 K
D1               2.00000000 s
D11              0.03000000 s
TD0                       1

======== CHANNEL f1 ======
SFO1            100.6233324 M
NUC1                    13C
P1                    10.00 u
SI                    32768
SF              100.6127690 M
WDW                      EM
SSB                       0
LB                     1.00 H
GB                        0
PC                     1.40
```

^{13}C NMR (100 MHz) spectrum of **8e** in $CDCl_3$

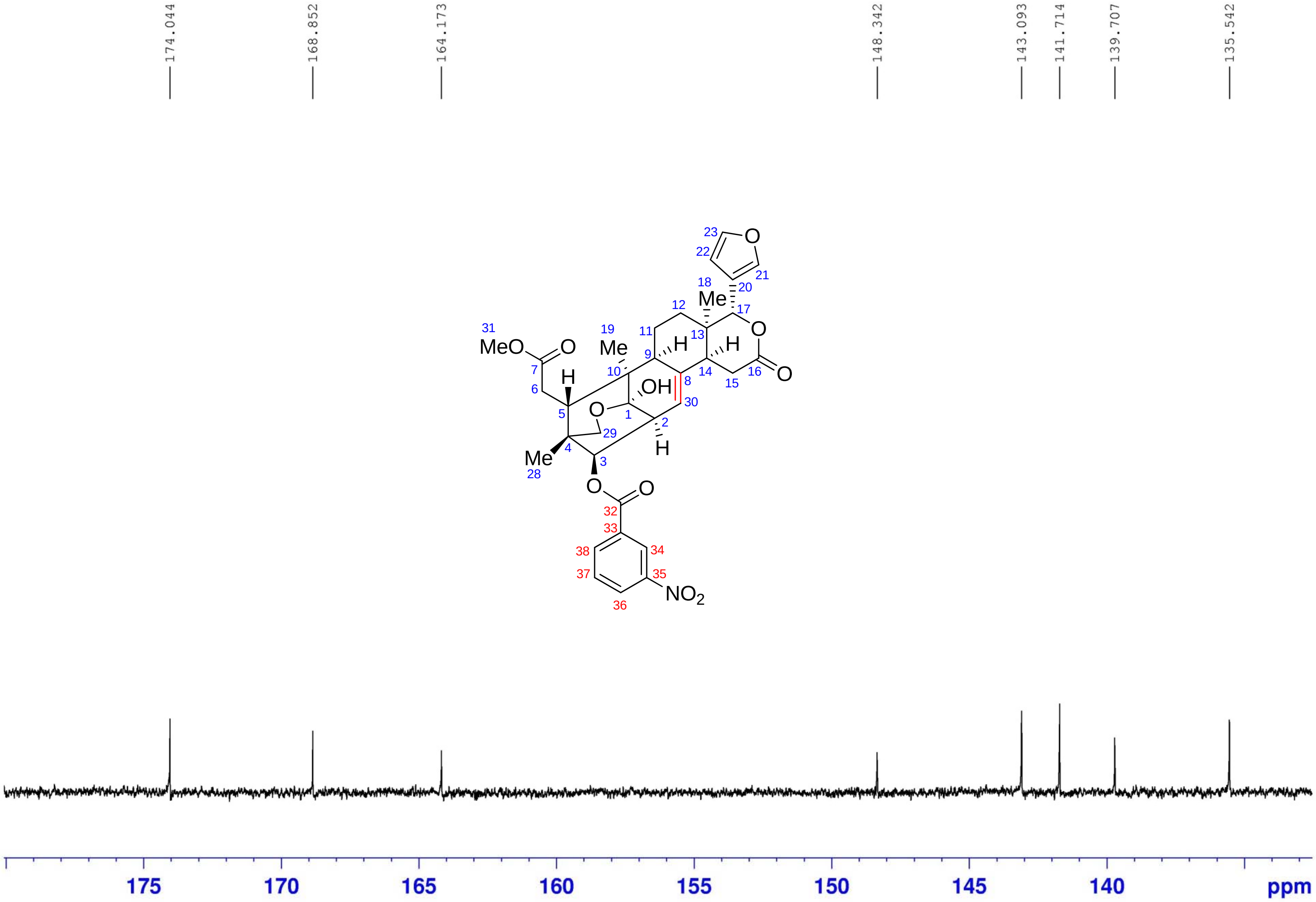

^{13}C NMR (100 MHz) spectrum of **8e** in $CDCl_3$

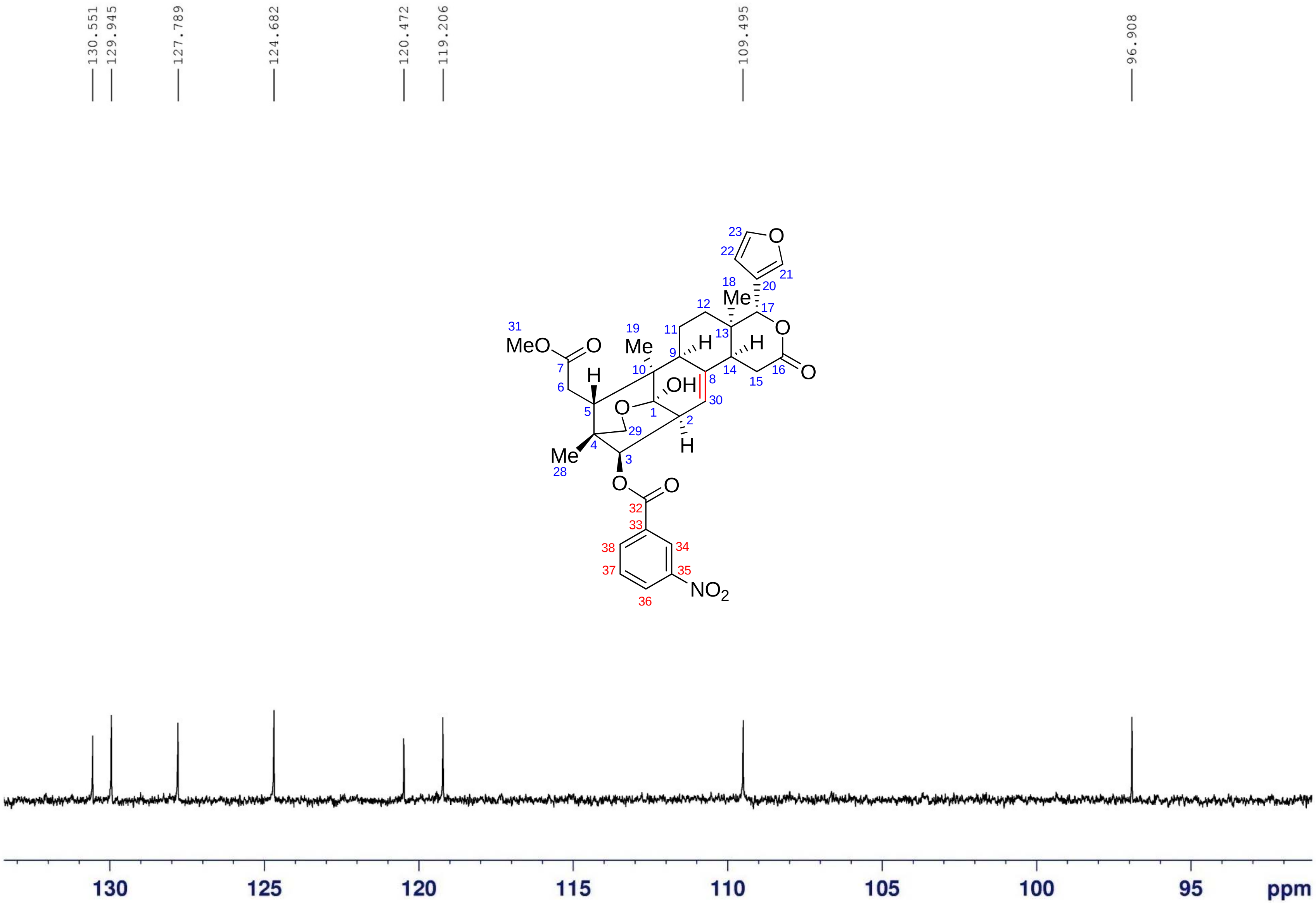

^{13}C NMR (100 MHz) spectrum of **8e** in $CDCl_3$

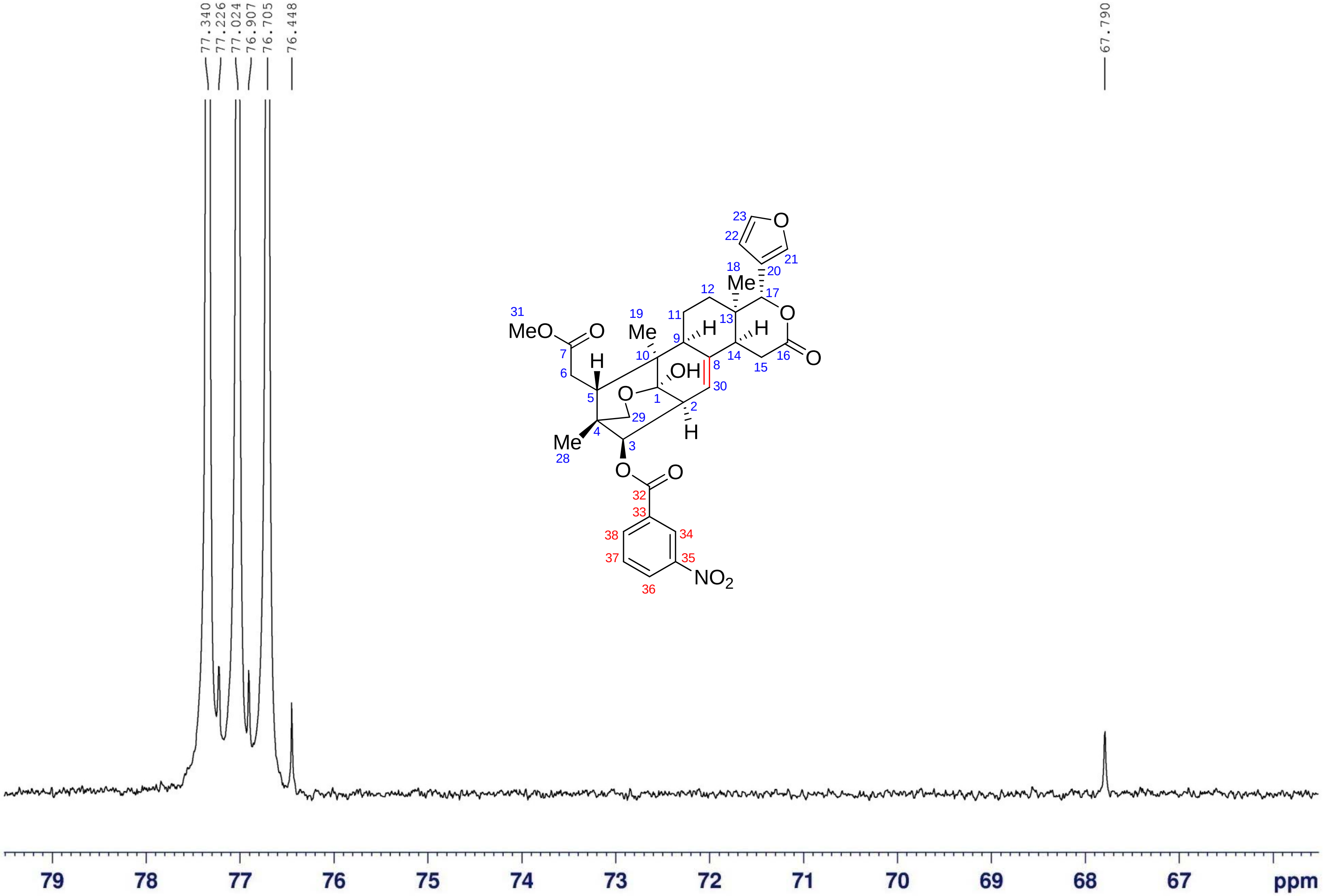

^{13}C NMR (100 MHz) spectrum of **8e** in $CDCl_3$

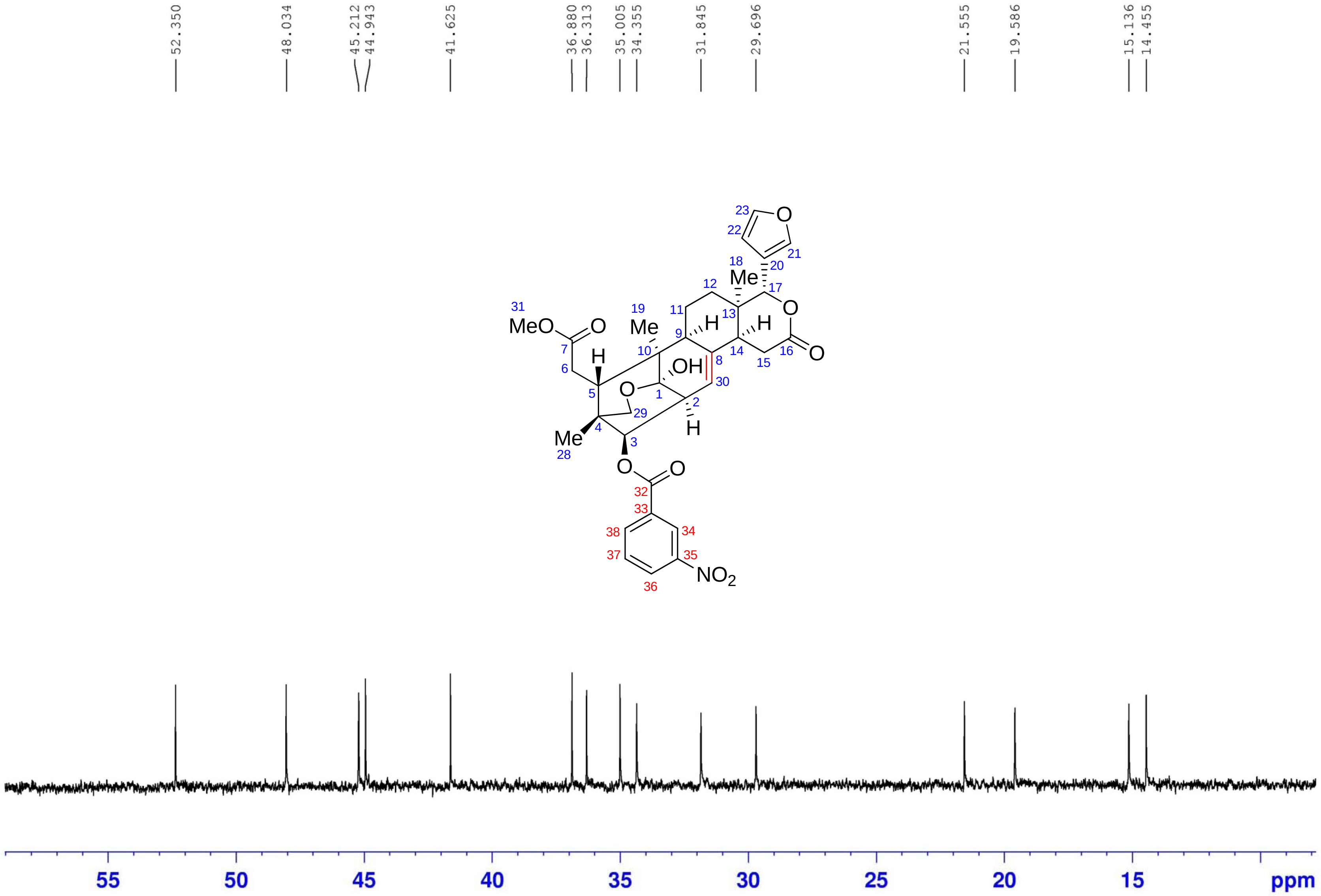

DEPT 135 spectrum of **8e** in $CDCl_3$

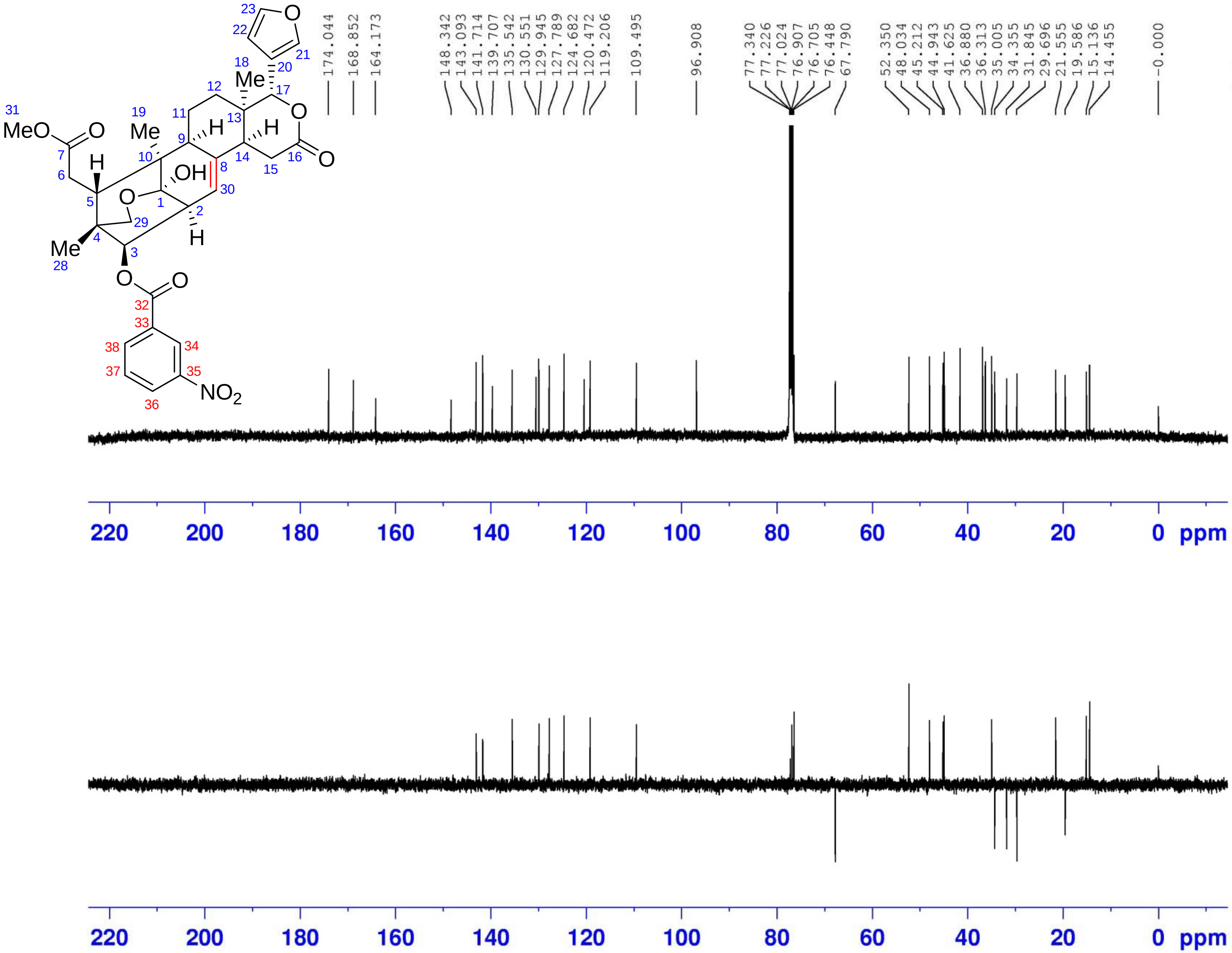

NAME	czc-1-CH3-jianx
EXPNO	3
PROCNO	1
Date_	20171016
Time	19.06
INSTRUM	spect
PROBHD	5 mm CPPBBO BB
PULPROG	deptsp135
TD	65536
SOLVENT	CDCl3
NS	300
DS	4
SWH	24038.461
FIDRES	0.366798
AQ	1.3631988
RG	73.92
DW	20.800
DE	18.00
TE	297.0
CNST2	145.0000000
D1	2.00000000
D2	0.00344828
D12	0.00002000
TD0	1

======== CHANNEL f1 ========

SFO1	100.6233324
NUC1	13C
P1	10.00
P13	2000.00
SI	32768
SF	100.6127692
WDW	EM
SSB	0
LB	1.00
GB	0
PC	1.40

DEPT 135 spectrum of **8e** in $CDCl_3$

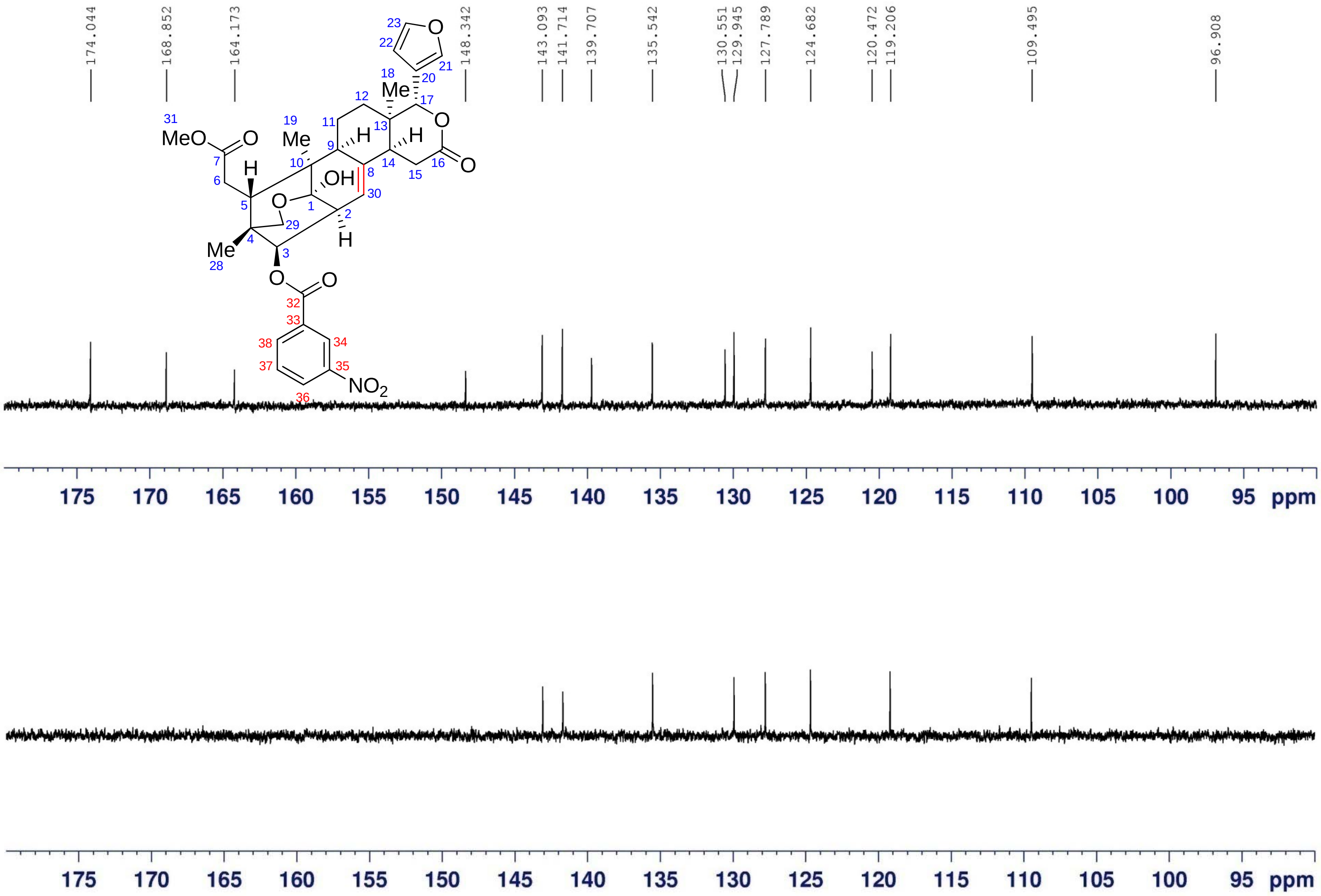

DEPT 135 spectrum of **8e** in $CDCl_3$

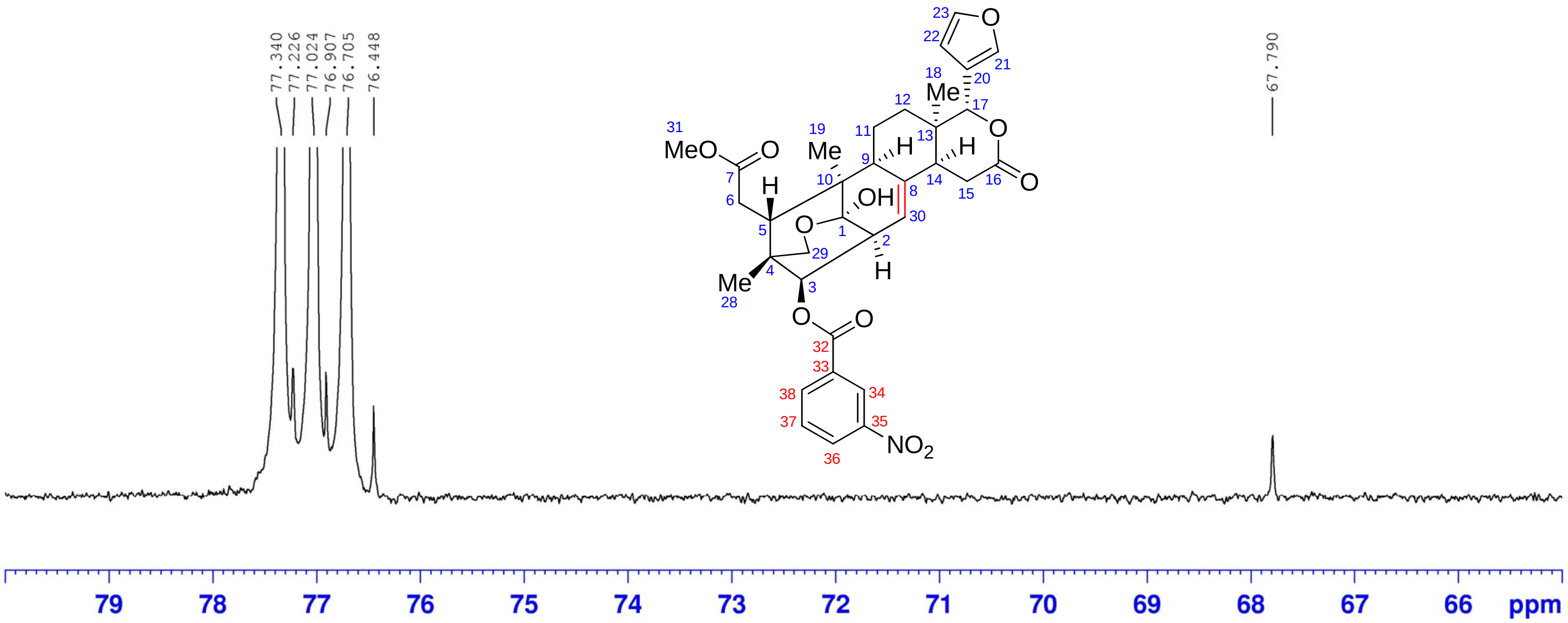

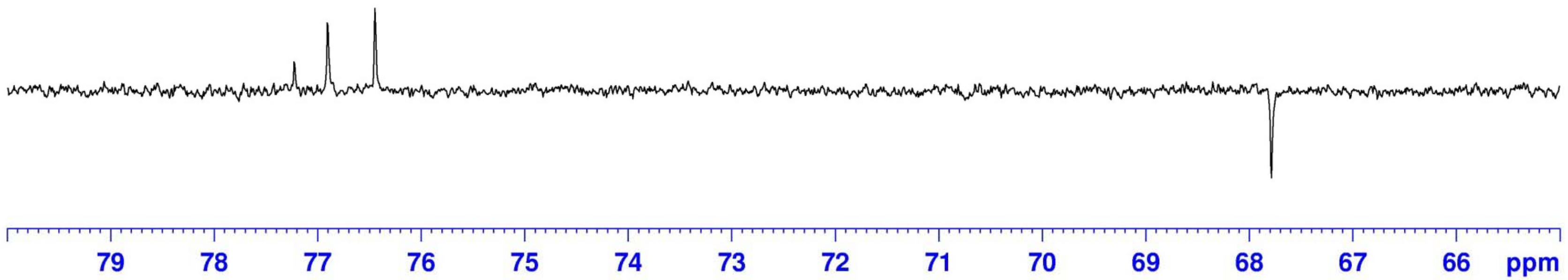

DEPT 135 spectrum of **8e** in $CDCl_3$

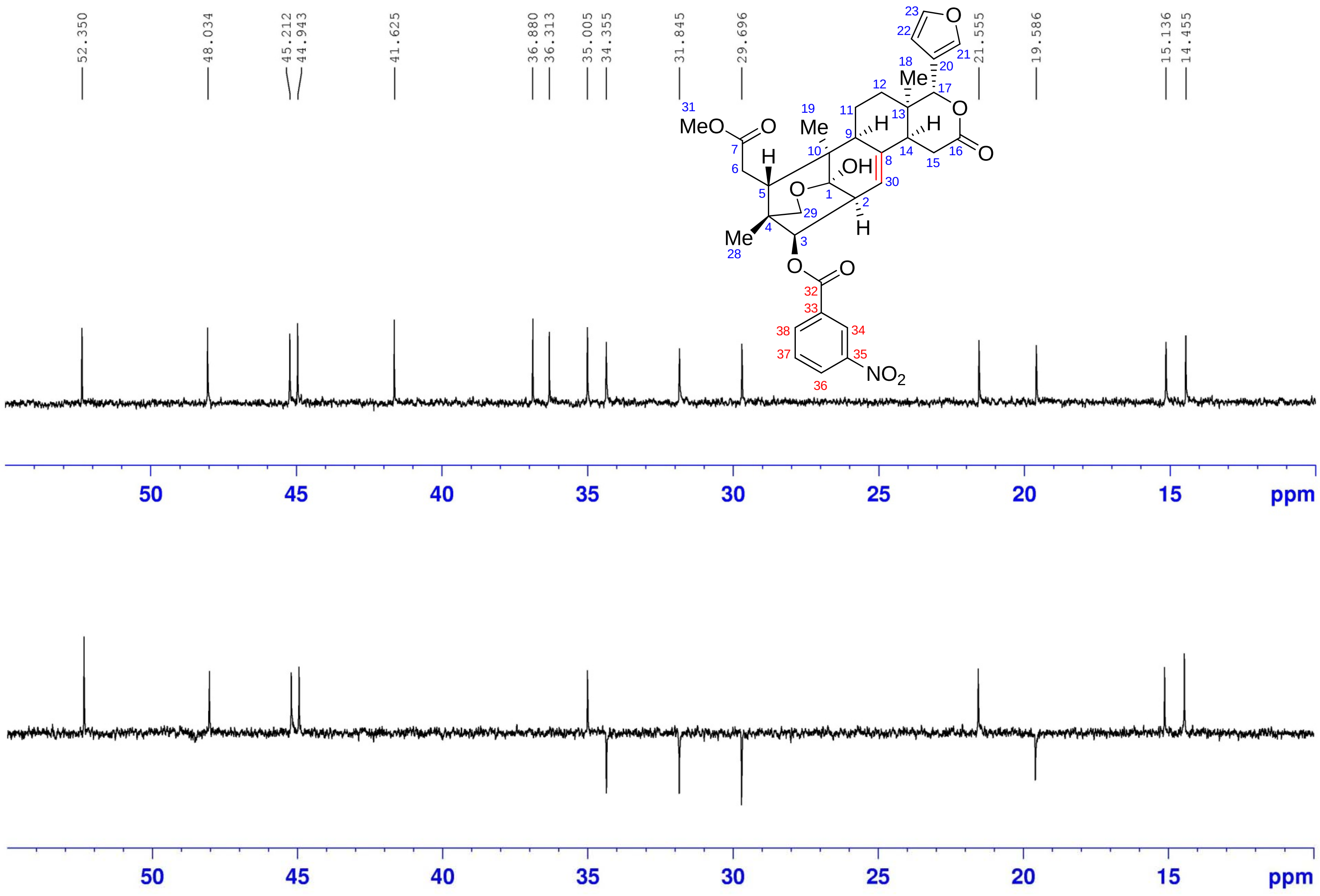

HR-ESIMS for compound **8f**

Generic Display Report

Analysis Info

Analysis Name	D:\Data\MS\data\201809\liwanshan_WSD-71_pos_17_01_5578.d	Acquisition Date	9/30/2018 6:15:53 PM
Method	LC_Direct Infusion_pos_100-1000mz.m	Operator	SCSIO
Sample Name	liwanshan_WSD-71_pos	Instrument	maXis
Comment			

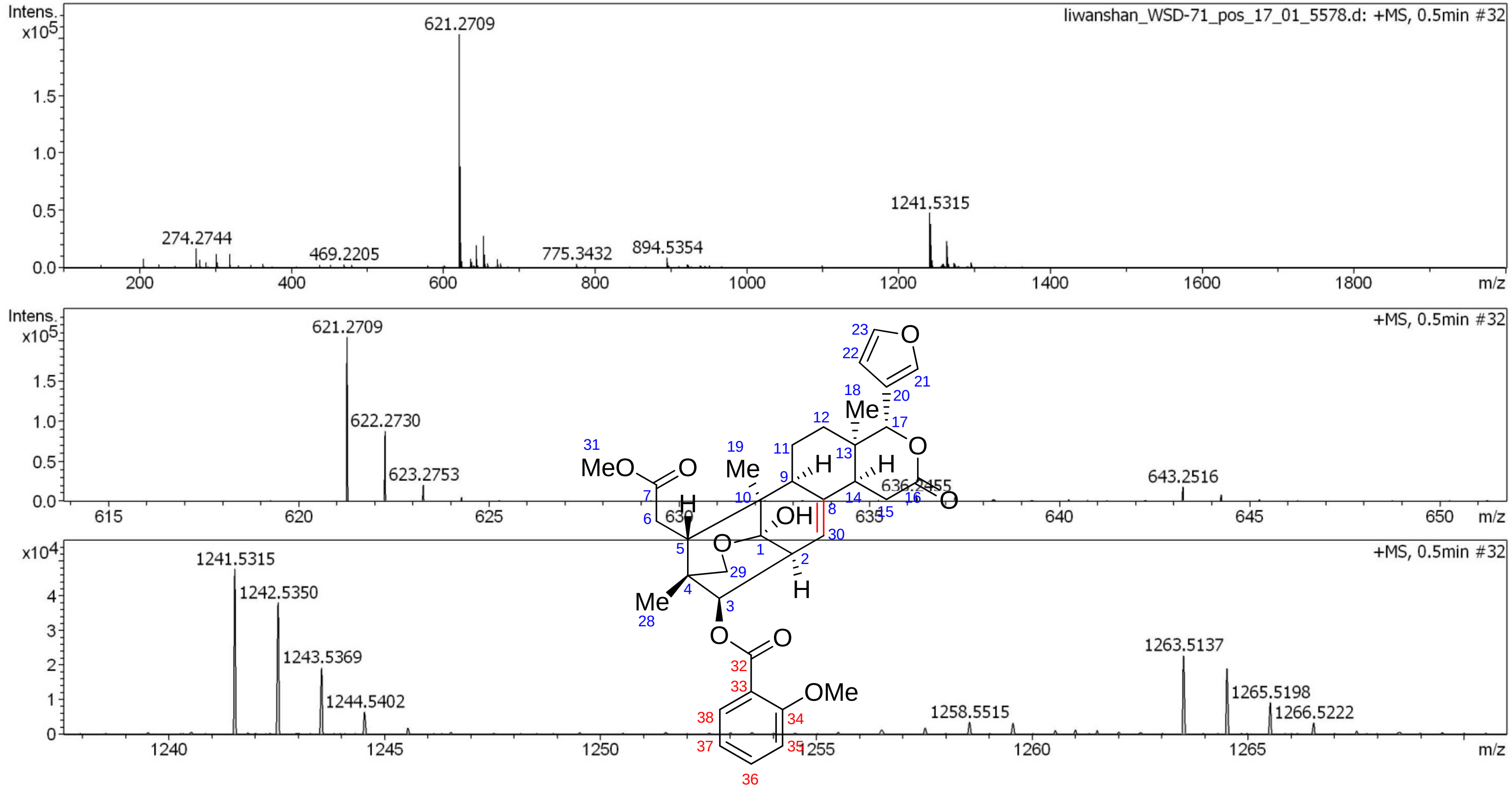

HR-ESIMS for compound **8f**

Mass Spectrum SmartFormula Report

Analysis Info

Analysis Name	D:\Data\MS\data\201809\liwanshan_WSD-71_pos_17_01_5578.d	Acquisition Date	9/30/2018 6:15:53 PM
Method	LC_Direct Infusion_pos_100-1000mz.m	Operator	SCSIO
Sample Name	liwanshan_WSD-71_pos	Instrument	maXis 255552.00029
Comment			

Acquisition Parameter

Source Type	ESI	Ion Polarity	Positive	Set Nebulizer	0.4 Bar
Focus	Active	Set Capillary	4500 V	Set Dry Heater	180 °C
Scan Begin	100 m/z	Set End Plate Offset	-500 V	Set Dry Gas	4.0 l/min
Scan End	2000 m/z	Set Charging Voltage	0 V	Set Divert Valve	Waste
		Set Corona	0 nA	Set APCI Heater	0 °C

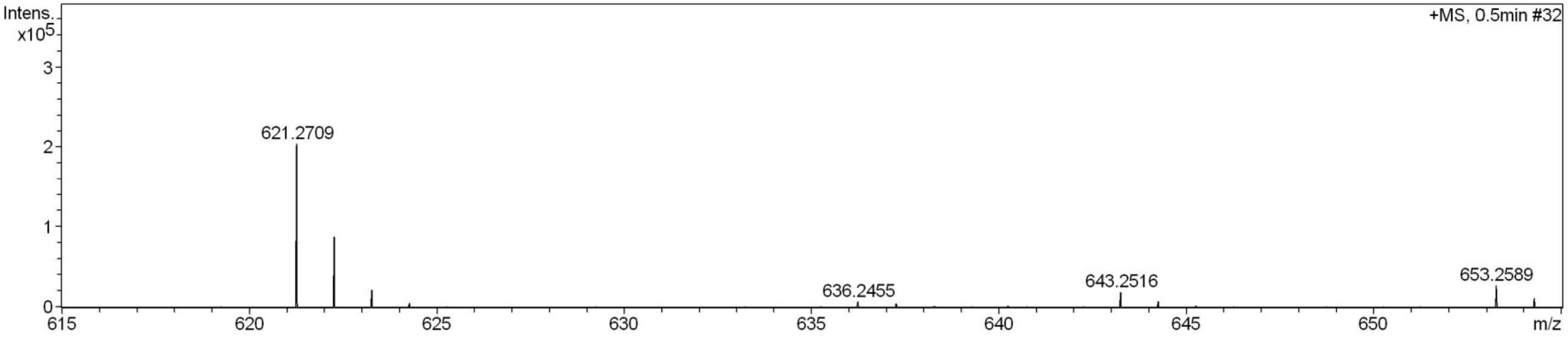

Meas. m/z	#	Ion Formula	Score	m/z	err [ppm]	err [mDa]	mSigma	rdb	e⁻ Conf	N-Rule
621.2709	1	C35H41O10	100.00	621.2694	2.3	1.4	24.5	15.5	even	ok
643.2516	1	C35H40NaO10	100.00	643.2514	0.4	0.2	8.1	15.5	even	ok
1241.5315	1	C70H81O20	100.00	1241.5316	0.0	0.1	32.1	30.5	even	ok
1263.5137	1	C70H80NaO20	100.00	1263.5135	0.2	0.2	39.2	30.5	even	ok

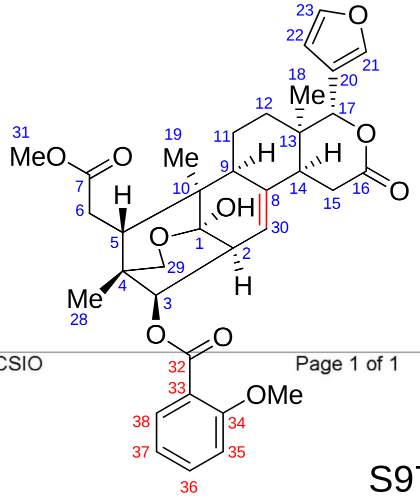

^{1}H NMR (400 MHz) spectrum of **8f** in $CDCl_3$

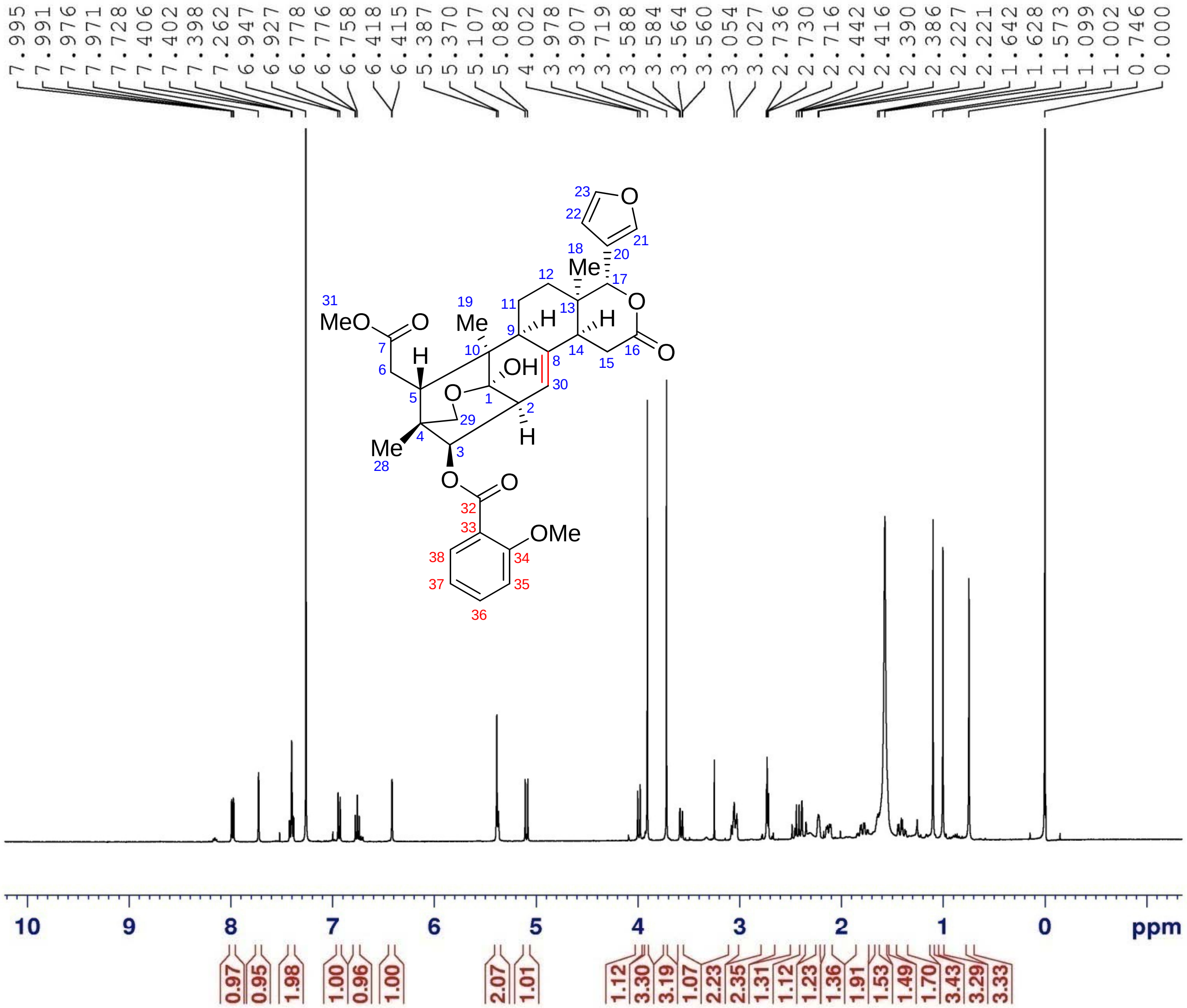

NAME	czc-1-CH3-LINJIAYANG
EXPNO	1
PROCNO	1
Date_	20171018
Time	12.16
INSTRUM	spect
PROBHD	5 mm CPPBBO BB
PULPROG	zg30
TD	65536
SOLVENT	CDCl3
NS	16
DS	2
SWH	8223.685 Hz
FIDRES	0.125483 Hz
AQ	3.9846387 sec
RG	208.5
DW	60.800 usec
DE	10.00 usec
TE	297.0 K
D1	1.00000000 sec
TD0	1

======== CHANNEL f1 ========

SFO1	400.1324710 MHz
NUC1	1H
P1	11.50 usec
SI	65536
SF	400.1300089 MHz
WDW	EM
SSB	0
LB	0.30 Hz
GB	0
PC	1.00

^1H NMR (400 MHz) spectrum of **8f** in $CDCl_3$

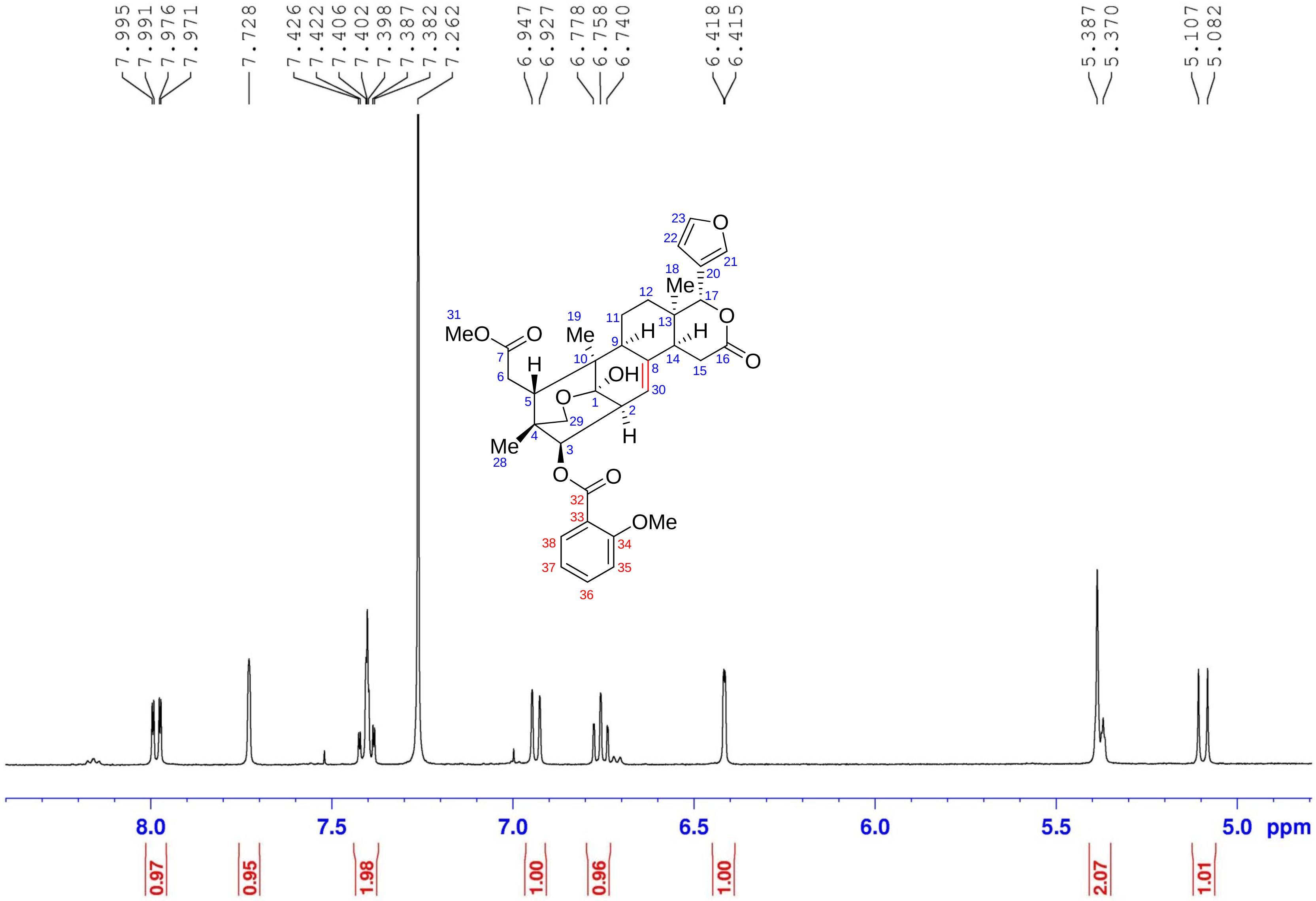

^1H NMR (400 MHz) spectrum of **8f** in $CDCl_3$

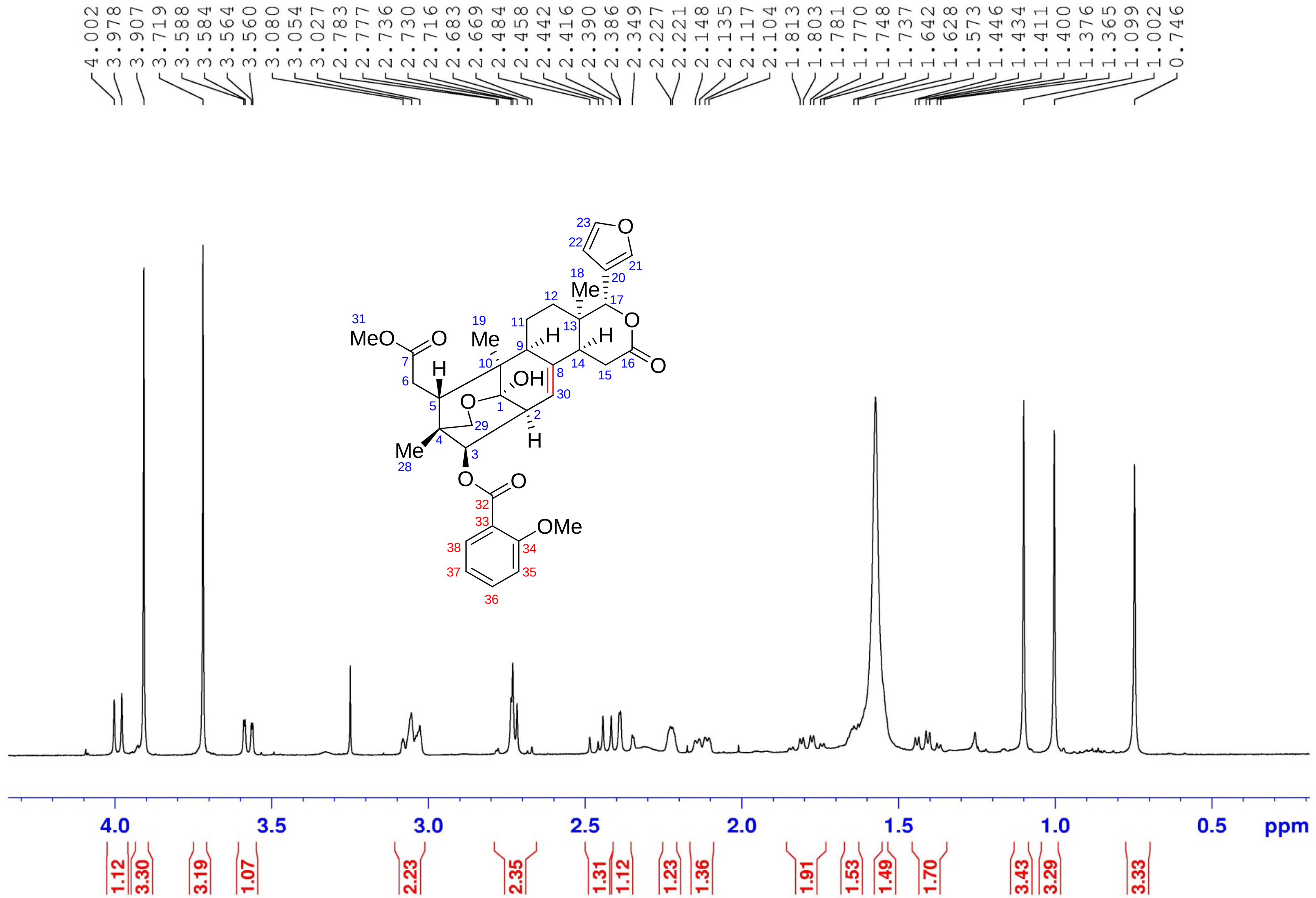

^{13}C NMR (100 MHz) spectrum of **8f** in $CDCl_3$

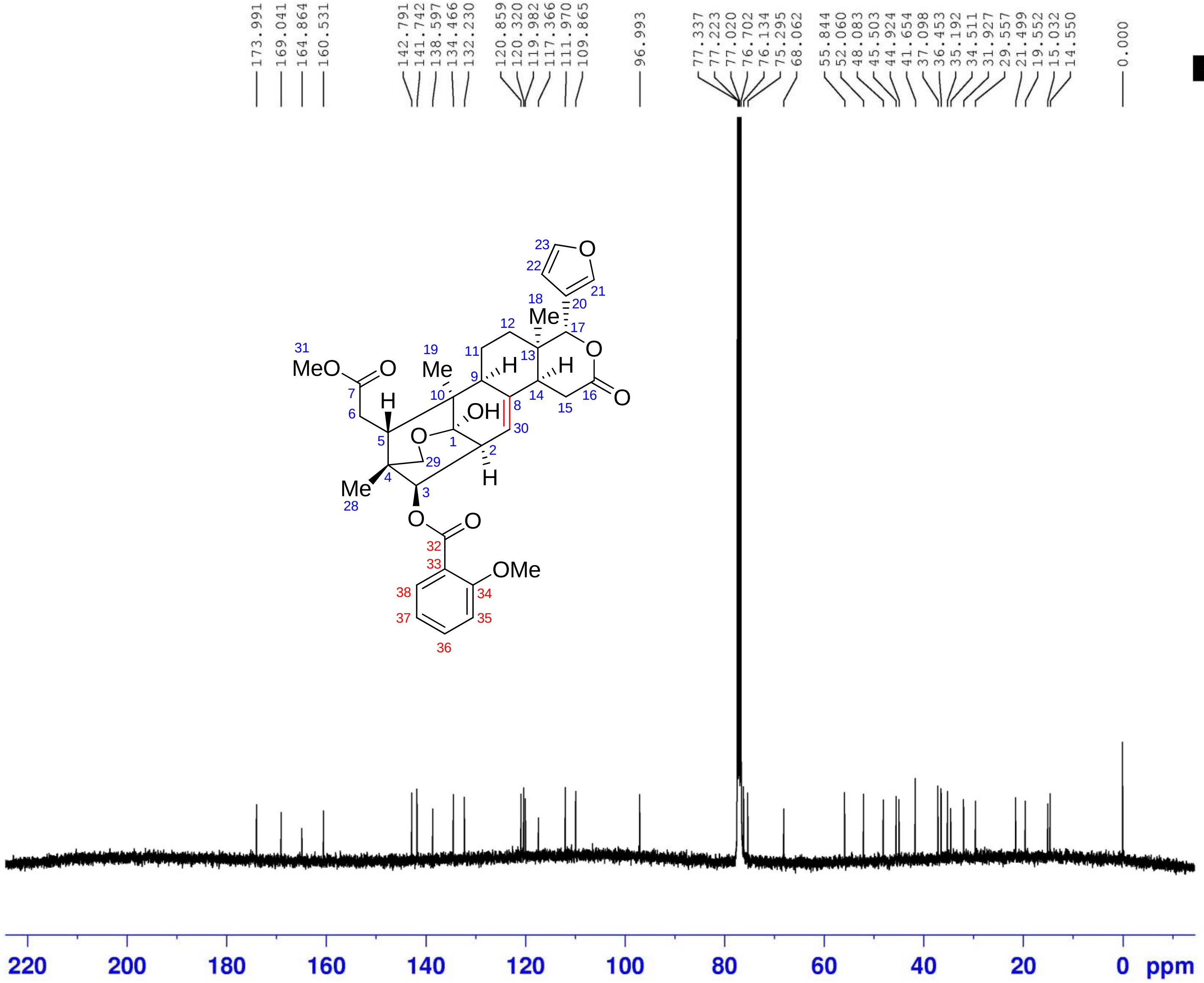


```
NAME       czc-1-CH3-LINJIAYA
EXPNO                     102
PROCNO                      1
Date_                20171021
Time                    14.09
INSTRUM                 spect
PROBHD    5 mm CPPBBO BB
PULPROG               zgpg30
TD                      65536
SOLVENT                 CDCl3
NS                       3000
DS                          4
SWH                 24038.461 Hz
FIDRES               0.366798 Hz
AQ                  1.3631988 sec
RG                     147.94
DW                     20.800 use
DE                      18.00 use
TE                      297.0 K
D1                 2.00000000 sec
D11                0.03000000 sec
TD0                         1

======== CHANNEL f1 =======
SFO1              100.6233324 MHz
NUC1                      13C
P1                      10.00 use
SI                      32768
SF                100.6127691 MHz
WDW                        EM
SSB                         0
LB                       1.00 Hz
GB                          0
PC                       1.40
```

^{13}C NMR (100 MHz) spectrum of **8f** in $CDCl_3$

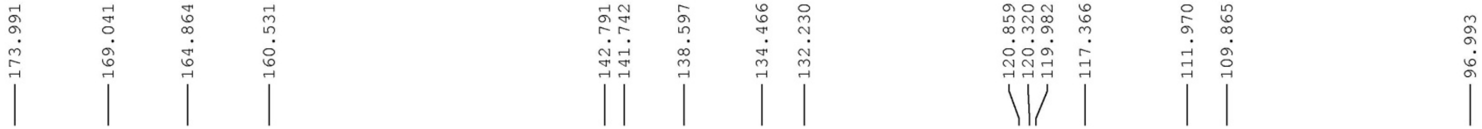

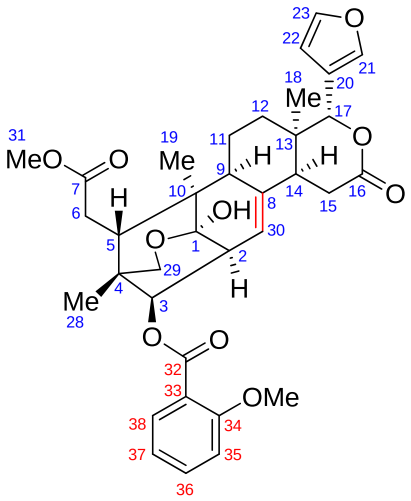

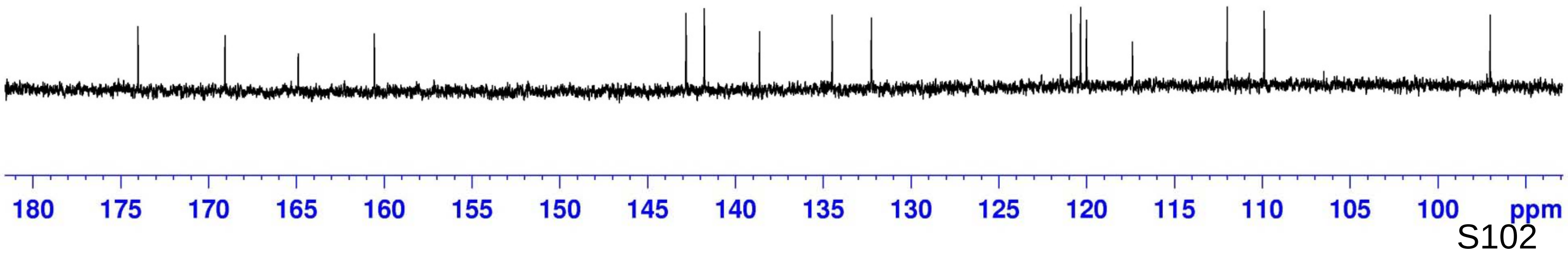

^{13}C NMR (100 MHz) spectrum of **8f** in $CDCl_3$

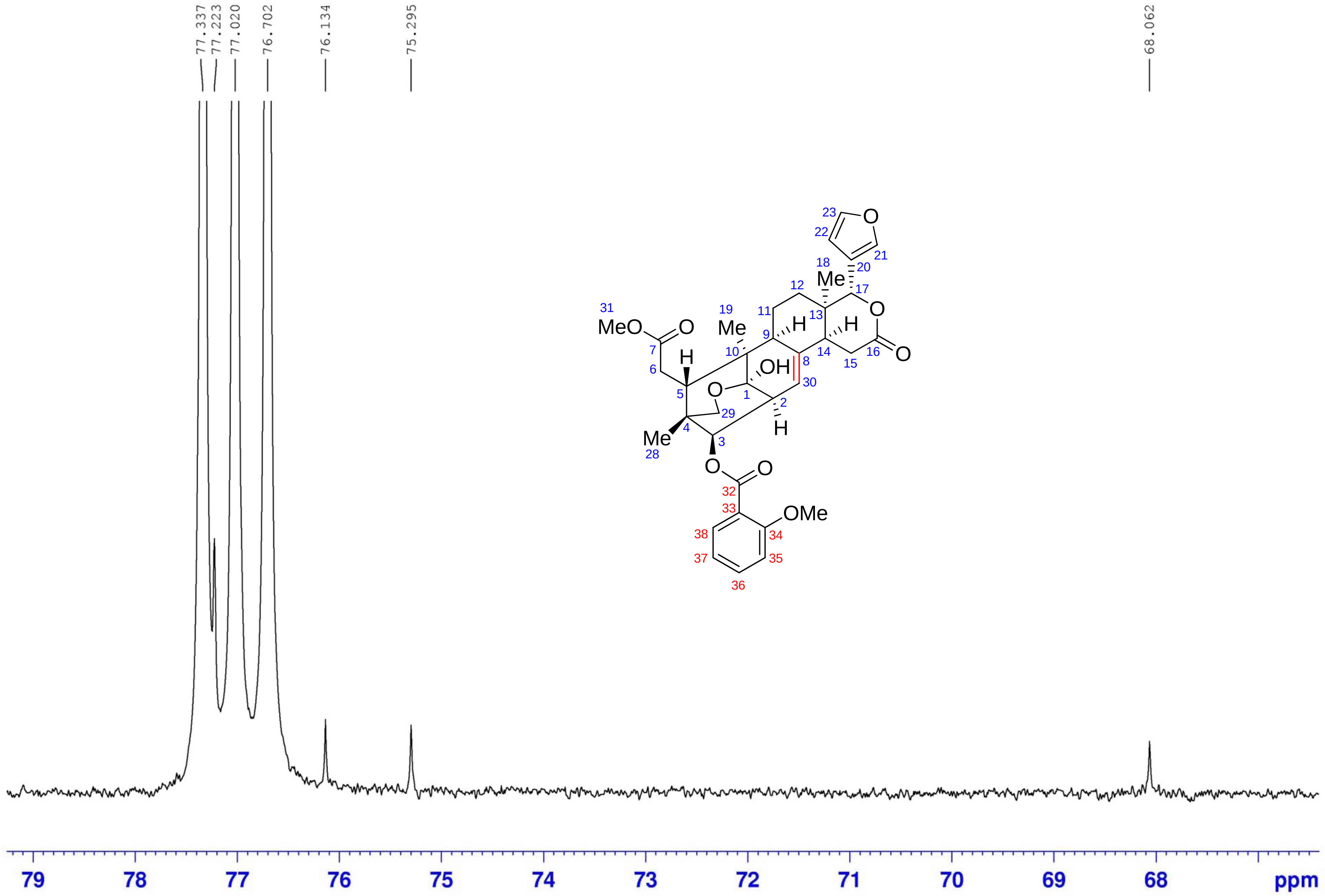

^{13}C NMR (100 MHz) spectrum of **8f** in $CDCl_3$

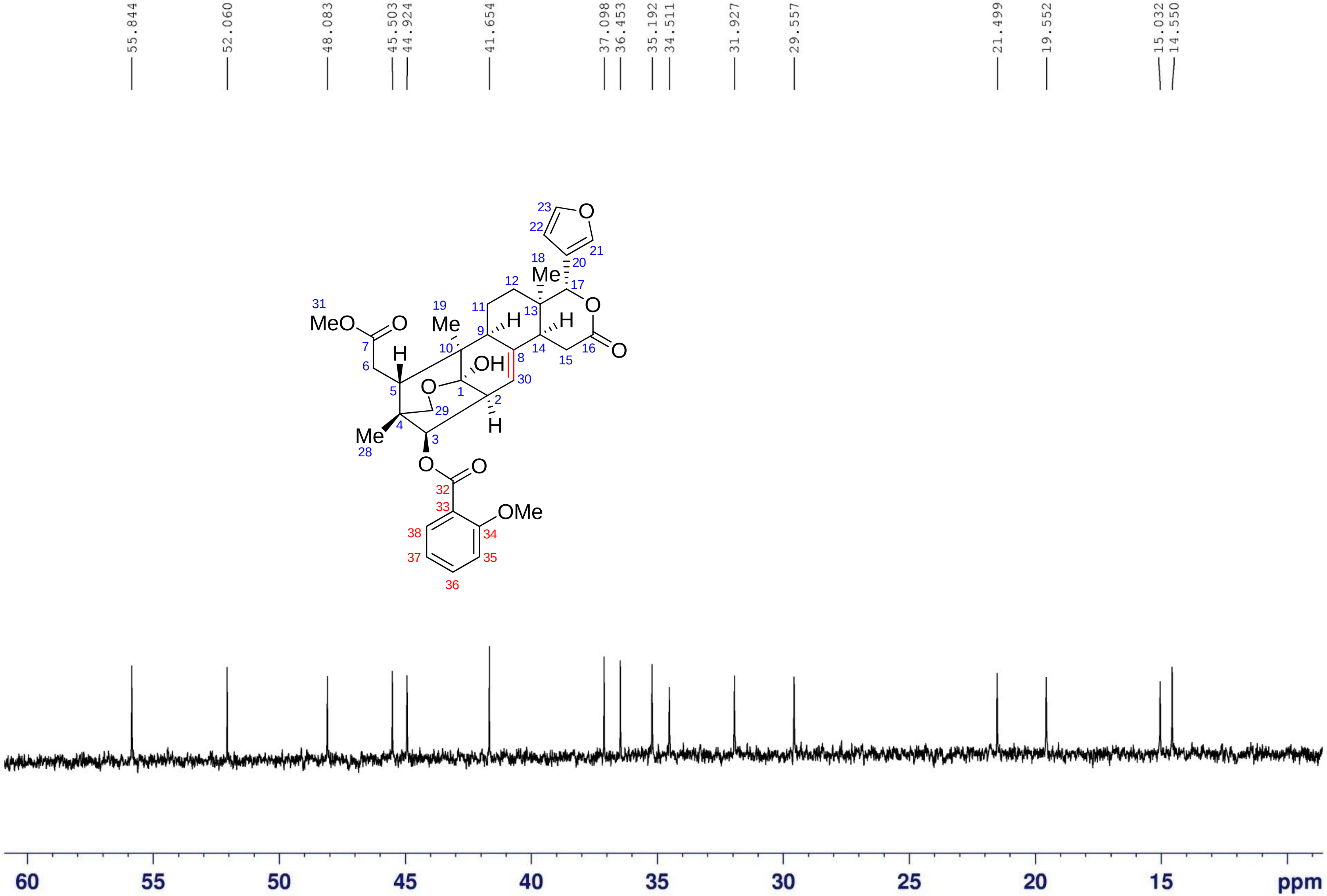

DEPT 135 spectrum of **8f** in $CDCl_3$

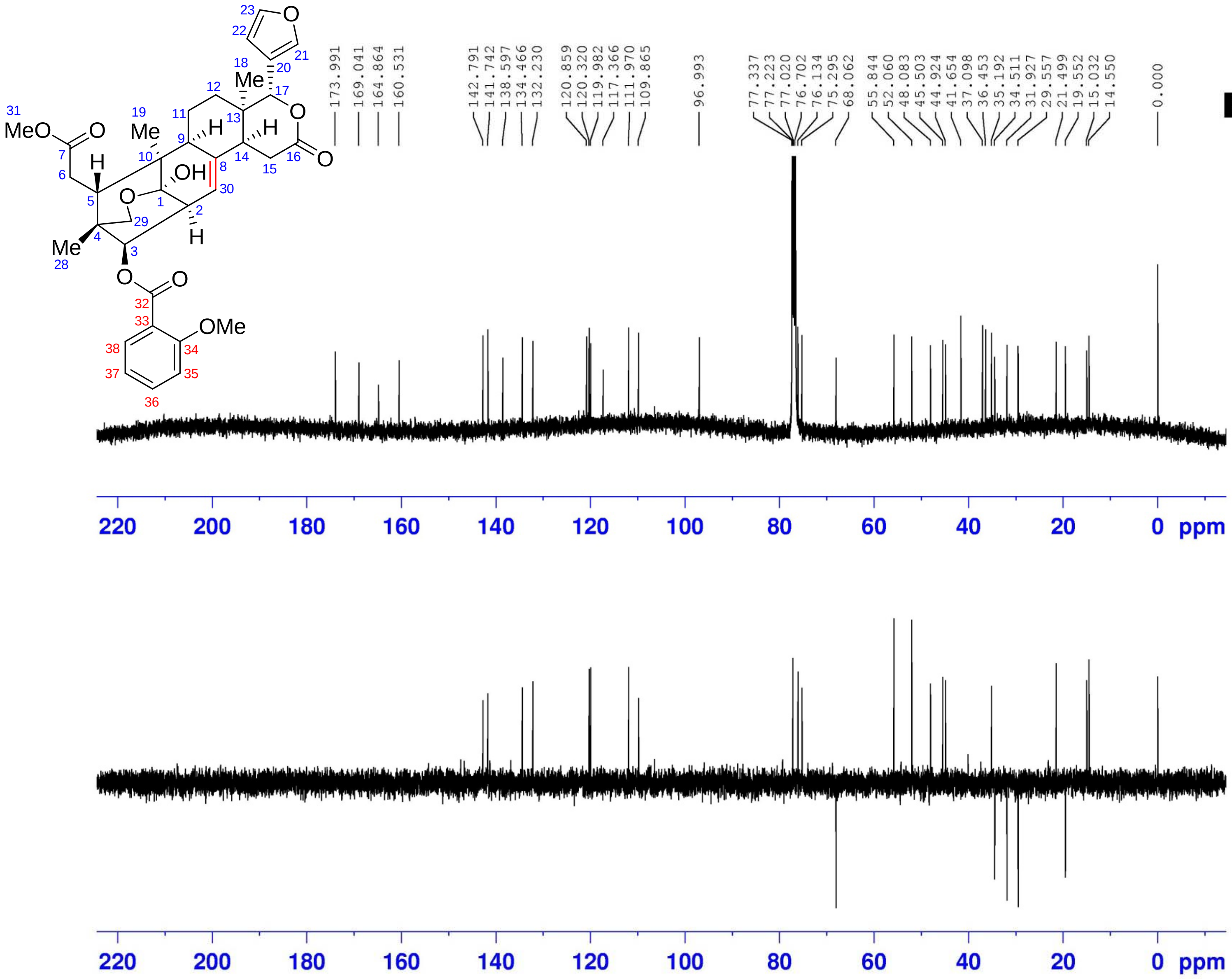

NAME	czc-1-CH3-LINJIAY.
EXPNO	103
PROCNO	1
Date_	20171021
Time	14.56
INSTRUM	spect
PROBHD	5 mm CPPBBO BB
PULPROG	deptsp135
TD	65536
SOLVENT	CDCl3
NS	800
DS	4
SWH	24038.461 Hz
FIDRES	0.366798 Hz
AQ	1.3631988 se
RG	130.26
DW	20.800 us
DE	18.00 us
TE	297.0 K
CNST2	145.0000000
D1	2.00000000 se
D2	0.00344828 se
D12	0.00002000 se
TD0	1

======== CHANNEL f1 ======

SFO1	100.6233324 MH
NUC1	13C
P1	10.00 us
P13	2000.00 us
SI	32768
SF	100.6127693 MH
WDW	EM
SSB	0
LB	1.00 Hz
GB	0
PC	1.40

DEPT 135 spectrum of **8f** in $CDCl_3$

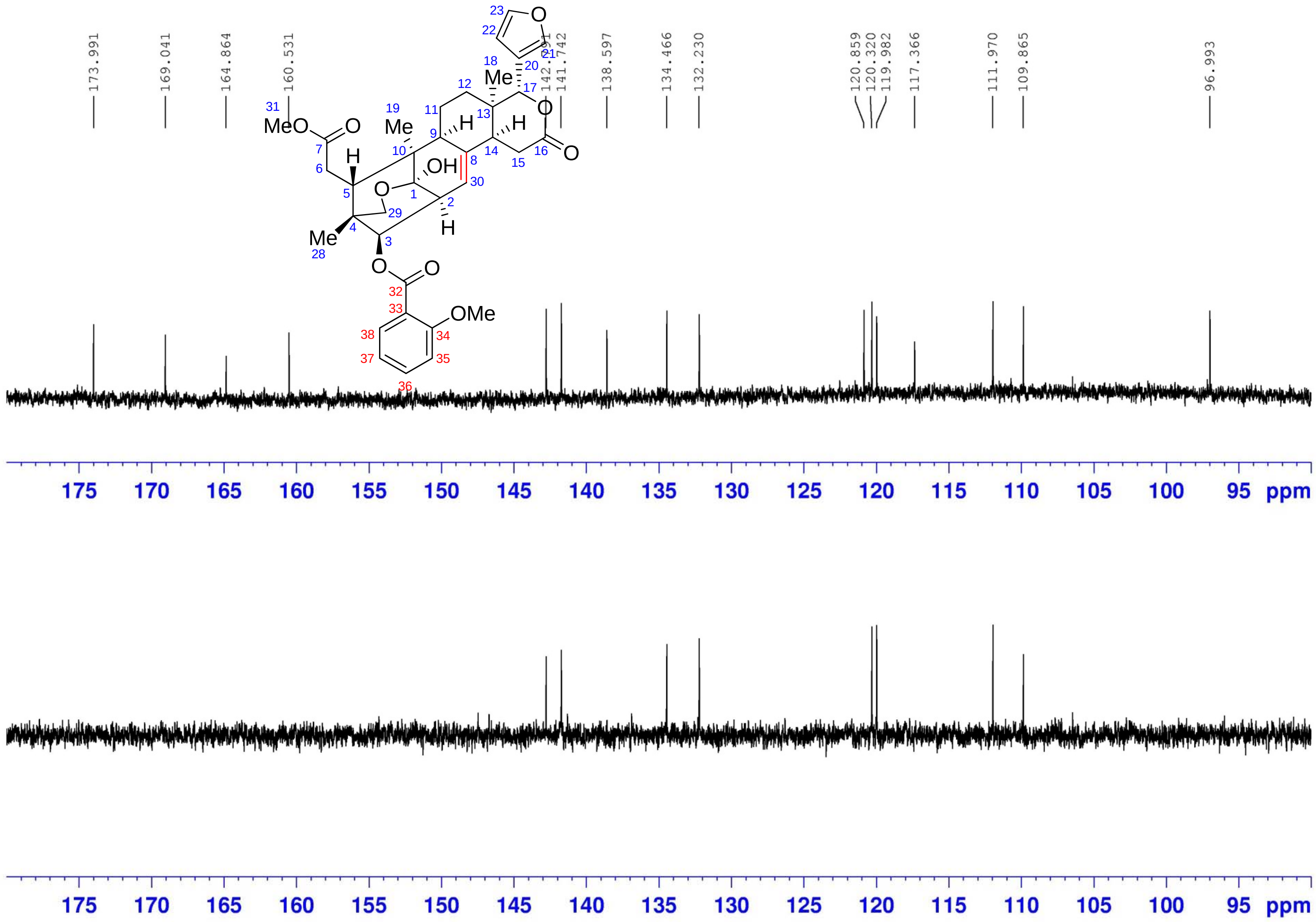

DEPT 135 spectrum of **8f** in $CDCl_3$

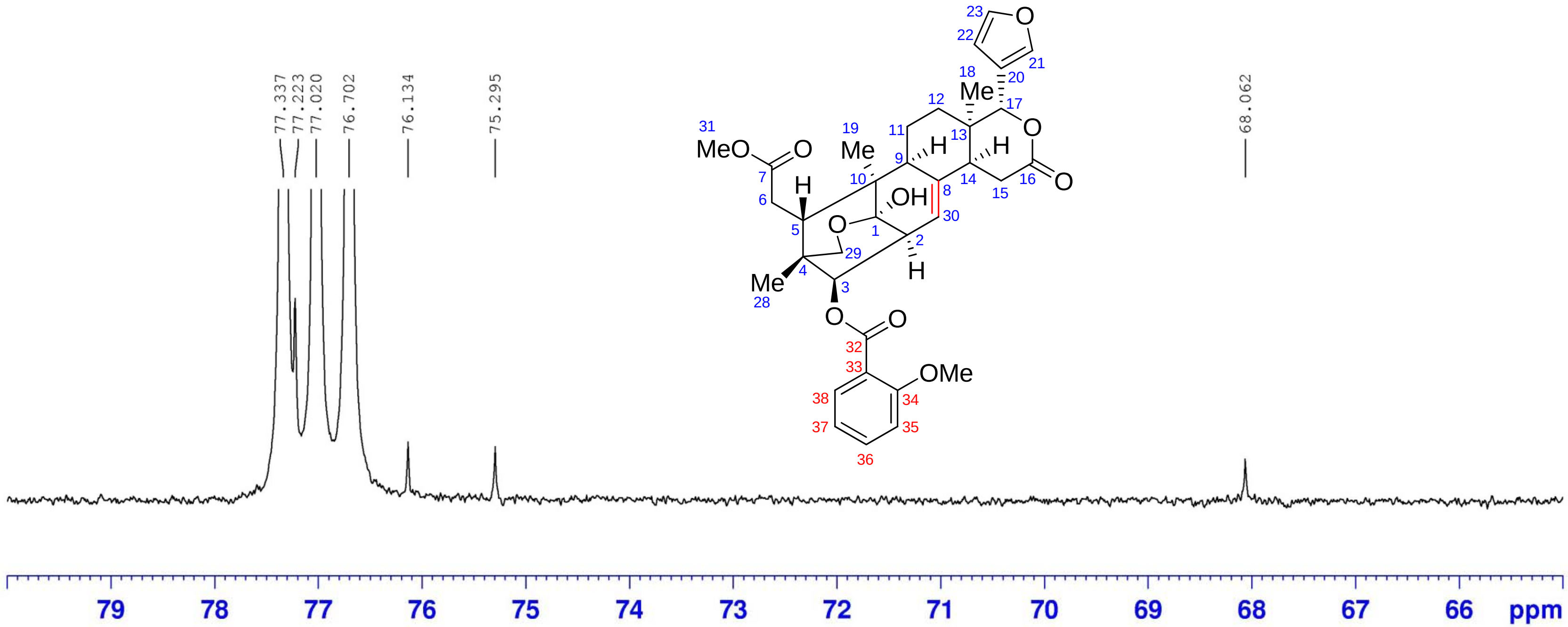

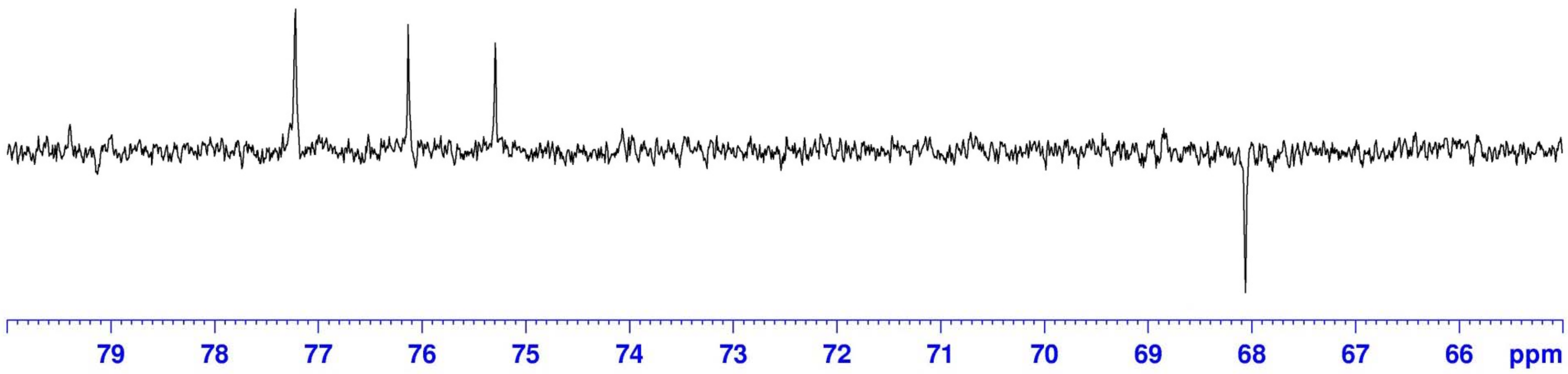

DEPT 135 spectrum of **8f** in $CDCl_3$

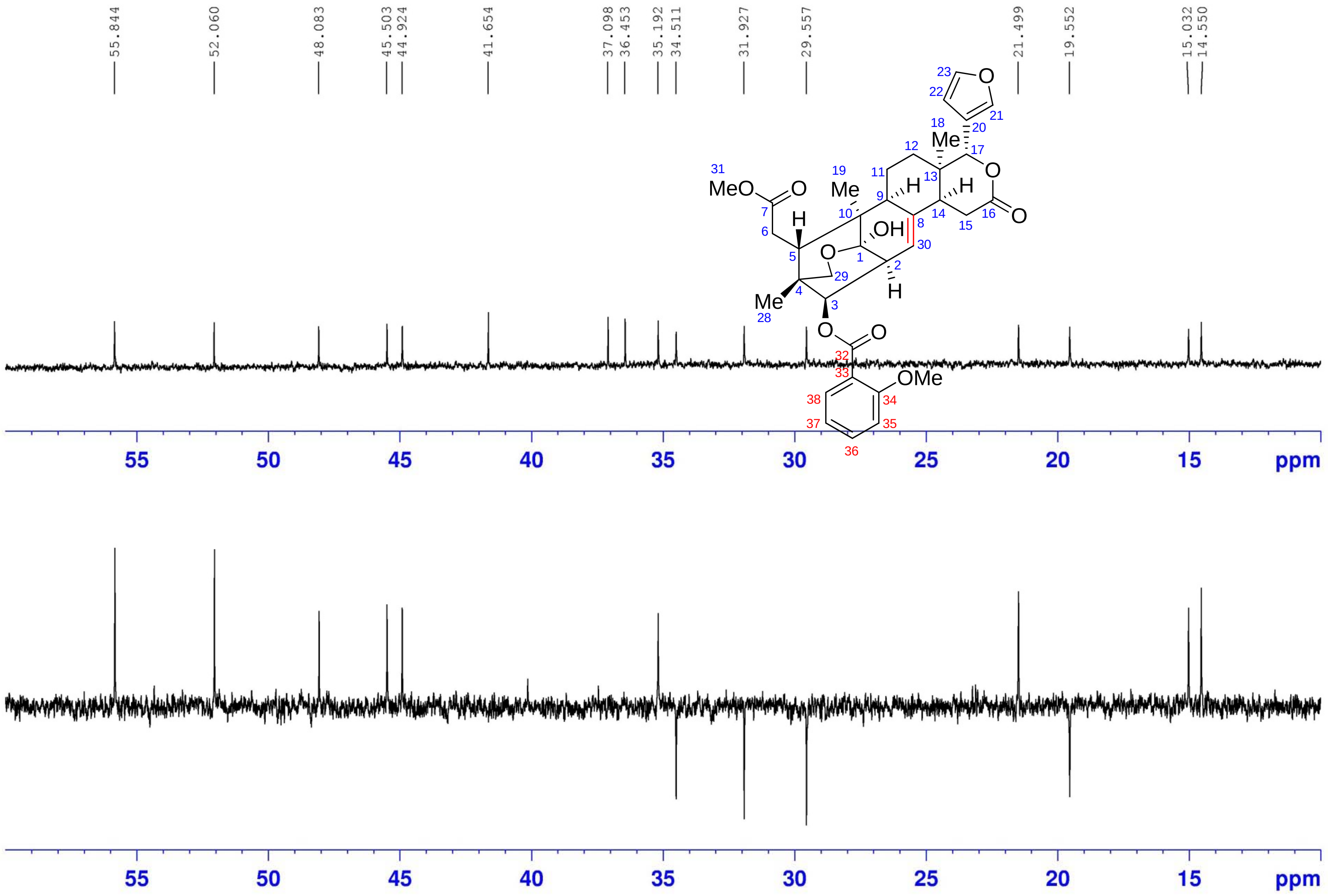

HR-ESIMS for compound **8g**

Generic Display Report

Analysis Info

Analysis Name	D:\Data\MS\data\201809\liwanshan_WSD-74_pos_19_01_5580.d	Acquisition Date	9/30/2018 6:22:50 PM
Method	LC_Direct Infusion_pos_100-1000mz.m	Operator	SCSIO
Sample Name	liwanshan_WSD-74_pos	Instrument	maXis
Comment			

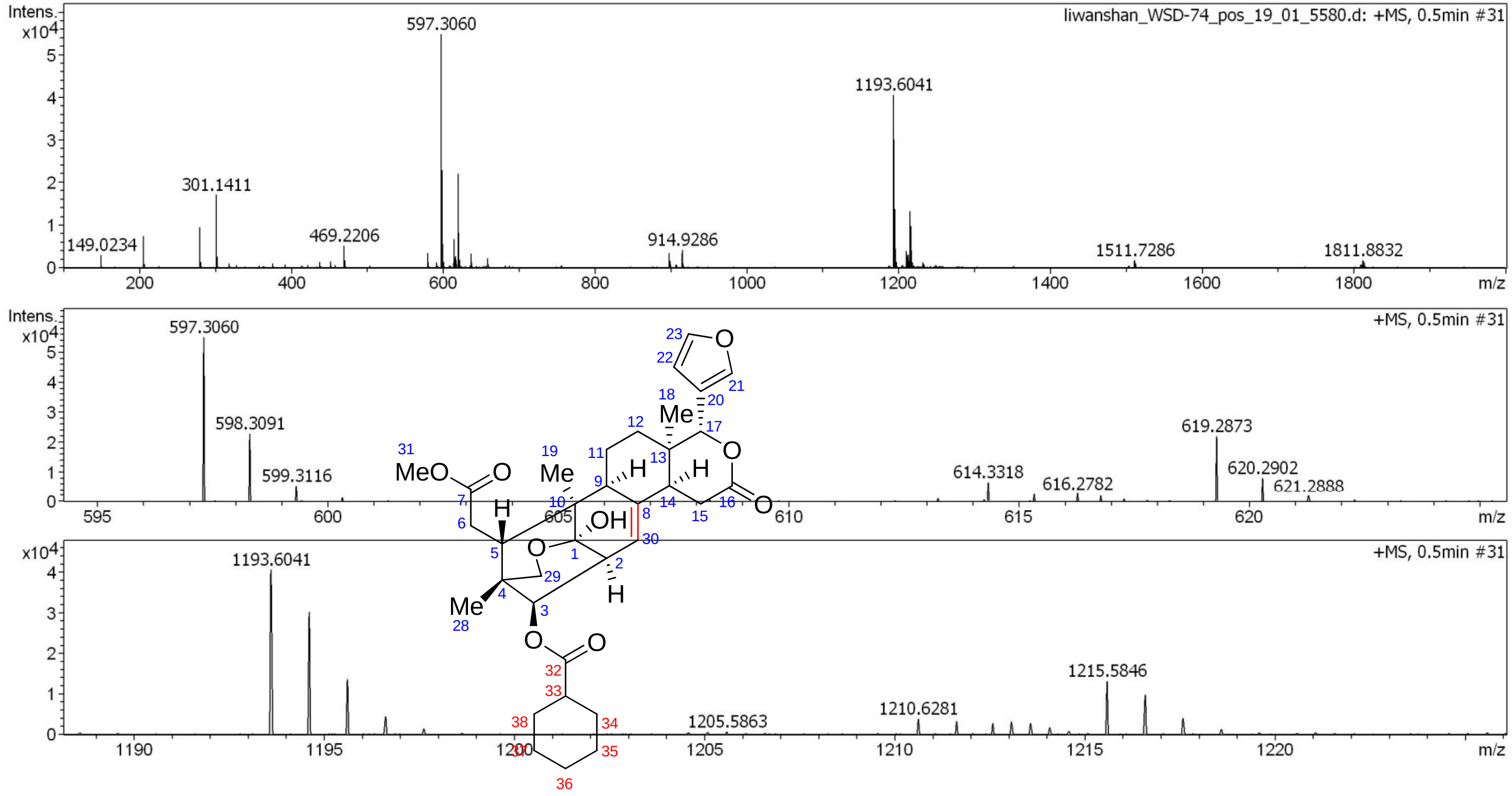

HR-ESIMS for compound **8g**

Mass Spectrum SmartFormula Report

Analysis Info

Analysis Name	D:\Data\MS\data\201809\liwanshan_WSD-74_pos_19_01_5580.d	Acquisition Date	9/30/2018 6:22:50 PM
Method	LC_Direct Infusion_pos_100-1000mz.m	Operator	SCSIO
Sample Name	liwanshan_WSD-74_pos	Instrument	maXis 255552.00029
Comment			

Acquisition Parameter

Source Type	ESI	Ion Polarity	Positive	Set Nebulizer	0.4 Bar
Focus	Active	Set Capillary	4500 V	Set Dry Heater	180 °C
Scan Begin	100 m/z	Set End Plate Offset	-500 V	Set Dry Gas	4.0 l/min
Scan End	2000 m/z	Set Charging Voltage	0 V	Set Divert Valve	Waste
		Set Corona	0 nA	Set APCI Heater	0 °C

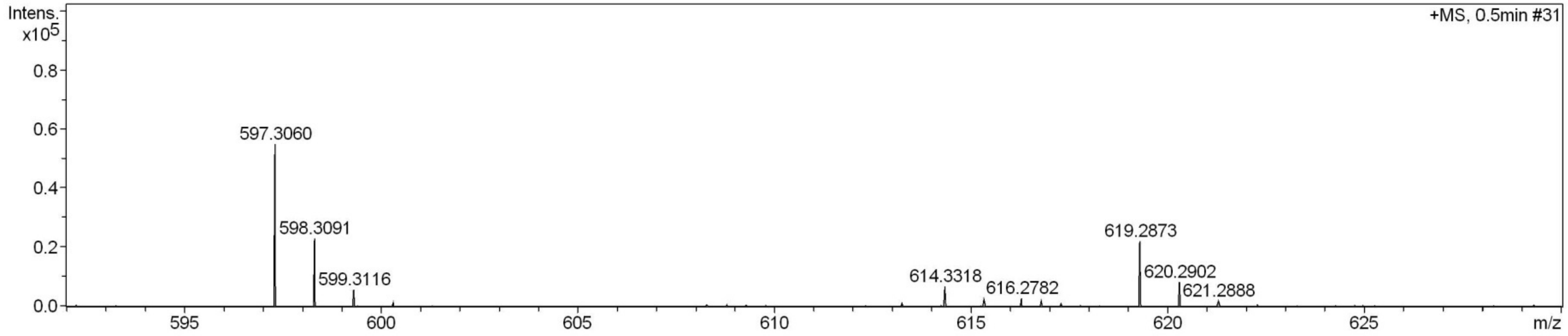

Meas. m/z	#	Ion Formula	Score	m/z	err [ppm]	err [mDa]	mSigma	rdb	e⁻ Conf	N-Rule
597.3060	1	C34H45O9	100.00	597.3058	-0.3	-0.2	22.1	12.5	even	ok
619.2873	1	C34H44NaO9	100.00	619.2878	-0.7	-0.5	5.8	12.5	even	ok
1193.6041	1	C68H89O18	100.00	1193.6043	-0.2	-0.2	11.3	24.5	even	ok
1215.5846	1	C68H88NaO18	100.00	1215.5863	1.4	1.7	8.7	24.5	even	ok

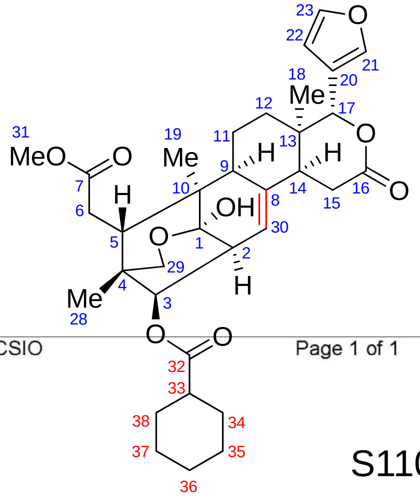

^1H NMR (400 MHz) spectrum of **8g** in $CDCl_3$

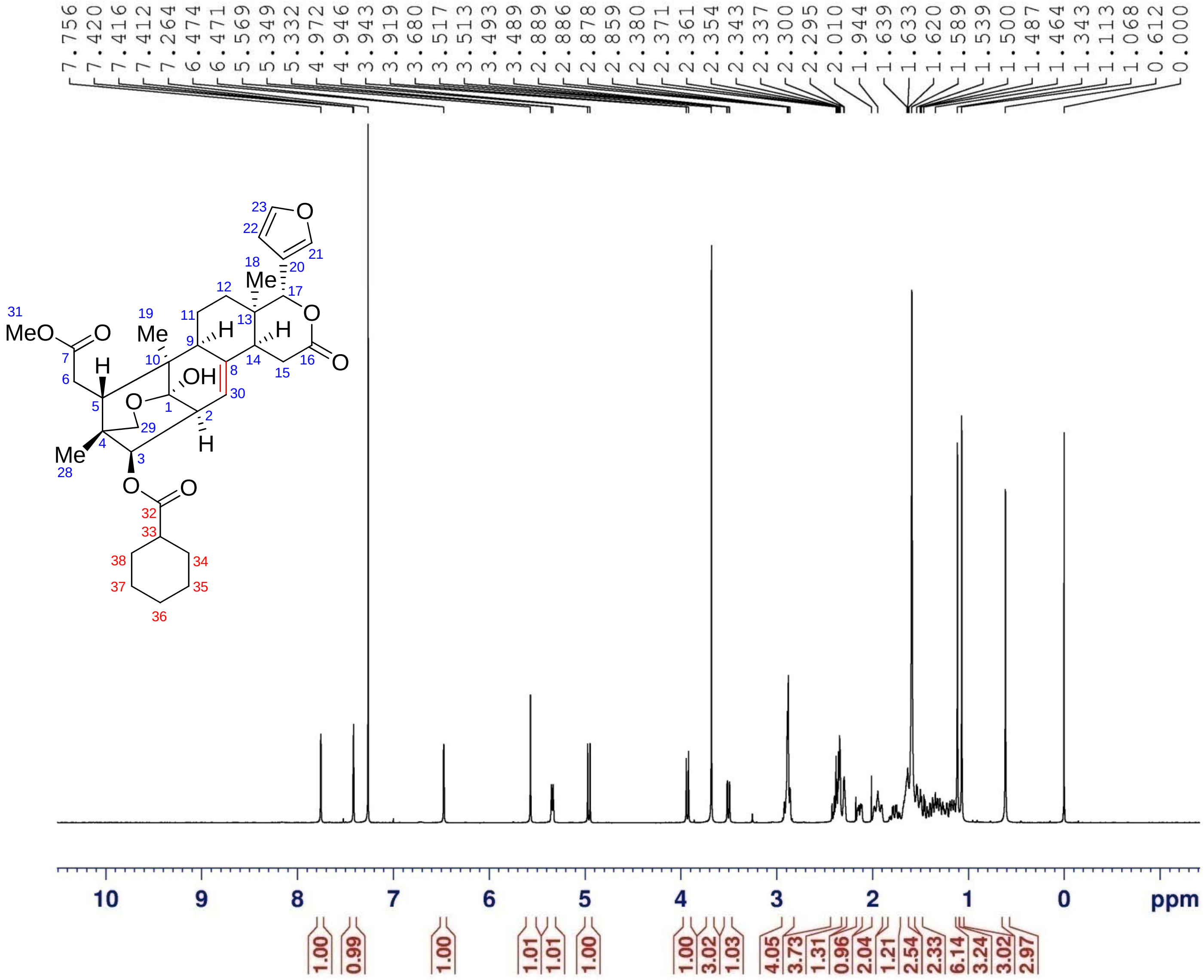


```
NAME       czc-1-CH3-huanjijiasu
EXPNO                      1
PROCNO                     1
Date_               20171016
Time                   15.33
INSTRUM                spect
PROBHD     5 mm CPPBBO BB
PULPROG                 zg30
TD                     65536
SOLVENT                CDCl3
NS                        16
DS                         2
SWH                 8223.685 Hz
FIDRES              0.125483 Hz
AQ                 3.9846387 sec
RG                     208.5
DW                    60.800 usec
DE                     10.00 usec
TE                     297.0 K
D1                1.00000000 sec
TD0                        1

======== CHANNEL f1 ========
SFO1             400.1324710 MHz
NUC1                      1H
P1                     11.50 usec
SI                     65536
SF               400.1300082 MHz
WDW                       EM
SSB                        0
LB                      0.30 Hz
GB                         0
PC                      1.00
```

^{1}H NMR (400 MHz) spectrum of **8g** in $CDCl_3$

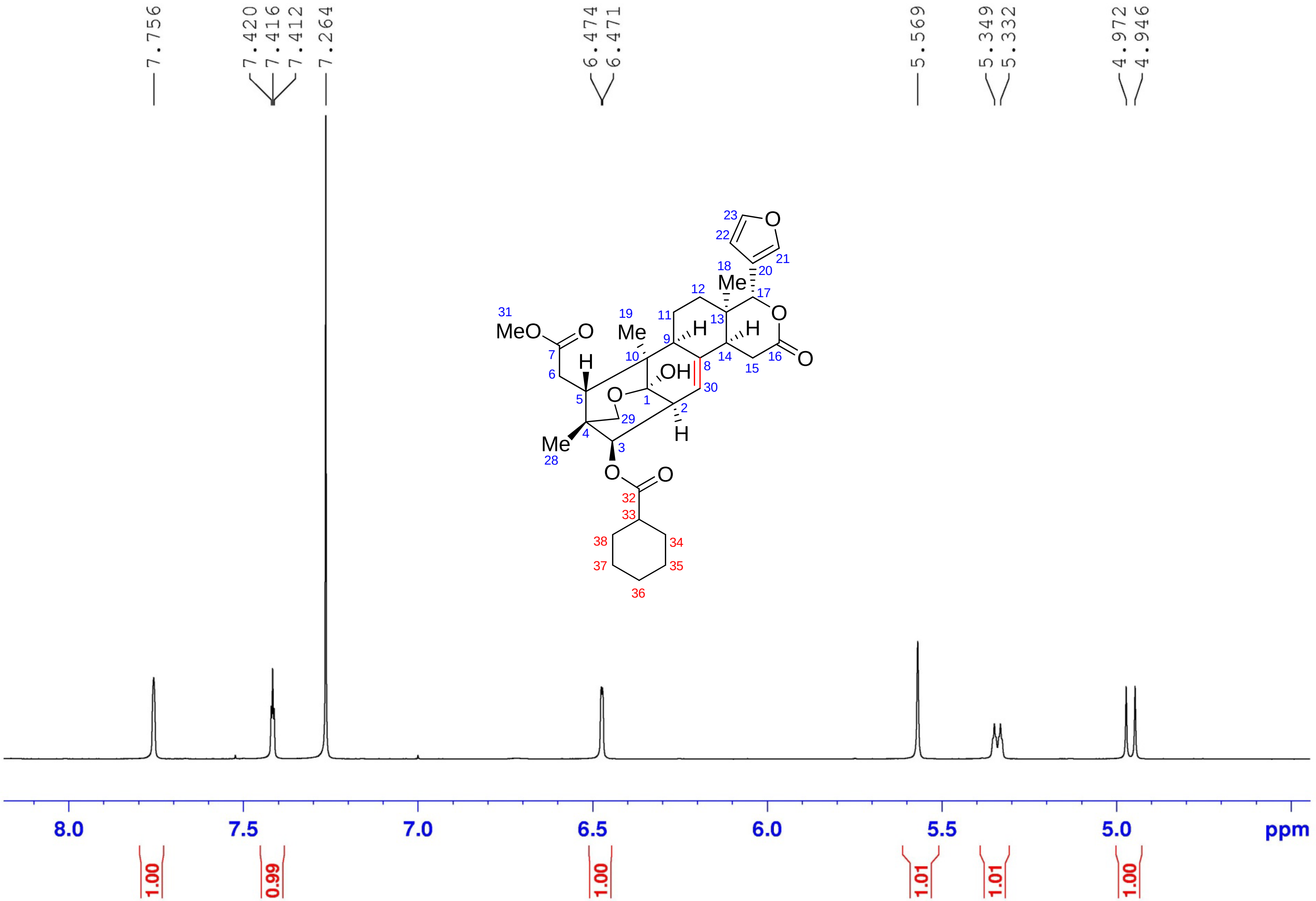

^{1}H NMR (400 MHz) spectrum of **8g** in $CDCl_3$

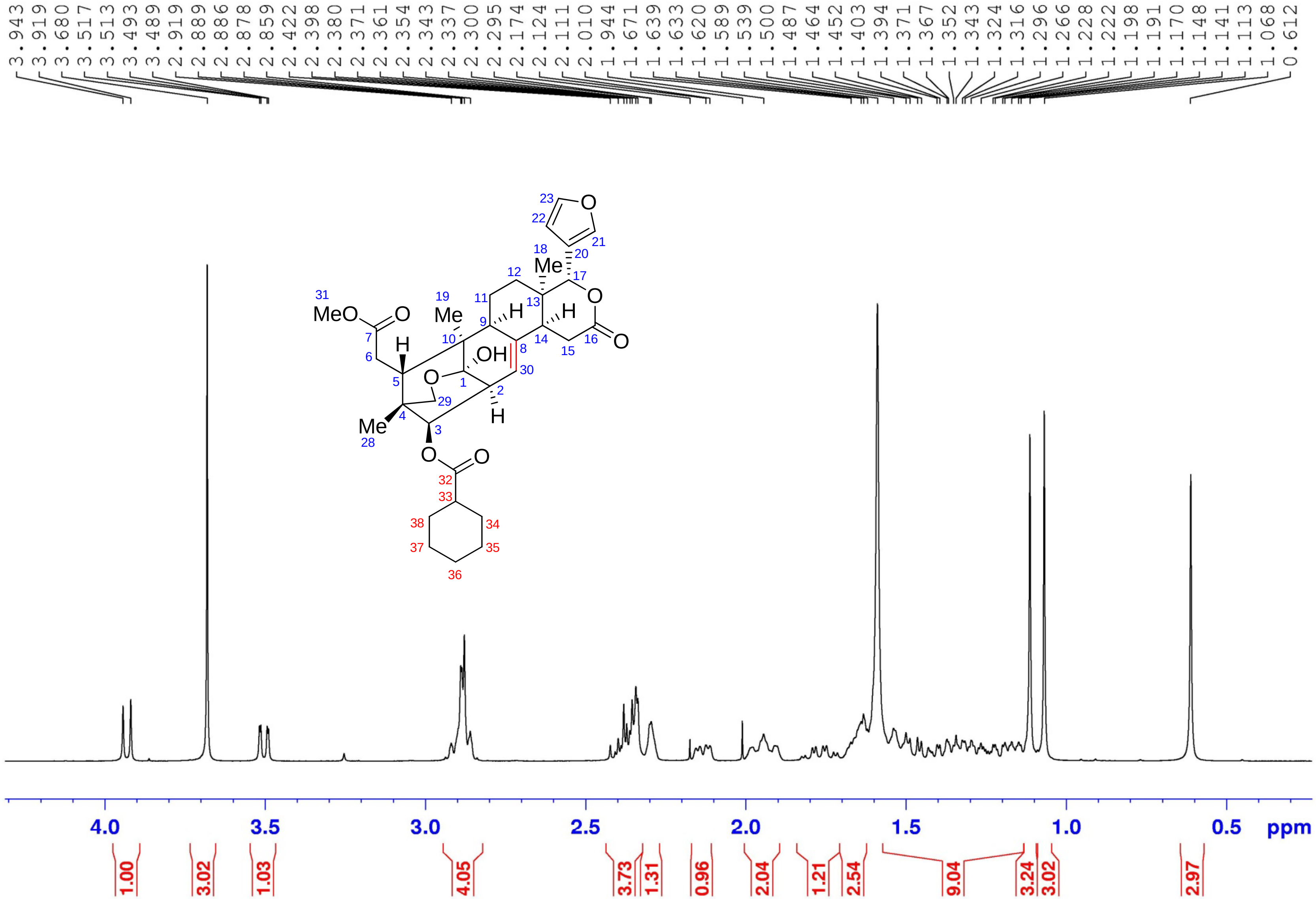

^{13}C NMR (100 MHz) spectrum of **8g** in $CDCl_3$

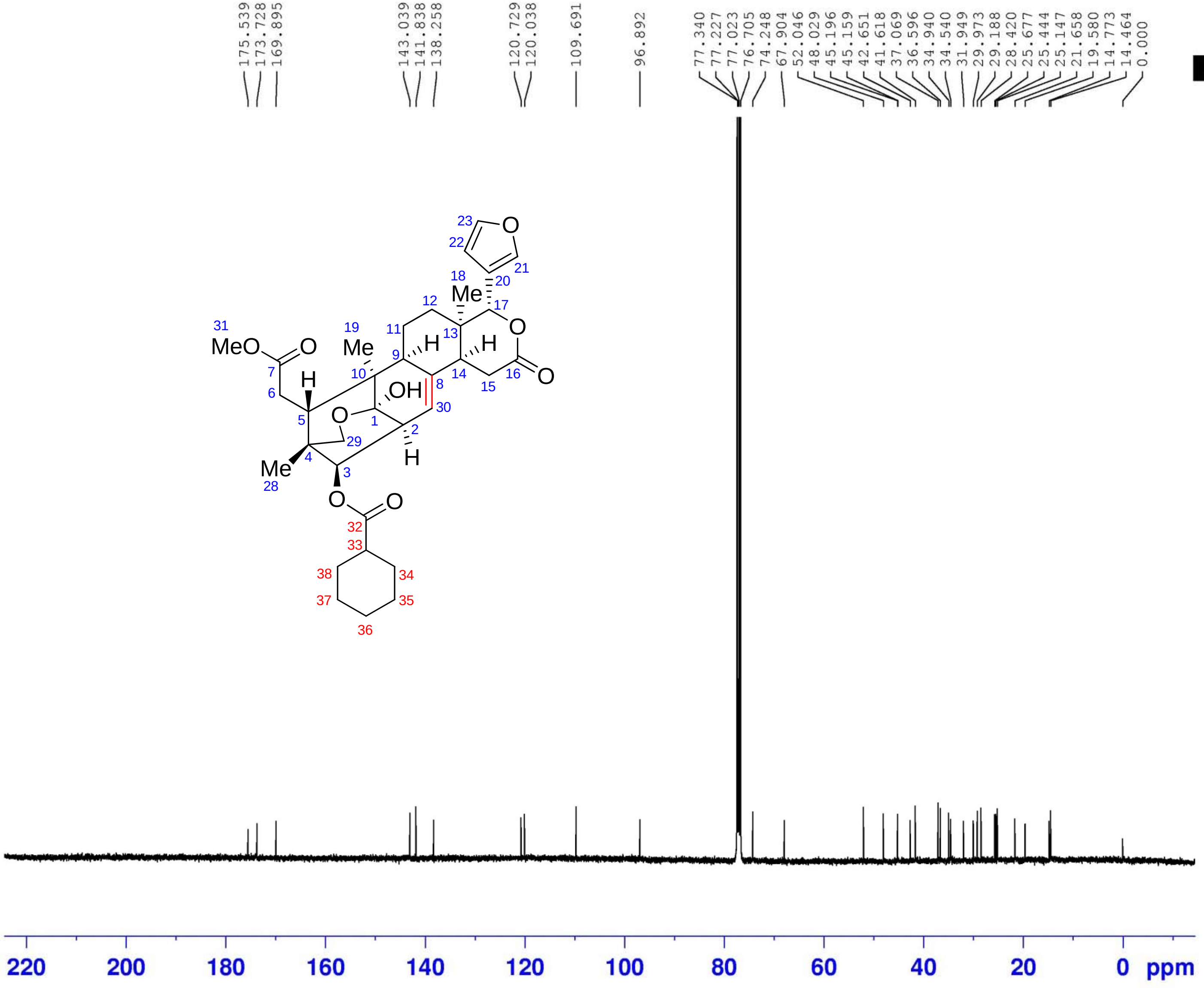

NAME	czc-1-CH3-huanjijia
EXPNO	2
PROCNO	1
Date_	20171016
Time	22.33
INSTRUM	spect
PROBHD	5 mm CPPBBO BB
PULPROG	zgpg30
TD	65536
SOLVENT	CDCl3
NS	1024
DS	4
SWH	24038.461 Hz
FIDRES	0.366798 Hz
AQ	1.3631988 sec
RG	85.34
DW	20.800 usec
DE	18.00 usec
TE	297.0 K
D1	2.00000000 sec
D11	0.03000000 sec
TD0	1

======== CHANNEL f1 ========

SFO1	100.6233324 MHz
NUC1	13C
P1	10.00 usec
SI	32768
SF	100.6127689 MHz
WDW	EM
SSB	0
LB	1.00 Hz
GB	0
PC	1.40

^{13}C NMR (100 MHz) spectrum of **8g** in $CDCl_3$

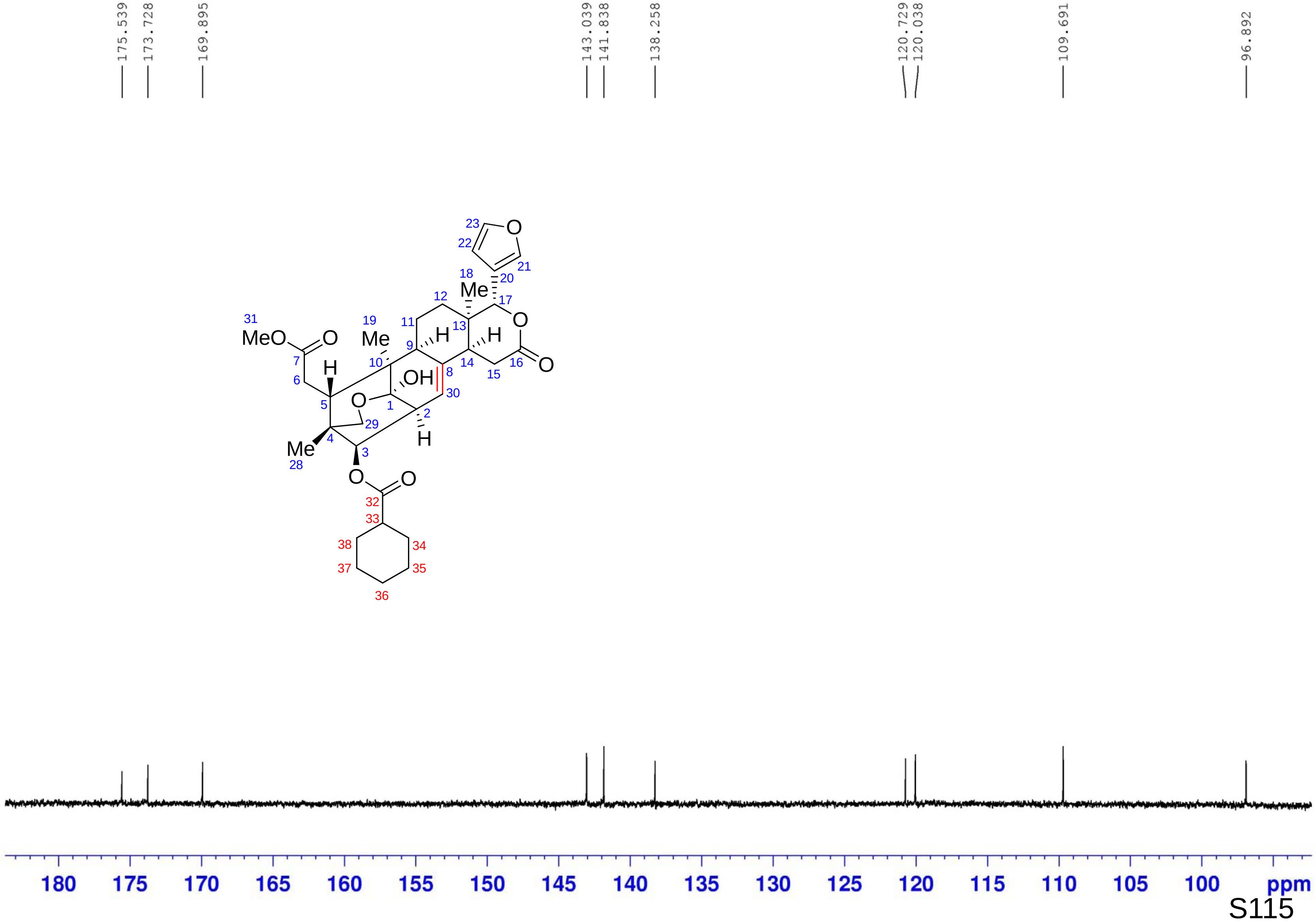

^{13}C NMR (100 MHz) spectrum of **8g** in $CDCl_3$

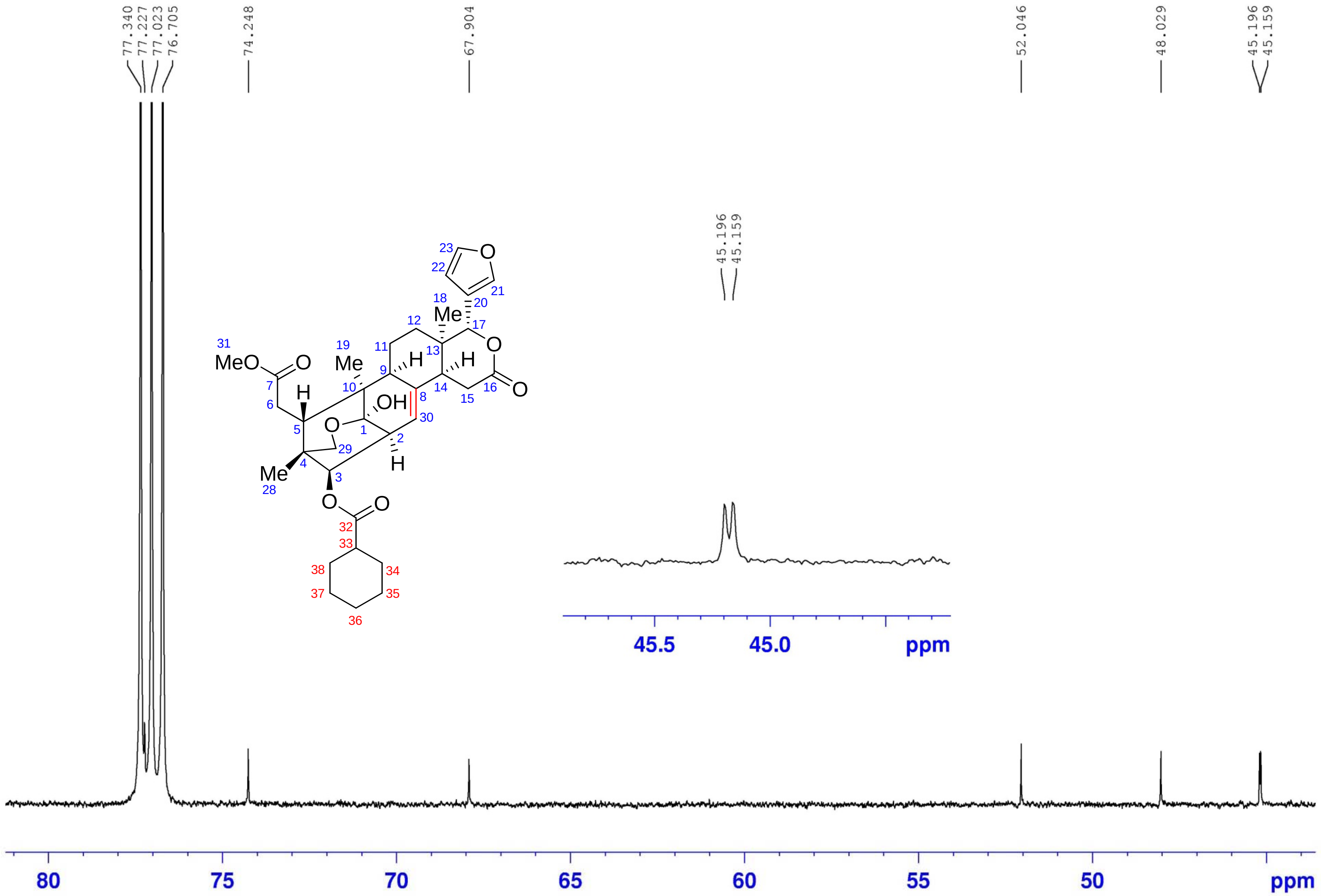

^{13}C NMR (100 MHz) spectrum of **8g** in $CDCl_3$

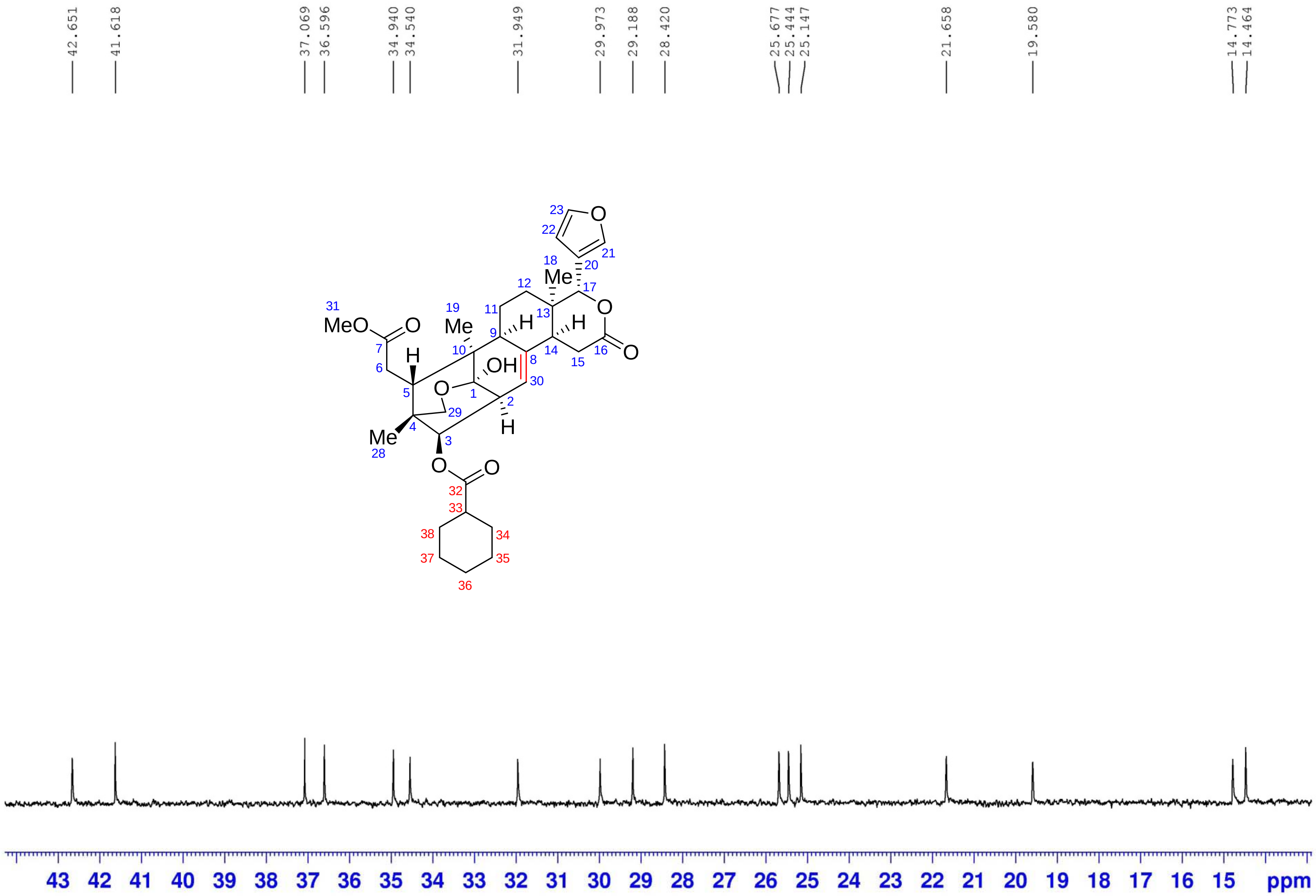

DEPT 135 spectrum of **8g** in $CDCl_3$

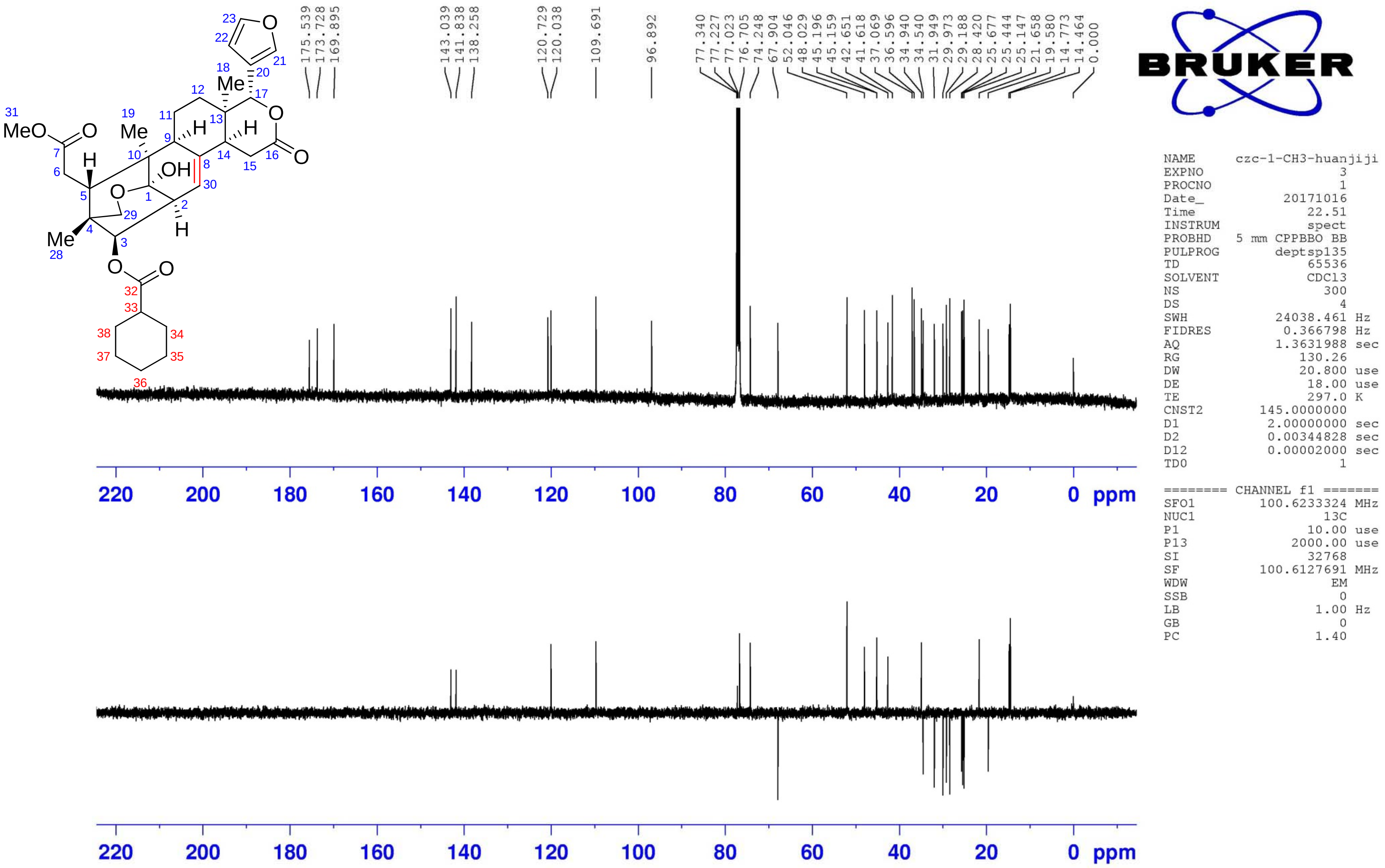

DEPT 135 spectrum of **8g** in $CDCl_3$

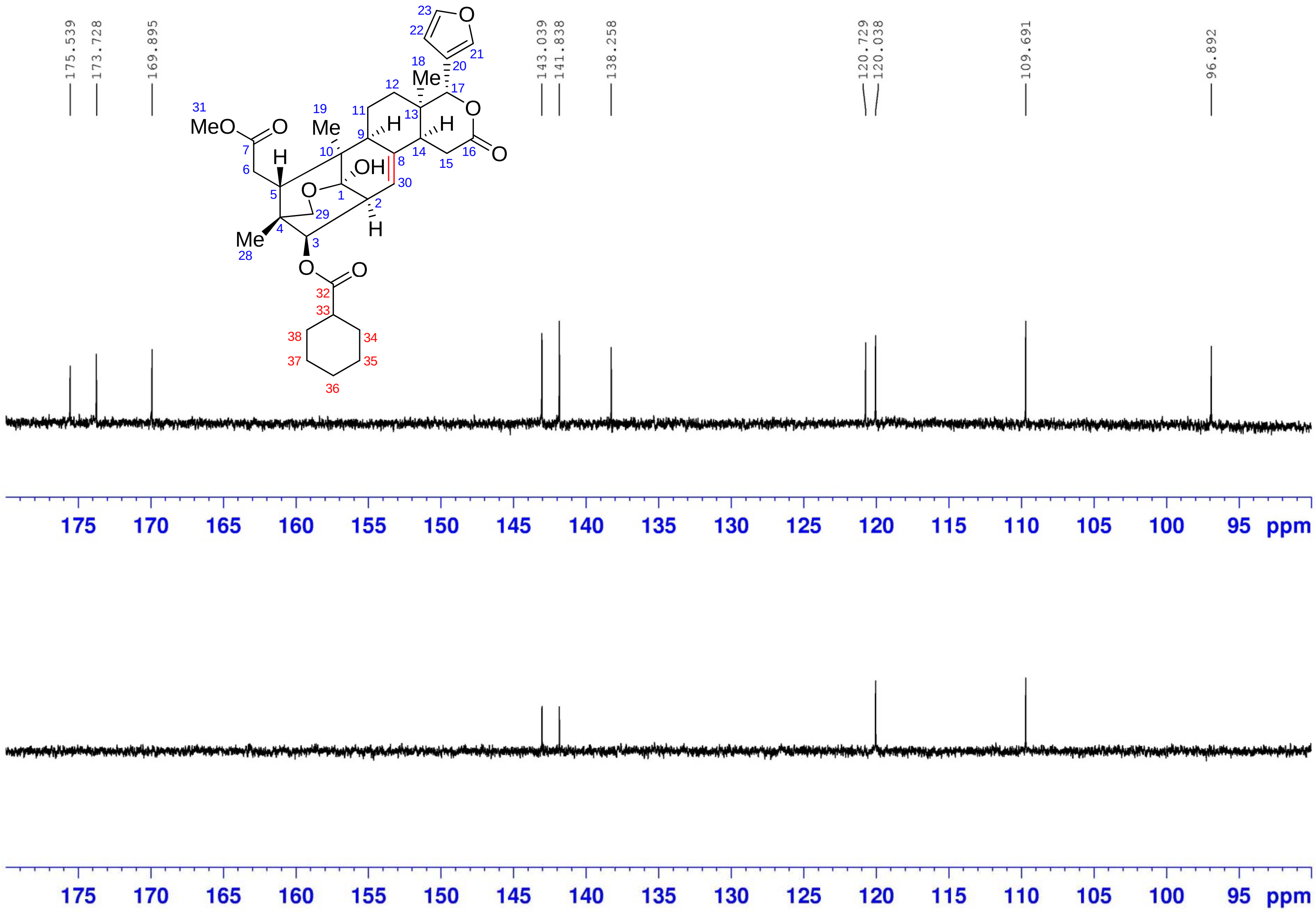

DEPT 135 spectrum of **8g** in $CDCl_3$

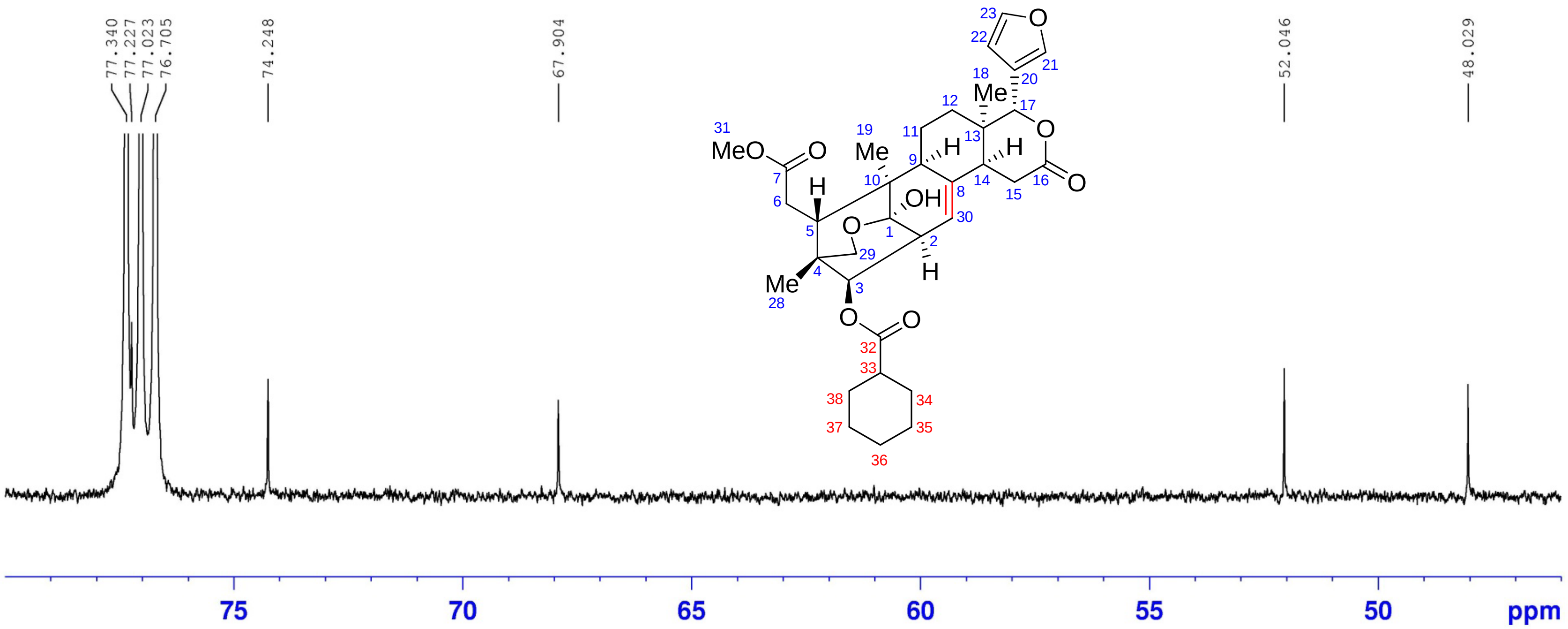

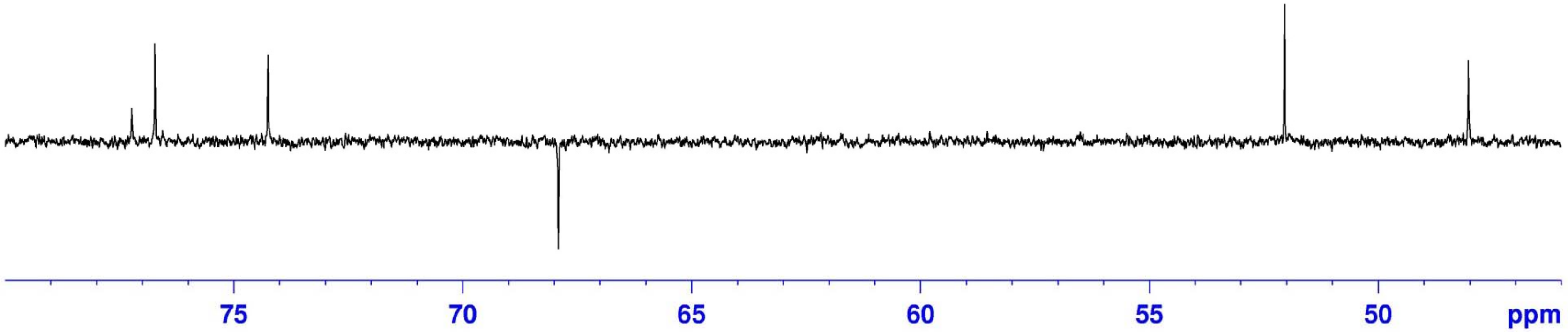

DEPT 135 spectrum of **8g** in $CDCl_3$

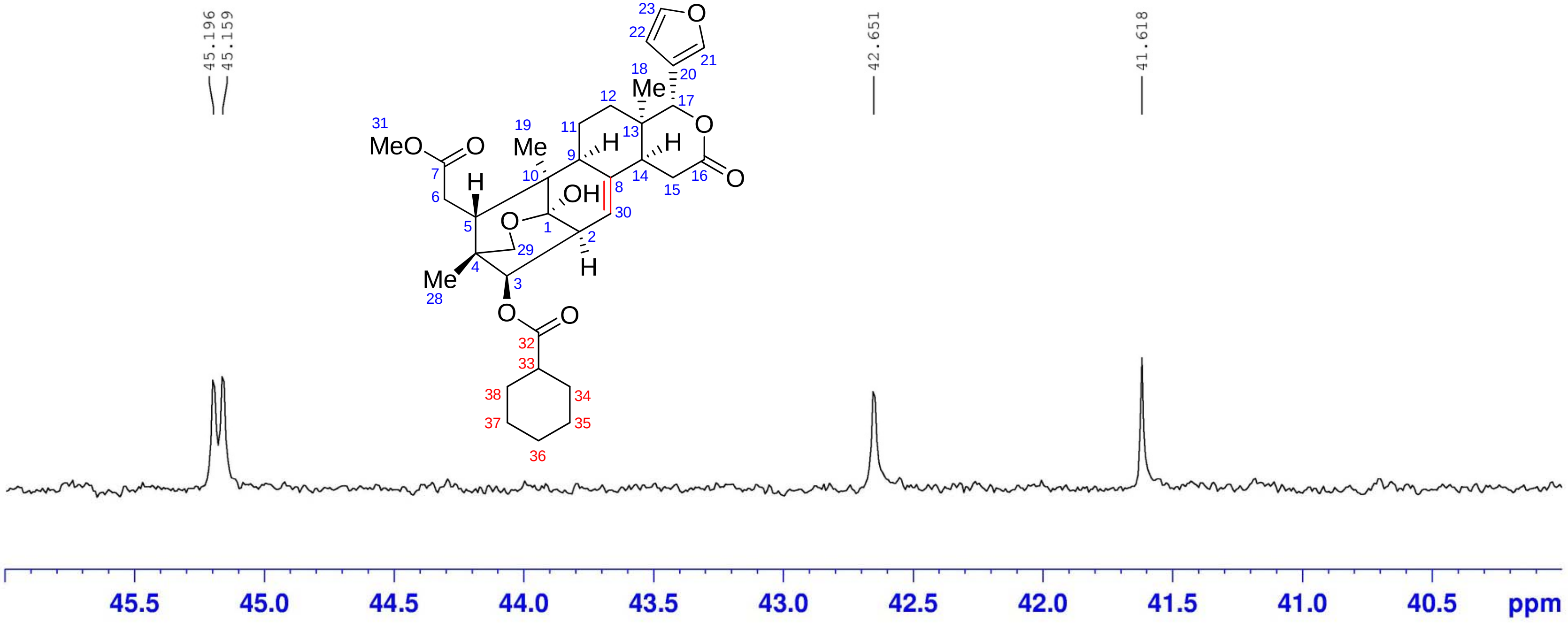

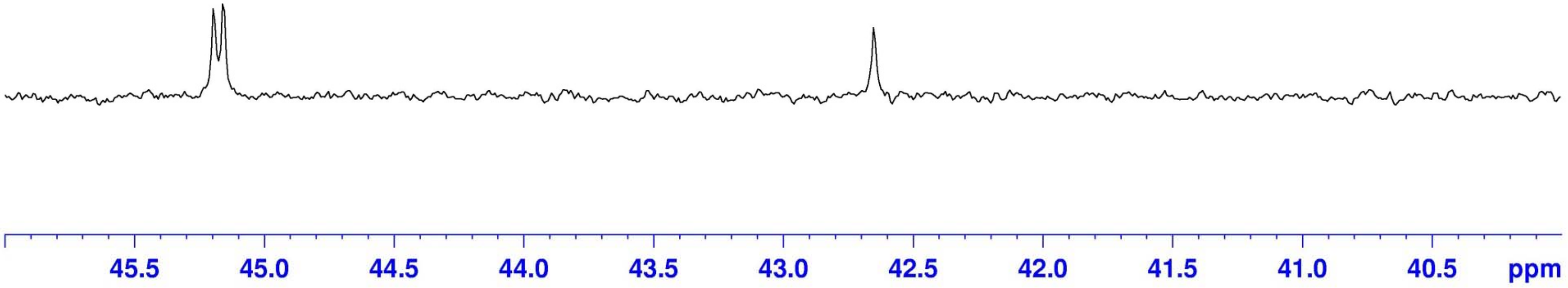

DEPT 135 spectrum of **8g** in $CDCl_3$

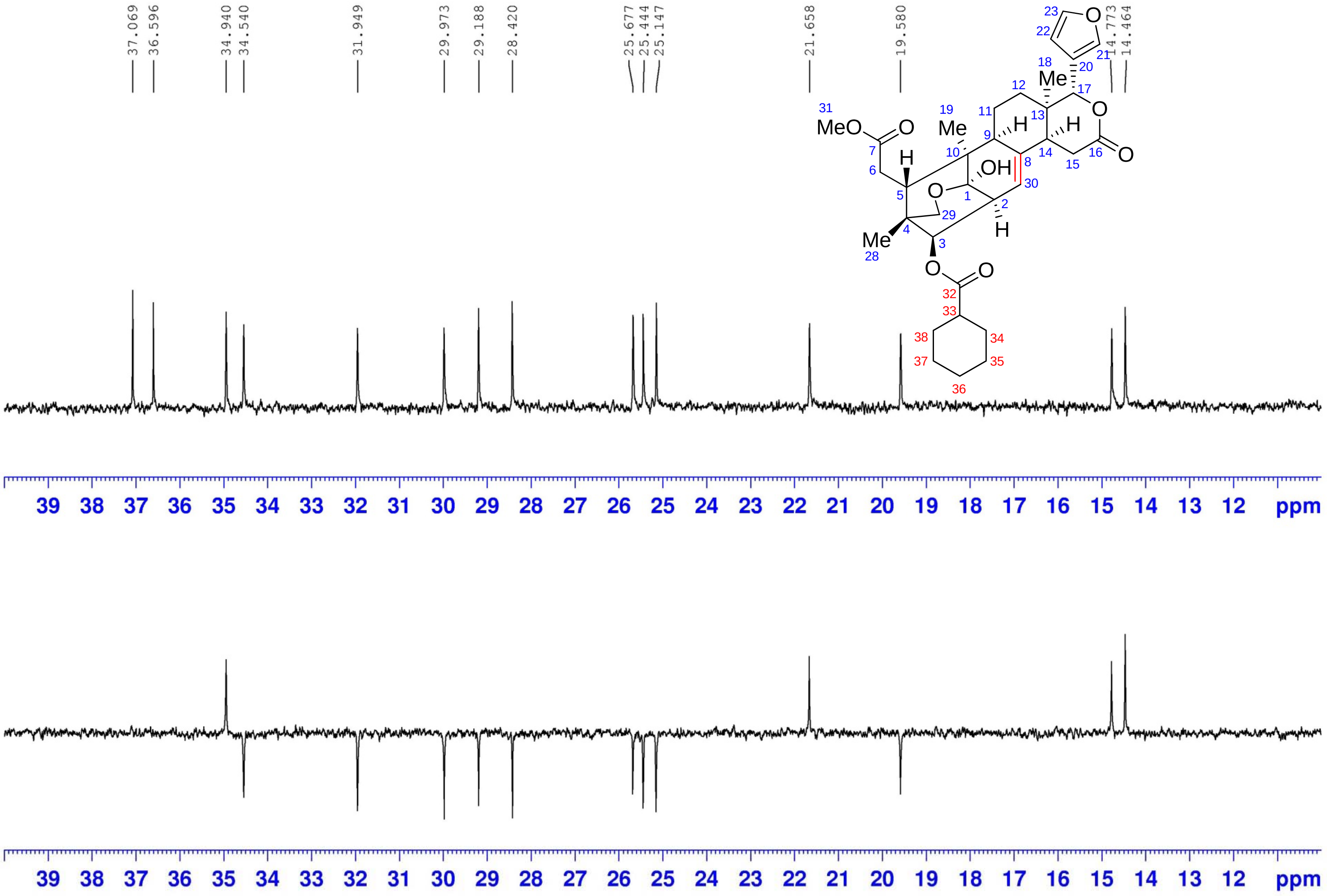

HR-ESIMS for compound **8h**

Generic Display Report

**Analysis Info**		Acquisition Date	9/30/2018 4:07:58 PM
Analysis Name	D:\Data\MS\data\201809\liwanshan_WSD-73_pos_14_01_5571.d		
Method	LC_Direct Infusion_pos_100-1000mz.m	Operator	SCSIO
Sample Name	liwanshan_WSD-73_pos	Instrument	maXis
Comment			

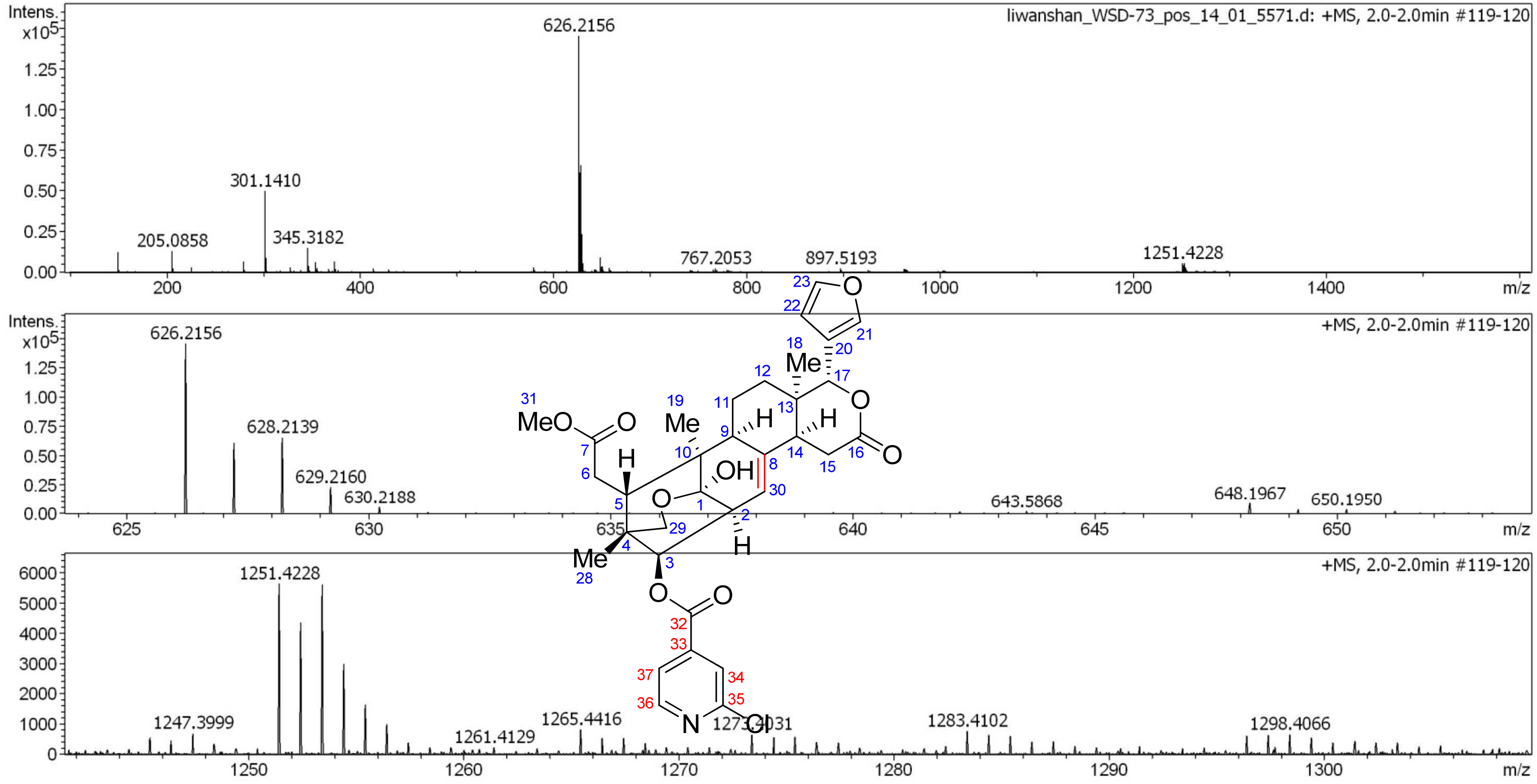

Bruker Compass DataAnalysis 4.1 printed: 9/30/2018 4:19:52 PM by: SCSIO Page 1 of 1

HR-ESIMS for compound **8h**

Mass Spectrum SmartFormula Report

Analysis Info

Analysis Name	D:\Data\MS\data\201809\liwanshan_WSD-73_pos_14_01_5571.d	Acquisition Date	9/30/2018 4:07:58 PM
Method	LC_Direct Infusion_pos_100-1000mz.m	Operator	SCSIO
Sample Name	liwanshan_WSD-73_pos	Instrument	maXis 255552.00029
Comment			

Acquisition Parameter

Source Type	ESI	Ion Polarity	Positive	Set Nebulizer	0.4 Bar
Focus	Active	Set Capillary	4500 V	Set Dry Heater	180 °C
Scan Begin	100 m/z	Set End Plate Offset	-500 V	Set Dry Gas	4.0 l/min
Scan End	2000 m/z	Set Charging Voltage	0 V	Set Divert Valve	Waste
		Set Corona	0 nA	Set APCI Heater	0 °C

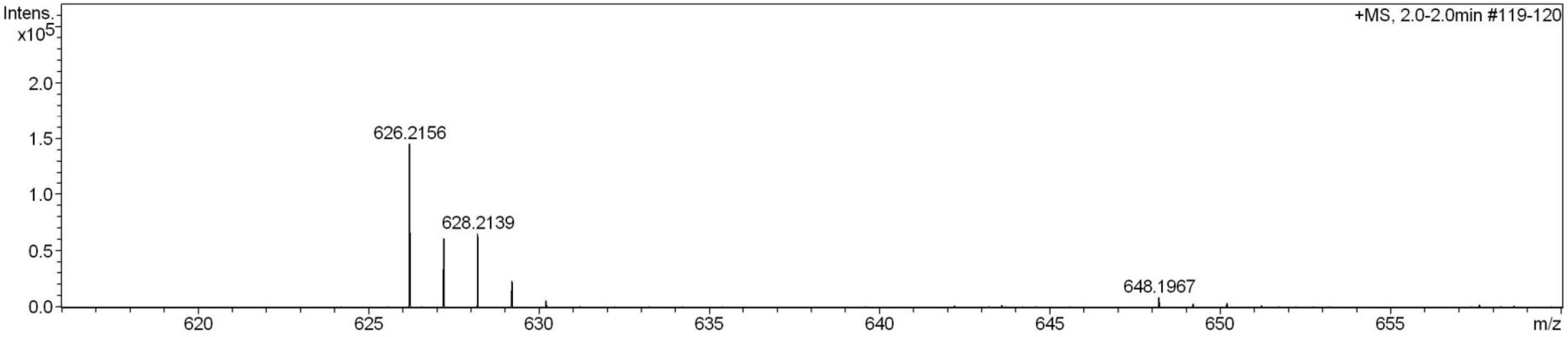

Meas. m/z	#	Ion Formula	Score	m/z	err [ppm]	err [mDa]	mSigma	rdb	e⁻ Conf	N-Rule
626.2156	1	C33H37ClNO9	100.00	626.2151	-0.8	-0.5	31.6	15.5	even	ok
648.1967	1	C33H36ClNNaO9	100.00	648.1971	0.5	0.3	26.0	15.5	even	ok

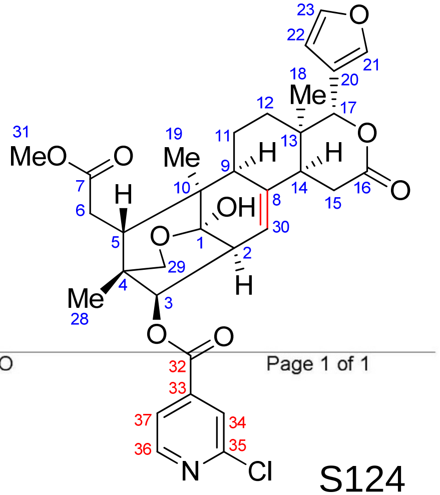

^{1}H NMR (400 MHz) spectrum of **8h** in $CDCl_3$

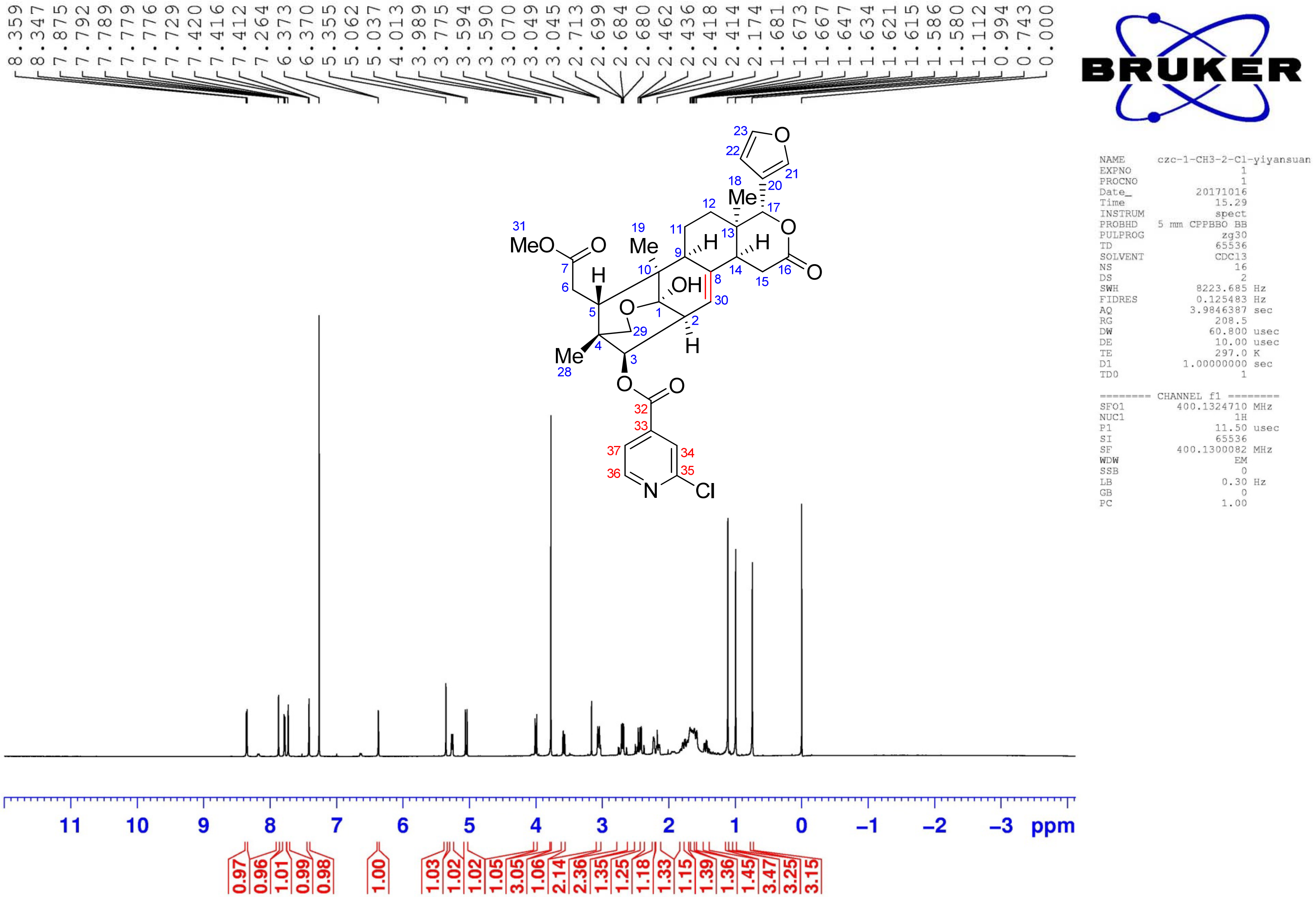

^{1}H NMR (400 MHz) spectrum of **8h** in $CDCl_3$

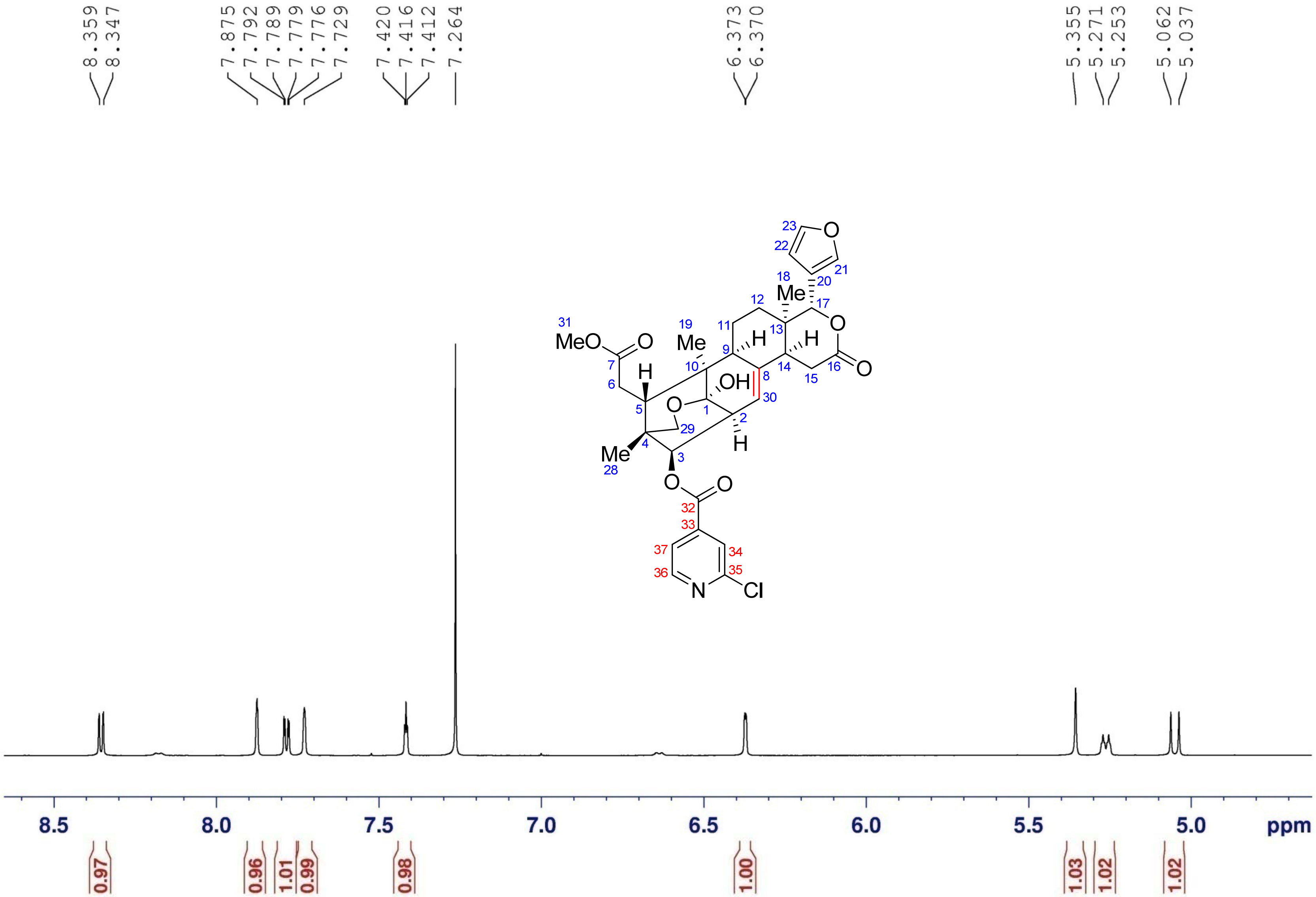

^{1}H NMR (400 MHz) spectrum of **8h** in $CDCl_3$

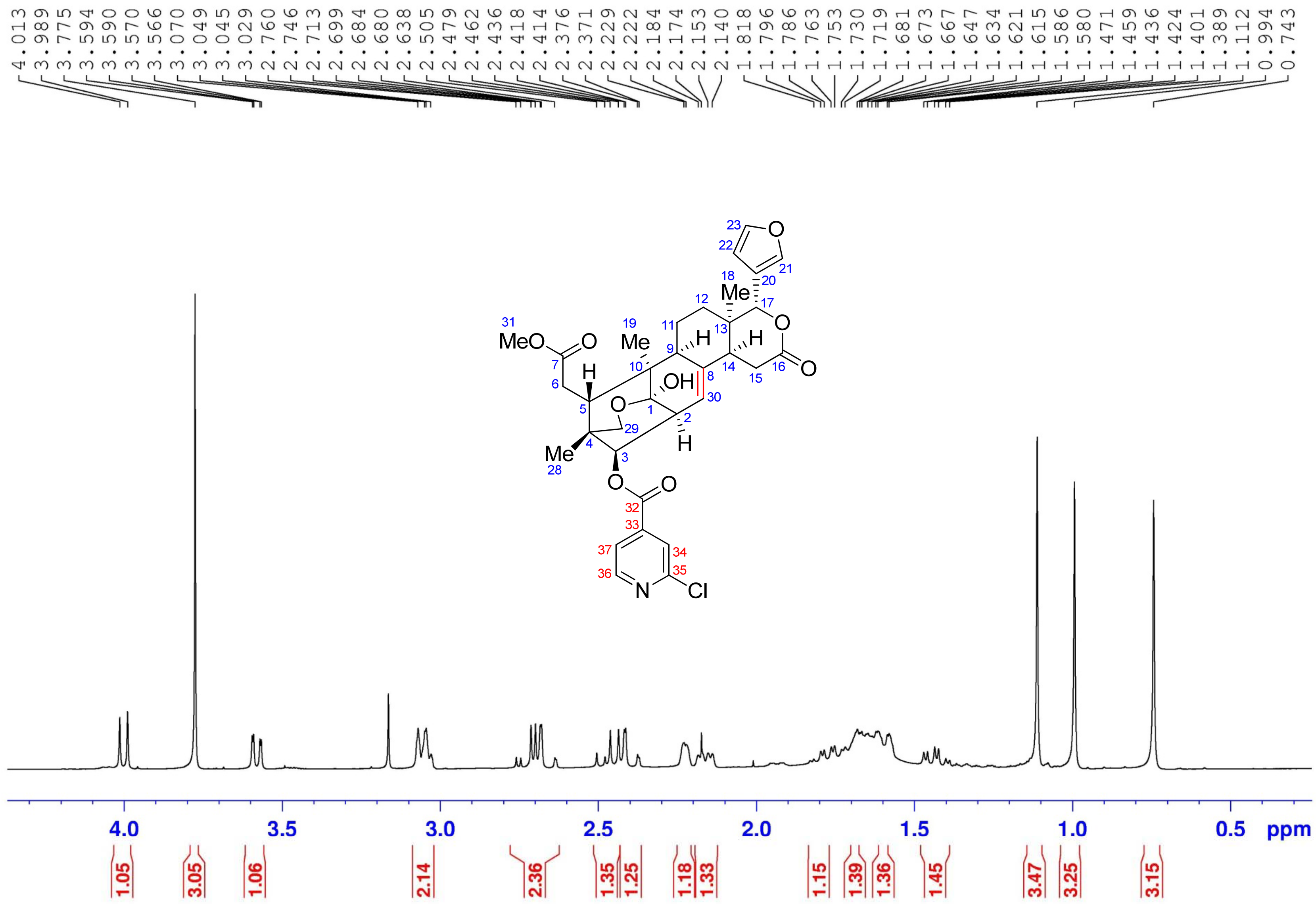

^{13}C NMR (100 MHz) spectrum of **8h** in $CDCl_3$

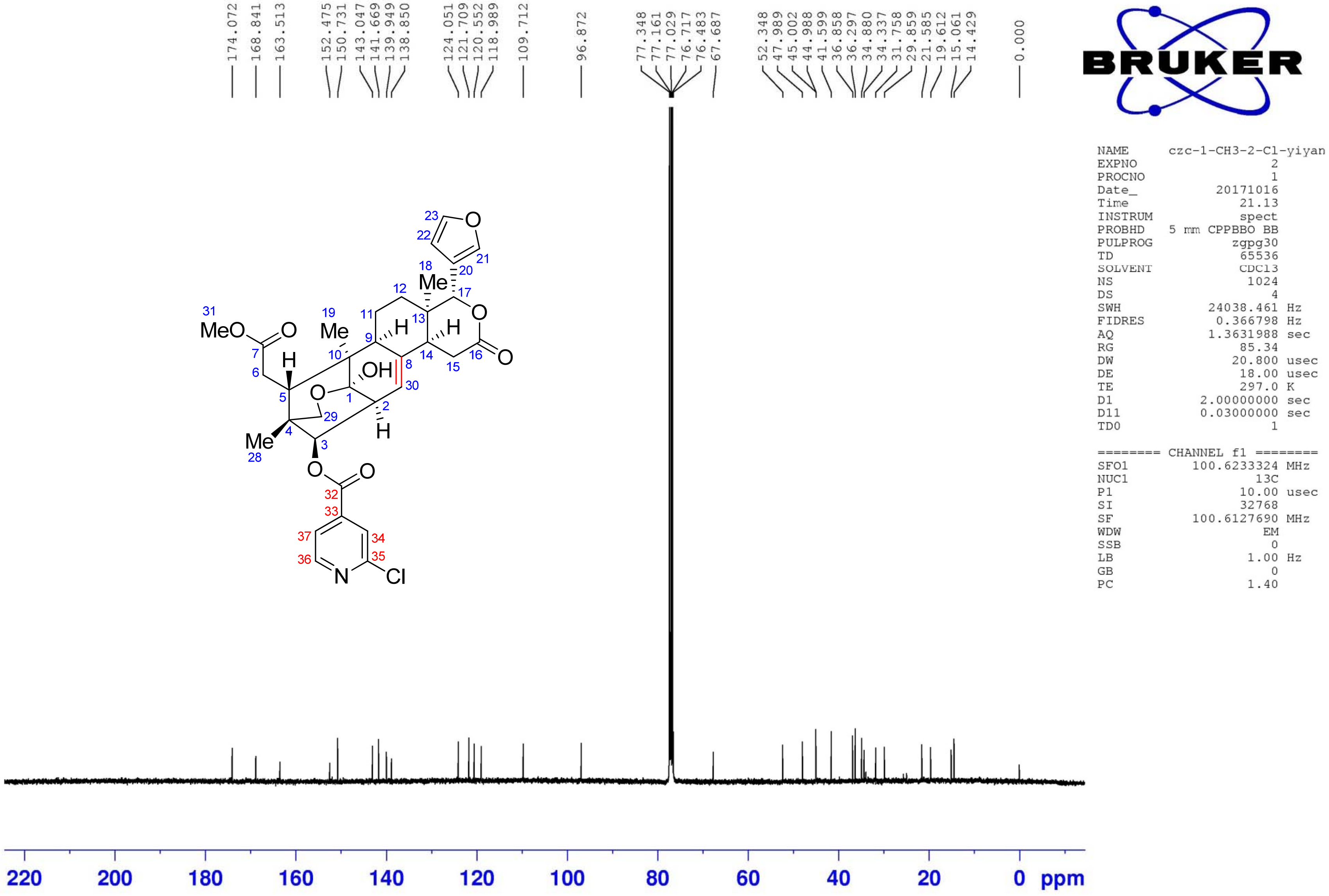


```
NAME       czc-1-CH3-2-Cl-yiyan
EXPNO                     2
PROCNO                    1
Date_              20171016
Time                  21.13
INSTRUM               spect
PROBHD   5 mm CPPBBO BB
PULPROG              zgpg30
TD                    65536
SOLVENT               CDCl3
NS                     1024
DS                        4
SWH              24038.461 Hz
FIDRES            0.366798 Hz
AQ               1.3631988 sec
RG                    85.34
DW                   20.800 usec
DE                    18.00 usec
TE                    297.0 K
D1               2.00000000 sec
D11              0.03000000 sec
TD0                       1

======== CHANNEL f1 ========
SFO1         100.6233324 MHz
NUC1                    13C
P1                    10.00 usec
SI                    32768
SF           100.6127690 MHz
WDW                      EM
SSB                       0
LB                     1.00 Hz
GB                        0
PC                     1.40
```

^{13}C NMR (100 MHz) spectrum of **8h** in $CDCl_3$

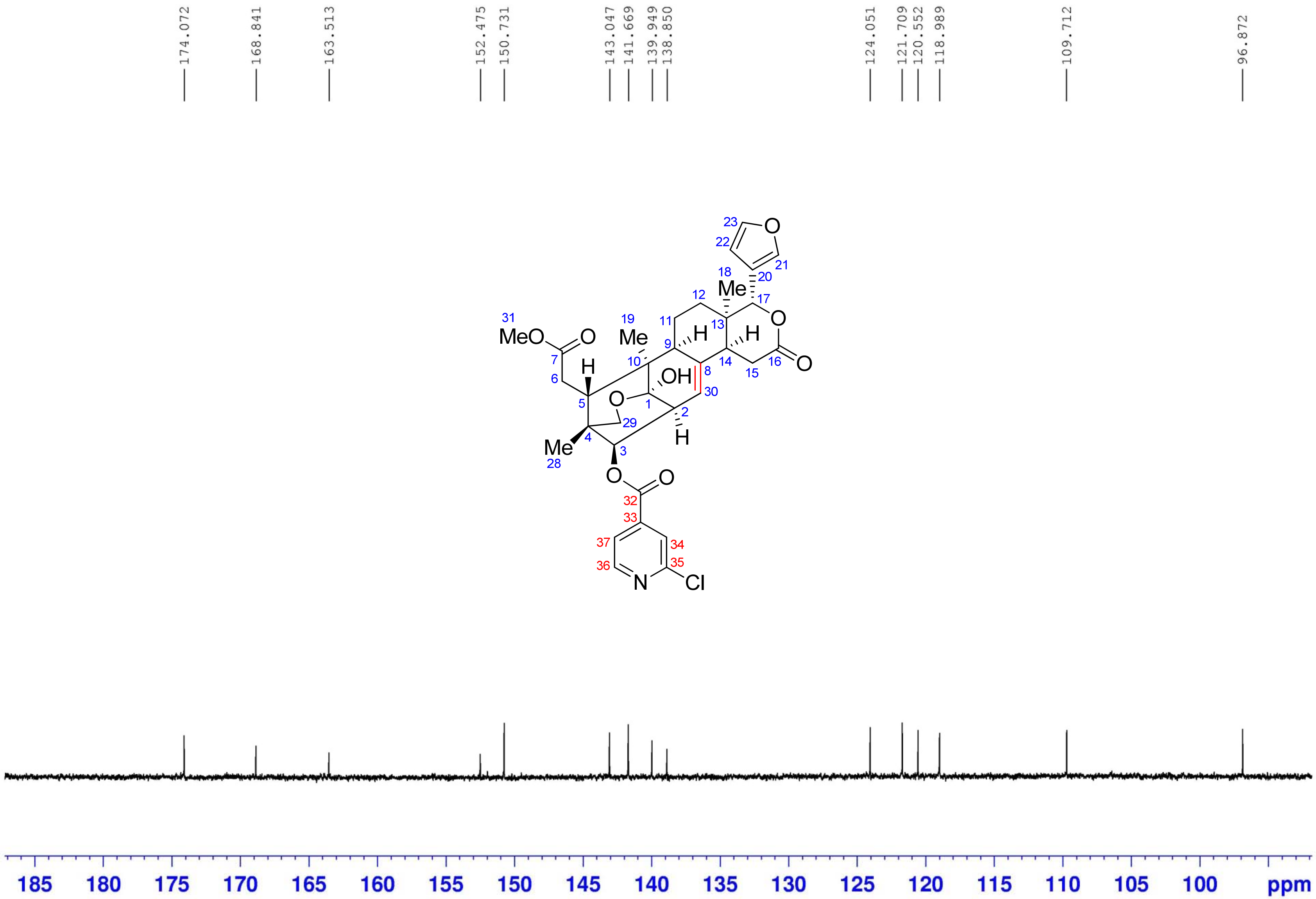

^{13}C NMR (100 MHz) spectrum of **8h** in $CDCl_3$

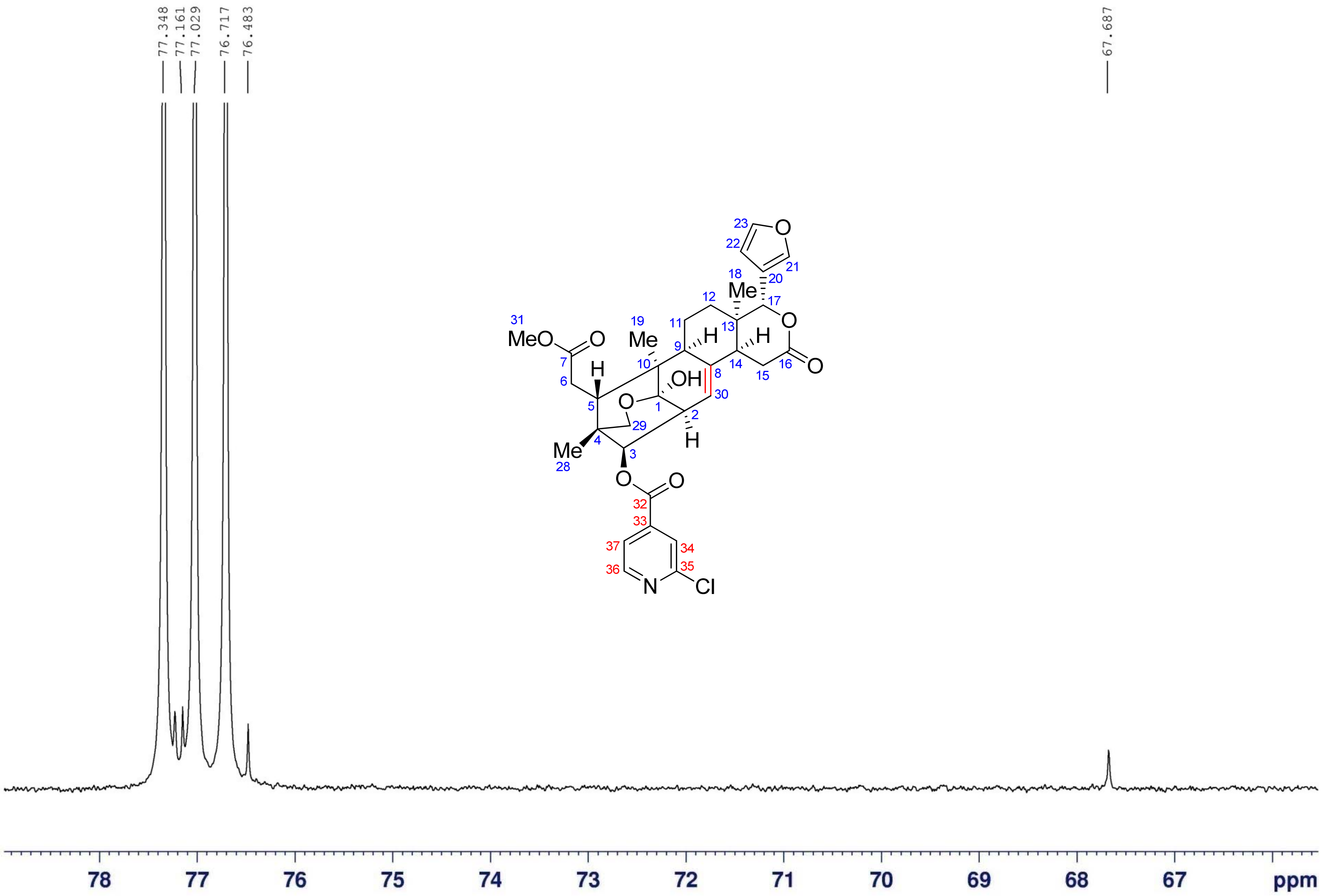

^{13}C NMR (100 MHz) spectrum of **8h** in $CDCl_3$

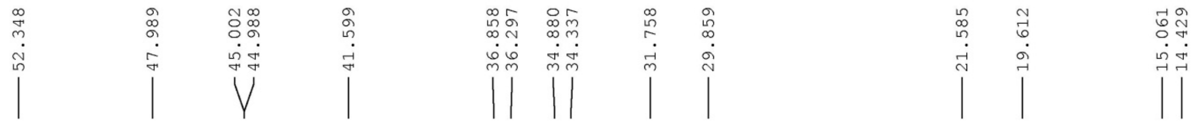

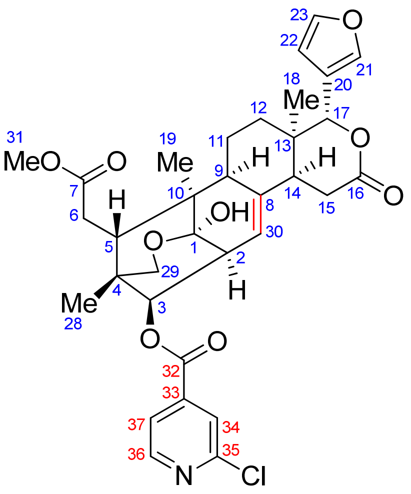

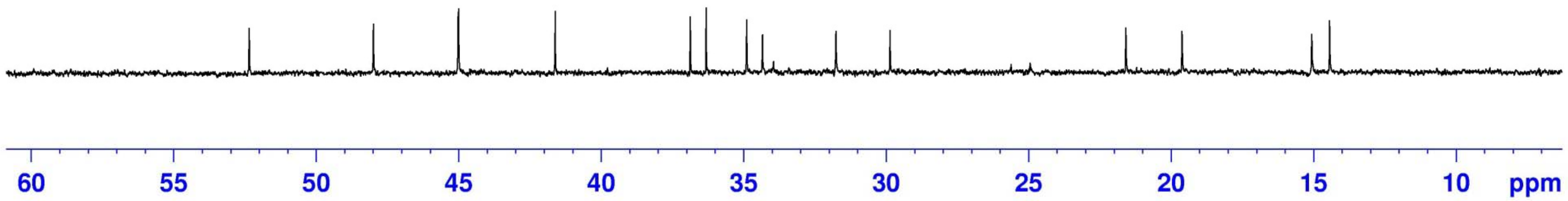

DEPT 135 spectrum of **8h** in $CDCl_3$

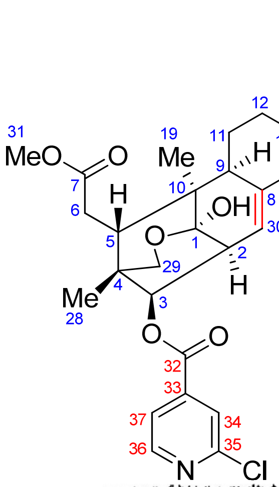

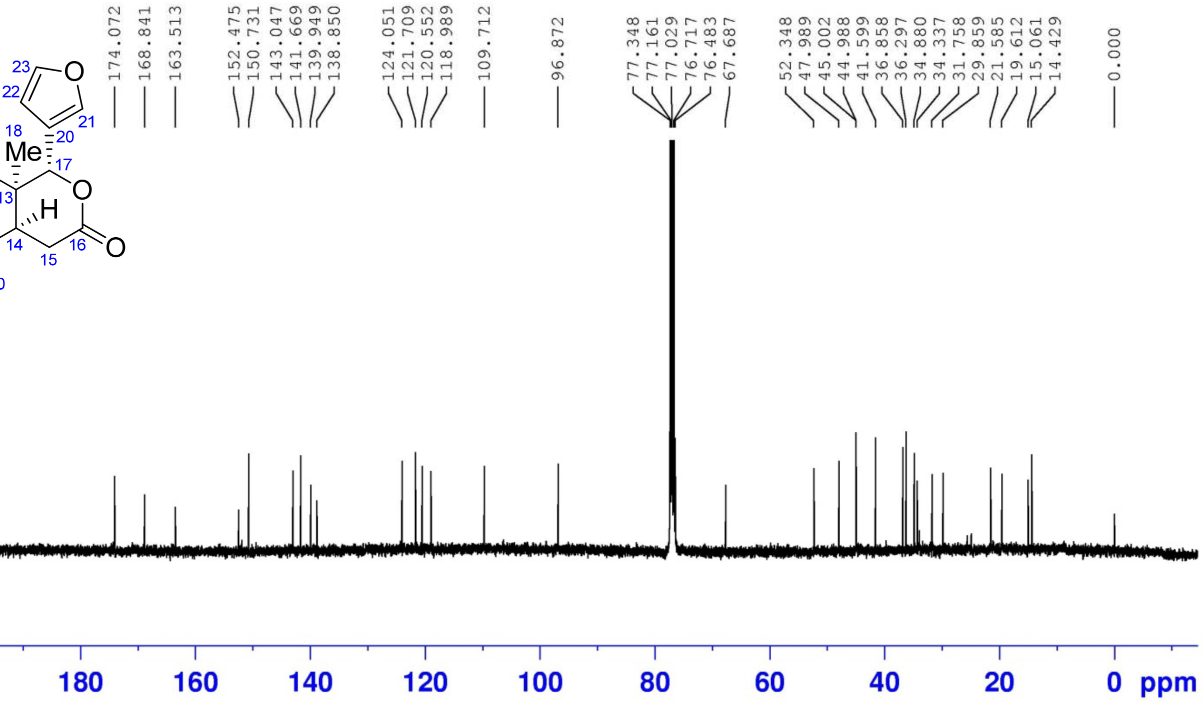

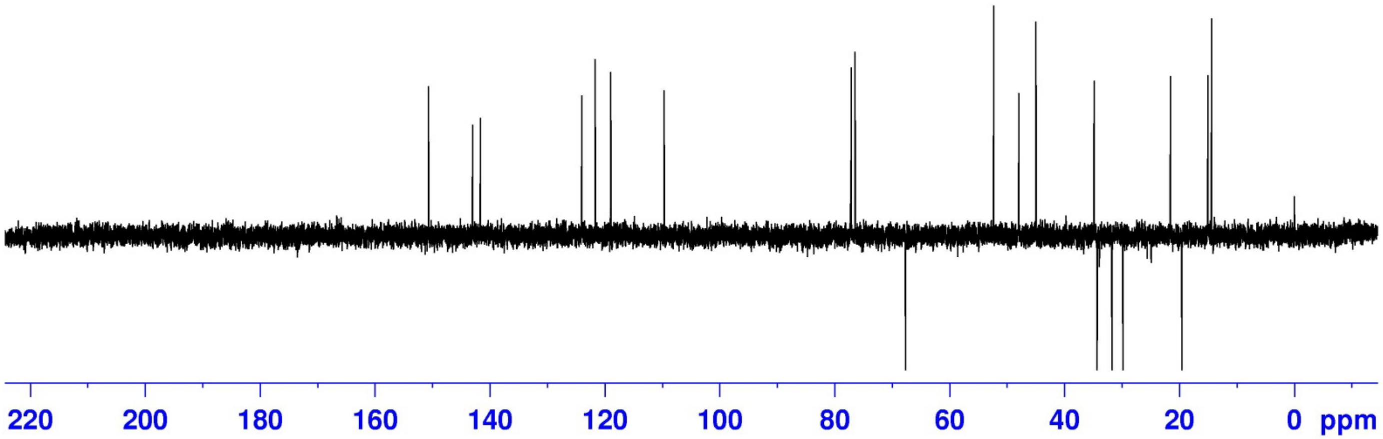

NAME	czc-1-CH3-2-Cl-yiya	
EXPNO	3	
PROCNO	1	
Date_	20171016	
Time	21.31	
INSTRUM	spect	
PROBHD	5 mm CPPBBO BB	
PULPROG	deptsp135	
TD	65536	
SOLVENT	CDCl3	
NS	300	
DS	4	
SWH	24038.461	Hz
FIDRES	0.366798	Hz
AQ	1.3631988	sec
RG	130.26	
DW	20.800	usec
DE	18.00	usec
TE	297.0	K
CNST2	145.0000000	
D1	2.00000000	sec
D2	0.00344828	sec
D12	0.00002000	sec
TD0	1	
======== CHANNEL f1 ========		
SFO1	100.6233324	MHz
NUC1	13C	
P1	10.00	usec
P13	2000.00	usec
SI	32768	
SF	100.6127691	MHz
WDW	EM	
SSB	0	
LB	1.00	Hz
GB	0	
PC	1.40	

DEPT 135 spectrum of **8h** in $CDCl_3$

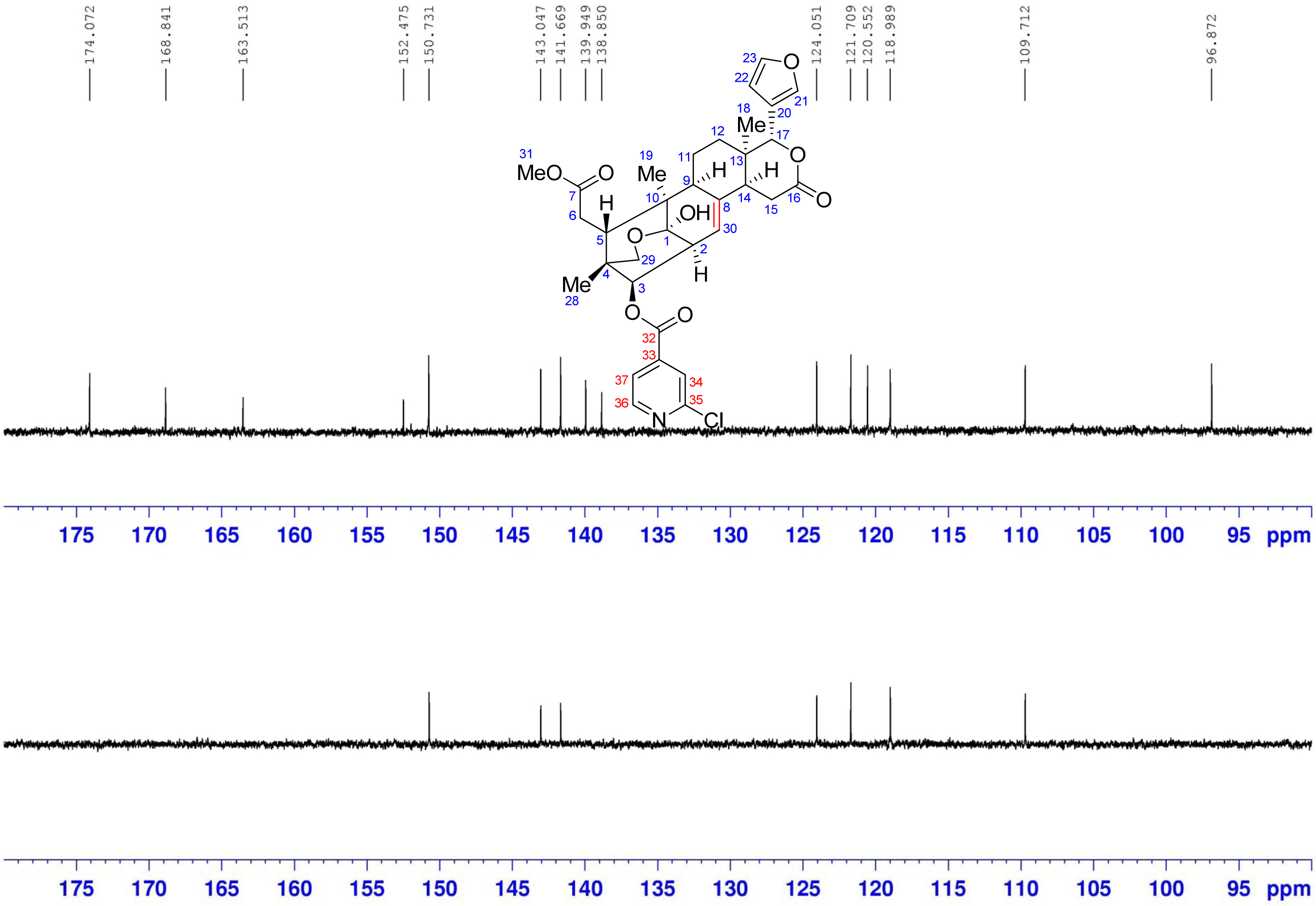

DEPT 135 spectrum of **8h** in $CDCl_3$

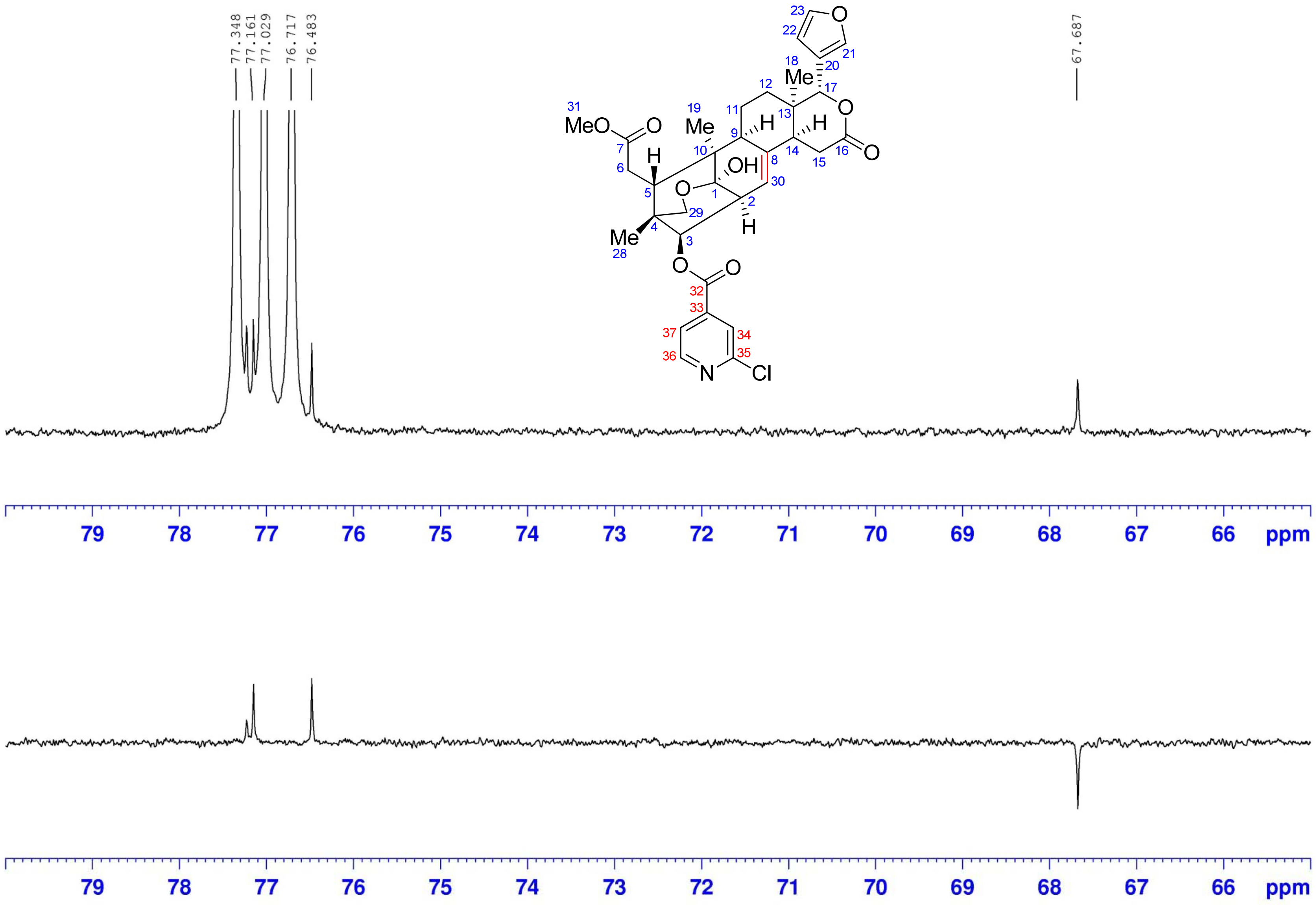

DEPT 135 spectrum of **8h** in $CDCl_3$

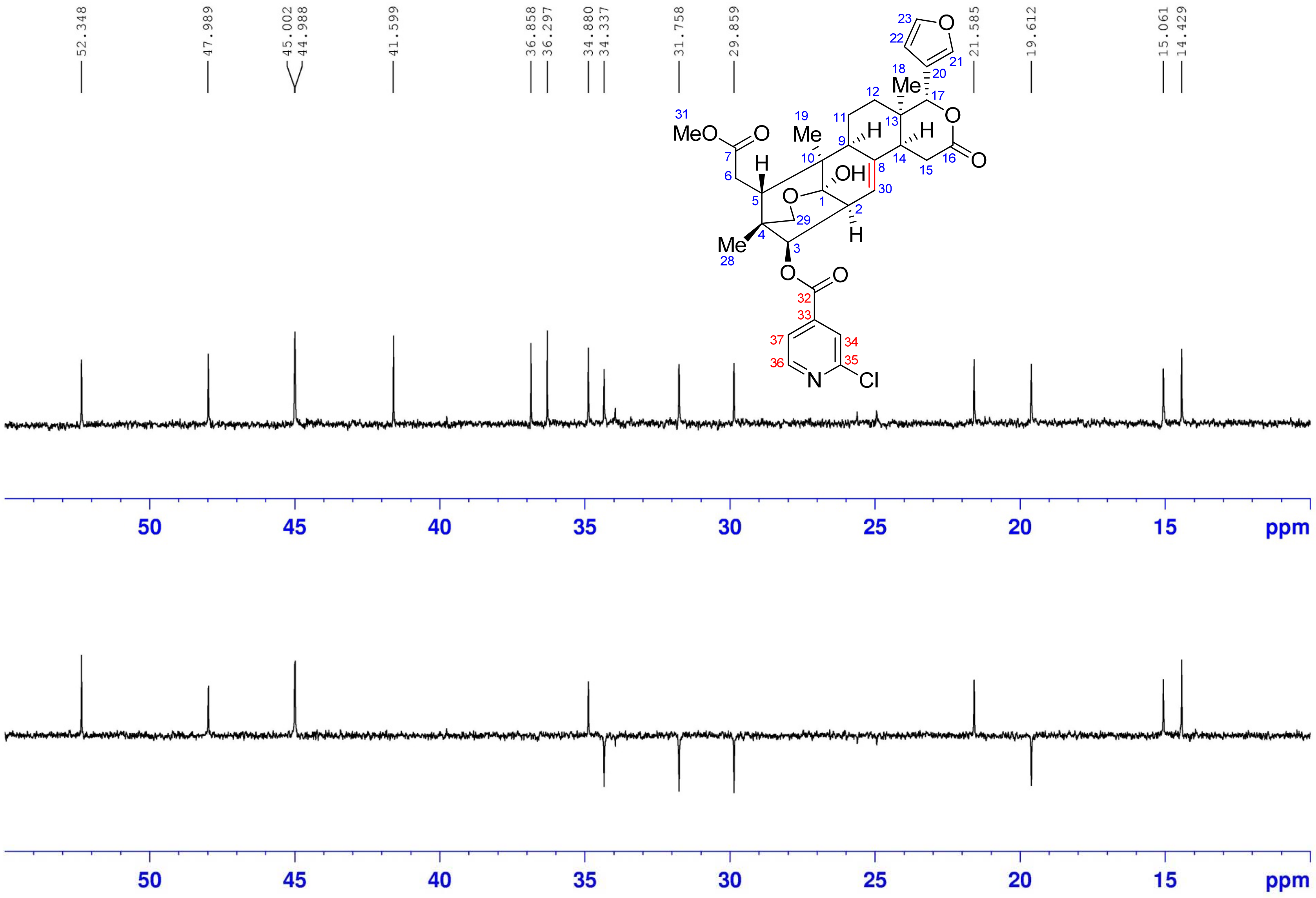

HR-ESIMS for compound **8i**

Generic Display Report

**Analysis Info**		Acquisition Date	9/30/2018 6:26:21 PM
Analysis Name	D:\Data\MS\data\201809\liwanshan_WSD-75_pos_20_01_5581.d		
Method	LC_Direct Infusion_pos_100-1000mz.m	Operator	SCSIO
Sample Name	liwanshan_WSD-75_pos	Instrument	maXis
Comment			

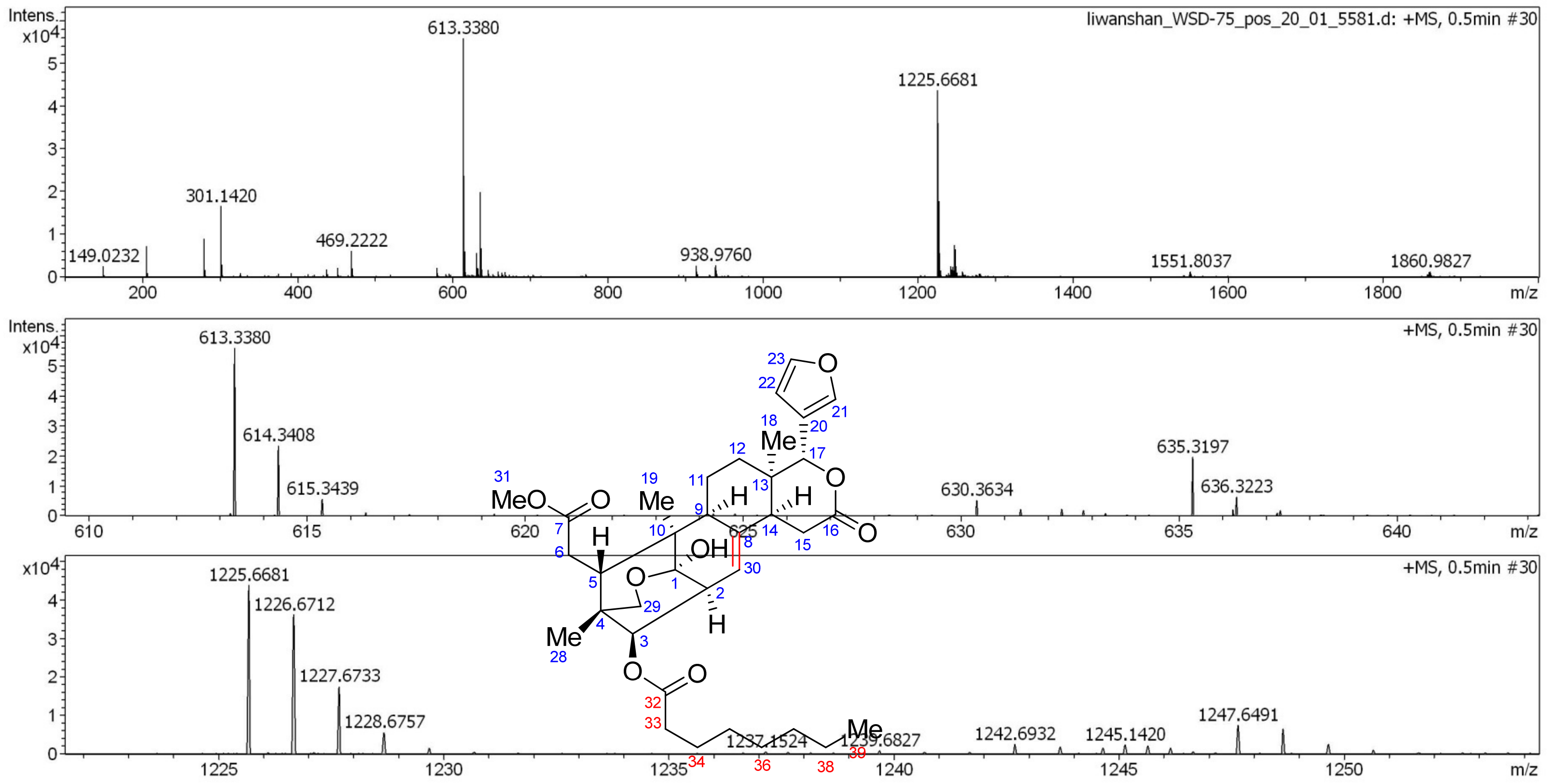

HR-ESIMS for compound **8i**

Mass Spectrum SmartFormula Report

Analysis Info

Analysis Name	D:\Data\MS\data\201809\liwanshan_WSD-75_pos_20_01_5581.d	Acquisition Date	9/30/2018 6:26:21 PM
Method	LC_Direct Infusion_pos_100-1000mz.m	Operator	SCSIO
Sample Name	liwanshan_WSD-75_pos	Instrument	maXis 255552.00029
Comment			

Acquisition Parameter

Source Type	ESI	Ion Polarity	Positive	Set Nebulizer	0.4 Bar
Focus	Active	Set Capillary	4500 V	Set Dry Heater	180 °C
Scan Begin	100 m/z	Set End Plate Offset	-500 V	Set Dry Gas	4.0 l/min
Scan End	2000 m/z	Set Charging Voltage	0 V	Set Divert Valve	Waste
		Set Corona	0 nA	Set APCI Heater	0 °C

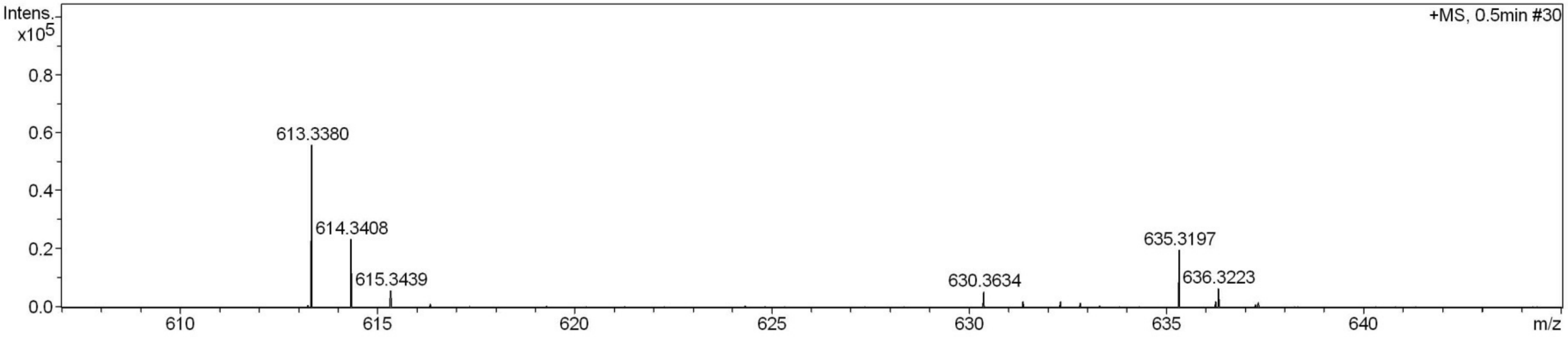

Meas. m/z	#	Ion Formula	Score	m/z	err [ppm]	err [mDa]	mSigma	rdb	e⁻ Conf	N-Rule
613.3380	1	C35H49O9	100.00	613.3371	-1.5	-0.9	19.6	11.5	even	ok
635.3197	1	C35H48NaO9	100.00	635.3191	-1.0	-0.6	25.4	11.5	even	ok
1225.6681	1	C70H97O18	100.00	1225.6669	-1.0	-1.2	36.0	22.5	even	ok
1247.6491	1	C70H96NaO18	100.00	1247.6489	-0.2	-0.3	39.1	22.5	even	ok

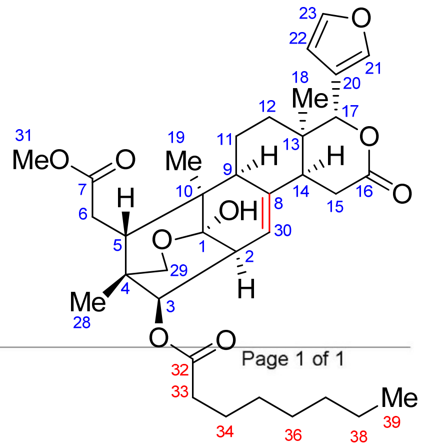

^{1}H NMR (400 MHz) spectrum of **8i** in $CDCl_3$

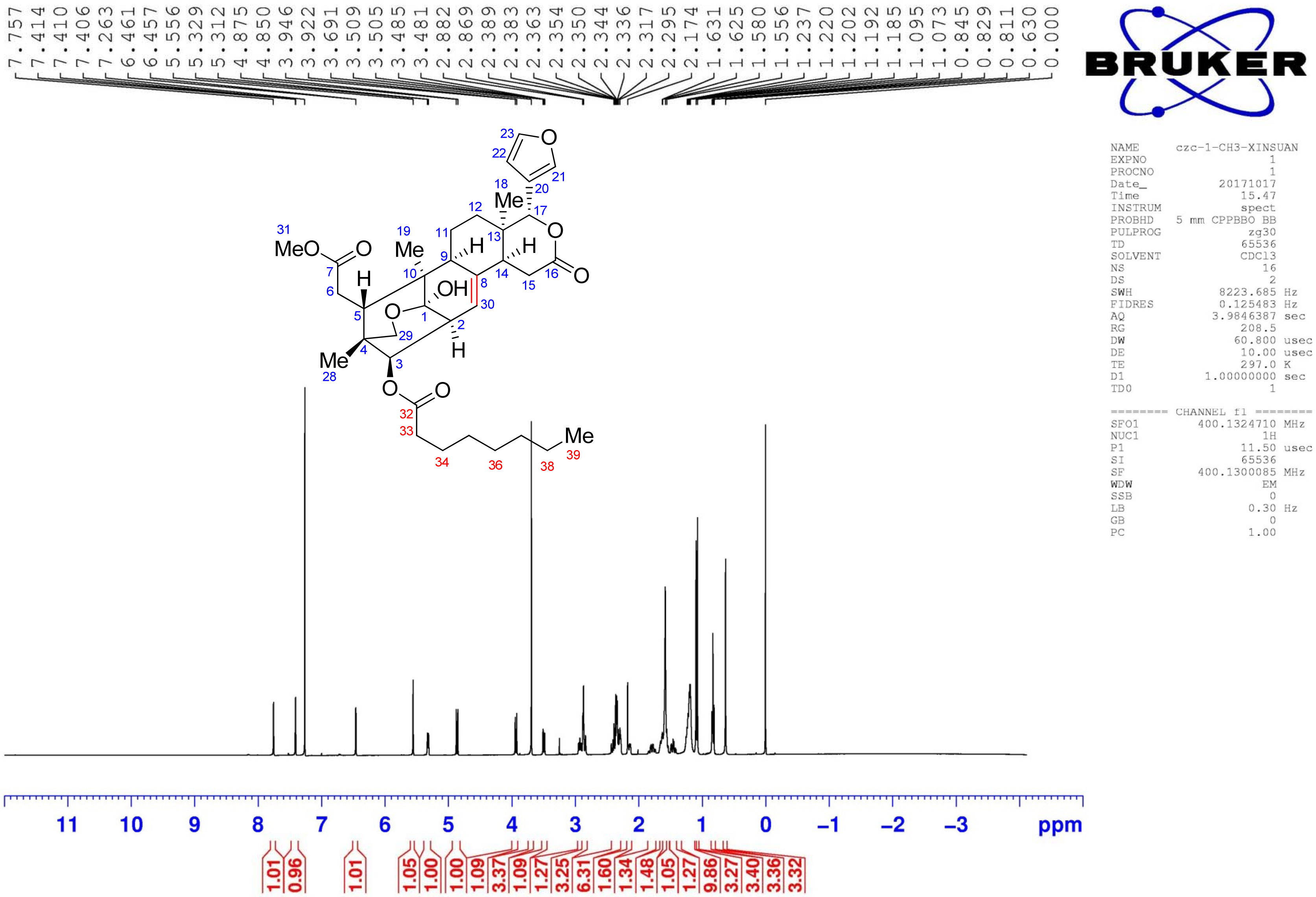

^{1}H NMR (400 MHz) spectrum of **8i** in $CDCl_3$

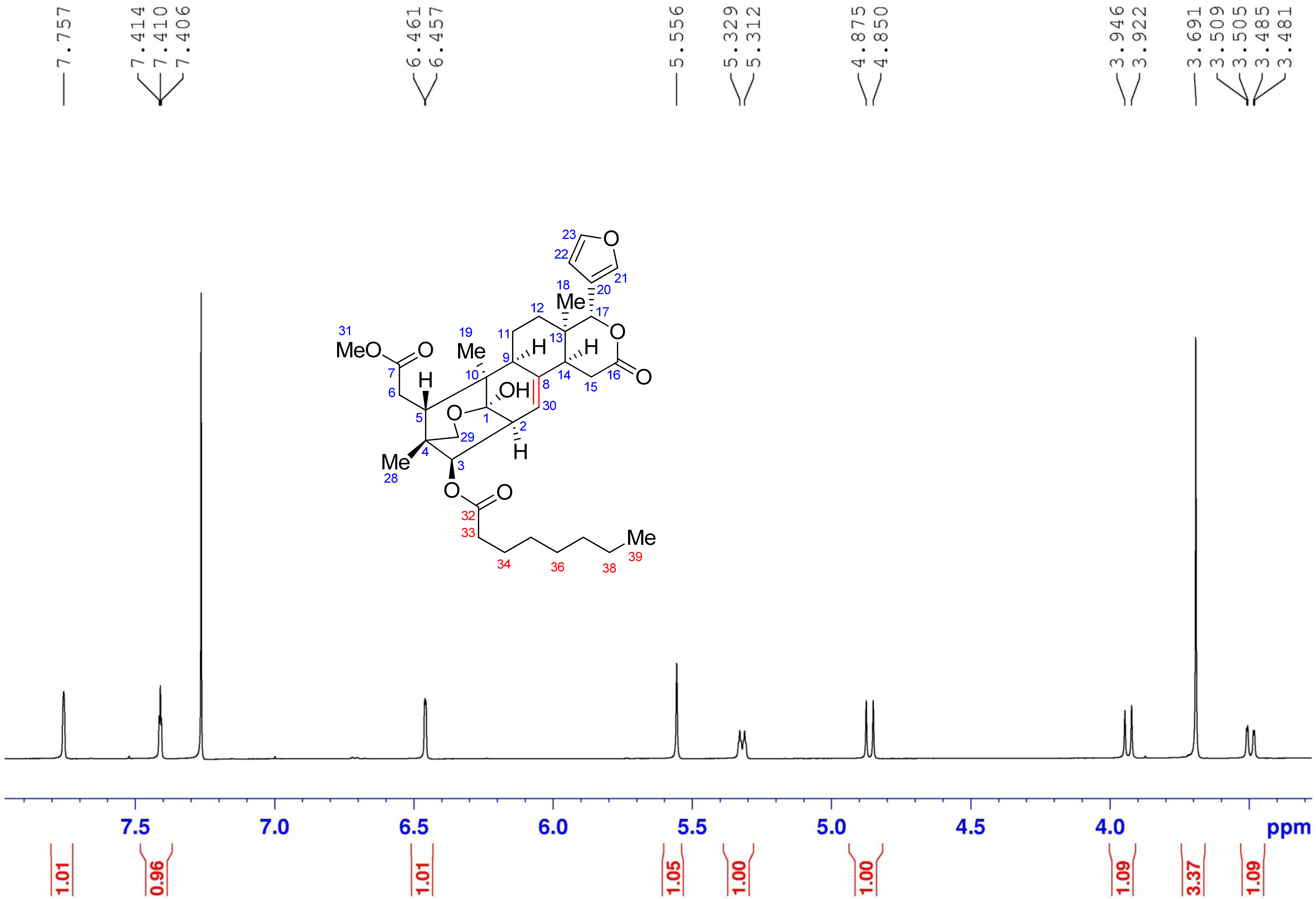

^{1}H NMR (400 MHz) spectrum of **8i** in $CDCl_3$

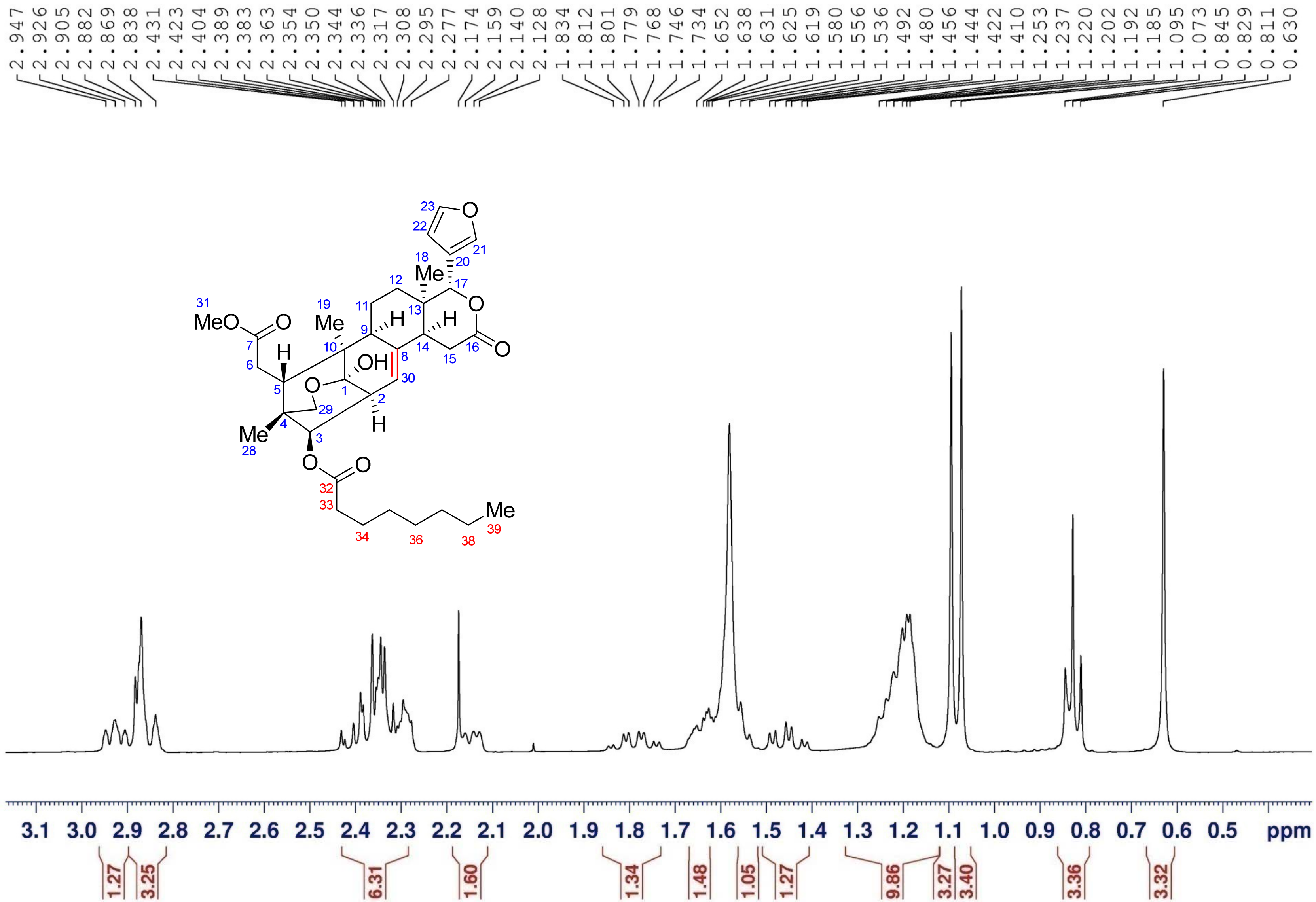

^{13}C NMR (100 MHz) spectrum of **8i** in $CDCl_3$

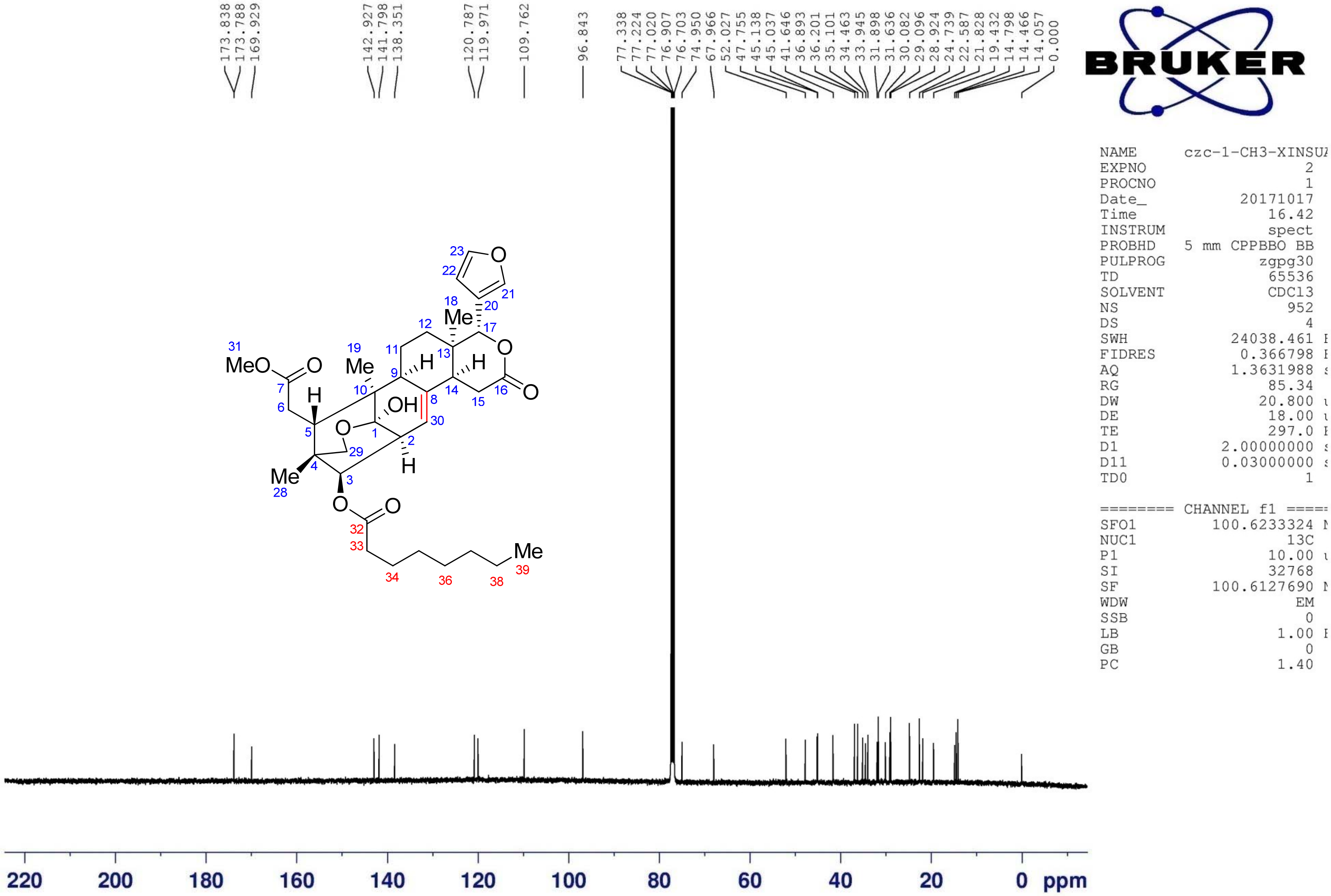


```
NAME        czc-1-CH3-XINSUA
EXPNO                      2
PROCNO                     1
Date_               20171017
Time                   16.42
INSTRUM                spect
PROBHD     5 mm CPPBBO BB
PULPROG              zgpg30
TD                     65536
SOLVENT                CDCl3
NS                       952
DS                         4
SWH                24038.461 H
FIDRES              0.366798 H
AQ                 1.3631988 s
RG                     85.34
DW                    20.800 u
DE                     18.00 u
TE                     297.0 K
D1                2.00000000 s
D11               0.03000000 s
TD0                        1

======== CHANNEL f1 =====
SFO1             100.6233324 M
NUC1                     13C
P1                     10.00 u
SI                     32768
SF               100.6127690 M
WDW                       EM
SSB                        0
LB                      1.00 H
GB                         0
PC                      1.40
```

^{13}C NMR (100 MHz) spectrum of **8i** in $CDCl_3$

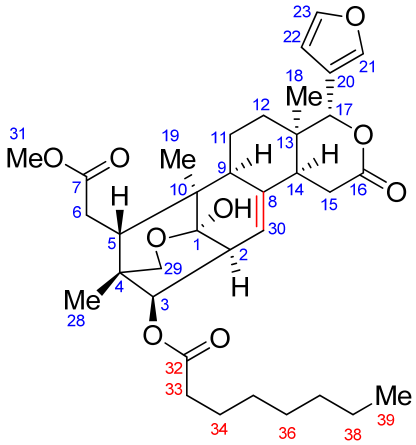

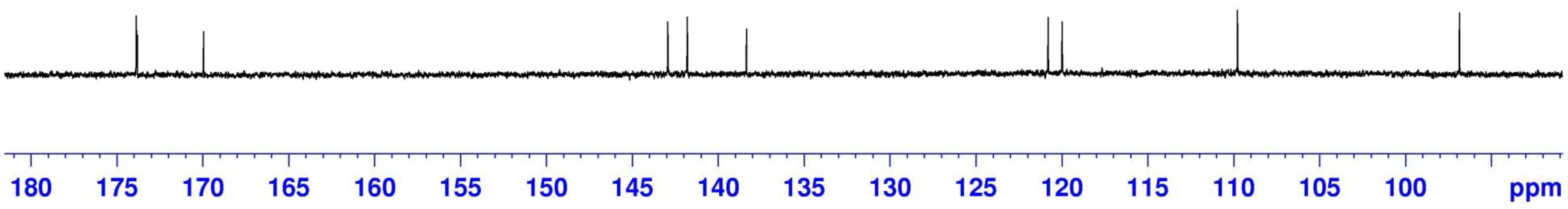

^{13}C NMR (100 MHz) spectrum of **8i** in $CDCl_3$

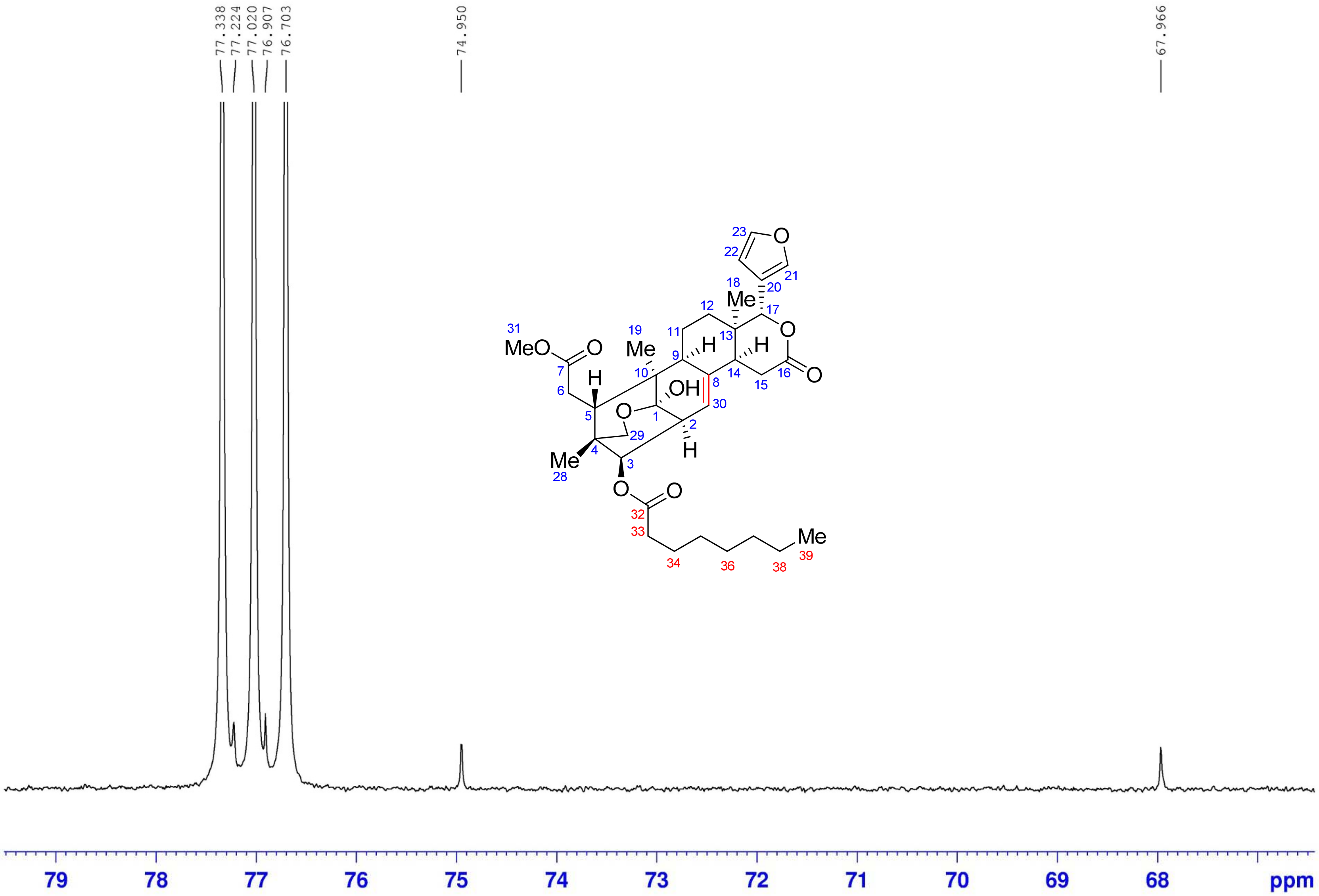

^{13}C NMR (100 MHz) spectrum of **8i** in $CDCl_3$

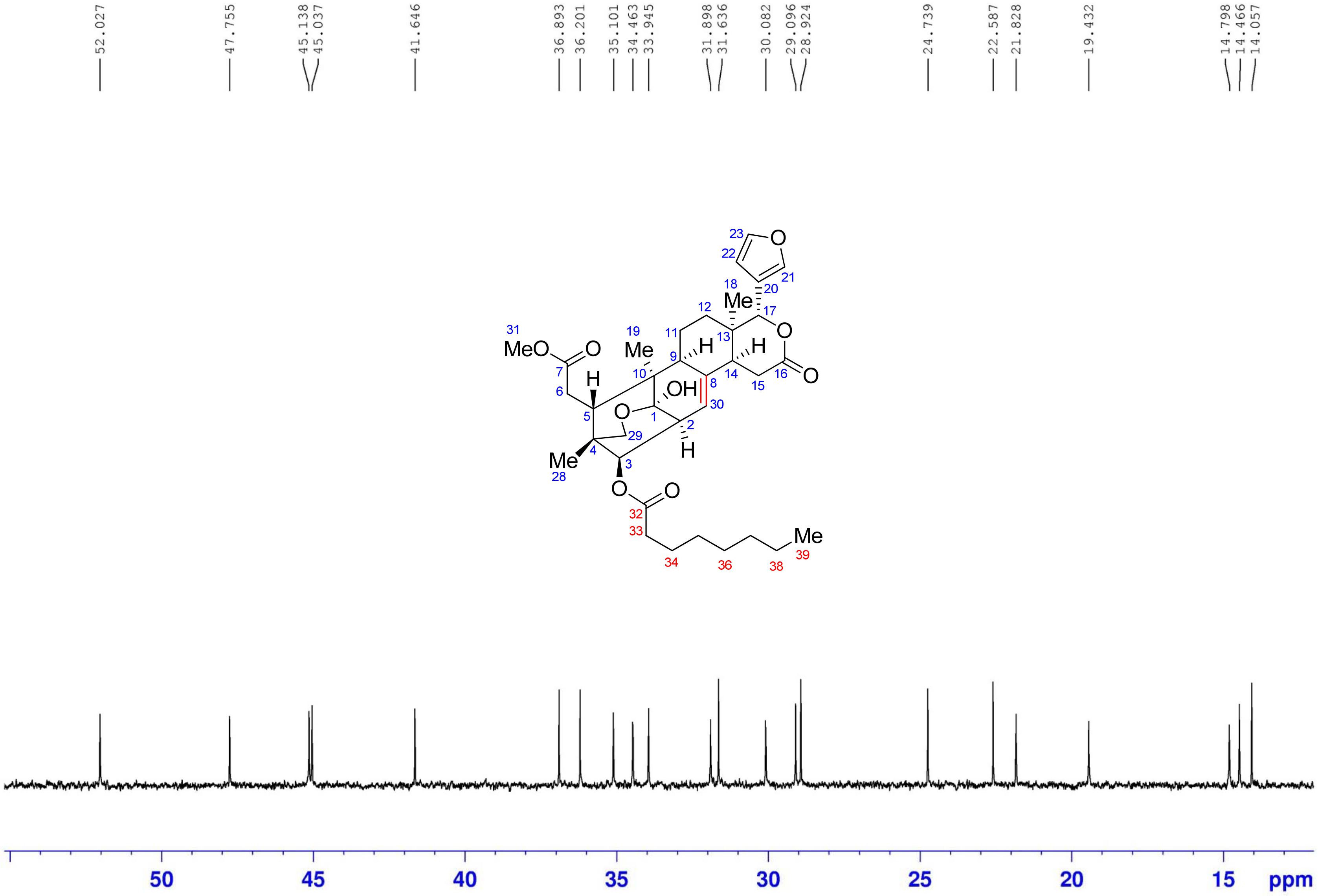

DEPT 135 spectrum of **8i** in $CDCl_3$

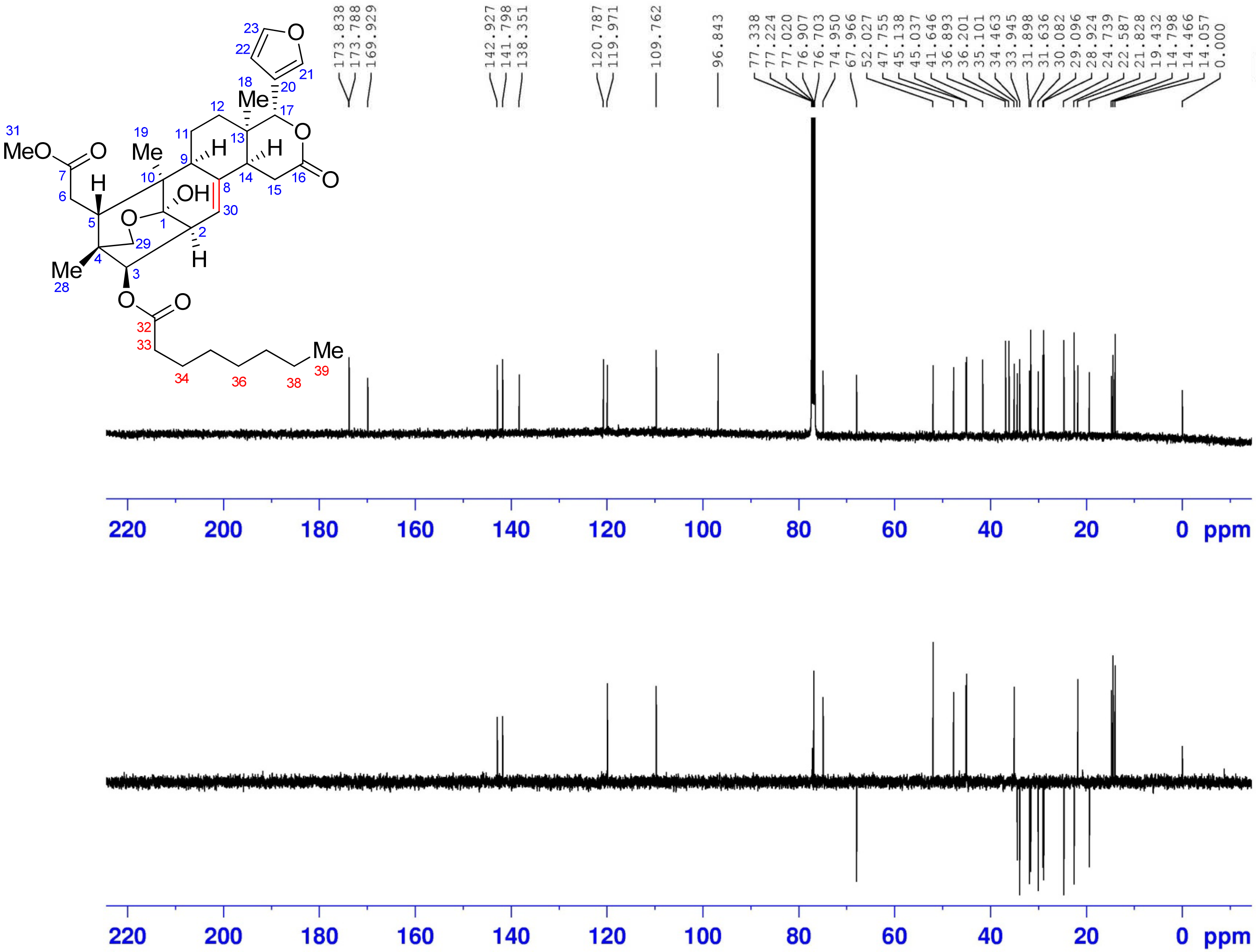

NAME	czc-1-CH3-XINSU
EXPNO	3
PROCNO	1
Date_	20171017
Time	16.44
INSTRUM	spect
PROBHD	5 mm CPPBBO BB
PULPROG	deptsp135
TD	65536
SOLVENT	CDCl3
NS	300
DS	4
SWH	24038.461
FIDRES	0.366798
AQ	1.3631988
RG	85.34
DW	20.800
DE	18.00
TE	297.0
CNST2	145.0000000
D1	2.00000000
D2	0.00344828
D12	0.00002000
TD0	1
======== CHANNEL f1 ====	
SFO1	100.6233324
NUC1	13C
P1	10.00
P13	2000.00
SI	32768
SF	100.6127690
WDW	EM
SSB	0
LB	1.00
GB	0
PC	1.40

DEPT 135 spectrum of **8i** in $CDCl_3$

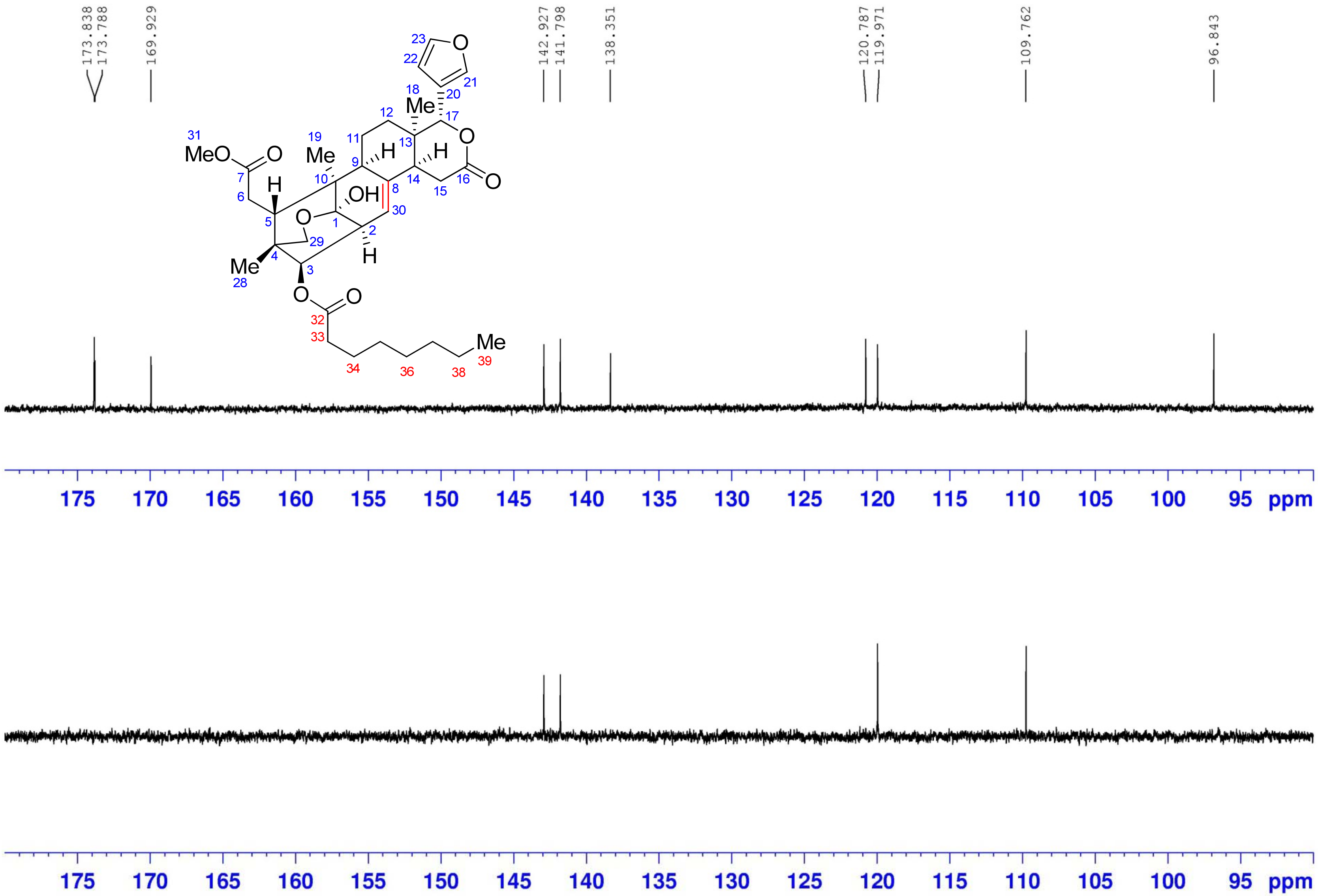

DEPT 135 spectrum of **8i** in $CDCl_3$

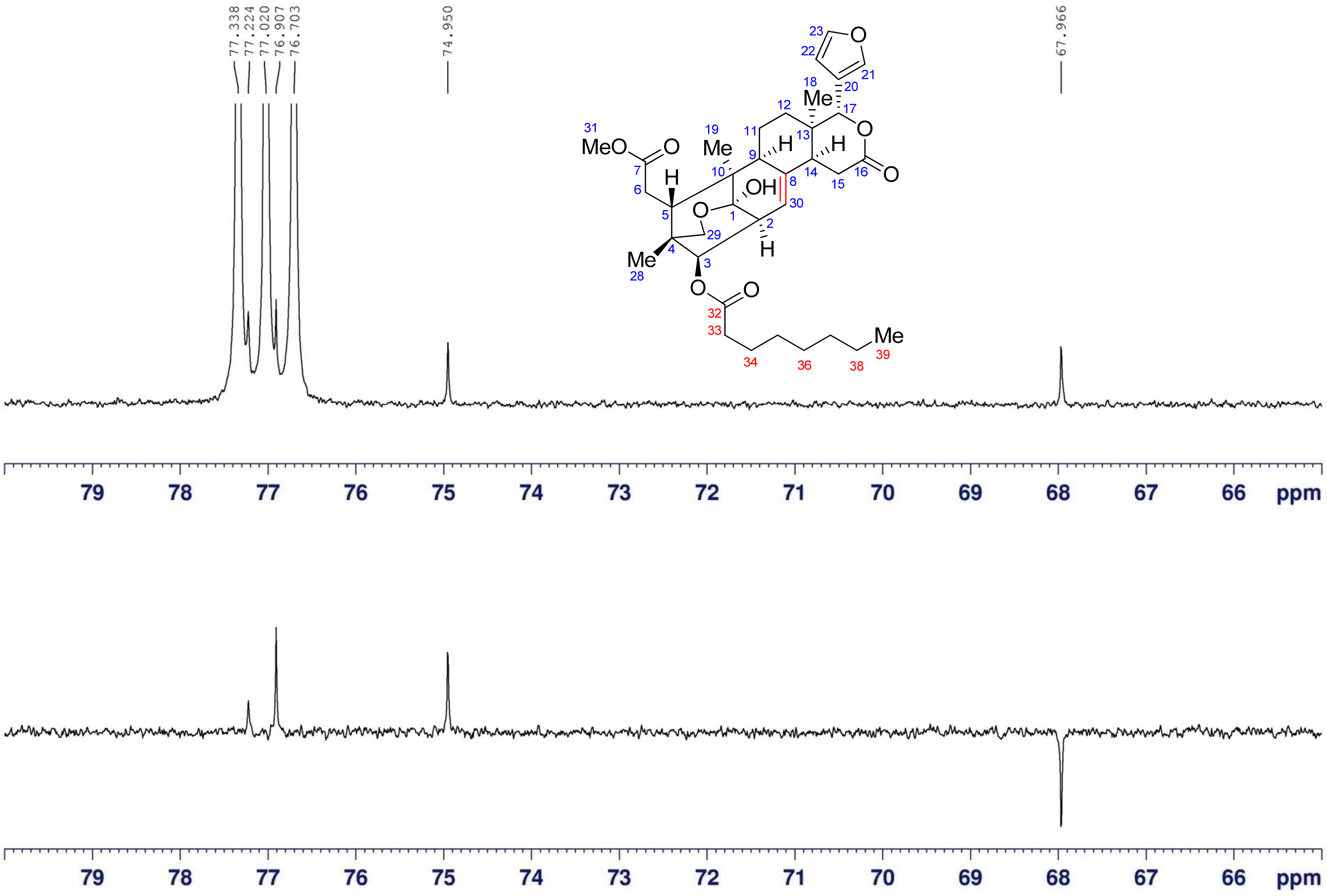

DEPT 135 spectrum of **8i** in $CDCl_3$

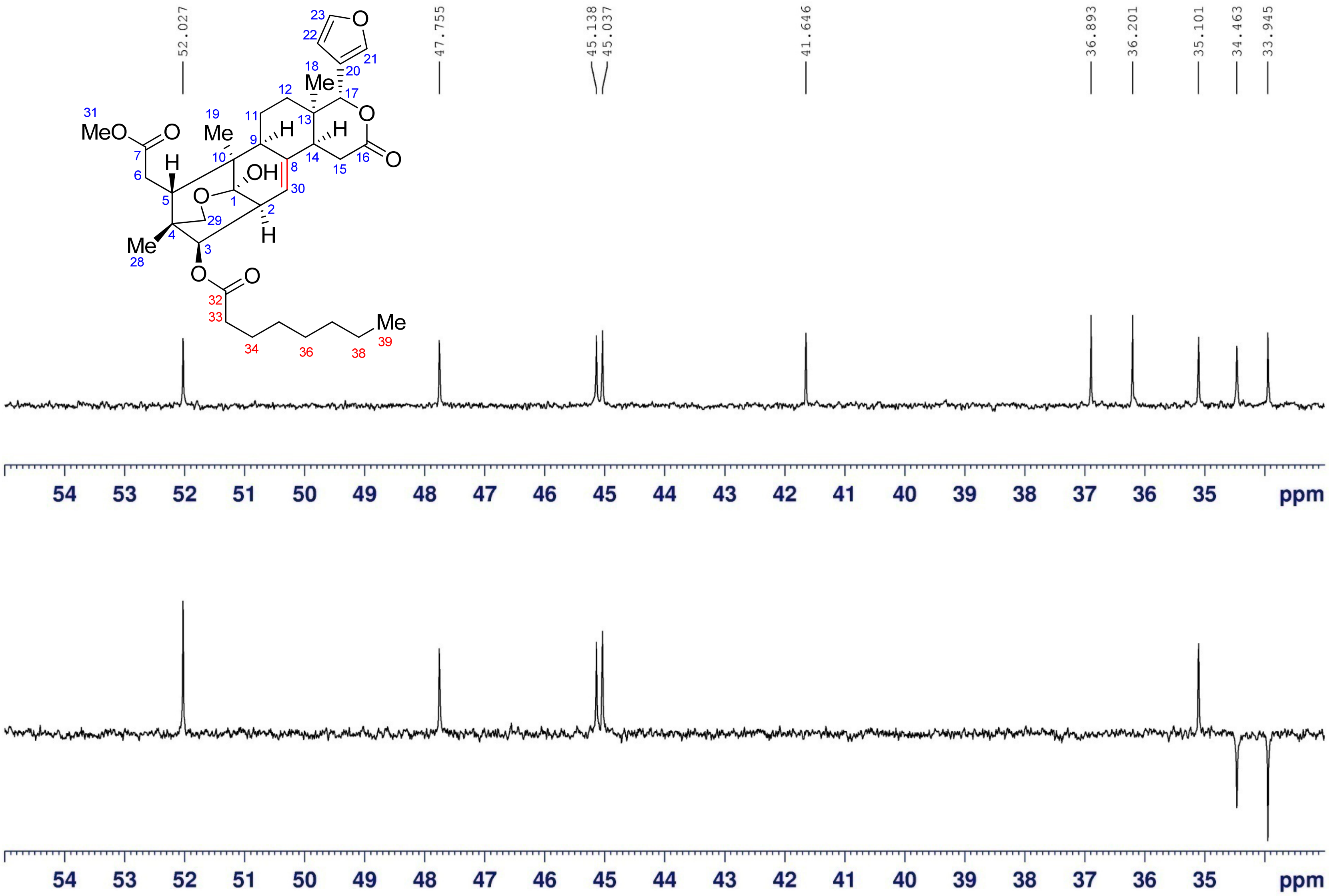

DEPT 135 spectrum of **8i** in $CDCl_3$

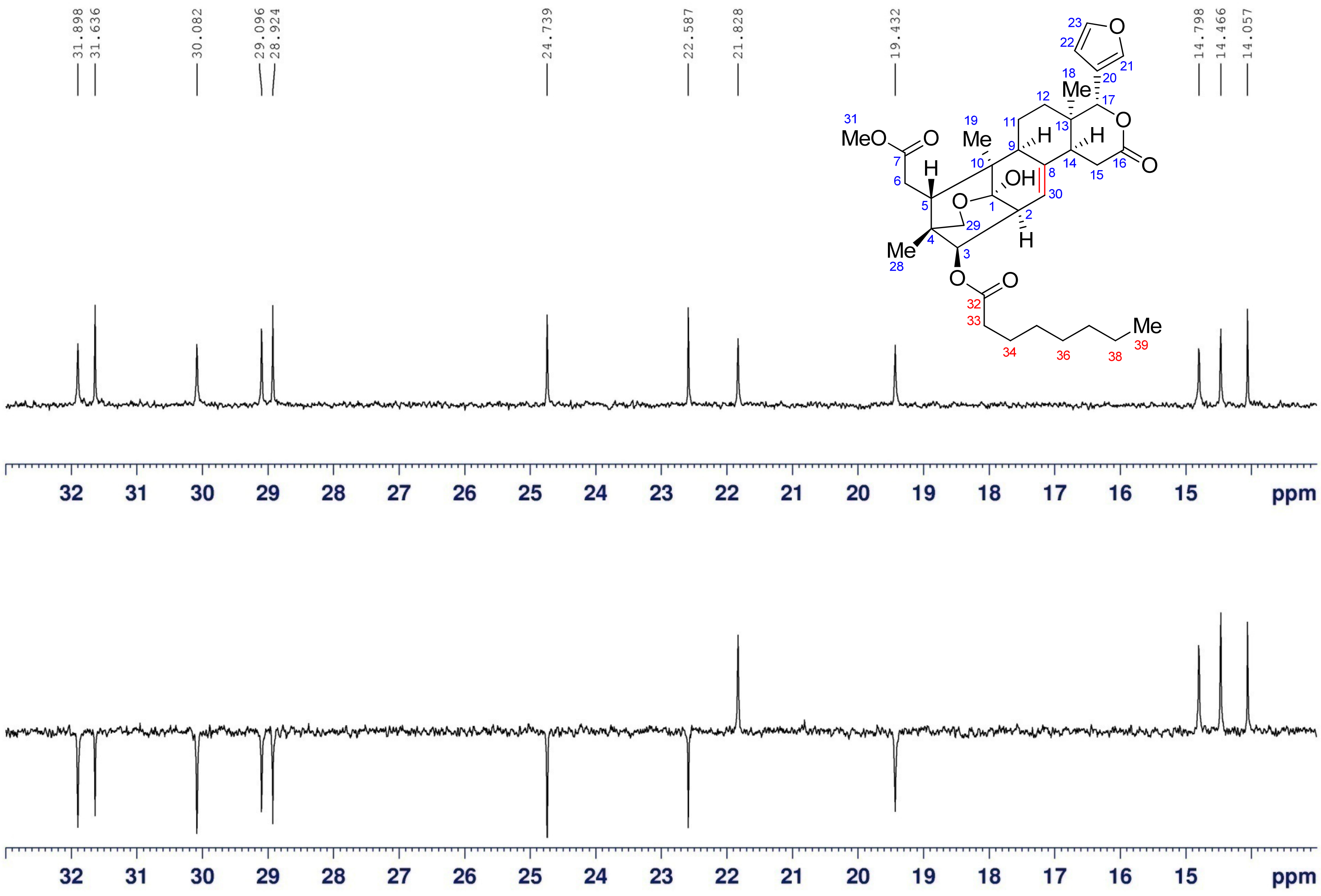

LR-ESIMS for compound **9**

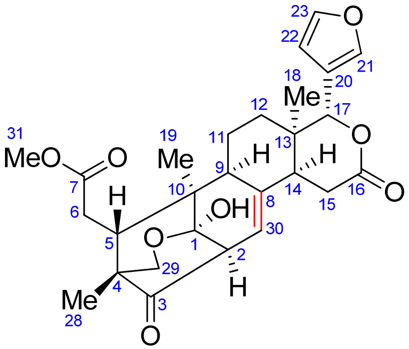

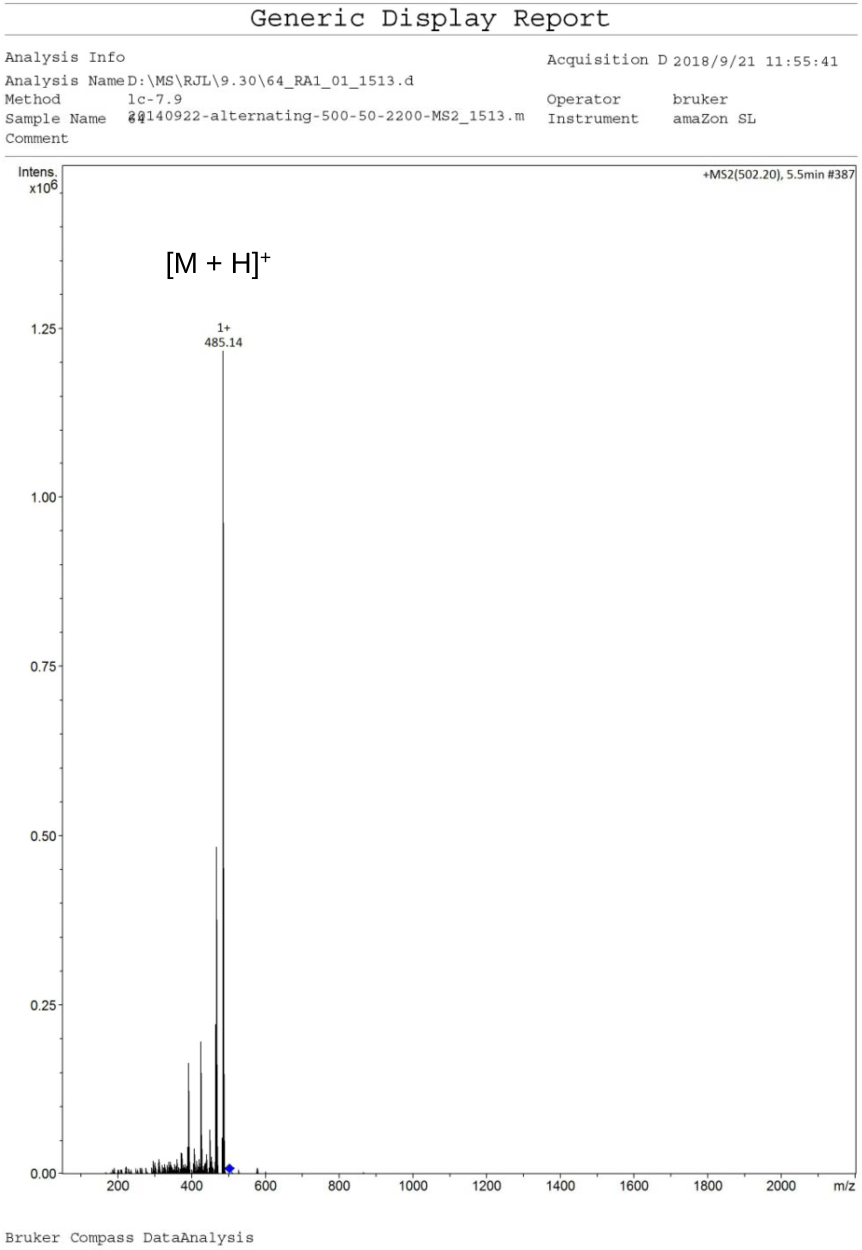

^{1}H NMR (400 MHz) spectrum of **9** in $CDCl_3$

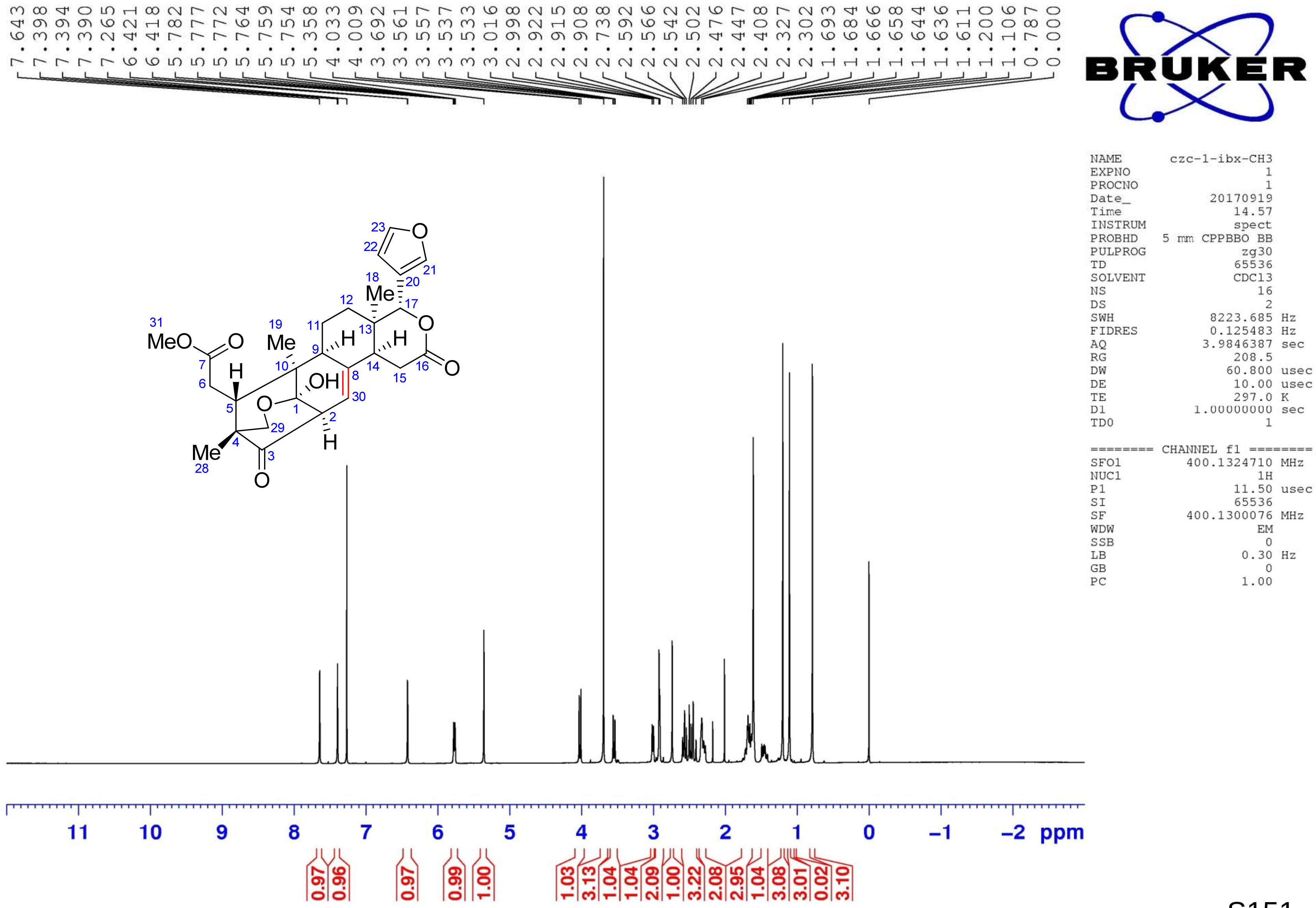

^{1}H NMR (400 MHz) spectrum of **9** in $CDCl_3$

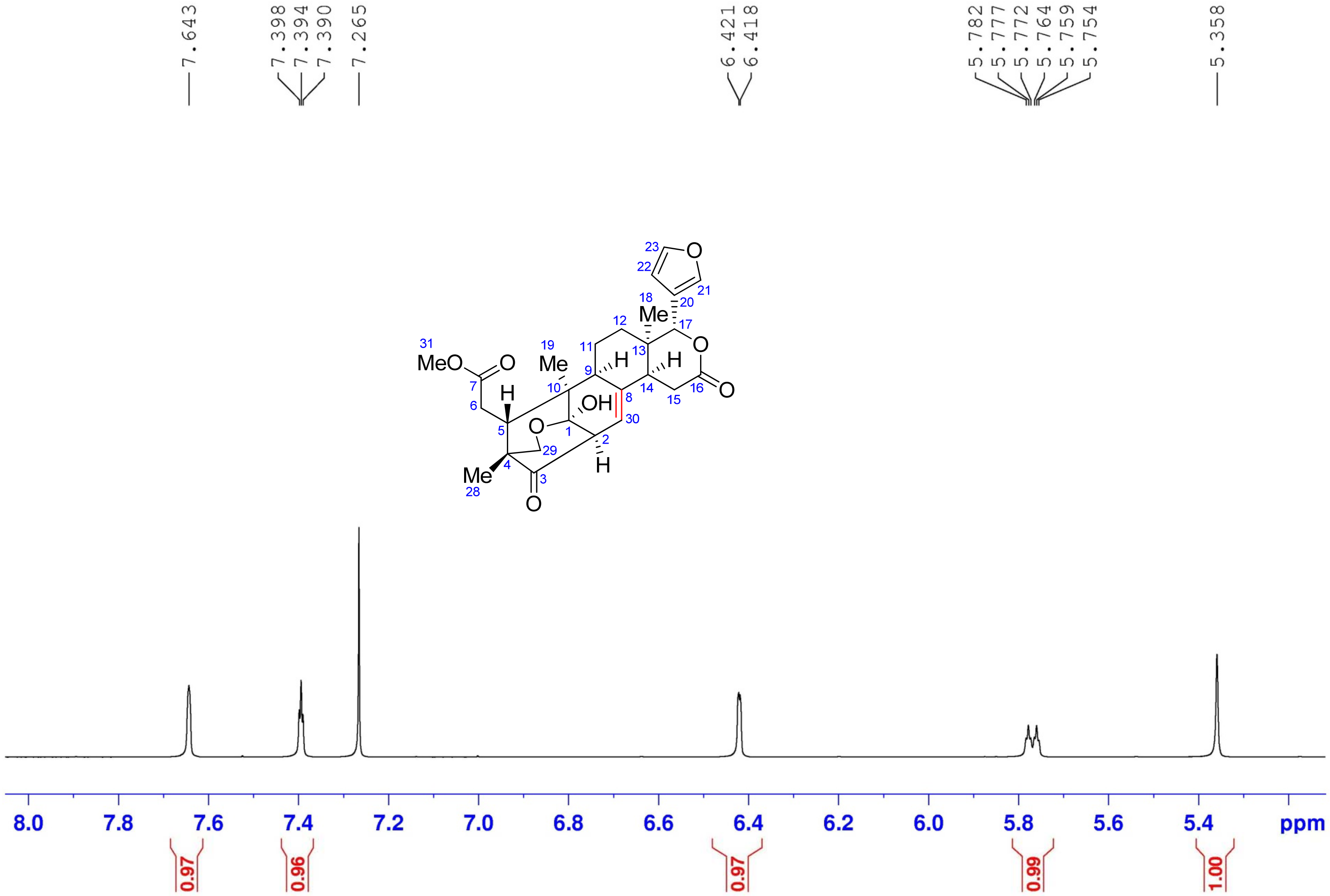

^{1}H NMR (400 MHz) spectrum of **9** in $CDCl_3$

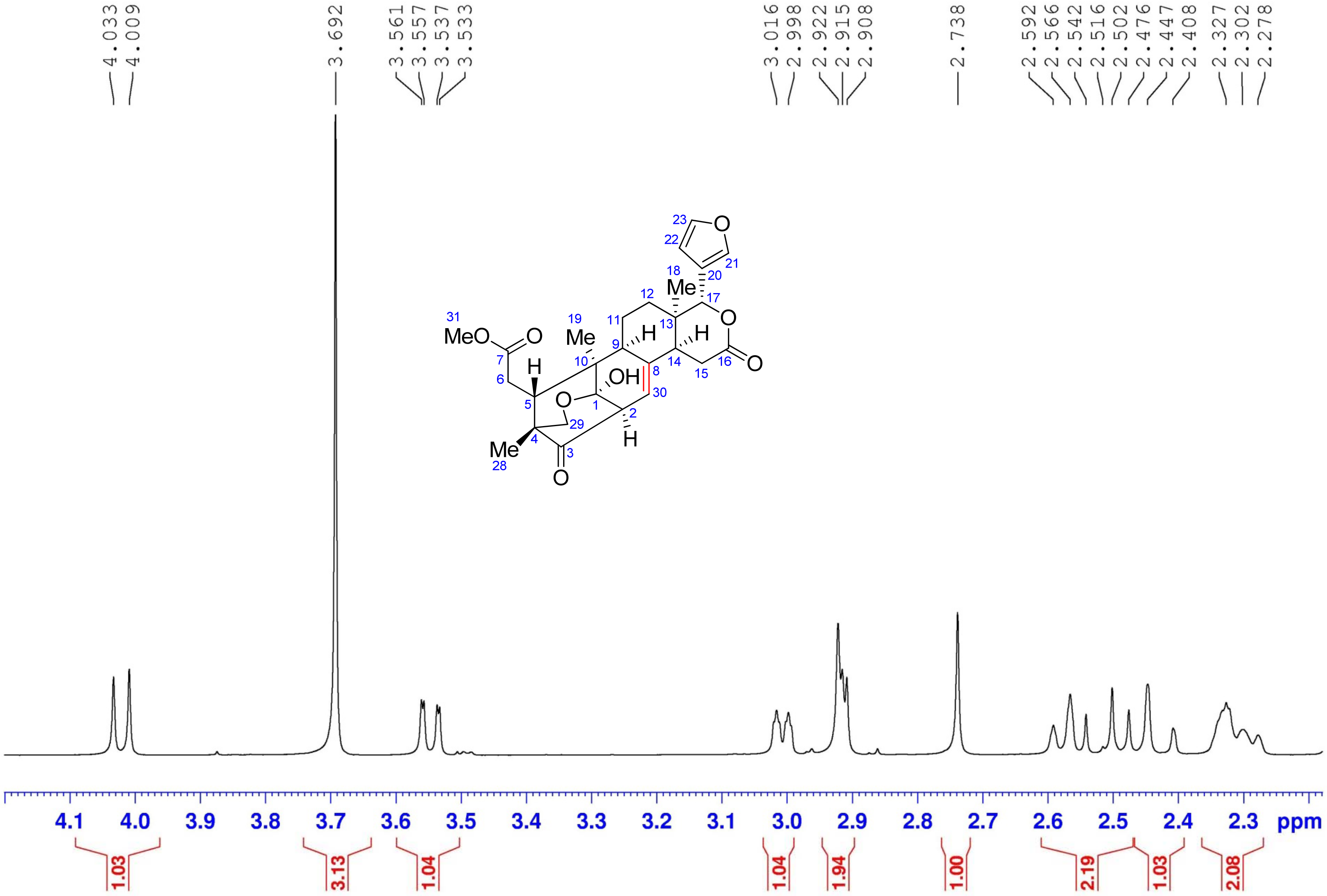

^{1}H NMR (400 MHz) spectrum of **9** in $CDCl_3$

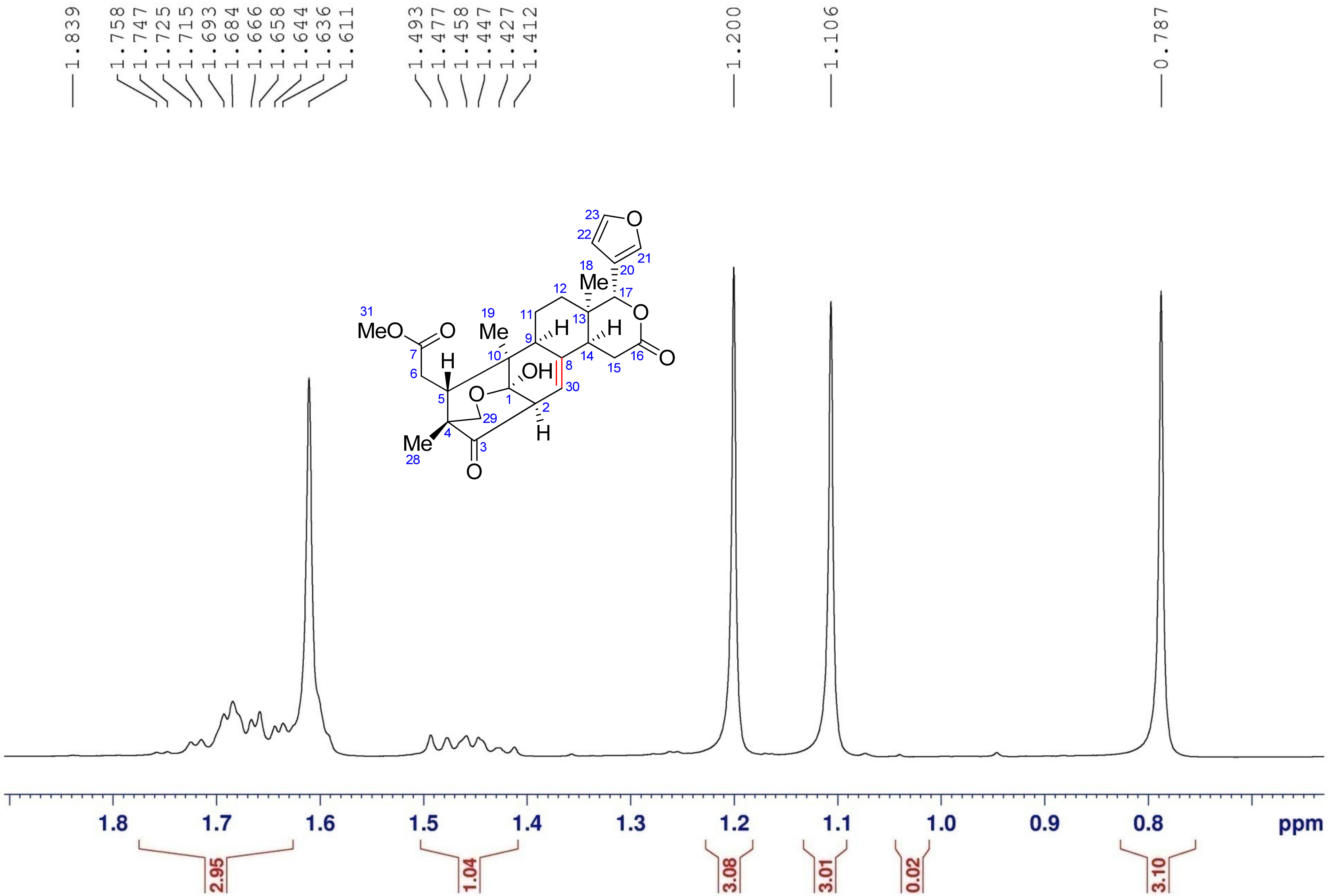

^{13}C NMR (100 MHz) spectrum of **9** in $CDCl_3$

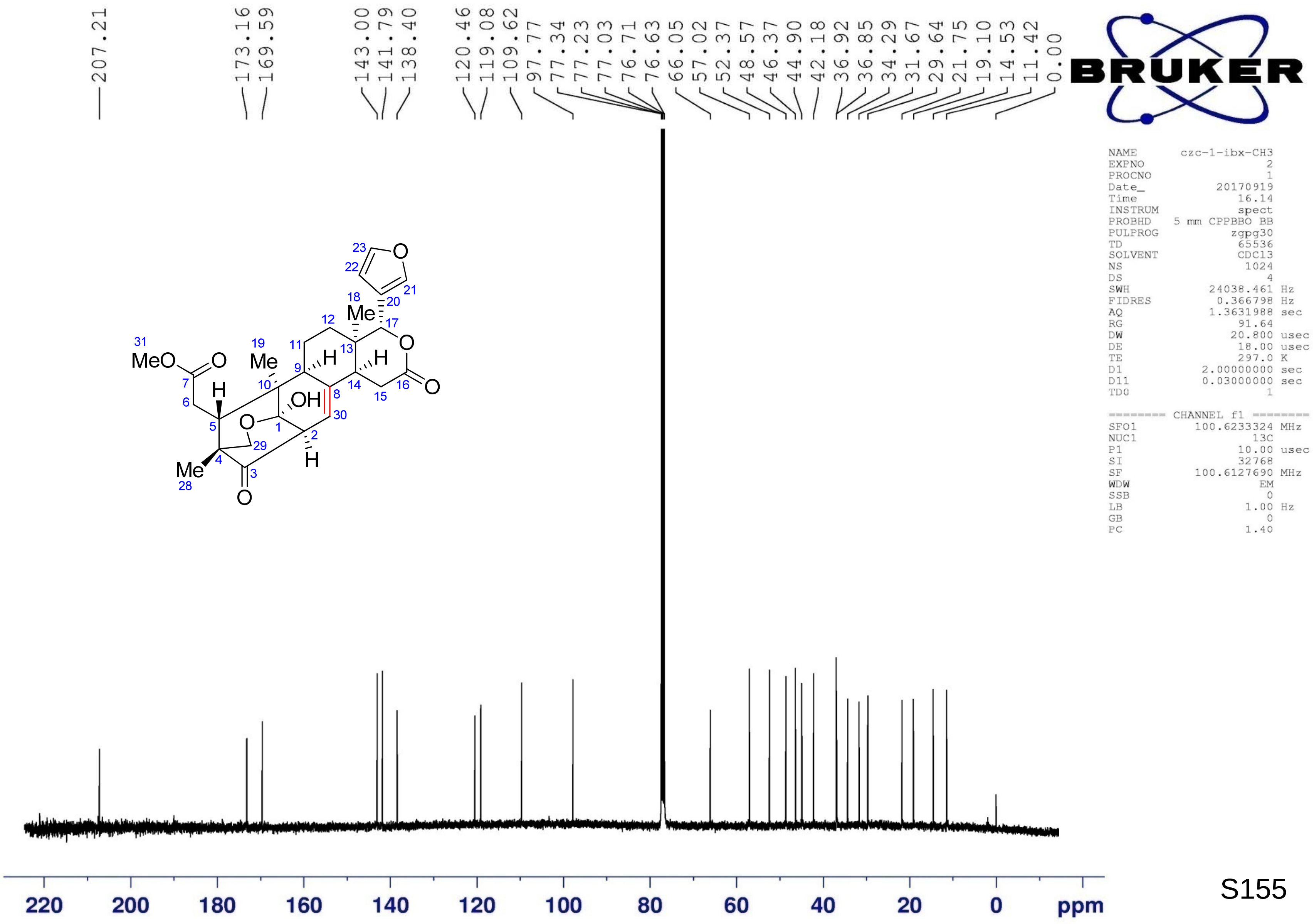

^{13}C NMR (100 MHz) spectrum of **9** in $CDCl_3$

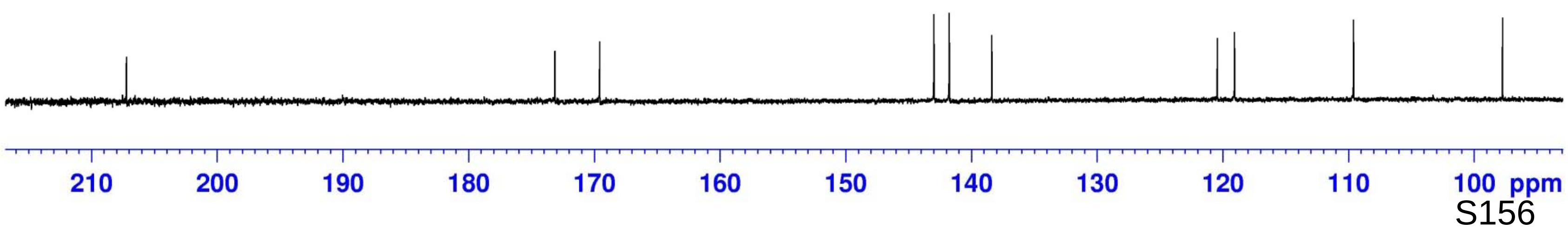

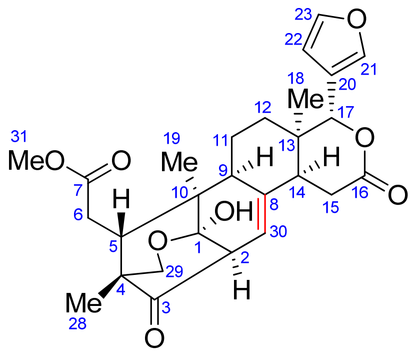

^{13}C NMR (100 MHz) spectrum of **9** in $CDCl_3$

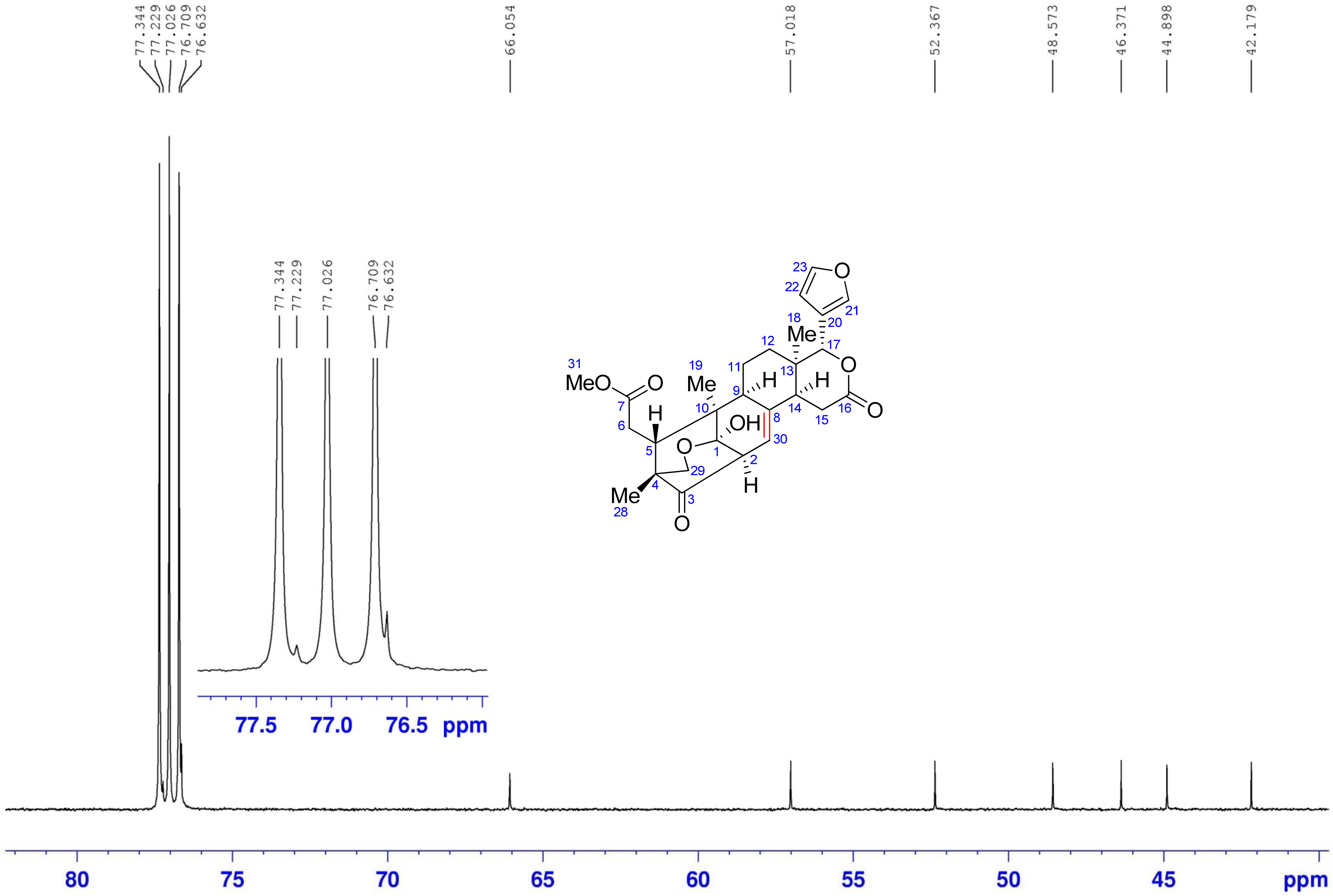

^{13}C NMR (100 MHz) spectrum of **9** in $CDCl_3$

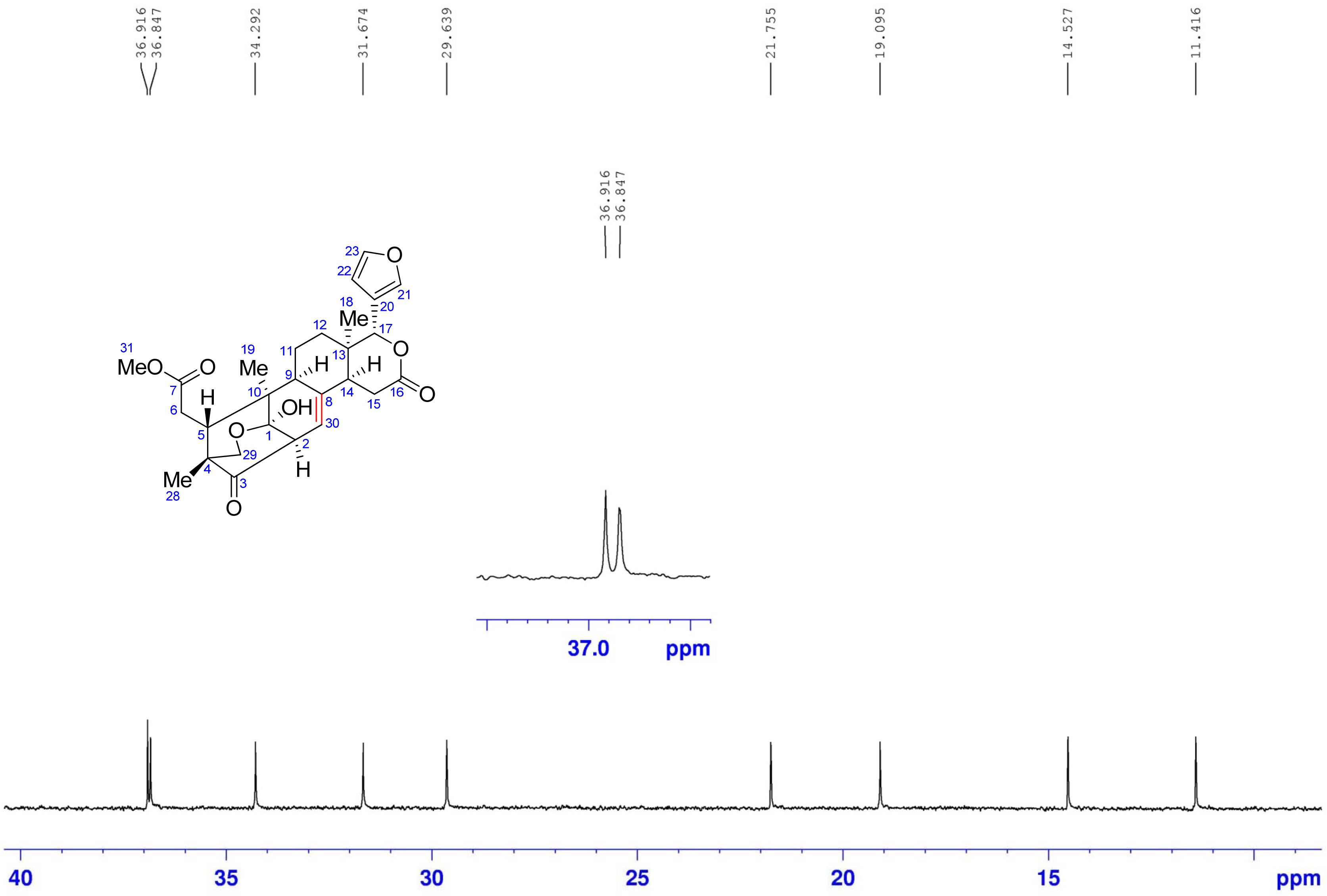

DEPT 135 spectrum of **9** in $CDCl_3$

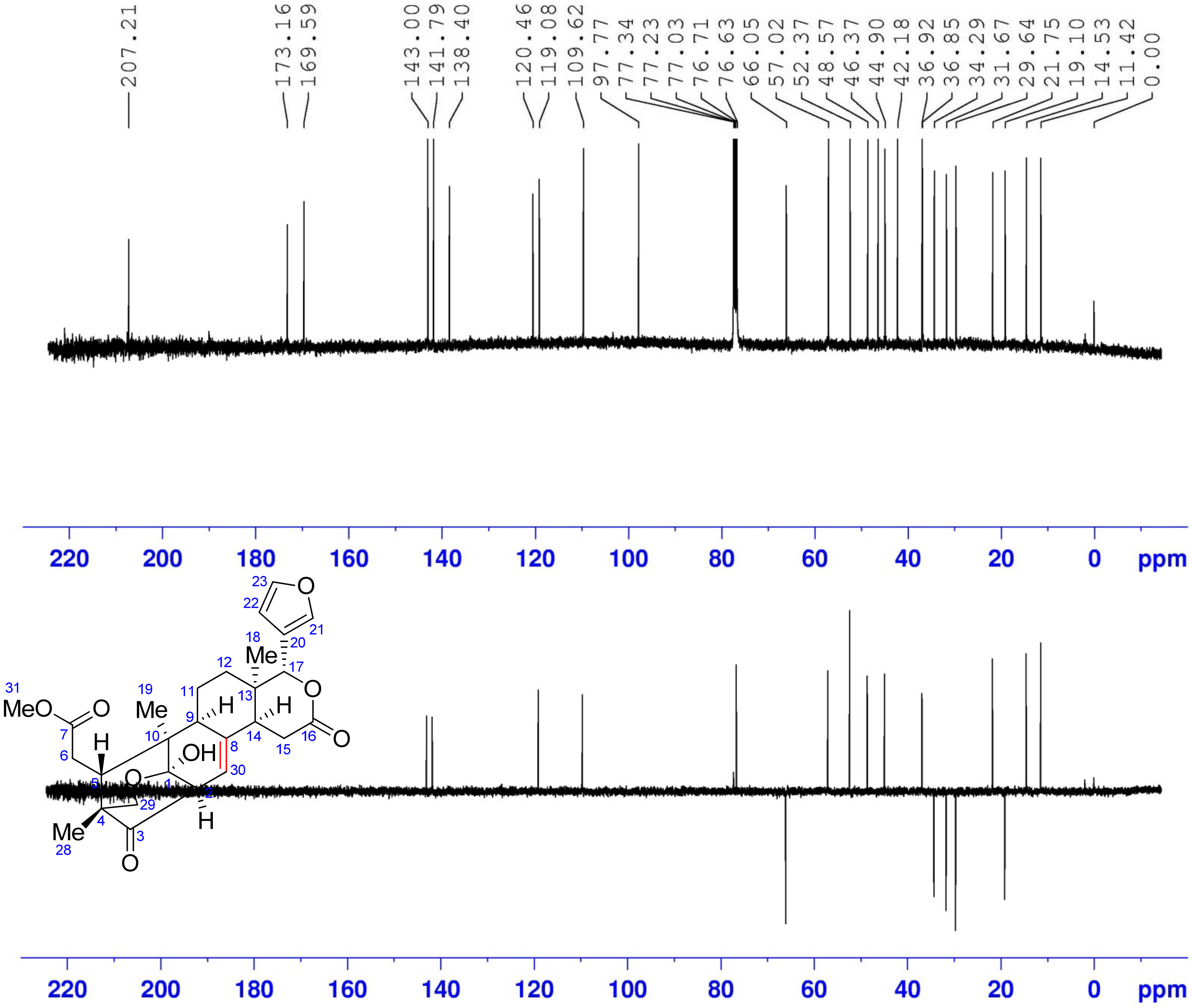

NAME	czc-1-ibx-CH3
EXPNO	3
PROCNO	1
Date_	20170919
Time	16.32
INSTRUM	spect
PROBHD	5 mm CPPBBO BB
PULPROG	deptsp135
TD	65536
SOLVENT	CDCl3
NS	300
DS	4
SWH	24038.461 Hz
FIDRES	0.366798 Hz
AQ	1.3631988 sec
RG	130.26
DW	20.800 usec
DE	18.00 usec
TE	297.0 K
CNST2	145.0000000
D1	2.00000000 sec
D2	0.00344828 sec
D12	0.00002000 sec
TD0	1
======== CHANNEL f1 ========	
SFO1	100.6233324 MHz
NUC1	13C
P1	10.00 usec
P13	2000.00 usec
SI	32768
SF	100.6127692 MHz
WDW	EM
SSB	0
LB	1.00 Hz
GB	0
PC	1.40

DEPT 135 spectrum of **9** in $CDCl_3$

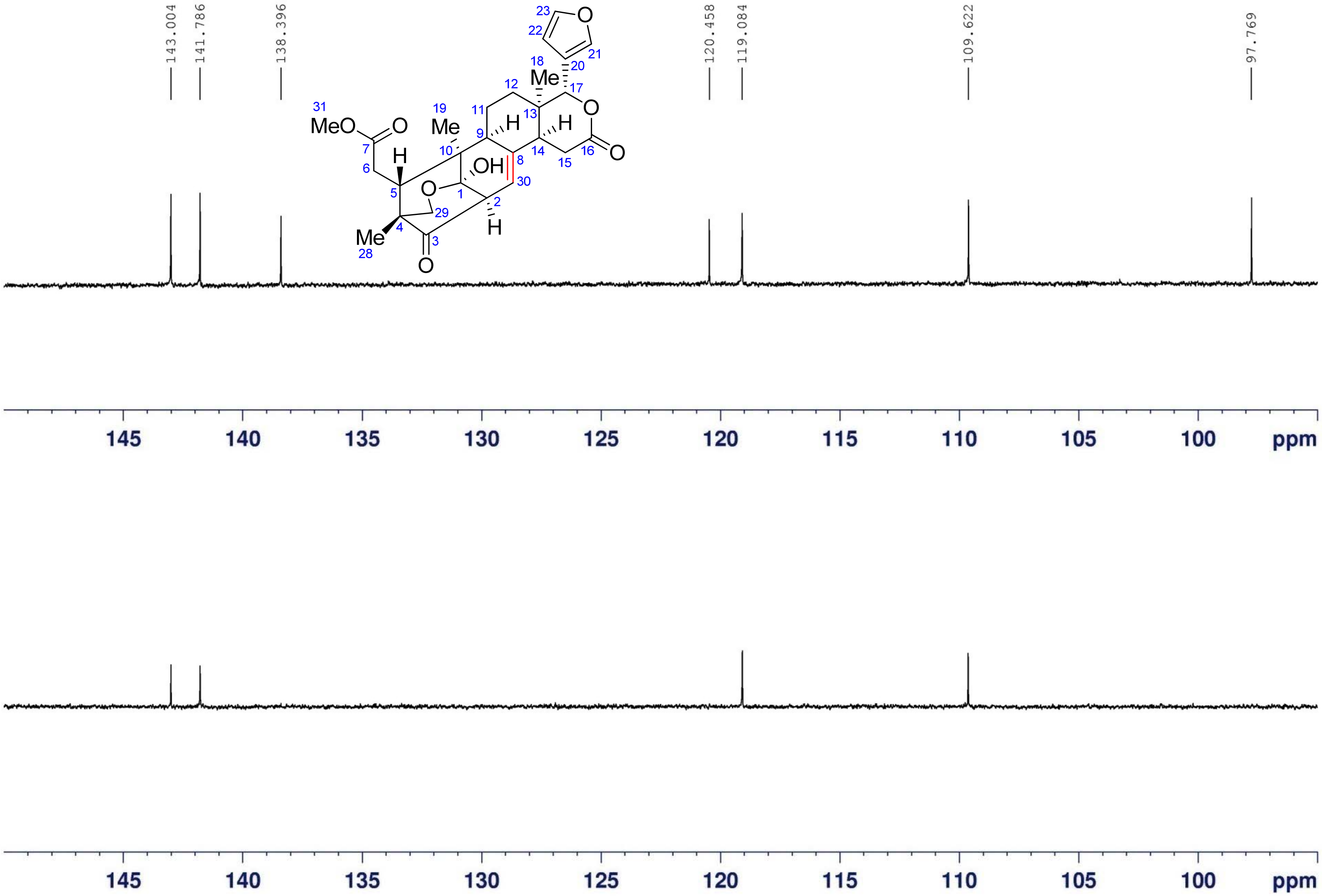

DEPT 135 spectrum of **9** in $CDCl_3$

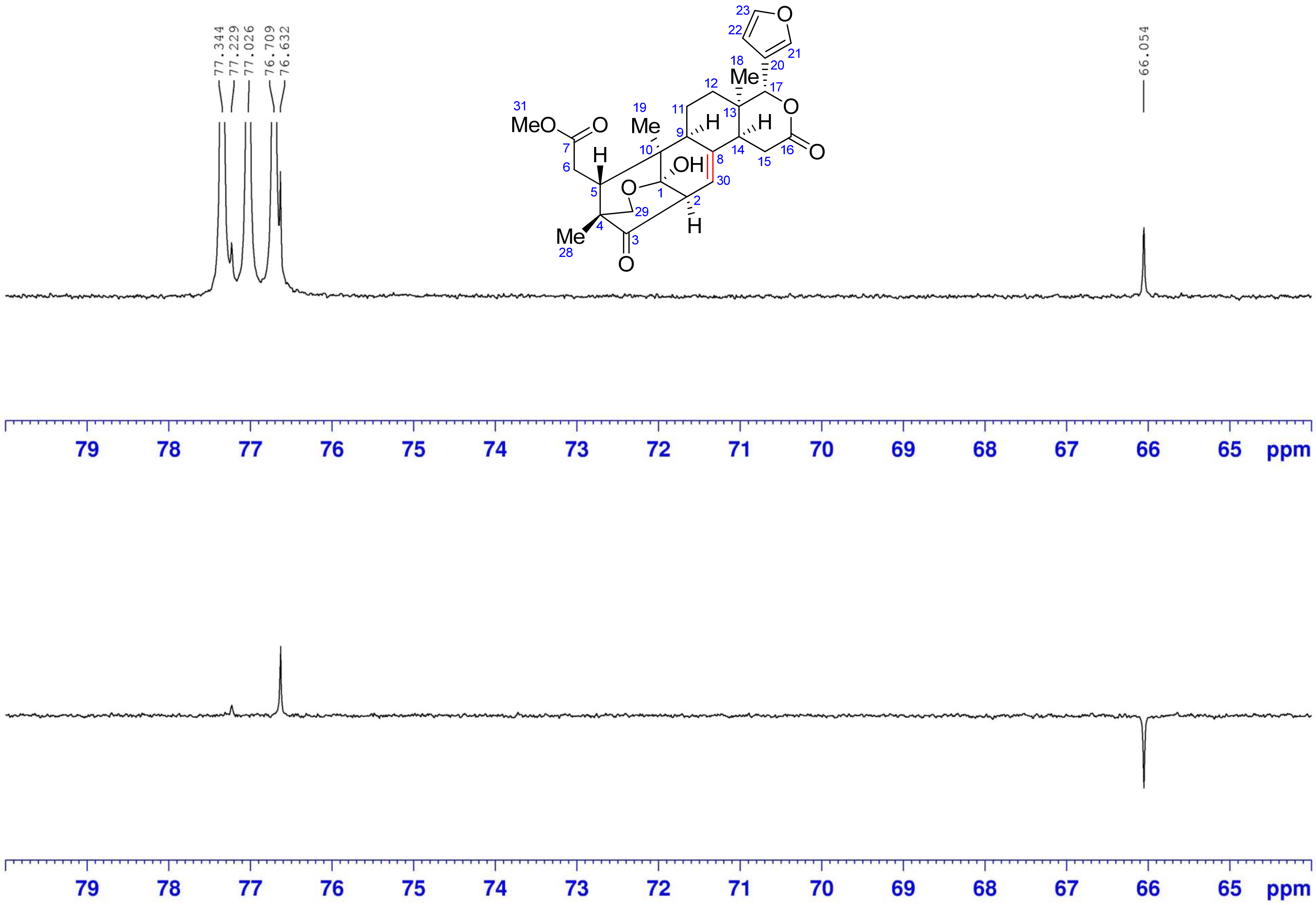

DEPT 135 spectrum of **9** in $CDCl_3$

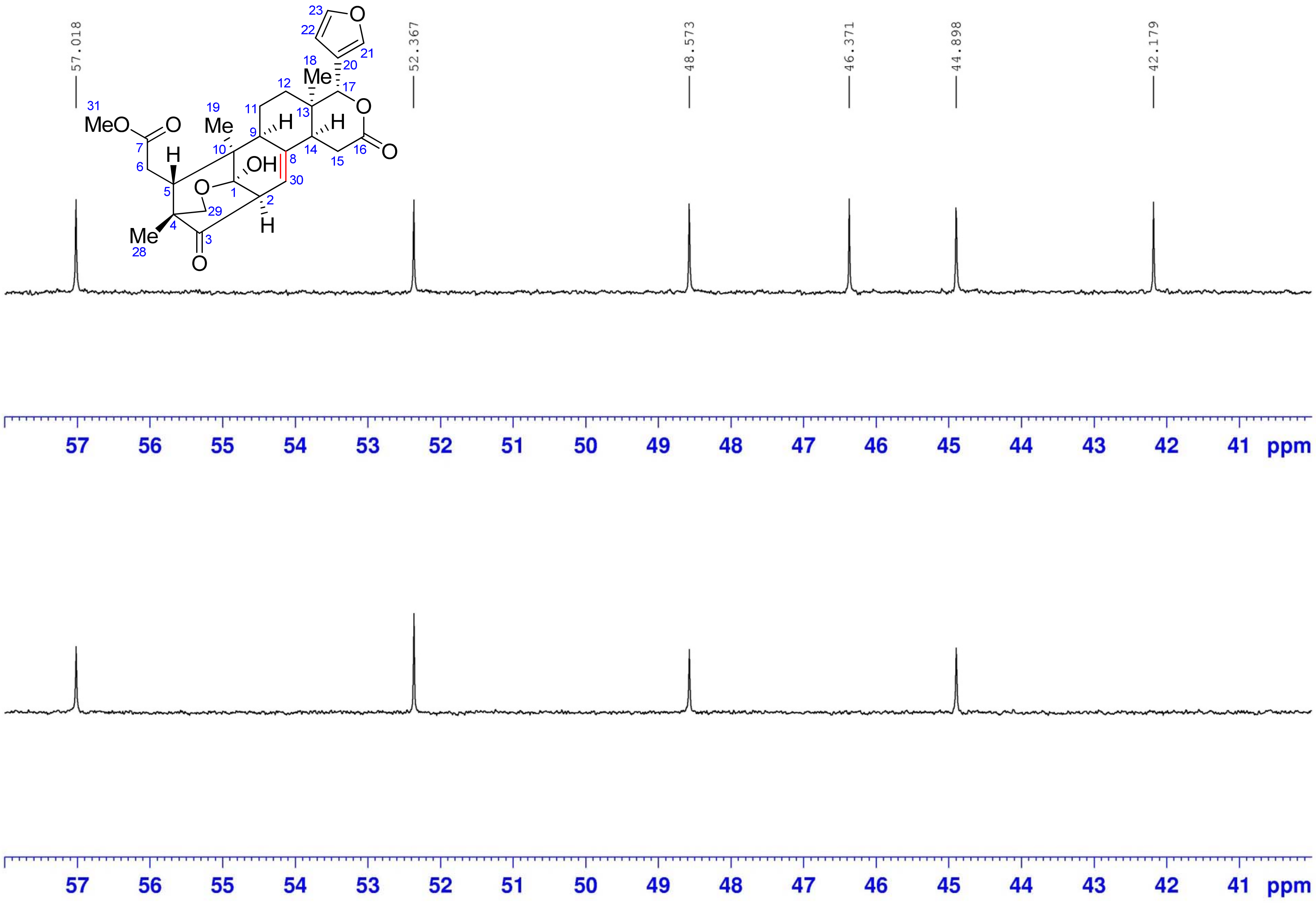

DEPT 135 spectrum of **9** in $CDCl_3$

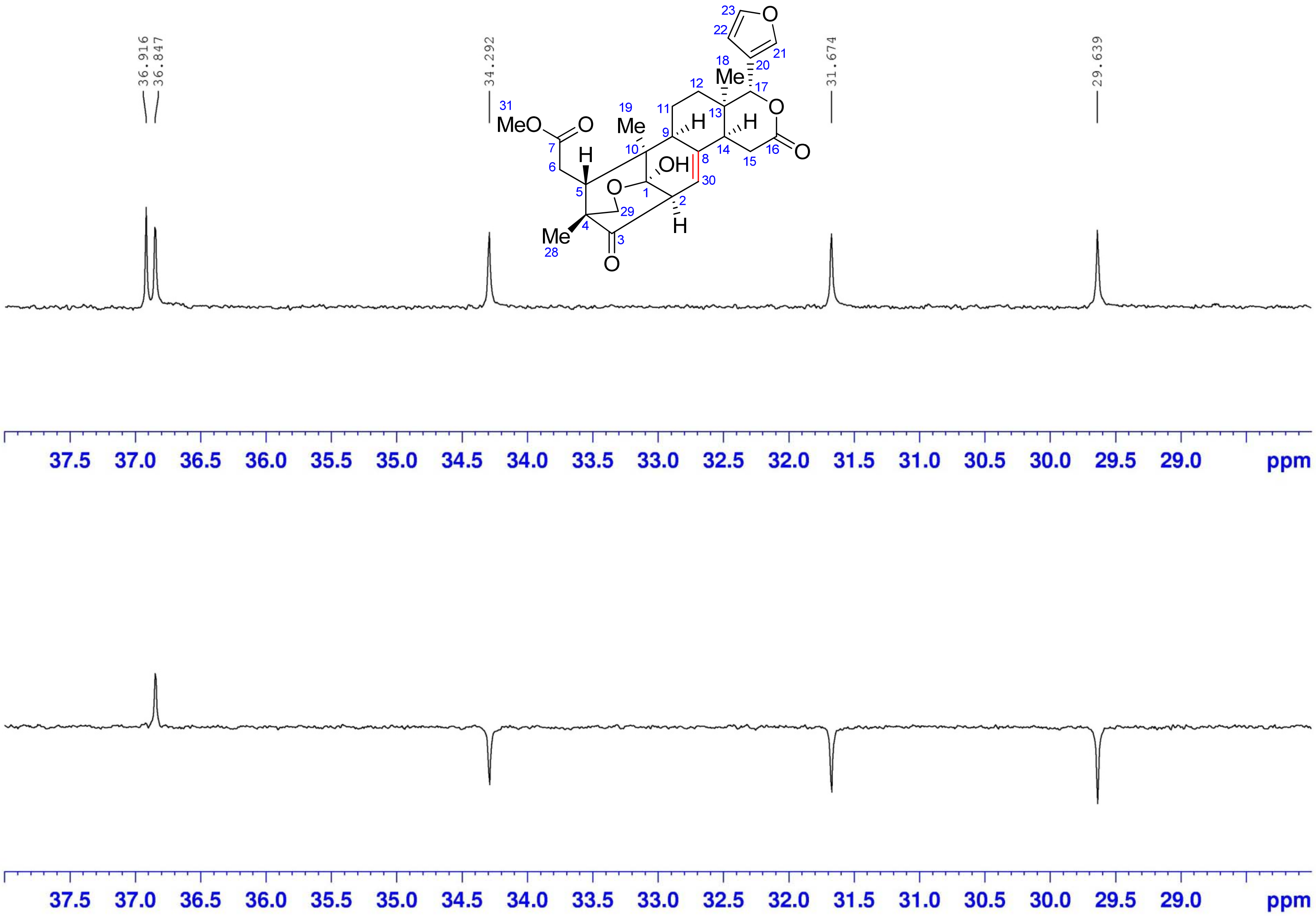

DEPT 135 spectrum of **9** in $CDCl_3$

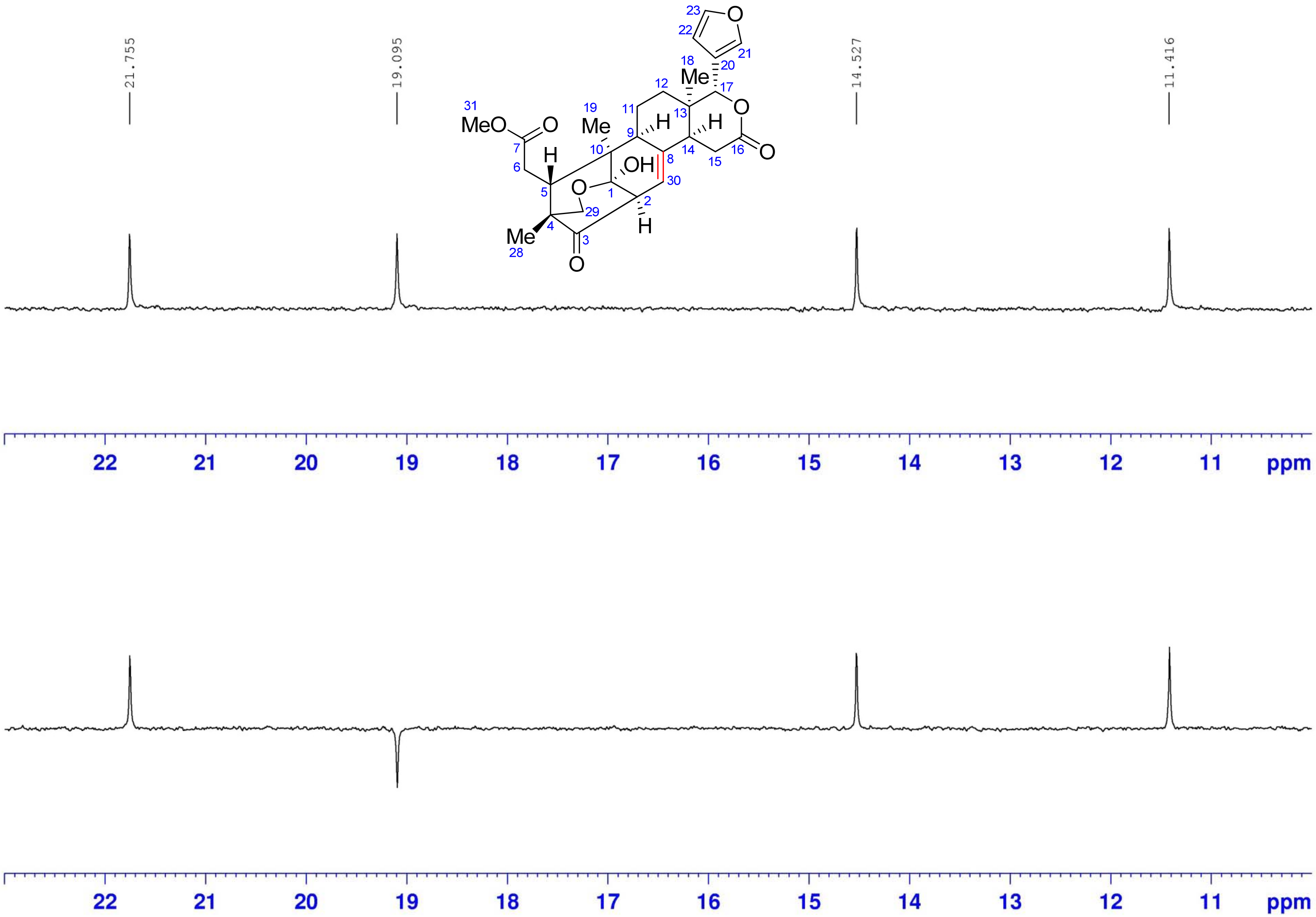

LR-ESIMS for compound **9a**

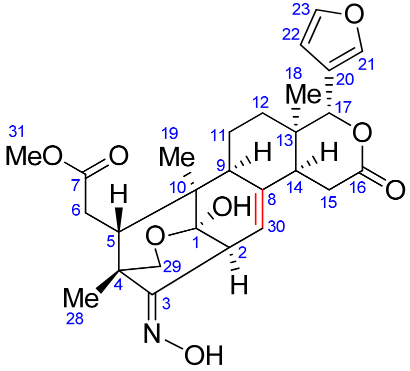

Generic Display Report

Analysis Info		Acquisition D	2018/10/1 20:32:42
Analysis Name	D:\MS\RJL\20181001\66-1_RA1_01_1592.d		
Method	lc-7.9	Operator	bruker
Sample Name	66-1 20140922-alternating-500-50-2200-MS2_1592.m	Instrument	amaZon SL
Comment			

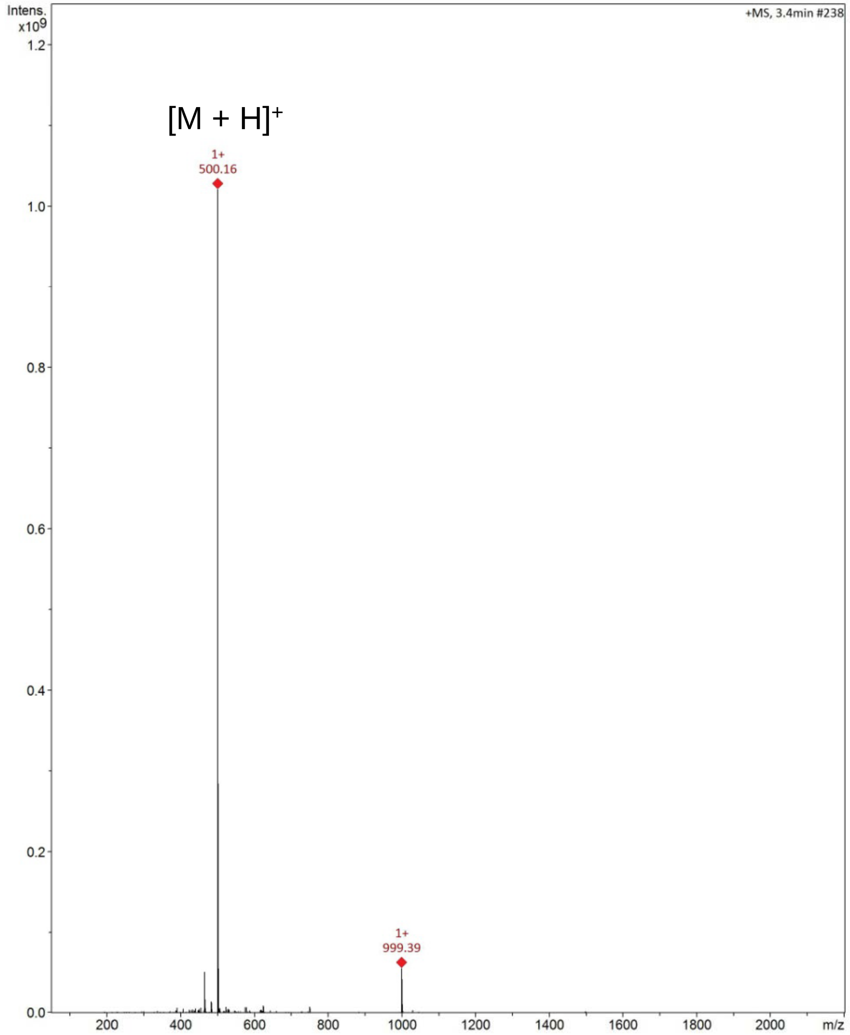

Bruker Compass DataAnalysis

^{1}H NMR (400 MHz) spectrum of **9a** in $CDCl_3$

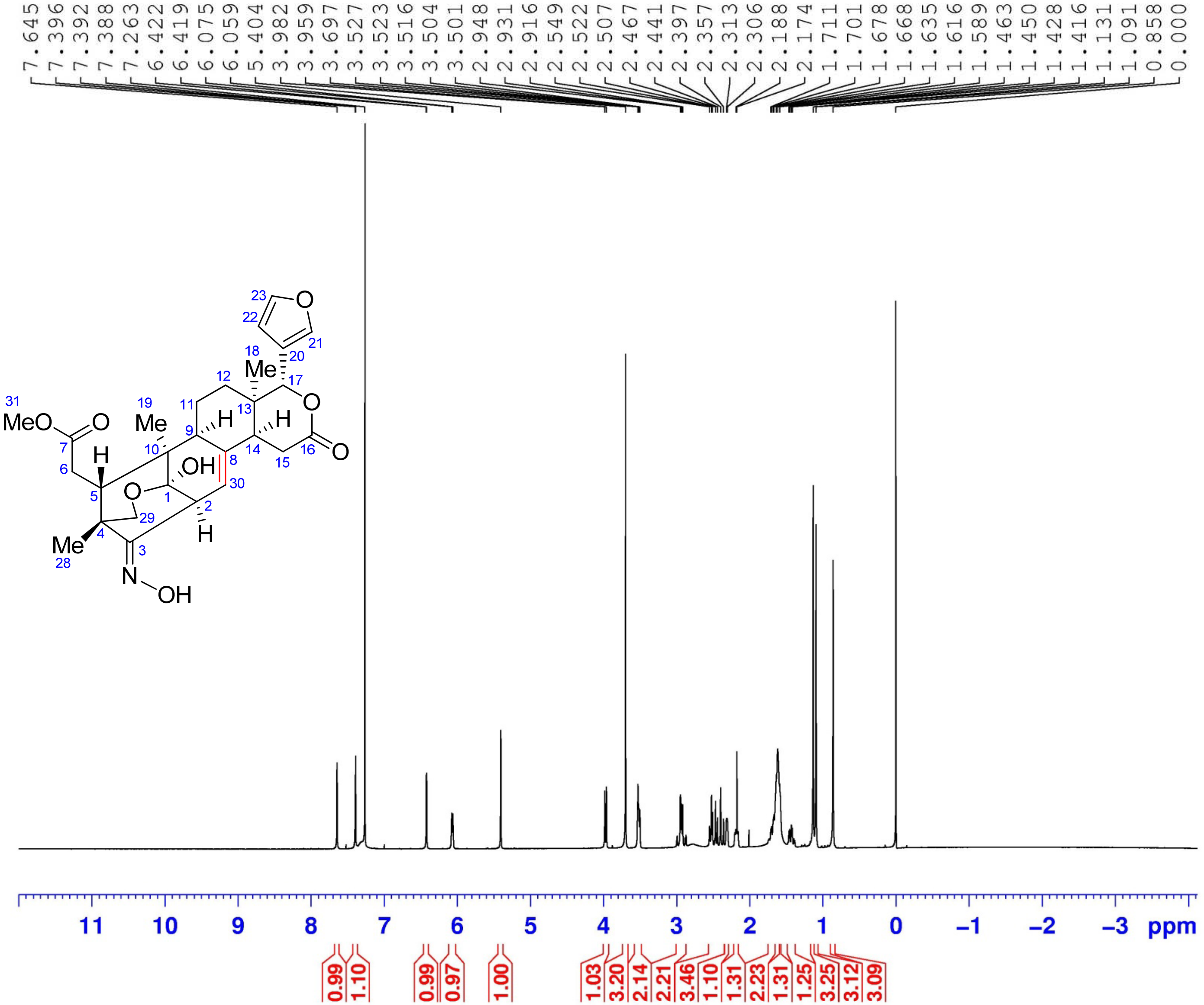


```
NAME       czc-1-ibx-CH3-yansuanqianan
EXPNO                    1
PROCNO                   1
Date_             20171001
Time                 21.06
INSTRUM              spect
PROBHD   5 mm CPPBBO BB
PULPROG               zg30
TD                   65536
SOLVENT              CDCl3
NS                      16
DS                       2
SWH               8223.685 Hz
FIDRES            0.125483 Hz
AQ               3.9846387 sec
RG                   208.5
DW                  60.800 usec
DE                   10.00 usec
TE                   297.0 K
D1              1.00000000 sec
TD0                      1

======== CHANNEL f1 ========
SFO1           400.1324710 MHz
NUC1                    1H
P1                   11.50 usec
SI                   65536
SF             400.1300084 MHz
WDW                     EM
SSB                      0
LB                    0.30 Hz
GB                       0
PC                    1.00
```

^{1}H NMR (400 MHz) spectrum of **9a** in $CDCl_3$

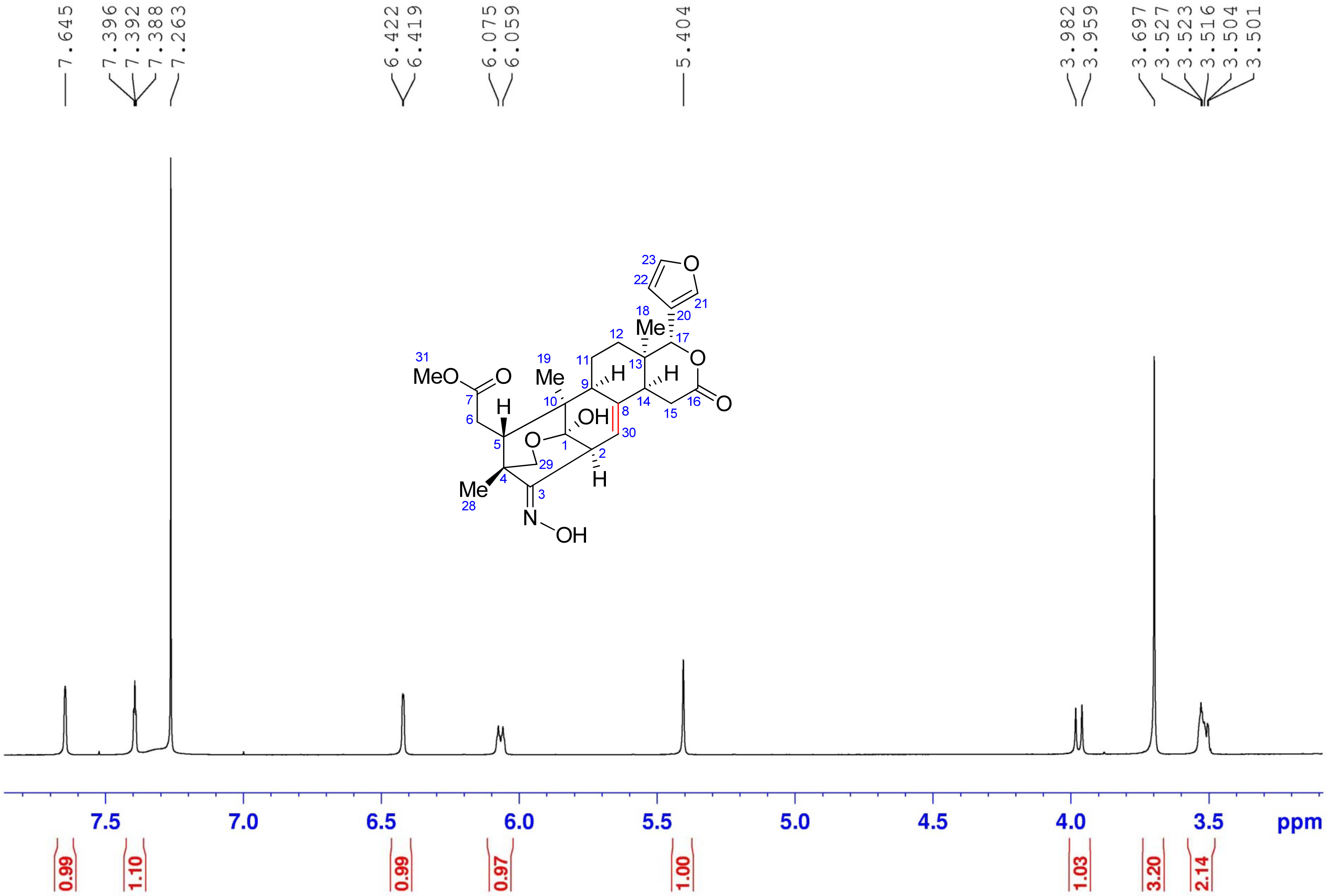

^{1}H NMR (400 MHz) spectrum of **9a** in $CDCl_3$

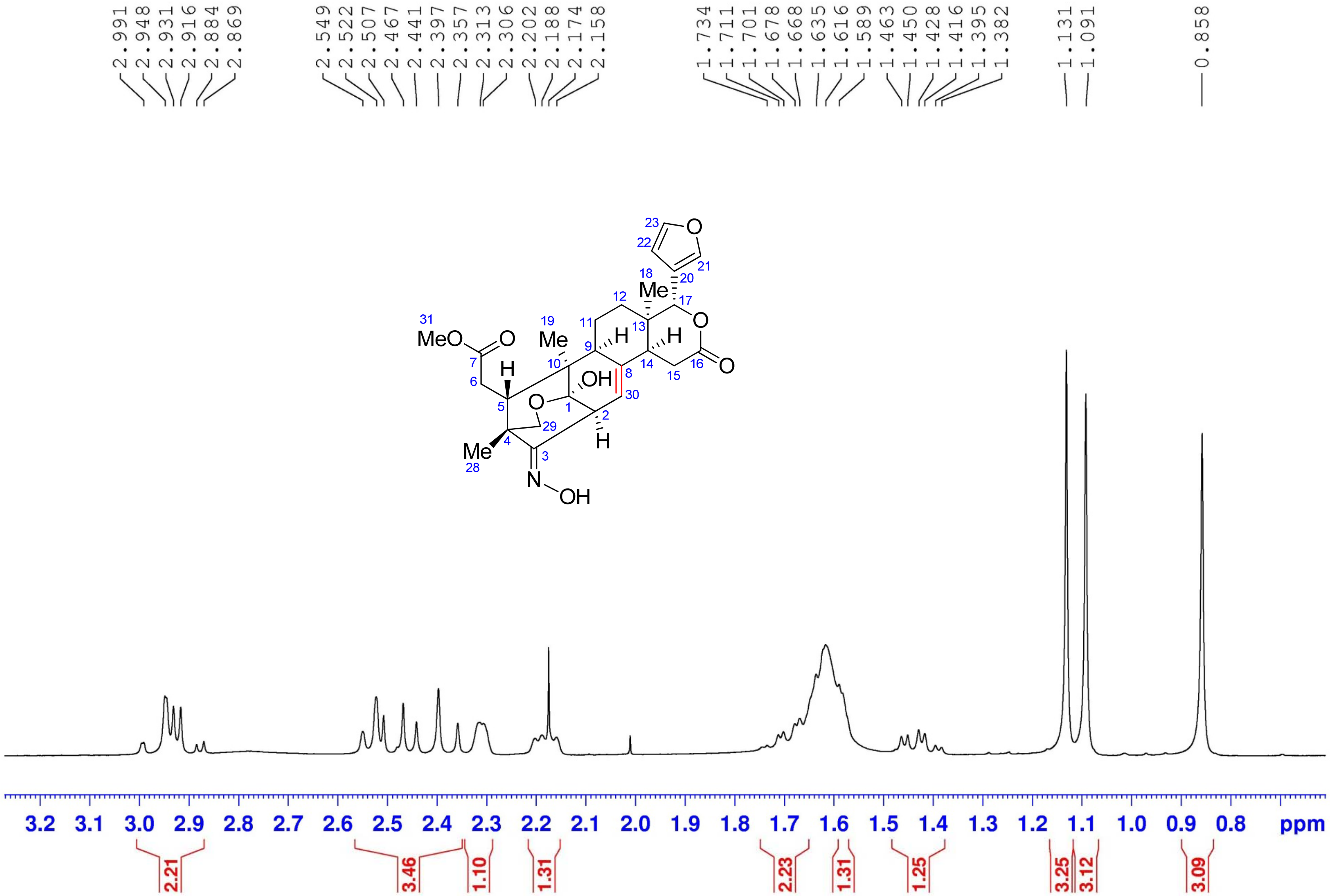

^{13}C NMR (100 MHz) spectrum of **9a** in $CDCl_3$

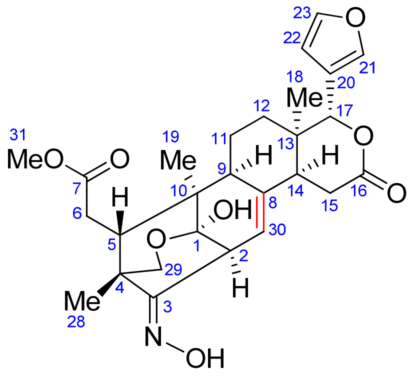

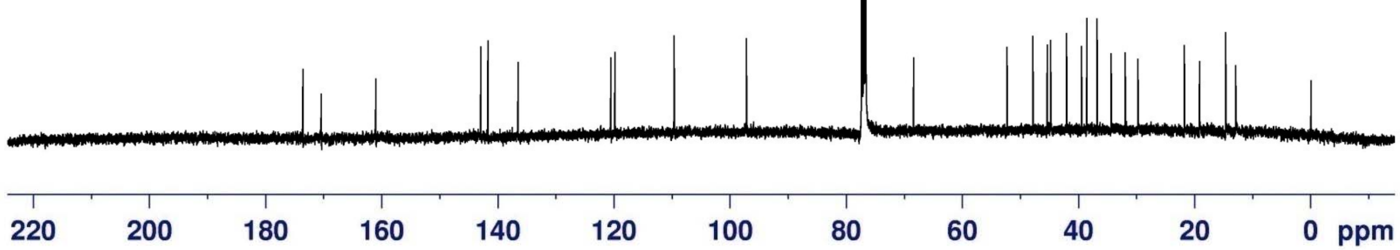

NAME	czc-1-ibx-CH3-yansuanqi
EXPNO	2
PROCNO	1
Date_	20171001
Time	22.05
INSTRUM	spect
PROBHD	5 mm CPPBBO BB
PULPROG	zgpg30
TD	65536
SOLVENT	CDCl3
NS	1024
DS	4
SWH	24038.461 Hz
FIDRES	0.366798 Hz
AQ	1.3631988 sec
RG	130.26
DW	20.800 usec
DE	18.00 usec
TE	297.0 K
D1	2.00000000 sec
D11	0.03000000 sec
TD0	1

======== CHANNEL f1 ========

SFO1	100.6233324 MHz
NUC1	13C
P1	10.00 usec
SI	32768
SF	100.6127689 MHz
WDW	EM
SSB	0
LB	1.00 Hz
GB	0
PC	1.40

^{13}C NMR (100 MHz) spectrum of **9a** in $CDCl_3$

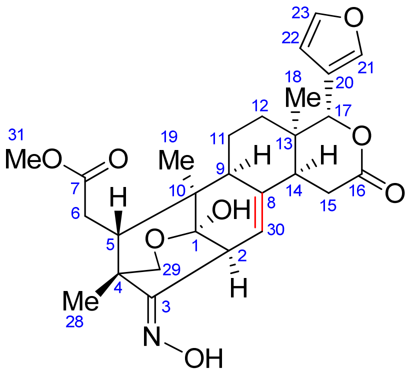

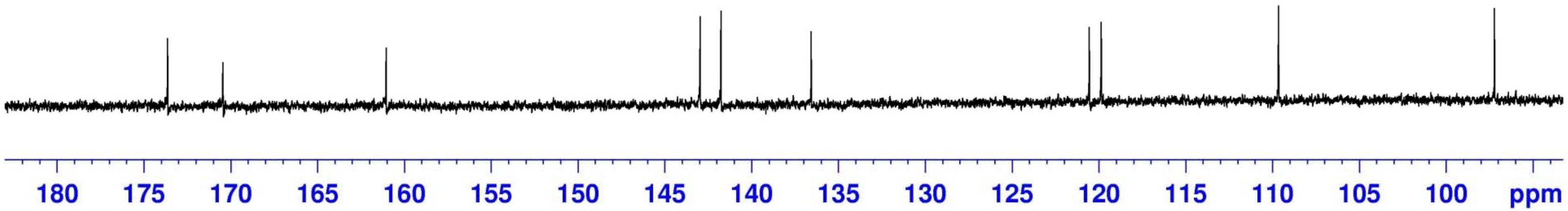

^{13}C NMR (100 MHz) spectrum of **9a** in $CDCl_3$

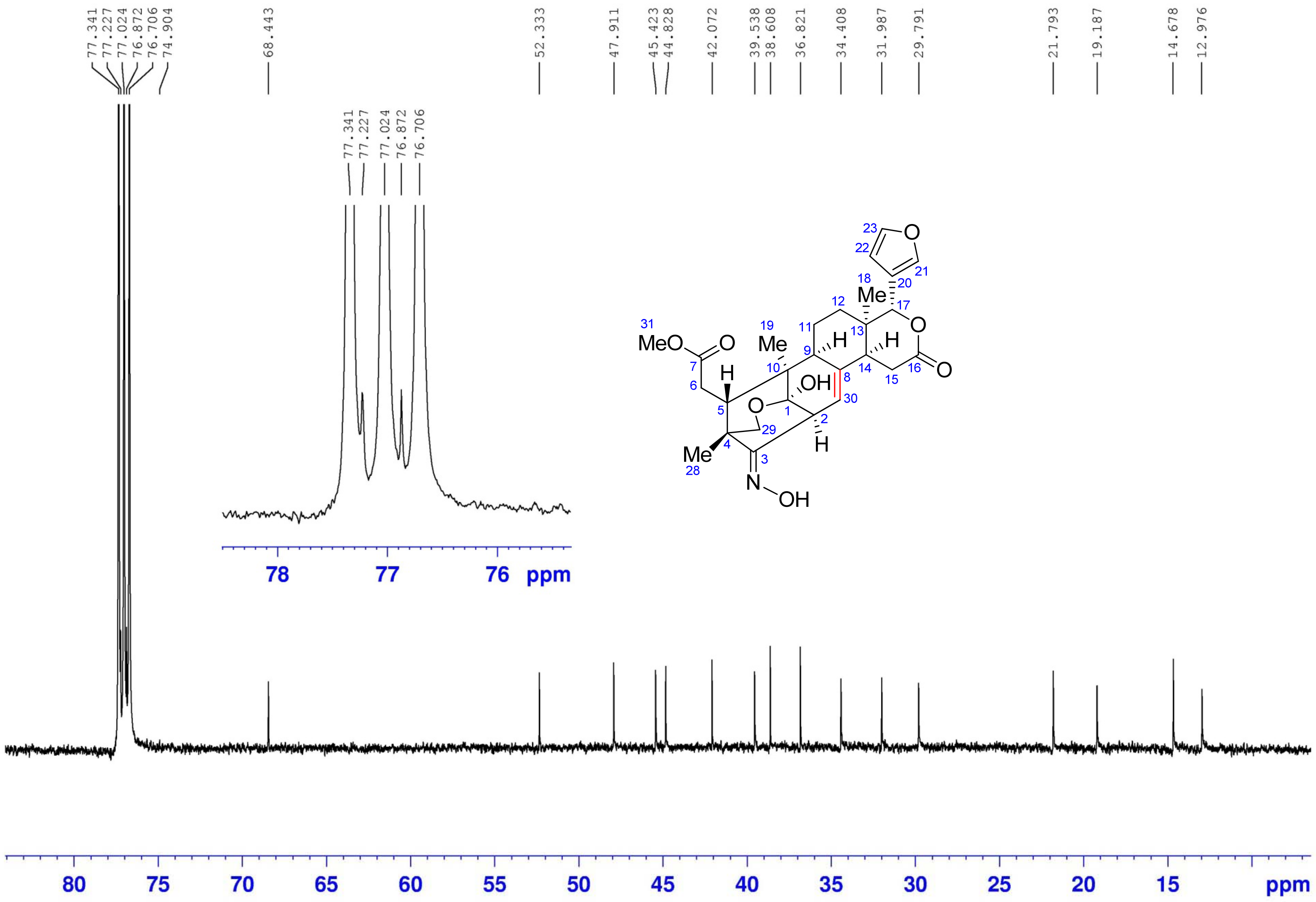

DEPT 135 spectrum of **9a** in $CDCl_3$

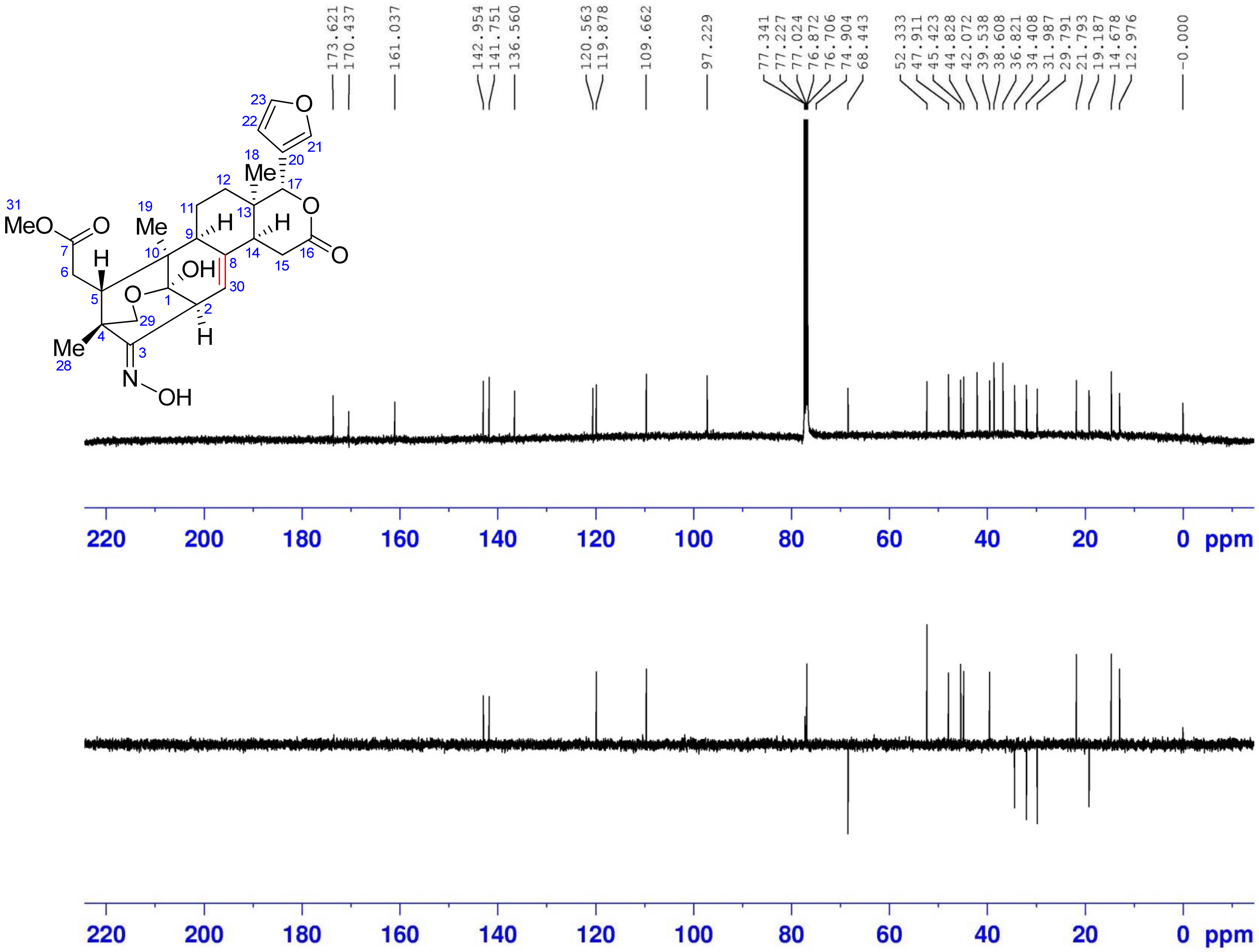


```
NAME       czc-1-ibx-CH3-yansuang:
EXPNO                        3
PROCNO                       1
Date_                 20171001
Time                     22.24
INSTRUM                  spect
PROBHD    5 mm CPPBBO BB
PULPROG             deptsp135
TD                       65536
SOLVENT                  CDCl3
NS                         300
DS                           4
SWH                  24038.461 Hz
FIDRES                0.366798 Hz
AQ                   1.3631988 sec
RG                      130.26
DW                      20.800 usec
DE                       18.00 usec
TE                       297.0 K
CNST2              145.0000000
D1                  2.00000000 sec
D2                  0.00344828 sec
D12                 0.00002000 sec
TD0                          1

======== CHANNEL f1 ========
SFO1               100.6233324 MHz
NUC1                       13C
P1                       10.00 usec
P13                    2000.00 usec
SI                       32768
SF                 100.6127692 MHz
WDW                         EM
SSB                          0
LB                        1.00 Hz
GB                           0
PC                        1.40
```

DEPT 135 spectrum of **9a** in $CDCl_3$

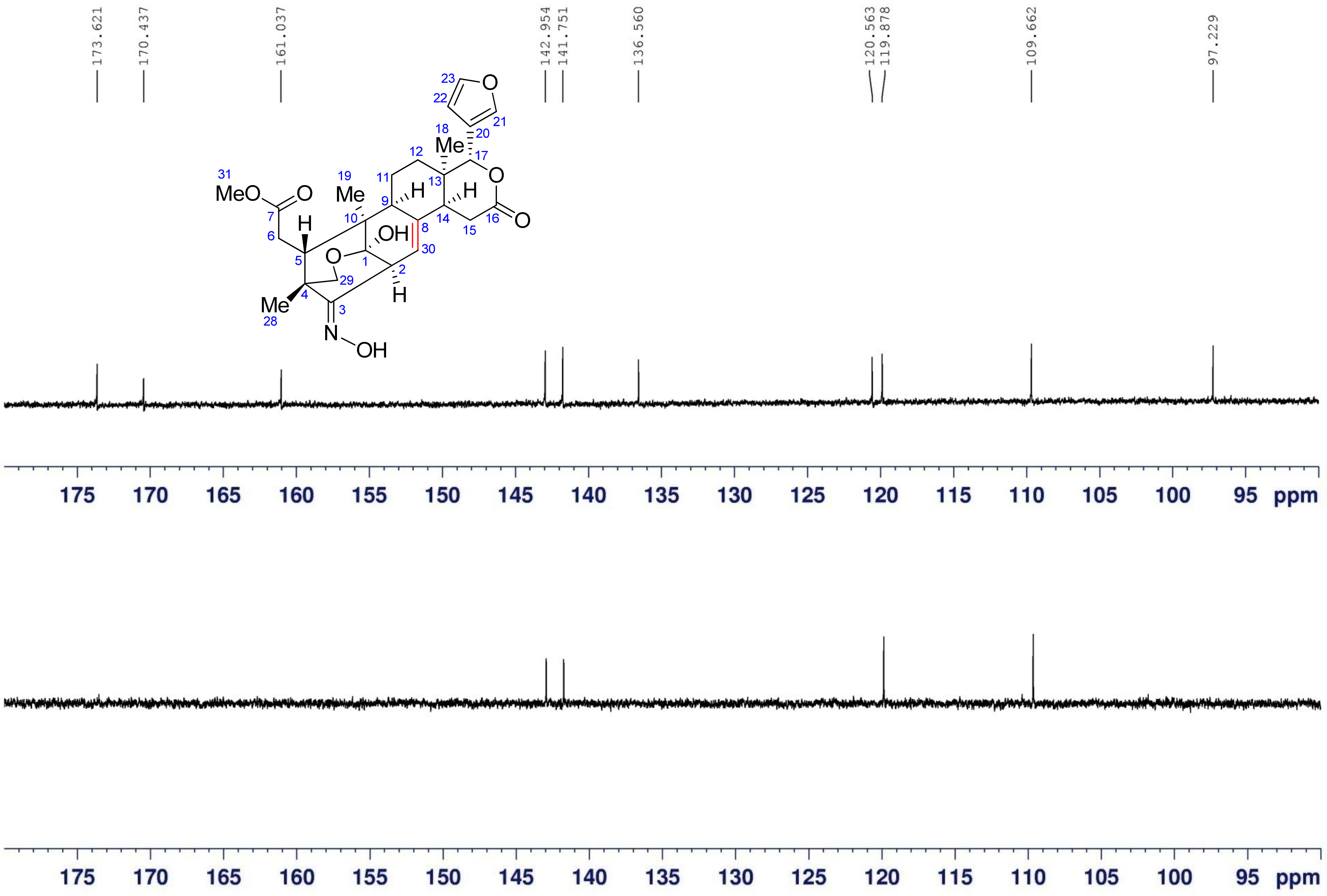

DEPT 135 spectrum of **9a** in $CDCl_3$

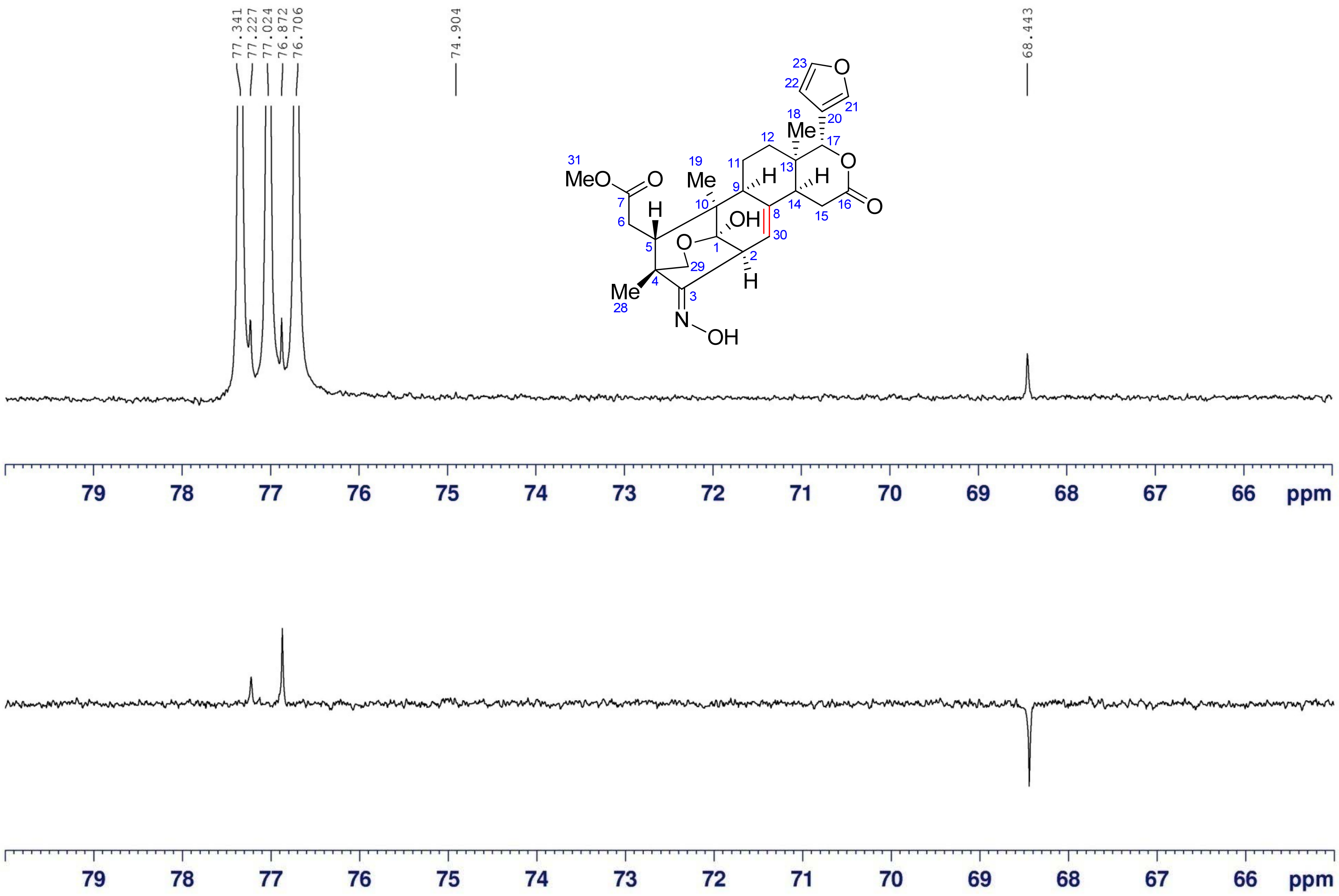

DEPT 135 spectrum of **9a** in $CDCl_3$

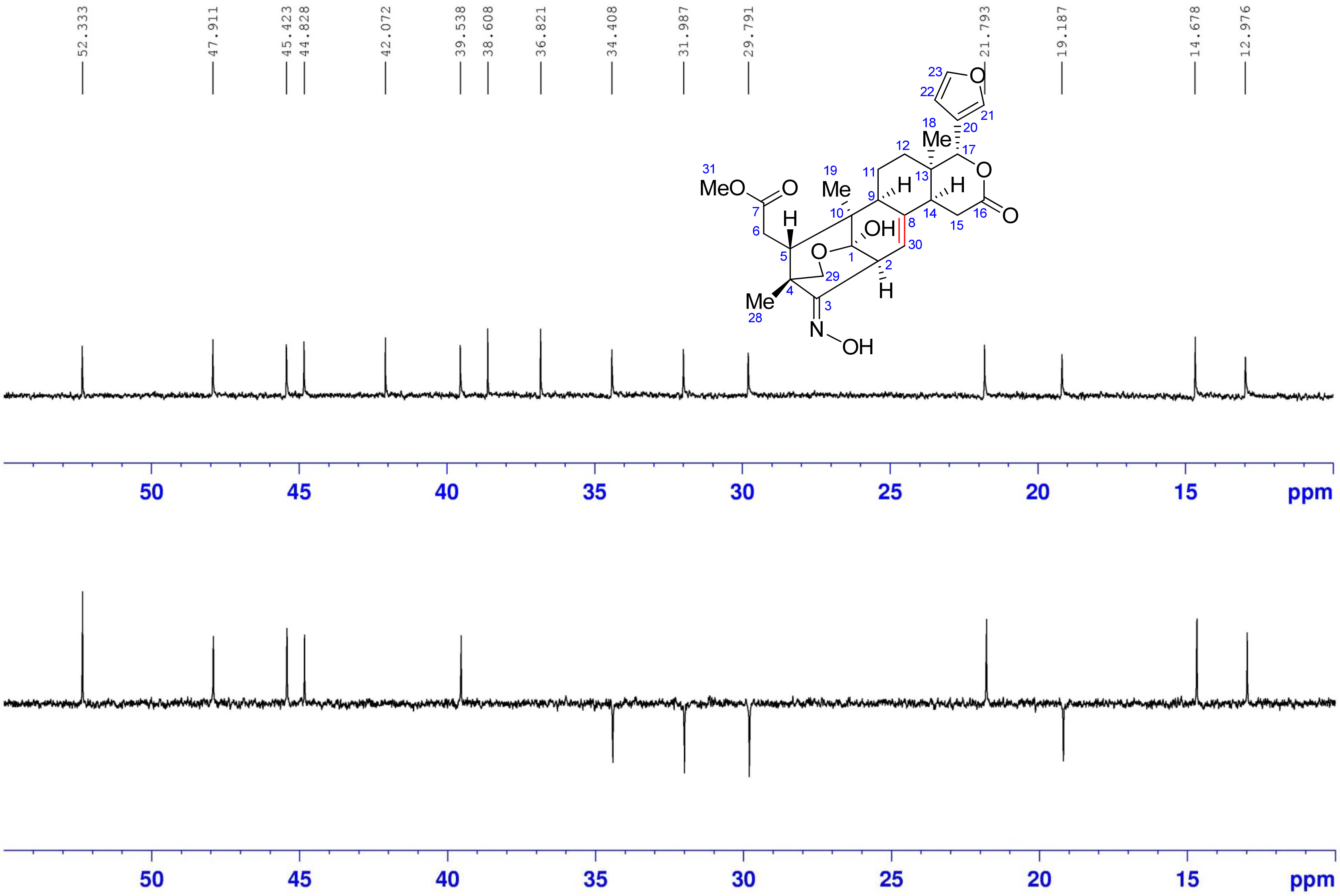

LR-ESIMS for compound **9b**

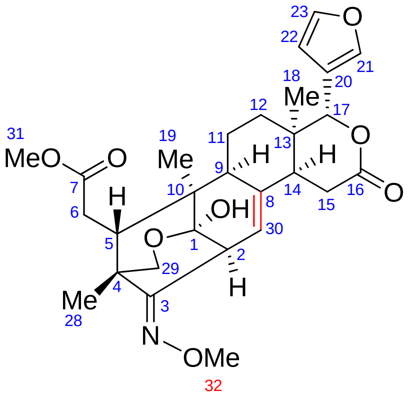

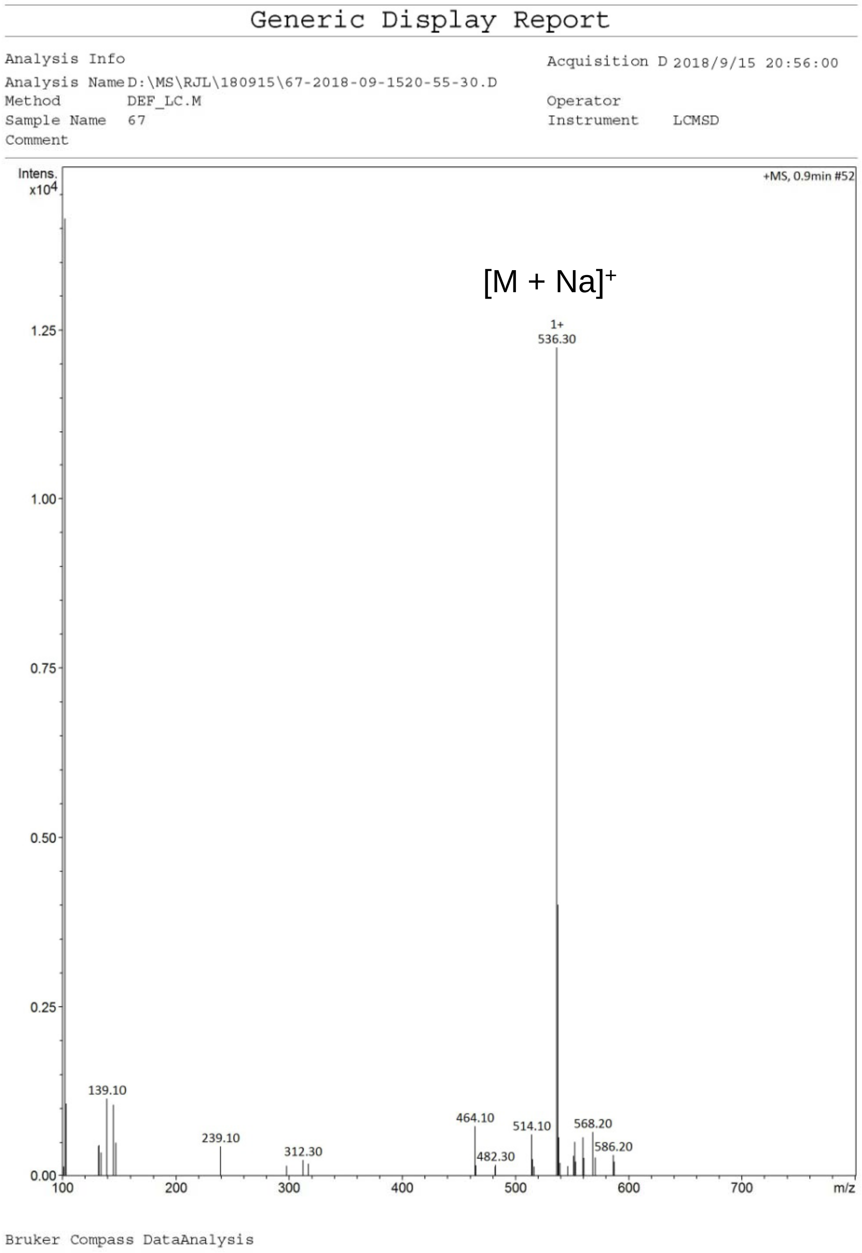

^{1}H NMR (400 MHz) spectrum of **9b** in $CDCl_3$

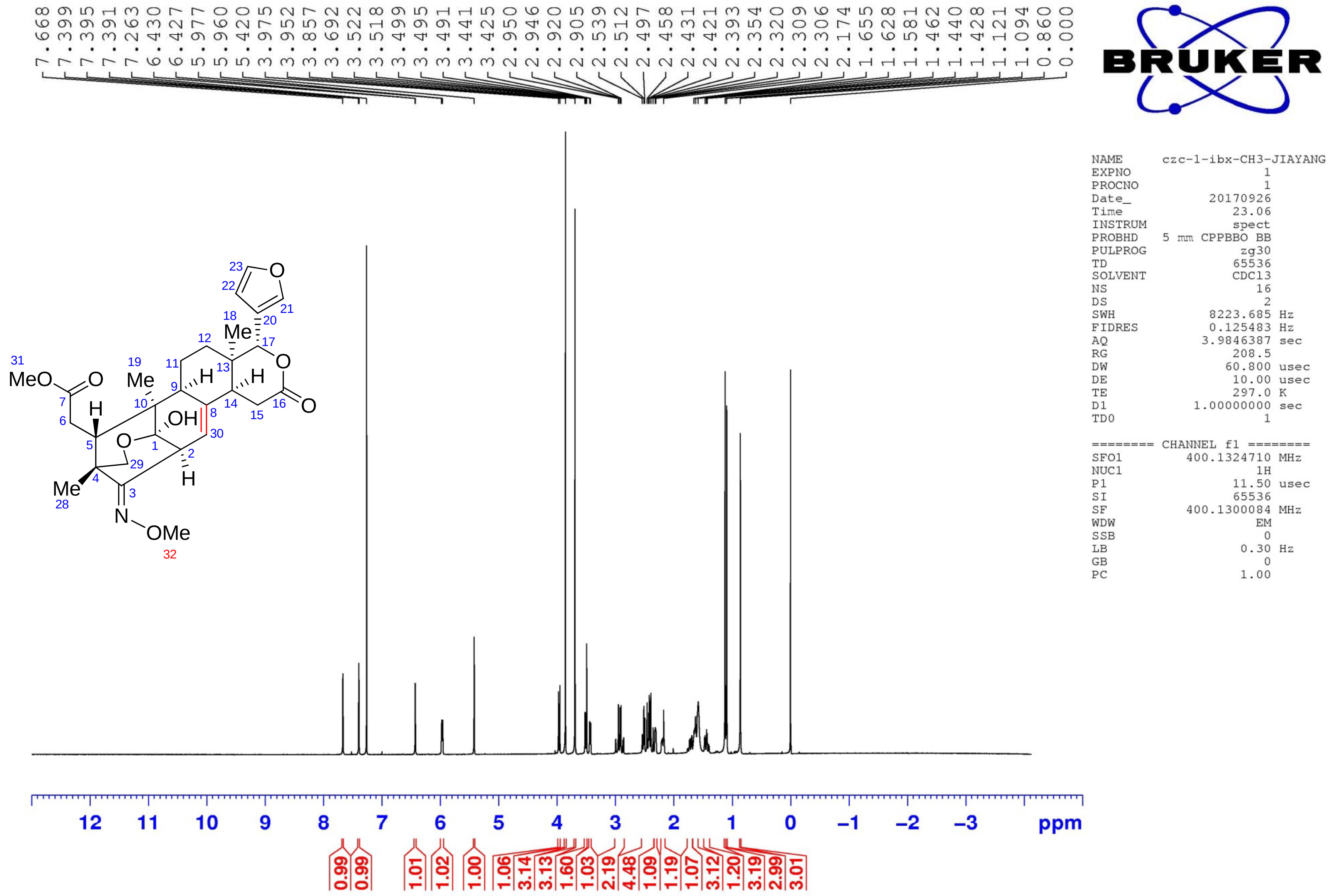

^{1}H NMR (400 MHz) spectrum of **9b** in $CDCl_3$

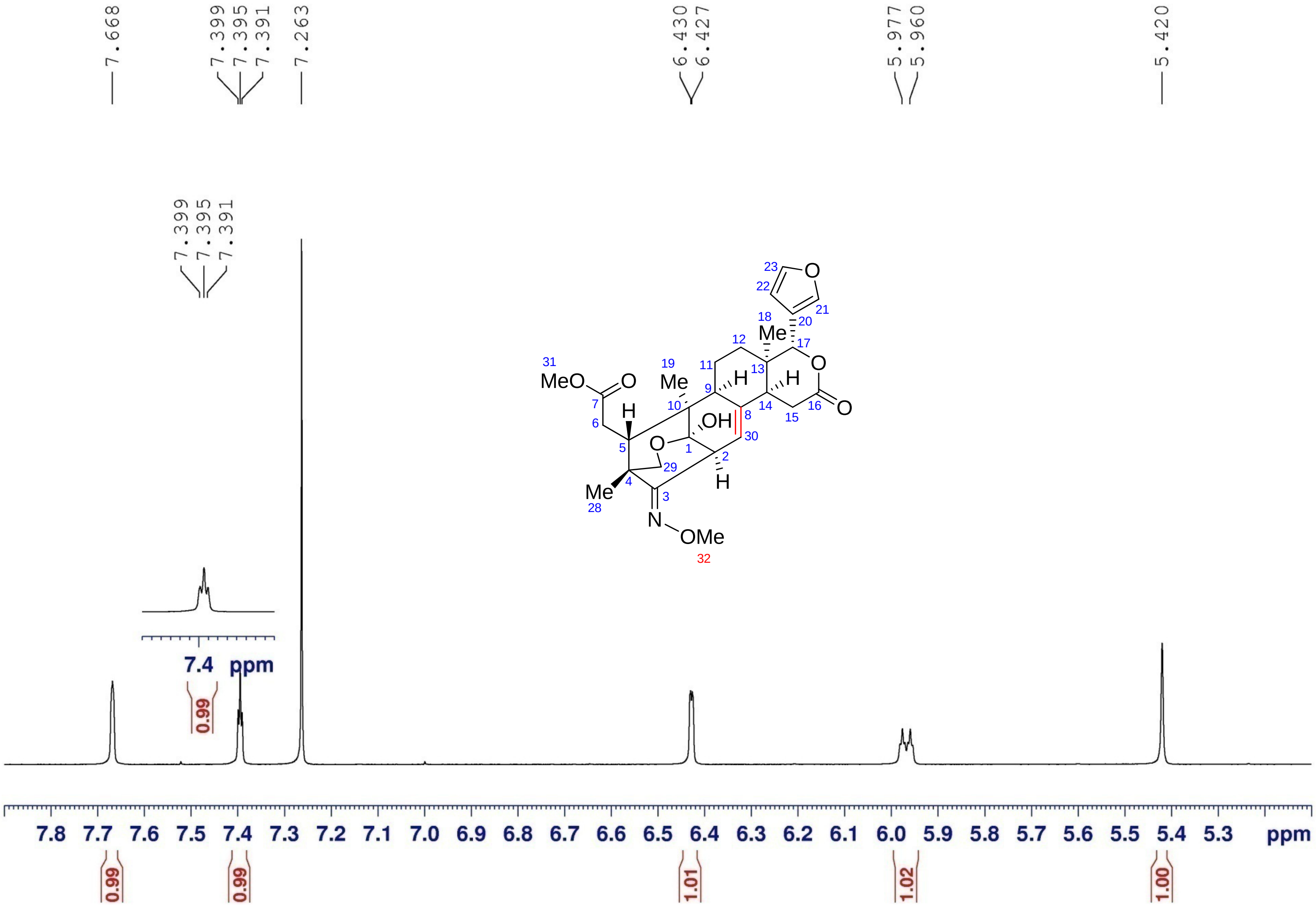

^{1}H NMR (400 MHz) spectrum of **9b** in $CDCl_3$

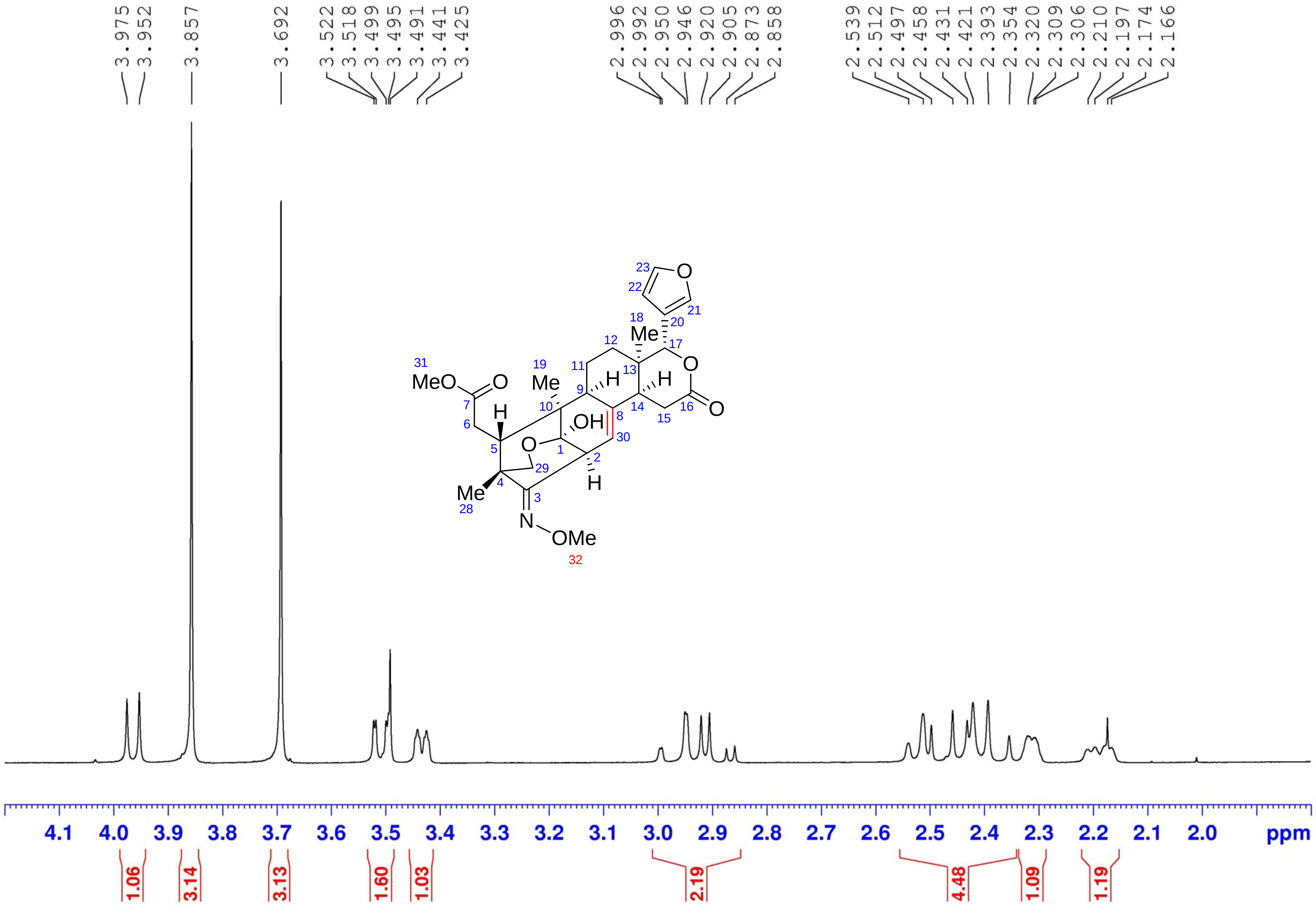

^{1}H NMR (400 MHz) spectrum of **9b** in $CDCl_3$

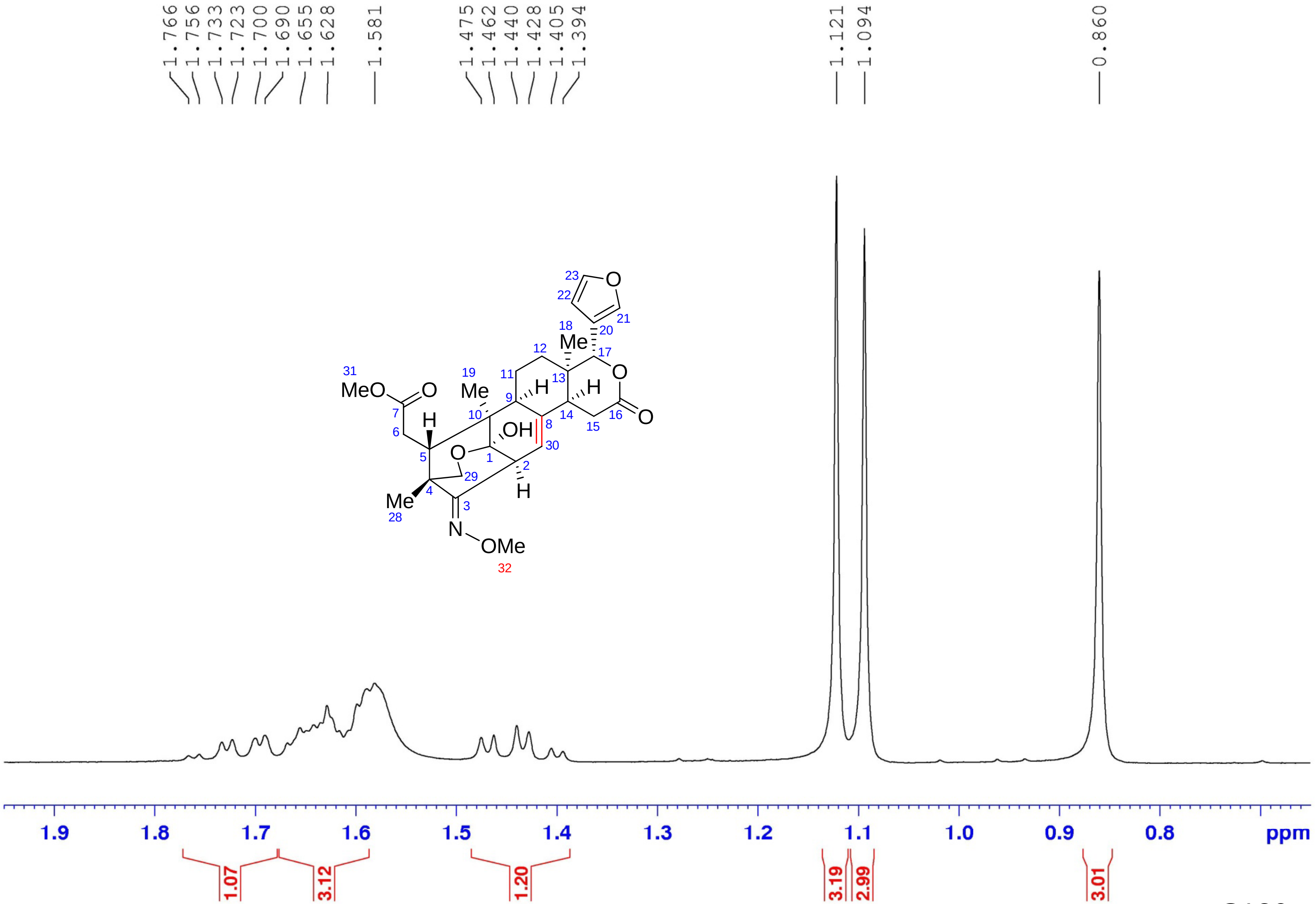

^{13}C NMR (100 MHz) spectrum of **9b** in $CDCl_3$

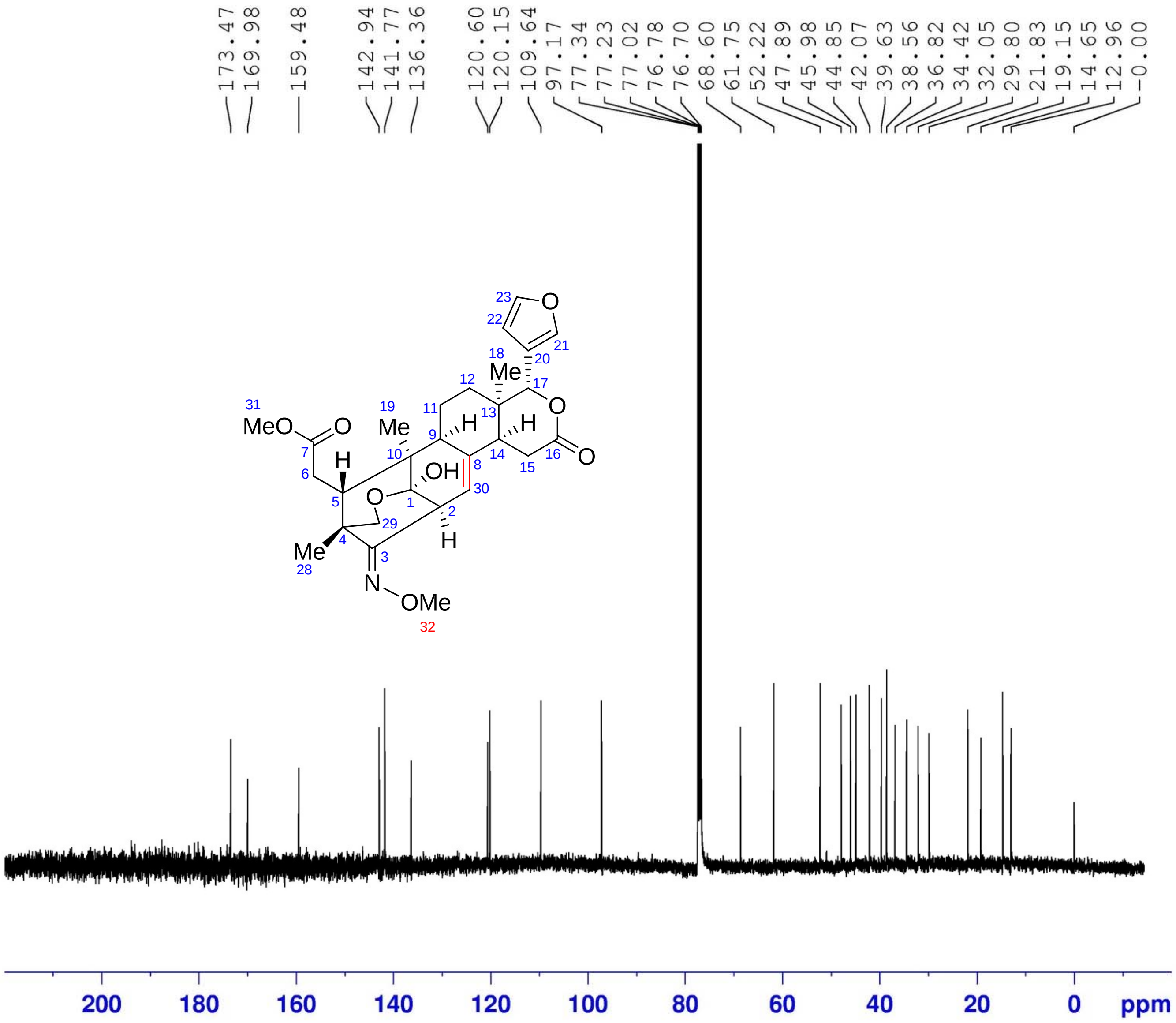


```
NAME       czc-1-ibx-CH3-JIAYANG
EXPNO                      2
PROCNO                     1
Date_               20170927
Time                    8.39
INSTRUM                spect
PROBHD    5 mm CPPBBO BB
PULPROG              zgpg30
TD                     65536
SOLVENT                CDCl3
NS                      1024
DS                         4
SWH               24038.461 Hz
FIDRES             0.366798 Hz
AQ                1.3631988 sec
RG                    117.37
DW                    20.800 usec
DE                     18.00 usec
TE                     297.0 K
D1                2.00000000 sec
D11               0.03000000 sec
TD0                        1

======== CHANNEL f1 ========
SFO1           100.6233324 MHz
NUC1                   13C
P1                     10.00 usec
SI                     32768
SF             100.6127690 MHz
WDW                       EM
SSB                        0
LB                      1.00 Hz
GB                         0
PC                      1.40
```

^{13}C NMR (100 MHz) spectrum of **9b** in $CDCl_3$

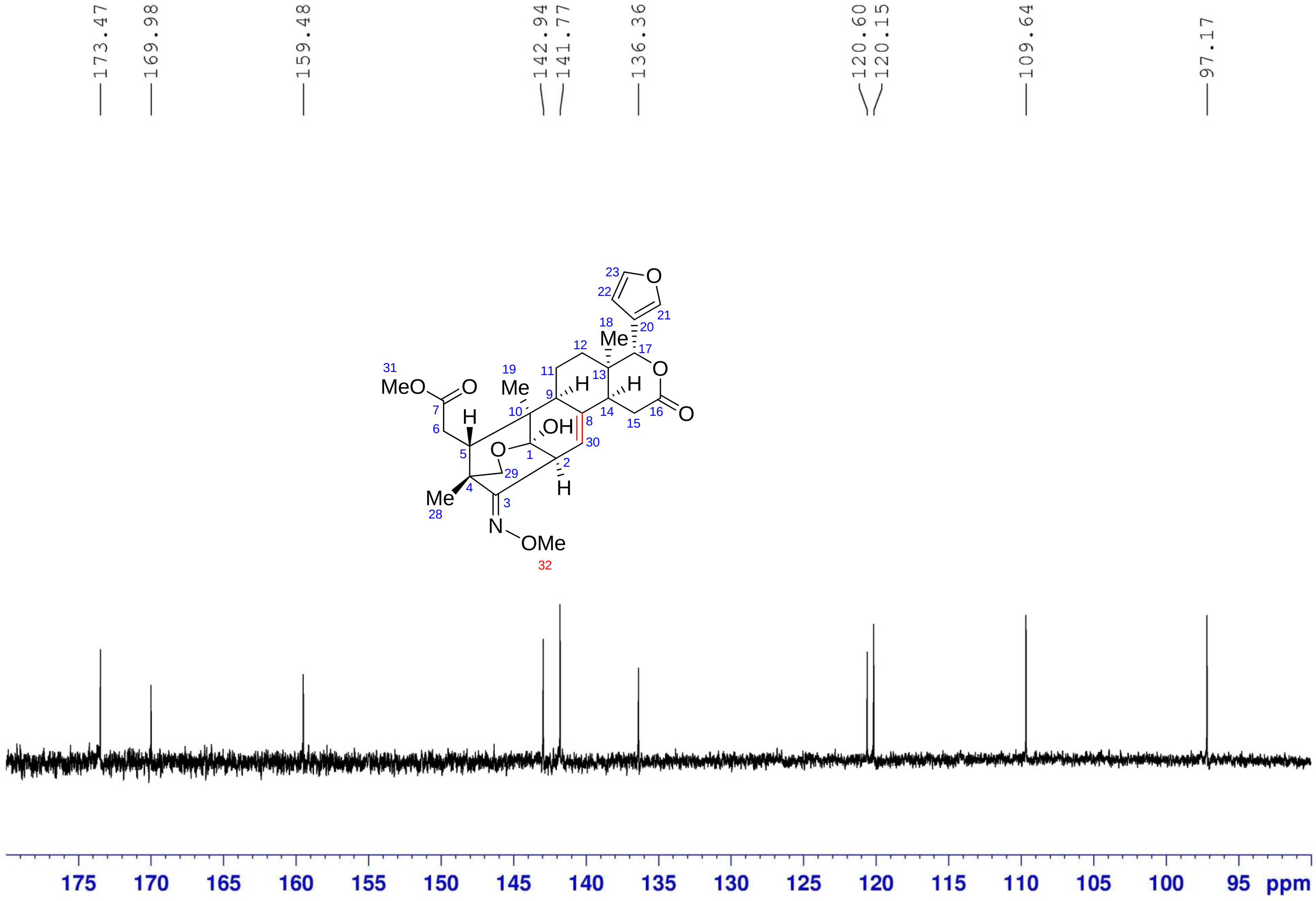

^{13}C NMR (100 MHz) spectrum of **9b** in $CDCl_3$

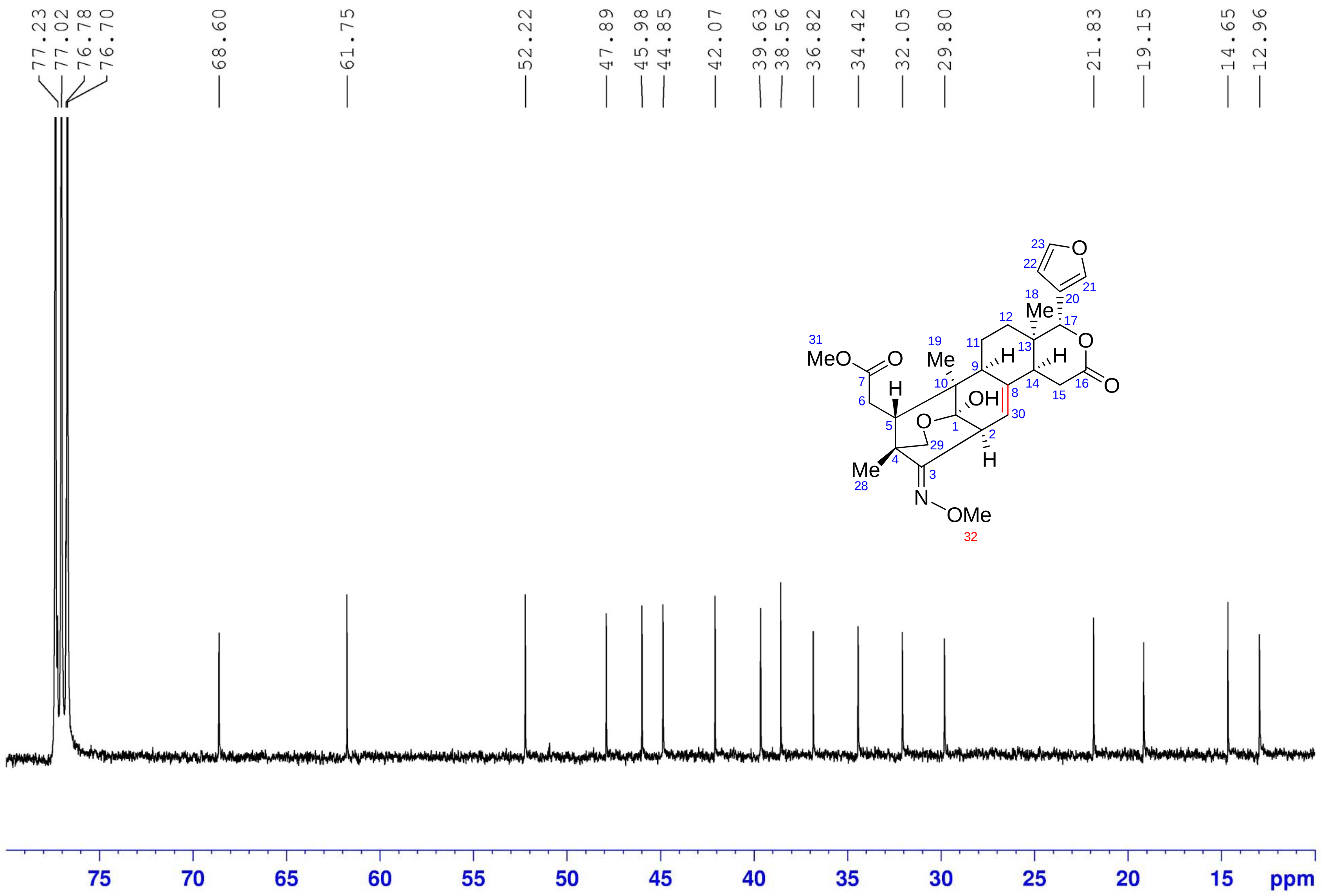

DEPT 135 spectrum of **9b** in $CDCl_3$

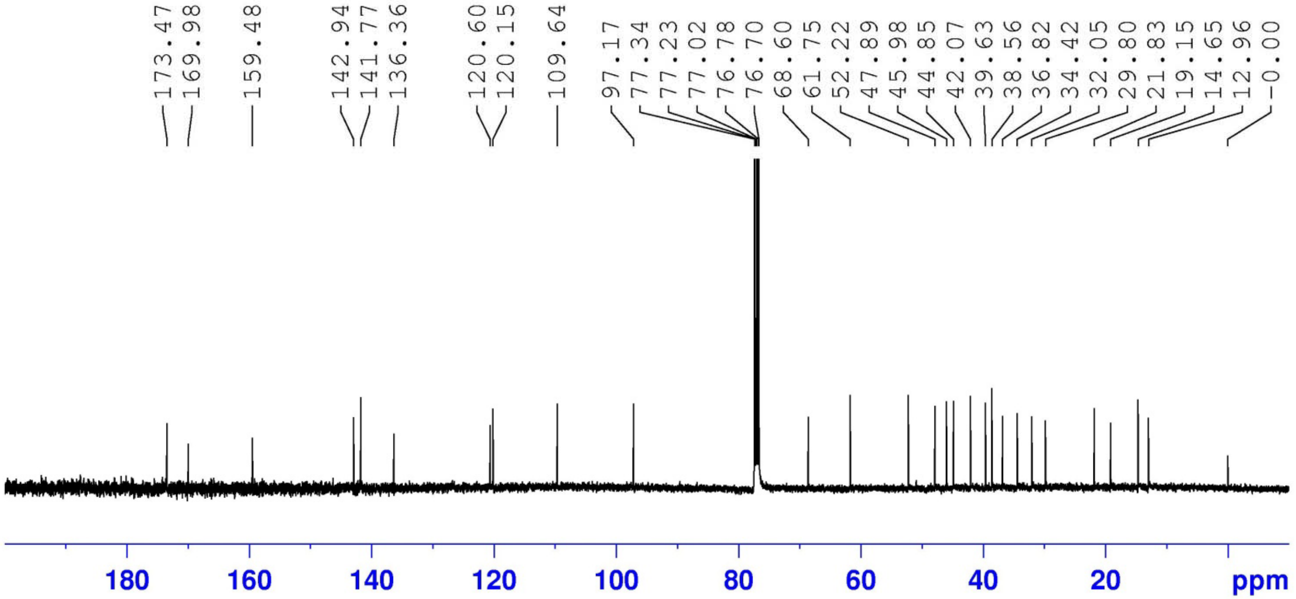

NAME	czc-1-ibx-CH3-JIAYANG
EXPNO	2
PROCNO	1
Date_	20170927
Time	8.39
INSTRUM	spect
PROBHD	5 mm CPPBBO BB
PULPROG	zgpg30
TD	65536
SOLVENT	CDCl3
NS	1024
DS	4
SWH	24038.461 Hz
FIDRES	0.366798 Hz
AQ	1.3631988 sec
RG	117.37
DW	20.800 usec
DE	18.00 usec
TE	297.0 K
D1	2.00000000 sec
D11	0.03000000 sec
TD0	1

======== CHANNEL f1 ========

SFO1	100.6233324 MHz
NUC1	13C
P1	10.00 usec
SI	32768
SF	100.6127690 MHz
WDW	EM
SSB	0
LB	1.00 Hz
GB	0
PC	1.40

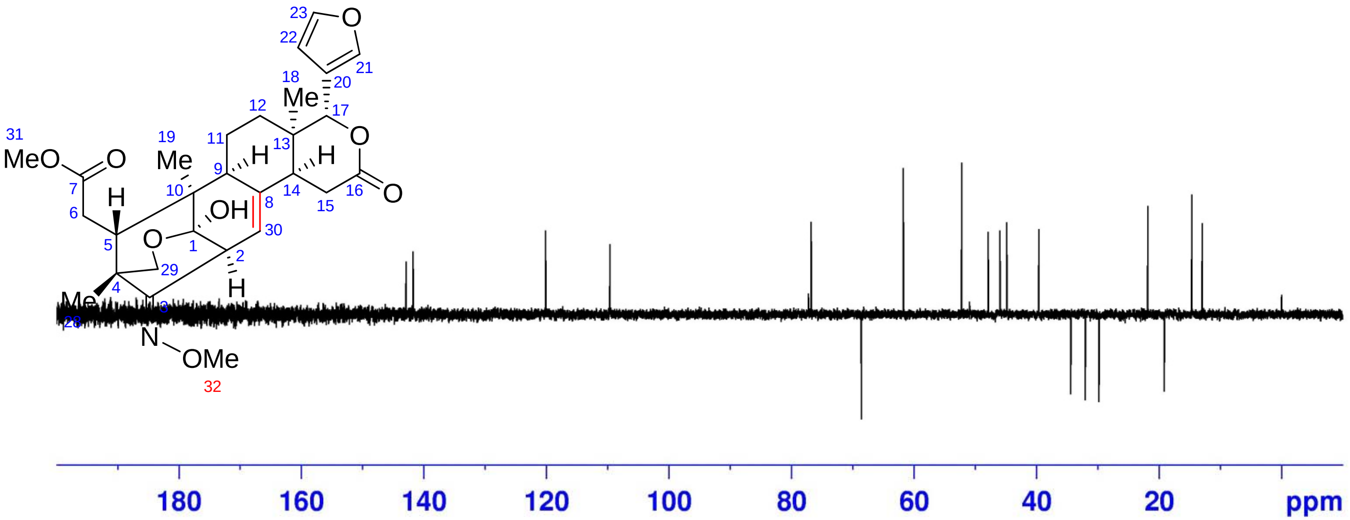

DEPT 135 spectrum of **9b** in $CDCl_3$

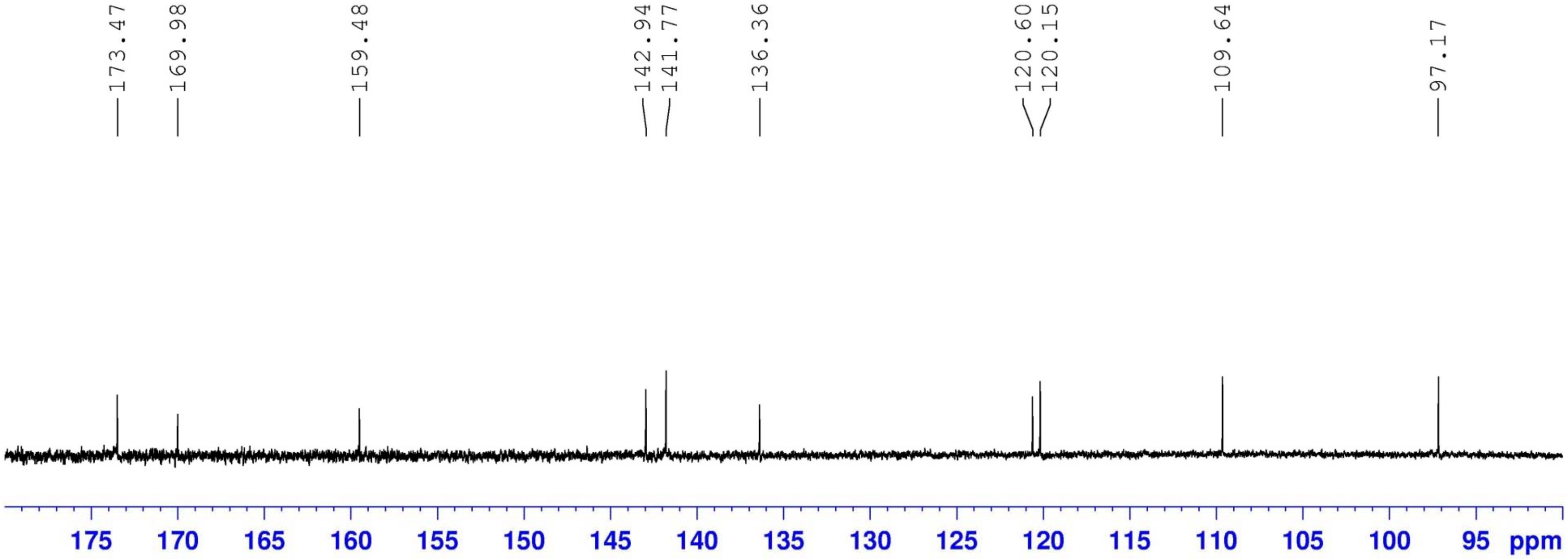

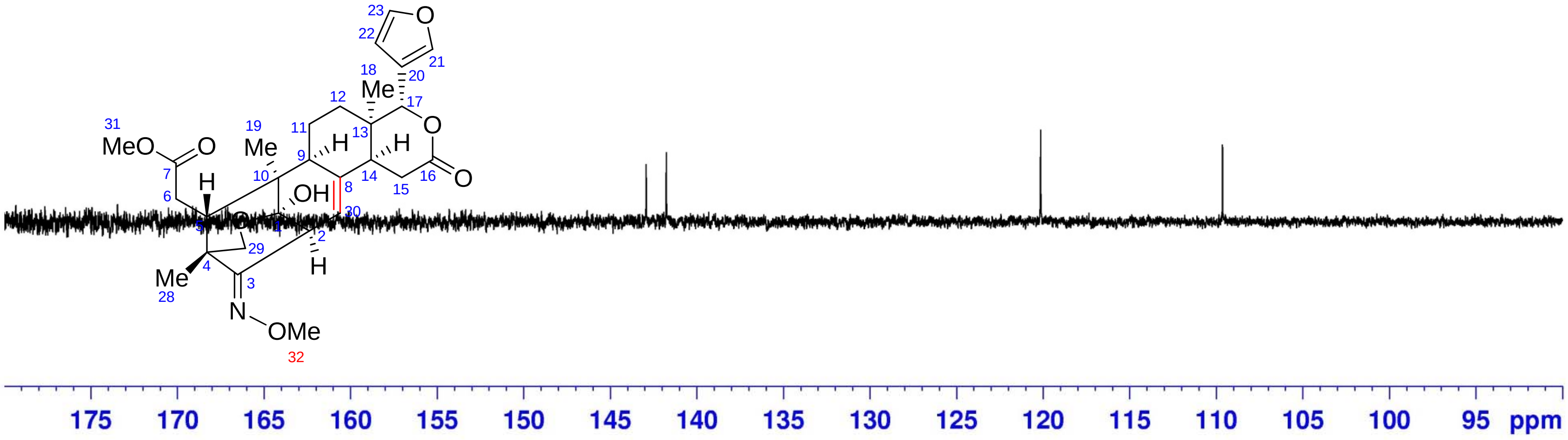

DEPT 135 spectrum of **9b** in $CDCl_3$

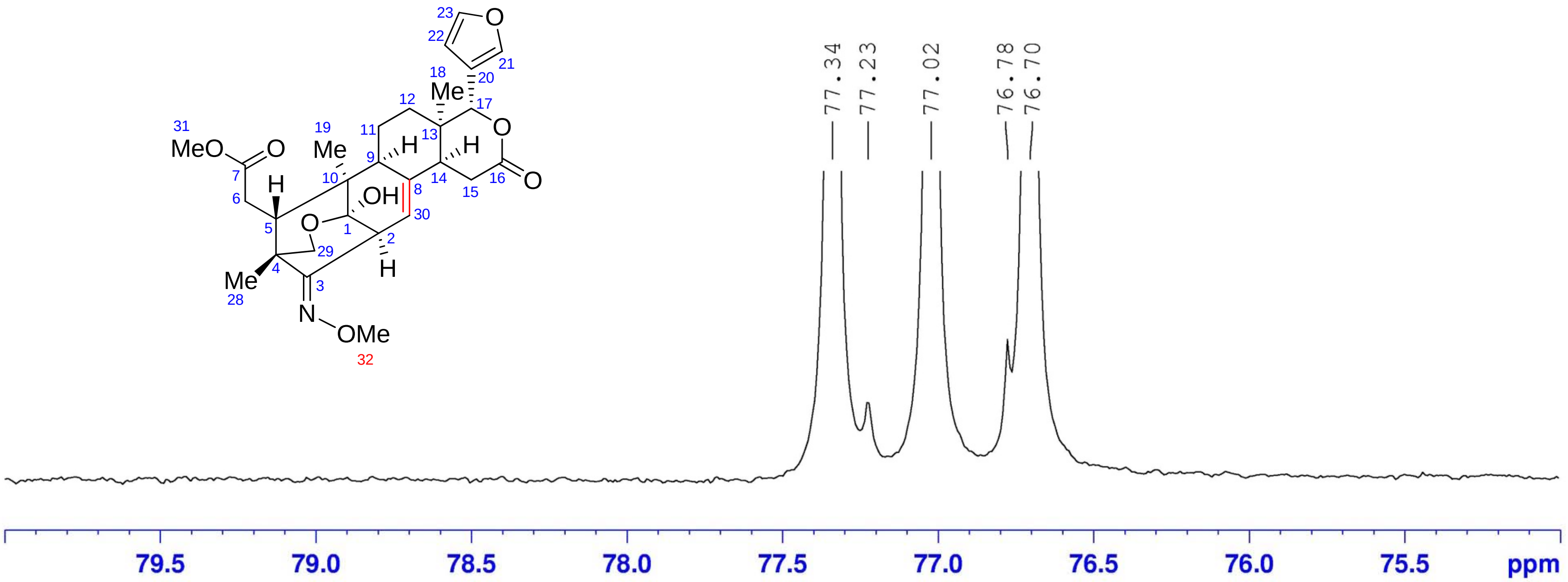

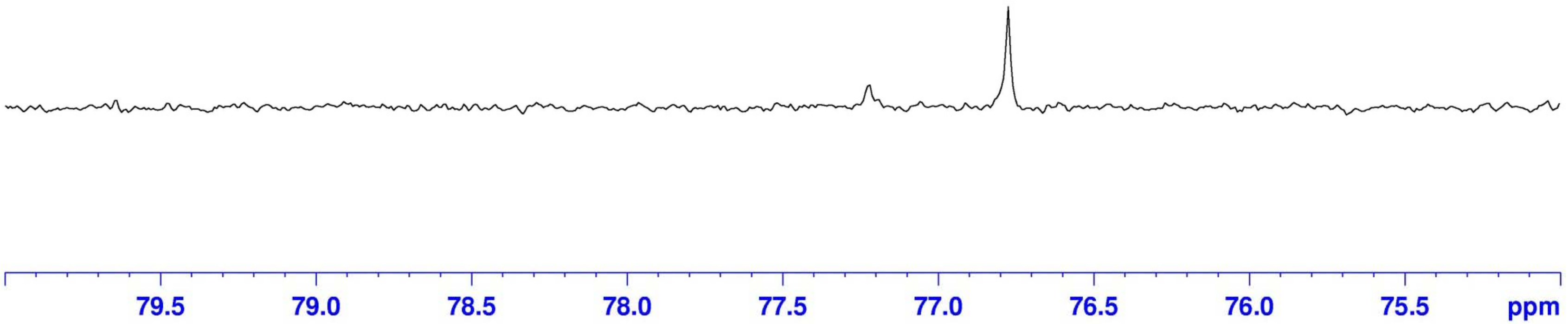

DEPT 135 spectrum of **9b** in $CDCl_3$

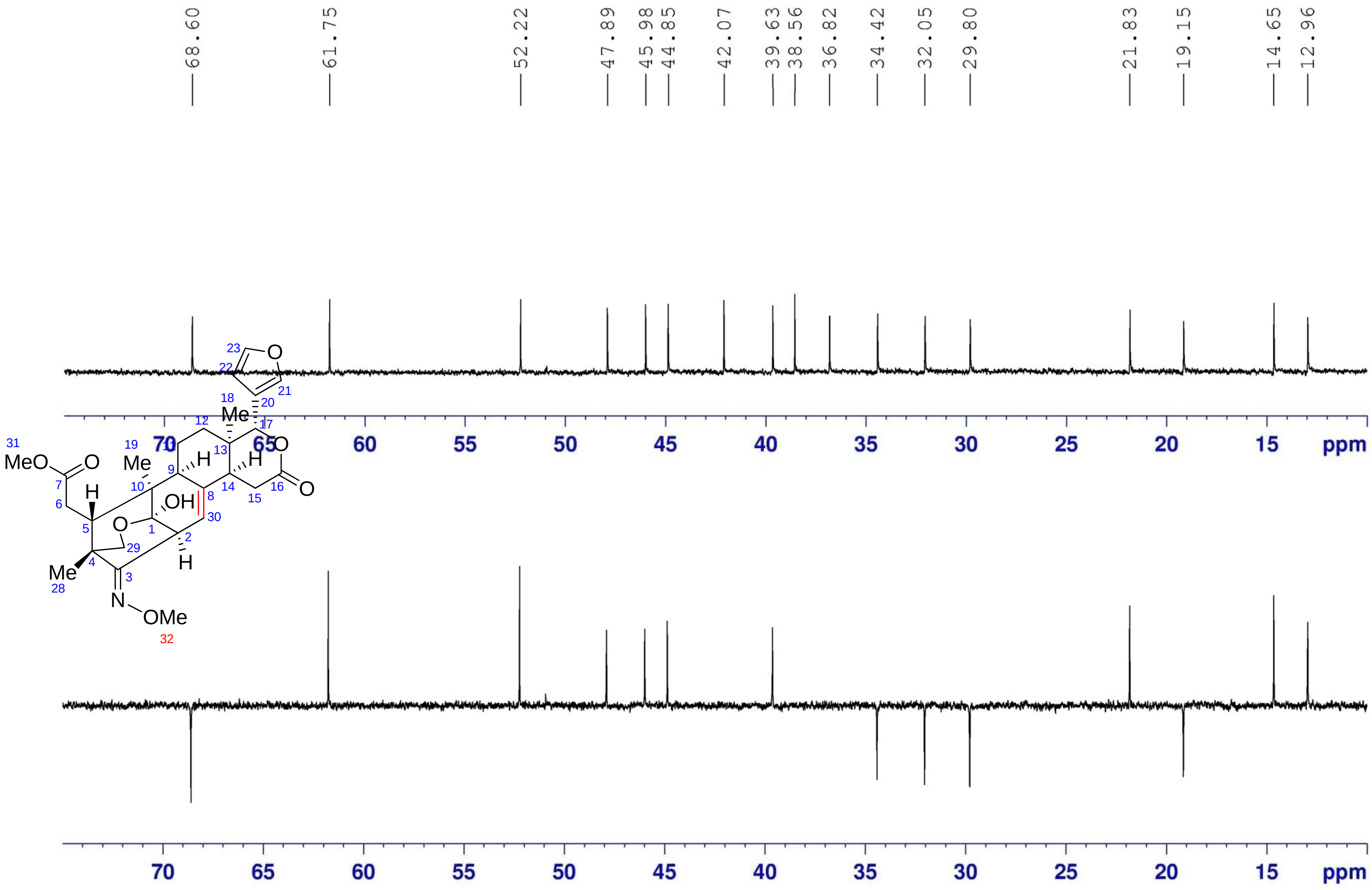

LR-ESIMS for compound **10**

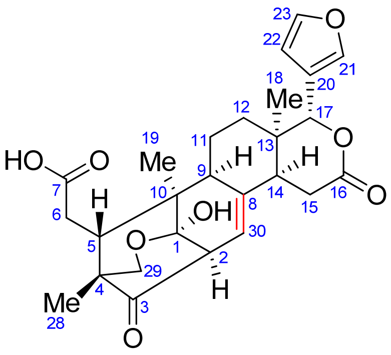

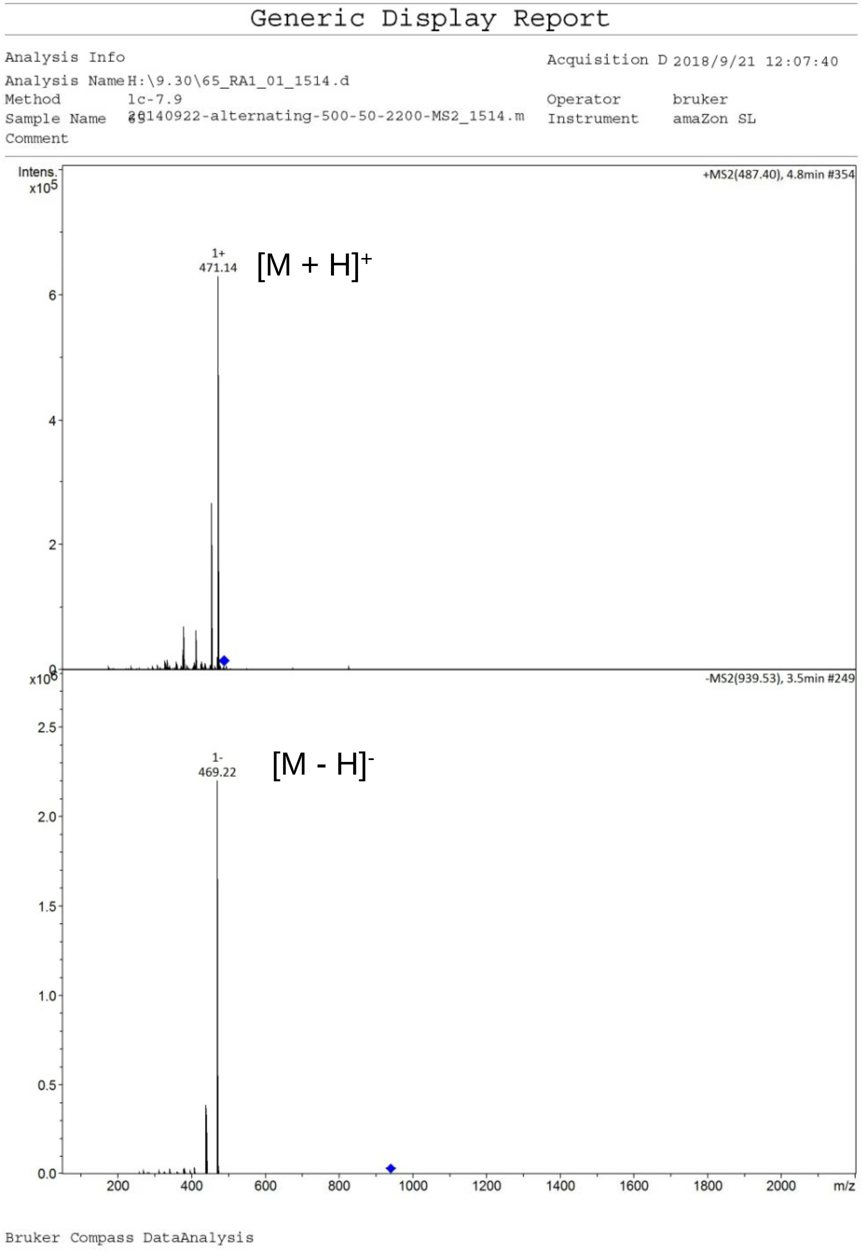

^{1}H NMR (400 MHz) spectrum of **10** in $(CD_3)_2CO$

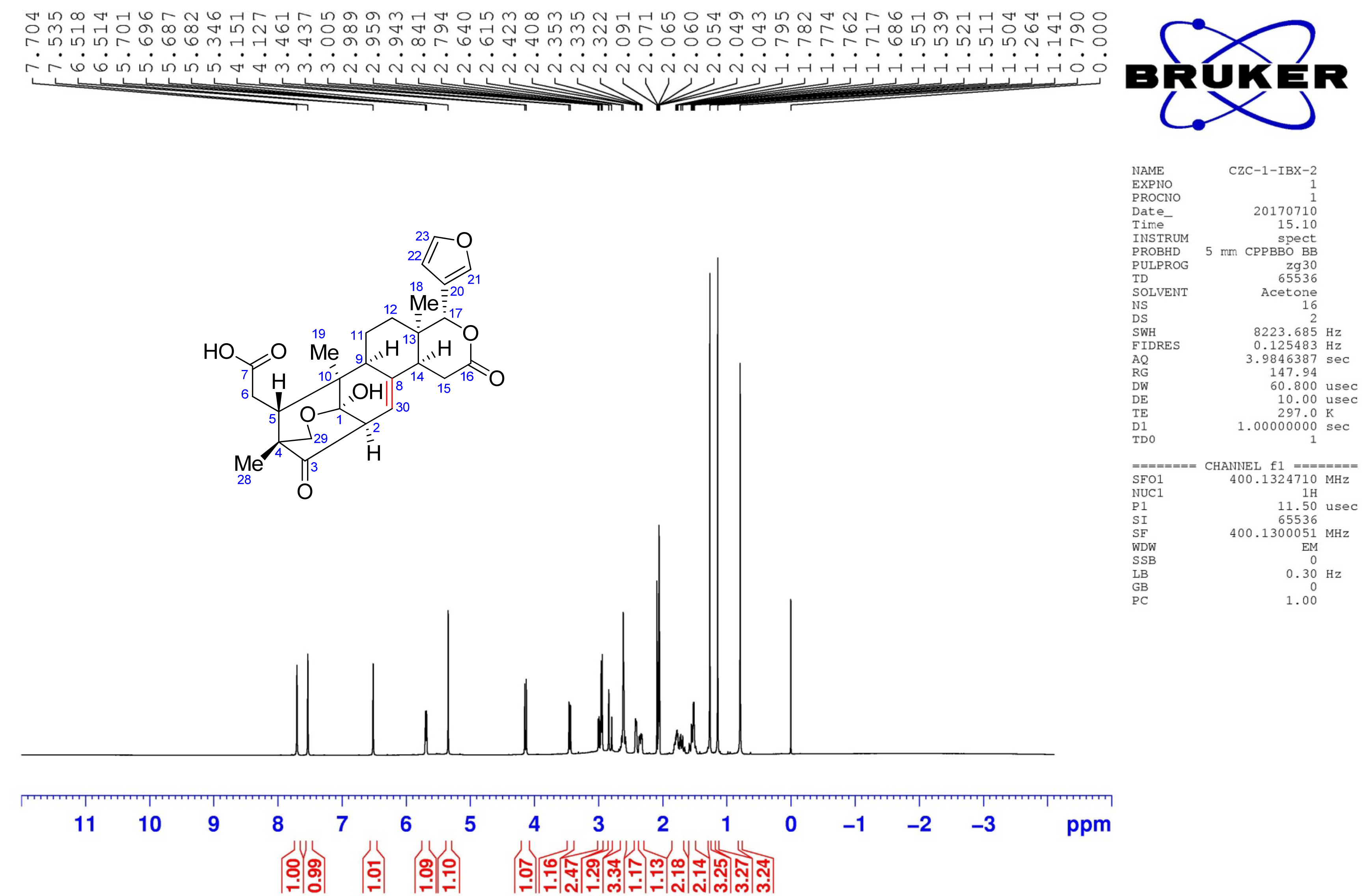

^{1}H NMR (400 MHz) spectrum of **10** in $(CD_3)_2CO$

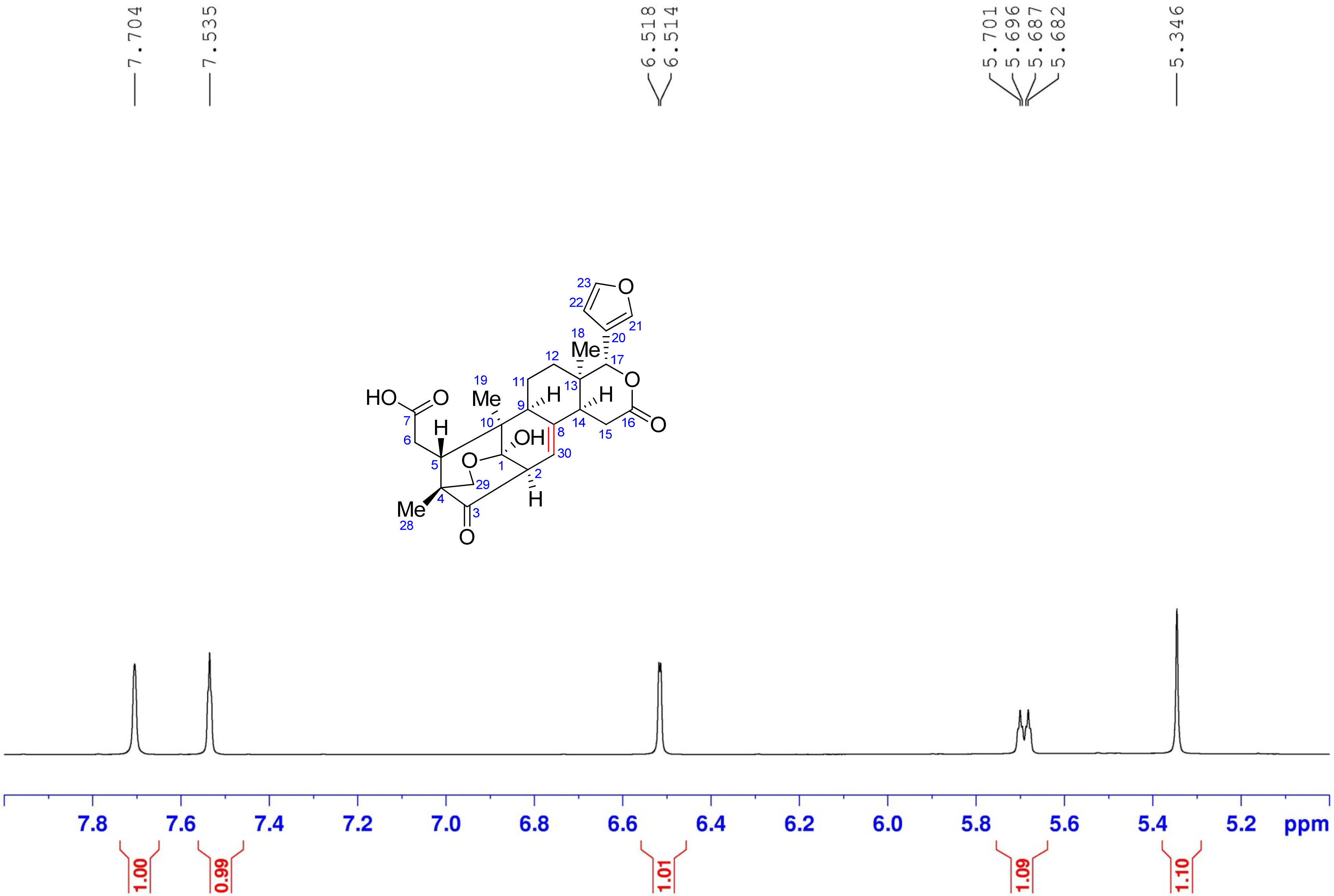

^{1}H NMR (400 MHz) spectrum of **10** in $(CD_3)_2CO$

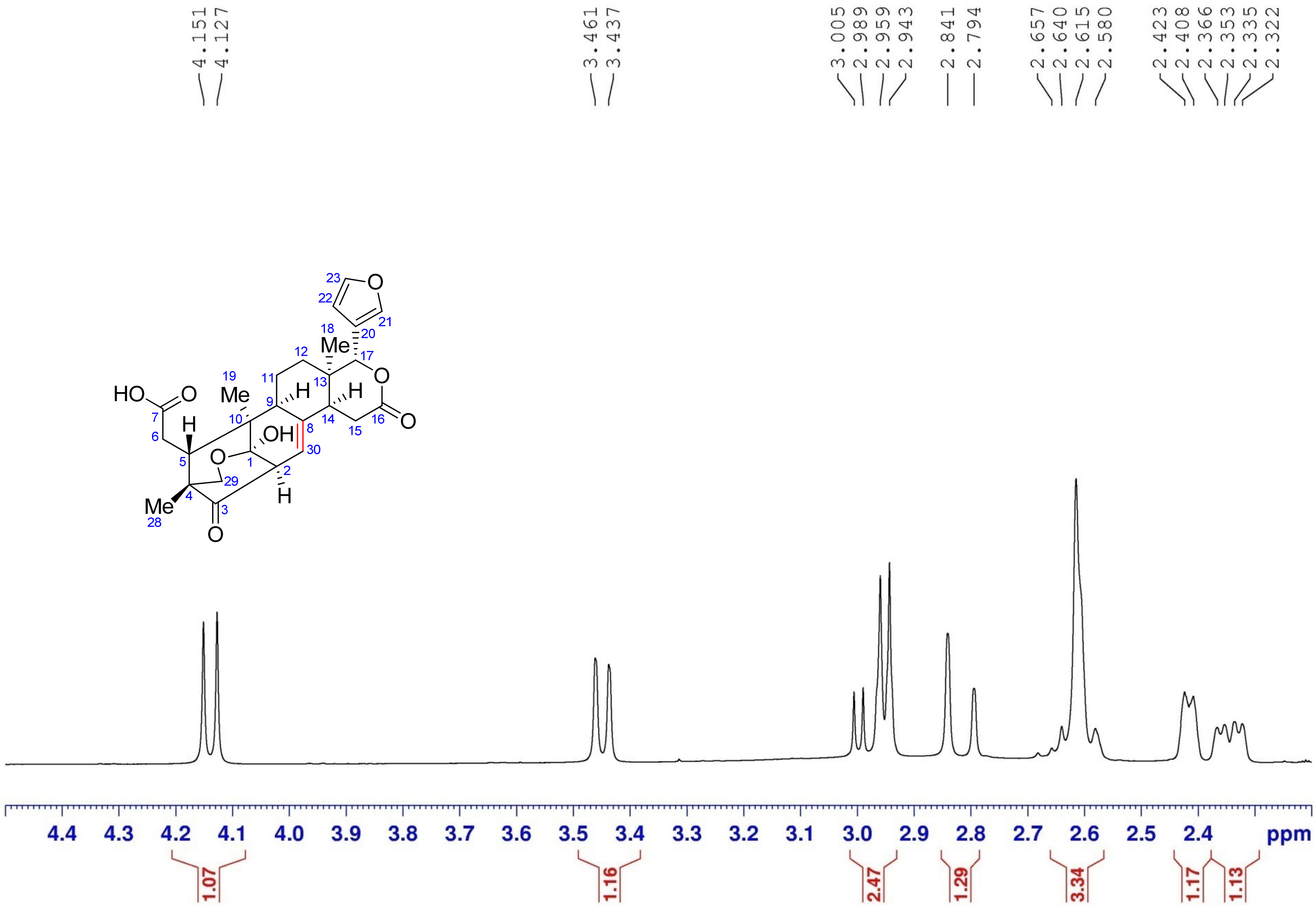

^{1}H NMR (400 MHz) spectrum of **10** in $(CD_3)_2CO$

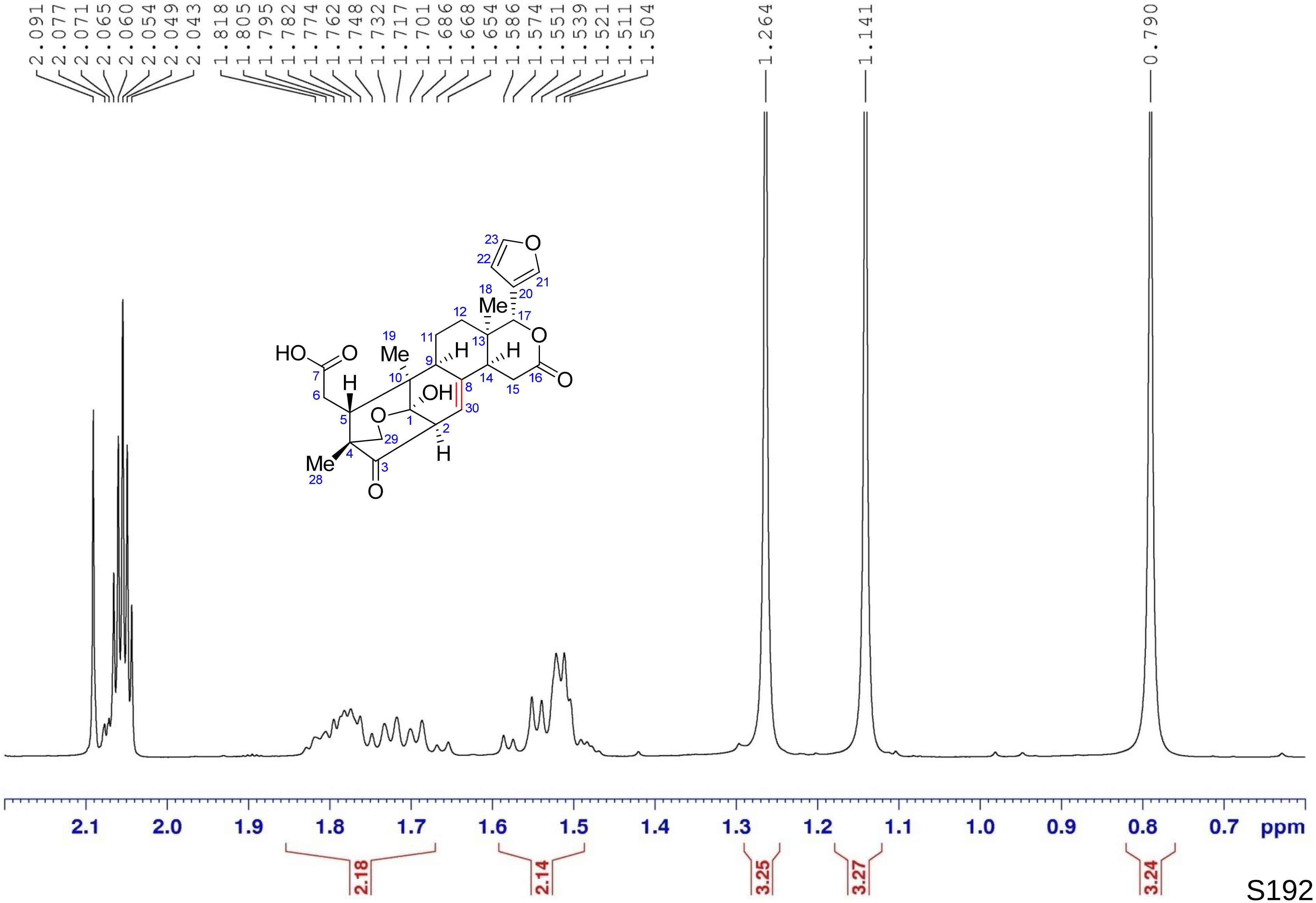

^{13}C NMR (100 MHz) spectrum of **10** in $(CD_3)_2CO$

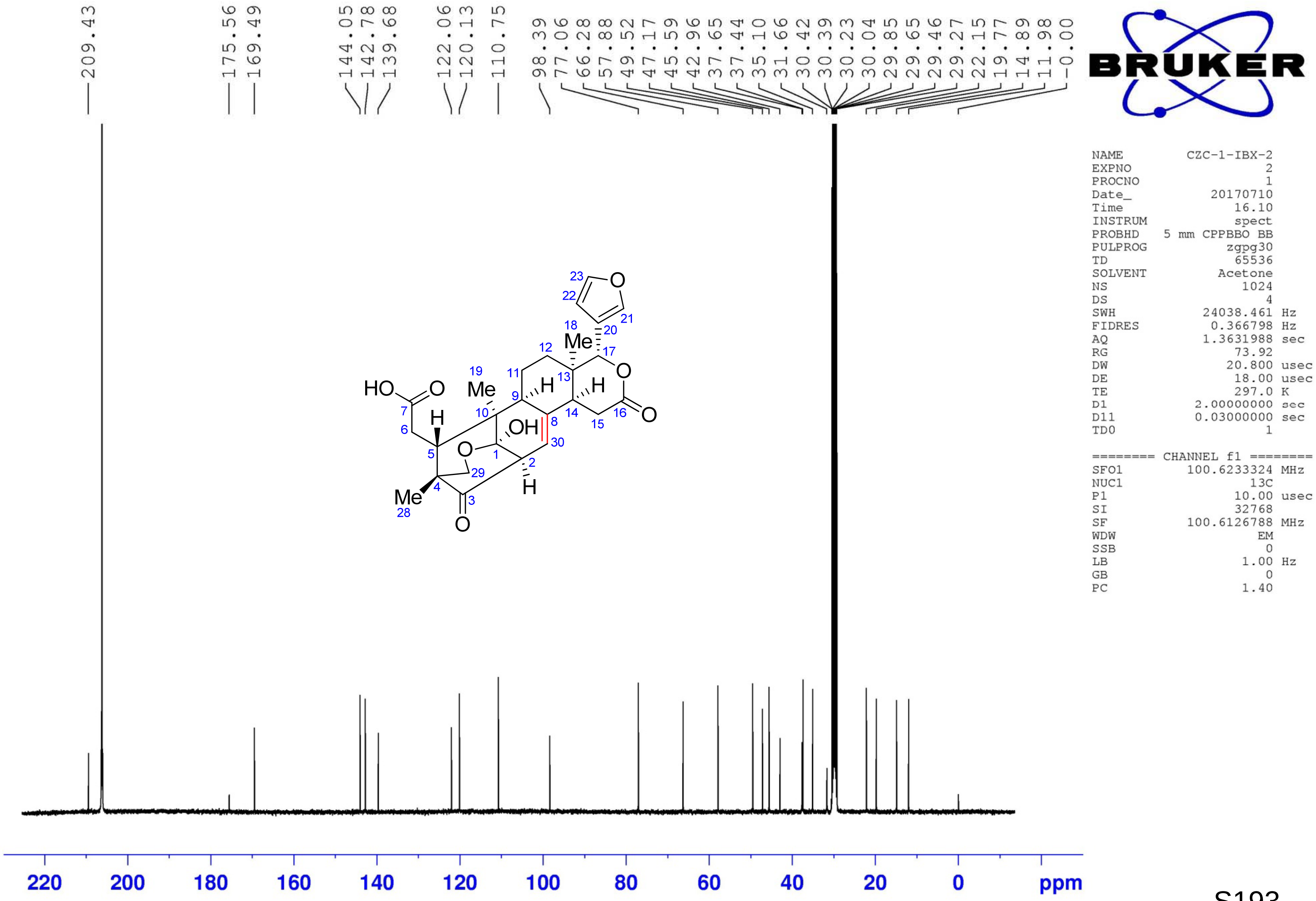

^{13}C NMR (100 MHz) spectrum of **10** in $(CD_3)_2CO$

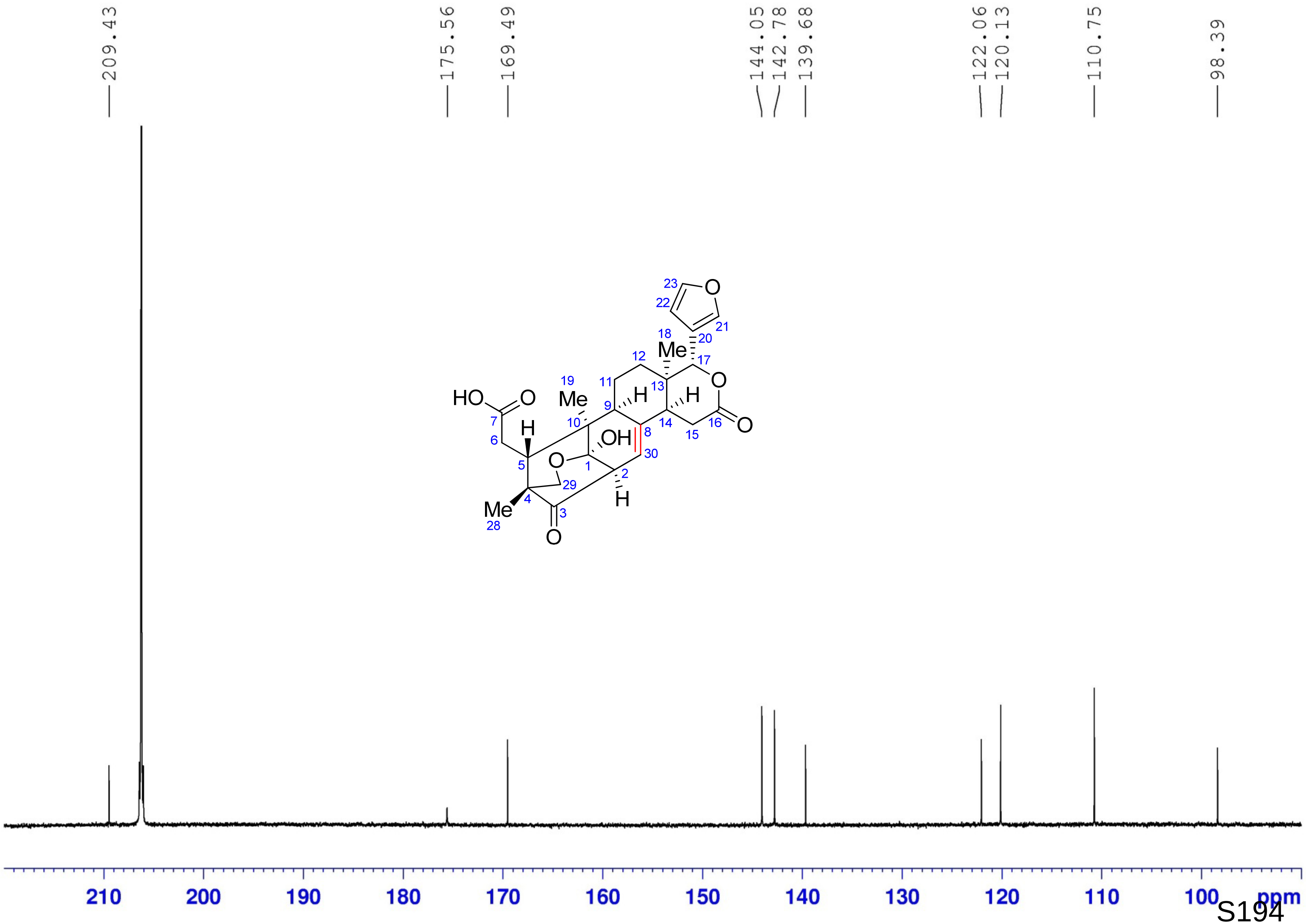

^{13}C NMR (100 MHz) spectrum of **10** in $(CD_3)_2CO$

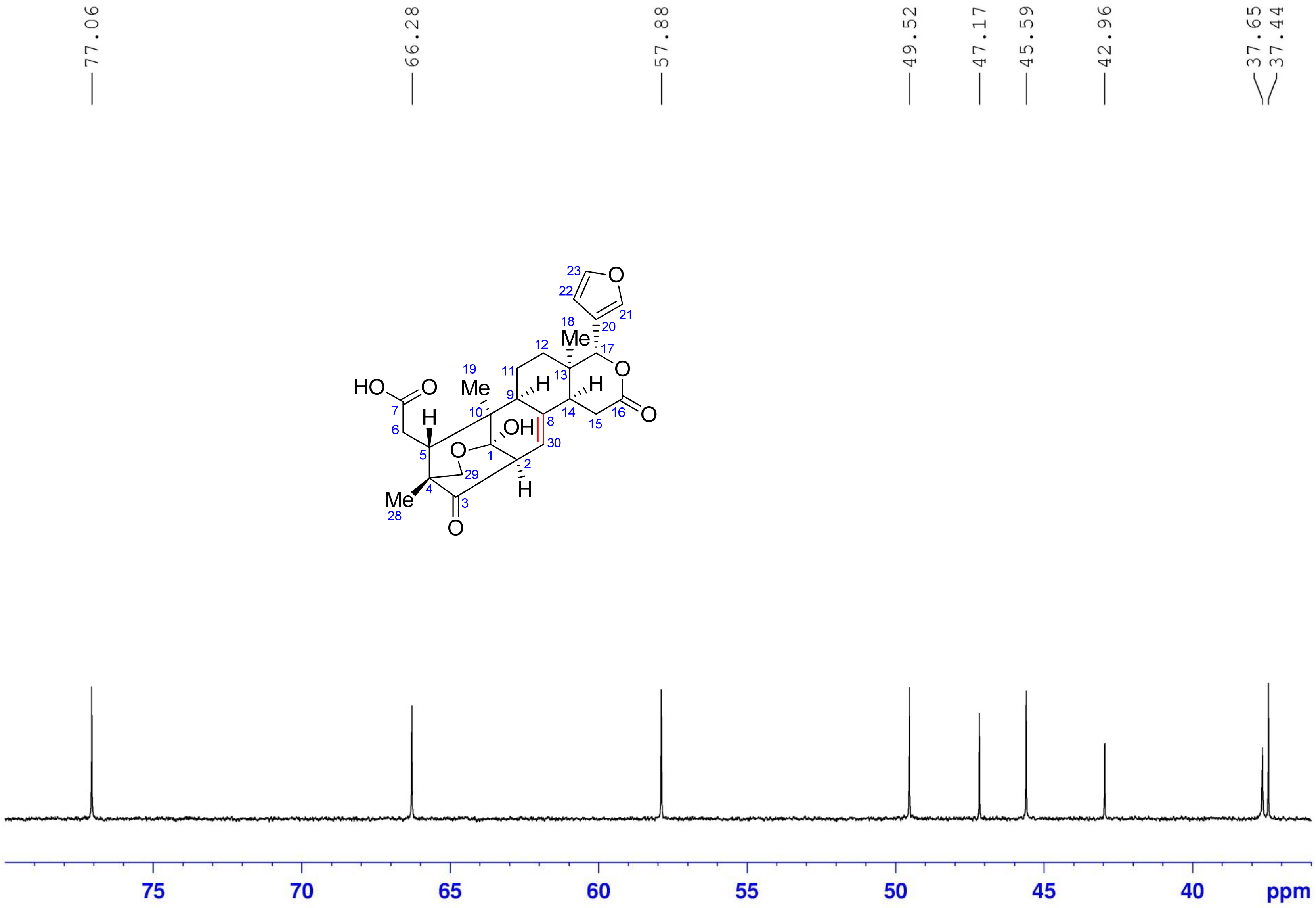

^{13}C NMR (100 MHz) spectrum of **10** in $(CD_3)_2CO$

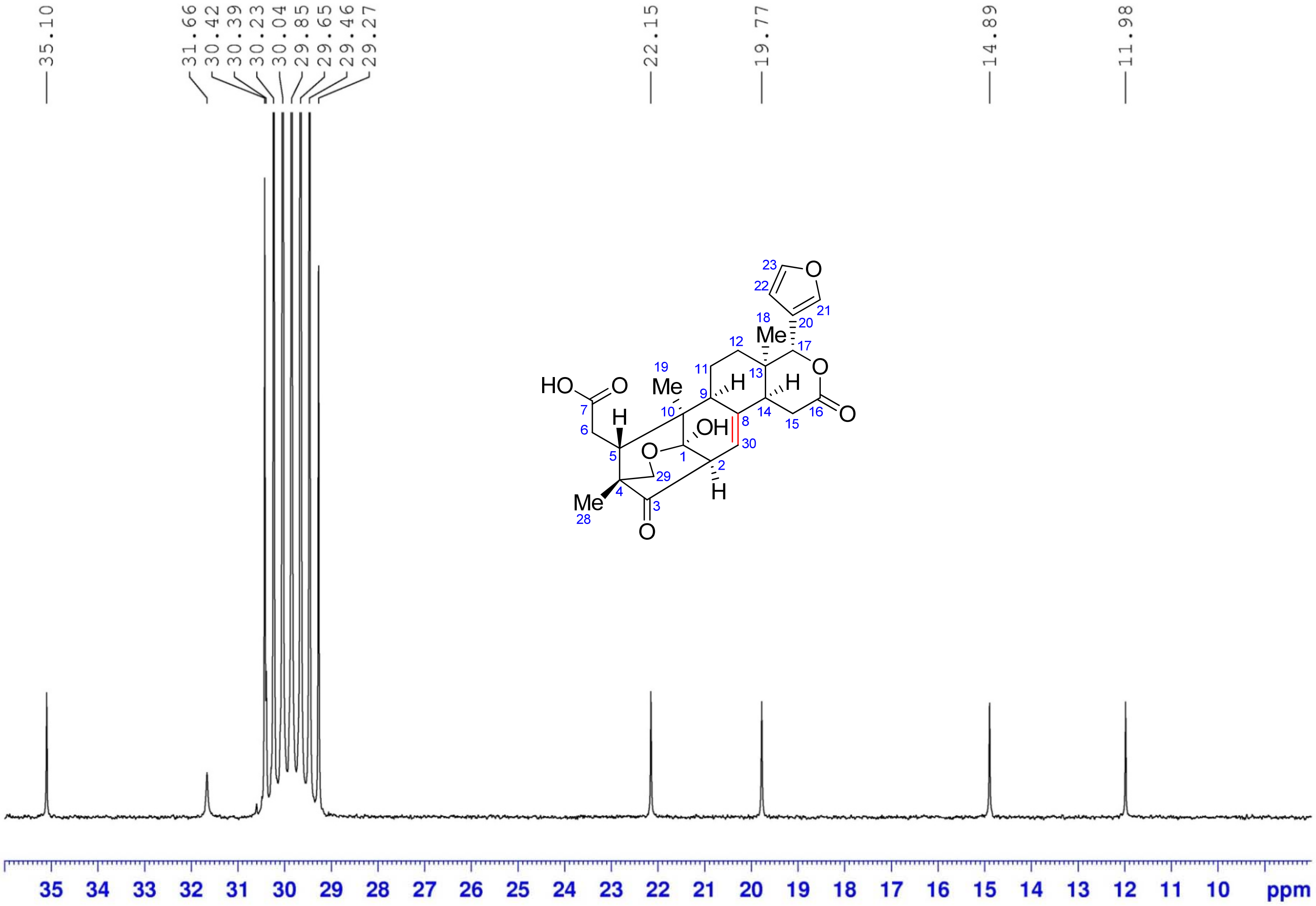

DEPT 135 spectrum of **10** in $(CD_3)_2CO$

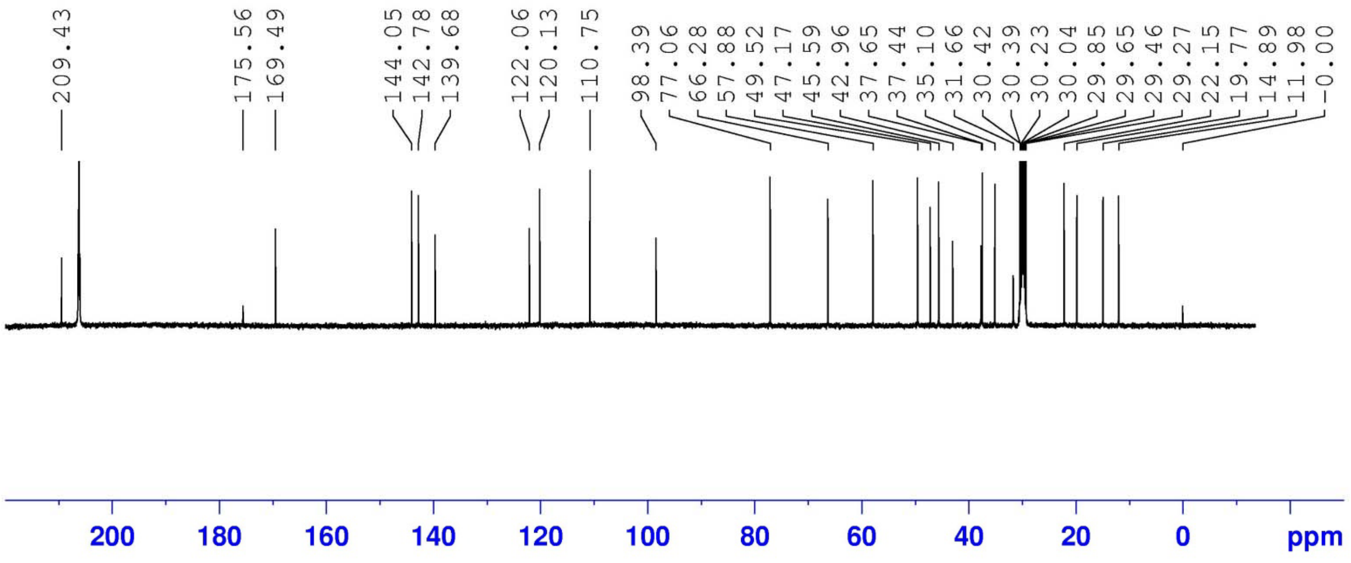

NAME	CZC-1-IBX-2
EXPNO	3
PROCNO	1
Date_	20170710
Time	16.28
INSTRUM	spect
PROBHD	5 mm CPPBBO BB
PULPROG	deptsp135
TD	65536
SOLVENT	Acetone
NS	300
DS	4
SWH	24038.461 Hz
FIDRES	0.366798 Hz
AQ	1.3631988 sec
RG	130.26
DW	20.800 usec
DE	18.00 usec
TE	297.0 K
CNST2	145.0000000
D1	2.00000000 sec
D2	0.00344828 sec
D12	0.00002000 sec
TD0	1
======== CHANNEL f1 ========	
SFO1	100.6233324 MHz
NUC1	13C
P1	10.00 usec
P13	2000.00 usec
SI	32768
SF	100.6126794 MHz
WDW	EM
SSB	0
LB	1.00 Hz
GB	0
PC	1.40

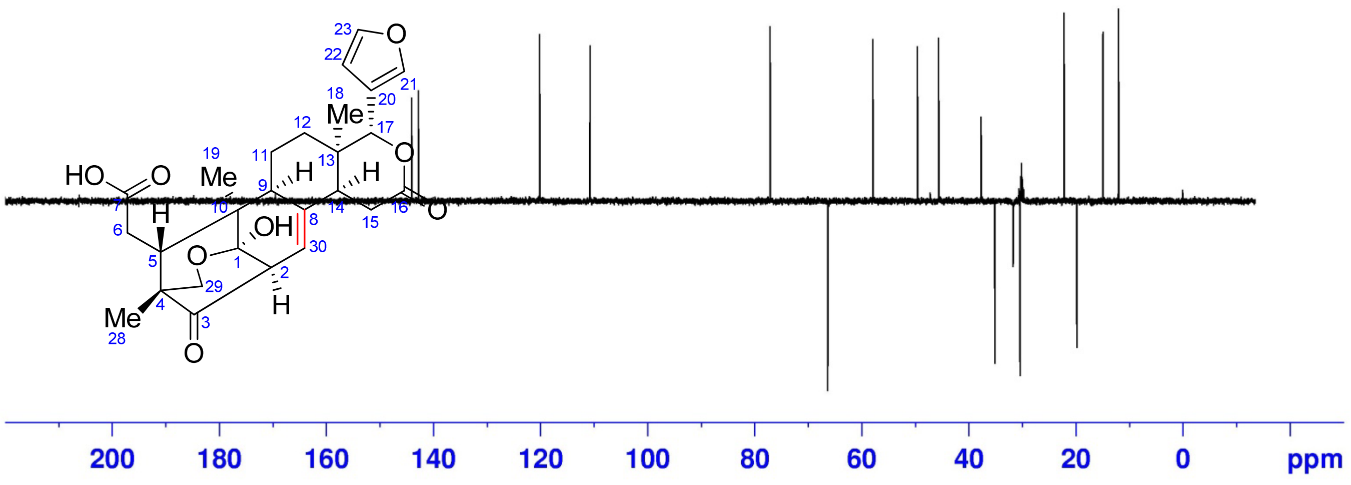

DEPT 135 spectrum of **10** in $(CD_3)_2CO$

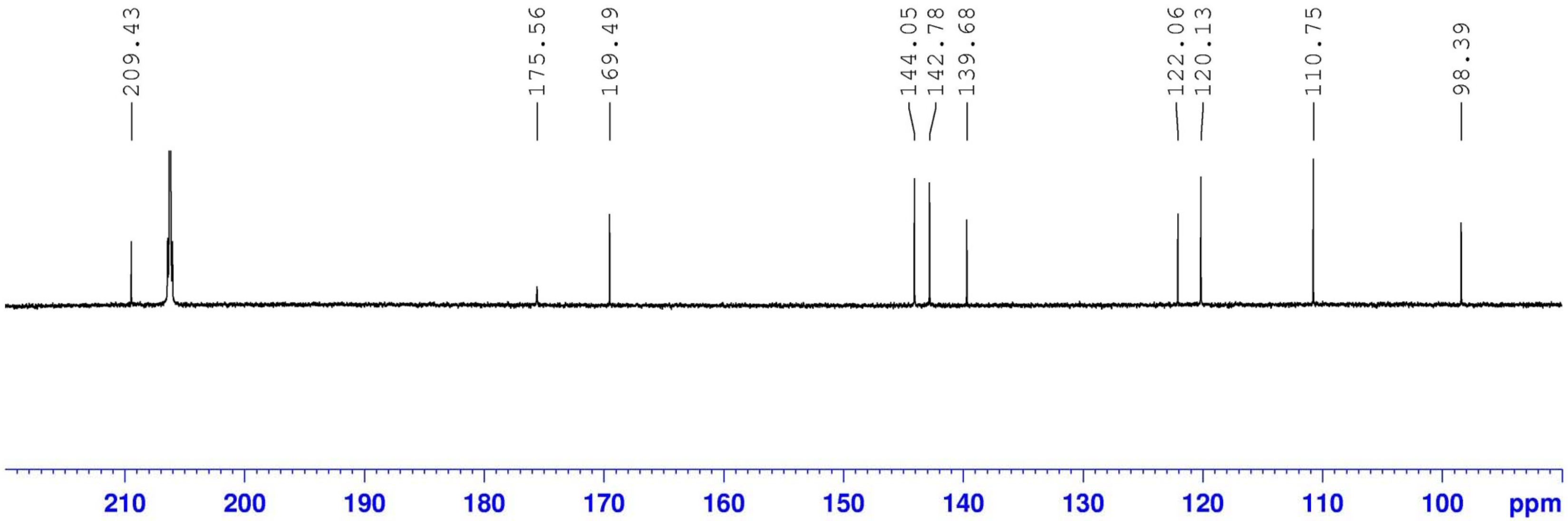

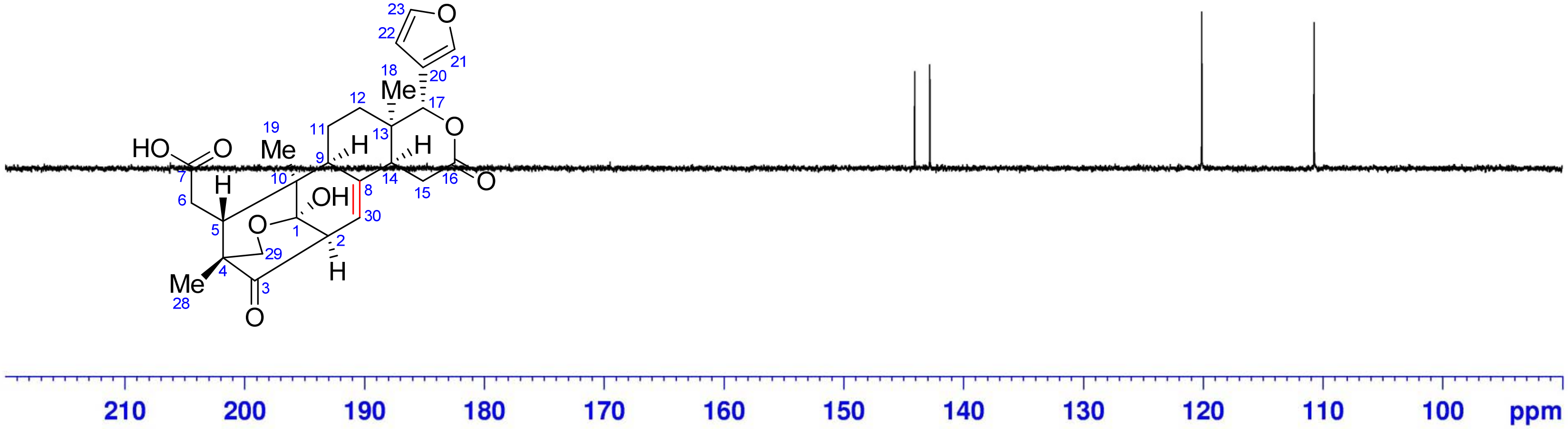

DEPT 135 spectrum of **10** in $(CD_3)_2CO$

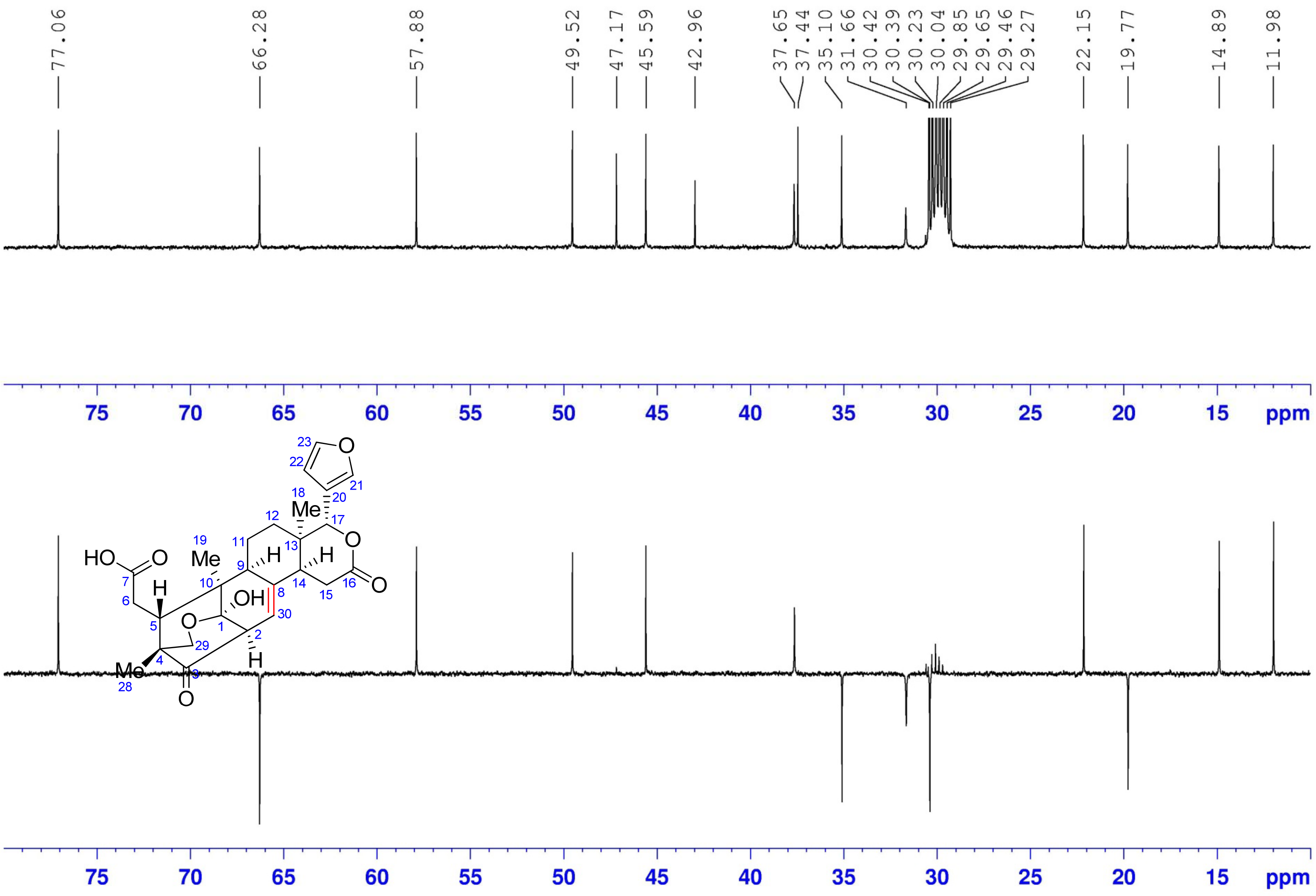

LR-ESIMS for compound **10a**

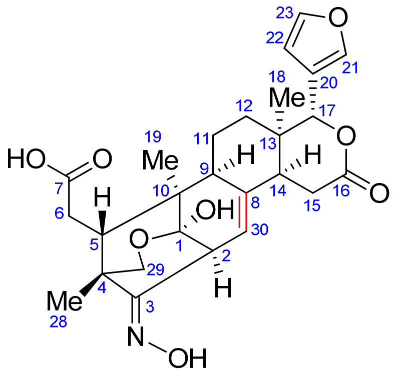

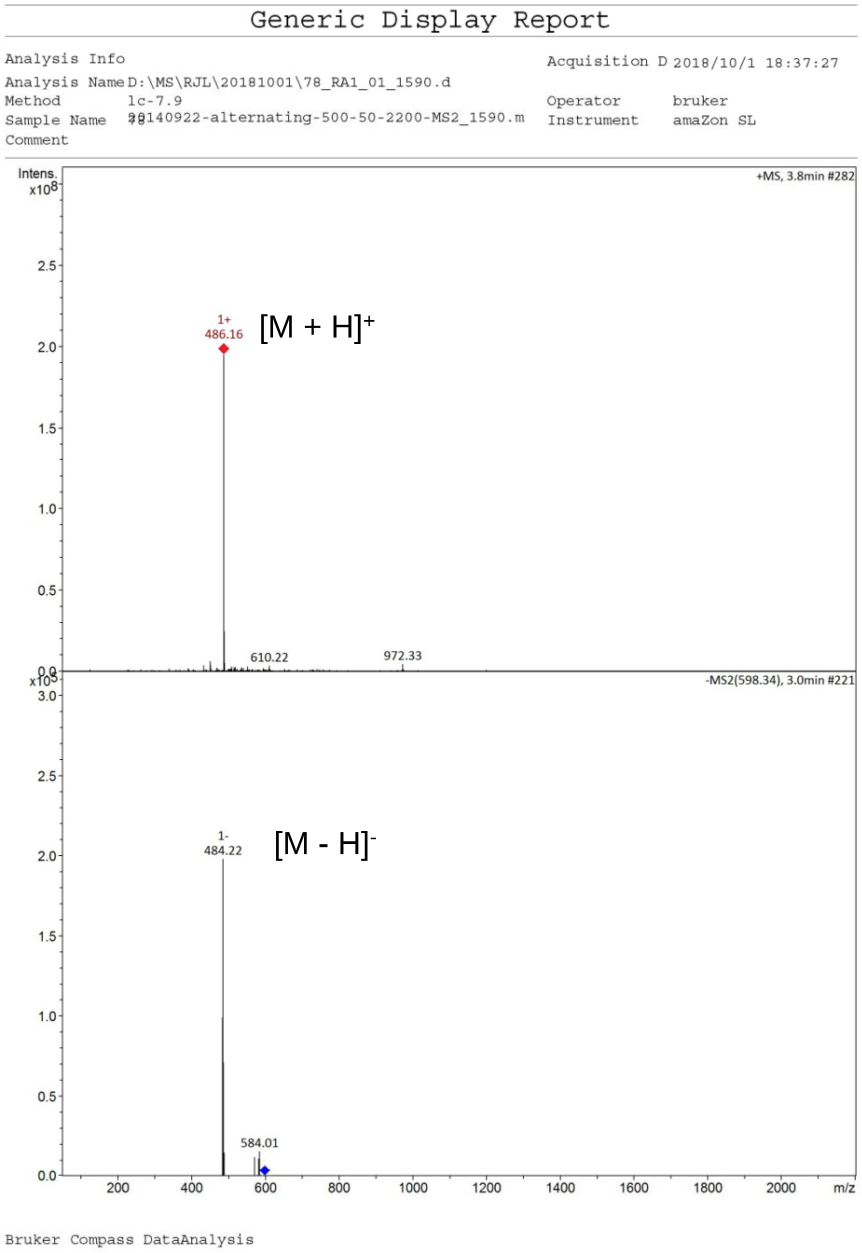

^{1}H NMR (400 MHz) spectrum of **10a** in $(CD_3)_2CO$

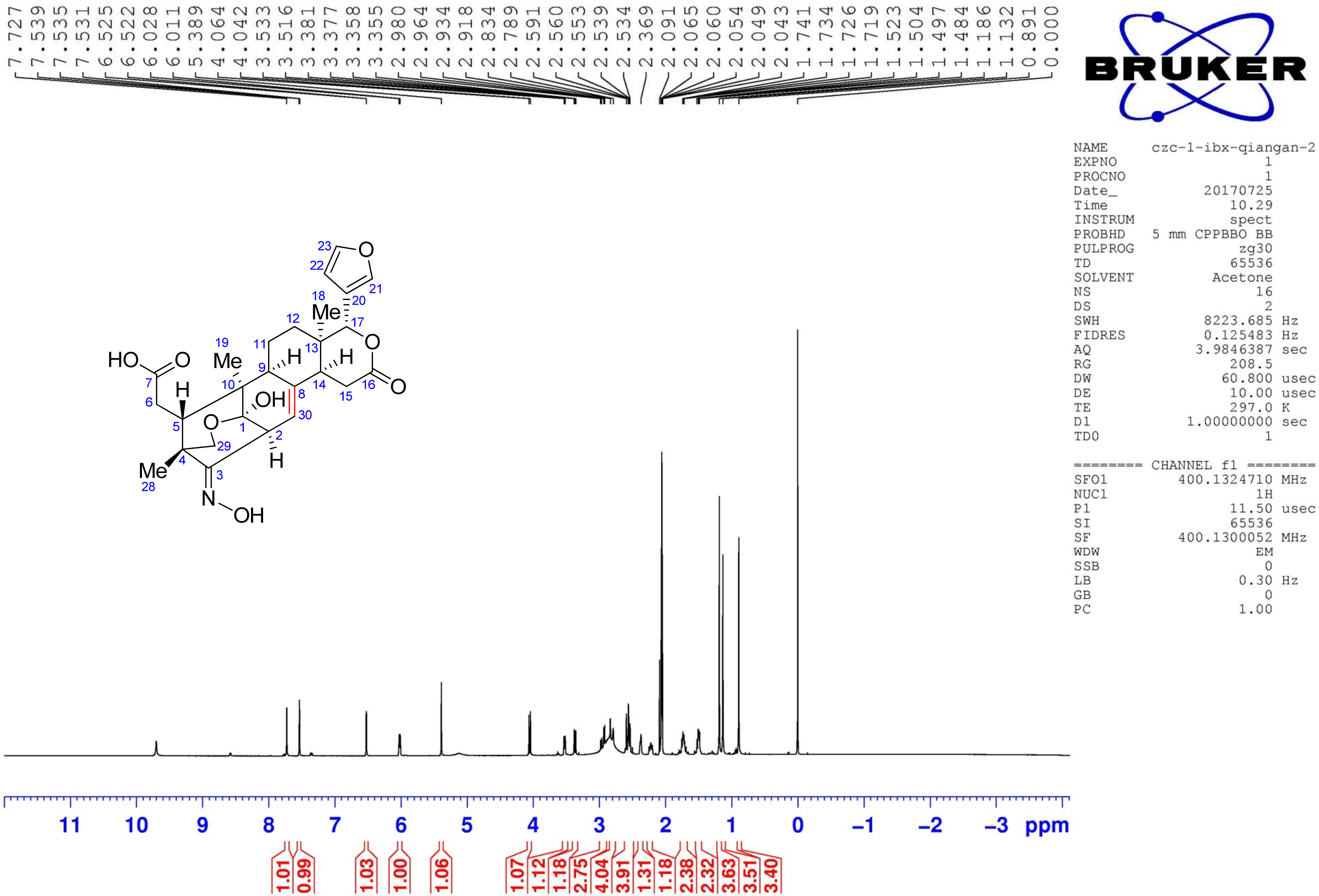

^{1}H NMR (400 MHz) spectrum of **10a** in $(CD_3)_2CO$

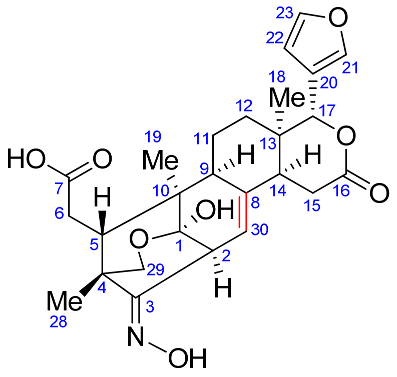

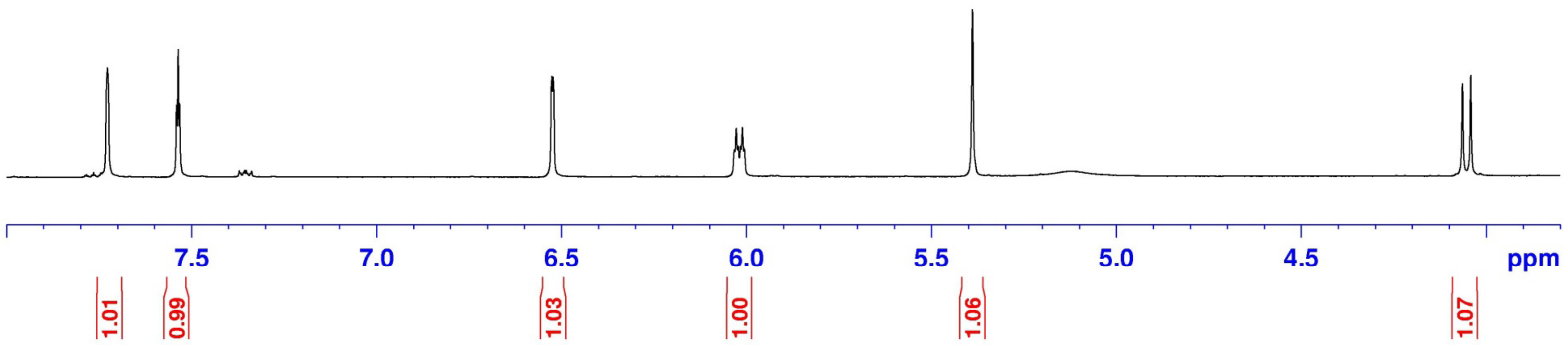

^{1}H NMR (400 MHz) spectrum of **10a** in $(CD_3)_2CO$

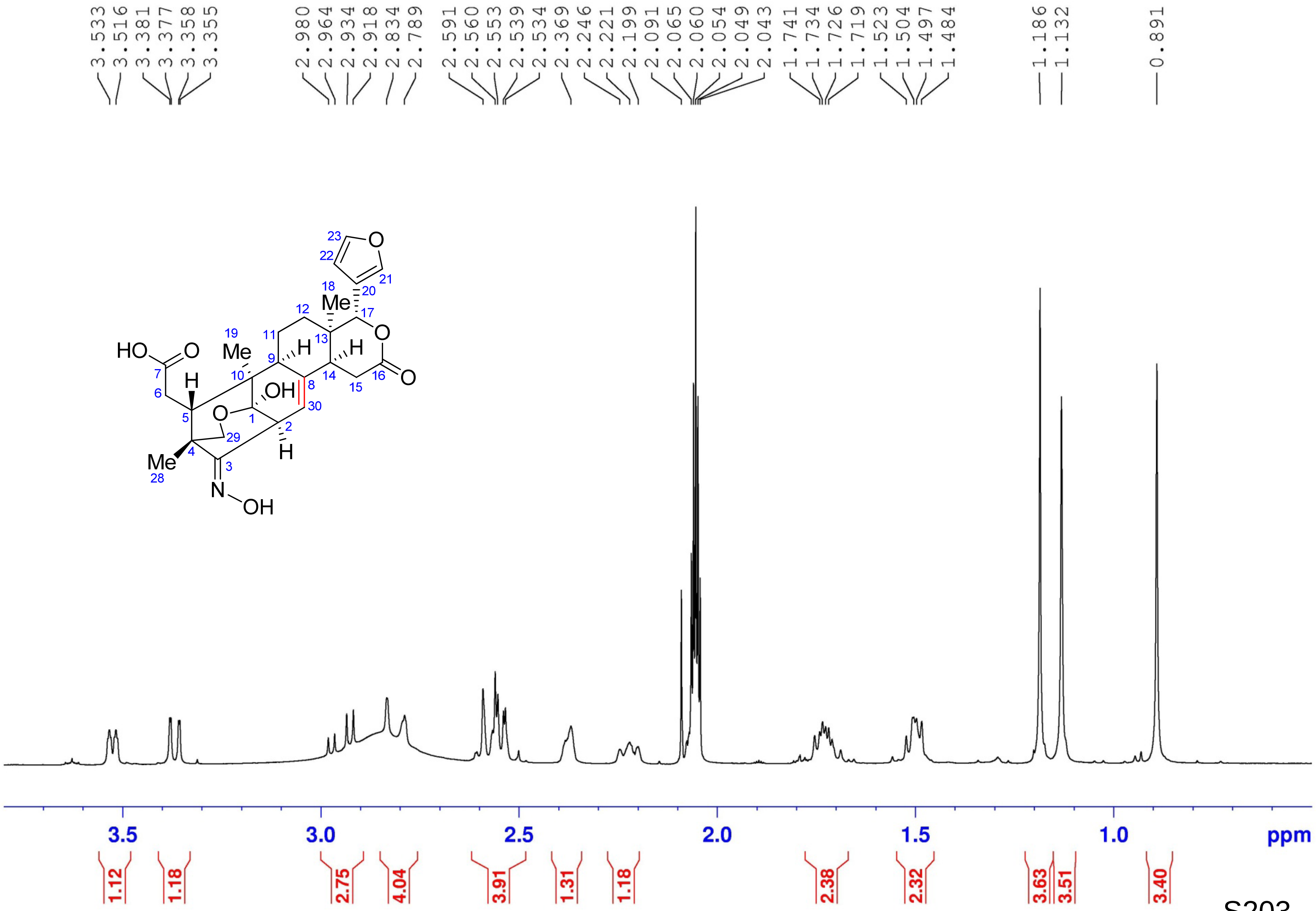

^{13}C NMR (100 MHz) spectrum of **10a** in $(CD_3)_2CO$

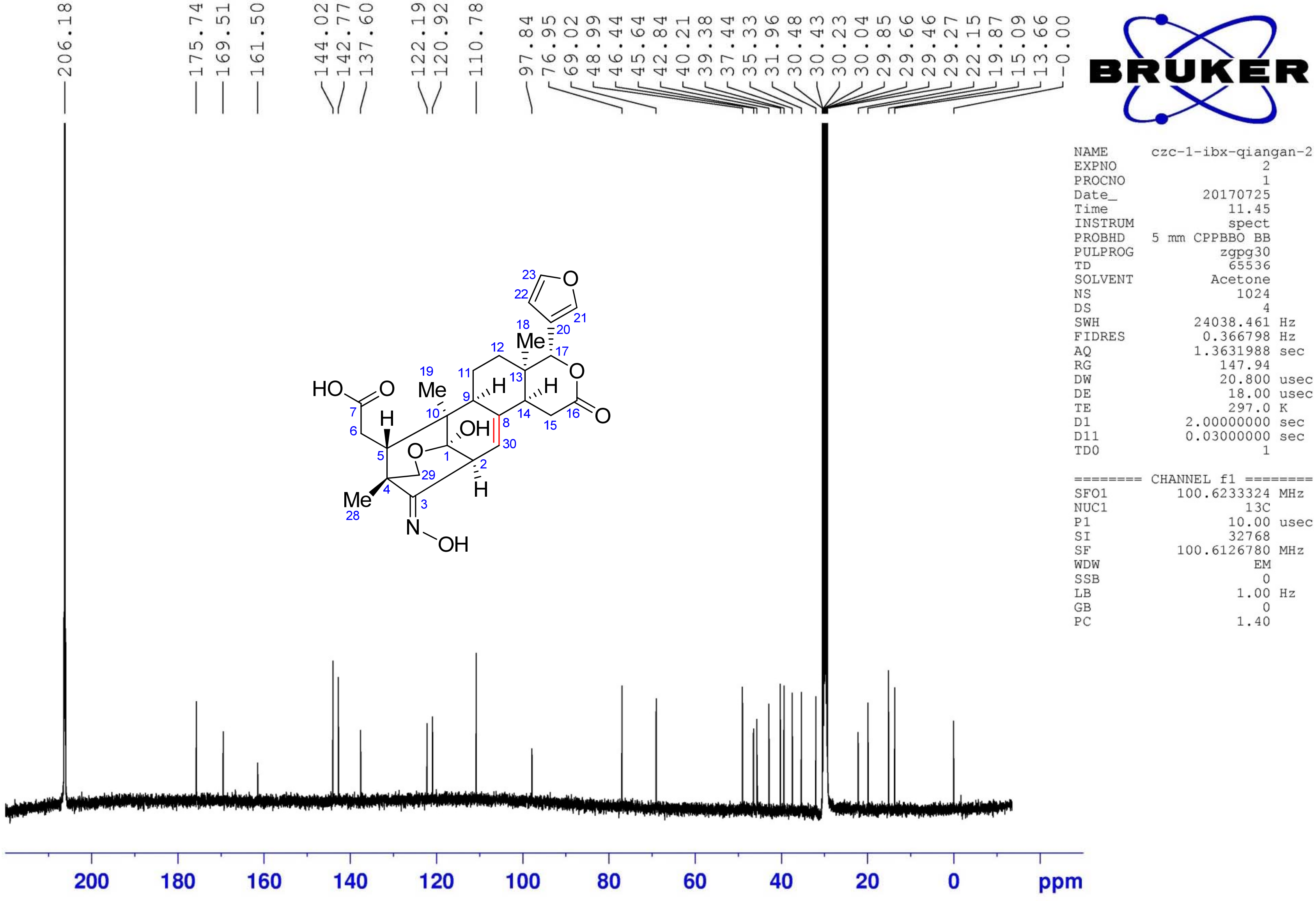

NAME	czc-1-ibx-qiangan-2
EXPNO	2
PROCNO	1
Date_	20170725
Time	11.45
INSTRUM	spect
PROBHD	5 mm CPPBBO BB
PULPROG	zgpg30
TD	65536
SOLVENT	Acetone
NS	1024
DS	4
SWH	24038.461 Hz
FIDRES	0.366798 Hz
AQ	1.3631988 sec
RG	147.94
DW	20.800 usec
DE	18.00 usec
TE	297.0 K
D1	2.00000000 sec
D11	0.03000000 sec
TD0	1

======== CHANNEL f1 ========

SFO1	100.6233324 MHz
NUC1	13C
P1	10.00 usec
SI	32768
SF	100.6126780 MHz
WDW	EM
SSB	0
LB	1.00 Hz
GB	0
PC	1.40

^{13}C NMR (100 MHz) spectrum of **10a** in $(CD_3)_2CO$

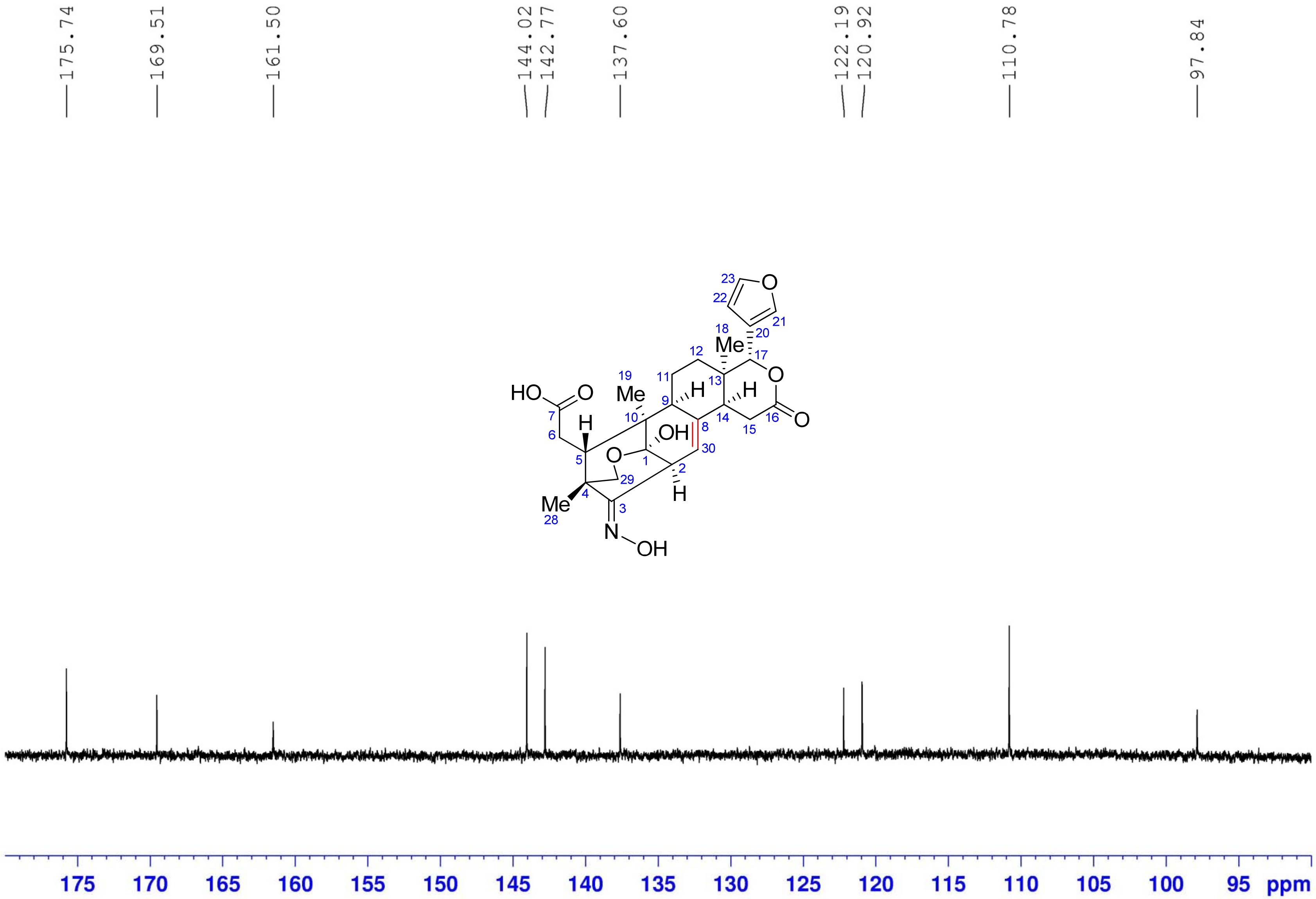

^{13}C NMR (100 MHz) spectrum of **10a** in $(CD_3)_2CO$

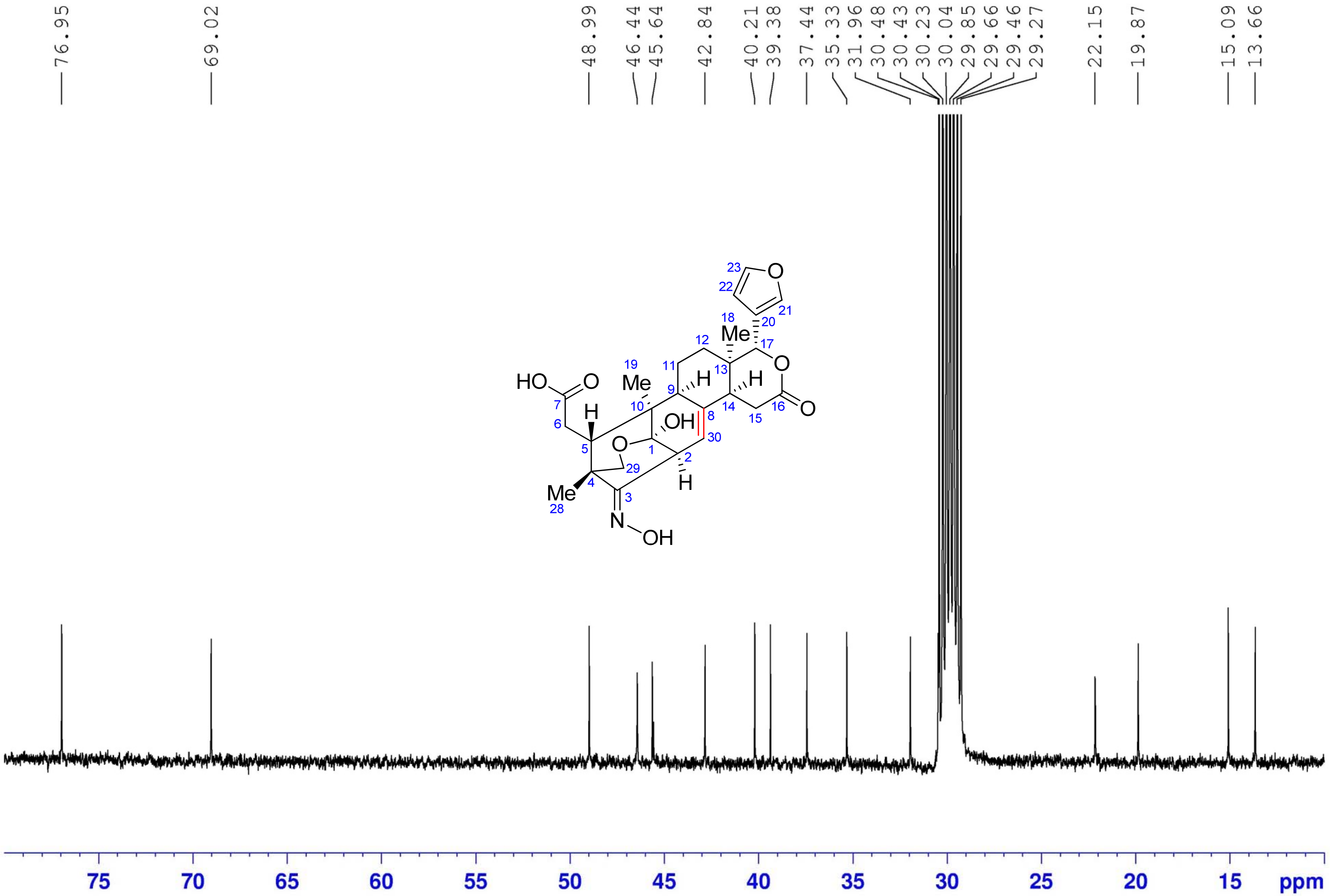

DEPT 135 spectrum of **10a** in $(CD_3)_2CO$

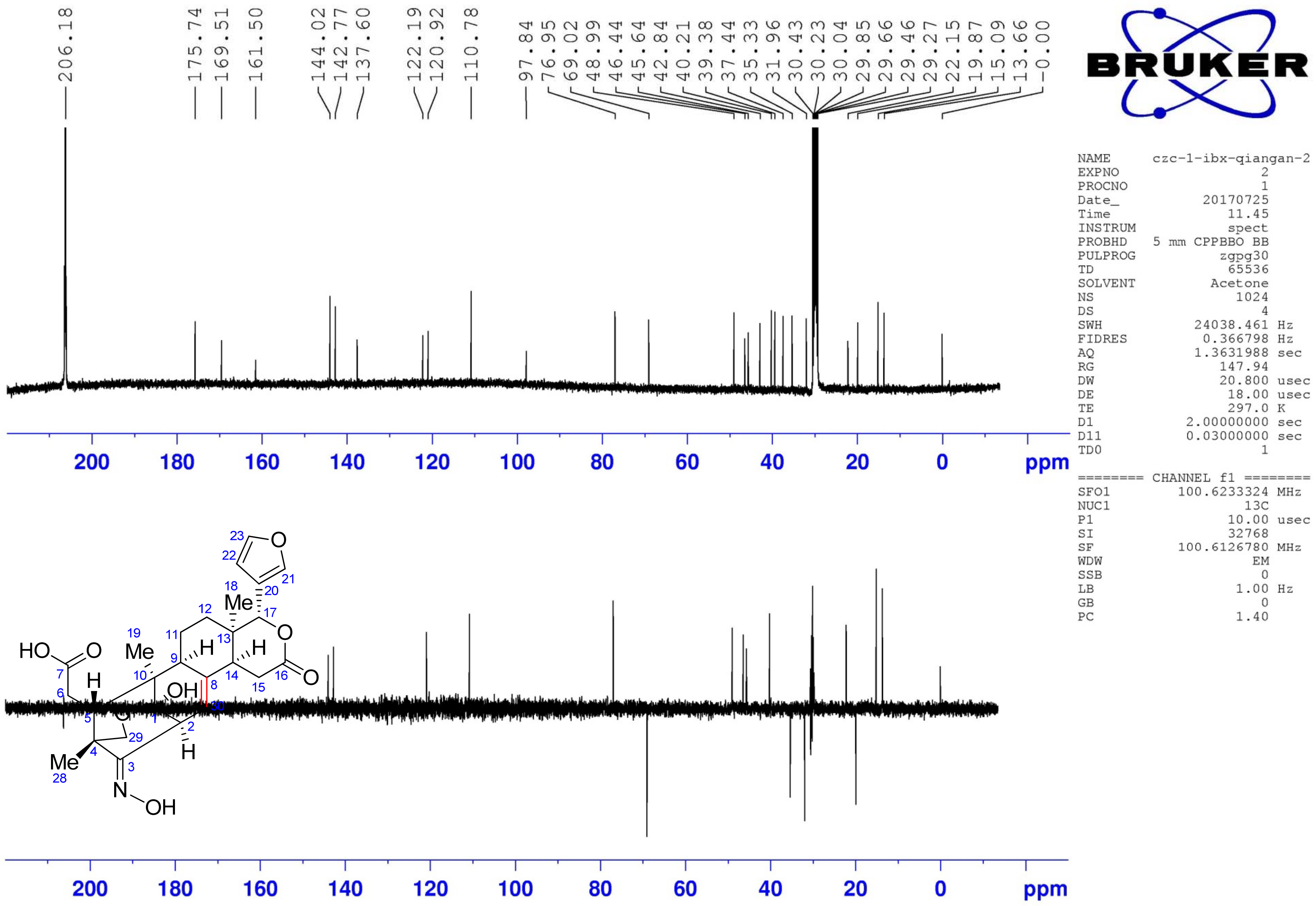

DEPT 135 spectrum of **10a** in $(CD_3)_2CO$

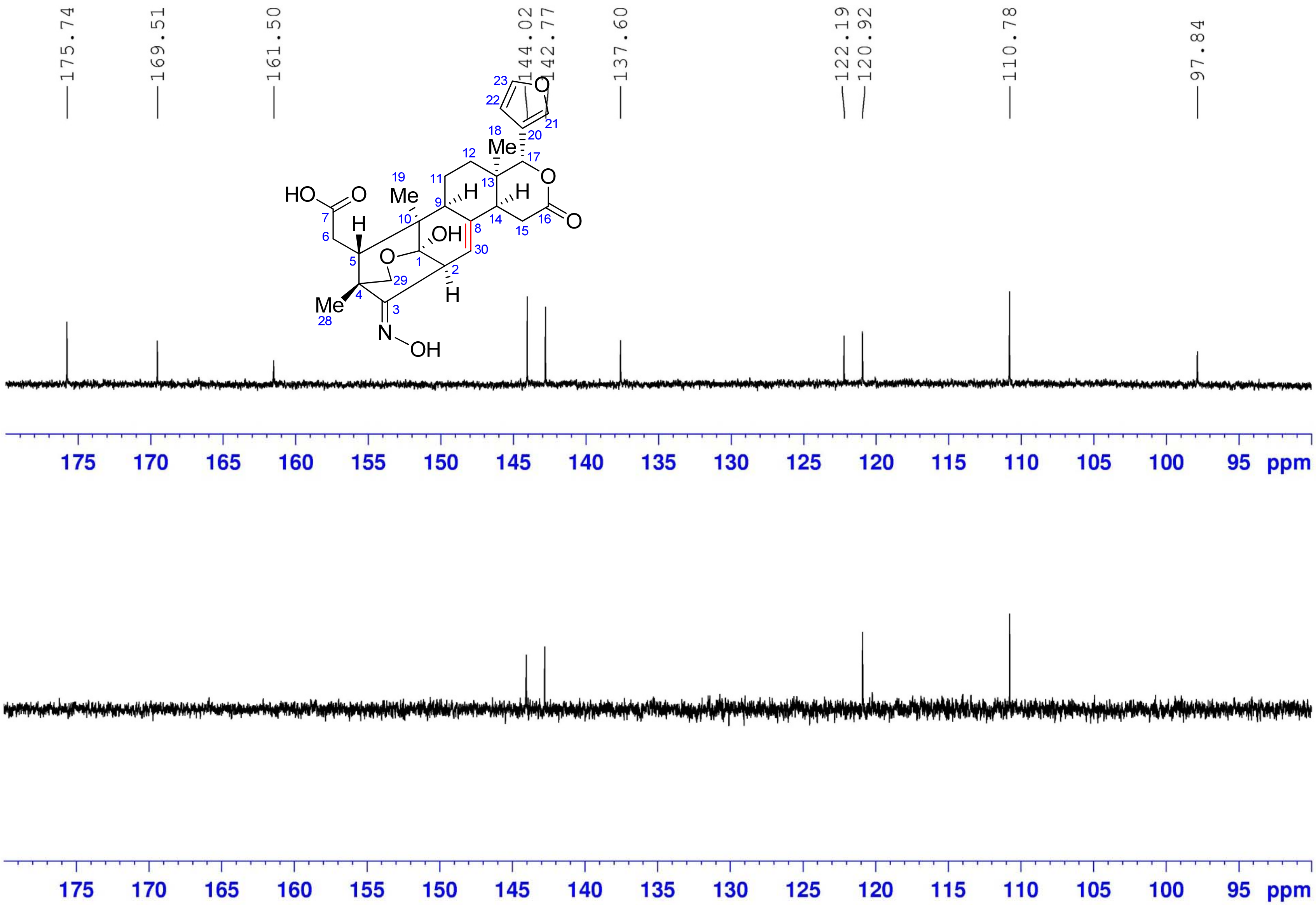

DEPT 135 spectrum of **10a** in $(CD_3)_2CO$

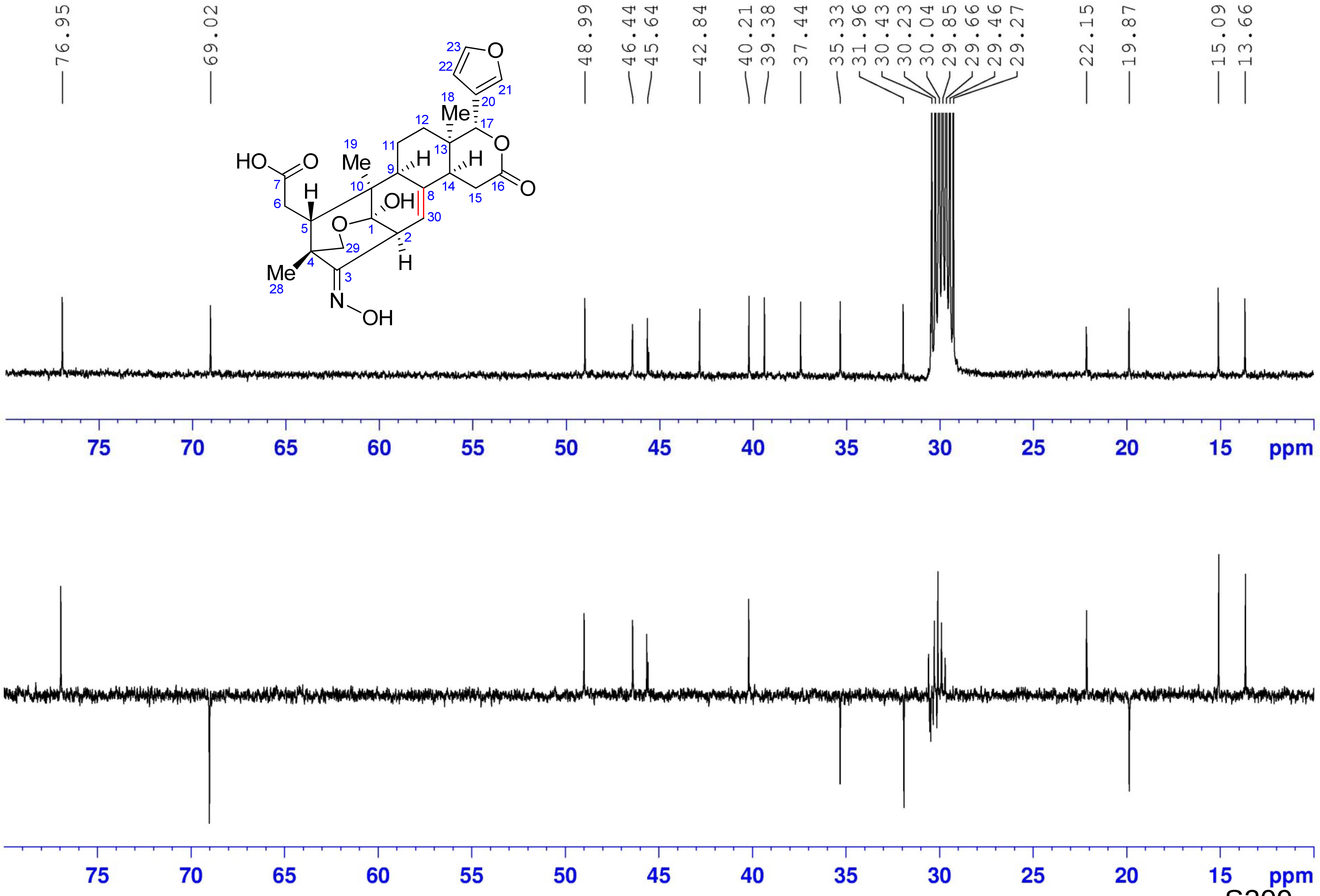

LR-ESIMS for compound **10b**

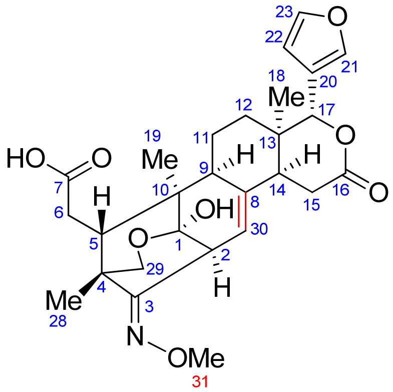

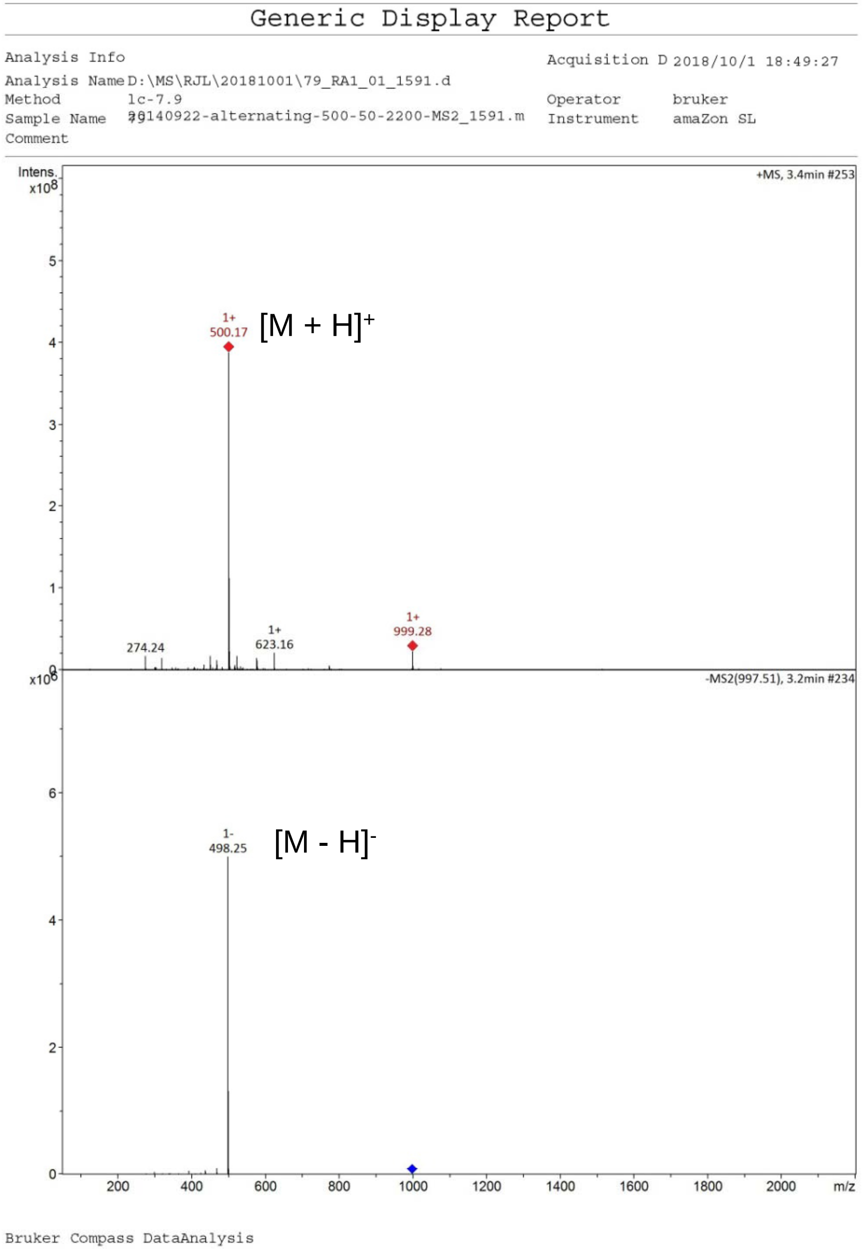

^{1}H NMR (400 MHz) spectrum of **10b** in $(CD_3)_2CO$

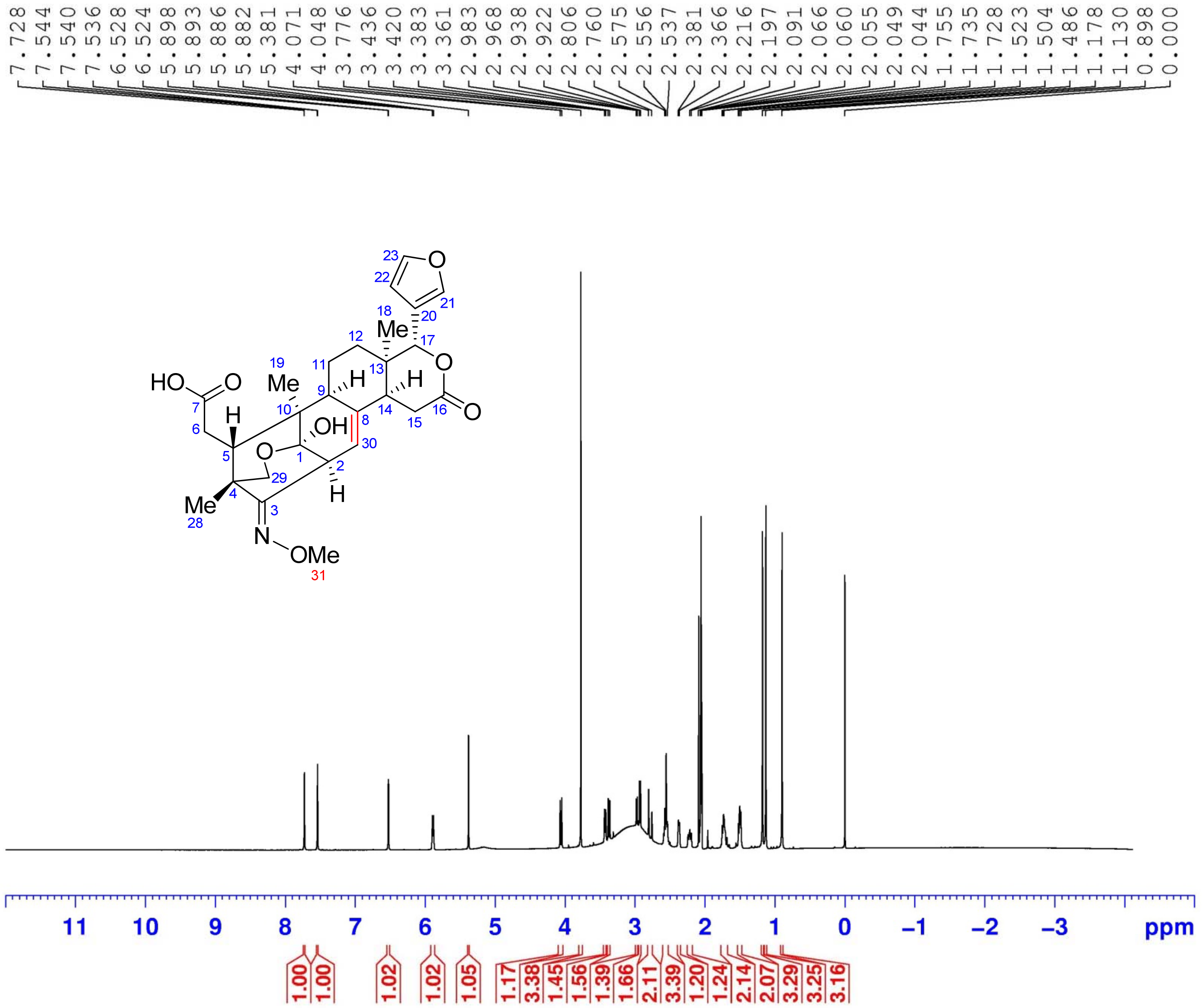

NAME	czc-1-IBX-jiayang
EXPNO	1
PROCNO	1
Date_	20170706
Time	23.25
INSTRUM	spect
PROBHD	5 mm CPPBBO BB
PULPROG	zg30
TD	65536
SOLVENT	Acetone
NS	16
DS	2
SWH	8223.685 Hz
FIDRES	0.125483 Hz
AQ	3.9846387 sec
RG	208.5
DW	60.800 usec
DE	10.00 usec
TE	297.0 K
D1	1.00000000 sec
TD0	1

======== CHANNEL f1 ========

SFO1	400.1324710 MHz
NUC1	1H
P1	11.50 usec
SI	65536
SF	400.1300049 MHz
WDW	EM
SSB	0
LB	0.30 Hz
GB	0
PC	1.00

^{1}H NMR (400 MHz) spectrum of **10b** in $(CD_3)_2CO$

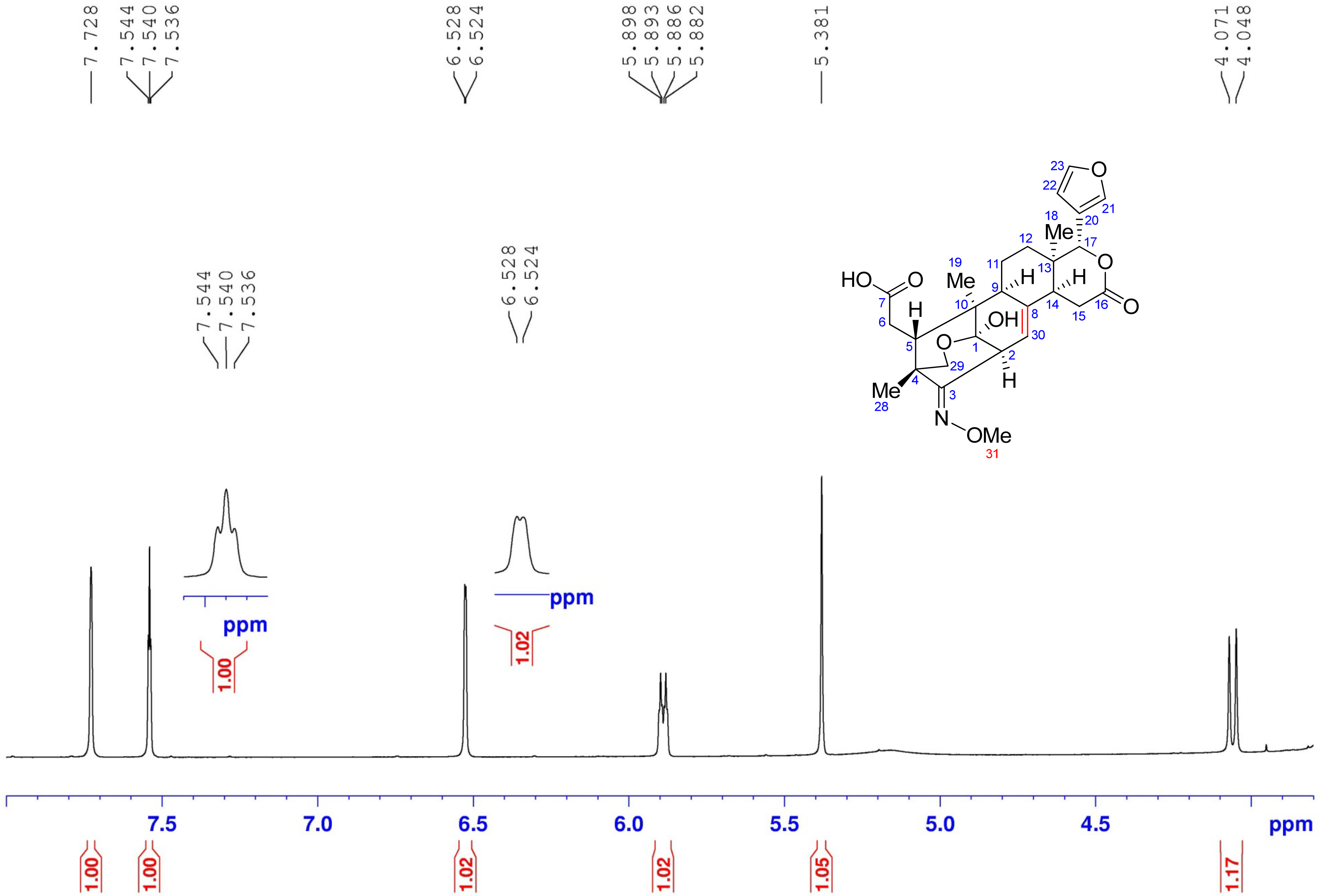

^{1}H NMR (400 MHz) spectrum of **10b** in $(CD_3)_2CO$

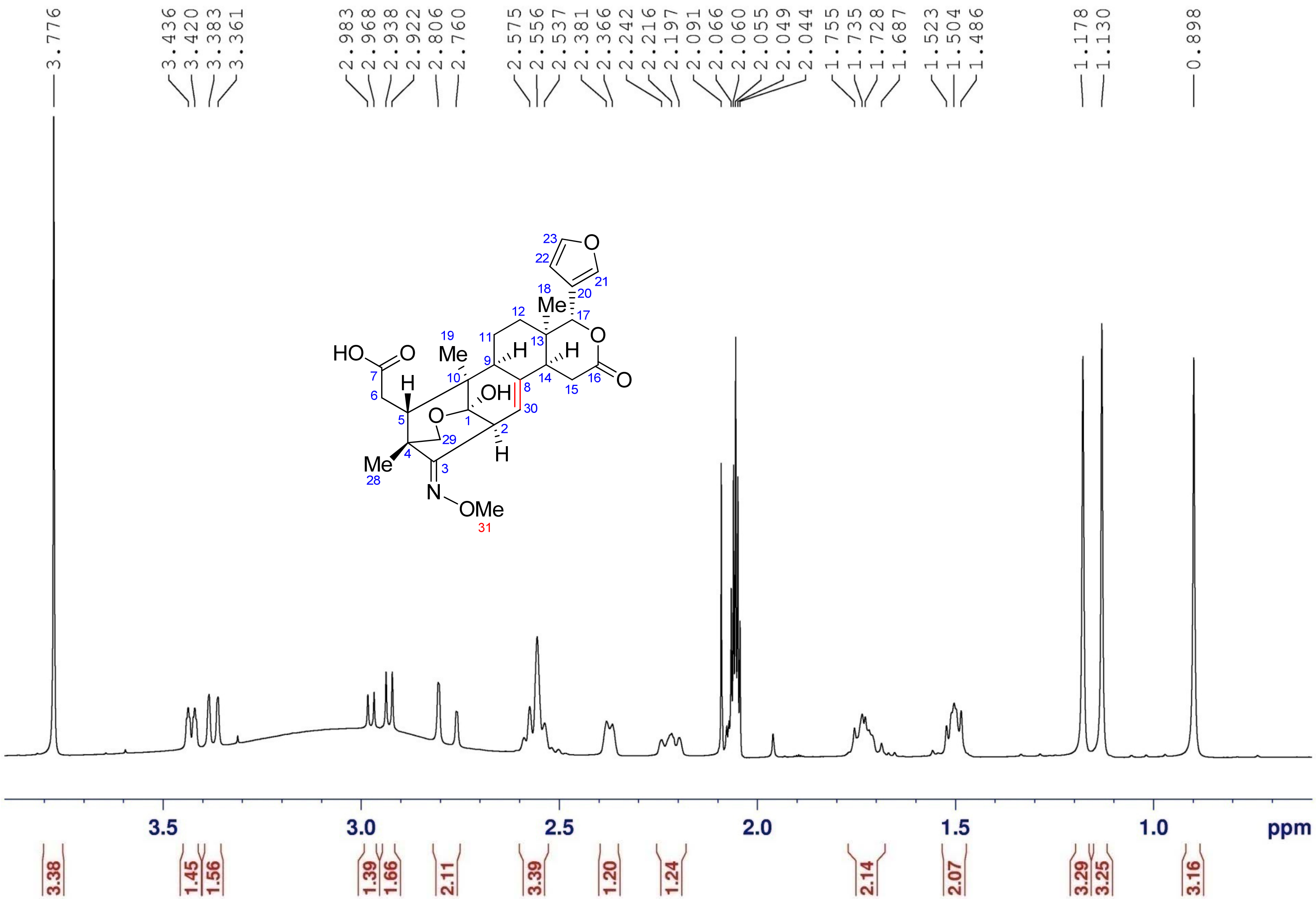

^{13}C NMR (100 MHz) spectrum of **10b** in $(CD_3)_2CO$

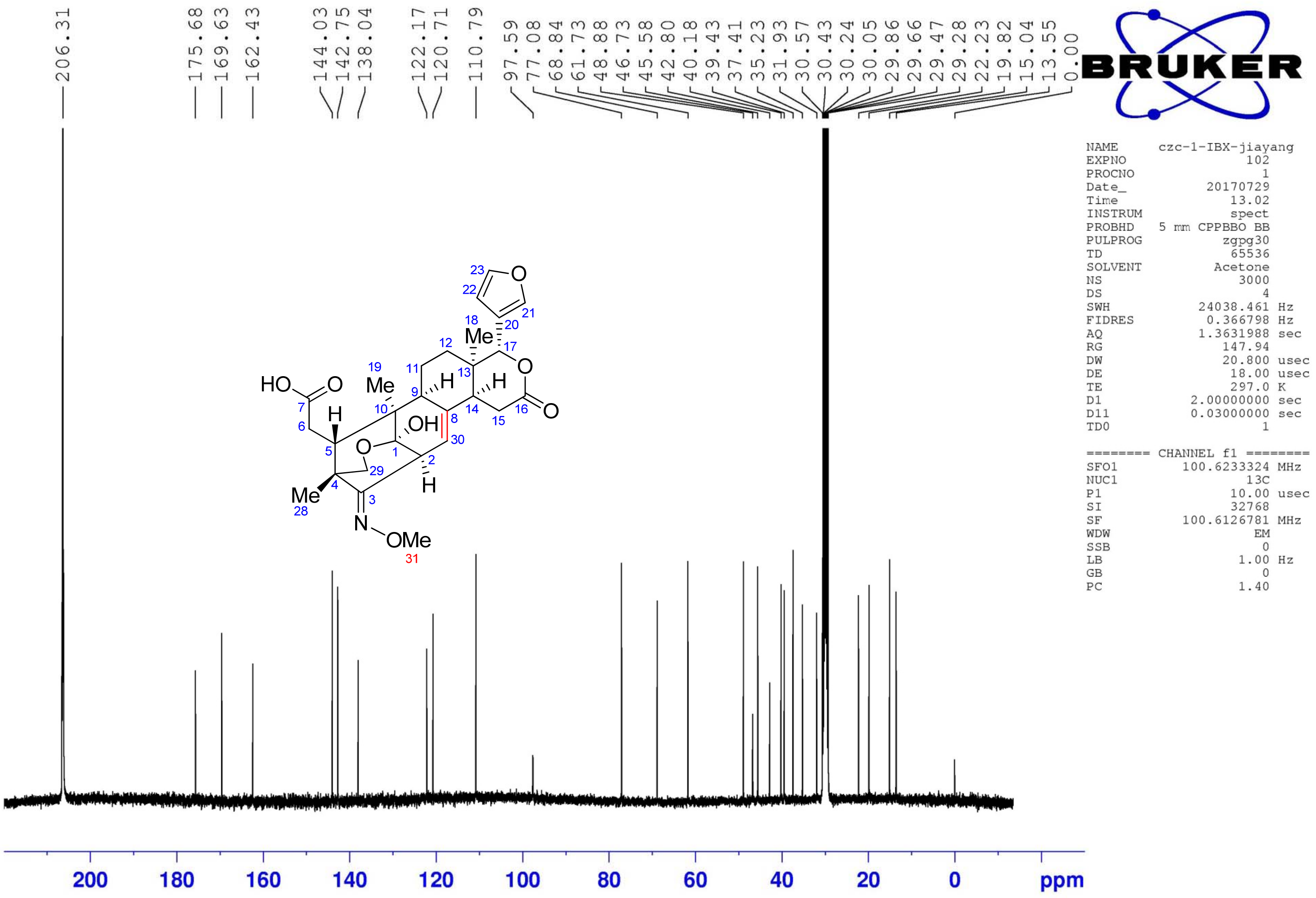

^{13}C NMR (100 MHz) spectrum of **10b** in $(CD_3)_2CO$

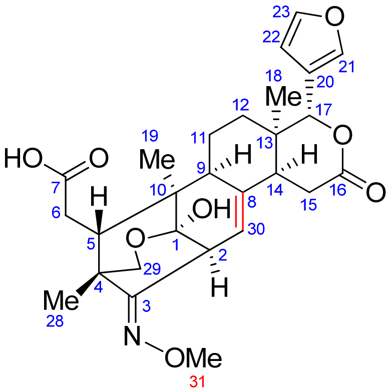

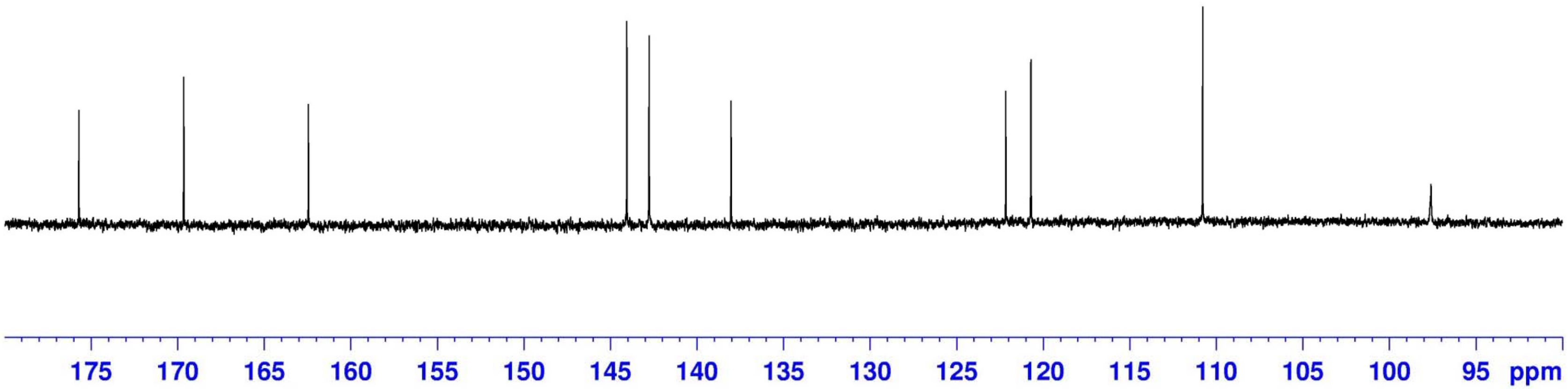

^{13}C NMR (100 MHz) spectrum of **10b** in $(CD_3)_2CO$

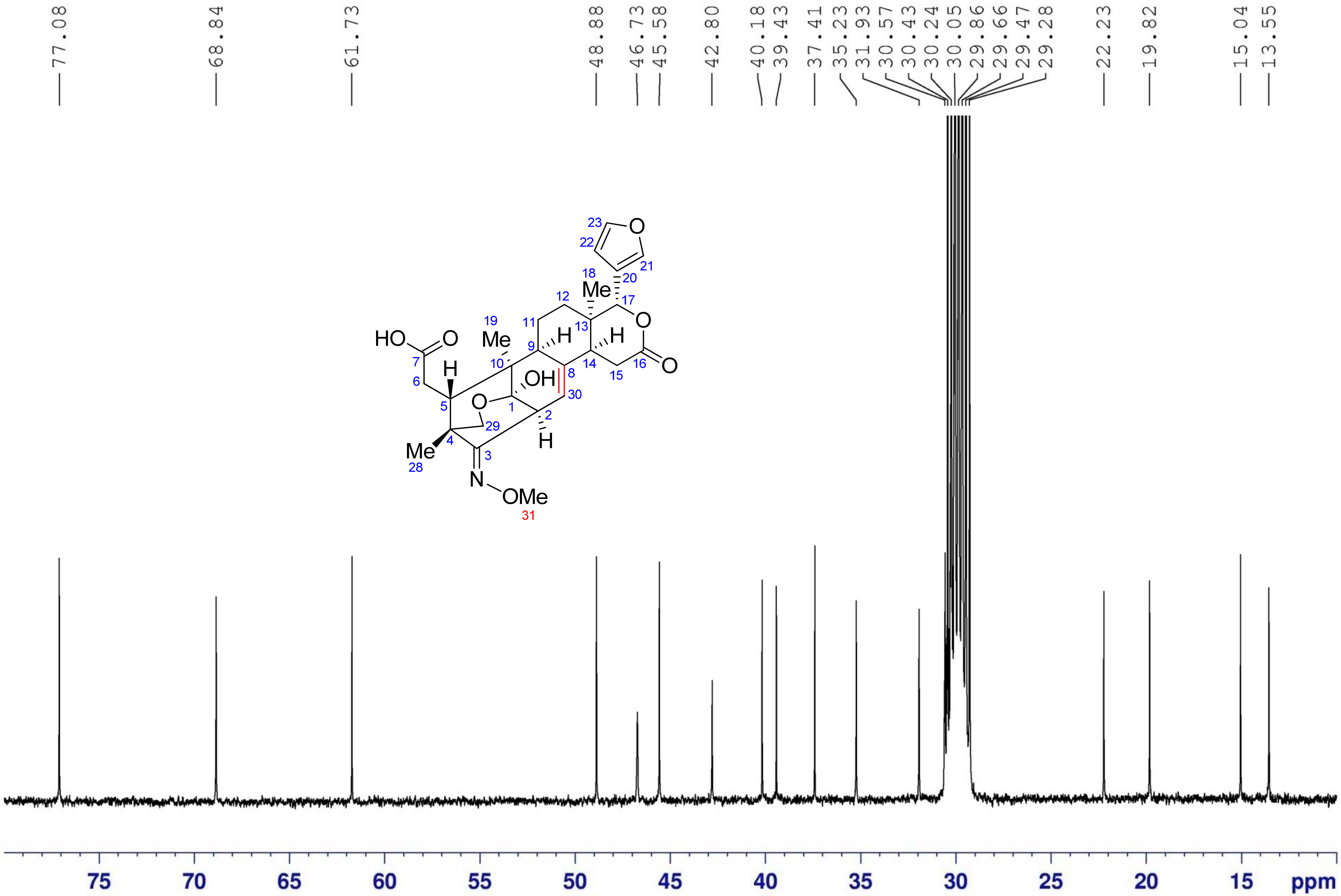

DEPT 135 spectrum of **10b** in $(CD_3)_2CO$

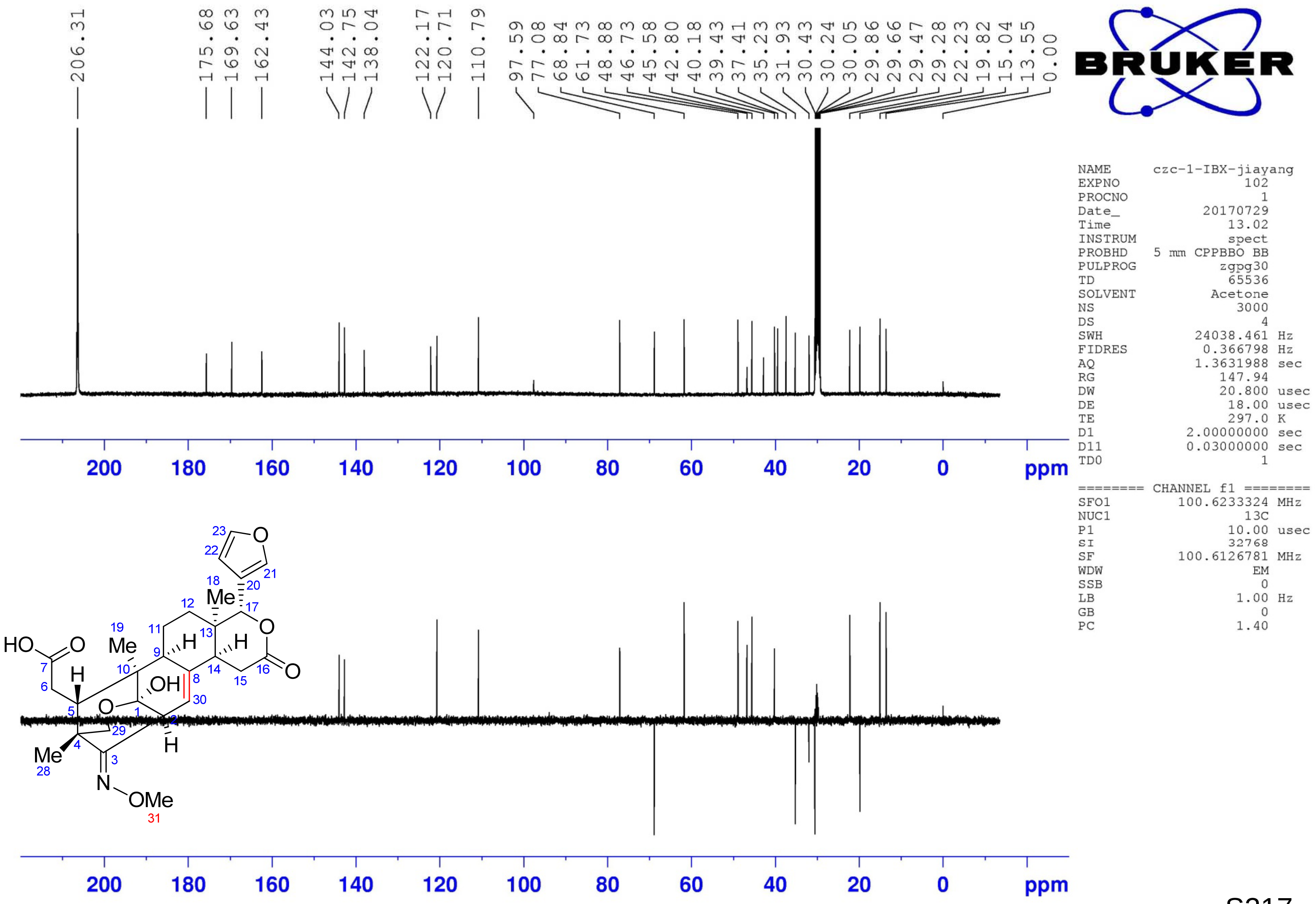

DEPT 135 spectrum of **10b** in $(CD_3)_2CO$

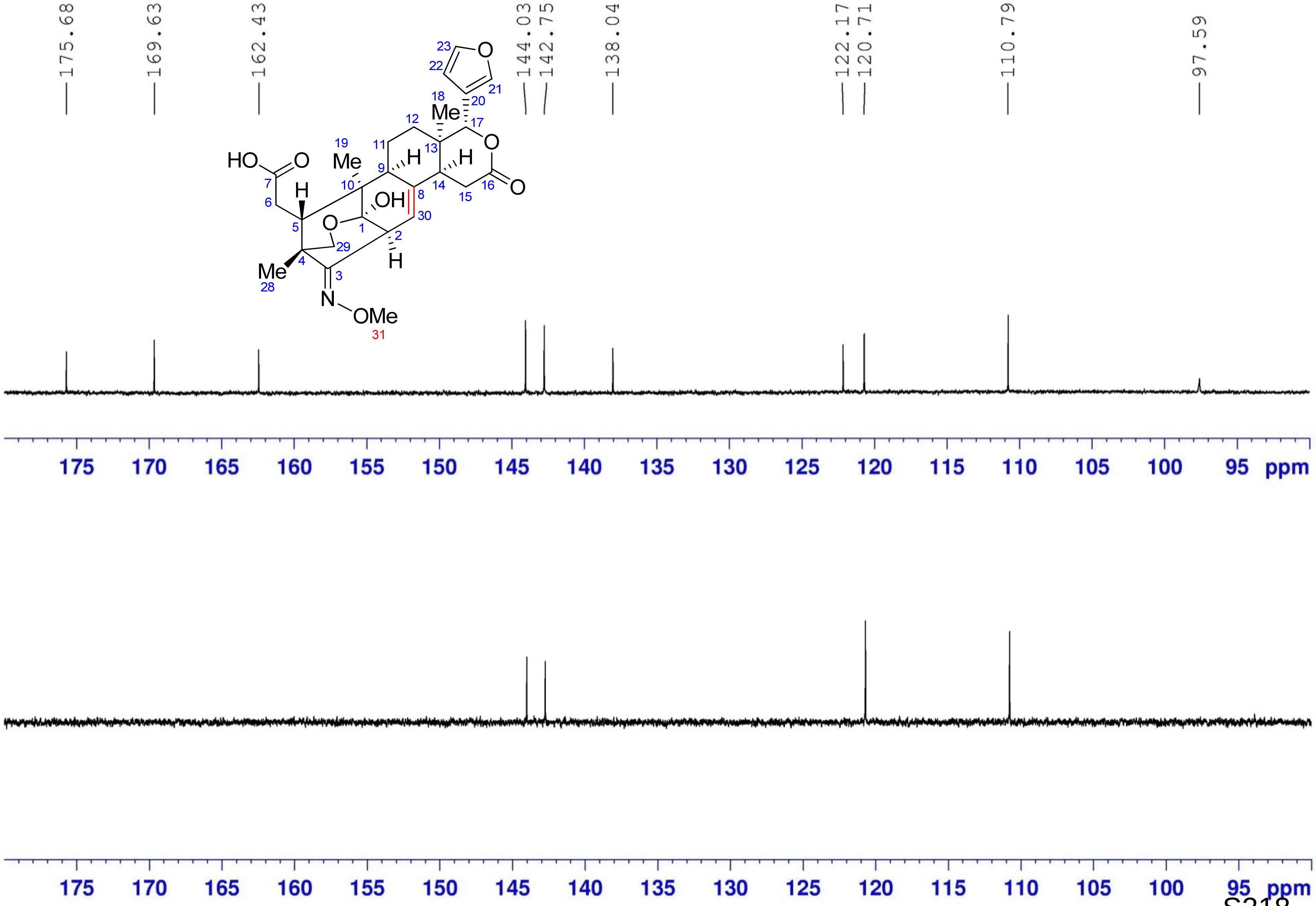

DEPT 135 spectrum of **10b** in $(CD_3)_2CO$

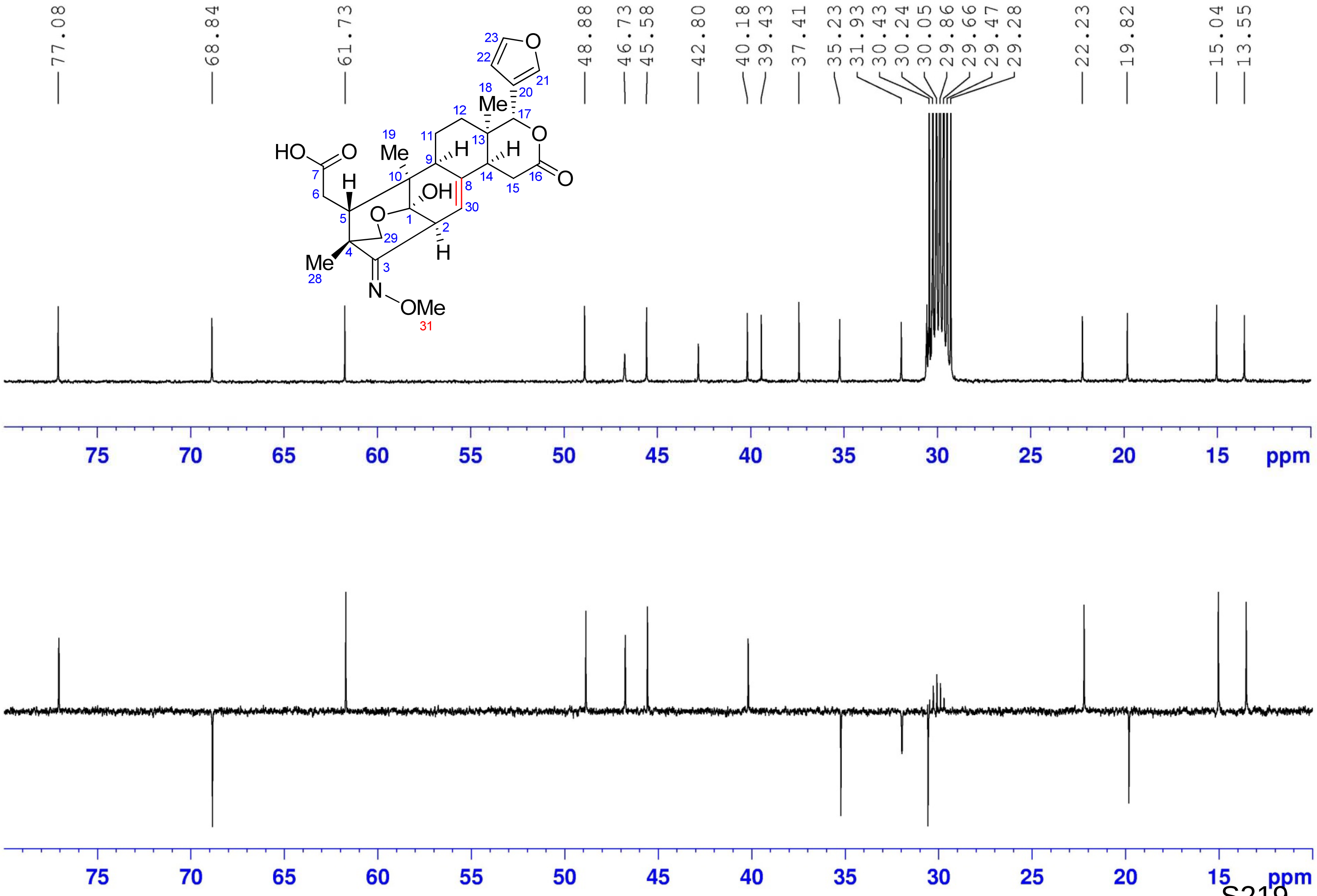

LR-ESIMS for compound **10c**

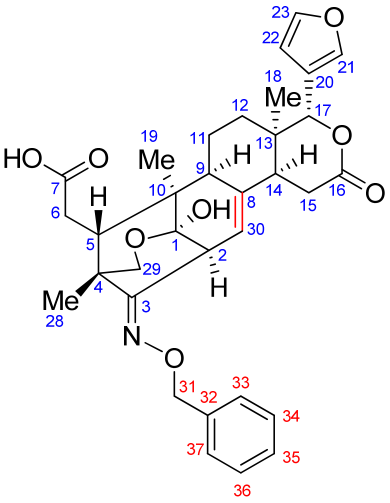

Generic Display Report

Analysis Info		Acquisition D	2018/10/1 18:25:28
Analysis Name	D:\MS\RJL\20181001\77_RA1_01_1589.d		
Method	lc-7.9	Operator	bruker
Sample Name	7720140922-alternating-500-50-2200-MS2_1589.m	Instrument	amaZon SL
Comment			

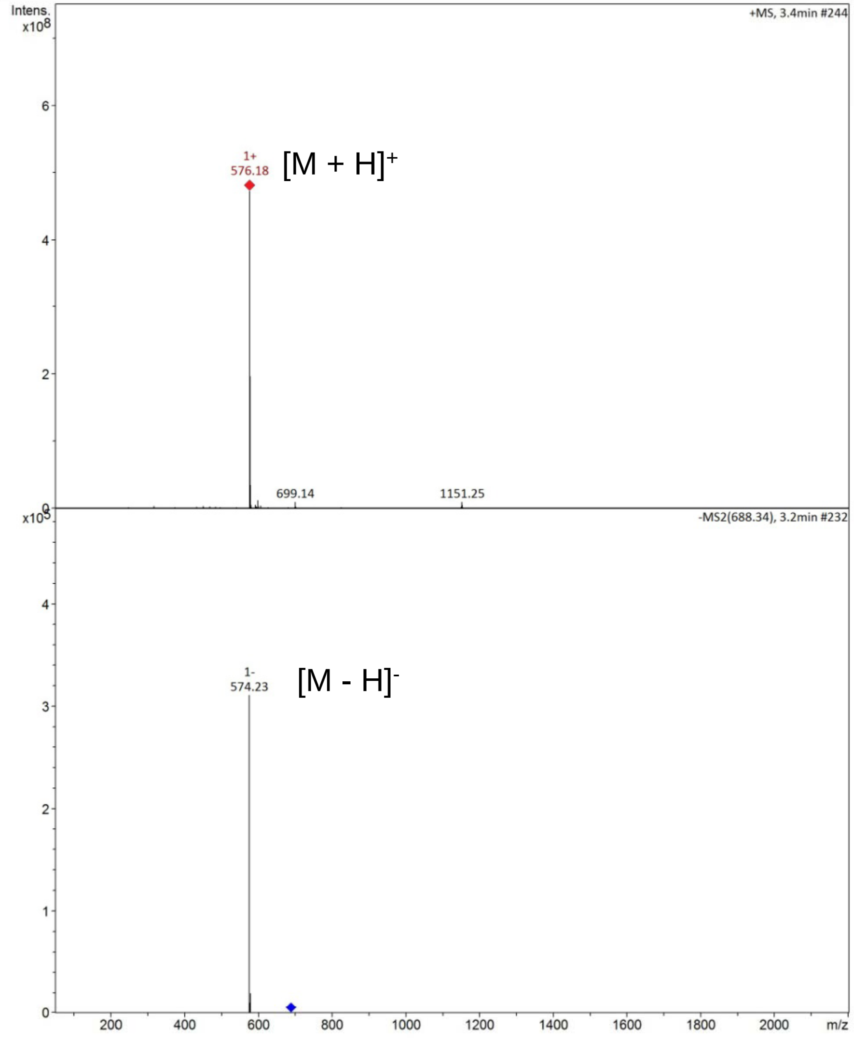

Bruker Compass DataAnalysis

^{1}H NMR (400 MHz) spectrum of **10c** in $(CD_3)_2CO$

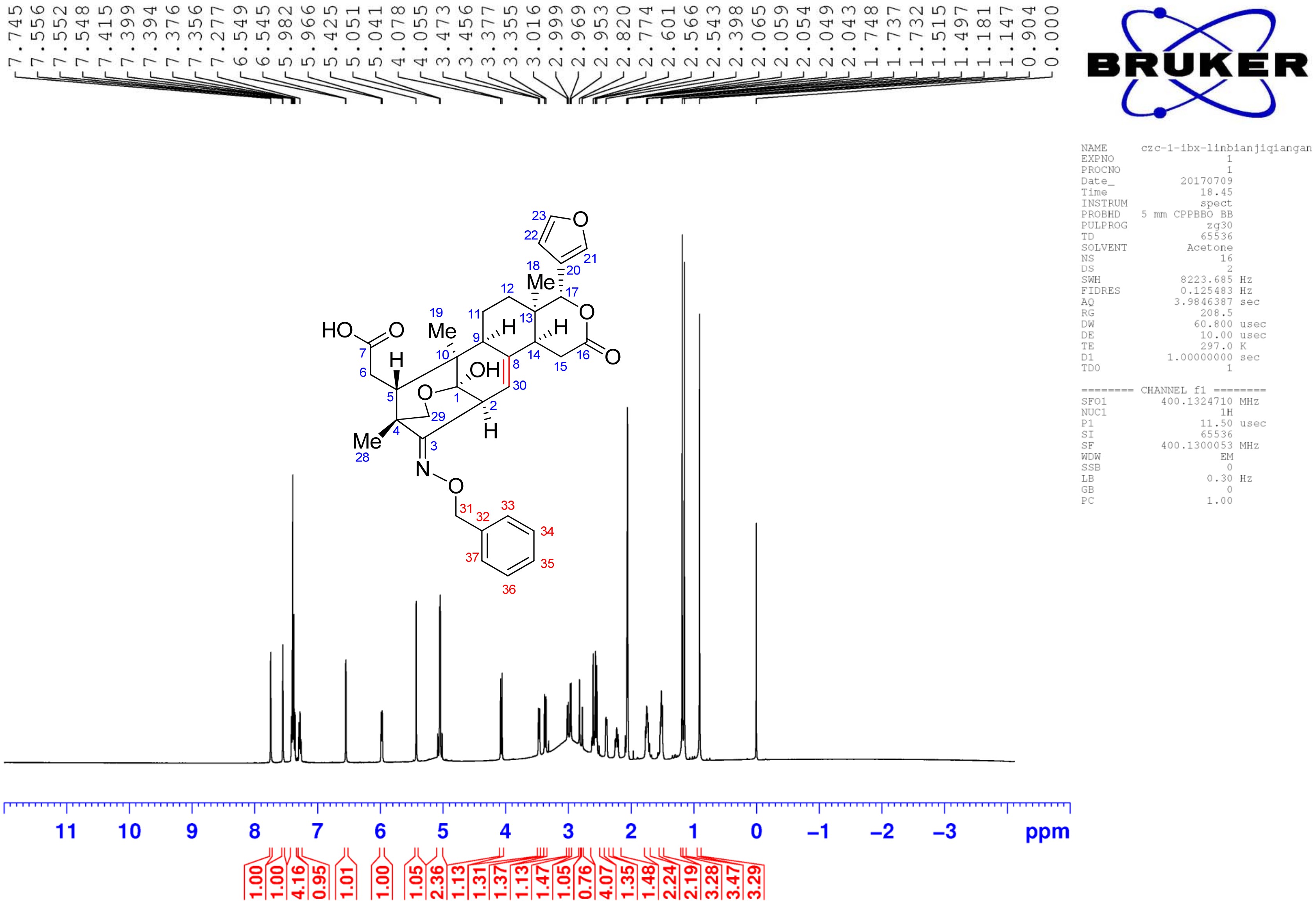

^{1}H NMR (400 MHz) spectrum of **10c** in $(CD_3)_2CO$

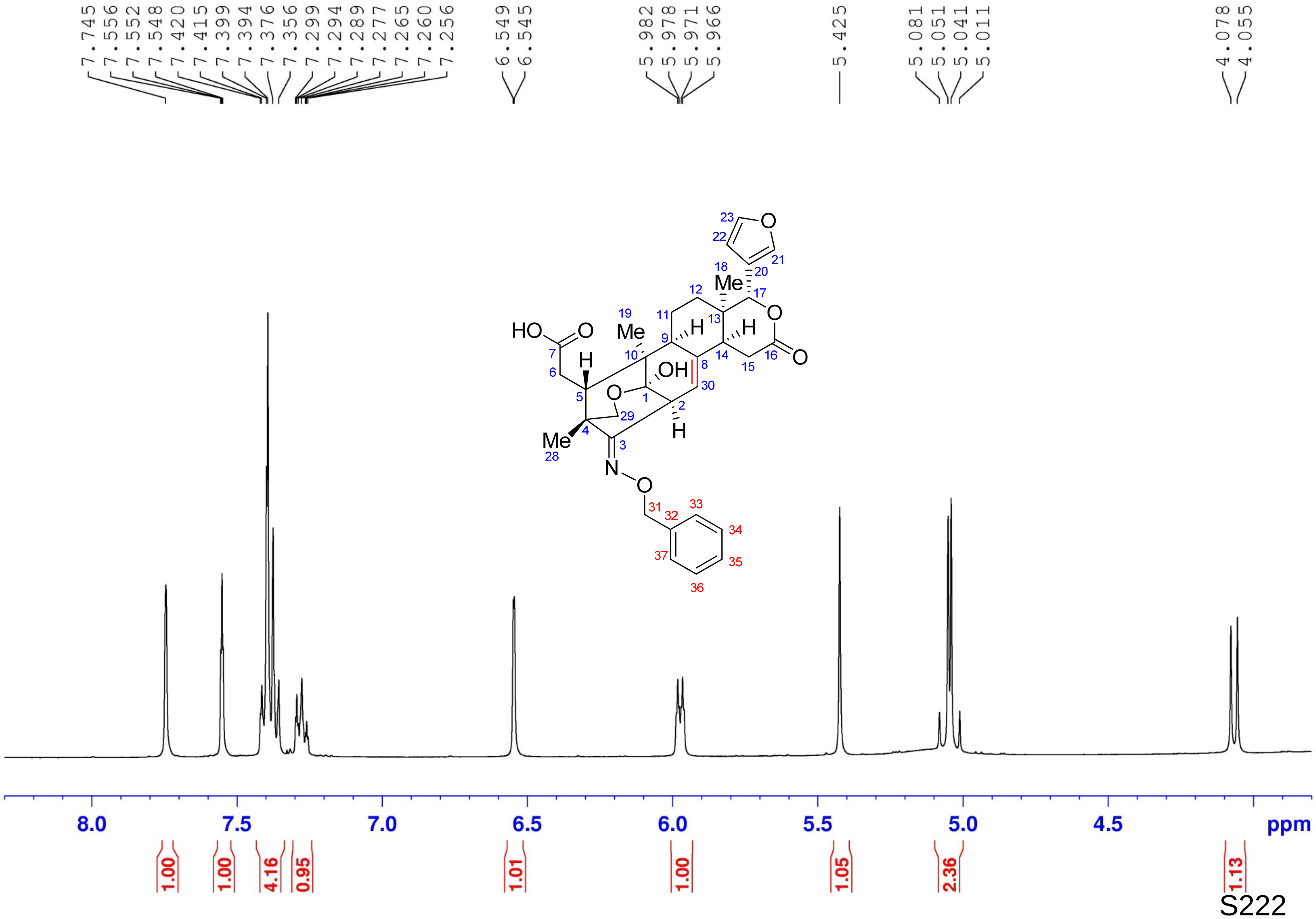

^{1}H NMR (400 MHz) spectrum of **10c** in $(CD_3)_2CO$

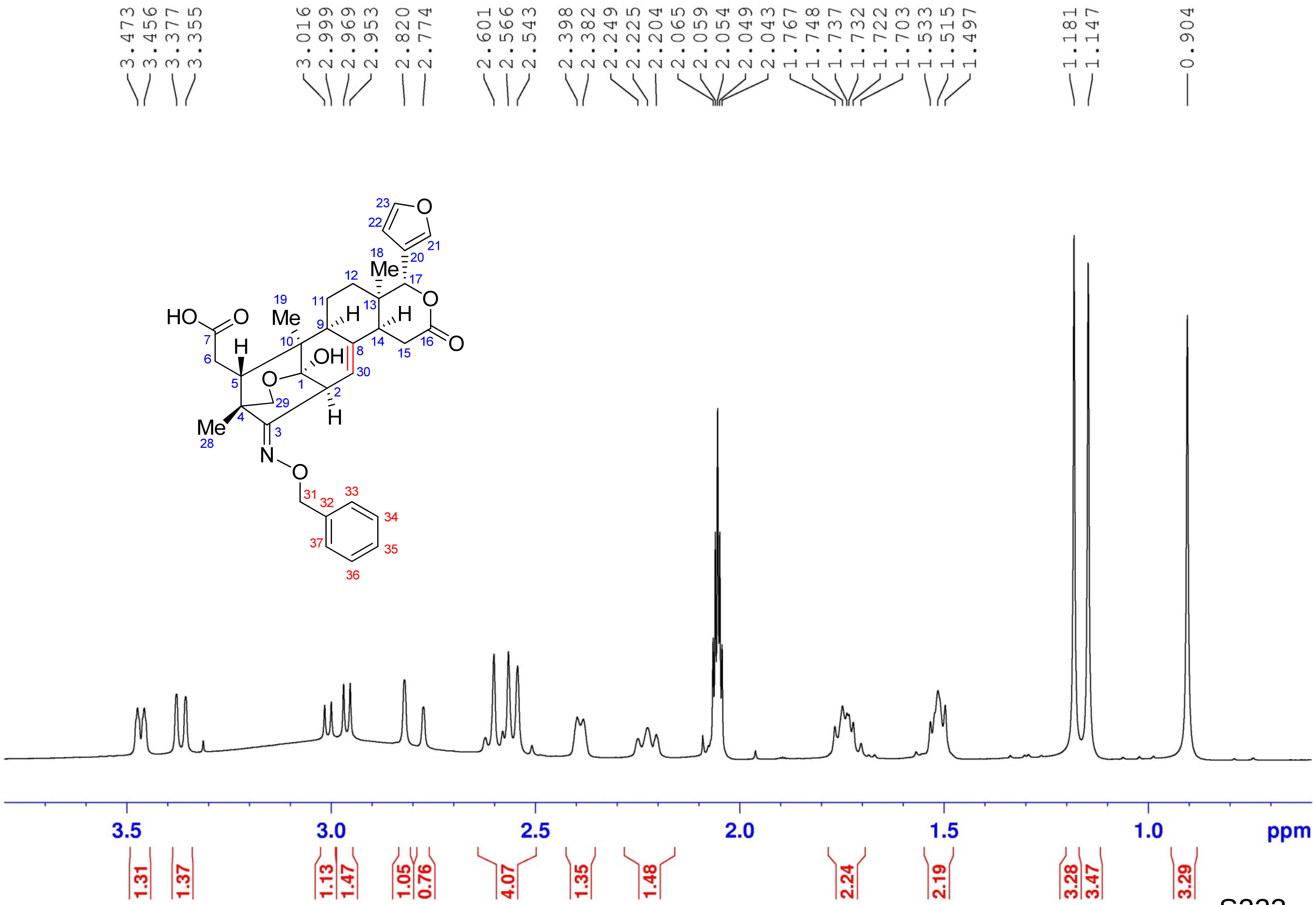

^{13}C NMR (100 MHz) spectrum of **10c** in $(CD_3)_2CO$

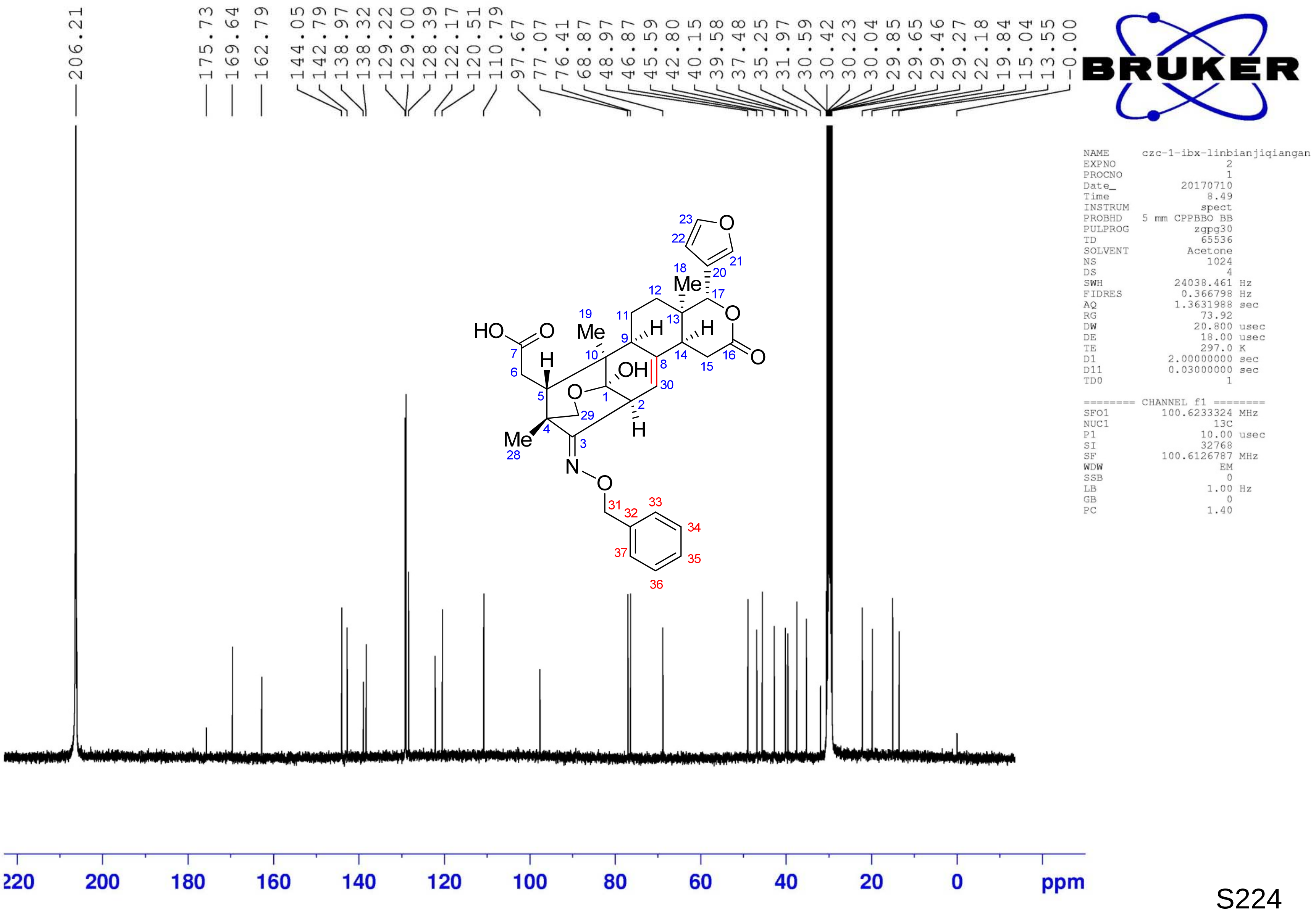

^{13}C NMR (100 MHz) spectrum of **10c** in $(CD_3)_2CO$

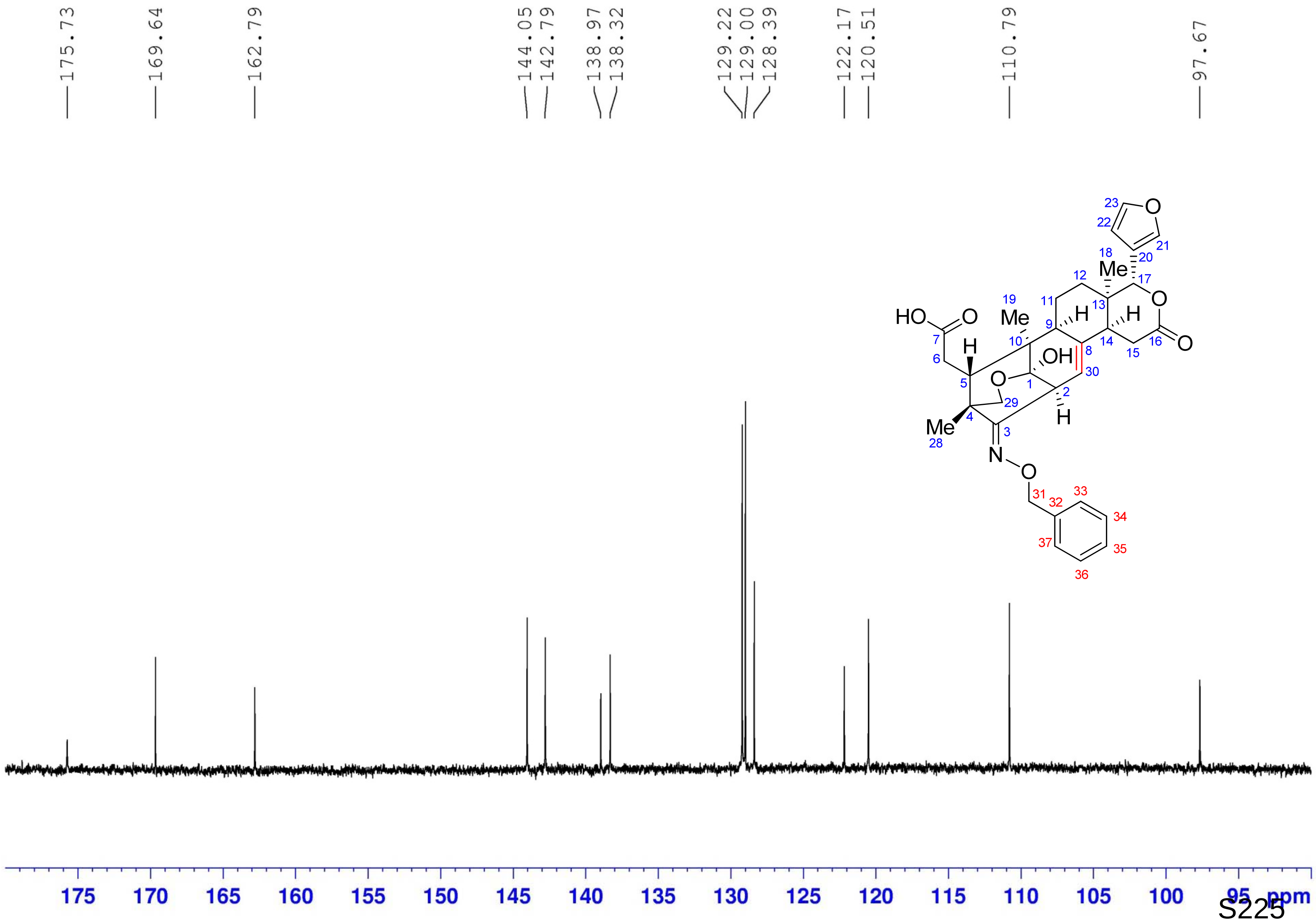

^{13}C NMR (100 MHz) spectrum of **10c** in $(CD_3)_2CO$

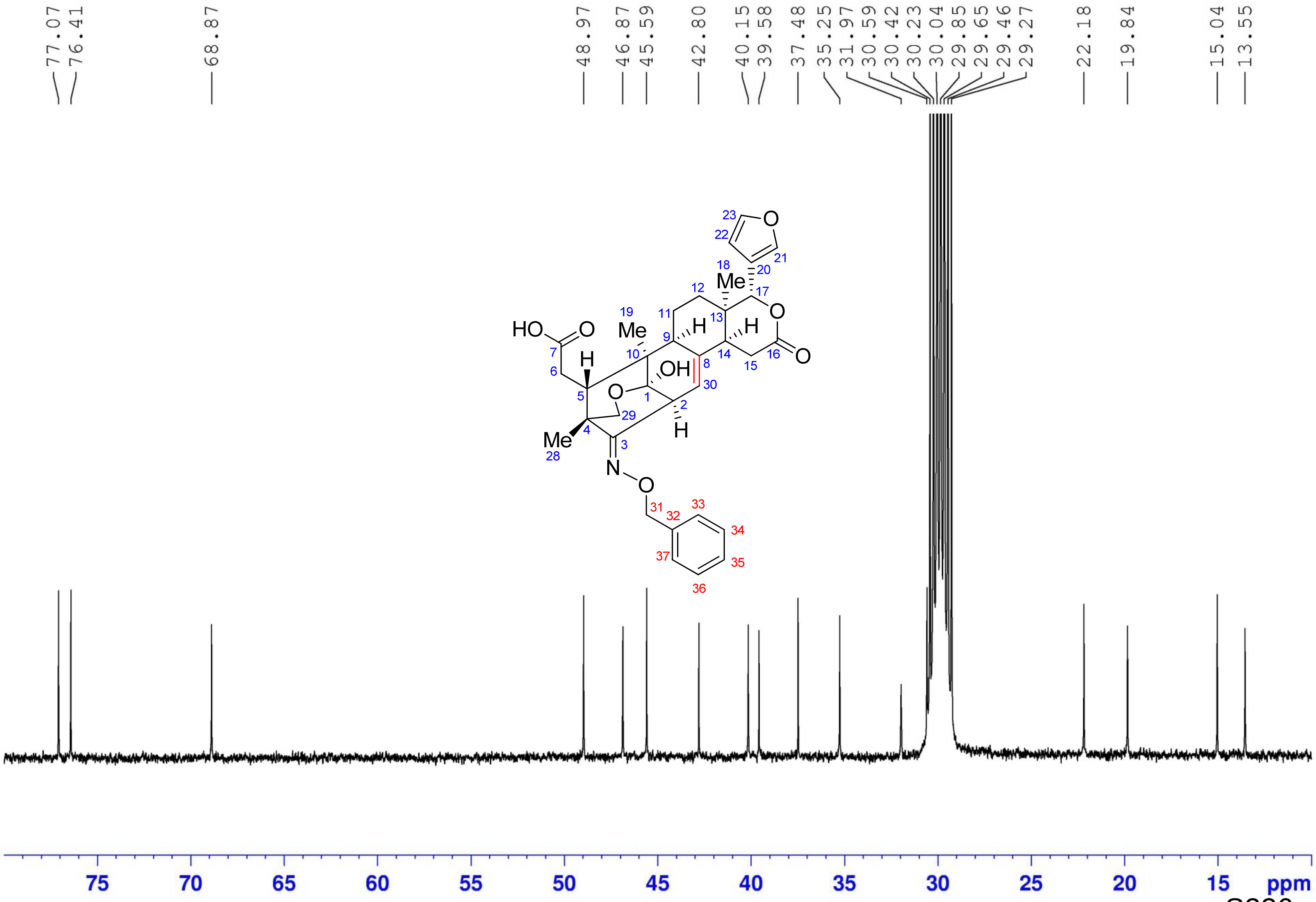

DEPT 135 spectrum of **10c** in $(CD_3)_2CO$

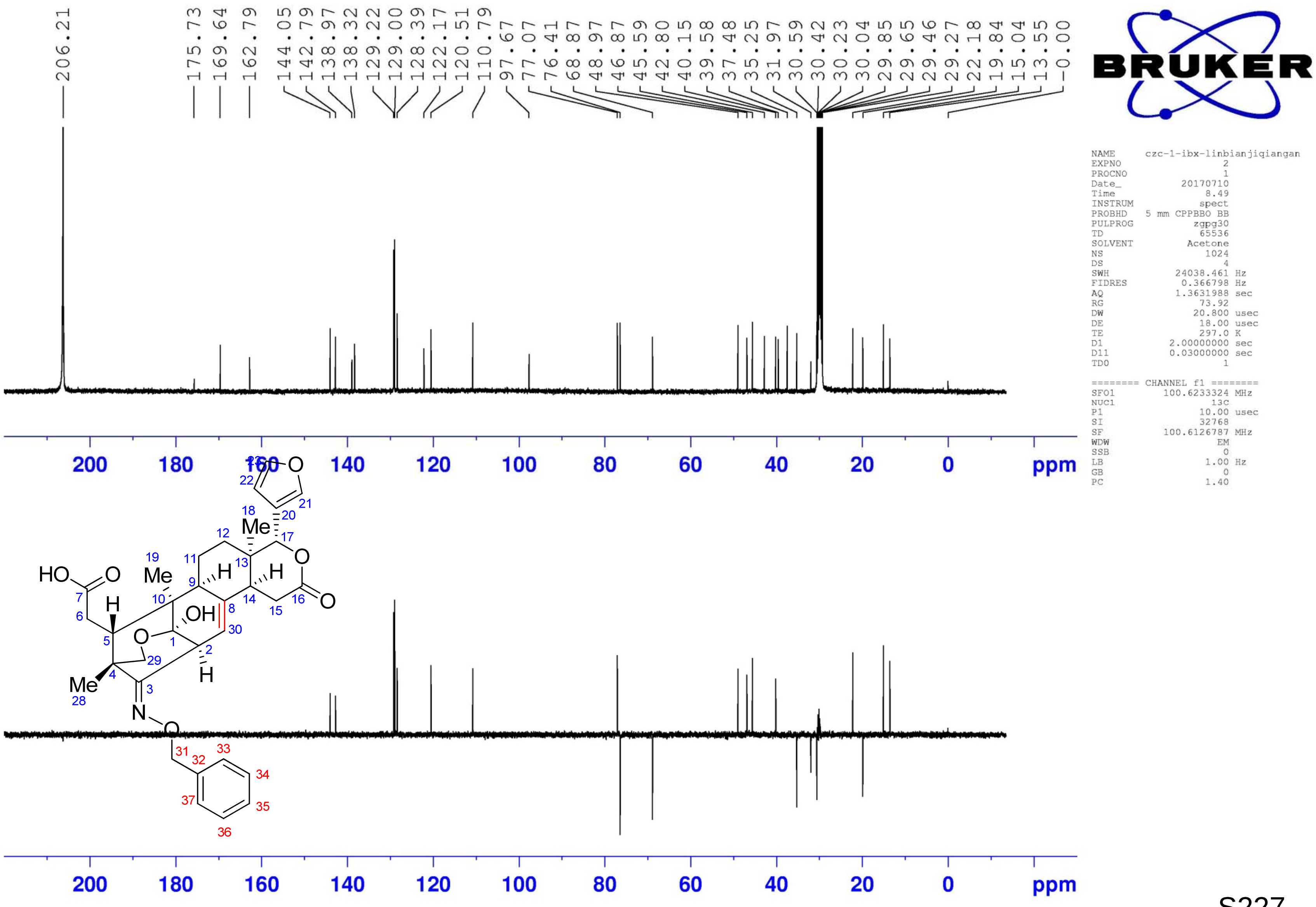

DEPT 135 spectrum of **10c** in $(CD_3)_2CO$

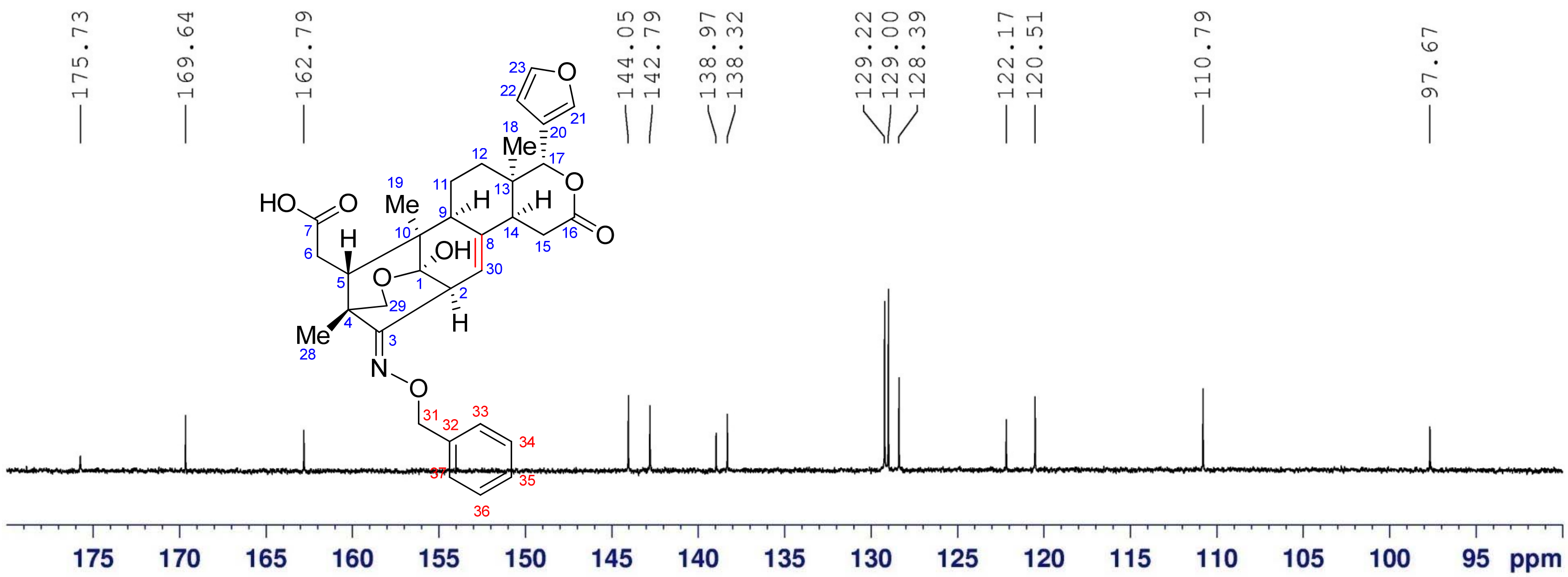

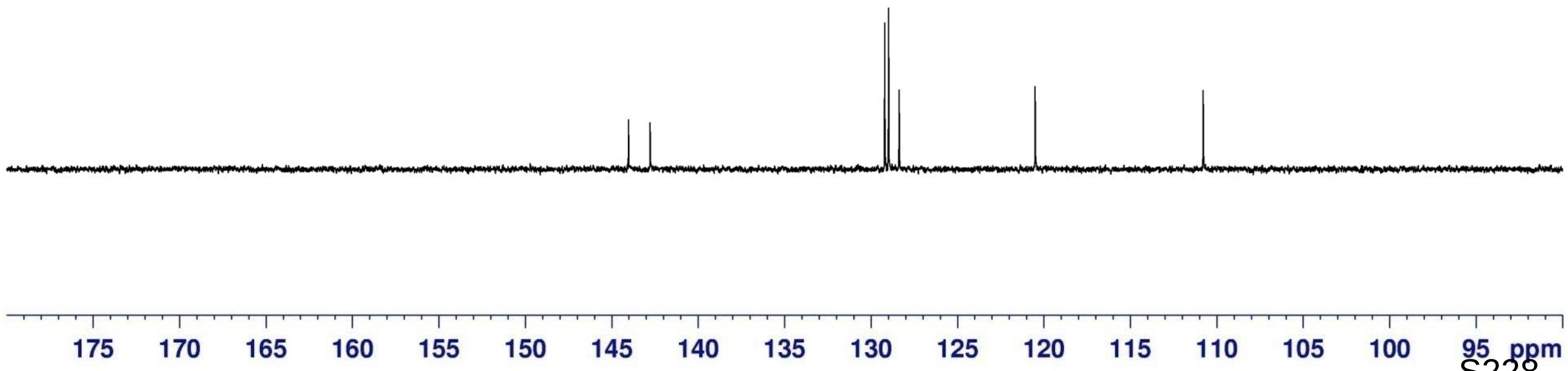

DEPT 135 spectrum of **10c** in $(CD_3)_2CO$

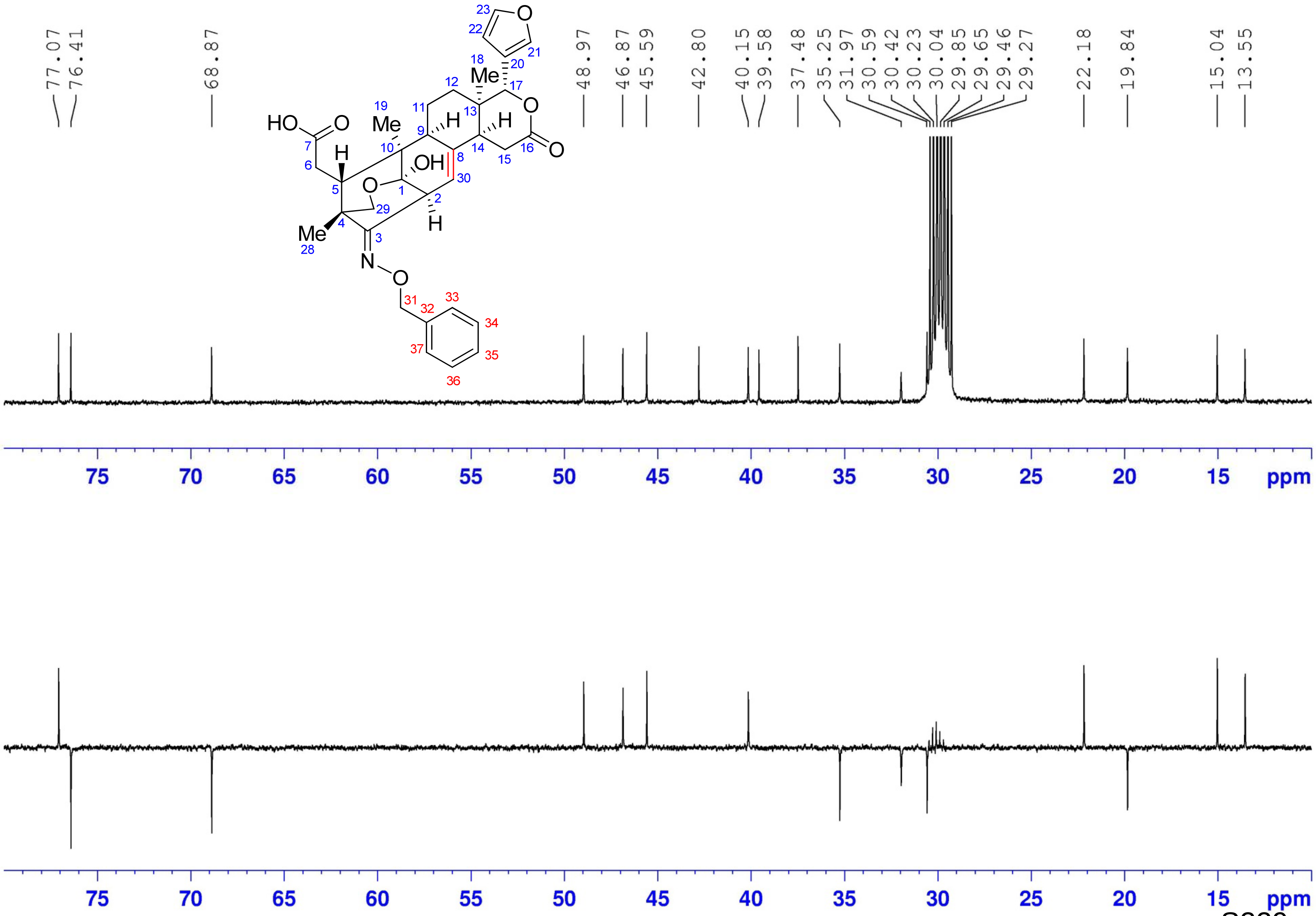

Supplement: Supplementary file 1 [file marinedrugs-16-00434-s001.zip › SupportingInformation-files/SI-II.pdf]
